# Supplementary material for: Systematic approach to outcome assessment from coded electronic healthcare records in the DaRe2THINK NHS-embedded randomized trial
Source: Eur Heart J Digit Health. 2022 Sep 16;3(3):426–36. doi: 10.1093/ehjdh/ztac046 (PMC9708037; doi:10.1093/ehjdh/ztac046)
Supplement: ztac046_Supplementary_Data [file ztac046_supplementary_data.zip › D2Tcoding_Appendix1_codelists_23May2022.pdf]

Systematic approach to outcome assessment from coded electronic healthcare records in the DaRe2THINK NHS-embedded randomised trial

Appendix 1: DaRe2THINK code lists

Chapter 1. Code lists for selection criteria **3**

Table S1. Atrial fibrillation Aurum codes 3

Table S2. Anticoagulant Aurum product codes 5

Table S3. Low molecular weight heparin Aurum product codes 7

Table S4. Stroke Aurum codes 10

Table S5. Transient ischaemic attack selection Aurum codes 13

Table S6. Arterial thromboembolism Aurum codes 14

Table S7. Myocadial infarction Aurum codes 17

Table S8. Periperal arterial disease Aurum codes 20

Table S9. Aortic plaque Aurum codes 21

Table S10. Diabetes Aurum codes 22

Table S11. Anti-Diabetic Aurum product codes 35

Table S12. Hypertension Aurum codes 51

Table S13. Anti-Hypertension Aurum product codes 54

Table S14. Heart failure selection Aurum codes 77

Table S15. Loop Diuretics Aurum product codes 79

Table S16. Gastrointestinal and intracranial Bleeding Aurum codes 82

Table S17. Gastrointestinal tract ulcer Aurum codes 87

Table S18. Brain injury Aurum codes 94

Table S19. Spinal Injury Aurum codes 114

Table S20. Eye Injury Aurum codes 115

Table S21. Estimated glomerular filtration rate Aurum codes 116

Table S22. Azole Aurum codes 117

Table S23. Dementia Aurum codes 118

Chapter 2. Code lists for trial outcomes **124**

Table S24. Cardivascular mortality HES (ICD10) codes 124

Table S25. Ischaemic stroke Aurum codes 125

Table S26. Ischaemic stroke HES (ICD10) codes 128

Table S27. Transient ischaemic attack outcome Aurum codes 129

Table S28. Transient ischaemic attack HES (ICD10) codes 131

Table S29. Venous thromoemoblism and pulmonary embolism Aurum codes 132

Table S30. Arterial thromboembolic Aurum codes 145

Table S31. Thromboembolic HES (ICD10) codes 153

Table S32. Myocardial infarction outcome Aurum codes 155

Table S33. Myocardial infarction HES (ICD10) codes 159

Table S34. Vascular dementia Aurum codes 160

Table S35. Vascular dementia HES (ICD10) codes 162

Table S36. Gastrointestinal bleeding Aurum codes 163

Table S37. Gastrointestinal bleeding HES (ICD10) codes ... 168
Table S38. Bleeding at other anatomy sites Aurum codes ... 169
Table S39. Bleeding at other anatomy sites HES (ICD10) codes ... 188
Table S40. Hospitalisation Aurum codes ... 190
Table S41. Intracranial haemorrhage Aurum codes ... 199
Table S42. Intracranial haemorrhage HES (ICD10) codes ... 205
Table S43. Heart failure outcome Aurum codes ... 206
Table S44. Heart failure HES (ICD10) codes ... 209
Chapter 3. Code lists for baseline characteristics ... 210
Table S45. Ethinicity Aurum codes ... 210
Table S46. Smoking Aurum codes ... 219
Table S47. Measurements Aurum codes ... 222
Table S48. Blood test Aurum codes ... 224
Table S49. Atrial ablation Aurum codes ... 228
Table S50. Hyperthyoidism Aurum codes ... 230
Table S51. Hypothyroidism Aurum codes ... 232
Table S52. Chronic obstructive pulmonary disease Aurum codes ... 235
Table S53. Diabetic retinopathy Aurum codes ... 241
Table S54. Retinal vascular occlusion Aurum codes ... 243
Table S55. Glaucoma Aurum codes ... 244
Table S56. Age-related macular degeneration Aurum codes ... 248
Table S57. Retinal Laser Aurum codes ... 249
Table S58. Vitrectomy Aurum codes ... 250
Table S59. Scleral buckle Aurum codes ... 251
Table S60. Intravitreal injection Aurum codes ... 252
Table S61. Antiplatelet Aurum product codes ... 253
Table S62. Thiazide diuretics Aurum product codes ... 256
Table S63. Thiazide like diuretics Aurum product codes ... 261
Table S64. Potassium sparing diuretics Aurum product codes ... 262
Table S65. Dihydropyridines Aurum product codes ... 264
Table S66. Non Dihydropyridines Aurum product codes ... 269
Table S67. Angiotensin converting enzyme inhibitors Aurum product codes ... 273
Table S68. Angiotensin II receptor blocker Aurum product codes ... 275
Table S69. Beta blockers Aurum product codes ... 279
Table S70. Alpha blockers Aurum product codes ... 283
Table S71. Aldosterone Aurum product codes ... 285
Table S72. Other antihypertensive Aurum product codes ... 286
Table S73. Antiarrhythmic drugs class 1 Aurum product codes ... 289
Table S74. Antiarrhythmic drugs class 3 Aurum product codes ... 292
Table S75. Digoxin Aurum product codes ... 293
Table S76. Sodium-glucose co-transporter-2 inhibitors Aurum product codes ... 297
Table S77. Nonsteroidal anti-inflammatory drug Aurum product codes ... 299

Chapter 1. Code lists for selection criteria

Table S1. Atrial fibrillation Aurum codes

Term	MedCode ID	SNOMED CT Concept ID	SNOMED CT DescriptionID
AF - Atrial fibrillation	3299911000006116	49436004	1230726010
AF - Paroxysmal atrial fibrillation	5669591000006113	282825002	421232012
Atrial fibrillation	82343012	49436004	82343012
Atrial fibrillation and flutter	300130013	195080001	300130013
Atrial fibrillation and flutter NOS	300132017	195080001	300130013
Atrial fibrillation annual review	406861000000119	248411000000105	406861000000119
Atrial fibrillation confirmed	1823951000006111	1823951000006107	1823951000006111
Atrial fibrillation detected	8439861000006115	1066831000000104	2679661000000115
Atrial fibrillation monitoring first letter	408081000000116	717221000000101	1569761000000112
Atrial fibrillation monitoring invitation	8214691000006119	711411000000101	1555841000000110
Atrial fibrillation monitoring second letter	408101000000110	716981000000106	1569531000000111
Atrial fibrillation monitoring telephone invitation	8218281000006110	716721000000107	1569271000000111
Atrial fibrillation monitoring telephone invite	408241000000115	716721000000107	1570381000000119
Atrial fibrillation monitoring third letter	408121000000118	716181000000109	1568741000000115
Atrial fibrillation monitoring verbal invitation	8218591000006111	717011000000100	1569561000000118
Atrial fibrillation monitoring verbal invite	408141000000113	717011000000100	1570401000000119
Atrial fibrillation resolved	294601000000110	196371000000102	294601000000110
Atrial fibrillation with rapid ventricular response	8037691000006113	120041000119109	2981207015
Chronic atrial fibrillation	2675253013	426749004	2675253013
Controlled atrial fibrillation	5887651000006116	300996004	442207015
ECG: atrial fibrillation	256478018	164889003	256478018
Electrocardiographic atrial fibrillation	4586611000006112	164889003	3300048013
Evaluation of AF (atrial fibrillation) burden	12734511000006115	143601000000104	1628691000000119
Evaluation of atrial fibrillation rate control	12736851000006112	1127481000000104	1855311000000119
H/O: atrial fibrillation	456154015	312442005	456154015
History of atrial fibrillation	6016401000006115	312442005	2986292016
History of maze procedure for atrial fibrillation	7100011000006116	429218009	2695416012
Insertion of pacemaker for control of atrial fibrillation	7375931000006117	449863006	2912517016
Intermittent atrial fibrillation	5669611000006119	282825002	421234013
Lone atrial fibrillation	5057261000006110	233910005	350464013
Longstanding persistent atrial fibrillation	7610331000006110	706923002	3027950014
Maze procedure for atrial fibrillation	7099921000006111	429211003	2694655012
Non-rheumatic atrial fibrillation	350465014	233911009	350465014
NRAF - Non-rheumatic atrial fibrillation	5057281000006117	233911009	350466010
PAF - Paroxysmal atrial fibrillation	5669601000006117	282825002	421233019
Paroxysmal atrial fibrillation	421235014	282825002	421235014

Permanent atrial fibrillation	636721000000112	440028005	2793259018
Persistent atrial fibrillation	636701000000115	440059007	2793372019
QOF (Quality and Outcomes Framework) atrial fibrillation quality indicator-related care invitation	12626271000006110	1110851000000100	2779681000000117
QOF (Quality and Outcomes Framework) atrial fibrillation quality indicator-related care invitation using preferred method of communication	12734281000006112	133111000000106	1628151000000119
Rapid atrial fibrillation	6043781000006115	314208002	458527016
Transient cerebral ischaemia due to atrial fibrillation	7064691000006118	426814001	2675245017
Transient cerebral ischemia due to atrial fibrillation	7064701000006118	426814001	2675244018

Table S2. Anticoagulant Aurum product codes

Term from EMIS	Prod code ID	dmd ID	Drug substance name
warfarin wbp tablets	1531941000033113		
warfarin wbp tablets 1 mg	1532041000033119		
warfarin wbp tablets 3 mg	1532141000033115		
warfarin wbp tablets 5 mg	1532241000033110		
warfarin (evans) tablets 3 mg	1532341000033117		
sinthrome tablets 4 mg	1337441000033110		
warfarin 1mg tablets	1531641000033118	319733000	Warfarin sodium
marevan 1mg tablets (advanz pharma)	868141000033114	933311000001105	Warfarin sodium
marevan 3mg tablets (advanz pharma)	868241000033119	284011000001104	Warfarin sodium
warfarin 3mg tablets	1531741000033110	319734006	Warfarin sodium
warfarin 5mg tablets	1531841000033117	319735007	Warfarin sodium
marevan 5mg tablets (advanz pharma)	868341000033112	461911000001100	Warfarin sodium
marevan 500microgram tablets (advanz pharma)	1819741000033117	204811000001109	Warfarin sodium
warfarin 500microgram tablets	1819641000033114	319736008	Warfarin sodium
acenocoumarol 1mg tablets	3097541000033112	319740004	Acenocoumarol
sinthrome 1mg tablets (norgine pharmaceuticals ltd)	1337341000033116	738511000001103	Acenocoumarol
phenindione 10mg tablets	1081541000033110	319745009	Phenindione
phenindione 25mg tablets	1082341000033112	319746005	Phenindione
phenindione 50mg tablets	1082441000033118	319747001	Phenindione
coumadin 4mg tablets (imported (canada))	6112441000033111	18509011000001102	Warfarin sodium
apixaban 2.5mg tablets	6444441000033114	703907006	Apixaban
eliquis 2.5mg tablets (bristol-myers squibb pharmaceuticals ltd)	6444541000033110	19506911000001105	Apixaban
eliquis 5mg tablets (bristol-myers squibb pharmaceuticals ltd)	8233041000033112	21677511000001105	Apixaban
apixaban 5mg tablets	8232941000033119	703908001	Apixaban
warfarin 10mg/5ml oral suspension	2639041000033119	8798011000001109	Warfarin sodium
warfarin 1mg/5ml oral solution	6000541000033119	8798111000001105	Warfarin sodium
warfarin 1mg/5ml oral suspension	6000841000033117	8798211000001104	Warfarin sodium
warfarin 3mg/5ml oral solution	6000641000033118	8798511000001101	Warfarin sodium
warfarin 3mg/5ml oral suspension	6000941000033113	8798611000001102	Warfarin sodium
warfarin 5mg/5ml oral solution	6000741000033110	8798711000001106	Warfarin sodium
warfarin 5mg/5ml oral suspension	2620141000033114	8798811000001103	Warfarin sodium
dabigatran etexilate 110mg capsules	4500341000033115	13532811000001109	Dabigatran etexilate mesilate
pradaxa 110mg capsules (boehringer ingelheim ltd)	4500541000033110	13505411000001109	Dabigatran etexilate mesilate
dabigatran etexilate 75mg capsules	4500241000033113	13532911000001104	Dabigatran etexilate mesilate
pradaxa 75mg capsules (boehringer ingelheim ltd)	4500441000033114	13504711000001102	Dabigatran etexilate mesilate
rivaroxaban 10mg tablets	4656341000033115	14254711000001104	Rivaroxaban
xarelto 10mg tablets (bayer plc)	4656441000033114	14237311000001106	Rivaroxaban
warfarin 1mg/ml oral suspension sugar free	6066441000033118	18290011000001102	Warfarin sodium

dabigatran etexilate 150mg capsules	6436141000033111	19469811000001101	Dabigatran etexilate mesilate
pradaxa 150mg capsules (boehringer ingelheim ltd)	6436241000033116	19465811000001104	Dabigatran etexilate mesilate
rivaroxaban 15mg tablets	6511341000033117	19842111000001101	Rivaroxaban
xarelto 15mg tablets (bayer plc)	6511541000033112	19840811000001107	Rivaroxaban
xarelto 20mg tablets (bayer plc)	6511641000033113	19841411000001101	Rivaroxaban
rivaroxaban 20mg tablets	6511441000033111	19842211000001107	Rivaroxaban
xarelto 2.5mg tablets (bayer plc)	9704641000033115	27160311000001106	Rivaroxaban
rivaroxaban 2.5mg tablets	9704541000033116	27810711000001104	Rivaroxaban
lixiana 15mg tablets (daiichi sankyo uk ltd)	10493841000033112	29902111000001100	Edoxaban tosilate
edoxaban 15mg tablets	10493541000033110	29903211000001100	Edoxaban tosilate
edoxaban 30mg tablets	10493641000033111	29903311000001108	Edoxaban tosilate
lixiana 30mg tablets (daiichi sankyo uk ltd)	10493941000033116	29902411000001105	Edoxaban tosilate
lixiana 60mg tablets (daiichi sankyo uk ltd)	10494041000033119	29902711000001104	Edoxaban tosilate
edoxaban 60mg tablets	10493741000033119	29903411000001101	Edoxaban tosilate
xarelto 15mg / 20mg treatment initiation pack (bayer plc)	12407141000033118	34793111000001102	
rivaroxaban 15mg tablets and rivaroxaban 20mg tablets	12407041000033117	34819111000001102	

Table S3. Low molecular weight heparin Aurum product codes

Term from EMIS	Prod code ID	dmd ID	Drug substance name
Clexane 300mg/3ml solution for injection multidose vials (Sanofi)	4153241000033118	11507011000001102	Enoxaparin sodium
Enoxaparin sodium 300mg/3ml solution for injection vials	4153141000033113	11509311000001109	Enoxaparin sodium
Inhixa 300mg/3ml solution for injection multidose vials (Techdow Pharma England Ltd)	13711341000033114	38232211000001100	Enoxaparin sodium
Clexane Forte 120mg/0.8ml solution for injection pre-filled syringes (Sanofi)	2732541000033114	4198711000001100	Enoxaparin sodium
Enoxaparin sodium 120mg/0.8ml solution for injection pre-filled syringes	2041741000033113	36563911000001108	Enoxaparin sodium
Inhixa 120mg/0.8ml solution for injection pre-filled syringes (Techdow Pharma England Ltd)	12988541000033114	36784211000001100	Enoxaparin sodium
Arovi 120mg/0.8ml solution for injection pre-filled syringes (ROVI Biotech Ltd)	12584741000033119	35559211000001107	Enoxaparin sodium
Clexane 100mg/1ml solution for injection pre-filled syringes (Sanofi)	274941000033116	4199111000001108	Enoxaparin sodium
Arovi 100mg/1ml solution for injection pre-filled syringes (ROVI Biotech Ltd)	12584641000033111	35566911000001104	Enoxaparin sodium
Inhixa 100mg/1ml solution for injection pre-filled syringes (Techdow Pharma England Ltd)	12373141000033110	34701011000001102	Enoxaparin sodium
Enoxaparin Becat 100mg/1ml solution for injection pre-filled syringes (ROVI Biotech Ltd)	12476641000033118	35084011000001109	Enoxaparin sodium
Enoxaparin sodium 100mg/1ml solution for injection pre-filled syringes	516641000033117	36564011000001106	Enoxaparin sodium
Clexane 40mg/0.4ml solution for injection pre-filled syringes (Sanofi)	276541000033115	3825411000001104	Enoxaparin sodium
Enoxaparin sodium 40mg/0.4ml solution for injection pre-filled syringes	517141000033112	36565711000001107	Enoxaparin sodium
Enoxaparin Becat 40mg/0.4ml solution for injection pre-filled syringes (ROVI Biotech Ltd)	12476241000033116	35083411000001103	Enoxaparin sodium
Inhixa 40mg/0.4ml solution for injection pre-filled syringes (Techdow Pharma England Ltd)	12372841000033114	34700211000001101	Enoxaparin sodium
Arovi 40mg/0.4ml solution for injection pre-filled syringes (ROVI Biotech Ltd)	12585041000033116	35569011000001109	Enoxaparin sodium
Clexane 60mg/0.6ml solution for injection pre-filled syringes (Sanofi)	275041000033116	4199811000001101	Enoxaparin sodium
Enoxaparin sodium 60mg/0.6ml solution for injection pre-filled syringes	516741000033114	38895011000001100	Enoxaparin sodium
Enoxaparin Becat 60mg/0.6ml solution for injection pre-filled syringes (ROVI Biotech Ltd)	12476341000033114	35083611000001100	Enoxaparin sodium
Inhixa 60mg/0.6ml solution for injection pre-filled syringes (Techdow Pharma England Ltd)	12372941000033118	34700511000001103	Enoxaparin sodium
Arovi 60mg/0.6ml solution for injection pre-filled syringes (ROVI Biotech Ltd)	12585141000033117	35568611000001106	Enoxaparin sodium
Clexane 80mg/0.8ml solution for injection pre-filled syringes (Sanofi)	275141000033117	4199511000001104	Enoxaparin sodium
Arovi 80mg/0.8ml solution for injection pre-filled syringes (ROVI Biotech Ltd)	12585241000033112	35567911000001101	Enoxaparin sodium
Inhixa 80mg/0.8ml solution for injection pre-filled syringes (Techdow Pharma England Ltd)	12373041000033111	34700811000001100	Enoxaparin sodium
Enoxaparin Becat 80mg/0.8ml solution for injection pre-filled syringes (ROVI Biotech Ltd)	12476441000033115	35083811000001101	Enoxaparin sodium
Enoxaparin sodium 80mg/0.8ml solution for injection pre-filled syringes	516841000033116	38895111000001104	Enoxaparin sodium
Clexane Forte 150mg/1ml solution for injection pre-filled syringes (Sanofi)	2732641000033110	4198211000001107	Enoxaparin sodium
Enoxaparin sodium 150mg/1ml solution for injection pre-filled syringes	2041941000033111	38895211000001105	Enoxaparin sodium

Arovi 150mg/1ml solution for injection pre-filled syringes (ROVI Biotech Ltd)	12584841000033112	35559411000001106	Enoxaparin sodium
Inhixa 150mg/1ml solution for injection pre-filled syringes (Techdow Pharma England Ltd)	12988641000033110	36784411000001101	Enoxaparin sodium
Clexane 20mg/0.2ml solution for injection pre-filled syringes (Sanofi)	276441000033116	3826311000001101	Enoxaparin sodium
Enoxaparin sodium 20mg/0.2ml solution for injection pre-filled syringes	517041000033113	38896011000001109	Enoxaparin sodium
Arovi 20mg/0.2ml solution for injection pre-filled syringes (ROVI Biotech Ltd)	12584941000033116	35569511000001101	Enoxaparin sodium
Enoxaparin Becat 20mg/0.2ml solution for injection pre-filled syringes (ROVI Biotech Ltd)	12476141000033111	35083211000001102	Enoxaparin sodium
Inhixa 20mg/0.2ml solution for injection pre-filled syringes (Techdow Pharma England Ltd)	12372741000033116	34699911000001109	Enoxaparin sodium
Tinzaparin sodium 12,000units/0.6ml solution for injection pre-filled syringes	10046341000033113	28566111000001107	Tinzaparin sodium
Innohep 12,000units/0.6ml solution for injection pre-filled syringes (LEO Pharma)	10046641000033117	28499911000001109	Tinzaparin sodium
Innohep 16,000units/0.8ml solution for injection pre-filled syringes (LEO Pharma)	10046741000033114	28498711000001103	Tinzaparin sodium
Tinzaparin sodium 16,000units/0.8ml solution for injection pre-filled syringes	10046441000033119	28566311000001109	Tinzaparin sodium
Tinzaparin sodium 8,000units/0.4ml solution for injection pre-filled syringes	10046241000033115	28566511000001103	Tinzaparin sodium
Innohep 8,000units/0.4ml solution for injection pre-filled syringes (LEO Pharma)	10046541000033118	28501011000001100	Tinzaparin sodium
Innohep 10,000units/0.5ml solution for injection pre-filled syringes (LEO Pharma)	763041000033118	3918011000001100	Tinzaparin sodium
Tinzaparin sodium 10,000units/0.5ml solution for injection pre-filled syringes	1440541000033116	35918811000001101	Tinzaparin sodium
Innohep 14,000units/0.7ml solution for injection pre-filled syringes (LEO Pharma)	763141000033119	3919011000001105	Tinzaparin sodium
Tinzaparin sodium 14,000units/0.7ml solution for injection pre-filled syringes	1440641000033115	35918911000001106	Tinzaparin sodium
Innohep 18,000units/0.9ml solution for injection pre-filled syringes (LEO Pharma)	762941000033111	3920011000001106	Tinzaparin sodium
Tinzaparin sodium 18,000units/0.9ml solution for injection pre-filled syringes	1440741000033112	35919011000001102	Tinzaparin sodium
Innohep 2,500units/0.25ml solution for injection pre-filled syringes (LEO Pharma)	2149141000033117	4120211000001102	Tinzaparin sodium
Tinzaparin sodium 2,500units/0.25ml solution for injection pre-filled syringes	2149041000033116	35919111000001101	Tinzaparin sodium
Innohep 20,000units/2ml solution for injection vials (LEO Pharma)	1781941000033119	4121311000001100	Tinzaparin sodium
Tinzaparin sodium 20,000units/2ml solution for injection vials	1781841000033110	35919211000001107	Tinzaparin sodium
Innohep 3,500units/0.35ml solution for injection pre-filled syringes (LEO Pharma)	1620241000033118	3916311000001101	Tinzaparin sodium
Tinzaparin sodium 3,500units/0.35ml solution for injection pre-filled syringes	1700641000033119	35919311000001104	Tinzaparin sodium
Innohep 4,500units/0.45ml solution for injection pre-filled syringes (LEO Pharma)	1620341000033111	4120611000001100	Tinzaparin sodium
Tinzaparin sodium 4,500units/0.45ml solution for injection pre-filled syringes	1700741000033111	35919411000001106	Tinzaparin sodium
Innohep 40,000units/2ml solution for injection vials (LEO Pharma)	762041000033110	4121711000001101	Tinzaparin sodium
Tinzaparin sodium 40,000units/2ml solution for injection vials	1439841000033110	35919511000001105	Tinzaparin sodium
Dalteparin sodium 7,500units/0.3ml solution for injection pre-filled syringes	2640441000033116	4865111000001103	Dalteparin sodium
Fragmin 7,500units/0.3ml solution for injection pre-filled syringes (Pfizer Ltd)	2640341000033110	4864211000001108	Dalteparin sodium

Fragmin 10,000units/1ml solution for injection ampoules (Pfizer Ltd)	610941000033116	3968911000001100	Dalteparin sodium
Dalteparin sodium 10,000units/1ml solution for injection ampoules	404041000033110	36092911000001101	Dalteparin sodium
Fragmin 10,000units/1ml solution for injection pre-filled syringes (Pfizer Ltd)	1781641000033114	3970311000001105	Dalteparin sodium
Dalteparin sodium 10,000units/1ml solution for injection pre-filled syringes	1781541000033113	36093011000001109	Dalteparin sodium
Fragmin 10,000units/4ml solution for injection ampoules (Pfizer Ltd)	611141000033113	3969411000001100	Dalteparin sodium
Dalteparin sodium 10,000units/4ml solution for injection ampoules	404241000033119	36093111000001105	Dalteparin sodium
Fragmin 100,000units/4ml solution for injection vials (Pfizer Ltd)	1822041000033115	3970611000001100	Dalteparin sodium
Dalteparin sodium 100,000units/4ml solution for injection vials	1821941000033114	36093311000001107	Dalteparin sodium
Fragmin 12,500units/0.5ml solution for injection pre-filled syringes (Pfizer Ltd)	1731141000033112	3838711000001100	Dalteparin sodium
Dalteparin sodium 12,500units/0.5ml solution for injection pre-filled syringes	1726841000033111	36563811000001103	Dalteparin sodium
Fragmin 15,000units/0.6ml solution for injection pre-filled syringes (Pfizer Ltd)	1731241000033117	3968311000001101	Dalteparin sodium
Dalteparin sodium 15,000units/0.6ml solution for injection pre-filled syringes	1726941000033115	36564211000001101	Dalteparin sodium
Fragmin 5,000units/0.2ml solution for injection pre-filled syringes (Pfizer Ltd)	611241000033118	3723611000001103	Dalteparin sodium
Dalteparin sodium 5,000units/0.2ml solution for injection pre-filled syringes	404341000033112	36566011000001101	Dalteparin sodium
Fragmin 10,000units/0.4ml solution for injection pre-filled syringes (Pfizer Ltd)	1731041000033113	3967611000001107	Dalteparin sodium
Dalteparin sodium 10,000units/0.4ml solution for injection pre-filled syringes	1726741000033118	37084311000001106	Dalteparin sodium
Fragmin 18,000units/0.72ml solution for injection pre-filled syringes (Pfizer Ltd)	1731341000033110	3968611000001106	Dalteparin sodium
Dalteparin sodium 18,000units/0.72ml solution for injection pre-filled syringes	1727041000033119	37084411000001104	Dalteparin sodium
Fragmin 2,500units/0.2ml solution for injection pre-filled syringes (Pfizer Ltd)	611041000033114	3840411000001109	Dalteparin sodium
Dalteparin sodium 2,500units/0.2ml solution for injection pre-filled syringes	404141000033114	37084511000001100	Dalteparin sodium

Table S4. Stroke Aurum codes

Term	MedCode ID	SNOMED CT Concept ID	SNOMED CT Description ID
Cerebral haemorrhage	884421000006119	274100004	884421000006119
External capsule haemorrhage	300276019	195167002	300276019
Basilar artery occlusion	300303013	195180004	300303013
Cerebral infarction due to thrombosis of cerebral	300321011	195189003	300321011
Anterior cerebral artery syndrome	300363010	195210002	300363010
Occlusion and stenosis of anterior cerebral artery	300396012	195233001	300396012
Occlusion and stenosis of posterior cerebral arter	300398013	195234007	300398013
Occlusion and stenosis of cerebellar arteries	300399017	195235008	300399017
Right sided cerebral infarction	451134017	307767006	451134017
H/O: Stroke in last year	451371010	308067002	451371010
Basal ganglion stroke	6837051000006119	413102000	2966612019
Subarachnoid haemorrhage from middle cerebral arte	123511000006114	21454007	36011016
Subarachnoid haemorrhage from posterior communicat	123521000006118	21454007	36011016
Left sided intracerebral haemorrhage, unspecified	748941000006115	274100004	409859018
Cerebrl infarctn due/unspcf occlusn or sten/cerebr	543141000006110	20059004	33759015
Internal capsule haemorrhage	496232015	52201006	496232015
Stroke due to cerebral arterial occlusion	122361000006113	230691006	345638019
Subarachnoid haemorrhage from basilar artery	123491000006115	276284000	412361011
[V]Personal history of transient ischaemic attack	1667741000000110	751371000000107	1667741000000110
Cerebral haemorrhage	989201000006117	274100004	989201000006117
Lobar cerebral haemorrhage	345675012	230710000	345675012
Cerebral A. occlusion NOS	884501000006113	682621000000105	884501000006113
Subdural haemorrhage	884441000006114	195176009	884441000006114
Lateral medullary syndrome	130375018	78569004	130375018
[RFC] Stroke	907591000006116	907591000006100	907591000006116
[RFC] Stroke	908801000006114	908801000006105	908801000006114
Right sided intracerebral haemorrhage, unspecified	163261000006119	195168007	300277011
Subarachnoid haemorrh from intracranial artery, un	123441000006112	21454007	481028017
[X]Other intracerebral haemorrhage	11919571000006110	274100004	11919571000006110
Infarction of basal ganglia	2474651019	413102000	2474651019
[V]Personal history of stroke	1227591017	275526006	2476091017
Basilar artery occluded	884461000006113	195180004	884461000006113
H/O: CVA/stroke	809421000006116	275526006	2986886017
Intracerebral haemorrhage NOS	300287010	274100004	2819959010
Cerebral thrombosis	118689010	71444005	118689010
H/O: CVA	411518010	275526006	411518010
Left sided cerebral infarction	451133011	307766002	451133011
Brain stem stroke	4057041000006110	95457000	2966556014
Lacunar stroke	5011161000006115	230698000	345652017

Cereb infarct due unsp occlus/stenos precerebr art	542261000006114	125081000119106	3042974014
[X]Other transnt cerebral ischaemic attacks+relate	416991000006112	266257000	395788015
Stroke due to intracerebral haemorrhage	11903571000006110	274100004	11903571000006110
Subdural haematoma - nontraumatic	2475119012	195176009	2469010019
Stroke due to intracerebral haemorrhage	122371000006118	274100004	409860011
[X]Other subarachnoid haemorrhage	300936018	21454007	481028017
Carotid territory transient ischaemic attack	345684012	230716006	345684012
Cerebral amyloid angiopathy	345701012	230724001	345701012
Subarachnoid haemorrhage from basilar artery aneur	5583101000006116	276284000	412361011
Posterior cerebral artery syndrome	300364016	195211003	300364016
Sequelae of intracerebral haemorrhage	300407010	195241001	300407010
Brainstem infarction	524541000006117	95457000	158113017
Cereb infarct due cerebral venous thrombosis, nonp	542251000006112	195230003	300393016
Cerebral haemorrhage NOS	989211000006119	274100004	989211000006119
Stroke and cerebrovascular accident unspecified	12727691000006117	685631000000102	1501361000000113
Intracerebral haemorrhage	744901000006114	274100004	2819959010
Cortical haemorrhage	495394013	49422009	495394013
Cerebellar haemorrhage	502878012	75038005	502878012
Bulbar haemorrhage	483988011	732923001	3467313018
CVA - cerebral artery occlusion	605461000006117	230691006	345638019
CVA - Cerebrovascular accident unspecified	605491000006113	230690007	345635016
Extradural haemorrhage - nontraumatic	660111000006117	397809001	1773153015
Subarachnoid haemorrhage from anterior communicati	123481000006118	21454007	36011016
Stroke	884531000006117	685631000000102	884531000006117
Ruptured berry aneurysm	300242011	195154000	300242011
Subarachnoid haemorrhage from carotid siphon and b	300244012	195155004	300244012
Subarachnoid haemorrhage from vertebral artery	300253017	195160000	300253017
Subdural haemorrhage - nontraumatic	300294013	195176009	300294013
Cerebral infarction due to embolism of precerebral	300313017	195186005	300313017
Sequelae of subarachnoid haemorrhage	300406018	195240000	300406018
Sequelae of cerebral infarction	300411016	195243003	300411016
[X]Cereb infarct due unsp occlus/stenos precerebr	370661000006114	125081000119106	3042974014
Cerebral infarction due to embolism of cerebral ar	300322016	195190007	300322016
Cerebral embolus	542831000006116	75543006	125470015
[X]Other cerebral infarction	300941014	432504007	2770034014
Intracerebral haemorrhage in hemisphere, unspecifi	744921000006116	274100004	409860011
Intracerebral haemorrhage, multiple localized	746571000006116	195169004	300280012
[X]Occlusion and stenosis of other cerebral arteri	300943012	20059004	33759015
[X]Other intracerebral haemorrhage	300939013	274100004	409860011
Cerebral haemorrhage NOS	884451000006111	700251000000105	884451000006111
CVA unspecified	605501000006117	230690007	345637012

[X]Subarachnoid haemorrh from intracranial artery,	428181000006115	21454007	481028017
Sequelae of other nontraumatic intracranial haemor	149551000006111	363302008	482447013
Sequelae of stroke,not specfd as h'morrhage or inf	149571000006118	195239002	300403014
Infarction - cerebral	218511000000117	432504007	2770034014
Cerebral infarction NOS	12727431000006119	682621000000105	1495311000000117
Basal nucleus haemorrhage	503791000006114	195165005	300272017
Stroke/CVA - undefined	884521000006115	685631000000102	884521000006115
[X]Cerebrl infarctn due/unspcf occlusn or sten/cer	370701000006118	20059004	33759015
History of stroke in last year	5974521000006116	308067002	2986393012
[RFC] Stroke/CVA	907581000006119	907581000006103	907581000006119
Brainstem infarction NOS	345650013	95457000	158113017
Intracerebral haemorrhage, intraventricular	300277011	195168007	300277011
Cerebral infarct due to thrombosis of precerebral	300312010	195185009	300312010
Middle cerebral artery syndrome	300362017	195209007	300362017
Right sided CVA	300371014	195217004	300371014
Intracranial haemorrhage NOS	300298011	1386000	475553012
Subarachnoid haemorrhage NOS	300257016	21454007	481028017
[V]Personal history of cerebrovascular accident (C	1227592012	266995000	397829016
Right sided intracerebral haemorrhage, unspecified	12722481000006116	308128006	451441015
Subarachnoid haemorrhage	481028017	21454007	481028017
[X]Intracerebral haemorrhage in hemisphere, unspec	300956017	274100004	409860011
Pontine haemorrhage	503469016	7713009	503469016
H/O: stroke	2476091017	275526006	2476091017
Cerebellar infarction	158118014	95460007	158118014
Intracranial subarachnoid haemorrhage from vertebr	4777761000006112	195160000	2916525016
Stroke and cerebrovascular accident unspecified	405339016	230690007	345637012
Wallenberg syndrome	57341000006119	78569004	130374019
Other and unspecified intracranial haemorrhage	300290016	62914000	104563015
CVA - cerebrovascular accid due to intracerebral h	605471000006112	274100004	409859018
Stroke	5010981000006119	230690007	345636015
CT - Cerebral thrombosis	3662331000006119	71444005	1233388010
Occlusive stroke	1212072018	373606000	1212072018
Cerebral arterial occlusion	1222398015	20059004	1222398015
H/O: TIA	251692018	161511000	251692018
Cerebral infarction NOS	395780010	432504007	2770034014
Stroke unspecified	122401000006115	230690007	345637012
Cerebral embolism	125470015	75543006	125470015
Left sided CVA	300370010	195216008	300370010
Occlusion and stenosis of middle cerebral artery	300395011	195232006	300395011
[X]Subarachnoid haemorrhage from other intracrania	300935019	21454007	36011016

Table S5. Transient ischaemic attack selection Aurum codes

Term	Medcode ID	SNOMED CT Concept ID	SNOMED CT Description ID
Anterior circulation transient ischaemic attack	5011491000006115	230716006	345683018
Transient cerebral ischemia	11920121000006117	266257000	395785017
Personal history of transient ischaemic attack	8231151000006110	751371000000107	1653121000000119
[V]Personal history of transient ischaemic attack	1667741000000110	751371000000107	1667741000000110
Transient ischaemic attack	395783012	266257000	395783012
Transient cerebral ischaemia	395788015	266257000	395788015
Carotid territory transient ischaemic attack	345684012	230716006	345684012
Transient Ischaemic Attacks	884511000006111	584181000000100	884511000006111
H/O: TIA	251692018	161511000	251692018
Transient Ischaemic Attacks	988951000006117	266257000	988951000006117
TIA - Transient ischaemic attack	5492201000006111	266257000	395784018

Table S6. Arterial thromboembolism Aurum codes

Term	MedCode ID	SNOMED CT Concept ID	SNOMED CT Description ID
Embolism and/or thrombosis of the internal iliac artery	300554012	734298005	3503835018
Cerebral infarction due to thrombosis of cerebral arteries	300321011	195189003	300321011
Embolism and thrombosis of the brachial artery	300535012	195319003	300535012
Embolism and thrombosis of the posterior tibial artery	300543019	195327007	300543019
Recurrent pulmonary embolism	632111000000118	438773007	2793990014
Embolism and thrombosis of the celiac artery	4778981000006116	195342005	300560012
Superior mesenteric artery embolus	4785631000006110	196999001	302978019
Cerebral arterial thrombosis	3662321000006117	71444005	118691019
Saddle embolus of abdominal aorta	5058281000006118	233972005	350565011
Peripheral arterial embolism	884661000006110	583731000000103	884661000006110
Embolus/thrombus artery NOS	884671000006115	583761000000108	884671000006115
Arterial embolism and thrombosis NOS	12733161000006111	583761000000108	1295451000000114
Thrombosis - coronary	1786198013	398274000	1786198013
Thrombosis of cerebral arteries	3662311000006113	71444005	118690018
Cerebral arterial embolism	3728451000006116	75543006	125471016
Renal artery thrombosis	158317012	95579008	158317012
Embolism and thrombosis of other specified artery	300552011	266262004	395794011
Arterial embolism and thrombosis NOS	300564015	266262004	395794011
Aortic bifurcation embolus	5058291000006115	233972005	350566012
Embolism and thrombosis of a leg artery NOS	300547018	195318006	300534011
Peripheral arterial embolism and thrombosis NOS	300550015	195318006	300534011
Renal artery embolism	158318019	95580006	158318019
Thromboembolus of internal iliac artery	3503835018	734298005	3503835018
SMAE - Superior mesenteric artery embolus	4785641000006117	196999001	302979010
Renal artery stent thrombosis	1848121000006110	840961000000108	2182101000000113
Thrombosis, carotid artery	100771000006112	86003009	142588012
Embolism and thrombosis of an arm artery NOS	300538014	195318006	300534011
Saddle embolus	350563016	233972005	350563016
Embolism and/or thrombosis of the external iliac artery	300555013	734299002	3503839012
Embolism and thrombosis of an arm or leg artery	300534011	195318006	300534011
Embolism and/or thrombosis of the common iliac artery	300553018	195335005	300553018
Embolism and thrombosis of the splenic artery	300558010	195340002	300558010
Embolus/thrombosis abd. aorta	884641000006111	266263009	884641000006111
Embolus/thrombosis aorta NOS	884651000006113	195317001	884651000006113
Embolism and thrombosis of other and unspec parts aorta	638871000006114	274101000	409861010
Embolus of the superior mesenteric artery	639241000006110	196999001	302978019
Arterial embolus and thrombosis	395795012	266262004	395795012

Embolism and thrombosis of the iliac artery unspecified	300556014	266262004	395794011
[X]Embolism and thrombosis of other arteries	300964011	266262004	395794011
Cerebral infarction due to embolism of precerebral arteries	300313017	195186005	300313017
Embolism and thrombosis of the anterior tibial artery	300541017	195325004	300541017
Embolism and thrombosis of the axillary artery	300559019	195341003	300559019
Incomplete spontaneous abortion with embolism	305331019	198650006	305331019
Embolism and thrombosis of other arteries NOS	300563014	266262004	395794011
Cerebral infarction due to embolism of cerebral arteries	300322016	195190007	300322016
Embolism and thrombosis of the radial artery	300536013	195320009	300536013
Embolism and thrombosis of the coeliac artery	300561011	195342005	300561011
Arterial embolism/thrombosis	884631000006118	266262004	884631000006118
Peripheral arterial embolism and thrombosis NOS	12733151000006114	583731000000103	1295391000000110
Thrombosis of renal artery	4059051000006119	95579008	158316015
Aortic thromboembolism	5560171000006116	274101000	409861010
Embolism and thrombosis of the abdominal aorta	395796013	266263009	395796013
Other embolism and thrombosis	300712012	429098002	2692478010
Thrombosis of artery of transplanted kidney	2234541000000115	864271000000105	2234541000000115
Chronic thromboembolic pulmonary hypertension	5057801000006113	233947005	2968345015
Thromboembolic pulmonary hypertension	350517010	233947005	350517010
[V] Personal history of pulmonary embolism	451479013	161512007	251693011
Cerebral infarct due to thrombosis of precerebral arteries	300312010	195185009	300312010
Embolism and thrombosis of the thoracic aorta	300533017	195317001	300533017
Embolism and thrombosis of the ulnar artery	300537016	195321008	300537016
Embolism and thrombosis of the popliteal artery	300540016	195324000	300540016
Pulmonary embolism	98484016	59282003	98484016
Embolism and thrombosis of the hepatic artery	300562016	195343000	3491726018
Embolism and thrombosis NOS	300713019	429098002	2692478010
Anterior spinal artery thrombosis	345723011	230740003	345723011
Embolism and thrombosis of hepatic artery	4778991000006118	195343000	3491726018
Arteriovenous fistula thrombosis	350892013	234205007	350892013
Renal artery stent thrombosis	1809151000006116	1809151000006100	1809151000006116
Thrombus of the superior mesenteric artery	100811000006112	197000003	302980013
Thromboembolus of external iliac artery	3503839012	734299002	3503839012
Embolism and thrombosis NOS	638821000006113	429098002	2692478010
Thrombosis - arterial	218541000000116	65198009	108347015
Arterial embolism and thrombosis	491241000006118	266262004	395795012
PE - Pulmonary embolism	3462921000006119	59282003	1231937012
Renal artery embolus	1235925019	95580006	1235925019
[RFC] Arterial embolism of limbs	905541000006119	905541000006103	905541000006119
Arterial embolic and thrombotic occlusion	395794011	266262004	395794011

Embolism and thrombosis of the femoral artery	300539018	195323006	300539018
Embolism and thrombosis of the dorsalis pedis artery	300542012	195326003	300542012
Embolism and thrombosis of the subclavian artery	300557017	195339004	300557017
Thromboembolic pulmonary hypertension	1749291000006113	1749291000006109	1749291000006113

Table S7. Myocadial infarction Aurum codes

Term	Medcode ID	SNOMED CT Concept ID	SNOMED CT Description ID
Acute anteroapical infarction	299708014	52035003	3038718019
Subsequent myocardial infarction of inferior wall	299812011	194858006	299812011
Acute Q-wave infarct	447324018	304914007	447324018
Acute transmural myocardial infarction of unspecif site	460681000006116	57054005	94884017
Postmyocardial infarction syndrome	3576371000006117	66189004	109915012
Acute non-Q wave infarction	450322013	307140009	450322013
Myocardial infarct	2855351000006111	22298006	1784873012
Acute myocardial infarction of inferolateral wall	3565871000006113	65547006	108912018
Acute inferior myocardial infarction	3699921000006110	73795002	1233665015
Subsequent myocardial infarction of unspecified site	118831000006118	194856005	299808017
Postoperative transmural myocardial infarction other sites	212081000006112	129574000	208365015
Postoperative transmural myocardial infarction unspec site	212091000006110	129574000	208365015
Acute myocardial infarction of atrium	4775891000006119	194809007	2470032018
Acute myocardial infarction of septum	3784911000006111	79009004	131106018
Other acute myocardial infarction NOS	299720018	57054005	94884017
History of myocardial infarction in last year	5974481000006116	308065005	2986726013
Acute papillary muscle infarction	1218860015	10273003	1218860015
Acute myocardial infarction of anterolateral wall	3641641000006116	70211005	116613011
Personal history of myocardial infarction	230021000006115	399211009	2986492018
Acute Q wave myocardial infarction	5935321000006111	304914007	447323012
Silent myocardial infarction	350376014	233843008	350376014
Acute ST segment elevation myocardial infarction	1780491019	401303003	1780491019
MI - Myocardial infarction	2855341000006114	22298006	1784872019
History of myocardial infarction	1738171000006114	399211009	2986492018
Pericarditis following myocardial infarction	5056951000006117	233885007	350432018
Postoperative myocardial infarction	208365015	129574000	208365015
MI - Silent myocardial infarction	5056461000006113	233843008	350377017
Acute myocardial infarction of septum alone	3784921000006115	79009004	131107010
Acute posterolateral myocardial infarction	967931000006114	15990001	27071012
Previous myocardial infarction	6619201000006117	399211009	545891000000115
Acute myocardial infarction of lateral wall	3452181000006112	58612006	97399018
Myocardial infarction with complication	6348651000006112	371068009	6348651000006112
Lateral myocardial infarction NOS	299711010	58612006	1231860015
Hemopericardium due to and following acute myocardial infarction	12220751000006113	194862000	3673281015
Ventric septal defect/curr comp fol acut myocardal infarctn	67081000006119	233846000	350381017
Postmyocardial infarction syndrome	109915012	66189004	500341014
AMI - Acute myocardial infarction	3427201000006111	57054005	1231678011

Postoperative transmural myocardial infarction inferior wall	212071000006114	311793000	455419012
Other specified anterior myocardial infarction	299707016	54329005	1231324017
Posterior myocardial infarction NOS	299710011	233838001	350371016
Other acute myocardial infarction	299718016	57054005	94884017
Acute myocardial infarction of anterior wall	3381601000006117	54329005	90302019
Anterior myocardial infarction NOS	299709018	54329005	1231324017
Past history of myocardial infarction	6619191000006115	399211009	1786753012
True posterior myocardial infarction	299712015	194802003	299712015
Subsequent myocardial infarction of anterior wall	299811016	194857001	299811016
Acute inferolateral infarction	457531000006110	65547006	1232697013
Acute myocardial infarction of inferoposterior wall	3745741000006117	76593002	127193015
[RFC] Myocardial infarction (MI)	905351000006113	905351000006109	905351000006113
Cardiac rupture following myocardial infarction (MI)	537751000006115	233847009	350383019
Old myocardial infarction	4031011	1755008	4031011
Subsequent myocardial infarction	299808017	194856005	299808017
Acute anteroseptal infarction	455651000006114	62695002	104192010
[X]Acute transmural myocardial infarction of unspecif site	362461000006119	57054005	94884017
First myocardial infarction	932081000006118	932081000006102	932081000006118
STEMI - ST elevation myocardial infarction	6651221000006117	401303003	2840840013
Postoperative subendocardial myocardial infarction	455422014	311796008	455422014
Acute anterolateral infarction	455641000006112	70211005	1233238016
[X]Subsequent myocardial infarction of unspecified site	300882013	194856005	299808017
Postoperative myocardial infarction, unspecified	455423016	129574000	208365015
Acute myocardial infarction of diaphragmatic wall	3699911000006119	73795002	122559017
Acute subendocardial infarction	116992017	70422006	116992017
Inferior myocardial infarction NOS	299714019	73795002	122557015
Acute myocardial infarction NOS	299721019	57054005	94884017
Subsequent myocardial infarction of other sites	299813018	194856005	299808017
Cardiac rupture due to and following acute myocardial infarction	12221301000006119	233847009	3673284011
Hemopericardium as current complication following acute myocardial infarction	4776221000006110	194862000	299817017
Acute septal infarction	1234306015	79009004	1234306015
Acute non-ST segment elevation myocardial infarction	1780501013	401314000	1780501013
History of myocardial infarct at age greater than sixty	4540481000006111	161503005	2986493011
Postoperative transmural myocardial infarction anterior wall	212061000006119	311792005	455418016
Acute anteroapical myocardial infarction	3343471000006116	52035003	86618010
[X]Subsequent myocardial infarction of other sites	300881018	194856005	299808017
Postmyocardial infarction pericarditis	3576391000006116	66189004	109917016
History of myocardial infarct at age less than sixty	4540461000006118	161502000	2986996013
Acute inferoposterior infarction	1234005010	76593002	1234005010

Myocardial Infarction	884151000006119	57054005	884151000006119
MI - acute myocardial infarction	219531000000117	57054005	94884017
Acute myocardial infarction	94884017	57054005	94884017
Myocardial infarction	2855301000006112	22298006	37436014

Table S8. Periperal arterial disease Aurum codes

Term	Medcode ID	SNOMED CT Concept ID	SNOMED CT Description ID
Ischaemic foot	443199013	301755001	443199013
Peripheral angiopathic disease EC NOS	300511019	400047006	1779317016
Other specified peripheral vascular disease NOS	300514010	400047006	1779317016
Peripheral vascular disease NOS	300515011	400047006	1779317016
Peripheral arterial disease confirmed	1823971000006118	1823971000006102	1823971000006118
Peripheral ischaemic vascular disease	350535018	233958001	350535018
Ischaemic foot	1699111000006110	1699111000006106	1699111000006110
Other specified peripheral vascular disease	300510018	400047006	1779317016
Peripheral vascular disease NOS	235911000006116	400047006	1779317016
Ischaemia of legs	742481000006118	233961000	350546015
Other peripheral vascular disease	395791015	400047006	1779317016
[X]Other specified peripheral vascular diseases	300963017	400047006	1779317016
Peripheral ischaemia	350533013	233958001	350533013
Peripheral vascular disease NOS	12729241000006119	646351000000103	1422221000000115
Peripheral arterial disease	1847121000006114	399957001	1787050010
Aortic atherosclerosis	218531000000113	81817003	135728018

Table S9. Aortic plaque Aurum codes

Term	MedCode ID	SNOMED CT Concept ID	SNOMED CT Description ID
Atherosclerosis aorta	3830431000006112	81817003	2923328010
Aortic thromboembolism	5560171000006116	274101000	409861010
Aortoiliac atherosclerosis	5057931000006110	233956002	350530011
Aortic atherosclerosis	218531000000113	81817003	135728018

Table S10. Diabetes Aurum codes

Term	Medcode ID	SNOMED CT Concept ID	SNOMED CT Description ID
Pre-existing type 2 diabetes mellitus in pregnancy	11931861000006112	237627000	1988741000006117
Unstable type I diabetes mellitus	429970018	290002008	429970018
Diabetes mellitus with ketoacidosis	2622193012	420422005	2622193012
Lipoatrophic diabetes mellitus	5687751000006119	284449005	1495355018
Other specified diabetes mellitus with multiple comps	13751000006117	385041000000108	760111000000115
Other specified diabetes mellitus with periph circ comps	13791000006111	421895002	2618203015
Unstable type 1 diabetes mellitus	72711000006117	290002008	429971019
Type 1 diabetes mellitus - poor control	84291000006117	444073006	2872487013
Type 1 diabetes mellitus with nephropathy	84371000006110	421893009	2623054013
Type 1 diabetes mellitus with polyneuropathy	84421000006115	713705003	3297342019
Type 1 diabetes mellitus with ulcer	84451000006112	190368000	292540012
Type 2 diabetes mellitus - poor control	84481000006116	443694000	2921019012
Type 2 diabetes mellitus with arthropathy	84491000006118	314903002	459310018
Type 2 diabetes mellitus with neuropathic arthropathy	84571000006115	314904008	459313016
Type 2 diabetes mellitus with peripheral angiopathy	84591000006119	314902007	459308015
Type 2 diabetes mellitus with polyneuropathy	84601000006110	713706002	3297353013
Type I diabetes mellitus with arthropathy	84681000006118	314893005	459294012
Type I diabetes mellitus with hypoglycaemic coma	84711000006117	314771006	459163017
Type I diabetes mellitus with ulcer	84821000006118	190368000	292538019
Type II diabetes mellitus with polyneuropathy	84971000006111	713706002	3297353013
Type II diabetes mellitus with renal complications	84981000006114	420279001	3013392012
Insulin dependent diabetes mellitus with arthropathy	6051171000006117	314893005	459293018
Insulin-dependent diabetes mellitus secretory diarrhea syndrome	5109851000006119	237618001	356116016
Type I diabetes mellitus without complication	457325013	313435000	457325013
Non-insulin-dependent diabetes mellitus without complication	457328010	313436004	457328010
MODY - Maturity onset diabetes glucokinase-related	5109621000006112	237604008	356087019
DKA - diabetic ketoacidosis	6953901000006115	420422005	3303994013
Maturity onset diabetes of the young type 5	1968641000006114	609572000	2967853015
Diabetes mellitus, juvenile type, + unspecified complication	616611000006111	420868002	3013528019
Pre-existing diabetes mellitus in mother complicating childbirth	8033291000006116	106281000119103	3005790010
ADM - atypical diabetes mellitus	11633221000006116	530558861000132104	3334355014
Type I diabetes mellitus with hypoglycemic coma	6050061000006116	314771006	459164011
Type 1 diabetes mellitus with multiple complications	84361000006115	422228004	2618232017
Type I diabetes mellitus with neurological complications	84751000006116	421468001	3695401013
Non-insulin dependent diabetes mellitus with mononeuropathy	280521000006117	420436000	3697667019

Non-insulin-dependent diabetes mellitus with ophthalm comps	587111000006111	422099009	3698440016
Diabetes mellitus, adult onset, with renal manifestation	616551000006111	420279001	2615535015
Diabetes mellitus, adult with gangrene	616561000006113	421631007	2618206011
Diabetes mellitus, juvenile +peripheral circulatory disorder	616591000006117	421365002	2618205010
Type 1 diabetes mellitus with multiple complications	913541000006118	422228004	2618232017
Type I diabetes mellitus with retinopathy	913671000006113	420789003	3699407019
Type I diabetes mellitus with mononeuropathy	913791000006119	420918009	3697664014
Type II diabetes mellitus with neurological complications	914081000006117	421326000	3695406015
Type II diabetes mellitus with retinopathy	914161000006114	422034002	3699410014
Type II diabetes mellitus with mononeuropathy	914231000006111	420436000	3697667019
Type II diabetes mellitus with diabetic cataract	914311000006114	420756003	3688522015
Type II diabetes mellitus with gastroparesis	1667921000000117	713703005	3698412014
Type I diabetes mellitus with gastroparesis	1667941000000112	713702000	3698406016
Insulin-dependent diabetes mellitus with neurological comps	771481000006112	422088007	3688477014
Insulin-dependent diabetes mellitus with ophthalmic comps	771491000006110	25093002	3688467012
Other specified diabetes mellitus with ketoacidosis	292478012	420422005	2616611010
Diabetes mellitus NOS with ketoacidotic coma	292489017	420422005	2616611010
Other specified diabetes mellitus with renal complications	292495016	127013003	301016
Type I diabetes mellitus - poor control	292550013	444073006	2872487013
Type II diabetes mellitus - poor control	292589019	443694000	2921019012
[X]Other specified diabetes mellitus	293756010	73211009	121589010
Insulin-dependent diabetes mellitus with ulcer	4758061000006110	190368000	292539010
Diabetes type 2 with cataract	6959811000006110	420756003	3035281015
Diabetes type 2 with retinopathy	6982521000006119	422034002	3035295017
Diabetes mellitus, Addison's disease and myxoedema	3862341000006115	83728000	505747013
Type 1 diabetes mellitus with arthropathy	459292011	314893005	459292011
Type I diabetes mellitus with arthropathy	459294012	314893005	459294012
Type II diabetes mellitus with peripheral angiopathy	459306016	314902007	459306016
Type 2 diabetes mellitus with peripheral angiopathy	459308015	314902007	459308015
Non-insulin dependent diabetes mellitus with arthropathy	459309011	314903002	459309011
NIDDM - Non-insulin dependent diabetes mellitus	493773010	44054006	493773010
Secondary diabetes mellitus without complication	189721000000113	8801005	15518018
Pre-existing diabetes mellitus, unspecified	213141000006111	385051000000106	760131000000111
Type 1 diabetes mellitus with hyperosmolar coma	4757991000006116	190330002	2967854014
Type 1 diabetes mellitus with hypoglycemic coma	6050081000006114	314771006	459166013
Diabetes type 1 with ketoacidosis	6951091000006110	420270002	3035606019
NIDDM with peripheral circulatory disorder	12485441000006119	422166005	674961000006118
DM - Diabetes mellitus	3690681000006114	73211009	502372015

Diabetes mellitus type 2 with ketoacidotic coma	6979251000006110	421847006	3035601012
Diet controlled diabetes mellitus	4636411000006110	170745003	264680017
Type I diabetes mellitus with ophthalmic complications	913491000006111	739681000	3698438014
Diabetes mellitus NOS with neurological manifestation	292512019	422088007	2618195014
Other specified diabetes mellitus with neurological comps	13761000006115	422088007	2618195014
Type 1 diabetes mellitus with diabetic cataract	84321000006114	421920002	2618234016
Type 2 diabetes mellitus with gangrene	84511000006112	421631007	2618206011
Type 2 diabetes mellitus with mononeuropathy	84531000006118	420436000	2618201018
Diabetes mellitus, adult onset, + ophthalmic manifestation	616491000006116	422099009	2618233010
Type 1 diabetes mellitus with mononeuropathy	913781000006117	420918009	2618199015
Type 1 diabetes mellitus with ophthalmic complications	84401000006113	739681000	3537386015
Diabetes mellitus, juvenile type, + ophthalmic manifestation	616601000006113	739681000	3537386015
Diabetes mellitus with polyneuropathy	616451000006110	49455004	82373015
Diabetes mellitus, juvenile, + other specified manifestation	616681000006116	73211009	121589010
Diabetes mellitus confirmed	1823921000006119	1823921000006103	1823921000006119
Diabetic on insulin	264682013	170747006	264682013
Diabetic - cooperative patient	264717015	170769004	264717015
Insulin treated non-insulin dependent diabetes mellitus	1223148019	237599002	1223148019
Maturity onset diabetes	223291000000111	44054006	493774016
Newly diagnosed diabetes	1694761000006113	405749004	2157525015
Diabetic acidosis	6953891000006119	420422005	2622194018
MODY - Maturity onset diabetes in youth type 2	5109611000006116	237604008	356086011
Diabetes mellitus with neuropathy	345487013	230572002	345487013
Diabetes type 2 with neurological disorder	6969931000006116	421326000	3035455016
Type 2 diabetes mellitus with hyperosmolar coma	4758011000006112	190331003	2967818010
Diabetes with coma	932641000006116	630521000000101	932641000006116
DM + persist microalbuminuria	933211000006112	658061000000102	933211000006112
Diabetes + nephropathy	881481000006117	127013003	881481000006117
Diabetes + eye manifestation	881491000006119	25093002	881491000006119
Diabetes + periph.circulat.dis	881511000006113	422275004	881511000006113
Lipodystrophic diabetes with partial lipoatrophy	3730121000006115	75659004	125663012
Secondary endocrine diabetes mellitus	356078011	237601000	356078011
Diabetes mellitus associated with genetic syndrome	10928019	5969009	10928019
Type 1 diabetes mellitus with persistent microalbuminuria	1780311019	401110002	1780311019
Diabetes mellitus	121589010	73211009	121589010
Maturity onset diabetes in youth type II	5109581000006112	237604008	356083015
Insulin treated Type II diabetes mellitus	841351000006110	237599002	1223147012
Type I diabetes mellitus with renal complications	913461000006115	421893009	2623054013
Type I diabetes mellitus with nephropathy	913851000006119	421893009	2623054013

Type II diabetes mellitus with polyneuropathy	914251000006116	713706002	3297353013
Insulin treated Type 2 diabetes mellitus	914391000006116	237599002	2967820013
Type I diabetes mellitus with persistent microalbuminuria	928491000006110	401110002	1780311019
Type I diabetes mellitus with ketoacidosis	928511000006116	420270002	2967817017
Type I diabetes mellitus with ketoacidotic coma	928531000006110	421075007	2967758019
Type II diabetes mellitus with ketoacidosis	928591000006114	421750000	2967754017
Type I diabetes mellitus with exudative maculopathy	938311000006112	420486006	2618236019
Insulin dependent diabetes mellitus with hypoglycaemic coma	459162010	237632004	356141011
Pre-existing type 1 diabetes mellitus in pregnancy	2967831017	199223000	306106018
Type 1 diabetes mellitus with peripheral angiopathy	84411000006111	31211000119101	3315042015
Insulin dependent diabetes mellitus with multiple complicatn	771381000006115	385041000000108	760111000000115
Insulin dependent diabetes mellitus with polyneuropathy	771401000006115	49455004	82373015
Insulin-dependent diabetes mellitus with renal complications	771501000006119	127013003	301016
Insulin dependent diab mell with peripheral angiopathy	772141000006118	421895002	2618203015
IDDM with peripheral circulatory disorder	787101000006114	421895002	2618203015
Insulin-dependent diabetes mellitus with renal complications	913441000006119	127013003	301016
Insulin dependent diabetes mellitus with retinopathy	913651000006115	4855003	9093013
Insulin dependent diabetes mellitus with nephropathy	913831000006114	127013003	301016
Insulin dependent diab mell with peripheral angiopathy	913921000006119	421895002	2618203015
Type 1 diabetes mellitus with peripheral angiopathy	913931000006116	31211000119101	3315042015
Insulin dependent diab mell with neuropathic arthropathy	913981000006115	201724008	309740011
[RFC] Diabetes	905621000006113	905621000006109	905621000006113
[RFC] Diabetes mellitus	908831000006118	908831000006102	908831000006118
Diabetes mellitus co-occurrent and due to cystic fibrosis	7063141000006118	426705001	3440582013
[X]Diabetes mellitus	377001000006117	73211009	121589010
Type I diabetes mellitus maturity onset	292551012	190372001	292551012
Type 1 diabetes mellitus maturity onset	292553010	190372001	292553010
Diabetes type 1 with gangrene	6961101000006114	420825003	3035408014
Maturity onset diabetes in youth	483886014	609561005	2967884018
Diabetes mellitus, Addison's disease and myxedema	3862421000006116	83728000	505755018
Diabetes mellitus type 1	3253241000006117	46635009	197985011
Diabetes with coma	881471000006115	26298008	881471000006115
Diabetes mellitus autosomal dominant type 2	840971000006112	237604008	356082013
Type 1 diabetes mellitus with nephropathy	913841000006116	421893009	2623054013
Type 2 diabetes mellitus with renal complications	914031000006118	420279001	2618208012
Type 1 diabetes mellitus with ketoacidosis	928501000006119	420270002	2967817017

Type 2 diabetes mellitus with persistent proteinuria	928541000006117	421986006	2967751013
Type 2 diabetes mellitus	197761014	44054006	197761014
Latent diabetes	2649991000006110	9414007	16500010
Type 1 diabetes mellitus without complication	457326014	313435000	457326014
Type 2 diabetes mellitus with arthropathy	459310018	314903002	459310018
Diabetic on insulin and glucagon-like peptide 1	1966321000006112	976361000000100	2483761000000117
Diabetes mellitus NOS with hyperosmolar coma	292484010	422126006	2618221017
[X]Unspecified diabetes mellitus with renal complications	293759015	127013003	301016
DM caused by insulin receptor ab	3296773014	75682002	3296773014
DM due to insulin receptor ab	2164134013	75682002	2164134013
Non-insulin dependent diabetes mellitus	73466011	44054006	493773010
Diabetes with peripheral circulatory disorder	4392211000006114	127014009	3313383012
Ketoacidosis in type II diabetes mellitus	6977421000006110	421750000	2618220016
Diabetes mellitus, adult onset, + unspecified complication	616501000006112	422014003	3013072014
Diabetes mellitus, juvenile type, no mention of complication	616621000006115	313435000	457326014
Insulin dependent diabetes mellitus - poor control	772181000006112	268519009	401531012
Insulin dependent diabetes mellitus - poor control	913681000006111	268519009	401531012
Type I diabetes mellitus with peripheral angiopathy	913941000006114	31211000119101	3315042015
Type II diabetes mellitus with hypoglycaemic coma	459169018	719216001	3316336015
Brittle diabetes mellitus	2683561000006119	11530004	2658852011
Non-insulin dependent diabetes mellitus with gangrene	281211000006117	421631007	2967779019
Type II diabetes mellitus with exudative maculopathy	12489851000006117	421779007	938331000006118
Diabetes type 2 on insulin	5109461000006115	237599002	3037103012
Diabetes mellitus NOS with ketoacidosis	292479016	420422005	2616611010
Ketoacidosis in diabetes mellitus	6953871000006115	420422005	2622192019
Jamaica type diabetes	1550571000000114	75524006	1550571000000114
Non-insulin dependent d m with neuropathic arthropathy	281181000006116	314904008	459314010
Type II diabetes mellitus with renal complications	914041000006111	420279001	3013392012
Type II diabetes mellitus with persistent microalbuminuria	928571000006113	420715001	2967769016
Type I diabetes mellitus with mononeuropathy	84721000006113	420918009	3697664014
Type II diabetes mellitus with diabetic cataract	84871000006117	420756003	3688522015
Type II diabetes mellitus with mononeuropathy	84901000006117	420436000	3697667019
Type II diabetes mellitus with neurological complications	84931000006113	421326000	3695406015
Diabetes mellitis with nephropathy NOS	292496015	127013003	301016
Diabetes mellitus NOS with unspecified complication	292626017	74627003	123939015
Type 1 diabetes mellitus uncontrolled	7287611000006113	444073006	2967763015
Insulin-dependent diabetes without complication	6030001000006116	313435000	457327017
Type I diabetes mellitus with neurological complications	913521000006113	421468001	3695401013

Type 1 diabetes mellitus with retinopathy	913661000006118	420789003	3699407019
Type 2 diabetes mellitus with ophthalmic complications	914051000006113	422099009	3698440016
Type I diabetes mellitus with ophthalmic complications	84771000006114	739681000	3698438014
MODY - Maturity onset diabetes in youth type II	5109591000006110	237604008	356084014
Type 1 diabetes mellitus with polyneuropathy	913811000006115	713705003	3297342019
Type II diabetes mellitus with nephropathy	914271000006114	420279001	3013392012
Type I diabetes mellitus with persistent proteinuria	928471000006114	420514000	2967914011
Diabetes mellitus with hyperosmolar coma	292480018	422126006	2618221017
Diabetes mellitus with other specified manifestation	292617015	74627003	123939015
Type I diabetes mellitus with peripheral angiopathy	84781000006112	31211000119101	3315042015
Insulin-dependent diabetes without complication	746791000006111	111552007	178796018
Insulin dependent diabetes mellitus with arthropathy	771331000006116	39710007	1229552016
Insulin dependent diabetes mellitus with mononeuropathy	771371000006118	230577008	345492010
Insulin dependent diabetes mellitus with retinopathy	771411000006117	4855003	9093013
Insulin dependent diab mell with neuropathic arthropathy	772131000006111	201724008	309740011
Insulin dependent diabetes maturity onset	772151000006116	73211009	121589010
IDDM-Insulin dependent diabetes mellitus	787111000006112	73211009	121589010
Diabetes mellitus type II	3209431000006116	44054006	73465010
Unstable diabetes	411891014	11530004	19931010
Diabetes mellitus autosomal dominant	483882011	609561005	2967884018
Type I diabetes mellitus - poor control	84661000006111	444073006	2872487013
Type I diabetes mellitus with multiple complications	84731000006111	422228004	2967771016
Diabetes mellitus, adult onset, with no mention of complication	12718861000006118	532411000000102	1188841000000119
Type 1 diabetes mellitus with ulcer	292540012	190368000	292540012
Type II diabetes mellitus with multiple complications	292576013	190388001	292576013
Lipoatrophic diabetes	4392141000006116	127012008	300015
Diabetes mellitus induced by steroids	616241000006112	190447002	292667010
Insulin dependent diabetes mellitus with ulcer	771421000006113	422183001	2623058011
Pre-existing diabetes mellitus in childbirth	8033301000006115	106281000119103	3005777015
Unstable diabetes mellitus	2683591000006110	11530004	2871747019
[X]Unspecified diabetes mellitus with renal complications	12703991000006112	127013003	430881000006111
Type 2 diabetes mellitus with retinopathy	84621000006117	422034002	3699410014
Non-insulin dependent diabetes mellitus with ulcer	280551000006114	190389009	292580015
Non-insulin-dependent d m with peripheral angiopath	280561000006111	314902007	459307013
Non-insulin-dependent diabetes mellitus with multiple comps	280581000006118	190388001	292578014
Diabetes mellitus - juvenile	881441000006111	73211009	881441000006111
Diabetes+ketoacidosis -no coma	881461000006110	420422005	881461000006110

Diabetes + neuropathy	881501000006110	230572002	881501000006110
Insulin-dependent diabetes maturity onset	4758121000006115	190372001	292552017
DM + persistent proteinuria	933221000006116	658061000000102	933221000006116
Diabetes mellitus NOS with other specified manifestation	12730981000006113	658061000000102	1445831000000112
Cystic fibrosis related diabetes mellitus	494831000000119	426705001	2674608014
Diabetes mellitus with ketoacidotic coma	616381000006111	26298008	44045012
Non-insulin-dependent diabetes mellitus with retinopathy	641581000006115	422034002	3035295017
Type 1 diabetes mellitus with persistent proteinuria	928461000006119	420514000	2967914011
Diabetes mellitus type 1 with diabetic Charcot's arthropathy	8022471000006114	71771000119100	3028508015
Diabetes type 2 with ketoacidosis	6977431000006113	421750000	3035594017
Diabetes mellitus, adult onset, + neurological manifestation	616481000006119	421326000	2618196010
Diabetes mellitus, adult onset, with ketoacidosis	616531000006116	421750000	2618220016
Diabetes mellitus, adult onset, with ketoacidotic coma	616541000006114	421847006	2618218019
Diabetes mellitus, juvenile type, with ketoacidosis	616641000006110	420270002	2618216015
Diabetes mellitus, juvenile type, with ketoacidotic coma	616651000006112	421075007	2618217012
Type 1 diabetes mellitus with gangrene	292541011	420825003	2618207019
Diabetes mellitus caused by non-steroid drugs without complication	6837991000006112	413183008	3290307015
Ketoacidosis in type I diabetes mellitus	6951081000006112	420270002	2618216015
Type II diabetes mellitus with exudative maculopathy	938331000006118	421779007	3698529010
Type 1 diabetes mellitus with hypoglycaemic coma	84341000006119	314771006	459161015
Type 2 diabetes mellitus	84471000006119	44054006	197761014
Type 2 diabetes mellitus with hypoglycaemic coma	84521000006116	719216001	3316336015
Type 2 diabetes mellitus with nephropathy	84551000006113	420279001	3035432019
Type 2 diabetes mellitus with ulcer	84631000006119	190389009	292579018
Type I diabetes mellitus with renal complications	84801000006111	421893009	2623054013
Type II diabetes mellitus with ulcer	85001000006117	190389009	292581016
Diabetes mellitus type 2	3209441000006114	44054006	197763012
Diabetes mellitus type 1 with ketoacidotic coma	6965571000006111	421075007	3035596015
Maturity onset diabetes in youth type 2	356085010	237604008	356085010
NIDDM with peripheral circulatory disorder	674961000006118	422166005	2623039019
Non-insulin-dependent diabetes mellitus with renal comps	587521000006111	420279001	2615535015
Diabetes mellitus, adult, + peripheral circulatory disorder	616581000006115	422166005	2618204014
Type I diabetes mellitus with diabetic cataract	913911000006110	421920002	3688521010
Type 2 diabetes mellitus with diabetic cataract	914301000006111	420756003	3688522015
Insulin-dependent diabetes mellitus with ophthalmic comps	913471000006110	25093002	3688467012
Type I diabetes mellitus with gangrene	292543014	420825003	2618207019
Diabetes mellitus, juvenile, + neurological manifestation	616671000006119	421468001	2618197018

Pre-existing diabetes mellitus, non-insulin-dependent	306113018	199230006	306113018
Type I diabetes mellitus with polyneuropathy	84791000006110	713705003	3297390014
Diabetes mellitus NOS with ophthalmic manifestation	292503016	25093002	42062018
Type 1 diabetes mellitus with diabetic cataract	913901000006112	421920002	2618234016
Type 2 diabetes mellitus with mononeuropathy	914221000006113	420436000	2618201018
Labile type I diabetes mellitus	5754621000006110	290002008	2872768015
Diabetes mellitus NOS with no mention of complication	292475010	111552007	178796018
Insulin dependent diabetes mellitus with diabetic cataract	771341000006114	43959009	73294018
Insulin dependent diabetes mellitus	772161000006119	73211009	121589010
Insulin dependent diabetes mellitus with multiple complicat	913531000006111	385041000000108	760111000000115
Type II diabetes mellitus with hypoglycaemic coma	84891000006116	719216001	3316336015
Atypical diabetes mellitus	11633211000006112	530558861000132104	3334354013
Acute complication co-occurrent and due to diabetes mellitus	3636752017	762489000	3636752017
Type II diabetes mellitus with ophthalmic complications	12702421000006111	422099009	12702421000006111
Type 2 diabetes mellitus with diabetic cataract	84501000006114	420756003	3688522015
Type I diabetes mellitus with retinopathy	84811000006114	420789003	3699407019
Diabetes mellitus, juvenile type, with renal manifestation	616661000006114	421893009	2618211013
Type 1 diabetes mellitus with renal complications	913451000006117	421893009	2618211013
Type II diabetes mellitus with ophthalmic complications	914061000006110	422099009	3698440016
Insulin-dependent diabetes mellitus with neurological comps	913501000006115	422088007	3688477014
Type 2 diabetes mellitus with gangrene	292583018	421631007	2618206011
Other specified diabetes mellitus with ophthalmic complicatn	13771000006110	25093002	42062018
Type 1 diabetes mellitus with mononeuropathy	84351000006117	420918009	2618199015
Type 1 diabetes mellitus with neurological complications	84381000006113	421468001	2618197018
Type I diabetes mellitus with gangrene	84701000006115	420825003	2618207019
Type 1 diabetes mellitus with neurological complications	913511000006117	421468001	2618197018
Diabetes mellitus, juvenile type, with hyperosmolar coma	292482014	190330002	292482014
Diabetes mellitus, adult onset, with hyperosmolar coma	292483016	190331003	292483016
Type 2 diabetes mellitus with multiple complications	292577016	190388001	292577016
Diabetes mellitus caused by insulin receptor antibodies	3291658019	75682002	3291658019
History of diabetes mellitus	4539561000006114	161445009	2986596017
Type 1 diabetes mellitus with gastroparesis	299601000000114	713702000	3698406016
Diabetes mellitus with ophthalmic manifestation	616421000006118	25093002	3688467012
Diabetes mellitus with renal manifestation	616461000006112	127013003	301016
Insulin treated Type 2 diabetes mellitus	840951000006119	237599002	2967820013
Lipoatrophic diabetes mellitus	967701000006116	127012008	300015

Pre-existing diabetes mellitus, insulin-dependent	306112011	385051000000106	760131000000111
Diabetes mellitus, adult onset, no mention of complication	616511000006110	313436004	457329019
Insulin dependent diabetes mellitus	772171000006114	73211009	121589010
Insulin dependent diabetes maturity onset	913711000006112	73211009	121589010
Insulin dependent diabetes mellitus with diabetic cataract	913891000006113	43959009	73294018
Type 2 diabetes mellitus with nephropathy	914261000006119	420279001	3035432019
DM induced by non-steroid drugs without complication	967631000006115	413183008	2474729016
Other specified diabetes mellitus with coma	12725191000006116	630521000000101	1390421000000119
Diabetes mellitus with other specified manifestation	12730971000006110	658011000000104	1445721000000115
Ketoacidotic coma in type 2 diabetes mellitus	12704991000006114	421847006	2967777017
Type II diabetes mellitus with neurological complications	12702411000006115	421326000	84931000006113
Insulin dependent diabetes mellitus with ulcer	913591000006110	422183001	2623058011
H/O: diabetes mellitus	251591016	161445009	251591016
Type 1 diabetes mellitus with retinopathy	84441000006110	420789003	3699407019
Type II diabetes mellitus with retinopathy	84991000006112	422034002	3699410014
Type 1 diabetes mellitus with gangrene	84331000006112	420825003	2618207019
Type II diabetes mellitus with gangrene	84881000006119	421631007	2618206011
Type 1 diabetes mellitus with ophthalmic complications	913481000006113	739681000	3537386015
Diabetic on oral treatment	264681018	170746002	264681018
Insulin dependent diabetes mellitus with ulcer	11927551000006115	19429009	913591000006110
Latent autoimmune diabetes mellitus in adult (LADA)	7065531000006115	426875007	2674066012
Diabetic ketoacidosis	6953861000006110	420422005	2616611010
MODY5 - maturity-onset diabetes of the young type 5	7500341000006113	609572000	3435008016
Diabetes type 1 with neurological disorder	6972451000006115	421468001	3035338016
Type II diabetes mellitus with ophthalmic complications	84951000006118	422099009	3698440016
Diabetes mellitus with peripheral circulatory disorder	616441000006113	421895002	3688501014
Type 2 diabetes mellitus with neurological complications	914071000006115	421326000	3695406015
Type 2 diabetes mellitus with retinopathy	914151000006112	422034002	3699410014
Type 2 diabetes mellitus with ketoacidotic coma	928601000006118	421847006	2618218019
Type 1 diabetes mellitus	84281000006115	46635009	197984010
Type 1 diabetes mellitus maturity onset	84301000006116	190372001	292553010
Type 1 diabetes mellitus with arthropathy	84311000006118	314893005	459292011
Type 2 diabetes mellitus without complication	84641000006112	313436004	457329019
Type I diabetes mellitus without complication	84831000006115	313435000	457325013
Type II diabetes mellitus with arthropathy	84861000006112	314903002	459311019
Non-insulin dependent diabetes mellitus with nephropathy	280531000006119	420279001	3035432019
Type 1 diabetes mellitus without complication	84461000006114	313435000	457326014
Type I diabetes mellitus with nephropathy	84741000006118	421893009	2623054013

Type II diabetes mellitus with multiple complications	84911000006119	190388001	292576013
Type II diabetes mellitus without complication	85011000006119	313436004	457330012
Non-insulin dependent diabetes mellitus with polyneuropathy	280541000006112	713706002	3297353013
Non-insulin depend diabetes mellitus with diabetic cataract	281161000006114	420756003	3035281015
Diabetes mellitus NOS with other specified manifestation	292621010	73211009	121589010
Diabetes mellitus with unspecified complication	292622015	74627003	123939015
Type I diabetes mellitus uncontrolled	7287621000006117	444073006	2842075017
Type 1 diabetes mellitus with hypoglycaemic coma	459161015	314771006	459161015
Type 1 diabetes mellitus with neuropathic arthropathy	459296014	71771000119100	3010513018
Type II diabetes mellitus with arthropathy	459311019	314903002	459311019
Diabetes mellitus -adult onset	881451000006113	532411000000102	881451000006113
Diabetes + other complications	881521000006117	658011000000104	881521000006117
Diabetic on diet only	264679015	170745003	264679015
Type 2 diabetes mellitus without complication	457329019	313436004	457329019
Type II diabetes mellitus without complication	457330012	313436004	457330012
Diabetic on insulin and oral treatment	458512016	314194001	458512016
Type II diabetes mellitus with neuropathic arthropathy	459312014	314904008	459312014
Type 2 diabetes mellitus with neuropathic arthropathy	459313016	314904008	459313016
Maturity onset diabetes of the young, type 2	5109561000006119	237604008	2967875018
Diabetic ketoacidosis without coma	178798017	111556005	178798017
Type 2 diabetes mellitus with hypoglycaemic coma	459167016	719216001	3316336015
Maturity onset diabetes in youth type 1	2476117016	609562003	2967867012
Other specified diabetes mellitus with unspecified comps	13811000006110	74627003	123939015
Diabetes mellitus type I	3253231000006110	46635009	77728011
Type I diabetes mellitus with ulcer	292538019	190368000	292538019
Type 2 diabetes mellitus with ulcer	292579018	190389009	292579018
Type II diabetes mellitus with ulcer	292581016	190389009	292581016
Pre-existing malnutrition-related diabetes mellitus	306114012	199231005	306114012
Type 1 diabetes mellitus - poor control	292548017	444073006	2872487013
Lipoatrophic diabetes mellitus without complication	189711000000119	112991000000101	189711000000119
Secondary diabetes mellitus	15518018	8801005	15518018
Type 2 diabetes mellitus - poor control	292590011	443694000	2921019012
Brittle diabetes	19931010	11530004	19931010
[X]Pre-existing diabetes mellitus, unspecified	308110013	385051000000106	760131000000111
Insulin-dependent diabetes without complication	457327017	111552007	178796018
Insulin dependent diabetes mellitus with arthropathy	459293018	39710007	1229552016
Insulin dependent diabetes mellitus with hypoglycaemic coma	771361000006113	237632004	356141011

Insulin dependent diabetes mellitus with mononeuropathy	913771000006115	230577008	345492010
Insulin dependent diabetes mellitus with polyneuropathy	913801000006118	49455004	82373015
Ketoacidotic coma in type I diabetes mellitus	6965561000006116	421075007	2618217012
Type 1 diabetes mellitus	197984010	46635009	197984010
Type II diabetes mellitus	493774016	44054006	493774016
Diabetes mellitus due to insulin receptor antibodies	125705011	75682002	125705011
Insulin-dependent diabetes mellitus secretory diarrhoea syndrome	356111014	237618001	356111014
Lipodystrophic diabetes	4392151000006119	127012008	205214013
Maturity onset diabetes of the young, type 1	7500191000006118	609562003	2967867012
Latent autoimmune diabetes mellitus in adult	2674067015	426875007	2674067015
Other specified diabetes mellitus with other spec comps	13781000006113	73211009	121589010
Unstable type I diabetes mellitus	72721000006113	290002008	429970018
Type 2 diabetes mellitus with multiple complications	84541000006111	190388001	292577016
Type I diabetes mellitus	84651000006114	46635009	494564012
Type I diabetes mellitus maturity onset	84671000006116	190372001	292551012
Type I diabetes mellitus with neuropathic arthropathy	84761000006119	71771000119100	3010513018
Type II diabetes mellitus - poor control	84851000006110	443694000	2921019012
Type II diabetes mellitus with neuropathic arthropathy	84941000006115	314904008	459312014
Type I diabetes mellitus with polyneuropathy	913821000006111	713705003	3297390014
Insulin dependent diabetes mellitus	77727018	73211009	121589010
Insulin dependent diabetes mellitus with gangrene	771351000006111	422275004	2616612015
Insulin dependent diabetes mellitus with nephropathy	771391000006117	127013003	301016
Insulin dependent diabetes mellitus with gangrene	913621000006112	422275004	2616612015
Pre-existing diabetes mellitus in pregnancy	7500211000006117	609563008	2967860014
Secondary pancreatic diabetes mellitus	1230929011	51002006	1230929011
Diabetes mellitus induced by non-steroid drugs	2160090014	408540003	2160090014
Acute complication with diabetes mellitus	3636751012	762489000	3636751012
Insulin dependent diabetes mellitus with hypoglycaemic coma	6050041000006115	314771006	459162010
Secondary pancreatic diabetes mellitus without complication	198461000000116	51002006	84990019
Brittle type 1 diabetes mellitus	5754581000006110	290002008	2872766016
Diabetes mellitus with gangrene	616351000006115	422275004	2616612015
Diabetes mellitus, adult, + other specified manifestation	616571000006118	73211009	121589010
Diabetes with gangrene	616831000006118	422275004	2616612015
Type I diabetes mellitus with multiple complications	913551000006116	422228004	2967771016
Type II diabetes mellitus with persistent proteinuria	928551000006115	421986006	2967751013
Diabetic acidosis with coma	2920971000006115	26298008	44046013
Insulin dependent diabetes mellitus with hypoglycemic coma	6050071000006111	314771006	459165012

Diabetes mellitus caused by non-steroid drugs	6763751000006119	408540003	3290374011
Type 2 diabetes mellitus with persistent microalbuminuria	928561000006118	420715001	2967769016
Type I diabetes mellitus	494564012	46635009	494564012
Type II diabetes mellitus with nephropathy	84921000006110	420279001	3013392012
Non-insulin dependent diabetes mellitus	280571000006116	44054006	493773010
Type 2 diabetes mellitus with polyneuropathy	914241000006118	713706002	3297353013
Type 1 diabetes mellitus with ketoacidotic coma	928521000006112	421075007	2967758019
Type II diabetes mellitus with ketoacidotic coma	928611000006115	421847006	2618218019
Diabetic on oral treatment and glucagon-like peptide 1	1966311000006116	976341000000101	2483721000000113
Pre-existing type 2 diabetes mellitus in pregnancy	1988741000006117	237627000	356133011
Other specified diabetes mellitus with coma	292488013	420662003	2618230013
Unstable insulin dependent diabetes mellitus	429972014	11530004	19931010
Diabetic on subcutaneous treatment	1177721000000112	527691000000107	1177721000000112
Type 2 diabetes mellitus uncontrolled	7281511000006118	443694000	3321322011
Diabetes type 1 with cataract	6980441000006114	421920002	3035484013
Diabetes mellitus NOS with peripheral circulatory disorder	292523015	421895002	2618203015
Pre-existing type 2 diabetes mellitus	4796321000006113	199230006	2967812011
Non-insulin dependent diabetes mellitus - poor control	281171000006119	443694000	2842387018
Diabetes mellitus with no mention of complication	292466013	111552007	178796018
Unstable insulin dependent diabetes mellitus	72651000006114	11530004	19931010
Unstable type 1 diabetes mellitus	429971019	290002008	429971019
Insulin treated Type II diabetes mellitus	1223147012	237599002	1223147012
Pre-existing diabetes mellitus	4539551000006112	161445009	2820057017
Type 2 diabetes mellitus with ketoacidosis	928581000006111	421750000	2967754017
Unspecified diabetes mellitus with multiple complications	292565014	385041000000108	760111000000115
Diabetic on non-insulin injectable	1780981000006115	1780981000006104	1780981000006115
Type I diabetes mellitus with hypoglycaemic coma	459163017	314771006	459163017
Type 2 diabetes mellitus with gastroparesis	299621000000117	713703005	3698412014
Type 1 diabetes mellitus with exudative maculopathy	938301000006114	420486006	3698521013
Type I diabetes mellitus with neuropathic arthropathy	459295013	71771000119100	3010513018
Type 1 diabetes mellitus with neuropathic arthropathy	84391000006111	71771000119100	3010513018
Type 2 diabetes mellitus with renal complications	84611000006113	420279001	2618208012
Type II diabetes mellitus	84841000006113	44054006	493774016
Type II diabetes mellitus with peripheral angiopathy	84961000006116	314902007	459306016
Type I diabetes mellitus with diabetic cataract	84691000006115	421920002	3688521010
Diabetes mellitus with neurological manifestation	616391000006114	422088007	3688477014
Type 2 diabetes mellitus with exudative maculopathy	938321000006116	421779007	2618237011
Non-insulin-dependent diabetes mellitus with neuro comps	280591000006115	421326000	3695406015

Non-insulin dependent diabetes mellitus with hypoglyca coma	280511000006113	719216001	3316336015
Diabetes mellitus uncontrolled	5505651000006113	268519009	2920470017
Diabetic on non-insulin injectable medication	2460251000000115	719566006	3316952014
Brittle type I diabetes mellitus	5754611000006119	290002008	2872767013
Diabetes type 1 with retinopathy	6960441000006112	420789003	3035448018
Type 1 diabetes mellitus with renal complications	84431000006117	421893009	2618211013
Type 2 diabetes mellitus with neurological complications	84561000006110	421326000	3695406015
Type 2 diabetes mellitus with ophthalmic complications	84581000006117	422099009	3698440016
Type II diabetes mellitus with gangrene	292582011	421631007	2618206011

Table S11. Anti-Diabetic Aurum product codes

Term from EMIS	Prod code ID	dmd ID	Drug substance name
Insulin aspart 100units/ml solution for injection 3ml cartridges	1862041000033113	3468611000001108	Insulin aspart
NovoRapid Penfill 100units/ml solution for injection 3ml cartridges (Novo Nordisk Ltd)	1862341000033110	3279211000001105	Insulin aspart
Fiasp Penfill 100units/ml solution for injection 3ml cartridges (Novo Nordisk Ltd)	12186641000033113	34043211000001101	Insulin aspart
NovoRapid Novolet 100units/ml solution for injection (Novo Nordisk Ltd)	1862441000033116	3281611000001102	Insulin aspart
NovoRapid FlexPen 100units/ml solution for injection 3ml pre-filled pens (Novo Nordisk Ltd)	2796641000033115	3282211000001106	Insulin aspart
Insulin aspart 100units/ml solution for injection 3ml pre-filled disposable devices	3277741000033118	3468711000001104	Insulin aspart
NovoRapid FlexTouch 100units/ml solution for injection 3ml pre-filled pens (Novo Nordisk Ltd)	6456441000033119	19570211000001106	Insulin aspart
Fiasp FlexTouch 100units/ml solution for injection 3ml pre-filled pens (Novo Nordisk Ltd)	12186541000033112	34043011000001106	Insulin aspart
NovoMix 30 Penfill 100units/ml suspension for injection 3ml cartridges (Novo Nordisk Ltd)	2724741000033111	3277811000001103	Insulin aspart/ Insulin aspart protamine
NovoMix 30 FlexPen 100units/ml suspension for injection 3ml pre-filled pens (Novo Nordisk Ltd)	2724841000033118	3277211000001104	Insulin aspart/ Insulin aspart protamine
Human Mixtard 10 Penfill 100units/ml suspension for injection 1.5ml cartridges (Novo Nordisk Ltd)	1818141000033110	3268711000001103	Insulin isophane human/ Insulin soluble human
Mixtard 10 Penfill 100units/ml suspension for injection 3ml cartridges (Novo Nordisk Ltd)	2953541000033112	3264711000001106	Insulin isophane human/ Insulin soluble human
Mixtard 10 NovoLet 100units/ml suspension for injection (Novo Nordisk Ltd)	2953441000033111	3269711000001107	Insulin isophane human/ Insulin soluble human
Insuman Comb 15 100units/ml suspension for injection 3ml cartridges (Sanofi)	2158541000033117	3273911000001104	Insulin isophane human/ Insulin soluble human
Insuman Comb 15 100units/ml suspension for injection 3ml pre-filled OptiSet pens (Sanofi)	2267041000033118	3270211000001103	Insulin isophane human/ Insulin soluble human
Insuman Comb 15 100units/ml suspension for injection 5ml vials (Aventis Pharma)	2158441000033118	3271711000001102	Insulin isophane human/ Insulin soluble human
Human Mixtard 20 Penfill 100units/ml suspension for injection 1.5ml cartridges (Novo Nordisk Ltd)	1818241000033115	3267811000001107	Insulin isophane human/ Insulin soluble human
Mixtard 20 Penfill 100units/ml suspension for injection 3ml cartridges (Novo Nordisk Ltd)	2953741000033116	3264411000001100	Insulin isophane human/ Insulin soluble human
Humulin M2 100units/ml suspension for injection 3ml cartridges (Eli Lilly and Company Ltd)	1616241000033111	3263611000001100	Insulin isophane human/ Insulin soluble human
Mixtard 20 NovoLet 100units/ml suspension for injection (Novo Nordisk Ltd)	2953641000033113	3266111000001103	Insulin isophane human/ Insulin soluble human
Insuman Comb 25 100units/ml suspension for injection 3ml cartridges (Sanofi)	2035641000033115	3264111000001105	Insulin isophane human/ Insulin soluble human
Insuman Comb 25 100units/ml suspension for injection 3ml pre-filled OptiSet pens (Sanofi)	2267141000033119	3259811000001109	Insulin isophane human/ Insulin soluble human

Insuman Comb 25 100units/ml suspension for injection 3ml pre-filled SoloStar pens (Sanofi)	6011841000033112	18150311000001109	Insulin isophane human/ Insulin soluble human
Insuman Comb 25 100units/ml suspension for injection 5ml vials (Sanofi)	2035541000033116	3263011000001107	Insulin isophane human/ Insulin soluble human
Human Mixtard 30 Penfill 100units/ml suspension for injection 1.5ml cartridges (Novo Nordisk Ltd)	1818341000033113	3275311000001104	Insulin isophane human/ Insulin soluble human
Humulin M3 100units/ml suspension for injection 10ml vials (Eli Lilly and Company Ltd)	725341000033110	3274811000001107	Insulin isophane human/ Insulin soluble human
Mixtard 30 100units/ml suspension for injection 10ml vials (Novo Nordisk Ltd)	2953941000033118	3275011000001102	Insulin isophane human/ Insulin soluble human
Mixtard 30 Penfill 100units/ml suspension for injection 3ml cartridges (Novo Nordisk Ltd)	2954041000033116	3273111000001102	Insulin isophane human/ Insulin soluble human
Humulin M3 100units/ml suspension for injection 3ml cartridges (Eli Lilly and Company Ltd)	1616341000033118	3273611000001105	Insulin isophane human/ Insulin soluble human
HumaJect M3 Pen 100units/ml suspension for injection (Eli Lilly and Company Ltd)	727341000033117	3272211000001102	Insulin isophane human/ Insulin soluble human
Mixtard 30 InnoLet 100units/ml suspension for injection 3ml pre-filled pens (Novo Nordisk Ltd)	2644741000033116	3271611000001106	Insulin isophane human/ Insulin soluble human
Mixtard 30 NovoLet 100units/ml suspension for injection (Novo Nordisk Ltd)	2953841000033114	3270511000001100	Insulin isophane human/ Insulin soluble human
Humulin M3 Pen 100units/ml suspension for injection 3ml pre-filled pens (Eli Lilly and Company Ltd)	3333841000033113	9437511000001101	Insulin isophane human/ Insulin soluble human
Humulin M3 KwikPen 100units/ml suspension for injection 3ml pre-filled pens (Eli Lilly and Company Ltd)	5910741000033117	17609511000001109	Insulin isophane human/ Insulin soluble human
Human Mixtard 40 Penfill 100units/ml suspension for injection 1.5ml cartridges (Novo Nordisk Ltd)	1818441000033119	3285511000001103	Insulin isophane human/ Insulin soluble human
Mixtard 40 Penfill 100units/ml suspension for injection 3ml cartridges (Novo Nordisk Ltd)	2954241000033112	3281211000001104	Insulin isophane human/ Insulin soluble human
Mixtard 40 NovoLet 100units/ml suspension for injection (Novo Nordisk Ltd)	2954141000033117	3272011000001107	Insulin isophane human/ Insulin soluble human
Human Mixtard 50 Penfill 100units/ml suspension for injection 1.5ml cartridges (Novo Nordisk Ltd)	1818541000033118	3273411000001107	Insulin isophane human/ Insulin soluble human
Humulin M5 100units/ml suspension for injection 10ml vials (Eli Lilly and Company Ltd)	722841000033112	3271011000001104	Insulin isophane human/ Insulin soluble human
Insuman Comb 50 100units/ml suspension for injection 3ml cartridges (Sanofi)	2158741000033113	3272411000001103	Insulin isophane human/ Insulin soluble human
Mixtard 50 Penfill 100units/ml suspension for injection 3ml cartridges (Novo Nordisk Ltd)	2954541000033114	3272811000001101	Insulin isophane human/ Insulin soluble human
Mixtard 50 NovoLet 100units/ml suspension for injection (Novo Nordisk Ltd)	2954341000033119	3278311000001108	Insulin isophane human/ Insulin soluble human
Insuman Comb 50 100units/ml suspension for injection 3ml pre-filled OptiSet pens (Sanofi)	2267241000033114	3278611000001103	Insulin isophane human/ Insulin soluble human

Insuman Comb 50 100units/ml suspension for injection 5ml vials (Aventis Pharma)	2158641000033116	3274511000001109	Insulin isophane human/ Insulin soluble human
Hypurin Porcine 30/70 Mix 100units/ml suspension for injection 1.5ml cartridges (C P Pharmaceuticals Ltd)	2018941000033112	3277711000001106	Insulin isophane porcine/ Insulin soluble porcine
Hypurin Porcine 30/70 Mix 100units/ml suspension for injection 10ml vials (Wockhardt UK Ltd)	2018841000033116	3266811000001105	Insulin isophane porcine/ Insulin soluble porcine
Pork Mixtard 30 100units/ml suspension for injection 10ml vials (Novo Nordisk Ltd)	915641000033117	3267911000001102	Insulin isophane porcine/ Insulin soluble porcine
Hypurin Bovine Isophane 100units/ml suspension for injection 10ml vials (Wockhardt UK Ltd)	735841000033116	3280011000001100	Insulin isophane bovine
Insulin isophane bovine 100units/ml suspension for injection 10ml vials	1821641000033119	3472311000001106	Insulin isophane bovine
Human Insulatard Penfill 100units/ml suspension for injection 1.5ml cartridges (Novo Nordisk Ltd)	1714041000033116	3259411000001107	Insulin isophane human
Humulin I 100units/ml suspension for injection 10ml vials (Eli Lilly and Company Ltd)	724041000033112	3255911000001101	Insulin isophane human
Insulatard 100units/ml suspension for injection 10ml vials (Novo Nordisk Ltd)	2953241000033110	3256111000001105	Insulin isophane human
Insulin isophane human 100units/ml suspension for injection 10ml vials	779141000033111	3472511000001100	Insulin isophane human
Insuman Basal 100units/ml suspension for injection 5ml vials (Sanofi)	2035241000033118	3258411000001104	Insulin isophane human
Insulin isophane porcine 100units/ml suspension for injection 10ml vials	1821441000033116	3473011000001104	Insulin isophane porcine
Hypurin Porcine Isophane 100units/ml suspension for injection 10ml vials (Wockhardt UK Ltd)	734941000033111	3284011000001107	Insulin isophane porcine
Pork Insulatard 100units/ml suspension for injection 10ml vials (Novo Nordisk Ltd)	763341000033116	3333111000001107	Insulin isophane porcine
Humalog Mix25 Pen 100units/ml suspension for injection 3ml pre-filled pens (Waymade Healthcare Plc)	1737041000033117	5268611000001103	Insulin lispro/ Insulin lispro protamine
Humalog Mix25 KwikPen 100units/ml suspension for injection 3ml pre-filled pens (Eli Lilly and Company Ltd)	4608641000033119	13884711000001104	Insulin lispro/ Insulin lispro protamine
Humalog Mix50 KwikPen 100units/ml suspension for injection 3ml pre-filled pens (Eli Lilly and Company Ltd)	4608741000033111	13884911000001102	Insulin lispro/ Insulin lispro protamine
Humalog Mix50 Pen 100units/ml suspension for injection 3ml pre-filled pens (Sigma Pharmaceuticals Plc)	2182141000033117	14248811000001102	Insulin lispro/ Insulin lispro protamine
Humalog Mix25 100units/ml suspension for injection 3ml cartridges (Eli Lilly and Company Ltd)	1736941000033118	3275711000001100	Insulin lispro/ Insulin lispro protamine
Hypurin Bovine Lente 100units/ml suspension for injection 10ml vials (Wockhardt UK Ltd)	735941000033112	3280611000001107	Insulin zinc suspension mixed bovine
Hypurin Porcine 30/70 Mix 100units/ml suspension for injection 3ml cartridges (Wockhardt UK Ltd)	2917341000033116	4029411000001100	Insulin isophane porcine/ Insulin soluble porcine
Hypurin Bovine Isophane 100units/ml suspension for injection 3ml cartridges (Wockhardt UK Ltd)	2917041000033118	4028311000001101	Insulin isophane bovine
Insulin isophane bovine 100units/ml suspension for injection 3ml cartridges	1821741000033111	4033211000001103	Insulin isophane bovine
Hypurin Bovine Neutral 100units/ml solution for injection 3ml cartridges (Wockhardt UK Ltd)	2916941000033119	4028811000001105	Insulin soluble bovine
Insulin isophane porcine 100units/ml suspension for injection 3ml cartridges	1821541000033115	4053611000001100	Insulin isophane porcine
Hypurin Porcine Isophane 100units/ml suspension for injection 3ml cartridges (Wockhardt UK Ltd)	2917241000033114	4034311000001108	Insulin isophane porcine

Hypurin Porcine Neutral 100units/ml solution for injection 3ml cartridges (Wockhardt UK Ltd)	2917141000033119	4034911000001109	Insulin soluble porcine
Insulin detemir 100units/ml solution for injection 3ml pre-filled disposable devices	3136941000033116	7594211000001102	Insulin detemir
Levemir FlexPen 100units/ml solution for injection 3ml pre-filled pens (Novo Nordisk Ltd)	3137241000033111	7589411000001100	Insulin detemir
Levemir InnoLet 100units/ml solution for injection 3ml pre-filled pens (Novo Nordisk Ltd)	4126541000033110	11148111000001101	Insulin detemir
Insulin detemir 100units/ml solution for injection 3ml cartridges	3137041000033115	7597611000001106	Insulin detemir
Levemir Penfill 100units/ml solution for injection 3ml cartridges (Novo Nordisk Ltd)	3137141000033116	7589911000001108	Insulin detemir
Insulin glulisine 100units/ml solution for injection 3ml cartridges	3345741000033117	9532111000001100	Insulin glulisine
Apidra 100units/ml solution for injection 3ml cartridges (Sanofi)	3345941000033119	9528311000001103	Insulin glulisine
Apidra 100units/ml solution for injection 3ml OptiClik cartridges (Sanofi)	3963941000033117	10898411000001103	Insulin glulisine
Insulin glulisine 100units/ml solution for injection 3ml pre-filled disposable devices	3914241000033112	10097211000001102	Insulin glulisine
Apidra 100units/ml solution for injection 3ml pre-filled SoloStar pens (Sanofi)	4258641000033112	12144611000001100	Insulin glulisine
Humalog Mix50 100units/ml suspension for injection 3ml cartridges (Eli Lilly and Company Ltd)	3922641000033111	10344911000001108	Insulin lispro/ Insulin lispro protamine
Exubera 1mg inhalation powder blisters (Pfizer Ltd)	3945141000033114	10690511000001105	Insulin human
Exubera 3mg inhalation powder blisters (Pfizer Ltd)	3945241000033119	10690811000001108	Insulin human
Insulin human 100units/ml solution for injection 10ml vials	10673941000033110	13859411000001101	Insulin human
Insuman Infusat 100units/ml solution for injection 10ml vials (Sanofi)	10674141000033111	18030311000001100	Insulin human
Humulin R 500units/ml solution for injection 20ml vials (Imported (United States))	4423041000033114	15603111000001104	Insulin human
Humalog Mix25 100units/ml suspension for injection 10ml vials (Eli Lilly and Company Ltd)	5403541000033118	16530311000001107	Insulin lispro/ Insulin lispro protamine
Insulin human 100units/ml solution for injection 3.15ml cartridges	10674041000033112	18083911000001104	Insulin human
Insuman Infusat 100units/ml solution for injection 3.15ml cartridges (Sanofi)	10674341000033114	18046311000001103	Insulin human
Insulin degludec 100units/ml solution for injection 3ml cartridges	8264341000033118	21939511000001100	Insulin degludec
Tresiba Penfill 100units/ml solution for injection 3ml cartridges (Novo Nordisk Ltd)	8264441000033112	21928511000001109	Insulin degludec
Tresiba FlexTouch 100units/ml solution for injection 3ml pre-filled pens (Novo Nordisk Ltd)	8264141000033116	21930011000001100	Insulin degludec
Insulin degludec 100units/ml solution for injection 3ml pre-filled disposable devices	8263941000033117	21939611000001101	Insulin degludec
Insulin degludec 200units/ml solution for injection 3ml pre-filled disposable devices	8264041000033115	21939711000001105	Insulin degludec
Tresiba FlexTouch 200units/ml solution for injection 3ml pre-filled pens (Novo Nordisk Ltd)	8264241000033111	21931911000001108	Insulin degludec
Insulin aspart 100units/ml solution for injection 1.6ml cartridges	9677041000033116	26655811000001102	Insulin aspart
NovoRapid PumpCart 100units/ml solution for injection 1.6ml cartridges (Novo Nordisk Ltd)	9677141000033117	26209611000001109	Insulin aspart
Insulin degludec 100units/ml / Liraglutide 3.6mg/ml solution for injection 3ml pre-filled disposable devices	10044541000033113	28279611000001109	Insulin degludec/ Liraglutide
Xultophy 100units/ml / 3.6mg/ml solution for injection 3ml pre-filled pens (Novo Nordisk Ltd)	10044641000033114	28054311000001106	Insulin degludec/ Liraglutide

Lyumjev KwikPen 200units/ml solution for injection 3ml pre-filled pens (Eli Lilly and Company Ltd)	13604941000033116	38501011000001104	Insulin lispro
Insulin lispro 200units/ml solution for injection 3ml pre-filled disposable devices	10252641000033111	28989711000001106	Insulin lispro
Humalog KwikPen 200units/ml solution for injection 3ml pre-filled pens (Eli Lilly and Company Ltd)	10252741000033119	28926811000001100	Insulin lispro
Insulin glargine 300units/ml solution for injection 1.5ml pre-filled disposable devices	10494241000033110	29903611000001103	Insulin glargine
Toujeo 300units/ml solution for injection 1.5ml pre-filled SoloStar pens (Sanofi)	10494441000033111	29866811000001104	Insulin glargine
Humulin R KwikPen 500units/ml solution for injection 3ml pre-filled pens (Imported (United States))	12481641000033115	35214311000001104	Insulin human
NovoRapid 100units/ml solution for injection 10ml vials (Novo Nordisk Ltd)	1862241000033117	3280711000001103	Insulin aspart
Insulin aspart 100units/ml solution for injection 10ml vials	1861941000033119	36047011000001105	Insulin aspart
Fiasp 100units/ml solution for injection 10ml vials (Novo Nordisk Ltd)	12186441000033111	34043411000001102	Insulin aspart
Lantus 100units/ml solution for injection 10ml vials (Sanofi)	2780741000033111	3287911000001100	Insulin glargine
Insulin glargine 100units/ml solution for injection 10ml vials	2798841000033115	36047111000001106	Insulin glargine
Apidra 100units/ml solution for injection 10ml vials (Sanofi)	3345841000033110	9528811000001107	Insulin glulisine
Insulin glulisine 100units/ml solution for injection 10ml vials	3345641000033114	36047211000001100	Insulin glulisine
Insuman Basal 100units/ml suspension for injection 3ml cartridges (Sanofi)	2035441000033117	3265011000001108	Insulin isophane human
Humulin I 100units/ml suspension for injection 3ml cartridges (Eli Lilly and Company Ltd)	1615941000033113	3264211000001104	Insulin isophane human
Insulatard Penfill 100units/ml suspension for injection 3ml cartridges (Novo Nordisk Ltd)	2953341000033117	3263711000001109	Insulin isophane human
Insulatard InnoLet 100units/ml suspension for injection 3ml pre-filled pens (Novo Nordisk Ltd)	2644641000033113	3261411000001102	Insulin isophane human
Insulatard FlexPen 100units/ml suspension for injection (Novo Nordisk Ltd)	2796741000033112	3262011000001103	Insulin isophane human
Insulatard NovoLet 100units/ml suspension for injection (Novo Nordisk Ltd)	2953141000033115	3262511000001106	Insulin isophane human
Insuman Basal 100units/ml suspension for injection 3ml pre-filled OptiSet pens (Sanofi)	2266841000033110	3260611000001109	Insulin isophane human
Humulin I Pen 100units/ml suspension for injection 3ml pre-filled pens (Eli Lilly and Company Ltd)	2182441000033113	3260811000001108	Insulin isophane human
Insuman Basal 100units/ml suspension for injection 3ml pre-filled SoloStar pens (Sanofi)	6389641000033111	19354411000001104	Insulin isophane human
Humulin I KwikPen 100units/ml suspension for injection 3ml pre-filled pens (Eli Lilly and Company Ltd)	5911541000033119	17608511000001103	Insulin isophane human
Humalog 100units/ml solution for injection 1.5ml cartridges (Eli Lilly and Company Ltd)	720541000033111	3282711000001104	Insulin lispro
Insulin lispro 100units/ml solution for injection 1.5ml cartridges	757641000033112	36047511000001102	Insulin lispro
Humalog 100units/ml solution for injection 10ml vials (Eli Lilly and Company Ltd)	723241000033118	3280111000001104	Insulin lispro
Insulin lispro 100units/ml solution for injection 10ml vials	762441000033118	36047611000001103	Insulin lispro
Insulin lispro Sanofi 100units/ml solution for injection 10ml vials (Sanofi)	12600741000033116	35776411000001102	Insulin lispro
Lyumjev 100units/ml solution for injection 10ml vials (Eli Lilly and Company Ltd)	13605141000033117	38456911000001105	Insulin lispro
Humalog 100units/ml solution for injection 3ml cartridges (Eli Lilly and Company Ltd)	1736841000033114	3284311000001105	Insulin lispro

Lyumjev 100units/ml solution for injection 3ml cartridges (Eli Lilly and Company Ltd)	13605041000033116	38456111000001107	Insulin lispro
Insulin lispro Sanofi 100units/ml solution for injection 3ml cartridges (Sanofi)	12600841000033114	35776511000001103	Insulin lispro
Insulin lispro 100units/ml solution for injection 3ml cartridges	1739941000033110	36047711000001107	Insulin lispro
Humalog KwikPen 100units/ml solution for injection 3ml pre-filled pens (Eli Lilly and Company Ltd)	4608541000033115	13877811000001105	Insulin lispro
Humalog Pen 100units/ml solution for injection 3ml pre-filled pens (Sigma Pharmaceuticals Plc)	2182341000033119	14249011000001103	Insulin lispro
Insulin lispro 100units/ml solution for injection 3ml pre-filled disposable devices	2182241000033112	36047811000001104	Insulin lispro
Insulin lispro Sanofi 100units/ml solution for injection 3ml pre-filled pens (Sanofi)	12600941000033118	35776811000001100	Insulin lispro
Humalog Junior KwikPen 100units/ml solution for injection 3ml pre-filled pens (Eli Lilly and Company Ltd)	12486241000033111	35216811000001101	Insulin lispro
Lyumjev KwikPen 100units/ml solution for injection 3ml pre-filled pens (Eli Lilly and Company Ltd)	13604841000033112	38465811000001104	Insulin lispro
Lyumjev Junior KwikPen 100units/ml solution for injection 3ml pre-filled pens (Eli Lilly and Company Ltd)	13604741000033119	38479611000001109	Insulin lispro
Hypurin Bovine Protamine Zinc 100units/ml suspension for injection 10ml vials (Wockhardt UK Ltd)	736141000033115	3269911000001109	Insulin protamine zinc bovine
Insulin protamine zinc bovine 100units/ml suspension for injection 10ml vials	1119741000033114	36047911000001109	Insulin protamine zinc bovine
Hypurin Bovine Neutral 100units/ml solution for injection 10ml vials (Wockhardt UK Ltd)	736041000033119	3271311000001101	Insulin soluble bovine
Human Actrapid Penfill 100units/ml solution for injection 1.5ml cartridges (Novo Nordisk Ltd)	721041000033110	3309511000001105	Insulin soluble human
Actrapid 100units/ml solution for injection 10ml vials (Novo Nordisk Ltd)	2952841000033116	3312111000001108	Insulin soluble human
Velosulin 100units/ml solution for injection 10ml vials (Novo Nordisk Ltd)	2954641000033110	3312411000001103	Insulin soluble human
Humulin S 100units/ml solution for injection 10ml vials (Eli Lilly and Company Ltd)	724341000033114	3312611000001100	Insulin soluble human
Actrapid Penfill 100units/ml solution for injection 3ml cartridges (Novo Nordisk Ltd)	2953041000033119	3310711000001105	Insulin soluble human
Humulin S 100units/ml solution for injection 3ml cartridges (Eli Lilly and Company Ltd)	1616041000033115	3311311000001101	Insulin soluble human
Insuman Rapid 100units/ml solution for injection 3ml cartridges (Sanofi)	2034641000033119	3311611000001106	Insulin soluble human
Actrapid NovoLet 100units/ml solution for injection (Novo Nordisk Ltd)	2952941000033112	3288511000001106	Insulin soluble human
HumaJect S Pen 100units/ml solution for injection (Eli Lilly and Company Ltd)	727041000033119	3290411000001108	Insulin soluble human
Insuman Rapid 100units/ml solution for injection 3ml pre-filled OptiSet pens (Sanofi)	2266941000033119	3291711000001105	Insulin soluble human
Insuman Rapid 100units/ml solution for injection 5ml vials (Aventis Pharma)	2034441000033116	3294911000001105	Insulin soluble human
Hypurin Porcine Neutral 100units/ml solution for injection 10ml vials (Wockhardt UK Ltd)	735441000033119	3285011000001106	Insulin soluble porcine
Pork Actrapid 100units/ml solution for injection 10ml vials (Novo Nordisk Ltd)	1749441000033116	3285611000001104	Insulin soluble porcine
Insulin soluble porcine 100units/ml solution for injection 10ml vials	12624241000033111	36048711000001108	Insulin soluble porcine
Ultratard 100units/ml suspension for injection 10ml vials (Novo Nordisk Ltd)	2954841000033111	3282311000001103	Insulin zinc suspension crystalline human

Humulin Zn 100units/ml suspension for injection 10ml vials (Eli Lilly and Company Ltd)	724541000033119	3281811000001103	Insulin zinc suspension crystalline human
Humulin Lente 100units/ml suspension for injection 10ml vials (Eli Lilly and Company Ltd)	725841000033118	3284911000001106	Insulin zinc suspension mixed human
Monotard 100units/ml suspension for injection 10ml vials (Novo Nordisk Ltd)	2954741000033118	3284111000001108	Insulin zinc suspension mixed human
Suliqua 100units/ml / 33micrograms/ml solution for injection 3ml pre-filled SoloStar pens (Sanofi)	12902541000033110	36620611000001107	Insulin glargine/ Lixisenatide
Insulin glargine 100units/ml / Lixisenatide 33micrograms/ml solution for injection 3ml pre-filled disposable devices	12902741000033119	36630511000001105	Insulin glargine/ Lixisenatide
Insulin glargine 100units/ml / Lixisenatide 50micrograms/ml solution for injection 3ml pre-filled disposable devices	12902841000033112	36630611000001109	Insulin glargine/ Lixisenatide
Suliqua 100units/ml / 50micrograms/ml solution for injection 3ml pre-filled SoloStar pens (Sanofi)	12902641000033111	36618311000001109	Insulin glargine/ Lixisenatide
Toujeo 300units/ml solution for injection 3ml pre-filled DoubleStar pens (Sanofi)	12998841000033112	36911311000001101	Insulin glargine
Insulin glargine 300units/ml solution for injection 3ml pre-filled disposable devices	12998741000033119	36931811000001104	Insulin glargine
Lantus 100units/ml solution for injection 3ml cartridges (Sanofi)	2780841000033118	3284211000001102	Insulin glargine
Lantus 100units/ml solution for injection 3ml OptiClik cartridges (Sanofi)	3869341000033114	10250211000001101	Insulin glargine
Abasaglar 100units/ml solution for injection 3ml cartridges (Eli Lilly and Company Ltd)	10600641000033118	30172211000001102	Insulin glargine
Insulin glargine 100units/ml solution for injection 3ml cartridges	2798941000033111	38896911000001108	Insulin glargine
Lantus 100units/ml solution for injection 3ml pre-filled OptiSet pens (Sanofi)	2780941000033114	3283211000001100	Insulin glargine
Lantus 100units/ml solution for injection 3ml pre-filled SoloStar pens (Sanofi)	4258541000033111	11933011000001106	Insulin glargine
Insulin glargine 100units/ml solution for injection 3ml pre-filled disposable devices	2799041000033119	38897011000001107	Insulin glargine
Abasaglar KwikPen 100units/ml solution for injection 3ml pre-filled pens (Eli Lilly and Company Ltd)	10600741000033110	30171811000001105	Insulin glargine
Semglee 100units/ml solution for injection 3ml pre-filled pens (Mylan)	12686641000033112	36082811000001107	Insulin glargine
Acarbose 50mg tablets	11541000033110	326037007	Acarbose
Glucobay 50mg tablets (Bayer Plc)	645341000033115	781411000001104	Acarbose
Glucobay 100mg tablets (Bayer Plc)	645241000033113	444311000001106	Acarbose
Acarbose 100mg tablets	11441000033114	326038002	Acarbose
Eperzan 30mg powder and solvent for solution for injection pre-filled pens (GlaxoSmithKline UK Ltd)	10952341000033119	31014311000001106	Albiglutide
Albiglutide 30mg powder and solvent for solution for injection pre-filled disposable devices	10951041000033114	31015711000001109	Albiglutide
Albiglutide 50mg powder and solvent for solution for injection pre-filled disposable devices	10951141000033113	31015811000001101	Albiglutide
Eperzan 50mg powder and solvent for solution for injection pre-filled pens (GlaxoSmithKline UK Ltd)	10953041000033113	31014611000001101	Albiglutide
Vipdomet 12.5mg/1000mg tablets (Takeda UK Ltd)	8959941000033110	23632611000001103	Alogliptin benzoate/ Metformin hydrochloride
Alogliptin 12.5mg / Metformin 1g tablets	8959841000033119	23637211000001102	Alogliptin benzoate/

			Metformin hydrochloride
Alogliptin 12.5mg tablets	8959341000033111	23637311000001105	Alogliptin benzoate
Vipidia 12.5mg tablets (Takeda UK Ltd)	8959641000033115	23636011000001109	Alogliptin benzoate
Vipidia 25mg tablets (Takeda UK Ltd)	8959741000033112	23636311000001107	Alogliptin benzoate
Alogliptin 25mg tablets	8959441000033117	23637411000001103	Alogliptin benzoate
Alogliptin 6.25mg tablets	8959241000033118	23637511000001104	Alogliptin benzoate
Vipidia 6.25mg tablets (Takeda UK Ltd)	8959541000033116	23634111000001103	Alogliptin benzoate
Canagliflozin 100mg tablets	9110341000033112	703682001	Canagliflozin hemihydrate
Invokana 100mg tablets (Napp Pharmaceuticals Ltd)	9110541000033117	24088611000001101	Canagliflozin hemihydrate
Canagliflozin 300mg tablets	9110441000033118	24104511000001103	Canagliflozin hemihydrate
Invokana 300mg tablets (Napp Pharmaceuticals Ltd)	9110641000033116	24088311000001106	Canagliflozin hemihydrate
Canagliflozin 50mg / Metformin 1g tablets	9851541000033114	28049211000001101	Canagliflozin hemihydrate/ Metformin hydrochloride
Vokanamet 50mg/1000mg tablets (Napp Pharmaceuticals Ltd)	9851841000033111	28024411000001103	Canagliflozin hemihydrate/ Metformin hydrochloride
Vokanamet 50mg/850mg tablets (Napp Pharmaceuticals Ltd)	9851741000033118	28022511000001109	Canagliflozin hemihydrate/ Metformin hydrochloride
Canagliflozin 50mg / Metformin 850mg tablets	9851641000033110	28049311000001109	Canagliflozin hemihydrate/ Metformin hydrochloride
Chlorpropamide 100mg tablets	249441000033114	325213005	Chlorpropamide
Chlorpropamide 250mg tablets	249541000033110	325214004	Chlorpropamide
Forxiga 5mg tablets (AstraZeneca UK Ltd)	8199541000033113	21609511000001105	Dapagliflozin propanediol monohydrate
Dapagliflozin 5mg tablets	8199341000033118	703679006	Dapagliflozin propanediol monohydrate
Dapagliflozin 10mg tablets	8199441000033112	703680009	Dapagliflozin propanediol monohydrate
Forxiga 10mg tablets (AstraZeneca UK Ltd)	8199641000033114	21609811000001108	Dapagliflozin propanediol monohydrate
Xigduo 5mg/1000mg tablets (AstraZeneca UK Ltd)	9106241000033119	24018511000001102	Dapagliflozin propanediol monohydrate/ Metformin hydrochloride
Dapagliflozin 5mg / Metformin 1g tablets	9106041000033110	24054611000001100	Dapagliflozin propanediol monohydrate/ Metformin hydrochloride

Dapagliflozin 5mg / Metformin 850mg tablets	9106141000033114	24054711000001109	Dapagliflozin propanediol monohydrate/ Metformin hydrochloride
Xigduo 5mg/850mg tablets (AstraZeneca UK Ltd)	9106341000033112	24018111000001106	Dapagliflozin propanediol monohydrate/ Metformin hydrochloride
Saxagliptin 5mg / Dapagliflozin 10mg tablets	11898041000033111	33745311000001109	Dapagliflozin propanediol monohydrate/ Saxagliptin hydrochloride
Qtern 5mg/10mg tablets (AstraZeneca UK Ltd)	11898141000033110	33682311000001103	Dapagliflozin propanediol monohydrate/ Saxagliptin hydrochloride
Dulaglutide 0.75mg/0.5ml solution for injection pre-filled disposable devices	10207841000033118	28789611000001108	Dulaglutide
Trulicity 0.75mg/0.5ml solution for injection pre-filled pens (Eli Lilly and Company Ltd)	10208041000033112	28461011000001109	Dulaglutide
Trulicity 1.5mg/0.5ml solution for injection pre-filled pens (Eli Lilly and Company Ltd)	10208141000033111	28462311000001103	Dulaglutide
Dulaglutide 1.5mg/0.5ml solution for injection pre-filled disposable devices	10207941000033114	28789711000001104	Dulaglutide
Jardiance 10mg tablets (Boehringer Ingelheim Ltd)	9337141000033114	25238811000001107	Empagliflozin
Empagliflozin 10mg tablets	9336641000033119	25290511000001101	Empagliflozin
Empagliflozin 25mg tablets	9336841000033118	25290611000001102	Empagliflozin
Jardiance 25mg tablets (Boehringer Ingelheim Ltd)	9337241000033119	25239711000001108	Empagliflozin
Empagliflozin 12.5mg / Metformin 1g tablets	10614441000033112	30318111000001109	Empagliflozin/ Metformin hydrochloride
Synjardy 12.5mg/1000mg tablets (Boehringer Ingelheim Ltd)	10614841000033110	30175711000001100	Empagliflozin/ Metformin hydrochloride
Synjardy 12.5mg/850mg tablets (Boehringer Ingelheim Ltd)	10614741000033117	30175011000001102	Empagliflozin/ Metformin hydrochloride
Empagliflozin 12.5mg / Metformin 850mg tablets	10614341000033118	30318211000001103	Empagliflozin/ Metformin hydrochloride
Empagliflozin 5mg / Metformin 1g tablets	10614241000033111	30318311000001106	Empagliflozin/ Metformin hydrochloride
Synjardy 5mg/1000mg tablets (Boehringer Ingelheim Ltd)	10614641000033114	30174111000001100	Empagliflozin/ Metformin hydrochloride
Synjardy 5mg/850mg tablets (Boehringer Ingelheim Ltd)	10614541000033113	30173411000001107	Empagliflozin/ Metformin hydrochloride
Empagliflozin 5mg / Metformin 850mg tablets	10614141000033116	30318411000001104	Empagliflozin/ Metformin hydrochloride
Ertugliflozin 15mg tablets	12881041000033118	36529411000001105	Ertugliflozin L-pyroglutamic acid
Steglatro 15mg tablets (Merck Sharp & Dohme Ltd)	12881241000033114	36515111000001107	Ertugliflozin L-pyroglutamic acid

Ertugliflozin 5mg tablets	12880941000033111	36529511000001109	Ertugliflozin L-pyroglutamic acid
Steglatro 5mg tablets (Merck Sharp & Dohme Ltd)	12881141000033119	36514811000001101	Ertugliflozin L-pyroglutamic acid
Exenatide 10micrograms/0.04ml solution for injection 2.4ml pre-filled disposable devices	4149441000033117	11494111000001102	Exenatide
Byetta 10micrograms/0.04ml solution for injection 2.4ml pre-filled pens (AstraZeneca UK Ltd)	4149641000033115	11494611000001105	Exenatide
Byetta 5micrograms/0.02ml solution for injection 1.2ml pre-filled pens (AstraZeneca UK Ltd)	4149541000033116	11494811000001109	Exenatide
Exenatide 5micrograms/0.02ml solution for injection 1.2ml pre-filled disposable devices	4149341000033111	11494211000001108	Exenatide
Exenatide 2mg powder and solvent for prolonged-release suspension for injection vials	6388241000033117	19275411000001107	Exenatide
Bydureon 2mg powder and solvent for prolonged-release suspension for injection vials (AstraZeneca UK Ltd)	6388341000033110	19274811000001108	Exenatide
Bydureon 2mg powder and solvent for prolonged-release suspension for injection pre-filled pens (AstraZeneca UK Ltd)	13427441000033112	28426011000001108	Exenatide
Exenatide 2mg powder and solvent for prolonged-release suspension for injection pre-filled disposable devices	13427341000033118	28440211000001102	Exenatide
Exenatide 2mg/0.85ml prolonged-release suspension for injection pre-filled disposable devices	13427541000033113	38082811000001104	Exenatide
Bydureon BCise 2mg/0.85ml prolonged-release suspension for injection pre-filled pens (AstraZeneca UK Ltd)	13427641000033114	38060511000001101	Exenatide
Glibenclamide 2.5mg tablets	644541000033111	325218001	Glibenclamide
Euglucon 2.5mg tablets (Aventis Pharma)	557241000033113	233411000001102	Glibenclamide
Semi-Daonil 2.5mg tablets (Sanofi)	1278041000033114	140711000001103	Glibenclamide
Diabetamide 2.5mg tablets (Ashbourne Pharmaceuticals Ltd)	462041000033119	13511000001103	Glibenclamide
Daonil 5mg tablets (Sanofi)	406841000033114	234011000001108	Glibenclamide
Diabetamide 5mg tablets (Ashbourne Pharmaceuticals Ltd)	462141000033115	287811000001101	Glibenclamide
Euglucon 5mg tablets (Sanofi)	557341000033115	322511000001103	Glibenclamide
Glibenclamide 5mg tablets	644641000033112	325219009	Glibenclamide
Glibenclamide 5mg/5ml oral solution	5968741000033115	8523711000001107	Glibenclamide
Glibenclamide 5mg/5ml oral suspension	5968841000033113	8523811000001104	Glibenclamide
Amglidia 0.6mg/ml oral suspension with 1ml oral syringe (Amring Pharmaceuticals Ltd)	13122741000033116	37337011000001106	Glibenclamide
Amglidia 0.6mg/ml oral suspension with 5ml oral syringe (Amring Pharmaceuticals Ltd)	13122841000033114	37337211000001101	Glibenclamide
Glibenclamide 600micrograms/ml oral suspension sugar free	13122541000033112	37405911000001104	Glibenclamide
Glibenclamide 6mg/ml oral suspension sugar free	13122641000033113	37406011000001107	Glibenclamide
Amglidia 6mg/ml oral suspension with 1ml oral syringe (Amring Pharmaceuticals Ltd)	13122941000033118	37337511000001103	Glibenclamide
Amglidia 6mg/ml oral suspension with 5ml oral syringe (Amring Pharmaceuticals Ltd)	13123041000033111	37337711000001108	Glibenclamide
Gliclazide 80mg tablets	646441000033117	325242002	Gliclazide
Diaglyk 80mg tablets (Ashbourne Pharmaceuticals Ltd)	1602941000033117	49111000001103	Gliclazide
Diamicron 80mg tablets (Servier Laboratories Ltd)	463541000033118	745911000001103	Gliclazide
Gliclazide 40mg/5ml oral suspension	5869241000033114	8524211000001102	Gliclazide
Gliclazide 80mg/5ml oral suspension	3953041000033113	8524311000001105	Gliclazide

Gliclazide 40mg tablets	5709741000033116	16702011000001102	Gliclazide
Zicron 40mg tablets (Bristol Laboratories Ltd)	5709841000033114	16677511000001108	Gliclazide
Laaglyda MR 60mg tablets (Consilient Health Ltd)	8298241000033118	22225011000001109	Gliclazide
Gliclazide 60mg modified-release tablets	8298141000033113	22226111000001104	Gliclazide
Vamju 60mg modified-release tablets (Advanz Pharma)	10043441000033110	28420911000001109	Gliclazide
Lamzarin 60mg modified-release tablets (Key Pharmaceuticals Ltd)	13345341000033117	38018511000001102	Gliclazide
Bilxona 60mg modified-release tablets (Accord Healthcare Ltd)	10701741000033113	37618711000001109	Gliclazide
Glydex 160mg tablets (Medreich Plc)	12999041000033113	36910511000001100	Gliclazide
Gliclazide 160mg tablets	12998941000033116	36914911000001102	Gliclazide
Diamicron 30mg MR tablets (Servier Laboratories Ltd)	2289941000033113	3661311000001107	Gliclazide
Nazdol MR 30mg tablets (Consilient Health Ltd)	4941241000033119	13456711000001106	Gliclazide
Edicil MR 30mg tablets (Teva UK Ltd)	5815341000033115	15334511000001109	Gliclazide
Vitile XL 30mg tablets (Actavis UK Ltd)	6135341000033117	18678711000001108	Gliclazide
Dacadis MR 30mg tablets (Mylan)	5378141000033117	16536211000001102	Gliclazide
Gliclazide 30mg modified-release tablets	2289441000033115	38896211000001104	Gliclazide
Lamzarin 30mg modified-release tablets (Key Pharmaceuticals Ltd)	13345241000033110	38018211000001100	Gliclazide
Zicron PR 30mg tablets (Bristol Laboratories Ltd)	11918941000033116	33766211000001100	Gliclazide
Bilxona 30mg modified-release tablets (Accord Healthcare Ltd)	10701641000033116	30982411000001107	Gliclazide
Vamju 30mg modified-release tablets (Advanz Pharma)	10043341000033116	28420711000001107	Gliclazide
Glimepiride 2mg tablets	645841000033112	325259007	Glimepiride
Amaryl 2mg tablets (Zentiva)	58841000033113	938211000001105	Glimepiride
Niddaryl 2mg tablets (Dee Pharmaceuticals Ltd)	4522141000033112	13626511000001104	Glimepiride
Niddaryl 1mg tablets (Dee Pharmaceuticals Ltd)	4522041000033113	13626011000001107	Glimepiride
Amaryl 1mg tablets (Zentiva)	59541000033116	228111000001101	Glimepiride
Glimepiride 1mg tablets	645941000033116	325261003	Glimepiride
Glimepiride 3mg tablets	646041000033114	325262005	Glimepiride
Amaryl 3mg tablets (Zentiva)	59641000033115	164511000001108	Glimepiride
Niddaryl 3mg tablets (Dee Pharmaceuticals Ltd)	4522241000033117	13626911000001106	Glimepiride
Niddaryl 4mg tablets (Dee Pharmaceuticals Ltd)	4522341000033110	13627211000001100	Glimepiride
Amaryl 4mg tablets (Zentiva)	59741000033112	259611000001101	Glimepiride
Glimepiride 4mg tablets	646141000033113	325263000	Glimepiride
Glimepiride 6mg/5ml oral suspension	8125241000033119	21366911000001103	Glimepiride
Minodiab 5mg tablets (Pfizer Ltd)	920241000033117	652411000001107	Glipizide
Glibenese 5mg tablets (Pfizer Ltd)	644741000033115	515111000001108	Glipizide
Glipizide 5mg tablets	644841000033113	325243007	Glipizide
Glipizide 2.5mg tablets	646541000033116	325248003	Glipizide
Minodiab 2.5mg tablets (Pfizer Ltd)	920141000033112	808711000001104	Glipizide
Guar gum 5g granules sachets sugar free	655141000033116	325287003	Guar gum
Guarem 5g granules sachets (Shire Pharmaceuticals Ltd)	654941000033115	4667411000001106	Guar gum

Linagliptin 5mg tablets	6444041000033117	19525211000001103	Linagliptin
Trajenta 5mg tablets (Boehringer Ingelheim Ltd)	6444141000033118	19492811000001108	Linagliptin
Jentadueto 2.5mg/1000mg tablets (Boehringer Ingelheim Ltd)	8115941000033116	21208511000001102	Linagliptin/ Metformin hydrochloride
Linagliptin 2.5mg / Metformin 1g tablets	8115741000033119	21245011000001106	Linagliptin/ Metformin hydrochloride
Linagliptin 2.5mg / Metformin 850mg tablets	8115841000033112	21245111000001107	Linagliptin/ Metformin hydrochloride
Jentadueto 2.5mg/850mg tablets (Boehringer Ingelheim Ltd)	8116041000033114	21208211000001100	Linagliptin/ Metformin hydrochloride
Empagliflozin 10mg / Linagliptin 5mg tablets	13116741000033112	37280311000001109	Empagliflozin/ Linagliptin
Glyxambi 10mg/5mg tablets (Boehringer Ingelheim Ltd)	13116941000033110	37225211000001100	Empagliflozin/ Linagliptin
Glyxambi 25mg/5mg tablets (Boehringer Ingelheim Ltd)	13117041000033111	37225511000001102	Empagliflozin/ Linagliptin
Empagliflozin 25mg / Linagliptin 5mg tablets	13116841000033119	37280511000001103	Empagliflozin/ Linagliptin
Victoza 6mg/ml solution for injection 3ml pre-filled pens (Novo Nordisk Ltd)	5131341000033117	15858611000001101	Liraglutide
Liraglutide 6mg/ml solution for injection 3ml pre-filled disposable devices	5131241000033110	15859111000001102	Liraglutide
Saxenda 6mg/ml solution for injection 3ml pre-filled pens (Novo Nordisk Ltd)	11919041000033113	33747711000001102	Liraglutide
Lixisenatide 10micrograms/0.2ml solution for injection 3ml pre-filled disposable devices	8267341000033110	21994611000001106	Lixisenatide
Lyxumia 10micrograms/0.2ml solution for injection 3ml pre-filled pens (Sanofi)	8267541000033115	21941511000001102	Lixisenatide
Lixisenatide 20micrograms/0.2ml solution for injection 3ml pre-filled disposable devices	8267441000033116	21994811000001105	Lixisenatide
Lyxumia 20micrograms/0.2ml solution for injection 3ml pre-filled pens (Sanofi)	8267641000033119	21941011000001105	Lixisenatide
Metformin 500mg tablets	896941000033112	325278007	Metformin hydrochloride
Glucophage 500mg tablets (Merck Serono Ltd)	644941000033117	363211000001102	Metformin hydrochloride
Glucophage 850mg tablets (Merck Serono Ltd)	645041000033117	365111000001109	Metformin hydrochloride
Metformin 850mg tablets	897041000033113	325279004	Metformin hydrochloride
Rosiglitazone 1mg / Metformin 500mg tablets	3191341000033115	409121008	Metformin hydrochloride/ Rosiglitazone maleate
Avandamet 1mg/500mg tablets (GlaxoSmithKline UK Ltd)	2995141000033110	5302911000001102	Metformin hydrochloride/ Rosiglitazone maleate
Avandamet 2mg/500mg tablets (GlaxoSmithKline UK Ltd)	2995241000033115	5303611000001103	Metformin hydrochloride/ Rosiglitazone maleate
Rosiglitazone 2mg / Metformin 500mg tablets	3191441000033114	409122001	Metformin hydrochloride/ Rosiglitazone maleate
Rosiglitazone 2mg / Metformin 1g tablets	3200541000033116	409124000	Metformin hydrochloride/

			Rosiglitazone maleate
Avandamet 2mg/1000mg tablets (GlaxoSmithKline UK Ltd)	3200741000033112	8176311000001105	Metformin hydrochloride/ Rosiglitazone maleate
Avandamet 4mg/1000mg tablets (GlaxoSmithKline UK Ltd)	3200841000033119	8174611000001109	Metformin hydrochloride/ Rosiglitazone maleate
Rosiglitazone 4mg / Metformin 1g tablets	3200641000033115	409125004	Metformin hydrochloride/ Rosiglitazone maleate
Metformin 500mg/5ml oral solution	5997041000033115	409197000	Metformin hydrochloride
Pioglitazone 15mg / Metformin 850mg tablets	3983941000033115	419873003	Metformin hydrochloride/ Pioglitazone hydrochloride
Competact 15mg/850mg tablets (Takeda UK Ltd)	3984241000033114	10922311000001108	Metformin hydrochloride/ Pioglitazone hydrochloride
Metformin 500mg/5ml oral suspension	2620241000033119	8664411000001102	Metformin hydrochloride
Metformin 500mg/5ml oral solution sugar free	3982341000033110	10750111000001100	Metformin hydrochloride
Metsol 500mg/5ml oral solution (Kappin Ltd)	3982441000033116	10741611000001100	Metformin hydrochloride
Vildagliptin 50mg / Metformin 1g tablets	4452441000033113	13413011000001108	Metformin hydrochloride/ Vildagliptin
Eucreas 50mg/1000mg tablets (Novartis Pharmaceuticals UK Ltd)	4452541000033114	13412311000001101	Metformin hydrochloride/ Vildagliptin
Eucreas 50mg/850mg tablets (Novartis Pharmaceuticals UK Ltd)	4452641000033110	13412611000001106	Metformin hydrochloride/ Vildagliptin
Vildagliptin 50mg / Metformin 850mg tablets	4452341000033119	13413111000001109	Metformin hydrochloride/ Vildagliptin
Metformin 1g oral powder sachets sugar free	5007741000033111	15411211000001106	Metformin hydrochloride
Glucophage 1000mg oral powder sachets (Merck Serono Ltd)	5007941000033114	15374311000001101	Metformin hydrochloride
Metformin 500mg oral powder sachets sugar free	5007641000033119	15411311000001103	Metformin hydrochloride
Glucophage 500mg oral powder sachets (Merck Serono Ltd)	5007841000033118	15373711000001104	Metformin hydrochloride
Janumet 50mg/1000mg tablets (Merck Sharp & Dohme Ltd)	5132241000033116	17060511000001108	Metformin hydrochloride/ Sitagliptin phosphate
Metformin 1g / Sitagliptin 50mg tablets	5576041000033115	17071811000001108	Metformin hydrochloride/ Sitagliptin phosphate
Saxagliptin 2.5mg / Metformin 1g tablets	8242641000033113	21711411000001100	Metformin hydrochloride/ Saxagliptin hydrochloride

Komboglyze 2.5mg/1000mg tablets (AstraZeneca UK Ltd)	8242841000033114	21705611000001102	Metformin hydrochloride/ Saxagliptin hydrochloride
Komboglyze 2.5mg/850mg tablets (AstraZeneca UK Ltd)	8242741000033116	21705311000001107	Metformin hydrochloride/ Saxagliptin hydrochloride
Saxagliptin 2.5mg / Metformin 850mg tablets	8242541000033112	21711511000001101	Metformin hydrochloride/ Saxagliptin hydrochloride
Metformin 1g/5ml oral solution sugar free	11781341000033117	33550811000001105	Metformin hydrochloride
Metformin 850mg/5ml oral solution sugar free	11781441000033111	33550911000001100	Metformin hydrochloride
Bolamyn SR 1000mg tablets (Teva UK Ltd)	8348941000033113	22308811000001109	Metformin hydrochloride
Metabet SR 1000mg tablets (Morningside Healthcare Ltd)	6279741000033117	18885611000001108	Metformin hydrochloride
Glucophage SR 1000mg tablets (Merck Serono Ltd)	4945341000033110	15367811000001105	Metformin hydrochloride
Metformin 1g modified-release tablets	4945241000033117	38893711000001104	Metformin hydrochloride
Yaltormin SR 1000mg tablets (Wockhardt UK Ltd)	12593341000033117	35548311000001108	Metformin hydrochloride
Meijumet 1000mg modified-release tablets (Medreich Plc)	12326741000033119	34553311000001105	Metformin hydrochloride
Sukkarto SR 1000mg tablets (Morningside Healthcare Ltd)	9230841000033114	24568211000001104	Metformin hydrochloride
Glucient SR 1000mg tablets (Consilient Health Ltd)	10598941000033117	30012311000001107	Metformin hydrochloride
Glucophage SR 750mg tablets (Merck Serono Ltd)	4549241000033113	13748611000001107	Metformin hydrochloride
Glucient SR 750mg tablets (Consilient Health Ltd)	10598841000033113	30012111000001105	Metformin hydrochloride
Meijumet 750mg modified-release tablets (Medreich Plc)	12326641000033111	34552811000001109	Metformin hydrochloride
Yaltormin SR 750mg tablets (Wockhardt UK Ltd)	12593241000033110	35548011000001105	Metformin hydrochloride
Metformin 750mg modified-release tablets	4549141000033118	38893811000001107	Metformin hydrochloride
Sukkarto SR 750mg tablets (Morningside Healthcare Ltd)	13429041000033112	38238111000001109	Metformin hydrochloride
Glucophage SR 500mg tablets (Merck Serono Ltd)	3228241000033112	8990711000001100	Metformin hydrochloride
Bolamyn SR 500mg tablets (Teva UK Ltd)	4957741000033118	14183911000001108	Metformin hydrochloride
Metabet SR 500mg tablets (Morningside Healthcare Ltd)	6029841000033115	18141911000001101	Metformin hydrochloride
Glucient SR 500mg tablets (Consilient Health Ltd)	6391041000033117	19308111000001109	Metformin hydrochloride
Diagemet XL 500mg tablets (Genus Pharmaceuticals Ltd)	7874941000033110	20552511000001105	Metformin hydrochloride
Metformin 500mg modified-release tablets	3228141000033117	39113511000001101	Metformin hydrochloride
Glucorex SR 500mg tablets (GlucoRx Ltd)	13606641000033119	38749111000001108	Metformin hydrochloride
Sukkarto SR 500mg tablets (Morningside Healthcare Ltd)	9230641000033113	24568411000001100	Metformin hydrochloride
Yaltormin SR 500mg tablets (Wockhardt UK Ltd)	12593141000033115	35547511000001101	Metformin hydrochloride

Meijumet 500mg modified-release tablets (Medreich Plc)	12326541000033110	34552411000001107	Metformin hydrochloride
Metuxtan SR 500mg tablets (Accord Healthcare Ltd)	12664441000033115	35849011000001104	Metformin hydrochloride
Starlix 180mg tablets (Novartis Pharmaceuticals UK Ltd)	2288741000033118	3883511000001107	Nateglinide
Nateglinide 180mg tablets	2288441000033113	134608004	Nateglinide
Nateglinide 120mg tablets	2288341000033119	134609007	Nateglinide
Starlix 120mg tablets (Novartis Pharmaceuticals UK Ltd)	2288641000033110	3651211000001105	Nateglinide
Starlix 60mg tablets (Novartis Pharmaceuticals UK Ltd)	2288541000033114	3650611000001102	Nateglinide
Nateglinide 60mg tablets	2288241000033112	134610002	Nateglinide
Actos 30mg tablets (Takeda UK Ltd)	2191041000033110	446711000001101	Pioglitazone hydrochloride
Pioglitazone 30mg tablets	2190841000033113	326061000	Pioglitazone hydrochloride
Glizofar 30mg tablets (Arrow Generics Ltd)	6526041000033117	20023111000001108	Pioglitazone hydrochloride
Diabiom 30mg tablets (Tillomed Laboratories Ltd)	10337041000033113	29743011000001108	Pioglitazone hydrochloride
Glidipion 30mg tablets (Actavis UK Ltd)	8839341000033116	23369611000001106	Pioglitazone hydrochloride
Glizofar 15mg tablets (Arrow Generics Ltd)	6525941000033110	20022911000001104	Pioglitazone hydrochloride
Pioglitazone 15mg tablets	2190741000033115	326062007	Pioglitazone hydrochloride
Actos 15mg tablets (Takeda UK Ltd)	2190941000033117	58011000001106	Pioglitazone hydrochloride
Diabiom 15mg tablets (Tillomed Laboratories Ltd)	10336941000033112	29742811000001105	Pioglitazone hydrochloride
Glidipion 15mg tablets (Actavis UK Ltd)	10251341000033112	28940811000001104	Pioglitazone hydrochloride
Actos 45mg tablets (Takeda UK Ltd)	2996041000033119	5199411000001105	Pioglitazone hydrochloride
Pioglitazone 45mg tablets	2995941000033112	374897009	Pioglitazone hydrochloride
Glizofar 45mg tablets (Arrow Generics Ltd)	6526141000033118	20023411000001103	Pioglitazone hydrochloride
Glidipion 45mg tablets (Actavis UK Ltd)	8839441000033110	23372211000001107	Pioglitazone hydrochloride
Diabiom 45mg tablets (Tillomed Laboratories Ltd)	10337141000033112	29743211000001103	Pioglitazone hydrochloride
Repaglinide 500microgram tablets	1672941000033110	326047005	Repaglinide
NovoNorm 500microgram tablets (Novo Nordisk Ltd)	1659941000033116	152311000001101	Repaglinide
Prandin 0.5mg tablets (Novo Nordisk Ltd)	3993941000033119	10952411000001100	Repaglinide
Enyglid 0.5mg tablets (Consilient Health Ltd)	6391141000033118	19306711000001106	Repaglinide
Enyglid 1mg tablets (Consilient Health Ltd)	6391241000033113	19306911000001108	Repaglinide
Prandin 1mg tablets (Novo Nordisk Ltd)	3994041000033117	10956911000001107	Repaglinide
NovoNorm 1mg tablets (Novo Nordisk Ltd)	1660041000033118	494111000001103	Repaglinide
Repaglinide 1mg tablets	1672741000033112	326048000	Repaglinide
Repaglinide 2mg tablets	1672841000033119	326049008	Repaglinide
NovoNorm 2mg tablets (Novo Nordisk Ltd)	1660141000033119	840811000001100	Repaglinide
Prandin 2mg tablets (Novo Nordisk Ltd)	3994141000033118	10957811000001100	Repaglinide
Enyglid 2mg tablets (Consilient Health Ltd)	6391341000033115	19307311000001105	Repaglinide

Saxagliptin 2.5mg tablets	6123241000033112	443067000	Saxagliptin hydrochloride
Onglyza 2.5mg tablets (AstraZeneca UK Ltd)	6123341000033119	18596311000001108	Saxagliptin hydrochloride
Saxagliptin 5mg tablets	5316641000033118	443713000	Saxagliptin hydrochloride
Onglyza 5mg tablets (AstraZeneca UK Ltd)	5316741000033110	15993011000001101	Saxagliptin hydrochloride
Ozempic 0.25mg/0.19ml solution for injection 1.5ml pre-filled pens (Novo Nordisk Ltd)	12876541000033113	36470811000001107	Semaglutide
Semaglutide 0.25mg/0.19ml solution for injection 1.5ml pre-filled disposable device	12876241000033111	36490911000001109	Semaglutide
Semaglutide 0.5mg/0.37ml solution for injection 1.5ml pre-filled disposable device	12876341000033118	36491011000001101	Semaglutide
Ozempic 0.5mg/0.37ml solution for injection 1.5ml pre-filled pens (Novo Nordisk Ltd)	12876641000033114	36471111000001106	Semaglutide
Ozempic 1mg/0.74ml solution for injection 3ml pre-filled pens (Novo Nordisk Ltd)	12876441000033112	36471411000001101	Semaglutide
Semaglutide 1mg/0.74ml solution for injection 3ml pre-filled disposable device	12876141000033116	36491111000001100	Semaglutide
Rybelsus 14mg tablets (Novo Nordisk Ltd)	13712741000033116	38732411000001108	Semaglutide
Semaglutide 14mg tablets	13712441000033111	38840111000001100	Semaglutide
Semaglutide 3mg tablets	13712241000033110	38840211000001106	Semaglutide
Rybelsus 3mg tablets (Novo Nordisk Ltd)	13712541000033112	38731811000001101	Semaglutide
Rybelsus 7mg tablets (Novo Nordisk Ltd)	13712641000033113	38732111000001103	Semaglutide
Semaglutide 7mg tablets	13712341000033117	38840311000001103	Semaglutide
Sitagliptin 25mg tablets	7687441000033111	423962004	Sitagliptin phosphate
Januvia 25mg tablets (Merck Sharp & Dohme Ltd)	7687541000033112	20115111000001106	Sitagliptin phosphate
Sitagliptin 100mg tablets	4133241000033117	424345005	Sitagliptin phosphate
Januvia 100mg tablets (Merck Sharp & Dohme Ltd)	4133341000033110	11473711000001108	Sitagliptin phosphate
Januvia 50mg tablets (Merck Sharp & Dohme Ltd)	7687341000033117	20114811000001100	Sitagliptin phosphate
Sitagliptin 50mg tablets	7687241000033110	424513004	Sitagliptin phosphate
Tolbutamide 500mg tablets	1450941000033113	325267004	Tolbutamide

Table S12. Hypertension Aurum codes

Term	Medcode ID	SNOMED CT Concept ID	SNOMED CT Description ID
HTN - Hypertension	3117511000006117	38341003	2164904016
Accelerated essential hypertension	3784371000006115	78975002	503982017
High blood pressure	3117411000006118	38341003	64172013
Malignant hypertensive heart disease NOS	299650019	54225002	90135019
History of hypertension	4540441000006117	161501007	2987063016
Benign hypertensive heart disease NOS	299654011	36221001	60444016
Secondary malignant hypertension NOS	299677011	89242004	147988014
Other specified hypertensive disease	299686018	38341003	1215744012
[X]Hypertension secondary to other renal disorders	300871017	31992008	53452019
Benign hypertensive renal disease	1409014	193003	1409014
HHD - Hypertensive heart disease	3552531000006110	64715009	499930018
Hypertensive disease	64168014	38341003	64168014
Hypertensive renal disease	64282015	38481006	64282015
Malignant hypertensive heart disease	90135019	54225002	90135019
Systolic hypertension	93494011	56218007	93494011
Primary hypertension	99047018	59621000	99047018
Hypertensive heart disease NOS	741661000006118	64715009	107545013
Hypertensive heart disease NOS with CCF	741681000006111	64715009	107545013
Hypertensive heart disease NOS without CCF	741691000006114	64715009	107545013
Malignant hypertension	884121000006111	78975002	884121000006111
Renal hypertension	47076011	38481006	64282015
Hypertensive crisis	84112010	50490005	84111015
Hypertensive heart disease	107545013	64715009	107545013
HT - Hypertension	3117481000006113	38341003	490281011
Stage 2 hypertension	8286581000006114	846371000000103	2211211000000110
Benign hypertensive heart disease without CCF	504911000006115	77970009	129404019
Hypertensive heart disease NOS	299655012	64715009	107545013
Hypertensive heart and renal disease NOS	299675015	86234004	143003017
Hypertensive renal failure	3296351000006114	49220004	81984019
Cardiomegaly - hypertensive	411508017	275516004	411508017
Severe hypertension (Nat Inst for Health Clinical Ex 2011)	1846961000006115	843841000000109	2189451000000110
Stage 2 hypertension (NICE - Nat Ins for Hth Clin Excl 2011)	1846991000006111	846371000000103	2194941000000119
Stage 1 hyperten (NICE 2011) with evidnce end organ damge	1908721000006111	908651000000101	2335801000000114
Systemic arterial hypertension	3117461000006115	38341003	490278018
High blood pressure	64172013	24184005	196353013
Secondary malignant hypertension	151161000006115	89242004	147988014
Benign hypertensive heart disease without congestive heart failure	3768511000006114	77970009	129404019
Malignant hypertension	3642801000006112	70272006	116721012

Hypertensive cardiopathy	3552501000006119	64715009	107547017
Hypertensive cardiomegaly	3552511000006116	64715009	107548010
Hypertensive heart&renal dis wth (congestive) heart failure	741701000006114	194779001	299672017
Hyperten heart&renal dis+both(congestv)heart and renal fail	789941000006117	194781004	299674016
Hypertensive heart and renal disease with renal failure	299673010	194780003	299673010
[X]Hypertensive diseases	300869017	38341003	1215744012
Hypertensive renal disease with renal failure	299665018	49220004	81984019
Systemic primary arterial hypertension	3468501000006117	59621000	99046010
Benign hypertensive heart disease with CCF	504901000006118	194767001	299653017
Stage 2 hypertension	1806081000006115	1806081000006104	1806081000006115
HBP - High blood pressure	3117471000006110	38341003	490280012
Accelerated secondary hypertension	3950631000006119	89242004	508416019
Hypertensive cardiovascular disease	3552521000006112	64715009	107549019
Benign essential hypertension	3135013	1201005	3135013
[RFC] Hypertension	909441000006118	909441000006102	909441000006118
Essential hypertension NOS	395751018	59621000	99042012
Secondary malignant renovascular hypertension	299676019	194783001	299676019
Essential hypertension	99042012	59621000	99042012
Severe hypertension	2193021000000110	843841000000109	2193021000000110
Hypertension resistant to drug therapy	2193971000000110	845891000000103	2193971000000110
Malignant hypertensive renal disease	108730018	65443008	108730018
Malignant hypertensive heart AND renal disease	110659019	66610008	110659019
H/O: hypertension	251674014	161501007	251674014
[X]Other secondary hypertension	300870016	31992008	53452019
Hypertension confirmed	1823901000006112	1823901000006108	1823901000006112
BP+ - Hypertension	3117501000006115	38341003	490283014
Hypertensive vascular disease	3117421000006114	38341003	64173015
Benign hypertensive heart AND renal disease	109700019	66052004	109700019
Malignant essential hypertension	131046010	78975002	131046010
Hypertensive heart AND renal disease	143003017	86234004	143003017
Hypertensive disorder, systemic arterial	3117521000006113	38341003	2671386015
BP - High blood pressure	3117451000006117	38341003	490277011
Resistant hypertension	8286321000006117	845891000000103	2194001000000116
Severe hypertension	1806141000006113	1806141000006109	1806141000006113
Hypertensive disease NOS	299687010	38341003	1215744012
Hypertension NOS	790121000006116	38341003	64176011
BP - hypertensive disease	523801000006119	38341003	64168014
Hypertensive nephropathy	3119661000006114	38481006	64285018
Hypertensive renal disease NOS	395753015	38481006	64282015
Hypertension due to renovascular disease	4356081000006115	123799005	1220673019
Malignant hypertensive heart disease with CCF	728671000006119	83105008	1236017010

Malignant hypertensive heart disease without CCF	728681000006116	36315003	60617018
Hypertensive renal + heart dis	884131000006114	86234004	884131000006114
Hypertensive retinopathy	12496011	6962006	12496011
Malignant hypertensive heart disease with congestive heart failure	3852501000006115	83105008	137848017
Benign hypertensive heart disease	60444016	36221001	60444016
Idiopathic hypertension	3468491000006113	59621000	99044013

Table S13. Anti-Hypertension Aurum product codes

Term from EMIS	Prod code ID	dmd ID	Drug substance name
Rasilez 300mg tablets (Noden Pharma DAC)	4213941000033117	11961711000001103	Aliskiren hemifumarate
Aliskiren 300mg tablets	4213741000033115	425669009	Aliskiren hemifumarate
Rasilez 150mg tablets (Noden Pharma DAC)	4213841000033113	11960911000001108	Aliskiren hemifumarate
Aliskiren 150mg tablets	4213641000033112	425960005	Aliskiren hemifumarate
Amoride 5mg tablets (Dr Reddy's Laboratories (UK) Ltd)	2956041000033112	220711000001102	Amiloride hydrochloride
Amiloride 5mg tablets	57141000033118	318052005	Amiloride hydrochloride
Amiloride 2.5mg / Cyclopenthiazide 250microgram tablets	1903541000033116	318096005	Amiloride hydrochloride/ Cyclopenthiazide
Navispare 2.5mg/250microgram tablets (Advanz Pharma)	956741000033114	535711000001100	Amiloride hydrochloride/ Cyclopenthiazide
Burinex A 5mg/1mg tablets (LEO Pharma)	172341000033112	33911000001104	Amiloride hydrochloride/ Bumetanide
Amiloride 5mg / Bumetanide 1mg tablets	1845641000033119	318097001	Amiloride hydrochloride/ Bumetanide
Co-amilozide 2.5mg/25mg tablets	376941000033117	318121006	Amiloride hydrochloride/ Hydrochlorothiazide
Moduret 25 tablets (Merck Sharp & Dohme Ltd)	935441000033112	314211000001100	Amiloride hydrochloride/ Hydrochlorothiazide
Synuretic 25 tablets (Dr Reddy's Laboratories (UK) Ltd)	2955841000033110	465111000001101	Amiloride hydrochloride/ Hydrochlorothiazide
Frumil LS 20mg/2.5mg tablets (Sanofi)	614341000033113	550711000001106	Amiloride hydrochloride/ Furosemide
Co-amilofruse 2.5mg/20mg tablets	376841000033113	318135008	Amiloride hydrochloride/ Furosemide
Co-amilofruse 5mg/40mg tablets	376641000033112	318136009	Amiloride hydrochloride/ Furosemide
Frumil 40mg/5mg tablets (Sanofi)	614641000033117	427411000001106	Amiloride hydrochloride/ Furosemide
Froop Co 5mg/40mg tablets (Ashbourne Pharmaceuticals Ltd)	1611441000033117	331311000001103	Amiloride hydrochloride/ Furosemide
Lasoride 5mg/40mg tablets (Sanofi)	818941000033112	678511000001106	Amiloride hydrochloride/ Furosemide
Fru-Co 5mg/40mg tablets (Teva UK Ltd)	615241000033116	818511000001106	Amiloride hydrochloride/ Furosemide
Frumil Forte 10mg/80mg tablets (Sanofi)	615641000033118	82611000001106	Amiloride hydrochloride/ Furosemide

Co-amilofruse 10mg/80mg tablets	376741000033115	318137000	Amiloride hydrochloride/ Furosemide
Moducren tablets (Merck Sharp & Dohme Ltd)	935341000033118	74311000001101	Amiloride hydrochloride/ Hydrochlorothiazide/ Timolol maleate
Kalten capsules (M & A Pharmachem Ltd)	793741000033117	237011000001100	Amiloride hydrochloride/ Atenolol/ Hydrochlorothiazide
Co-amilozide 5mg/50mg tablets	369141000033118	377566005	Amiloride hydrochloride/ Hydrochlorothiazide
Amilmaxco 5mg/50mg tablets (Ashbourne Pharmaceuticals Ltd)	57341000033115	712211000001108	Amiloride hydrochloride/ Hydrochlorothiazide
Moduretic 5mg/50mg tablets (Merck Sharp & Dohme Ltd)	935541000033113	453811000001103	Amiloride hydrochloride/ Hydrochlorothiazide
Amil-Co 5mg/50mg tablets (IVAX Pharmaceuticals UK Ltd)	60341000033110	636611000001107	Amiloride hydrochloride/ Hydrochlorothiazide
Synuretic 50 tablets (Dr Reddy's Laboratories (UK) Ltd)	2955741000033117	601011000001103	Amiloride hydrochloride/ Hydrochlorothiazide
Co-amilofruse 5mg/40mg/5ml oral suspension	5710441000033117	8427011000001108	Amiloride hydrochloride/ Furosemide
Co-amilozide 5mg/50mg/5ml oral solution	351941000033111	8792311000001109	Amiloride hydrochloride/ Hydrochlorothiazide
Timolol 10mg / Amiloride 2.5mg / Hydrochlorothiazide 25mg tablets (Essential Generics Ltd)	5376441000033114	16458411000001107	Amiloride hydrochloride/ Hydrochlorothiazide/ Timolol maleate
Amilamont 5mg/5ml oral solution sugar free (Rosemont Pharmaceuticals Ltd)	2101641000033119	799711000001108	Amiloride hydrochloride
Amiloride 5mg/5ml oral solution sugar free	53841000033117	35900111000001108	Amiloride hydrochloride
Amlodipine 5mg tablets	3038041000033111	319283006	Amlodipine
Istin 5mg tablets (Upjohn UK Ltd)	787941000033112	172711000001100	Amlodipine
Amlostin 5mg tablets (Discovery Pharmaceuticals)	3188741000033111	8046211000001107	Amlodipine
Amlostin 10mg tablets (Discovery Pharmaceuticals)	3188841000033118	8046411000001106	Amlodipine
Istin 10mg tablets (Upjohn UK Ltd)	788041000033110	408111000001107	Amlodipine
Amlodipine 10mg tablets	3038141000033110	319284000	Amlodipine
Sevikar 20mg/5mg tablets (Daiichi Sankyo UK Ltd)	5006141000033110	15773211000001105	Amlodipine besilate/ Olmesartan medoxomil
Olmesartan medoxomil 20mg / Amlodipine 5mg tablets	5005841000033114	429502004	Amlodipine besilate/ Olmesartan medoxomil

Olmesartan medoxomil 40mg / Amlodipine 5mg tablets	5005941000033118	429503009	Amlodipine besilate/ Olmesartan medoxomil
Sevikar 40mg/5mg tablets (Daiichi Sankyo UK Ltd)	5006241000033115	15772911000001108	Amlodipine besilate/ Olmesartan medoxomil
Sevikar 40mg/10mg tablets (Daiichi Sankyo UK Ltd)	5006341000033113	15772611000001102	Amlodipine besilate/ Olmesartan medoxomil
Olmesartan medoxomil 40mg / Amlodipine 10mg tablets	5006041000033111	429678006	Amlodipine besilate/ Olmesartan medoxomil
Amlodipine 2.5mg tablets	13422241000033114	429828006	Amlodipine
Amlodipine 10mg/5ml oral suspension	3963741000033115	8278111000001105	Amlodipine
Amlodipine 5mg/5ml oral suspension	5888741000033110	8278311000001107	Amlodipine
Amlodipine 10mg / Valsartan 160mg tablets	4021641000033111	11160111000001107	Amlodipine besilate/ Valsartan
Exforge 10mg/160mg tablets (Novartis Pharmaceuticals UK Ltd)	4021941000033116	11160711000001108	Amlodipine besilate/ Valsartan
Exforge 5mg/160mg tablets (Novartis Pharmaceuticals UK Ltd)	4021841000033112	11161511000001105	Amlodipine besilate/ Valsartan
Amlodipine 5mg / Valsartan 160mg tablets	4021541000033110	11160211000001101	Amlodipine besilate/ Valsartan
Amlodipine 5mg / Valsartan 80mg tablets	4021441000033114	11160311000001109	Amlodipine besilate/ Valsartan
Exforge 5mg/80mg tablets (Novartis Pharmaceuticals UK Ltd)	4021741000033119	11161811000001108	Amlodipine besilate/ Valsartan
Amlodipine 5mg/5ml oral solution	5490241000033113	13892511000001100	Amlodipine
Amlodipine 1.5mg/5ml oral suspension	4897741000033118	15773511000001108	Amlodipine
Sevikar HCT 20mg/5mg/12.5mg tablets (Daiichi Sankyo UK Ltd)	6180641000033112	18986411000001108	Amlodipine besilate/ Hydrochlorothiazide/ Olmesartan medoxomil
Sevikar HCT 40mg/10mg/12.5mg tablets (Daiichi Sankyo UK Ltd)	6180841000033113	18987011000001101	Amlodipine besilate/ Hydrochlorothiazide/ Olmesartan medoxomil
Sevikar HCT 40mg/10mg/25mg tablets (Daiichi Sankyo UK Ltd)	6181041000033110	18987611000001108	Amlodipine besilate/ Hydrochlorothiazide/ Olmesartan medoxomil
Sevikar HCT 40mg/5mg/12.5mg tablets (Daiichi Sankyo UK Ltd)	6180741000033115	18986711000001102	Amlodipine besilate/ Hydrochlorothiazide/ Olmesartan medoxomil
Sevikar HCT 40mg/5mg/25mg tablets (Daiichi Sankyo UK Ltd)	6180941000033117	18987311000001103	Amlodipine besilate/ Hydrochlorothiazide/ Olmesartan medoxomil
Amlodipine 10mg/5ml oral solution	7740641000033112	20478011000001105	Amlodipine
Perindopril erbumine 4mg / Amlodipine 10mg tablets	9105341000033116	23984911000001108	Amlodipine besilate/

			Perindopril erbumine
Perindopril erbumine 4mg / Amlodipine 5mg tablets	9105241000033114	23985011000001108	Amlodipine besilate/ Perindopril erbumine
Perindopril erbumine 8mg / Amlodipine 10mg tablets	9105541000033111	23985111000001109	Amlodipine besilate/ Perindopril erbumine
Perindopril erbumine 8mg / Amlodipine 5mg tablets	9105441000033110	23985211000001103	Amlodipine besilate/ Perindopril erbumine
Amlodipine 10mg/5ml oral solution sugar free	10387441000033111	29826211000001109	Amlodipine
Amlodipine 5mg/5ml oral solution sugar free	4508041000033119	29826311000001101	Amlodipine
Amlodipine 5mg/5ml oral suspension sugar free	12682241000033110	36237311000001101	Amlodipine
Edarbi 40mg tablets (Takeda UK Ltd)	7755241000033119	20351211000001103	Azilsartan medoxomil
Azilsartan medoxomil 40mg tablets	7754941000033110	449109006	Azilsartan medoxomil
Azilsartan medoxomil 80mg tablets	7755041000033110	449333009	Azilsartan medoxomil
Edarbi 80mg tablets (Takeda UK Ltd)	7755341000033112	20351811000001102	Azilsartan medoxomil
Azilsartan medoxomil 20mg tablets	7754841000033119	20418711000001104	Azilsartan medoxomil
Edarbi 20mg tablets (Takeda UK Ltd)	7755141000033114	20350911000001100	Azilsartan medoxomil
Bendroflumethiazide 2.5mg tablets	3083141000033112	317919004	Bendroflumethiazide
Urizide 2.5mg tablets (Dr Reddy's Laboratories (UK) Ltd)	2955941000033119	485311000001101	Bendroflumethiazide
Aprinox 2.5mg tablets (Advanz Pharma)	74541000033119	672111000001100	Bendroflumethiazide
Neo-Naclex 2.5mg tablets (Advanz Pharma)	6045641000033115	18149011000001101	Bendroflumethiazide
Urizide 5mg tablets (Dr Reddy's Laboratories (UK) Ltd)	1495441000033116	898211000001109	Bendroflumethiazide
Neo-Naclex 5mg tablets (Mercury Pharma Group Ltd)	965341000033114	817911000001100	Bendroflumethiazide
Aprinox 5mg tablets (Amdipharm Plc)	74641000033118	120911000001103	Bendroflumethiazide
Bendroflumethiazide 5mg tablets	3083241000033117	317920005	Bendroflumethiazide
Corgaretic 40mg tablets (Sanofi-Synthelabo Ltd)	375441000033118	3886211000001102	Bendroflumethiazide/ Nadolol
Corgaretic 80mg tablets (Sanofi-Synthelabo Ltd)	375541000033117	4057911000001102	Bendroflumethiazide/ Nadolol
Prestim tablets (Meda Pharmaceuticals Ltd)	1132741000033111	98411000001109	Bendroflumethiazide/ Timolol maleate
Timolol 10mg / Bendroflumethiazide 2.5mg tablets	3346641000033118	318556002	Bendroflumethiazide/ Timolol maleate
Tenben 25mg/1.25mg capsules (Galen Ltd)	1414241000033117	721411000001106	Atenolol/ Bendroflumethiazide
Inderetic 80mg/2.5mg capsules (AstraZeneca UK Ltd)	756641000033114	333111000001104	Bendroflumethiazide/ Propranolol hydrochloride

Bendroflumethiazide 5mg/5ml oral suspension	5401741000033110	8306711000001109	Bendroflumethiazide
Bendroflumethiazide 2.5mg/5ml oral suspension	3151841000033117	8306811000001101	Bendroflumethiazide
Bendroflumethiazide 1.25mg/5ml oral suspension	5453941000033119	8307011000001105	Bendroflumethiazide
Centyl K modified-release tablets (LEO Pharma)	234841000033110	3932711000001105	Bendroflumethiazide/ Potassium chloride
Bendroflumethiazide 2.5mg / Potassium chloride 573mg (potassium 7.7mmol) modified-release tablets	3161041000033115	35910011000001105	Bendroflumethiazide/ Potassium chloride
Neo-Naclex-K modified-release tablets (Mercury Pharma Group Ltd)	967041000033118	3638211000001100	Bendroflumethiazide/ Potassium chloride
Bendroflumethiazide 2.5mg / Potassium chloride 630mg (potassium 8.4mmol) modified-release tablets	3162841000033118	35910111000001106	Bendroflumethiazide/ Potassium chloride
Inderex 160mg/5mg modified-release capsules (AstraZeneca UK Ltd)	756741000033117	350811000001100	Bendroflumethiazide/ Propranolol hydrochloride
Amias 2mg tablets (Takeda UK Ltd)	59241000033118	97311000001103	Candesartan cilexetil
Candesartan 2mg tablets	211641000033118	318977009	Candesartan cilexetil
Candesartan 4mg tablets	211741000033110	318978004	Candesartan cilexetil
Amias 4mg tablets (Takeda UK Ltd)	59341000033111	857411000001100	Candesartan cilexetil
Amias 8mg tablets (Takeda UK Ltd)	59441000033117	36011000001106	Candesartan cilexetil
Candesartan 8mg tablets	211841000033117	318979007	Candesartan cilexetil
Candesartan 16mg tablets	211541000033119	318980005	Candesartan cilexetil
Amias 16mg tablets (Takeda UK Ltd)	59141000033113	908511000001100	Candesartan cilexetil
Candesartan 32mg tablets	3227241000033111	376998003	Candesartan cilexetil
Amias 32mg tablets (Takeda UK Ltd)	3227341000033118	8983911000001107	Candesartan cilexetil
Capozide LS 12.5mg/25mg tablets (Bristol-Myers Squibb Pharmaceuticals Ltd)	209241000033119	546711000001107	Captopril/ Hydrochlorothiazide
Co-zidocapt 12.5mg/25mg tablets	1893141000033119	318806002	Captopril/ Hydrochlorothiazide
Co-zidocapt 25mg/50mg tablets	1893241000033114	318807006	Captopril/ Hydrochlorothiazide
Acezide 25mg/50mg tablets (Bristol-Myers Squibb Pharmaceuticals Ltd)	13141000033119	263311000001101	Captopril/ Hydrochlorothiazide
Capozide 25mg/50mg tablets (Bristol-Myers Squibb Pharmaceuticals Ltd)	216441000033110	17311000001102	Captopril/ Hydrochlorothiazide
Capoten 12.5mg tablets (Bristol-Myers Squibb Pharmaceuticals Ltd)	214141000033113	134511000001103	Captopril
Ecopace 12.5mg tablets (Advanz Pharma)	2945841000033112	221411000001104	Captopril
Acepril 12.5mg tablets (Bristol-Myers Squibb Pharmaceuticals Ltd)	10841000033119	660111000001107	Captopril
Tensopril 12.5mg tablets (Teva UK Ltd)	2928341000033114	673711000001107	Captopril

Kaplon 12.5mg tablets (Teva UK Ltd)	1622041000033110	777711000001105	Captopril
Captopril 12.5mg tablets	213441000033119	318820009	Captopril
Captopril 25mg tablets	214441000033117	318821008	Captopril
Ecopace 25mg tablets (Advanz Pharma)	2945941000033116	817411000001108	Captopril
Acepril 25mg tablets (Bristol-Myers Squibb Pharmaceuticals Ltd)	10941000033110	517711000001103	Captopril
Kaplon 25mg tablets (Teva UK Ltd)	1622141000033114	477111000001100	Captopril
Tensopril 25mg tablets (Teva UK Ltd)	2928441000033115	597511000001109	Captopril
Capoten 25mg tablets (Bristol-Myers Squibb Pharmaceuticals Ltd)	214241000033118	455611000001103	Captopril
Capoten 50mg tablets (Bristol-Myers Squibb Pharmaceuticals Ltd)	214341000033111	386611000001109	Captopril
Kaplon 50mg tablets (Teva UK Ltd)	1622241000033119	572511000001102	Captopril
Acepril 50mg tablets (Bristol-Myers Squibb Pharmaceuticals Ltd)	11041000033117	814111000001108	Captopril
Ecopace 50mg tablets (Advanz Pharma)	2946041000033114	767911000001101	Captopril
Tensopril 50mg tablets (Teva UK Ltd)	2928541000033119	653111000001108	Captopril
Captopril 50mg tablets	214541000033116	318824000	Captopril
Captopril 10mg/5ml oral suspension	5008141000033111	8346811000001109	Captopril
Captopril 12.5mg/5ml oral solution	5898741000033114	8347111000001104	Captopril
Captopril 12.5mg/5ml oral suspension	5898841000033116	8347211000001105	Captopril
Captopril 15mg/5ml oral suspension	5401641000033118	8347411000001109	Captopril
Captopril 20mg/5ml oral suspension	2719841000033113	8348311000001101	Captopril
Captopril 25mg/5ml oral solution	3012741000033115	8348511000001107	Captopril
Captopril 25mg/5ml oral suspension	5132041000033112	8348611000001106	Captopril
Captopril 3mg/5ml oral solution	3851341000033117	8350511000001107	Captopril
Captopril 5mg/5ml oral solution	6001041000033115	8351611000001101	Captopril
Captopril 5mg/5ml oral suspension	2656341000033110	8351811000001102	Captopril
Captopril 6.25mg/5ml oral solution	5898941000033112	8351911000001107	Captopril
Captopril 6.25mg/5ml oral suspension	5899041000033115	8352011000001100	Captopril
Captopril 2mg capsules	3940841000033110	8791811000001109	Captopril
Captopril 4mg capsules	5454041000033117	8791911000001104	Captopril
Captopril 8mg/5ml oral suspension	3333941000033117	19820711000001101	Captopril
Noyada 25mg/5ml oral solution (Martindale Pharmaceuticals Ltd)	8961341000033119	23681711000001107	Captopril
Captopril 25mg/5ml oral solution sugar free	9108041000033111	23707311000001108	Captopril
Noyada 5mg/5ml oral solution (Martindale Pharmaceuticals Ltd)	8961241000033112	23682011000001102	Captopril
Captopril 5mg/5ml oral solution sugar free	9108141000033110	23707511000001102	Captopril
Irbesartan 300mg / Hydrochlorothiazide 12.5mg tablets	3161241000033111	134460003	Hydrochlorothiazide/ Irbesartan
CoAprovel 300mg/12.5mg tablets (Sanofi)	2216241000033110	682711000001109	Hydrochlorothiazide/ Irbesartan
CoAprovel 150mg/12.5mg tablets (Sanofi)	2216141000033115	792411000001108	Hydrochlorothiazide/ Irbesartan
Irbesartan 150mg / Hydrochlorothiazide 12.5mg tablets	3161141000033116	134461004	Hydrochlorothiazide/ Irbesartan
Co-Betaloc tablets (Pfizer Ltd)	372741000033116	2977611000001106	Hydrochlorothiazide/ Metoprolol tartrate

Metoprolol 100mg / Hydrochlorothiazide 12.5mg tablets	3161341000033118	318546001	Hydrochlorothiazide/ Metoprolol tartrate
Secadrex 200mg/12.5mg tablets (Sanofi)	1274641000033111	878911000001106	Acebutolol hydrochloride/ Hydrochlorothiazide
Acebutolol 200mg / Hydrochlorothiazide 12.5mg tablets	3162941000033114	318586009	Acebutolol hydrochloride/ Hydrochlorothiazide
Monozide 10 tablets (Wyeth Pharmaceuticals)	937341000033111	4542911000001104	Bisoprolol fumarate/ Hydrochlorothiazide
Enalapril 20mg / Hydrochlorothiazide 12.5mg tablets	3162241000033117	318849001	Enalapril maleate/ Hydrochlorothiazide
Innozide 20mg/12.5mg tablets (Merck Sharp & Dohme Ltd)	770141000033115	146811000001108	Enalapril maleate/ Hydrochlorothiazide
Zestoretic 20 tablets (AstraZeneca UK Ltd)	1552341000033118	3143111000001100	Hydrochlorothiazide/ Lisinopril
Carace 20 Plus tablets (Merck Sharp & Dohme Ltd)	217041000033116	3143511000001109	Hydrochlorothiazide/ Lisinopril
Caralpha 20mg/12.5mg tablets (Actavis UK Ltd)	3136141000033118	7385711000001106	Hydrochlorothiazide/ Lisinopril
Lisicostad 20mg/12.5mg tablets (Genus Pharmaceuticals Ltd)	3279641000033119	8145911000001100	Hydrochlorothiazide/ Lisinopril
Lisinopril 20mg / Hydrochlorothiazide 12.5mg tablets	3160941000033113	318880006	Hydrochlorothiazide/ Lisinopril
Lisoretic 20mg/12.5mg tablets (Bristol Laboratories Ltd)	13443541000033119	38240011000001105	Hydrochlorothiazide/ Lisinopril
Lisinopril 10mg / Hydrochlorothiazide 12.5mg tablets	3160841000033117	318884002	Hydrochlorothiazide/ Lisinopril
Caralpha 10mg/12.5mg tablets (Actavis UK Ltd)	3136041000033117	7385911000001108	Hydrochlorothiazide/ Lisinopril
Lisicostad 10mg/12.5mg tablets (Genus Pharmaceuticals Ltd)	3279541000033115	8145711000001102	Hydrochlorothiazide/ Lisinopril
Zestoretic 10 tablets (AstraZeneca UK Ltd)	1551841000033118	3144311000001101	Hydrochlorothiazide/ Lisinopril
Carace 10 Plus tablets (Merck Sharp & Dohme Ltd)	209841000033115	3144511000001107	Hydrochlorothiazide/ Lisinopril
Lisoretic 10mg/12.5mg tablets (Bristol Laboratories Ltd)	13443441000033115	38240211000001100	Hydrochlorothiazide/ Lisinopril
Quinapril 10mg / Hydrochlorothiazide 12.5mg tablets	3160641000033118	318892006	Hydrochlorothiazide/ Quinapril hydrochloride
Accuretic 10mg/12.5mg tablets (Pfizer Ltd)	11241000033113	260211000001104	Hydrochlorothiazide/ Quinapril hydrochloride
Losartan 50mg / Hydrochlorothiazide 12.5mg tablets	3161741000033117	318959004	Hydrochlorothiazide/ Losartan potassium
Cozaar-Comp 50mg/12.5mg tablets (Merck Sharp & Dohme Ltd)	370841000033110	255911000001105	Hydrochlorothiazide/ Losartan potassium
Hydrochlorothiazide 25mg tablets	744941000033110	376209006	Hydrochlorothiazide
Hydrosaluric 25mg tablets (Merck Sharp & Dohme Ltd)	742541000033112	4544011000001109	Hydrochlorothiazide
Hydrochlorothiazide 50mg tablets	745441000033118	376508004	Hydrochlorothiazide

Hydrosaluric 50mg tablets (Merck Sharp & Dohme Ltd)	742641000033113	4546211000001109	Hydrochlorothiazide
Co-Diovan 80mg/12.5mg tablets (Novartis Pharmaceuticals UK Ltd)	3190841000033119	8150111000001108	Hydrochlorothiazide/ Valsartan
Valsartan 80mg / Hydrochlorothiazide 12.5mg tablets	3190741000033112	377488008	Hydrochlorothiazide/ Valsartan
Cozaar-Comp 100mg/25mg tablets (Merck Sharp & Dohme Ltd)	3346941000033113	9566911000001105	Hydrochlorothiazide/ Losartan potassium
Losartan 100mg / Hydrochlorothiazide 25mg tablets	3346841000033117	395497004	Hydrochlorothiazide/ Losartan potassium
Chlorothiazide 250mg tablets	5093641000033110	395516007	Chlorothiazide
Valsartan 160mg / Hydrochlorothiazide 12.5mg tablets	3161441000033112	395521005	Hydrochlorothiazide/ Valsartan
Co-Diovan 160mg/12.5mg tablets (Novartis Pharmaceuticals UK Ltd)	3152541000033112	7668611000001104	Hydrochlorothiazide/ Valsartan
Telmisartan 40mg / Hydrochlorothiazide 12.5mg tablets	3162641000033119	407855002	Hydrochlorothiazide/ Telmisartan
MicardisPlus 40mg/12.5mg tablets (Boehringer Ingelheim Ltd)	2864241000033110	3806311000001109	Hydrochlorothiazide/ Telmisartan
Tolucombi 40mg/12.5mg tablets (Consilient Health Ltd)	9292641000033116	24573211000001101	Hydrochlorothiazide/ Telmisartan
Actelsar HCT 40mg/12.5mg tablets (Actavis UK Ltd)	9159341000033112	24411411000001100	Hydrochlorothiazide/ Telmisartan
MicardisPlus 80mg/12.5mg tablets (Boehringer Ingelheim Ltd)	2864341000033117	3806911000001105	Hydrochlorothiazide/ Telmisartan
Telmisartan 80mg / Hydrochlorothiazide 12.5mg tablets	3162741000033111	407856001	Hydrochlorothiazide/ Telmisartan
Actelsar HCT 80mg/12.5mg tablets (Actavis UK Ltd)	9159441000033118	24412511000001109	Hydrochlorothiazide/ Telmisartan
Tolucombi 80mg/12.5mg tablets (Consilient Health Ltd)	9292741000033113	24573411000001102	Hydrochlorothiazide/ Telmisartan
Chlorothiazide 250mg/5ml oral suspension	246241000033116	408039005	Chlorothiazide
Olmetec Plus 20mg/12.5mg tablets (Daiichi Sankyo UK Ltd)	3909141000033116	10261511000001103	Hydrochlorothiazide/ Olmesartan medoxomil
Olmesartan medoxomil 20mg / Hydrochlorothiazide 12.5mg tablets	3908941000033112	409184002	Hydrochlorothiazide/ Olmesartan medoxomil
Olmesartan medoxomil 40mg / Hydrochlorothiazide 12.5mg tablets	5566341000033111	409185001	Hydrochlorothiazide/ Olmesartan medoxomil
Olmetec Plus 40mg/12.5mg tablets (Daiichi Sankyo UK Ltd)	5566441000033117	17220911000001102	Hydrochlorothiazide/ Olmesartan medoxomil
Valsartan 160mg / Hydrochlorothiazide 25mg tablets	3161541000033113	409298002	Hydrochlorothiazide/ Valsartan
Co-Diovan 160mg/25mg tablets (Novartis Pharmaceuticals UK Ltd)	3152641000033113	7668911000001105	Hydrochlorothiazide/ Valsartan
Co-triamterzide 50mg/25mg tablets	370241000033111	410896007	Hydrochlorothiazide/ Triamterene
Triamaxco 50mg/25mg tablets (Ashbourne Pharmaceuticals Ltd)	1468141000033118	721311000001104	Hydrochlorothiazide/ Triamterene
Dyazide 50mg/25mg tablets (Advanz Pharma)	492341000033115	132811000001106	Hydrochlorothiazide/ Triamterene
Triam-Co 50mg/25mg tablets (IVAX Pharmaceuticals UK Ltd)	1470341000033112	191211000001106	Hydrochlorothiazide/ Triamterene
Chlorothiazide 150mg/5ml oral suspension	5899341000033118	8358511000001105	Chlorothiazide
Chlorothiazide 25mg/5ml oral suspension	5899441000033112	8358711000001100	Chlorothiazide

Hydrochlorothiazide 50mg/5ml oral solution	2746441000033111	8529611000001101	Hydrochlorothiazide
Olmesartan medoxomil 20mg / Hydrochlorothiazide 25mg tablets	3909041000033115	10270711000001105	Hydrochlorothiazide/ Olmesartan medoxomil
Olmetec Plus 20mg/25mg tablets (Daiichi Sankyo UK Ltd)	3909241000033111	10261811000001100	Hydrochlorothiazide/ Olmesartan medoxomil
Irbesartan 300mg / Hydrochlorothiazide 25mg tablets	3995241000033112	10970311000001105	Hydrochlorothiazide/ Irbesartan
CoAprovel 300mg/25mg tablets (Sanofi)	3995341000033119	10968611000001106	Hydrochlorothiazide/ Irbesartan
Chlorothiazide 200mg/5ml oral suspension	3924141000033111	12502311000001102	Chlorothiazide
Chlorothiazide 250mg/5ml oral solution	5991141000033110	12503011000001109	Chlorothiazide
Hydrochlorothiazide 5mg/5ml oral solution	5969841000033119	12538711000001107	Hydrochlorothiazide
Hydrochlorothiazide 5mg/5ml oral suspension	5969941000033110	12538811000001104	Hydrochlorothiazide
Losartan 100mg / Hydrochlorothiazide 12.5mg tablets	4424741000033110	13112711000001103	Hydrochlorothiazide/ Losartan potassium
Cozaar-Comp 100mg/12.5mg tablets (Merck Sharp & Dohme Ltd)	4424841000033117	13094111000001102	Hydrochlorothiazide/ Losartan potassium
MicardisPlus 80mg/25mg tablets (Boehringer Ingelheim Ltd)	4548841000033118	13719711000001103	Hydrochlorothiazide/ Telmisartan
Telmisartan 80mg / Hydrochlorothiazide 25mg tablets	4548741000033111	13731911000001109	Hydrochlorothiazide/ Telmisartan
Actelsar HCT 80mg/25mg tablets (Accord Healthcare Ltd)	9159541000033117	24413611000001103	Hydrochlorothiazide/ Telmisartan
Tolucombi 80mg/25mg tablets (Consilient Health Ltd)	9292841000033115	24573611000001104	Hydrochlorothiazide/ Telmisartan
Spironolactone 3mg / Chlorothiazide 30mg capsules	9278541000033112	16072811000001103	Chlorothiazide/ Spironolactone
Spironolactone 4mg / Chlorothiazide 40mg capsules	7681541000033117	18520511000001107	Chlorothiazide/ Spironolactone
Co-Betaloc SA tablets (Pfizer Ltd)	372841000033114	3853411000001104	Hydrochlorothiazide/ Metoprolol tartrate
Chlortalidone 50mg tablets	3086241000033111	317935006	Chlortalidone
Hygroton 50mg tablets (Alliance Pharmaceuticals Ltd)	742841000033114	285911000001101	Chlortalidone
Cilazapril 500microgram tablets	258441000033119	318915008	Cilazapril monohydrate
Vascace 500microgram tablets (Roche Products Ltd)	1504941000033117	3740211000001108	Cilazapril monohydrate
Vascace 1mg tablets (Roche Products Ltd)	1504641000033112	3669711000001105	Cilazapril monohydrate
Cilazapril 1mg tablets	258141000033110	318916009	Cilazapril monohydrate
Cilazapril 2.5mg tablets	258241000033115	318917000	Cilazapril monohydrate
Vascace 2.5mg tablets (Roche Products Ltd)	1504741000033115	3672411000001101	Cilazapril monohydrate
Vascace 5mg tablets (Roche Products Ltd)	1503441000033113	3671311000001104	Cilazapril monohydrate
Cilazapril 5mg tablets	258641000033117	318923005	Cilazapril monohydrate
Catapres 100microgram tablets (Boehringer Ingelheim Ltd)	215341000033112	215111000001101	Clonidine hydrochloride
Clonidine 100microgram tablets	285841000033118	318667005	Clonidine hydrochloride

Clonidine 300microgram tablets	287241000033110	318668000	Clonidine hydrochloride
Catapres 300microgram tablets (Boehringer Ingelheim Ltd)	215441000033118	368711000001103	Clonidine hydrochloride
Dixarit 25microgram tablets (Boehringer Ingelheim Ltd)	464341000033111	344511000001105	Clonidine hydrochloride
Clonidine 25microgram tablets	285941000033114	322840006	Clonidine hydrochloride
Clonidine 50micrograms/5ml oral solution	5900141000033118	8398511000001100	Clonidine hydrochloride
Clonidine 50micrograms/5ml oral suspension	5900241000033113	8398611000001101	Clonidine hydrochloride
Clonidine 100micrograms/5ml oral suspension	12569041000033115	11813011000001109	Clonidine hydrochloride
Catapres 150micrograms/1ml solution for injection ampoules (Boehringer Ingelheim Ltd)	196241000033111	364911000001108	Clonidine hydrochloride
Clonidine 150micrograms/1ml solution for injection ampoules	275641000033110	36089211000001103	Clonidine hydrochloride
Clonidine 250microgram modified-release capsules	261841000033118	36089511000001100	Clonidine hydrochloride
Clonidine 50micrograms/5ml oral solution sugar free	12684041000033114	36392711000001102	Clonidine hydrochloride
Aldactide 25 tablets (Pfizer Ltd)	40241000033110	762511000001105	Hydroflumethiazide/ Spironolactone
Co-flumactone 25mg/25mg tablets	377041000033116	318127005	Hydroflumethiazide/ Spironolactone
Co-flumactone 50mg/50mg tablets	377141000033117	318128000	Hydroflumethiazide/ Spironolactone
Aldactide 50 tablets (Pfizer Ltd)	40341000033117	4669111000001107	Hydroflumethiazide/ Spironolactone
Dytac 50mg capsules (Advanz Pharma)	491641000033116	3907911000001100	Triamterene
Triamterene 50mg capsules	1455341000033115	318082004	Triamterene
Triamterene 50mg / Benzthiazide 25mg capsules	3161941000033119	318098006	Benzthiazide/ Triamterene
Dytide capsules (Mercury Pharma Group Ltd)	491741000033113	714911000001108	Benzthiazide/ Triamterene
Kalspare tablets (DHP Healthcare Ltd)	796541000033115	3252011000001105	Chlortalidone/ Triamterene
Triamterene 50mg / Chlortalidone 50mg tablets	3162341000033110	318100006	Chlortalidone/ Triamterene
Triamterene 50mg / Furosemide 40mg tablets	3092741000033119	318101005	Furosemide/ Triamterene
Frusene 50mg/40mg tablets (Orion Pharma (UK) Ltd)	615041000033112	25411000001108	Furosemide/ Triamterene
Navidrex 500microgram tablets (Advanz Pharma)	955441000033111	487811000001100	Cyclopenthiazide
Cyclopenthiazide 500microgram tablets	395041000033117	317940003	Cyclopenthiazide
Trasidrex modified-release tablets (Mercury Pharma Group Ltd)	1469941000033114	3444411000001107	Cyclopenthiazide/ Oxprenolol hydrochloride
Co-prenozide 160mg/0.25mg modified-release tablets	377341000033119	36091611000001101	Cyclopenthiazide/ Oxprenolol hydrochloride
Pralenal 2.5 tablets (Opus Pharmaceuticals Ltd)	1918641000033112	448611000001106	Enalapril maleate
Innovace 2.5mg tablets (Merck Sharp & Dohme Ltd)	769141000033117	749611000001107	Enalapril maleate
Enalapril 2.5mg tablets	523541000033115	318850001	Enalapril maleate
Enalapril 5mg tablets	522841000033111	318851002	Enalapril maleate
Innovace 5mg tablets (Merck Sharp & Dohme Ltd)	769341000033119	730211000001104	Enalapril maleate
Pralenal 5 tablets (Opus Pharmaceuticals Ltd)	1918741000033115	578511000001100	Enalapril maleate

Innovace 10mg tablets (Merck Sharp & Dohme Ltd)	769041000033116	316111000001104	Enalapril maleate
Pralenal 10 tablets (Opus Pharmaceuticals Ltd)	1918841000033113	761411000001101	Enalapril maleate
Enalapril 10mg tablets	522641000033110	318853004	Enalapril maleate
Enalapril 20mg tablets	522741000033118	318855006	Enalapril maleate
Pralenal 20 tablets (Opus Pharmaceuticals Ltd)	1918941000033117	508511000001105	Enalapril maleate
Innovace 20mg tablets (Merck Sharp & Dohme Ltd)	769241000033112	302311000001104	Enalapril maleate
Enalapril 1.25mg/5ml oral solution	6012441000033118	8485511000001103	Enalapril maleate
Enalapril 1.25mg/5ml oral suspension	6012541000033117	8485611000001104	Enalapril maleate
Enalapril 10mg/5ml oral solution	5967041000033117	8485711000001108	Enalapril maleate
Enalapril 10mg/5ml oral suspension	5967141000033118	8485811000001100	Enalapril maleate
Enalapril 5mg/5ml oral solution	5992541000033111	8486911000001103	Enalapril maleate
Enalapril 5mg/5ml oral suspension	5992641000033112	8487011000001104	Enalapril maleate
Enalapril 5mg/5ml oral suspension sugar free	3279241000033117	20092911000001108	Enalapril maleate
Inspra 25mg tablets (Upjohn UK Ltd)	3199041000033116	8477211000001104	Eplerenone
Eplerenone 25mg tablets	3198841000033117	407011007	Eplerenone
Eplerenone 50mg tablets	3198941000033113	407012000	Eplerenone
Inspra 50mg tablets (Upjohn UK Ltd)	3199141000033117	8479811000001106	Eplerenone
Teveten 300mg tablets (Mylan)	2040841000033113	401211000001105	Eprosartan mesilate
Eprosartan 300mg tablets	2036341000033115	318994006	Eprosartan mesilate
Eprosartan 400mg tablets	2036441000033114	318995007	Eprosartan mesilate
Teveten 400mg tablets (Abbott Healthcare Products Ltd)	2040941000033117	151411000001103	Eprosartan mesilate
Teveten 600mg tablets (Mylan)	2041041000033110	872011000001109	Eprosartan mesilate
Eprosartan 600mg tablets	2036541000033110	318996008	Eprosartan mesilate
Felodipine 2.5mg modified-release / Ramipril 2.5mg tablets	3163341000033119	318176004	Felodipine/ Ramipril
Triapin 2.5mg/2.5mg modified-release tablets (Sanofi)	8264941000033119	4093211000001109	Felodipine/ Ramipril
Triapin 5mg/5mg modified-release tablets (Sanofi)	1834341000033110	3887911000001109	Felodipine/ Ramipril
Felodipine 5mg modified-release / Ramipril 5mg tablets	3163241000033112	318177008	Felodipine/ Ramipril
Plendil 2.5mg modified-release tablets (AstraZeneca UK Ltd)	1093941000033116	562711000001104	Felodipine
Cabren 2.5mg modified-release tablets (Teva UK Ltd)	2928841000033117	3800311000001106	Felodipine
Neofel XL 2.5mg tablets (Actavis UK Ltd)	4521541000033113	18167311000001103	Felodipine
Felotens XL 2.5mg tablets (Thornton & Ross Ltd)	4429241000033115	13127311000001107	Felodipine
Folpik XL 2.5mg tablets (Teva UK Ltd)	5576441000033112	13565311000001101	Felodipine
Cardioplen XL 2.5mg tablets (Chiesi Ltd)	4152841000033112	11506711000001103	Felodipine
Felodipine 2.5mg modified-release tablets	568041000033111	39020311000001104	Felodipine
Parmid XL 2.5mg tablets (Sandoz Ltd)	9121541000033117	24221811000001105	Felodipine
Plendil 10mg modified-release tablets (AstraZeneca UK Ltd)	1094041000033119	48511000001101	Felodipine
Cabren 10mg modified-release tablets (Teva UK Ltd)	2929041000033116	3800711000001105	Felodipine
Felogen XL 10mg tablets (Mylan)	3177541000033111	4973011000001100	Felodipine
Folpik XL 10mg tablets (Teva UK Ltd)	5576341000033118	5008911000001100	Felodipine

Felotens XL 10mg tablets (Thornton & Ross Ltd)	2980041000033115	4785511000001107	Felodipine
Vascalpha 10mg modified-release tablets (Accord Healthcare Ltd)	3034441000033111	5638811000001106	Felodipine
Parmid XL 10mg tablets (Sandoz Ltd)	9121741000033113	7388311000001103	Felodipine
Neofel XL 10mg tablets (Kent Pharmaceuticals Ltd)	4521741000033117	8089811000001107	Felodipine
Cardioplen XL 10mg tablets (Chiesi Ltd)	3154941000033110	7887511000001107	Felodipine
Felodipine 10mg modified-release tablets	569041000033115	39020511000001105	Felodipine
Cardioplen XL 5mg tablets (Chiesi Ltd)	3154841000033119	7887011000001104	Felodipine
Neofel XL 5mg tablets (Kent Pharmaceuticals Ltd)	4521641000033114	8090111000001106	Felodipine
Parmid XL 5mg tablets (Sandoz Ltd)	9121641000033116	7387911000001103	Felodipine
Vascalpha 5mg modified-release tablets (Accord Healthcare Ltd)	3034341000033117	5638311000001102	Felodipine
Felotens XL 5mg tablets (Thornton & Ross Ltd)	2979941000033119	4785111000001103	Felodipine
Folpik XL 5mg tablets (Teva UK Ltd)	5576541000033113	5008511000001107	Felodipine
Felogen XL 5mg tablets (Mylan)	3177441000033110	4972811000001103	Felodipine
Cabren 5mg modified-release tablets (Teva UK Ltd)	2928941000033113	3800511000001100	Felodipine
Plendil 5mg modified-release tablets (AstraZeneca UK Ltd)	1094141000033115	490211000001101	Felodipine
Felodipine 5mg modified-release tablets	569141000033116	39020611000001109	Felodipine
Fosinopril 10mg tablets	609141000033111	318909008	Fosinopril sodium
Staril 10mg tablets (Bristol-Myers Squibb Pharmaceuticals Ltd)	1387341000033114	462511000001104	Fosinopril sodium
Staril 20mg tablets (Bristol-Myers Squibb Pharmaceuticals Ltd)	1387441000033115	348111000001107	Fosinopril sodium
Fosinopril 20mg tablets	609241000033116	318910003	Fosinopril sodium
Hydralazine 25mg tablets	741941000033113	318649003	Hydralazine hydrochloride
Apresoline 25mg tablets (Advanz Pharma)	74341000033114	657011000001101	Hydralazine hydrochloride
Hydralazine 50mg tablets	742041000033119	318650003	Hydralazine hydrochloride
Hydralazine 10mg tablets	12877141000033119	414426001	Hydralazine hydrochloride
Apo-Hydralazine 10mg tablets (Imported (United States))	12877241000033114	10637411000001100	Hydralazine hydrochloride
Hydralazine 10mg/5ml oral suspension	4456241000033111	8528611000001105	Hydralazine hydrochloride
Hydralazine 25mg/5ml oral solution	11072741000033116	8580911000001106	Hydralazine hydrochloride
Apresoline 20mg powder for solution for injection ampoules (Advanz Pharma)	73441000033117	3925011000001100	Hydralazine hydrochloride
Hydralazine 20mg powder for solution for injection ampoules	734141000033114	34193811000001100	Hydralazine hydrochloride
Tanatril 20mg tablets (Mitsubishi Tanabe Pharma Europe Ltd)	2145741000033116	533511000001106	Imidapril hydrochloride
Imidapril 20mg tablets	2145841000033114	318942009	Imidapril hydrochloride
Imidapril 5mg tablets	1739841000033119	318943004	Imidapril hydrochloride
Tanatril 5mg tablets (Mitsubishi Tanabe Pharma Europe Ltd)	1753941000033114	797911000001103	Imidapril hydrochloride
Tanatril 10mg tablets (Mitsubishi Tanabe Pharma Europe Ltd)	1753841000033118	680811000001102	Imidapril hydrochloride
Imidapril 10mg tablets	1739741000033112	318944005	Imidapril hydrochloride

Indapamide 2.5mg tablets	768641000033118	317956008	Indapamide hemihydrate
Nindaxa 2.5 tablets (Ashbourne Pharmaceuticals Ltd)	769741000033118	424311000001108	Indapamide hemihydrate
Natrilix 2.5mg tablets (Servier Laboratories Ltd)	955341000033117	321811000001109	Indapamide hemihydrate
Coversyl Plus tablets (Servier Laboratories Ltd)	2739541000033117	562511000001109	Indapamide/ Perindopril erbumine
Perindopril erbumine 4mg / Indapamide 1.25mg tablets	3161641000033114	3437611000001100	Indapamide/ Perindopril erbumine
Perindopril arginine 5mg / Indapamide 1.25mg tablets	4454541000033118	13454311000001101	Indapamide/ Perindopril arginine
Coversyl Arginine Plus 5mg/1.25mg tablets (Servier Laboratories Ltd)	4454641000033117	13444311000001104	Indapamide/ Perindopril arginine
Indapamide 2.5mg/5ml oral suspension	8197041000033112	21578911000001105	Indapamide
Perindopril tosilate 5mg / Indapamide 1.25mg tablets	8263641000033112	21940011000001101	Indapamide/ Perindopril tosilate
Natrilix SR 1.5mg tablets (Servier Laboratories Ltd)	953141000033119	456611000001108	Indapamide
Rawel XL 1.5mg tablets (Consilient Health Ltd)	5566041000033114	16737911000001105	Indapamide
Ethibide XL 1.5mg tablets (Genus Pharmaceuticals Ltd)	4571241000033110	13824811000001102	Indapamide
Tensaid XL 1.5mg tablets (Mylan)	4656741000033119	14242211000001100	Indapamide
Varbim XL 1.5mg tablets (Teva UK Ltd)	5816741000033112	14693611000001102	Indapamide
Indipam XL 1.5mg tablets (Accord Healthcare Ltd)	5053141000033113	15436111000001104	Indapamide
Mapemid XL 1.5mg tablets (Teva UK Ltd)	5128741000033110	15600711000001107	Indapamide
Indapamide 1.5mg modified-release tablets	765741000033116	39020711000001100	Indapamide
Cardide SR 1.5mg tablets (Teva UK Ltd)	9121841000033115	24331611000001104	Indapamide
Alkapamid XL 1.5mg tablets (HBS Healthcare Ltd)	12312641000033118	34494911000001106	Indapamide
Lorvacs XL 1.5mg tablets (Torrent Pharma (UK) Ltd)	12988441000033113	36812711000001103	Indapamide
Indoramin 20mg tablets	768541000033119	318739007	Indoramin hydrochloride
Doralese Tiltab 20mg tablets (Chemidex Pharma Ltd)	480541000033110	3354611000001100	Indoramin hydrochloride
Baratol 25mg tablets (Amdipharm Plc)	116741000033118	3689111000001107	Indoramin hydrochloride
Indoramin 25mg tablets	769841000033111	318740009	Indoramin hydrochloride
Irbesartan 75mg tablets	775641000033115	318968002	Irbesartan
Aprovel 75mg tablets (Sanofi)	76141000033117	434511000001104	Irbesartan
Sabervel 75mg tablets (Aspire Pharma Ltd)	8193941000033116	21522211000001102	Irbesartan
Ifirmasta 75mg tablets (Consilient Health Ltd)	8554141000033116	22720311000001108	Irbesartan
Ifirmasta 150mg tablets (Consilient Health Ltd)	8554241000033111	22720611000001103	Irbesartan
Sabervel 150mg tablets (Aspire Pharma Ltd)	8194041000033119	21522411000001103	Irbesartan
Aprovel 150mg tablets (Sanofi)	75941000033114	859711000001103	Irbesartan
Irbesartan 150mg tablets	775441000033117	318969005	Irbesartan
Irbesartan 300mg tablets	775541000033116	318970006	Irbesartan
Aprovel 300mg tablets (Sanofi)	76041000033116	323211000001107	Irbesartan
Sabervel 300mg tablets (Aspire Pharma Ltd)	8194141000033115	21522611000001100	Irbesartan

Ifirmasta 300mg tablets (Consilient Health Ltd)	8554341000033118	22720811000001104	Irbesartan
Irbesartan 150mg/5ml oral suspension	5970641000033117	8580811000001101	Irbesartan
Irbesartan 300mg/5ml oral suspension	4954341000033111	12639511000001103	Irbesartan
Irbesartan 20mg oral powder sachets	6167841000033112	18852211000001107	Irbesartan
Irbesartan 30mg oral powder sachets	6423941000033116	19281511000001102	Irbesartan
Irbesartan 37.5mg oral powder sachets	6435741000033117	19481911000001105	Irbesartan
Prescal 2.5mg tablets (Novartis Pharmaceuticals UK Ltd)	1140241000033117	3689711000001108	Isradipine
Isradipine 2.5mg tablets	784641000033112	319280009	Isradipine
Lacidipine 2mg tablets	817541000033119	319300008	Lacidipine
Motens 2mg tablets (GlaxoSmithKline UK Ltd)	937041000033114	910911000001108	Lacidipine
Motens 4mg tablets (GlaxoSmithKline UK Ltd)	937141000033113	333011000001100	Lacidipine
Lacidipine 4mg tablets	817641000033118	319301007	Lacidipine
Molap 4mg tablets (Rivopharm (UK) Ltd)	10217641000033116	28882211000001100	Lacidipine
Lacidipine 6mg tablets	13344441000033112	38019311000001102	Lacidipine
Lercanidipine 10mg tablets	825941000033112	319316005	Lercanidipine hydrochloride
Zanidip 10mg tablets (Recordati Pharmaceuticals Ltd)	1550841000033111	20011000001105	Lercanidipine hydrochloride
Lercanidipine 20mg tablets	3908741000033114	10225911000001102	Lercanidipine hydrochloride
Zanidip 20mg tablets (Recordati Pharmaceuticals Ltd)	3908841000033116	10198711000001102	Lercanidipine hydrochloride
Zestril 2.5mg tablets (AstraZeneca UK Ltd)	1552041000033115	593111000001108	Lisinopril
Carace 2.5mg tablets (Bristol-Myers Squibb Pharmaceuticals Ltd)	216041000033118	321011000001103	Lisinopril
Lisinopril 2.5mg tablets	837941000033119	318857003	Lisinopril
Lisinopril 5mg tablets	838141000033117	318858008	Lisinopril
Carace 5mg tablets (Bristol-Myers Squibb Pharmaceuticals Ltd)	216241000033114	778011000001109	Lisinopril
Zestril 5mg tablets (AstraZeneca UK Ltd)	1552241000033111	823211000001109	Lisinopril
Zestril 10mg tablets (AstraZeneca UK Ltd)	1551941000033114	825311000001100	Lisinopril
Carace 10mg tablets (Bristol-Myers Squibb Pharmaceuticals Ltd)	215941000033111	315211000001104	Lisinopril
Lisinopril 10mg tablets	837841000033110	318859000	Lisinopril
Lisinopril 20mg tablets	838041000033116	318860005	Lisinopril
Carace 20mg tablets (Bristol-Myers Squibb Pharmaceuticals Ltd)	216141000033119	56711000001109	Lisinopril
Zestril 20mg tablets (AstraZeneca UK Ltd)	1552141000033116	891711000001107	Lisinopril
Lisinopril 2.5mg/5ml oral solution	5971341000033117	8622111000001109	Lisinopril
Lisinopril 2.5mg/5ml oral suspension	5971441000033111	8622211000001103	Lisinopril
Lisinopril 20mg/5ml oral solution	6044641000033119	8622311000001106	Lisinopril
Lisinopril 20mg/5ml oral suspension	6044541000033115	8622411000001104	Lisinopril
Lisinopril 5mg/5ml oral solution	5890241000033117	8622511000001100	Lisinopril
Lisinopril 5mg/5ml oral suspension	3152141000033115	8622611000001101	Lisinopril
Lisinopril 7.5mg/5ml oral solution	5971541000033112	8622811000001102	Lisinopril
Lisinopril 7.5mg/5ml oral suspension	5971641000033113	8622911000001107	Lisinopril
Lisinopril 40mg/5ml oral suspension	5568741000033113	20556011000001103	Lisinopril

Lisinopril 5mg/5ml oral solution sugar free	10645041000033114	30251811000001106	Lisinopril
Losartan 25mg tablets	851841000033117	318955005	Losartan potassium
Cozaar 25mg tablets (Merck Sharp & Dohme Ltd)	370441000033112	266511000001104	Losartan potassium
Cozaar 50mg tablets (Merck Sharp & Dohme Ltd)	370541000033113	53611000001106	Losartan potassium
Losartan 50mg tablets	851941000033113	318956006	Losartan potassium
Losartan 100mg tablets	2720141000033115	407784004	Losartan potassium
Cozaar 100mg tablets (Merck Sharp & Dohme Ltd)	2720241000033110	245811000001102	Losartan potassium
Losartan 50mg/5ml oral solution	5971941000033118	14159411000001106	Losartan potassium
Losartan 50mg/5ml oral suspension	5972041000033112	14159511000001105	Losartan potassium
Losartan 12.5mg tablets	4957541000033114	15148111000001100	Losartan potassium
Cozaar 12.5mg tablets (Merck Sharp & Dohme Ltd)	4957641000033110	15138911000001101	Losartan potassium
Losartan 100mg/5ml oral solution	5971741000033116	15451111000001107	Losartan potassium
Losartan 100mg/5ml oral suspension	5971841000033114	15451211000001101	Losartan potassium
Cozaar 2.5mg/ml oral suspension (Merck Sharp & Dohme Ltd)	5150041000033110	15506811000001105	Losartan potassium
Losartan 2.5mg/ml oral suspension sugar free	5149941000033118	15507411000001105	Losartan potassium
Methyldopa 125mg tablets	897341000033110	318671008	Methyldopa anhydrous
Methyldopa 250mg tablets	897441000033116	318672001	Methyldopa anhydrous
Aldomet 250mg tablets (Aspen Pharma Trading Ltd)	40941000033118	253711000001107	Methyldopa anhydrous
Aldomet 500mg tablets (Aspen Pharma Trading Ltd)	41041000033111	73611000001108	Methyldopa anhydrous
Methyldopa 500mg tablets	897541000033115	318673006	Methyldopa anhydrous
Methyldopa 250mg/5ml oral suspension	892341000033112	8667311000001100	Methyldopa anhydrous
Metenix 5mg tablets (Sanofi)	896841000033116	375611000001109	Metolazone
Metolazone 5mg tablets	903541000033116	317965001	Metolazone
Zaroxolyn 5mg tablets (Imported (Canada))	8196641000033116	21574611000001109	Metolazone
Metolazone 2.5mg tablets	8196441000033118	374173002	Metolazone
Zaroxolyn 2.5mg tablets (Imported (Canada))	8196541000033117	21574911000001103	Metolazone
Loniten 10mg tablets (Pfizer Ltd)	850441000033112	3666711000001100	Minoxidil
Minoxidil 10mg tablets	920641000033119	318657000	Minoxidil
Minoxidil 5mg tablets	920941000033114	318656009	Minoxidil
Loniten 5mg tablets (Pfizer Ltd)	850641000033114	3666411000001106	Minoxidil
Loniten 2.5mg tablets (Pfizer Ltd)	850541000033113	3667011000001104	Minoxidil
Minoxidil 2.5mg tablets	920841000033118	318655008	Minoxidil
Perdix 7.5mg tablets (UCB Pharma Ltd)	1066041000033113	4041111000001109	Moexipril hydrochloride
Moexipril 7.5mg tablets	938141000033112	318934008	Moexipril hydrochloride
Moexipril 15mg tablets	938041000033113	318935009	Moexipril hydrochloride
Perdix 15mg tablets (UCB Pharma Ltd)	1065941000033115	4040311000001101	Moexipril hydrochloride
Moxonidine 200microgram tablets	938541000033115	318707000	Moxonidine
Physiotens 200microgram tablets (Mylan)	1080841000033119	41111000001102	Moxonidine

Physiotens 400microgram tablets (Mylan)	1080941000033110	142811000001107	Moxonidine
Moxonidine 400microgram tablets	938641000033119	318708005	Moxonidine
Moxonidine 300microgram tablets	2635641000033111	408604009	Moxonidine
Physiotens 300microgram tablets (Mylan)	2635741000033119	522011000001109	Moxonidine
Cardene 20mg capsules (Astellas Pharma Ltd)	176741000033114	344811000001108	Nicardipine hydrochloride
Nicardipine 20mg capsules	967741000033115	319217004	Nicardipine hydrochloride
Nicardipine 30mg capsules	967141000033119	319218009	Nicardipine hydrochloride
Cardene 30mg capsules (Astellas Pharma Ltd)	176841000033116	291111000001102	Nicardipine hydrochloride
Nicardipine 10mg/10ml solution for infusion ampoules	9697741000033114	27126711000001103	Nicardipine hydrochloride
Cardene SR 30mg capsules (Astellas Pharma Ltd)	198941000033118	540311000001105	Nicardipine hydrochloride
Nicardipine 30mg modified-release capsules	971441000033112	39021711000001108	Nicardipine hydrochloride
Cardene SR 45mg capsules (Astellas Pharma Ltd)	199041000033110	118811000001102	Nicardipine hydrochloride
Nicardipine 45mg modified-release capsules	971541000033113	39021811000001100	Nicardipine hydrochloride
Adalat 5mg capsules (Bayer Plc)	14141000033116	27111000001107	Nifedipine
Nifedipine 5mg capsules	967341000033116	319222004	Nifedipine
Nifedipine 10mg capsules	967241000033114	319223009	Nifedipine
Adalat 10mg capsules (Bayer Plc)	14041000033115	782511000001108	Nifedipine
Angiopine 10 capsules (Ashbourne Pharmaceuticals Ltd)	61841000033111	811311000001107	Nifedipine
Nifedipine 10mg/5ml oral suspension	5973741000033117	8670111000001106	Nifedipine
Nifedipine 5mg/5ml oral suspension	5056241000033112	8670311000001108	Nifedipine
Nifedipine 20mg/ml oral drops	2639341000033117	9096811000001100	Nifedipine
Nifedipine 2.5mg/5ml oral suspension	5402541000033112	12303311000001109	Nifedipine
Coracten XL 60mg capsules (UCB Pharma Ltd)	1725041000033113	3381511000001105	Nifedipine
Nifedipine 60mg modified-release capsules	1778841000033111	38896511000001101	Nifedipine
Coracten SR 20mg capsules (UCB Pharma Ltd)	364241000033112	389611000001101	Nifedipine
Nifedipine 20mg modified-release capsules	974141000033119	39022511000001106	Nifedipine
Slofedipine XL 60 tablets (Zentiva)	1753041000033113	630411000001107	Nifedipine
Adalat LA 60mg tablets (Bayer Plc)	17241000033117	235511000001104	Nifedipine
Adipine XL 60mg tablets (Chiesi Ltd)	3225541000033116	9049911000001105	Nifedipine
Nimodrel XL 60mg tablets (Zurich Pharmaceuticals)	3952741000033118	10189311000001108	Nifedipine
Neozipine XL 60mg tablets (Kent Pharmaceuticals Ltd)	5817541000033118	10751411000001101	Nifedipine
Valni XL 60mg tablets (Zentiva)	4452041000033116	13402111000001101	Nifedipine
Adanif XL 60mg tablets (Advanz Pharma)	5892041000033114	17666211000001101	Nifedipine
Nifedipine 60mg modified-release tablets	971341000033118	39022611000001105	Nifedipine
Nidef 60mg modified-release tablets (Morningside Healthcare Ltd)	12372441000033111	34685811000001103	Nifedipine
Cardilate MR 10mg tablets (Teva UK Ltd)	1580841000033116	25911000001100	Nifedipine
Calchan MR 10 tablets (Ranbaxy (UK) Ltd)	2295641000033116	627111000001104	Nifedipine
Angiopine MR 10mg tablets (Ashbourne Pharmaceuticals Ltd)	66141000033114	568911000001104	Nifedipine

Adalat retard 10mg tablets (Bayer Plc)	19441000033113	569011000001108	Nifedipine
Tensipine MR 10 tablets (Genus Pharmaceuticals Ltd)	1421241000033110	413111000001101	Nifedipine
Adipine MR 10 tablets (Chiesi Ltd)	18341000033114	833611000001109	Nifedipine
Nifedipress MR 10 tablets (Dexcel-Pharma Ltd)	1745041000033110	904011000001104	Nifedipine
Nifedipine 10mg modified-release tablets	971841000033110	39022711000001101	Nifedipine
Cardilate MR 20mg tablets (IVAX Pharmaceuticals UK Ltd)	199141000033114	905711000001103	Nifedipine
Slofedipine 20mg tablets (Sterwin Medicines)	1697641000033111	843411000001105	Nifedipine
Adalat LA 20mg tablets (Bayer Plc)	1766341000033113	881811000001100	Nifedipine
Angiopine MR 20mg tablets (Ashbourne Pharmaceuticals Ltd)	65241000033114	865011000001105	Nifedipine
Hypolar Retard 20 tablets (Sandoz Ltd)	738141000033119	677411000001108	Nifedipine
Valni 20 Retard tablets (Tillomed Laboratories Ltd)	2955041000033115	693311000001101	Nifedipine
Tensipine MR 20 tablets (Genus Pharmaceuticals Ltd)	1421341000033117	385611000001104	Nifedipine
Nifedipress MR 20 tablets (Dexcel-Pharma Ltd)	1745141000033114	619111000001101	Nifedipine
Calchan MR 20 tablets (Ranbaxy (UK) Ltd)	2295741000033113	17011000001100	Nifedipine
Adalat retard 20mg tablets (Bayer Plc)	19341000033119	5011000001109	Nifedipine
Adipine MR 20 tablets (Chiesi Ltd)	18241000033116	74111000001103	Nifedipine
Nifopress Retard 20mg tablets (Advanz Pharma)	2189541000033117	280811000001100	Nifedipine
Coroday MR 20mg tablets (Mylan)	2051241000033116	309611000001102	Nifedipine
Nifedipine 20mg modified-release tablets	970941000033113	39022811000001109	Nifedipine
Coracten SR 10mg capsules (UCB Pharma Ltd)	336641000033114	126411000001108	Nifedipine
Nifedipine 10mg modified-release capsules	971141000033116	39022911000001104	Nifedipine
Coracten XL 30mg capsules (UCB Pharma Ltd)	1724941000033113	162811000001100	Nifedipine
Nifedipine 30mg modified-release capsules	1778741000033118	39107511000001109	Nifedipine
Fortipine LA 40 tablets (Advanz Pharma)	605941000033110	188711000001108	Nifedipine
Nifedipine 40mg modified-release tablets	972441000033116	39107611000001108	Nifedipine
Adalat LA 30mg tablets (Bayer Plc)	17141000033112	2881311000001105	Nifedipine
Hypolar XL 30 tablets (Sandoz Ltd)	2776541000033111	2881811000001101	Nifedipine
Slofedipine XL 30mg tablets (Zentiva)	1752941000033115	2882011000001104	Nifedipine
Nimodrel XL 30mg tablets (Zurich Pharmaceuticals)	3952641000033110	10189111000001106	Nifedipine
Adipine XL 30mg tablets (Chiesi Ltd)	3225441000033117	9049711000001108	Nifedipine
Neozipine XL 30mg tablets (Kent Pharmaceuticals Ltd)	5817441000033119	10751211000001100	Nifedipine
Valni XL 30mg tablets (Zentiva)	4451941000033110	13401911000001109	Nifedipine
Adanif XL 30mg tablets (Advanz Pharma)	5891941000033115	17666011000001106	Nifedipine
Nifedipine 30mg modified-release tablets	971241000033111	39111711000001105	Nifedipine
Nidef 30mg modified-release tablets (Morningside Healthcare Ltd)	12372341000033117	34685211000001104	Nifedipine
Nimotop 30mg tablets (Bayer Plc)	977741000033114	3879211000001104	Nimodipine
Nimodipine 30mg tablets	977441000033119	323273000	Nimodipine
Nimotop 0.02% solution for infusion 50ml vials (Bayer Plc)	970841000033117	4387211000001100	Nimodipine
Nimodipine 10mg/50ml solution for infusion vials	970741000033110	36031711000001104	Nimodipine
Syscor MR 10 tablets (Forest Laboratories UK Ltd)	1401341000033110	3877011000001100	Nisoldipine

Nisoldipine 10mg modified-release tablets	972141000033112	36031811000001107	Nisoldipine
Syscor MR 20 tablets (Forest Laboratories UK Ltd)	1401441000033116	4070011000001100	Nisoldipine
Nisoldipine 20mg modified-release tablets	972241000033117	36031911000001102	Nisoldipine
Syscor MR 30 tablets (Forest Laboratories UK Ltd)	1401541000033115	4069211000001108	Nisoldipine
Nisoldipine 30mg modified-release tablets	972341000033110	36032011000001109	Nisoldipine
Olmesartan medoxomil 20mg tablets	2944641000033112	385542009	Olmesartan medoxomil
Olmetec 20mg tablets (Daiichi Sankyo UK Ltd)	2944941000033117	4624311000001103	Olmesartan medoxomil
Olmetec 40mg tablets (Daiichi Sankyo UK Ltd)	2945041000033117	4624611000001108	Olmesartan medoxomil
Olmesartan medoxomil 40mg tablets	2944741000033115	385543004	Olmesartan medoxomil
Olmesartan medoxomil 10mg tablets	2944541000033111	408055003	Olmesartan medoxomil
Olmetec 10mg tablets (Daiichi Sankyo UK Ltd)	2944841000033113	4624011000001101	Olmesartan medoxomil
Olmesartan medoxomil 10mg/5ml oral suspension	4273241000033118	14680711000001103	Olmesartan medoxomil
Perindopril erbumine 2mg tablets	1067841000033115	318896009	Perindopril erbumine
Coversyl 2mg tablets (Servier Laboratories Ltd)	377541000033114	48211000001104	Perindopril erbumine
Coversyl 4mg tablets (Servier Laboratories Ltd)	377641000033110	902211000001100	Perindopril erbumine
Perindopril erbumine 4mg tablets	1067941000033111	318897000	Perindopril erbumine
Perindopril erbumine 8mg tablets	2846741000033119	374667004	Perindopril erbumine
Coversyl 8mg tablets (Servier Laboratories Ltd)	2846641000033111	3803711000001101	Perindopril erbumine
Perindopril erbumine 4mg/5ml oral suspension	8276041000033117	8671311000001103	Perindopril erbumine
Coversyl Arginine 10mg tablets (Servier Laboratories Ltd)	4454441000033119	13444611000001109	Perindopril arginine
Perindopril arginine 10mg tablets	4454141000033110	13454111000001103	Perindopril arginine
Perindopril arginine 2.5mg tablets	4453941000033114	13454211000001109	Perindopril arginine
Coversyl Arginine 2.5mg tablets (Servier Laboratories Ltd)	4454241000033115	13445211000001108	Perindopril arginine
Perindopril arginine 5mg tablets	4454041000033111	13454411000001108	Perindopril arginine
Coversyl Arginine 5mg tablets (Servier Laboratories Ltd)	4454341000033113	13444911000001103	Perindopril arginine
Perindopril erbumine 8mg/5ml oral solution	8275941000033110	14057311000001102	Perindopril erbumine
Perindopril erbumine 8mg/5ml oral suspension	8276141000033118	14057411000001109	Perindopril erbumine
Perindopril tosilate 10mg tablets	8263541000033111	21939811000001102	Perindopril tosilate
Perindopril tosilate 2.5mg tablets	8263341000033116	21939911000001107	Perindopril tosilate
Perindopril tosilate 5mg tablets	8263441000033110	21940111000001100	Perindopril tosilate
Perindopril erbumine 4mg/5ml oral solution	8869441000033119	23471511000001106	Perindopril erbumine
Polythiazide 1mg tablets	1110141000033119	317967009	Polythiazide
Nephril 1mg tablets (Pfizer Ltd)	965441000033115	4549211000001100	Polythiazide
Hypovase 500microgram tablets (Pfizer Ltd)	743541000033118	321311000001100	Prazosin hydrochloride

Prazosin 500microgram tablets	1131341000033118	318767003	Prazosin hydrochloride
Prazosin 1mg tablets	1131441000033112	318768008	Prazosin hydrochloride
Hypovase 1mg tablets (Pfizer Ltd)	743641000033117	347411000001101	Prazosin hydrochloride
Alphavase 1 tablets (Ashbourne Pharmaceuticals Ltd)	37741000033118	934411000001100	Prazosin hydrochloride
Alphavase 2 tablets (Ashbourne Pharmaceuticals Ltd)	37841000033111	570011000001106	Prazosin hydrochloride
Hypovase 2mg tablets (Pfizer Ltd)	743741000033114	150911000001104	Prazosin hydrochloride
Prazosin 2mg tablets	1131541000033113	318769000	Prazosin hydrochloride
Prazosin 5mg tablets	1131641000033114	318770004	Prazosin hydrochloride
Alphavase 5 tablets (Ashbourne Pharmaceuticals Ltd)	37941000033115	74711000001102	Prazosin hydrochloride
Prazosin 500micrograms/5ml oral solution	11567441000033119	13078011000001104	Prazosin hydrochloride
Accupro 5mg tablets (Pfizer Ltd)	13441000033110	582611000001106	Quinapril hydrochloride
Quinil 5mg tablets (Tillomed Laboratories Ltd)	3283141000033118	9207711000001100	Quinapril hydrochloride
Quinapril 5mg tablets	1149241000033111	318885001	Quinapril hydrochloride
Quinapril 10mg tablets	1149341000033118	318886000	Quinapril hydrochloride
Quinil 10mg tablets (Tillomed Laboratories Ltd)	3283241000033113	9208111000001100	Quinapril hydrochloride
Accupro 10mg tablets (Pfizer Ltd)	13241000033114	231111000001106	Quinapril hydrochloride
Accupro 20mg tablets (Pfizer Ltd)	13341000033116	829111000001100	Quinapril hydrochloride
Quinil 20mg tablets (Tillomed Laboratories Ltd)	3283341000033115	9208411000001105	Quinapril hydrochloride
Quinapril 20mg tablets	1149441000033112	318887009	Quinapril hydrochloride
Accupro 40mg tablets (Pfizer Ltd)	11641000033111	86411000001109	Quinapril hydrochloride
Quinil 40mg tablets (Tillomed Laboratories Ltd)	3283441000033114	9208711000001104	Quinapril hydrochloride
Quinapril 40mg tablets	1149541000033113	318894007	Quinapril hydrochloride
Ramipril 1.25mg capsules	1151341000033117	318900007	Ramipril
Tritace 1.25mg capsules (Aventis Pharma)	1455541000033110	111611000001109	Ramipril
Tritace 2.5mg capsules (Sanofi)	1455641000033111	835411000001105	Ramipril
Lopace 2.5mg capsules (Discovery Pharmaceuticals)	3159741000033111	7948711000001102	Ramipril
Ramipril 2.5mg capsules	1151441000033111	318901006	Ramipril
Ramipril 5mg capsules	1151541000033112	318902004	Ramipril
Lopace 5mg capsules (Discovery Pharmaceuticals)	3159841000033118	7948911000001100	Ramipril
Tritace 5mg capsules (Sanofi)	1455741000033119	802311000001101	Ramipril
Tritace 10mg capsules (Sanofi)	1769941000033115	43711000001100	Ramipril
Lopace 10mg capsules (Discovery Pharmaceuticals)	3159941000033114	7949111000001105	Ramipril
Ramipril 10mg capsules	1769841000033111	318906001	Ramipril
Ramipril 1.25mg tablets	2989541000033115	408040007	Ramipril
Tritace 1.25mg tablets (Sanofi)	2989941000033114	5010511000001106	Ramipril

Tritace 2.5mg tablets (Sanofi)	2990041000033116	5010811000001109	Ramipril
Ramipril 2.5mg tablets	2989641000033119	408050008	Ramipril
Ramipril 5mg tablets	2989741000033111	408051007	Ramipril
Tritace 5mg tablets (Sanofi)	2990141000033117	5011111000001108	Ramipril
Tritace 10mg tablets (Sanofi)	2990241000033112	5011411000001103	Ramipril
Ramipril 10mg tablets	2989841000033118	408052000	Ramipril
Ramipril 1.25mg/5ml oral solution	5998241000033112	8720211000001109	Ramipril
Ramipril 1.25mg/5ml oral suspension	4152241000033113	8720311000001101	Ramipril
Ramipril 10mg/5ml oral solution	5887841000033118	8720411000001108	Ramipril
Ramipril 10mg/5ml oral suspension	5887941000033114	8720511000001107	Ramipril
Ramipril 2.5mg/5ml oral solution	5890541000033115	8720611000001106	Ramipril
Ramipril 2.5mg/5ml oral suspension	4805941000033114	8720711000001102	Ramipril
Ramipril 5mg/5ml oral solution	5890341000033110	8720811000001105	Ramipril
Ramipril 5mg/5ml oral suspension	2883341000033118	8720911000001100	Ramipril
Ramipril 2.5mg/5ml oral solution sugar free	6517041000033110	19877111000001100	Ramipril
Sacubitril 24mg / Valsartan 26mg tablets	10943641000033117	31142011000001103	Sacubitril/ Valsartan
Entresto 24mg/26mg tablets (Novartis Pharmaceuticals UK Ltd)	10943941000033112	31136811000001109	Sacubitril/ Valsartan
Sacubitril 49mg / Valsartan 51mg tablets	10943741000033114	31142111000001102	Sacubitril/ Valsartan
Entresto 49mg/51mg tablets (Novartis Pharmaceuticals UK Ltd)	10944041000033114	31136411000001107	Sacubitril/ Valsartan
Sacubitril 97mg / Valsartan 103mg tablets	10943841000033116	31142211000001108	Sacubitril/ Valsartan
Entresto 97mg/103mg tablets (Novartis Pharmaceuticals UK Ltd)	10944141000033113	31138011000001104	Sacubitril/ Valsartan
Spironolactone 25mg tablets	1374241000033118	318056008	Spironolactone
Aldactone 25mg tablets (Pfizer Ltd)	40541000033112	930511000001105	Spironolactone
Aldactone 50mg tablets (Pfizer Ltd)	40641000033113	921811000001103	Spironolactone
Spironolactone 50mg tablets	1374341000033111	318057004	Spironolactone
Spironolactone 100mg tablets	1374141000033113	318058009	Spironolactone
Spirospare 100 tablets (Ashbourne Pharmaceuticals Ltd)	1374541000033116	3411000001104	Spironolactone
Aldactone 100mg tablets (Pfizer Ltd)	40441000033111	421611000001100	Spironolactone
Lasilactone 20mg/50mg capsules (Sanofi)	808841000033119	3645811000001107	Furosemide/ Spironolactone
Spironolactone 50mg / Furosemide 20mg capsules	3162541000033115	318102003	Furosemide/ Spironolactone
Spironolactone 5mg/5ml oral suspension	1371141000033111	8726311000001103	Spironolactone
Spironolactone 50mg/5ml oral suspension	1371341000033114	8726411000001105	Spironolactone
Spironolactone 25mg/5ml oral suspension	1371041000033112	8726511000001109	Spironolactone
Spironolactone 15mg/5ml oral suspension	5975441000033113	8727011000001103	Spironolactone
Spironolactone 10mg/5ml oral suspension	1371241000033116	8727311000001100	Spironolactone
Spironolactone 100mg/5ml oral suspension	2011541000033114	8727611000001105	Spironolactone
Spironolactone 250mg/5ml oral solution	5975541000033114	13353511000001101	Spironolactone
Spironolactone 250mg/5ml oral suspension	5975641000033110	13353611000001102	Spironolactone
Spironolactone 100mg/5ml oral solution	5999141000033111	13894611000001109	Spironolactone

Spironolactone 10mg/5ml oral solution	5999241000033116	13894711000001100	Spironolactone
Spironolactone 25mg/5ml oral solution	5890141000033112	13894811000001108	Spironolactone
Spironolactone 50mg/5ml oral solution	5890441000033116	13894911000001103	Spironolactone
Spironolactone 5mg/5ml oral solution	5999341000033114	13895011000001103	Spironolactone
Telmisartan 20mg tablets	2272641000033119	134463001	Telmisartan
Micardis 20mg tablets (Boehringer Ingelheim Ltd)	2272741000033111	648711000001100	Telmisartan
Tolura 20mg tablets (Consilient Health Ltd)	9209341000033116	24555211000001104	Telmisartan
Micardis 40mg tablets (Boehringer Ingelheim Ltd)	1849741000033114	924911000001106	Telmisartan
Telmisartan 40mg tablets	1849541000033118	318986004	Telmisartan
Tolura 40mg tablets (Consilient Health Ltd)	9209441000033110	24555411000001100	Telmisartan
Telmisartan 80mg tablets	1849641000033117	318987008	Telmisartan
Micardis 80mg tablets (Boehringer Ingelheim Ltd)	1849841000033116	527411000001102	Telmisartan
Tolura 80mg tablets (Consilient Health Ltd)	9209541000033111	24555611000001102	Telmisartan
Hytrin 5mg tablets (Advanz Pharma)	745841000033115	3154311000001103	Terazosin hydrochloride
Terazosin 5mg tablets	1429141000033119	318776005	Terazosin hydrochloride
Benph 5mg tablets (Mylan)	11807841000033119	33628611000001102	Terazosin hydrochloride
Terazosin 10mg tablets	1429041000033118	318777001	Terazosin hydrochloride
Hytrin 10mg tablets (Advanz Pharma)	745641000033116	3150911000001107	Terazosin hydrochloride
Hytrin 2mg tablets (Advanz Pharma)	745741000033113	3147611000001100	Terazosin hydrochloride
Terazosin 2mg tablets	1425341000033119	318779003	Terazosin hydrochloride
Benph 2mg tablets (Mylan)	11799841000033112	33630711000001107	Terazosin hydrochloride
Trandolapril 500microgram capsules	1454141000033119	318924004	Trandolapril
Gopten 500microgram capsules (Abbott Laboratories Ltd)	648541000033117	253511000001102	Trandolapril
Odrik 500microgram capsules (Aventis Pharma)	998741000033119	227511000001106	Trandolapril
Gopten 1mg capsules (Abbott Laboratories Ltd)	648341000033112	346811000001101	Trandolapril
Odrik 1mg capsules (Aventis Pharma)	998541000033110	432911000001102	Trandolapril
Trandolapril 1mg capsules	1453941000033115	318925003	Trandolapril
Trandolapril 2mg capsules	1454041000033118	318926002	Trandolapril
Gopten 2mg capsules (Abbott Laboratories Ltd)	648441000033118	273111000001109	Trandolapril
Odrik 2mg capsules (Aventis Pharma)	998641000033111	140511000001108	Trandolapril
Gopten 4mg capsules (Abbott Laboratories Ltd)	3014841000033118	5651611000001103	Trandolapril
Trandolapril 4mg capsules	3014741000033111	410958005	Trandolapril
Tarka modified-release capsules (Abbott Laboratories Ltd)	1406341000033117	3691211000001101	Trandolapril/ Verapamil hydrochloride
Verapamil 180mg modified-release / Trandolapril 2mg capsules	3163041000033116	36149211000001102	Trandolapril/ Verapamil hydrochloride
Trimetazidine 20mg tablets	7747541000033112	15243911000001109	Trimetazidine dihydrochloride
Trimetazidine 35mg modified-release tablets	10495841000033113	20456111000001105	Trimetazidine dihydrochloride

Vastarel MR 35mg tablets (Imported (France))	5492841000033111	20434311000001100	Trimetazidine dihydrochloride
Diovan 40mg capsules (Novartis Pharmaceuticals UK Ltd)	437741000033110	777611000001101	Valsartan
Valsartan 40mg capsules	1498341000033118	318961008	Valsartan
Valsartan 80mg capsules	1498441000033112	318962001	Valsartan
Diovan 80mg capsules (Novartis Pharmaceuticals UK Ltd)	437841000033117	554511000001105	Valsartan
Diovan 160mg capsules (Novartis Pharmaceuticals UK Ltd)	437641000033118	117011000001107	Valsartan
Valsartan 160mg capsules	1498241000033111	318963006	Valsartan
Valsartan 80mg tablets	6515441000033113	375034009	Valsartan
Valsartan 160mg tablets	6515541000033114	375035005	Valsartan
Valsartan 320mg tablets	4424941000033113	376487009	Valsartan
Diovan 320mg tablets (Novartis Pharmaceuticals UK Ltd)	4425041000033113	13143311000001102	Valsartan
Valsartan 40mg tablets	3201341000033115	416515008	Valsartan
Diovan 40mg tablets (Novartis Pharmaceuticals UK Ltd)	3201441000033114	8263211000001101	Valsartan
Valsartan 3mg/ml oral solution	6528441000033113	20007411000001100	Valsartan
Diovan 3mg/1ml oral solution (Novartis Pharmaceuticals UK Ltd)	6528541000033114	20001711000001102	Valsartan
Diurexan 20mg tablets (Mylan)	470741000033113	348911000001105	Xipamide
Xipamide 20mg tablets	1543941000033111	317970008	Xipamide
Doxazosin 1mg tablets	480641000033111	318781001	Doxazosin mesilate
Cardura 1mg tablets (Upjohn UK Ltd)	216741000033115	907711000001109	Doxazosin mesilate
Doxadura 1mg tablets (Dexcel-Pharma Ltd)	2957941000033110	4857511000001108	Doxazosin mesilate
Doxadura 2mg tablets (Dexcel-Pharma Ltd)	2958041000033113	4857711000001103	Doxazosin mesilate
Cascor 2mg tablets (Ranbaxy (UK) Ltd)	2760041000033110	904511000001107	Doxazosin mesilate
Cardura 2mg tablets (Upjohn UK Ltd)	216841000033113	41811000001109	Doxazosin mesilate
Doxazosin 2mg tablets	480741000033119	318782008	Doxazosin mesilate
Doxazosin 4mg tablets	480841000033112	318783003	Doxazosin mesilate
Cascor 4mg tablets (Ranbaxy (UK) Ltd)	2760141000033114	179311000001107	Doxazosin mesilate
Doxadura 4mg tablets (Dexcel-Pharma Ltd)	2958141000033112	4858111000001103	Doxazosin mesilate
Doxazosin 8mg tablets	12682141000033115	421069003	Doxazosin mesilate
Doxazosin 1mg/5ml oral suspension	4432941000033119	8483411000001101	Doxazosin mesilate
Doxazosin 4mg/5ml oral suspension	5138341000033111	8483511000001102	Doxazosin mesilate
Doxazosin 4mg/5ml oral solution	8870641000033119	23466611000001105	Doxazosin mesilate
Doxazosin 1mg/5ml oral solution	9173941000033111	24509711000001103	Doxazosin mesilate
Oxandosin XL 4mg tablets (Ratiopharm UK Ltd)	4288341000033111	11812211000001105	Doxazosin mesilate
Colixil XL 4mg tablets (Sandoz Ltd)	5403641000033117	11757711000001107	Doxazosin mesilate
Doxadura XL 4mg tablets (Dexcel-Pharma Ltd)	4063741000033112	11269911000001101	Doxazosin mesilate
Slocinx XL 4mg tablets (Zentiva)	4021141000033118	11098311000001104	Doxazosin mesilate
Doxzogen XL 4mg tablets (Mylan)	4575941000033115	13811611000001108	Doxazosin mesilate
Cardozin XL 4mg tablets (Almus Pharmaceuticals Ltd)	4936841000033112	18197411000001107	Doxazosin mesilate
Raporsin XL 4mg tablets (Actavis UK Ltd)	6036141000033119	18164911000001108	Doxazosin mesilate

Larbex XL 4mg tablets (Teva UK Ltd)	5809641000033111	17338211000001104	Doxazosin mesilate
Cardura XL 4mg tablets (Upjohn UK Ltd)	2274041000033118	123911000001106	Doxazosin mesilate
Doxazosin 4mg modified-release tablets	2273841000033111	39020411000001106	Doxazosin mesilate
Cardura XL 8mg tablets (Upjohn UK Ltd)	2274141000033119	873411000001109	Doxazosin mesilate
Doxazosin 8mg modified-release tablets	2273941000033115	39021111000001107	Doxazosin mesilate

Table S14. Heart failure selection Aurum codes

Term	Medcode ID	SNOMED CT Concept ID	SNOMED CT Description ID
Acute left ventricular failure	300190010	195114002	300190010
Impaired left ventricular function	411506018	275514001	411506018
H/O: heart failure	251680018	161505003	251680018
Congestive heart failure due to valvular disease	2675255018	426611007	2675255018
Left ventricular cardiac dysfunction	2694523019	429589006	2694523019
CCF - Congestive cardiac failure	3182551000006115	42343007	493288018
Chronic pulmonary oedema	494669012	46847001	494669012
Acute congestive heart failure	18472010	10633002	18472010
COCM - Congestive cardiomyopathy	6615881000006112	399020009	1786620011
Hypertensive heart disease NOS with CCF	741681000006111	64715009	107545013
Congestive heart failure	70653017	42343007	70653017
Congestive cardiomyopathy	1778488011	399020009	1778488011
Cardiac failure	139482012	84114007	139482012
Heart failure with preserved ejection fraction	7321121000006119	446221000	3496968011
Congestive cardiac failure	3182541000006117	42343007	493287011
CCM - Congestive cardiomyopathy	6615871000006114	399020009	1786619017
Heart failure with preserved ejection fraction	2227501000000110	446221000	2227501000000110
Left ventricular failure	141306010	85232009	141306010
Chronic congestive heart failure	147247018	88805009	147247018
Heart failure NOS	395772015	84114007	139475013
HF - Heart failure	3868341000006118	84114007	1234906013
History of heart failure in last year	5990971000006113	309634009	2986867013
Pulmonary oedema NOS	301694014	19242006	479262018
Congestive obstructive cardiomyopathy	350413012	233871002	350413012
Biventricular failure	4005301000006110	92506005	510016018
Hypertensive heart&renal dis wth (congestive) heart failure	741701000006114	194779001	299672017
Left-sided heart failure	3886061000006119	85232009	201199018
Heart failure confirmed	1488804017	395105005	1488804017
Hyperten heart&renal dis+both(congestv)heart and renal fail	789941000006117	194781004	299674016
Decompensated cardiac failure	300179017	195111005	300179017
Compensated cardiac failure	300180019	195112003	300180019
Benign hypertensive heart disease with CCF	504901000006118	194767001	299653017
HFNEF - heart failure with normal ejection fraction	1661371000000112	446221000	1713091000000115
Severe left ventricular systolic dysfunction	1991651000006115	1991651000006104	1991651000006115
LVF - Left ventricular failure	3886071000006114	85232009	1235017018
Left heart failure	3886041000006118	85232009	141303019
Cardiac failure NOS	223981000000118	84114007	139482012
[RFC] Cardiac failure	905391000006119	905391000006103	905391000006119
Congestive cardiac failure	493287011	42343007	70653017

Acute heart failure	94251011	56675007	94251011
Post cardiac operation heart failure NOS	300217019	195130005	300214014
CHF - Congestive heart failure	3182561000006118	42343007	493289014
Heart failure	139475013	84114007	139475013
Heart failure with normal ejection fraction	1647701000000118	446221000	2883808011
Echocardiogram shows left ventricular systolic dysfunction	2159197017	407596008	2159197017
Echocardiogram shows left ventricular diastolic dysfunction	2159198010	407597004	2159198010
Pulmonary oedema - acute	1490256017	40541001	492666016
Malignant hypertensive heart disease with CCF	728671000006119	83105008	1236017010
H/O: Heart failure in last year	453099015	309634009	453099015
Biventricular failure	510016018	92506005	153058012
Impaired left ventricular function	784191000006110	275514001	411506018
History of heart failure	4540521000006111	161505003	2986453013
Malignant hypertensive heart disease with congestive heart failure	3852501000006115	83105008	137848017
Left ventricular systolic dysfunction	216207010	134401001	216207010

Table S15. Loop Diuretics Aurum product codes

Term from EMIS	Prod code ID	dmd ID	Drug substance name
Burinex 1mg tablets (LEO Pharma)	172741000033113	846411000001100	Bumetanide
Bumetanide 1mg tablets	172441000033118	318021009	Bumetanide
Bumetanide 5mg tablets	172541000033117	318022002	Bumetanide
Burinex 5mg tablets (LEO Pharma)	172841000033115	521811000001107	Bumetanide
Bumetanide 1mg/5ml oral solution sugar free	169041000033112	318023007	Bumetanide
Burinex A 5mg/1mg tablets (LEO Pharma)	172341000033112	33911000001104	Amiloride hydrochloride/ Bumetanide
Amiloride 5mg / Bumetanide 1mg tablets	1845641000033119	318097001	Amiloride hydrochloride/ Bumetanide
Bumetanide 5mg/5ml oral suspension	10713941000033113	30798811000001102	Bumetanide
Bumetanide 2mg/4ml solution for injection ampoules	3858741000033112	35912911000001108	Bumetanide
Burinex K modified-release tablets (LEO Pharma)	172941000033111	3290211000001109	Bumetanide/ Potassium chloride
Bumetanide 500microgram / Potassium chloride 573mg (potassium 7.7mmol) modified-release tablets	1845741000033111	35913011000001100	Bumetanide/ Potassium chloride
Furosemide 20mg tablets	3092241000033113	317971007	Furosemide
Lasix 20mg tablets (Borg Medicare)	816341000033112	701611000001102	Furosemide
Froop 40mg tablets (Ashbourne Pharmaceuticals Ltd)	615541000033119	714311000001107	Furosemide
Lasix 40mg tablets (Sanofi)	816441000033118	79411000001107	Furosemide
Frusid 40mg tablets (Dr Reddy's Laboratories (UK) Ltd)	615141000033111	99311000001108	Furosemide
Furosemide 40mg tablets	3092341000033115	317972000	Furosemide
Furosemide 500mg tablets	3092441000033114	317973005	Furosemide
Lasix 500mg tablets (Sanofi)	816541000033117	829911000001102	Furosemide
Diuresal 500mg tablets (Ennogen Pharma Ltd)	9808241000033110	27992311000001106	Furosemide
Lasix 5mg/5ml oral solution (Borg Medicare)	812041000033114	701111000001105	Furosemide
Triamterene 50mg / Furosemide 40mg tablets	3092741000033119	318101005	Furosemide/ Triamterene
Frusene 50mg/40mg tablets (Orion Pharma (UK) Ltd)	615041000033112	25411000001108	Furosemide/ Triamterene
Lasilactone 20mg/50mg capsules (Sanofi)	808841000033119	3645811000001107	Furosemide/ Spironolactone
Spironolactone 50mg / Furosemide 20mg capsules	3162541000033115	318102003	Furosemide/ Spironolactone
Frumil LS 20mg/2.5mg tablets (Sanofi)	614341000033113	550711000001106	Amiloride hydrochloride/ Furosemide
Co-amilofruse 2.5mg/20mg tablets	376841000033113	318135008	Amiloride hydrochloride/ Furosemide
Co-amilofruse 5mg/40mg tablets	376641000033112	318136009	Amiloride hydrochloride/ Furosemide
Frumil 40mg/5mg tablets (Sanofi)	614641000033117	427411000001106	Amiloride hydrochloride/ Furosemide

Froop Co 5mg/40mg tablets (Ashbourne Pharmaceuticals Ltd)	1611441000033117	331311000001103	Amiloride hydrochloride/ Furosemide
Lasoride 5mg/40mg tablets (Sanofi)	818941000033112	678511000001106	Amiloride hydrochloride/ Furosemide
Fru-Co 5mg/40mg tablets (Teva UK Ltd)	615241000033116	818511000001106	Amiloride hydrochloride/ Furosemide
Frumil Forte 10mg/80mg tablets (Sanofi)	615641000033118	82611000001106	Amiloride hydrochloride/ Furosemide
Co-amilofruse 10mg/80mg tablets	376741000033115	318137000	Amiloride hydrochloride/ Furosemide
Diumide-K Continus tablets (Teofarma)	470541000033117	4540011000001102	Furosemide/ Potassium chloride
Furosemide 40mg / Potassium chloride 600mg (potassium 8mmol) modified-release tablets	3161841000033110	4557711000001102	Furosemide/ Potassium chloride
Co-amilofruse 5mg/40mg/5ml oral suspension	5710441000033117	8427011000001108	Amiloride hydrochloride/ Furosemide
Furosemide 5mg/5ml oral solution	3924941000033113	13893411000001108	Furosemide
Furosemide 5mg/5ml oral suspension	5993341000033112	13893511000001107	Furosemide
Furosemide 250mg/25ml solution for injection ampoules	3954441000033119	34193711000001108	Furosemide
Lasikal modified-release tablets (Borg Medicare)	816141000033114	3704811000001105	Furosemide/ Potassium chloride
Furosemide 20mg / Potassium chloride 750mg (potassium 10mmol) modified-release tablets	3162441000033116	36061311000001109	Furosemide/ Potassium chloride
Lasix 20mg/2ml solution for injection ampoules (Sanofi)	811841000033111	9611000001104	Furosemide
Furosemide 20mg/2ml solution for injection ampoules	3091841000033115	36061411000001102	Furosemide
Furosemide 50mg/5ml solution for injection ampoules	3091741000033113	36061511000001103	Furosemide
Furosemide 80mg/8ml solution for injection Minijet pre-filled syringes (UCB Pharma Ltd)	3092641000033111	2898811000001106	Furosemide
Furosemide 80mg/8ml solution for injection pre-filled syringes	3092541000033110	36061611000001104	Furosemide
Frusol 50mg/5ml oral solution (Rosemont Pharmaceuticals Ltd)	1731741000033111	855611000001109	Furosemide
Furosemide 50mg/5ml oral solution sugar free	3092141000033118	36564411000001102	Furosemide
Frusol 40mg/5ml oral solution (Rosemont Pharmaceuticals Ltd)	1731641000033119	494811000001105	Furosemide
Furosemide 40mg/5ml oral solution sugar free	3092041000033117	39021211000001101	Furosemide
Frusol 20mg/5ml oral solution (Rosemont Pharmaceuticals Ltd)	1731541000033115	602811000001100	Furosemide
Furosemide 20mg/5ml oral solution sugar free	3091941000033111	39108611000001107	Furosemide
Torasemide 2.5mg tablets	1450241000033116	318040003	Torasemide
Torem 2.5mg tablets (Mylan)	1449941000033115	3699711000001107	Torasemide
Torem 5mg tablets (Mylan)	1450041000033112	3700311000001109	Torasemide
Torasemide 5mg tablets	1450341000033114	318041004	Torasemide
Torasemide 10mg tablets	1450141000033111	318042006	Torasemide

Torem 10mg tablets (Mylan)	1449841000033111	3700811000001100	Torasemide

Table S16. Gastrointestinal and intracranial Bleeding Aurum codes

Term	MedCode ID	SNOMED CT Concept ID	SNOMED CT Description ID
Cerebral haemorrhage	884421000006119	274100004	884421000006119
External capsule haemorrhage	300276019	195167002	300276019
Oesophageal varices with bleeding in diseases EC	300776018	195475003	300776018
Oesophageal varices without bleeding in diseases EC	300779013	195476002	300779013
H/O: GI Bleed	411546012	275551007	411546012
Cerebral infarction with haemorrhagic transformation	1573101000006112	1573101000006108	1573101000006112
H/O: upper GIT bleed	456218012	312489007	456218012
History of subarachnoid haemorrhage	4540691000006112	161515009	2986875019
H/O: GIT bleed	5574481000006112	275551007	3036845016
Duodenal ulcer with haemorrhage	302534015	27281001	483490017
Subarachnoid haemorrhage from middle cerebral artery	123511000006114	21454007	36011016
Subarachnoid haemorrhage from posterior communicating artery	123521000006118	21454007	36011016
Left sided intracerebral haemorrhage, unspecified	748941000006115	274100004	409859018
Traumatic subdural haematoma without open intracranial wound	459511018	315046005	459511018
Acute haemorrhagic pancreatitis	493743018	4399003	493743018
Chronic gastrojejunal ulcer with haemorrhage AND perforation	494304013	45640006	494304013
Internal capsule haemorrhage	496232015	52201006	496232015
Subarachnoid haemorrhage from basilar artery	123491000006115	276284000	412361011
Traumatic intracerebral haemorrhage	7378421000006118	450418003	2915664016
Unspecified duodenal ulcer with haemorrhage and perforation	302537010	51868009	86362013
Unspecified gastrojejunal ulcer with haemorrhage	302606019	16121001	27283019
Gastro-oesophageal laceration-haemorrhage syndrome	3068361000006115	35265002	1216440014
Intrapontine haemorrhage	2622631000006114	7713009	503468012
Bleeding internal haemorrhoids	3733931000006110	75884004	200553019
Evacuation of extradural haematoma	649541000006113	171713001	265968017
Evacuation of haematoma from temporal lobe of brain	265689012	171473007	265689012
Chronic peptic ulcer with haemorrhage AND perforation	498977018	61300005	498977018
Haematemesis	507679012	8765009	507679012
Acute gastric ulcer with haemorrhage	508664015	89748001	508664015
Oesophageal varices with bleeding	1216060010	17709002	1216060010
Subdural haematoma evacuation	3469891000006117	59712006	498519016
Cerebral haemorrhage	989201000006117	274100004	989201000006117
Evacuation of subdural haematoma	3469851000006111	59712006	498516011
Lobar cerebral haemorrhage	345675012	230710000	345675012
SAH - Subarachnoid haemorrhage	2841141000006111	21454007	1216125018
Gastrointestinal bleeding	3710521000006115	74474003	123695010

GI bleeding	3710501000006113	74474003	123689014
Oversew of blood vessel of duodenal ulcer	362951000000115	226891000000104	362951000000115
Subperiosteal haematoma	933081000006112	669341000000103	933081000006112
Subdural haemorrhage	884441000006114	195176009	884441000006114
DU - acute + haemorrhage	885851000006113	12847006	885851000006113
PU - acute + haemorrhage	885941000006118	12274003	885941000006118
PU - acute + h'ge + perf.	885961000006119	111353003	885961000006119
PU - chronic + haemorrhage	885981000006112	49232000	885981000006112
Upper GI - gastrointestinal haemorrhage	3102201000006117	37372002	1216567017
Right sided intracerebral haemorrhage, unspecified	163261000006119	195168007	300277011
Subarachnoid haemorrh from intracranial artery, unspecif	123441000006112	21454007	481028017
Intracerebral haemorrhage	12223101000006118	274100004	3673215016
Traumatic subdural haematoma	5459261000006114	262952002	2913152010
Extradural haemorrhage	884431000006116	397809001	884431000006116
Gastrointestinal bleed	802131000006119	74474003	1233753013
Intracerebral haemorrhage NOS	300287010	274100004	2819959010
Unspecified peptic ulcer with haemorrhage and perforation	302571018	13200003	22592012
Intracerebral haemorrhage with intraventricular haemorrhage	4777861000006119	195168007	2915438018
Intracranial haemorrhage following injury	7378271000006116	450410005	2915426010
Endoscopic injection haemostasis of duodenal ulcer	459010010	314627002	459010010
Oesophageal varices with bleeding, associated with another disorder	4779721000006113	195475003	2575715017
Non-traumatic subdural haemorrhage	2475119012	195176009	2469010019
Upper GI haemorrhage	3102191000006115	37372002	486703016
Evacuation of intracerebral haematoma	265692011	10458001	279429014
Gastric haemorrhage	303660017	61401005	499003013
Cerebral haemorrhage	122371000006118	274100004	409860011
[X]Other subarachnoid haemorrhage	300936018	21454007	481028017
Subarachnoid haemorrhage following injury with open intracranial wound	320735017	5251007	496337012
Traumatic subdural hematoma	391035012	262952002	2912898019
Subarachnoid haemorrhage from basilar artery aneurysm	5583101000006116	276284000	412361011
Bleeding gastric ulcer	2752321000006114	15902003	1221244018
Sequelae of intracerebral haemorrhage	300407010	195241001	300407010
Traumatic cranial subdural haematoma	5459221000006115	262952002	391036013
Oesophageal haemorrhage	2741641000006118	15238002	477249012
Intestinal haemorrhage NOS	303663015	74474003	123688018
Intracerebral haemorrhage, multiple localised	4777881000006112	195169004	300280012
Cerebral haemorrhage NOS	989211000006119	274100004	989211000006119
GU - acute + h'ge + perf.	885791000006113	48974009	885791000006113
GU - chronic + haemorrhage	885811000006112	57246001	885811000006112
GU - chronic + h'ge + perf.	885831000006118	76181002	885831000006118

PU - chronic + h'ge + perf.	886001000006111	61300005	886001000006111
Intracerebral haemorrhage (ICH)	744901000006114	274100004	2819959010
Cortical haemorrhage	495394013	49422009	495394013
Cerebellar haemorrhage	502878012	75038005	502878012
Bleeding chronic gastric ulcer	1231704018	57246001	1231704018
ICH - intracerebral haemorrhage	12223121000006111	274100004	3673217012
Bulbar haemorrhage	483988011	732923001	3467313018
Acute peptic ulcer with haemorrhage and perforation	361430016	111353003	361430016
Choroidal haemorrhage	297943011	122003	1256014
Chronic gastric ulcer with haemorrhage AND with perforation	554311000006114	76181002	503219010
Nontraumatic extradural haemorrhage	660111000006117	397809001	1773153015
Evacuation of haematoma from cerebellum	265690015	171474001	265690015
Gastrotomy and ligation of bleeding point of stomach	269184010	173778009	269184010
Melaena	5913016	2901004	5913016
Unspec peptic ulcer; unspec haemorrhage and/or perforation	81501000006111	13200003	22592012
Bleeding chronic duodenal ulcer	1235462014	89469000	1235462014
Gastrointestinal tract haemorrhage NOS	303667019	74474003	123688018
Subarachnoid haemorrhage from anterior communicating artery	123481000006118	21454007	36011016
Ruptured berry aneurysm	300242011	195154000	300242011
Subarachnoid haemorrhage from carotid siphon and bifurcation	300244012	195155004	300244012
Subarachnoid haemorrhage from vertebral artery	300253017	195160000	300253017
Subdural haemorrhage - nontraumatic	300294013	195176009	300294013
Sequelae of subarachnoid haemorrhage	300406018	195240000	300406018
Duodenal ulcer with perforation	302535019	88968005	147516013
Subdural haemorrhage	2534198011	35486000	486181011
Gastric ulcer with haemorrhage AND perforation	81311000006115	62366003	499256017
Corpus cavernosum haematoma	304493010	198031007	304493010
History of gastrointestinal bleed	397836015	275551007	411546012
GI - Gastrointestinal haemorrhage	3710541000006110	74474003	1216914011
Intracerebral haemorrhage in hemisphere, unspecified	744921000006116	274100004	409860011
Intracerebral haemorrhage, multiple localized	746571000006116	195169004	300280012
Cerebral haemorrhage	300939013	274100004	409860011
H/O: haematemesis	251739019	161538007	251739019
Cerebral haemorrhage NOS	884451000006111	700251000000105	884451000006111
Bleeding oesophageal varices	2781011000006119	17709002	478405010
Gastrointestinal hemorrhage	396403016	74474003	502715010
Unspec duodenal ulcer; unspec haemorrhage and/or perforation	81301000006118	51868009	86362013
Sequelae of other nontraumatic intracranial haemorrhage	149551000006111	363302008	482447013
DU - chronic + haemorrhage	885891000006119	89469000	885891000006119

Acute duodenal ulcer with haemorrhage	474430017	12847006	474430017
Basal ganglia haemorrhage	503791000006114	195165005	300272017
H/O: upper GIT haemorrhage	456217019	312489007	456217019
Acute peptic ulcer with haemorrhage	470365013	12274003	470365013
Gastric bleeding	3497621000006112	61401005	102011018
Intracerebral haemorrhage, intraventricular	300277011	195168007	300277011
Intracranial haemorrhage	300298011	1386000	475553012
Haemorrhage	300840014	131148009	3035879011
Subarachnoid haemorrhage NOS	300257016	21454007	481028017
Unspecified gastric ulcer with haemorrhage and perforation	302490012	62366003	499256017
Unspecified peptic ulcer with haemorrhage	302568014	13200003	22592012
Subarachnoid haemorrhage	481028017	21454007	481028017
[X]Intracerebral haemorrhage in hemisphere, unspecified	300956017	274100004	409860011
Pontine haemorrhage	503469016	7713009	503469016
Acute gastrojejunal ulcer with haemorrhage AND perforation	504674011	81387001	504674011
Cerebral haemorrhage following injury	505322013	450418003	2916363013
Traumatic cerebral haemorrhage	505324014	450418003	2916058017
Lower gastrointestinal haemorrhage	507736019	87763006	507736019
Evacuation of intracranial extradural haematoma	4645641000006117	171713001	265968017
Bleeding haemorrhoids	300752014	51551000	198339016
Gastric ulcer with haemorrhage	302487018	15902003	477573015
GI haemorrhage	3710531000006117	74474003	502714014
H/O subarachnoid haemorrhage	808111000006110	161515009	251701010
Traumatic subdural haematoma with open intracranial wound	459514014	315047001	459514014
Upper gastrointestinal haemorrhage	486704010	37372002	486704010
Acute gastric ulcer with haemorrhage AND perforation	495273013	48974009	495273013
Chronic gastric ulcer with haemorrhage	497833010	57246001	497833010
Evacuation of intracranial subdural haematoma	498516011	59712006	498520010
Oesophageal bleeding	2741601000006115	15238002	477250012
Other and unspecified intracranial haemorrhage	300290016	62914000	104563015
Epidural haemorrhage	505381016	82999001	505381016
Cerebral hemorrhage	605471000006112	274100004	409859018
Haemorrhage of oesophagus	477248016	15238002	477248016
Acute haemorrhagic gastritis	478553011	2367005	481682019
Chronic duodenal ulcer with haemorrhage AND perforation	486596015	36975000	486596015
Chronic peptic ulcer with haemorrhage	495344012	49232000	495344012
Acute gastrojejunal ulcer with haemorrhage	499719010	63954007	499719010
Gastrointestinal haemorrhage	502715010	74474003	502715010
Acute duodenal ulcer with haemorrhage AND perforation	507303017	86895006	507303017
Chronic gastrojejunal ulcer with haemorrhage	499386010	62838000	499386010

Chronic duodenal ulcer with haemorrhage	508524014	89469000	508524014
Bleeding acute gastric ulcer	1235493010	89748001	1235493010
Extradural haemorrhage following injury without open intracranial wound	402930018	43216008	493552012
Upper GI bleeding	3102181000006118	37372002	62348012
GU - acute + haemorrhage	885771000006112	89748001	885771000006112
History of upper gastrointestinal tract haemorrhage	6017201000006118	312489007	2986370018

Table S17. Gastrointestinal tract ulcer Aurum codes

Term	Medcode ID	SNOMED CT Concept ID	SNOMED CT Description ID
Acute duodenal ulcer with hemorrhage AND obstruction	3926261000006112	87756006	145494013
Acute gastrojejunal ulcer with hemorrhage AND perforation	3823411000006113	81387001	135007015
Acute gastrojejunal ulcer with perforation but without obstruction	3677391000006113	72395008	120279015
Chronic duodenal ulcer with hemorrhage	3954241000006119	89469000	148344012
Unspec gastrojejunal ulcer; unspec haemorrhage/perforation	81331000006114	16121001	27283019
Acute gastric ulcer with haemorrhage, with perforation AND with obstruction	3365071000006116	53337006	496625019
Duodenal ulcer with haemorrhage	2937121000006115	27281001	2937121000006115
Duodenal ulcer with haemorrhage	302534015	27281001	483490017
Gastrojejunal ulcer with hemorrhage but without obstruction	3320581000006112	50663005	84434015
Chronic gastrojejunal ulcer with haemorrhage, with perforation AND with obstruction	2882871000006112	24001002	481784011
Peptic ulcer with hemorrhage, with perforation AND with obstruction	2919721000006119	26221006	43926010
Chronic duodenal ulcer with perforation but without obstruction	3057061000006111	34602004	57742016
Perforated chronic gastric ulcer	484836012	31301004	484836012
Chronic gastrojejunal ulcer with haemorrhage AND perforation	494304013	45640006	494304013
Unspecified duodenal ulcer with haemorrhage and perforation	302537010	51868009	86362013
Unspecified peptic ulcer with perforation	302569018	13200003	22592012
Unspecified gastrojejunal ulcer with haemorrhage	302606019	16121001	27283019
Duodenal ulcer with hemorrhage	11902881000006118	27281001	45608011
Chronic peptic ulcer with haemorrhage AND perforation	498977018	61300005	498977018
Acute gastric ulcer with haemorrhage	508664015	89748001	508664015
Duodenal ulcer with hemorrhage but without obstruction	3072881000006114	35560008	59330018
Acute peptic ulcer with hemorrhage AND with perforation but without obstruction	3260961000006111	47064007	78447013
Acute peptic ulcer NOS	12721881000006113	591741000000105	1312001000000116
Chronic gastric ulcer with haemorrhage AND with obstruction	3895591000006110	85859006	506779011
DU - acute NOS	885881000006117	618291000000105	885881000006117
DU - acute + haemorrhage	885851000006113	12847006	885851000006113
DU - acute + perforation	885861000006110	61347001	885861000006110
DU - chronic + h'ge + perf.	885911000006117	36975000	885911000006117
PU - acute + haemorrhage	885941000006118	12274003	885941000006118
PU - acute + perforation	885951000006116	79118000	885951000006116
PU - acute + h'ge + perf.	885961000006119	111353003	885961000006119
PU - chronic + haemorrhage	885981000006112	49232000	885981000006112
Acute peptic ulcer with hemorrhage and perforation	4194481000006117	111353003	90139013
Gastrojejunal ulcer with haemorrhage but without obstruction	3320571000006114	50663005	495743015

Acute gastric ulcer with hemorrhage, with perforation AND with obstruction	3365081000006118	53337006	88690011
Gastrojejunal ulcer with haemorrhage AND with perforation but without obstruction	3926841000006112	87796008	507754015
Esophageal bleeding due to ulcerative esophagitis	7222991000006116	439442003	2794960012
Chronic duodenal ulcer with haemorrhage but without obstruction	3513251000006119	62341002	499253013
Acute duodenal ulcer with hemorrhage AND with perforation but without obstruction	3340401000006119	51847008	86331014
Peptic ulcer with hemorrhage	3542821000006112	64121000	106588013
Gastric ulcer with haemorrhage AND perforation but without obstruction	2531561000006115	2066005	480818019
Acute gastrojejunal ulcer with hemorrhage	3539941000006116	63954007	106302011
Chronic gastrojejunal ulcer with hemorrhage AND perforation	3236981000006112	45640006	76108010
GU - acute + perforation	885781000006110	19850005	885781000006110
GU - chronic + perforation	885821000006116	31301004	885821000006116
Acute duodenal ulcer with haemorrhage AND obstruction	3926251000006110	87756006	507729015
Acute gastric ulcer with perforation AND obstruction	3203661000006112	43694004	72854019
Unspecified peptic ulcer with haemorrhage and perforation	302571018	13200003	22592012
Unspecified gastrojejunal ulcer with perforation	302608018	16121001	27283019
Acute peptic ulcer with perforation	131281018	79118000	131281018
Chronic peptic ulcer with hemorrhage AND obstruction	3417381000006115	56461008	93897011
Acute gastric ulcer with hemorrhage AND perforation	3292411000006119	48974009	81590012
Chronic peptic ulcer with hemorrhage	3296621000006114	49232000	82009014
Acute duodenal ulcer with perforation but without obstruction	2858711000006118	22511002	37788015
Chronic gastrojejunal ulcer with hemorrhage but without perforation	3463971000006114	59356009	98589012
Acute peptic ulcer without hemorrhage, without perforation AND without obstruction	2547461000006113	3023008	6131012
Gastric ulcer with haemorrhage, with perforation AND with obstruction	2779151000006110	17593008	478382011
Chronic duodenal ulcer with haemorrhage AND with perforation but without obstruction	3819871000006111	81142005	504625010
Chronic gastric ulcer without haemorrhage, without perforation AND without obstruction	2523571000006111	1567007	477462013
Chronic gastric ulcer with haemorrhage AND with perforation but without obstruction	3708391000006114	74341002	502673018
Acute gastrojejunal ulcer with haemorrhage but without obstruction	3466621000006113	59515005	498459015
Duodenal ulcer with haemorrhage AND perforation	2879911000006114	23812009	481717012
Acute gastrojejunal ulcer with haemorrhage AND obstruction	3677531000006115	72408002	502115017
Acute gastrojejunal ulcer with haemorrhage AND with perforation but without obstruction	3584201000006119	66673003	500495015
Chronic peptic ulcer with hemorrhage but without obstruction	3825521000006119	81518000	135230017
Chronic peptic ulcer with haemorrhage AND with perforation but without obstruction	3405521000006119	55746001	497387014
Bleeding ulcer of oesophagus	3438281000006118	57748001	497976013
Gastric ulcer with hemorrhage, with perforation AND with obstruction	2779161000006112	17593008	29732013

Gastrojejunal ulcer with hemorrhage AND with perforation but without obstruction	3926851000006114	87796008	145560014
Acute peptic ulcer with perforation AND obstruction	3074551000006112	35681000	59518011
Chronic gastric ulcer with hemorrhage AND with perforation	3739191000006114	76181002	126521010
Chronic duodenal ulcer with hemorrhage, with perforation AND with obstruction	3902501000006117	86258000	143049014
Chronic duodenal ulcer with hemorrhage but without obstruction	3513261000006117	62341002	103623018
Acute gastric ulcer with haemorrhage AND obstruction	3254501000006115	46708007	494593012
Acute bleeding ulcer	3239141000006110	45771005	76330018
Acute gastric ulcer with haemorrhage but without obstruction	3645271000006113	70418001	501576019
Bleeding peptic ulcer	3542831000006110	64121000	1232530018
Gastrojejunal ulcer without hemorrhage AND without perforation	2543301000006112	2783007	5708013
Duodenal ulcer with hemorrhage AND obstruction	2791911000006117	18367003	31008016
Bleeding stress ulcer of stomach	5982901000006117	308882008	452259016
Gastric ulcer with hemorrhage	2752311000006118	15902003	26937011
Chronic gastric ulcer with hemorrhage, with perforation AND with obstruction	3894481000006112	85787009	142209015
Chronic duodenal ulcer with hemorrhage AND with perforation but without obstruction	3819881000006114	81142005	134627014
Peptic ulcer with haemorrhage AND with perforation but without obstruction	3970661000006113	90489006	509021016
Gastrojejunal ulcer without haemorrhage AND without perforation	2543291000006111	2783007	483682014
Chronic gastrojejunal ulcer with hemorrhage AND obstruction	3967191000006116	90257004	149607014
Chronic gastric ulcer with haemorrhage but without obstruction	3737501000006117	76078009	503190016
Acute duodenal ulcer with hemorrhage	2704011000006118	12847006	22034017
Acute peptic ulcer with haemorrhage but without obstruction	2852931000006111	22157005	481240010
Acute duodenal ulcer with hemorrhage but without obstruction	3585601000006117	66767006	110913018
Gastric ulcer with hemorrhage but without obstruction	2764921000006114	16694003	28271019
Acute duodenal ulcer with haemorrhage but without obstruction	3585591000006113	66767006	500520016
Acute peptic ulcer with perforation but without obstruction	3062531000006115	34921009	58276010
Peptic ulcer with hemorrhage AND perforation	3403141000006119	55617001	92455015
Peptic ulcer with haemorrhage AND obstruction	3547581000006111	64398008	499847018
Haemorrhagic ulcer of oesophagus	3438311000006116	57748001	497977016
Chronic gastric ulcer with hemorrhage AND with obstruction	3895601000006119	85859006	142332019
Giant duodenal ulcer	3619491000006117	68834009	114337018
Chronic peptic ulcer with perforation but without obstruction	3816621000006116	80953005	134291011
Bleeding duodenal ulcer	2937141000006110	27281001	1225269012
Bleeding gastric ulcer	2752321000006114	15902003	1221244018
Gastrojejunal ulcer with haemorrhage AND obstruction	3188241000006116	42698006	493421013

Chronic peptic ulcer with hemorrhage, with perforation AND with obstruction	3763481000006114	77661009	128895010
Acute gastrojejunal ulcer with hemorrhage AND obstruction	3677541000006113	72408002	120295015
Chronic duodenal ulcer with haemorrhage AND obstruction	3047841000006117	34021006	485720014
Acute gastric ulcer with hemorrhage but without obstruction	3645281000006111	70418001	116984014
Gastric ulcer with haemorrhage AND obstruction	3373651000006112	53877005	496788013
Chronic gastric ulcer with perforation but without obstruction	3084181000006117	36246001	60497010
Acute duodenal ulcer with perforation AND obstruction	3523071000006113	62936002	104600011
Gastric ulcer with hemorrhage AND obstruction	3373661000006114	53877005	89552015
Peptic ulcer with haemorrhage but without obstruction	3287291000006112	48658001	495168013
Chronic peptic ulcer with hemorrhage AND with perforation but without obstruction	3405531000006116	55746001	92690019
Peptic ulcer with haemorrhage	3542811000006116	64121000	499762011
Gastrojejunal ulcer with hemorrhage AND perforation	3542351000006113	64094003	106539013
Duodenal ulcer with haemorrhage, with perforation AND with obstruction	2696661000006114	12355008	472362019
Acute gastric ulcer NOS	12724281000006114	606391000000100	1341841000000112
Chronic duodenal ulcer with haemorrhage, with perforation AND with obstruction	3902491000006113	86258000	507005018
Duodenal ulcer with haemorrhage AND obstruction	2791901000006115	18367003	478566011
Duodenal ulcer with haemorrhage but without obstruction	3072871000006111	35560008	486208012
GU - acute + h'ge + perf.	885791000006113	48974009	885791000006113
GU - chronic + haemorrhage	885811000006112	57246001	885811000006112
GU - chronic + h'ge + perf.	885831000006118	76181002	885831000006118
DU - chronic + perforation	885901000006115	49916007	885901000006115
PU - chronic + h'ge + perf.	886001000006111	61300005	886001000006111
Acute gastric ulcer with haemorrhage AND with perforation but without obstruction	2770951000006116	17067009	478052011
Acute duodenal ulcer with haemorrhage, with perforation AND with obstruction	3176721000006111	41986000	493172010
Acute duodenal ulcer NOS	12730031000006113	618291000000105	1365771000000117
Acute peptic ulcer with hemorrhage but without obstruction	2852941000006118	22157005	37201012
Perforated ulcer	3981721000006111	91182001	151086014
Bleeding chronic gastric ulcer	1231704018	57246001	1231704018
Oesophageal bleeding due to ulcerative oesophagitis	7222981000006119	439442003	2790933013
Acute peptic ulcer with haemorrhage AND with perforation but without obstruction	3260951000006114	47064007	494746019
Bleeding oesophageal ulcer	3438321000006112	57748001	3027281014
Acute peptic ulcer with haemorrhage and perforation	361430016	111353003	361430016
Chronic duodenal ulcer with hemorrhage AND perforation	3095701000006110	36975000	61676011
Gastric ulcer with hemorrhage AND perforation	3513611000006117	62366003	103655018
Acute gastric ulcer with bleeding	3958891000006118	89748001	148790018

Chronic gastric ulcer with haemorrhage AND with perforation	554311000006114	76181002	503219010
Chronic gastric ulcer with perforation AND with obstruction	3400861000006115	55483002	92236018
Chronic gastrojejunal ulcer with perforation	5742016	2807004	5742016
Unspec peptic ulcer; unspec haemorrhage and/or perforation	81501000006111	13200003	22592012
Bleeding chronic duodenal ulcer	1235462014	89469000	1235462014
Peptic ulcer with hemorrhage but without obstruction	3287301000006113	48658001	81073011
Bleeding esophageal ulcer	3438331000006110	57748001	3027309010
Chronic gastrojejunal ulcer with hemorrhage	3521501000006114	62838000	104433010
Duodenal ulcer with perforation	302535019	88968005	147516013
Gastric ulcer with haemorrhage AND perforation	81311000006115	62366003	499256017
PU - acute NOS	885971000006114	591741000000105	885971000006114
Peptic ulcer with hemorrhage AND obstruction	3547591000006114	64398008	107045019
Bleeding ulcer of esophagus	3438291000006115	57748001	96029011
Acute gastrojejunal ulcer with hemorrhage, with perforation AND with obstruction	3453731000006116	58711008	97562017
Acute gastrojejunal ulcer with hemorrhage but without obstruction	3466631000006111	59515005	98850013
Chronic gastric ulcer with hemorrhage but without obstruction	3737511000006119	76078009	126363010
Acute gastric ulcer with hemorrhage AND obstruction	3254511000006117	46708007	77847018
Acute gastric ulcer with hemorrhage AND with perforation but without obstruction	2770961000006119	17067009	28881013
DU - acute NOS	990721000006111	196652006	990721000006111
Unspec duodenal ulcer; unspec haemorrhage and/or perforation	81301000006118	51868009	86362013
DU - chronic + haemorrhage	885891000006119	89469000	885891000006119
PU - chronic + perforation	885991000006110	3483000	885991000006110
Acute duodenal ulcer with haemorrhage	474430017	12847006	474430017
Chronic gastrojejunal ulcer with hemorrhage, with perforation AND with obstruction	2882881000006110	24001002	40293019
Chronic duodenal ulcer with perforation	83140013	49916007	83140013
Acute peptic ulcer with haemorrhage	470365013	12274003	470365013
Duodenal ulcer with perforation AND obstruction	3759241000006113	77410006	128499012
Chronic peptic ulcer with perforation AND obstruction	3440281000006115	57871005	96224010
Acute peptic ulcer with hemorrhage AND obstruction	3199311000006117	43406003	72407011
Duodenal ulcer with haemorrhage AND with perforation but without obstruction	2739541000006116	15115006	477132014
Barrett's ulcer of oesophagus	302387019	196609006	302387019
Acute gastrojejunal ulcer with hemorrhage AND with perforation but without obstruction	3584211000006116	66673003	110759011
Gastric ulcer with perforation	302489015	9829001	17164015
Chronic gastric ulcer with hemorrhage AND with perforation but without obstruction	3708401000006111	74341002	123454019
Gastrojejunal ulcer with haemorrhage, with perforation AND with obstruction	3389331000006116	54798007	497049011
Unspecified gastric ulcer with haemorrhage and perforation	302490012	62366003	499256017

Unspecified peptic ulcer with haemorrhage	302568014	13200003	22592012
Chronic gastric ulcer with haemorrhage, with perforation AND with obstruction	3894471000006114	85787009	506732011
Peptic ulcer with perforation AND obstruction	3281961000006115	48336009	80529013
Gastrojejunal ulcer with haemorrhage AND perforation	3542341000006111	64094003	499758017
Acute gastric ulcer	158227015	95529005	158227015
Gastrojejunal ulcer with haemorrhage	3868431000006116	84124004	505930017
Acute peptic ulcer with hemorrhage, with perforation AND with obstruction	2965441000006115	28945005	48463010
Gastrojejunal ulcer with hemorrhage AND obstruction	3188251000006119	42698006	71244011
Peptic ulcer with haemorrhage, with perforation AND with obstruction	2919711000006110	26221006	483152019
Gastric ulcer with perforation AND obstruction	3678661000006114	72486001	120408012
Duodenal ulcer with hemorrhage, with perforation AND with obstruction	2696671000006119	12355008	21266019
Acute gastrojejunal ulcer with haemorrhage AND perforation	504674011	81387001	504674011
Gastric ulcer with hemorrhage AND perforation but without obstruction	2531571000006110	2066005	4556011
Acute peptic ulcer without haemorrhage, without perforation AND without obstruction	2547451000006111	3023008	484524010
Acute gastric ulcer with hemorrhage	3958881000006116	89748001	148789010
Chronic gastrojejunal ulcer with hemorrhage AND with perforation but without obstruction	3251351000006110	46523000	77544017
Chronic gastrojejunal ulcer with perforation but without obstruction	3515751000006113	62477005	103838019
Acute duodenal ulcer with hemorrhage AND perforation	3912771000006119	86895006	144111018
Gastric ulcer with haemorrhage	302487018	15902003	477573015
Acute peptic ulcer with haemorrhage, with perforation AND with obstruction	2965431000006113	28945005	484017015
Chronic gastrojejunal ulcer with haemorrhage AND obstruction	3967181000006119	90257004	508918010
Peptic ulcer with haemorrhage AND perforation	3403131000006112	55617001	497320014
Chronic duodenal ulcer with hemorrhage AND obstruction	3047851000006115	34021006	56818016
Chronic peptic ulcer with haemorrhage but without obstruction	3825511000006110	81518000	504712012
Hemorrhagic ulcer of esophagus	3438301000006119	57748001	96030018
Chronic peptic ulcer with hemorrhage AND perforation	3496021000006116	61300005	101859019
Acute gastrojejunal ulcer with perforation AND obstruction	3674881000006110	72219001	120001014
Gastrojejunal ulcer with perforation AND obstruction	2846221000006119	21759003	36512017
Acute gastrojejunal ulcer with haemorrhage, with perforation AND with obstruction	3453721000006119	58711008	498229010
Acute gastric ulcer with haemorrhage AND perforation	495273013	48974009	495273013
Chronic gastric ulcer with haemorrhage	497833010	57246001	497833010
Acute duodenal ulcer with hemorrhage, with perforation AND with obstruction	3176731000006114	41986000	70069011
Chronic gastric ulcer with hemorrhage	3430281000006116	57246001	95214011
Bleeding ulcer	3393951000006114	55075001	91564012

Chronic gastric ulcer without hemorrhage, without perforation AND without obstruction	2523581000006114	1567007	3721015
Chronic peptic ulcer with haemorrhage, with perforation AND with obstruction	3763471000006111	77661009	503637019
Duodenal ulcer with hemorrhage AND perforation	2879921000006118	23812009	39977013
Chronic duodenal ulcer with perforation AND obstruction	3483491000006119	60551006	100598014
Duodenal ulcer with perforation but without obstruction	3914141000006116	86983005	144261016
Peptic ulcer with hemorrhage AND with perforation but without obstruction	3970671000006118	90489006	149975019
Chronic duodenal ulcer with haemorrhage AND perforation	486596015	36975000	486596015
Chronic peptic ulcer with haemorrhage	495344012	49232000	495344012
Acute gastrojejunal ulcer with haemorrhage	499719010	63954007	499719010
Acute duodenal ulcer with haemorrhage AND perforation	507303017	86895006	507303017
Gastric ulcer with haemorrhage but without obstruction	2764911000006118	16694003	477900010
Acute duodenal ulcer with haemorrhage AND with perforation but without obstruction	3340391000006116	51847008	496129014
Chronic gastrojejunal ulcer with haemorrhage	499386010	62838000	499386010
Chronic duodenal ulcer with haemorrhage	508524014	89469000	508524014
Bleeding acute gastric ulcer	1235493010	89748001	1235493010
Acute peptic ulcer with haemorrhage AND obstruction	3199301000006115	43406003	493597015
Chronic gastrojejunal ulcer with haemorrhage but without perforation	3463961000006119	59356009	498412010
Gastrojejunal ulcer with hemorrhage	3868441000006114	84124004	139505014
Unspec gastrojejunal ulcer with haemorrhage and perforation	81321000006111	16121001	27283019
Chronic peptic ulcer with haemorrhage AND obstruction	3417371000006118	56461008	497616018
GU - acute + haemorrhage	885771000006112	89748001	885771000006112
DU - acute + h'ge + perf.	885871000006115	86895006	885871000006115
Acute gastric ulcer with perforation	33419017	19850005	33419017
Chronic gastrojejunal ulcer with haemorrhage AND with perforation but without obstruction	3251341000006113	46523000	494529015
Acute duodenal ulcer with perforation	101926016	61347001	101926016
Acute gastrojejunal ulcer with perforation	110704012	66636001	110704012
Gastrojejunal ulcer with hemorrhage, with perforation AND with obstruction	3389341000006114	54798007	91074015

Table S18. Brain injury Aurum codes

Term	MedCode ID	SNOMED CT Concept ID	SNOMED CT Description ID
Traumatic subdural hemorrhage confined to region of tentorium	7834491000006110	722628000	3325389012
Open skull fracture with intracranial injury	4198071000006115	111617009	178860014
Subdural hemorrhage following injury without open intracranial wound AND with moderate loss of consciousness (1-24 hours)	3529801000006110	63323000	105259018
Brain injury without open intracranial wound AND with moderate loss of consciousness (1-24 hours)	3267211000006116	47450003	79110011
Subarachnoid haemorrhage following injury without open intracranial wound	2950231000006111	28048009	2950231000006111
Oth cerebral laceration/contusion no open intracranial wound	39851000006115	269144002	402921014
Cerebellar contusion with open intracranial wound AND loss of consciousness	2805231000006116	19233004	32422011
Intracranial injury with prolonged coma without open wound	455459010	311827003	455459010
Brain stem contusion with open intracranial wound AND moderate loss of consciousness (1-24 hours)	2867121000006117	23026001	38683019
Subarachnoid hemorrhage following injury with open intracranial wound	2583451000006111	5251007	9804011
Intracranial hematoma following injury	3111241000006113	37955001	63313013
Traumatic extradural hematoma with open intracranial wound	6053351000006112	315049003	459517019
Non-traumatic intracranial subdural haemorrhage	4777951000006113	195176009	2913186018
Traumatic cranial subdural hematoma	5459251000006112	262952002	391039018
Open traumatic subdural hemorrhage	4854201000006110	209956005	320770015
Other cerebral h'ge after injury + open intracranial wound	35791000006117	450418003	2915288012
Other cerebral h'ge after injury no open intracranial wound	35801000006116	450418003	2915288012
Oth cereb h'ge inj + open intracran wnd+LOC unspec duration	39721000006117	450418003	2915288012
Oth cereb h'ge inj no open intracran wnd+concussion unspec	39751000006114	450418003	2915288012
Subarach h'ge inj no open intracran wnd + concussion unspec	123311000006115	28048009	46957015
Subarachnoid h'ge inj + open intracran wnd+concussion unspec	123351000006119	5251007	9804011
Subarachnoid h'ge inj no open intracran wnd+<1hr loss consc	123391000006113	28048009	46957015
Subarachnoid h'ge inj no open intracran wound + 1-24hr LOC	123421000006117	28048009	46957015
Subdural h'ge inj + open intracranial wound + unspec consc	124091000006112	209956005	320770015
Extradural h'ge inj + open intracran wnd+LOC unspec duration	659991000006112	65189006	108332012
Extradural h'ge inj + open intracranial wnd + unspec consc	660001000006119	65189006	108332012
Extradural h'ge inj no open intracra wnd+LOC unspec duration	660031000006110	262949005	391026017
Extradural h'ge inj no open intracran wnd+1-24hr loss consc	660061000006118	43216008	72101019
Extradural h'ge inj no open intracranial wnd + no loss consc	660081000006111	43216008	72101019
Extradural h'ge inj no open intracranial wnd + unspec consc	660091000006114	262949005	391026017

Extradural h'ge inj no open intracranial wnd+<1hr loss consc	660101000006115	43216008	72101019
[V]Personal history of malignant neoplasm of brain	1227581011	266987004	397818015
Nontraumatic subarachnoid intracranial hemorrhage	5518101000006114	270907008	3449601019
Intracranial hemorrhage following injury without open intracranial wound	4198351000006110	111668007	178881014
Intracranial haemorrhage following injury without open intracranial wound AND with brief loss of consciousness (less than one hour)	2780311000006112	17667005	478394019
Biopsy of lesion of brain stem NEC	512541000006116	230842002	345857013
Brain cont + open intracranial wound + concussion unspec	524321000006119	269144002	402921014
Brain cont + open intracranial wound + unspec state consc	524351000006111	269144002	402921014
Closed #skull bse + intracranial injury + concussion unspec	559971000006115	111603000	178853015
Closed #skull bse + intracranial injury, LOC unspec duration	560011000006110	111603000	178853015
Closed #skull bse no intracranial inj,>24hr LOC not restored	560041000006114	27644009	46274013
Closed #skull bse no intracranial injury + concussion unspec	560051000006111	27644009	46274013
Closed #skull NOS + intracranial inj, <1hr loss of consc	560121000006118	371162008	1209864014
Closed #skull NOS + intracranial inj, >24hr LOC not restored	560131000006115	371162008	1209864014
Closed #skull NOS + intracranial inj, >24hrs LOC + recovery	560141000006113	371162008	1209864014
Closed #skull NOS no intracranial inj, >24hrs LOC + recovery	560211000006115	371162008	1209864014
Closed #skull NOS no intracranial inj, LOC unspec duration	560231000006114	371162008	1209864014
Closed #skull vlt + intracranial injury, LOC unspec duration	560311000006112	207687004	318117015
Closed #skull vlt + intracranial injury, unspec state consc	560331000006118	207687004	318117015
Closed #skull/face, mult + intracranial inj, no loss consc	560451000006118	5468008	10127011
Closed #skull/face,mult + intracran inj, concussion unspec	560541000006119	5468008	10127011
Cortex lacn + open intracranial wound + concussion unspec	597901000006114	59748008	99251014
Cortex lacn + open intracranial wound + unspec state consc	597921000006116	59748008	99251014
Crushing injury of head, part unspecified	602701000006116	283851005	422396012
Non-traumatic intracranial subdural hemorrhage	4777941000006111	195176009	2913068019
Brain damage - traumatic	12483101000006111	127295002	524451000006116
Subdural h'ge after injury	991811000006115	209987007	991811000006115
Closed traumatic subdural haemorrhage	320752018	209947002	320752018
Open traumatic subdural haemorrhage	320771016	209956005	320771016
Traumatic subdural haemorrhage	320835014	209987007	320835014
Subdural haemorrhage following injury	320836010	209987007	320836010
Traumatic subdural haematoma without open intracranial wound	459511018	315046005	459511018
Traumatic extradural haematoma with open intracranial wound	459518012	315049003	459518012
Subdural h'ge inj no open intracran wnd+>24hr LOC -restored	124121000006111	209947002	320752018

Subdural h'ge inj no open intracran wnd+1-24hr loss consc	124131000006114	209947002	320752018
Subdural h'ge inj no open intracranial wound+no loss consc	124191000006113	209947002	320752018
Subdural or cerebral haemorrhage due to birth trauma OS	124261000006112	240312009	359984010
Open traumatic subdural intracranial haemorrhage	4854211000006113	209956005	2912603017
Traumatic intracerebral haemorrhage	7378421000006118	450418003	2915664016
Contusion of cerebral cortex with open intracranial wound	3928361000006118	87888006	145701014
Extradural haemorrhage following injury with open intracranial wound AND prolonged loss of consciousness (more than 24 hours) AND return to pre-existing conscious level	3825541000006114	81520002	3825541000006114
Traumatic brain injury with no loss of consciousness	4396321000006112	127302008	590014
Epidural haematoma following injury	647051000006114	262949005	391028016
Evacuation of extradural haematoma	649541000006113	171713001	265968017
Clipping of aneurysm of cerebral artery	506344012	85028008	506344012
Open #skull/face, mult + intracranial inj, unspec consc	263941000006114	5468008	10127011
Open #skull bse + intracranial injury, unspec state of consc	264171000006119	111607004	178855010
Open #skull NOS + intracranial inj, <1hr loss of consc	264271000006114	371161001	1209863015
Open #skull NOS + intracranial inj, >24hrs LOC not restored	264281000006112	371161001	1209863015
Traumatic cerebral edema with open intracranial wound	6009911000006116	311826007	455457012
Traumatic extradural hematoma without open intracranial wound	6053331000006117	315048006	459516011
Brain stem laceration without open intracranial wound AND with concussion	2782671000006110	17819003	30102010
Traumatic brain injury with moderate loss of consciousness	4396301000006119	127300000	588013
Subdural haemorrhage following injury without open intracranial wound	4854181000006114	209947002	2915859018
Traumatic intracranial extradural hematoma	5459191000006110	262949005	2915790014
Cerebral haemorrhage following injury NOS	320852013	450418003	2916363013
Other cerebral haemorrhage following injury	320853015	450418003	2916363013
Brain stem laceration without open intracranial wound AND with prolonged loss of consciousness (more than 24 hours) AND return to pre-existing conscious level	3649561000006119	70686002	117408014
Middle meningeal haemmorhage following injury	702641000006119	30400005	484576014
Intracran inj NOS no open intracran wnd+>24hr LOC -restored	746611000006114	127296001	584010
Intracranial inj NOS + open intracran wnd+>24hr LOC+recovery	769311000006117	127296001	584010
Intracranial inj NOS + open intracran wnd+concussion unspec	769321000006113	127296001	584010
Intracranial inj NOS + open intracranial wnd+<1hr loss consc	769351000006116	127296001	584010
Intracranial inj NOS + open intracranial wound+no loss consc	769361000006119	127296001	584010
Extradural hemorrhage following injury with open intracranial wound, with no loss of consciousness	4854301000006119	209978003	320818010
Intracerebral injury NOS	990131000006113	566951000000103	990131000006113

Traumatic intracranial haemorrhage	12757921000006111	82894007	505323015
Cerebral haemorrhage following injury	12757911000006115	82894007	505322013
Subarachnoid hemorrhage following injury without open intracranial wound	11923791000006114	28048009	46957015
Craniectomy with treatment of penetrating wound of brain	2546441000006111	2968008	6037013
Subarachnoid hemorrhage following injury without open intracranial wound AND with concussion	2567831000006110	4332009	8298019
Traumatic extradural haematoma	391028016	262949005	391028016
Traumatic cranial subarachnoid hemorrhage	5459281000006116	262955000	391046010
Closed skull fracture with intracranial injury	4198041000006111	111613008	4198041000006111
Subarachnoid hemorrhage following injury with open intracranial wound AND concussion	3284991000006114	48518008	80833014
Traumatic intracranial subdural haematoma with brief loss of consciousness	6348441000006113	371050006	1207227019
Extradural hemorrhage following injury with open intracranial wound AND prolonged loss of consciousness (more than 24 hours) AND return to pre-existing conscious level	11924651000006110	81520002	135232013
Ligation of aneurysm of cerebral artery NEC	271559016	230899005	345929019
Subarachnoid haemorrhage following injury without open intracranial wound AND with no loss of consciousness	3074431000006116	35672006	486236019
Intracranial hemorrhage following injury	12757871000006118	82894007	137497019
Lacerating brain injury	3783541000006116	78914008	503949015
Traumatic subdural haematoma	5459261000006114	262952002	2913152010
Cerebellar contusion without open intracranial wound	3438141000006115	57739006	96013015
Intracranial hemorrhage following injury without intracranial wound AND with moderate loss of consciousness (1-24 hours)	3589031000006116	66976009	111248010
Intracranial haemorrhage following injury with loss of consciousness	4396431000006115	127308007	474109017
Brain stem contusion without open intracranial wound AND with concussion	3788431000006116	79220008	131450012
Subdural haemorrhage following injury without open intracranial wound AND with moderate loss of consciousness (1-24 hours)	3529791000006114	63323000	499552019
Non-traumatic subdural hemorrhage	4777931000006118	195176009	2469434017
Nontraumatic intracranial haemorrhage	3533578018	738779002	3533578018
Hind brain contusion with open intracranial wound, with more than 24 hours loss of consciousness without return to pre-existing conscious level	823341000006110	209887008	320658014
Hind brain contusion with open intracranial wound, with 1-24 hours loss of consciousness	823351000006112	209885000	320656013
Hind brain contusion with open intracranial wound, with more than 24 hours loss of consciousness and return to pre-existing conscious level	823381000006116	209886004	320657016
Chronic non-traumatic intracranial subdural haemorrhage	7497191000006114	609382000	2959633012
Subarachnoid hemorrhage following injury with open intracranial wound AND moderate loss of consciousness (1-24 hours)	11903611000006117	87253004	144684019
Extradural hemorrhage following injury without open intracranial wound AND with concussion	3760981000006114	77498000	128652013
Brain stem laceration without open intracranial wound AND with brief loss of consciousness (less than one hour)	3467421000006112	59561005	98926012

Traumatic brain injury with prolonged loss of consciousness	4396311000006116	127301001	589017
Nontraumatic hemorrhage of subarachnoid space from intracranial artery	3511284017	291371000119100	3511284017
Brain injury with open intracranial wound AND brief loss of consciousness (less than one hour)	2951821000006119	28156009	47135017
Intracranial haemorrhage following injury	7378271000006116	450410005	2915426010
Nontraumatic extradural intracranial hemorrhage	6593141000006116	397809001	2915638010
Extradural hemorrhage following injury with open intracranial wound AND moderate loss of consciousness (1-24 hours)	3468981000006117	59648004	99099016
Intracranial hemorrhage following injury with intracranial wound AND moderate loss of consciousness (1-24 hours)	3055551000006113	34501004	57585011
Non-traumatic extradural intracranial hematoma	5656951000006110	281865000	419997012
Cerebellar laceration with open intracranial wound AND prolonged loss of consciousness (more than 24 hours) AND return to pre-existing conscious level	3366571000006117	53429002	88842018
Traumatic haemorrhage of subdural space of infratentorial region	7834471000006114	722628000	3325385018
Traumatic cerebral oedema without open intracranial wound	455455016	311825006	455455016
Excision of aneurysm of cerebral artery	271556011	175385008	271556011
Intracranial haemorrhage following injury with open intracranial wound AND concussion	3782881000006115	78879009	503942012
Craniotomy and evacuation of traumatic haematoma	7330521000006119	446816008	2883668012
Intracranial hemorrhage following injury without open intracranial wound AND with prolonged loss of consciousness (more than 24 hours) without return to pre-existing level	2521231000006111	1430001	3487015
Brain stem laceration with open intracranial wound AND moderate loss of consciousness (1-24 hours)	3160801000006118	41025001	68439012
Brain stem laceration without open intracranial wound AND with no loss of consciousness	3777371000006118	78525006	130297019
Subarachnoid haemorrhage following injury without open intracranial wound AND with loss of consciousness	3629571000006112	69458009	501279013
Multiple open fractures of skull AND/OR face with cerebral laceration AND/OR contusion	3930061000006116	88008005	145885012
Cerebellar contusion with open intracranial wound AND brief loss of consciousness (less than one hour)	3683771000006112	72797002	120920014
Intracranial hemorrhage following injury with open intracranial wound and prolonged loss of consciousness (more than 24 hours) without return to pre-existing level	4198371000006117	111671004	135096017
Cerebellar laceration with open intracranial wound	2997431000006117	30858001	51649013
Intracranial hemorrhage co-occurrent and due to complex wound of head	7840901000006116	723141009	3335258015
Brain stem contusion without open intracranial wound AND with loss of consciousness	2979771000006112	29807001	49879010
Traumatic injury of blood vessel of head	7317871000006113	446029009	2883183018
Extradural haemorrhage following injury without open intracranial wound AND with concussion	3760971000006111	77498000	503596015
Intracranial haemorrhage following injury without open intracranial wound	4198341000006113	111668007	189842012
Secondary traumatic haemorrhage of brainstem	7840851000006114	723139008	3334700013

Extradural haemorrhage following injury with open intracranial wound	320814012	65189006	500043017
Subarachnoid haemorrhage following injury with open intracranial wound AND prolonged loss of consciousness (more than 24 hours) without return to pre-existing conscious level	123291000006119	69178005	501220014
Subarachnoid haemorrhage following injury with open intracranial wound AND moderate loss of consciousness (1-24 hours)	123341000006116	87253004	507490011
Subdural haemorrhage following injury with open intracranial wound AND moderate loss of consciousness (1-24 hours)	124071000006111	90165008	508869011
Subdural haemorrhage following injury without open intracranial wound AND with loss of consciousness	3049841000006112	34135005	485760010
Subdural haemorrhage following injury with open intracranial wound AND brief loss of consciousness (less than one hour)	124101000006118	29635000	484345019
Subdural haemorrhage following injury with open intracranial wound, with no loss of consciousness	124111000006115	209958006	320775013
Extradural haemorrhage following injury without open intracranial wound AND with no loss of consciousness	4198421000006112	111679002	364069017
Brain stem laceration without open intracranial wound AND with moderate loss of consciousness (1-24 hours)	2794111000006117	18531006	31261015
Nontraumatic subarachnoid haemorrhage with brain compression	8044541000006113	141091000119105	3043206010
Extradural haemorrhage following injury without open intracranial wound AND with prolonged loss of consciousness (more than 24 hours) AND return to pre-existing conscious level	3879081000006114	84792001	506232012
Intracranial hemorrhage following injury with brief loss of consciousness	4396461000006112	127309004	597012
Subdural hemorrhage following injury without open intracranial wound AND with prolonged loss of consciousness (more than 24 hours) AND return to pre-existing conscious level	3920061000006118	87345009	144822016
Subacute non-traumatic intracranial subdural hemorrhage	8089881000006112	291591000119107	2959779014
Intracranial hemorrhage following injury without open intracranial wound AND with prolonged loss of consciousness (more than 24 hours) AND return to pre-existing conscious level	3776601000006116	78477003	130220011
Brain stem laceration with open intracranial wound AND prolonged loss of consciousness (more than 24 hours) without return to pre-existing conscious level	3547841000006115	64413001	107072010
Open fracture of base of skull with cerebral laceration AND contusion	2989141000006113	30371007	50833015
Subarachnoid hemorrhage following injury without open intracranial wound AND with no loss of consciousness	3074441000006114	35672006	59500013
Subarachnoid hemorrhage following injury without open intracranial wound AND with prolonged loss of consciousness (more than 24 hours) AND return to pre-existing conscious level	3942121000006114	88747000	147146018
Brain injury with open intracranial wound AND no loss of consciousness	2705081000006112	12912004	22137010
Subarachnoid haemorrhage following injury without open intracranial wound AND with moderate loss of consciousness (1-24 hours)	4198381000006119	111673001	364055017

Cerebellar laceration without open intracranial wound	2983451000006116	30031007	50251013
Subdural haemorrhage following injury without open intracranial wound AND with no loss of consciousness	3145681000006112	40135004	492453011
Extradural hemorrhage following injury with open intracranial wound AND loss of consciousness	4198451000006115	111681000	189006017
Traumatic intracranial subdural hematoma with brief loss of consciousness	6348451000006110	371050006	1208039019
Cranial puncture	5559151000006110	273989008	409716019
Compression of brain co-occurrent and due to nontraumatic subarachnoid haemorrhage	8044551000006110	141091000119105	3042983016
Subdural hemorrhage following injury without open intracranial wound AND with brief loss of consciousness (less than one hour)	2919471000006113	26205001	43899010
Subarachnoid haemorrhage following injury without open intracranial wound AND with brief loss of consciousness (less than one hour)	3914851000006114	87020000	507372011
Focal haemorrhagic contusion of cerebrum	7834261000006115	722615007	3332903015
Subarachnoid haemorrhage following injury with open intracranial wound AND moderate loss of consciousness (1-24 hours)	3918591000006111	87253004	3918591000006111
Cerebellar laceration with open intracranial wound AND prolonged loss of consciousness (more than 24 hours) without return to pre-existing conscious level	3546541000006115	64337000	106943019
Subarachnoid haemorrhage following injury without open intracranial wound AND with prolonged loss of consciousness (more than 24 hours) AND return to pre-existing conscious level	3942111000006118	88747000	508175017
Injury of head with otorrhagia	4063221000006112	95848000	158756016
Open fracture of base of skull with intracranial injury, with less than 1 hour loss of consciousness	264121000006115	207746007	318190017
Intracranial hemorrhage following injury with open intracranial wound AND brief loss of consciousness (less than one hour)	2795161000006118	18605003	31370015
Traumatic brain injury with prolonged loss of consciousness (more than 24 hours) and return to pre-existing conscious level	7380341000006114	450551009	2916174013
Subdural haemorrhage following injury without open intracranial wound AND with prolonged loss of consciousness (more than 24 hours) AND return to pre-existing conscious level	3920051000006115	87345009	507531010
Compression of brain co-occurrent and due to nontraumatic subarachnoid hemorrhage	8044561000006112	141091000119105	3043035013
Traumatic brain injury with loss of consciousness one hour or more	7380591000006114	450569000	2915718018
Intracranial hemorrhage following injury without open intracranial wound AND with brief loss of consciousness (less than one hour)	2780321000006116	17667005	29855017
Intracranial hemorrhage following injury with loss of consciousness	4396441000006113	127308007	596015
Traumatic subdural hematoma without open intracranial wound	6053291000006112	315046005	459512013
Intracranial hemorrhage following injury with open intracranial wound	3765301000006113	77768006	129069015
Subarachnoid hemorrhage following injury without open intracranial wound AND with brief loss of consciousness (less than one hour)	3914861000006111	87020000	144313014
Intracranial hemorrhage following injury without open intracranial wound AND with concussion	3781111000006112	78757009	130692016

Brain injury with open intracranial wound	2952391000006117	28188001	47189015
Cerebellar contusion with open intracranial wound AND concussion	3900201000006118	86125000	142812014
Traumatic brain injury with prolonged loss of consciousness (more than 24 hours) without return to pre-existing conscious level	7380351000006111	450552002	2915596018
Non-traumatic spinal subdural haematoma	6028571000006117	313304007	457177012
Extradural hemorrhage following injury without open intracranial wound AND with brief loss of consciousness (less than one hour)	3152821000006115	40549004	67610019
Brain damage - traumatic	11923371000006114	275272006	411234012
Subdural hemorrhage following injury without open intracranial wound AND with concussion	2606541000006112	6666006	12042015
Head injury with hemorrhage from nose	4395861000006117	127276009	205229010
Intracranial haemorrhage following injury with prolonged loss of consciousness without return to pre-existing conscious level	4396511000006110	127312001	474113012
Non-traumatic intracranial subdural hematoma	5656931000006115	281864001	419995016
Focal traumatic haematoma of brainstem	7834421000006113	722624003	3334493017
Nontraumatic subdural haematoma with brain compression	8044591000006116	141151000119101	2967571019
Subacute non-traumatic intracranial subdural haemorrhage	8089861000006119	291591000119107	2959517016
Subarachnoid haemorrhage following injury with open intracranial wound	320735017	5251007	496337012
Traumatic subdural hematoma	391035012	262952002	2912898019
Lobotomy of brain	5559171000006117	273991000	409719014
Intracranial haemorrhage following injury with open intracranial wound AND brief loss of consciousness (less than one hour)	2795151000006115	18605003	478724012
Brain stem laceration with open intracranial wound	2700331000006119	12589008	21640014
Traumatic subdural intracranial haemorrhage	4854361000006118	209987007	2912530015
Subarachnoid hemorrhage following injury with open intracranial wound AND brief loss of consciousness (less than one hour)	3468691000006111	59633005	99064015
Closed traumatic subdural hemorrhage	4854141000006115	209947002	320753011
Subdural haemorrhage following injury without open intracranial wound AND with concussion	2606531000006119	6666006	500492017
Extradural haemorrhage following injury without open intracranial wound AND with prolonged loss of consciousness (more than 24 hours) without return to pre-existing conscious level	2768441000006115	16907002	477986019
Acute nontraumatic subdural haemorrhage	3515795019	291581000119109	3515795019
Intracran inj NOS + open intracran wnd+LOC unspec duration	746601000006111	127296001	584010
Intracranial inj NOS no open intracran wnd+<1hr loss consc	768831000006114	127296001	584010
Intracranial inj NOS no open intracran wnd+1-24hr loss consc	768851000006119	127296001	584010
Subdural hemorrhage following injury without open intracranial wound AND with loss of consciousness	3049851000006114	34135005	57003014
Closed fracture of skull NOS with intracranial injury	12484051000006114	371162008	563781000006112
Subdural hemorrhage following injury with open intracranial wound AND prolonged loss of consciousness (more than 24 hours) AND return to pre-existing conscious level	3357881000006110	52902005	88030010

Focal traumatic hematoma of brainstem	7834431000006111	722624003	3332924013
Nontraumatic subdural hematoma with brain compression	8044601000006112	141151000119101	2967694013
Open fracture of vault of skull with intracranial injury, with 1-24 hours loss of consciousness	52941000006118	207709008	318139018
Cerebellar contusion without open intracranial wound AND with loss of consciousness	2772451000006114	17169009	29035017
Cerebral hemorrhage following injury	12757891000006117	82894007	505319011
Traumatic intracranial hemorrhage	12757901000006118	82894007	505320017
Subdural hemorrhage following injury without open intracranial wound AND with no loss of consciousness	3145691000006110	40135004	63714017
Subarachnoid haemorrhage following injury with open intracranial wound AND loss of consciousness	3727871000006112	75507000	503040018
Subdural hemorrhage following injury with open intracranial wound AND concussion	3197131000006115	43262000	72179018
Extradural hemorrhage following injury without open intracranial wound AND with prolonged loss of consciousness (more than 24 hours) AND return to pre-existing conscious level	3879091000006112	84792001	140578018
Brain injury with open intracranial wound AND moderate loss of consciousness (1-24 hours)	3898191000006111	86010003	142597011
Non-traumatic extradural intracranial haematoma	5656941000006113	281865000	419996015
Traumatic haemorrhage of brainstem	7834611000006115	722632006	3332940011
Cerebellar contusion with open intracranial wound AND prolonged loss of consciousness (more than 24 hours) without return to pre-existing conscious level	2863721000006113	22825007	38315013
Traumatic spinal subdural hematoma	5456261000006117	262721007	390720010
Cortex contusion with open intracranial wound	3928351000006115	87888006	145700010
Traumatic cranial subdural haematoma	5459221000006115	262952002	391036013
Subarachnoid haemorrhage following injury with open intracranial wound AND brief loss of consciousness (less than one hour)	123371000006112	59633005	498500014
Subarachnoid haemorrhage following injury with open intracranial wound, with no loss of consciousness	123381000006110	209940000	320739011
Subdural haemorrhage following injury with open intracranial wound AND prolonged loss of consciousness (more than 24 hours) AND return to pre-existing conscious level	124061000006116	52902005	496480019
Brain stem contusion with open intracranial wound AND brief loss of consciousness (less than one hour)	3426401000006114	57012007	94810019
Subarachnoid hemorrhage following injury without open intracranial wound AND with prolonged loss of consciousness (more than 24 hours) AND without return to pre-existing conscious level	2822141000006116	20276007	34108017
Subdural hemorrhage following injury with open intracranial wound, with no loss of consciousness	4854261000006111	209958006	320774012
Cerebellar laceration with open intracranial wound AND loss of consciousness	3880781000006111	84900008	140766015
Closed fracture of vault of skull with intracranial injury, with no loss of consciousness	560321000006116	207689001	318119017
Brain stem contusion with open intracranial wound AND no loss of consciousness	3358011000006113	52913008	88047011

Extradural haemorrhage following injury without open intracranial wound AND with loss of consciousness	3965881000006116	90178008	508877010
Oth cereb h'ge inj no open intracran wnd+LOC unspec duration	39761000006111	450418003	2915288012
Subarachnoid h'ge inj no open intracran wnd+no loss consc	123411000006113	28048009	46957015
Traumatic hemorrhage of brainstem	7834621000006111	722632006	3332939014
Subarachnoid haemorrhage following injury without open intracranial wound AND with prolonged loss of consciousness (more than 24 hours) AND without return to pre-existing conscious level	2822131000006114	20276007	480562018
Intracranial hemorrhage following injury without open intracranial wound AND with loss of consciousness	2793491000006117	18485009	31186017
Cerebellar laceration with open intracranial wound AND no loss of consciousness	2728701000006119	14434006	24535010
Multiple traumatic hemorrhages of brain tissue	7834661000006117	722633001	3332944019
Intracranial hemorrhage following injury with open intracranial wound AND loss of consciousness	2710751000006112	13289004	22738011
Open fracture of base of skull with intracranial injury	259601000006113	111607004	178855010
Open fracture of vault of skull with intracranial injury, with less than 1 hour loss of consciousness	264431000006110	207708000	318138014
Open fracture of base of skull with intracranial injury, with more than 24 hours loss of consciousness without return to pre-existing conscious level	265451000006116	207749000	318193015
Head injury with haemorrhage from ear	4063241000006117	95848000	512328010
Intracranial hemorrhage following injury with prolonged loss of consciousness AND return to pre-existing conscious level	4396501000006112	127311008	599010
Brain stem laceration without open intracranial wound	2832491000006115	20899000	35146017
Extradural haemorrhage following injury without open intracranial wound AND with brief loss of consciousness (less than one hour)	3152811000006111	40549004	492668015
Brain injury with open intracranial wound AND prolonged loss of consciousness (more than 24 hours) AND return to pre-existing conscious level	2948171000006119	27923006	46743012
Closed fracture of base of skull with cerebral laceration AND/OR contusion	3988021000006116	91589002	151735014
Nontraumatic haemorrhage of subarachnoid space from intracranial artery	3511285016	291371000119100	3511285016
Intracranial haemorrhage following injury with open intracranial wound AND no loss of consciousness	2624481000006115	7819003	503779018
Cerebellar laceration without open intracranial wound AND with prolonged loss of consciousness (more than 24 hours) AND return to pre-existing conscious level	3981471000006117	91168001	151064013
Subdural hemorrhage following injury with open intracranial wound AND loss of consciousness	3237321000006110	45659008	76138016
Traumatic cerebral hemorrhage	7378441000006113	450418003	2916150010
Subarachnoid hemorrhage following injury with open intracranial wound AND prolonged loss of consciousness (more than 24 hours) without return to pre-existing conscious level	11903601000006115	69178005	114903015
Traumatic intracerebral haemorrhage	12757941000006116	82894007	2475153011

Subarachnoid hemorrhage following injury with open intracranial wound, with no loss of consciousness	4854121000006110	209940000	320738015
Intracranial nontraumatic haemorrhage of foetus and newborn	394431000006118	206417009	2791314016
Brain contusion with open intracranial wound, with more than 1 hour loss of consciousness	524291000006111	209921006	320693019
Brain contusion with open intracranial wound, with 1-24 hours loss of consciousness	524311000006110	209922004	320694013
Brain contusion with open intracranial wound, with no loss of consciousness	524341000006114	209920007	320692012
Biopsy of lesion of cranial nerve	512591000006113	171687004	265940016
Brain cont + open intracranial wound + LOC unspec duration	524331000006116	269144002	402921014
Closed #skull bse no intracranial injury, <1hr loss of consc	560061000006113	27644009	46274013
Closed #skull vlt with intracranial injury+concussion unspec	560421000006110	207687004	318117015
Cortex cont + open intracranial wnd + LOC unspec duration	597681000006113	209843004	320611018
Cortex cont + open intracranial wound + unspec state consc	597731000006115	87888006	145700010
Cortex lacn + open intracranial wnd + LOC unspec duration	597861000006119	59748008	99251014
Cortex lacn no open intracranial wnd + LOC unspec duration	597951000006113	78914008	130942015
Late effect of injury to blood vessel of head, neck and extremities	750631000006119	210950000	322021015
Injuries of brain and cranial nerves with injuries of nerves and spinal cord at neck level	777751000006119	213383004	325280017
Intracranial haemorrhage following injury with brief loss of consciousness	4396451000006110	127309004	474110010
Intracranial hemorrhage following injury with open intracranial wound AND concussion	3782891000006117	78879009	130868014
Traction injury of cranial nerve	5459341000006115	262960001	391054012
Extradural hemorrhage following injury without open intracranial wound AND with prolonged loss of consciousness (more than 24 hours) without return to pre-existing conscious level	2768451000006118	16907002	28621013
Nontraumatic subarachnoid hemorrhage with brain compression	8044571000006117	141091000119105	3043074018
Closed skull fracture with cerebral laceration AND/OR contusion	3940751000006119	88651008	146986016
Cerebellar laceration without open intracranial wound AND with no loss of consciousness	3887091000006113	85314006	141420019
Craniotomy and removal of hematoma from extradural space	7312961000006111	445746008	2884424011
Closed fracture of vault of skull with intracranial injury, with less than 1 hour loss of consciousness	560281000006110	207690005	318120011
Cortex contusion with open intracranial wound, with more than 24 hours loss of consciousness and return to pre-existing conscious level	597661000006115	209849000	320617019
Cortex laceration with open intracranial wound, with more than 24 hours loss of consciousness and return to pre-existing conscious level	597881000006112	209867007	320635011
Cortex laceration with open intracranial wound, with 1-24 hours loss of consciousness	597891000006110	209866003	320634010
Cortex laceration with open intracranial wound, with no loss of consciousness	597911000006112	209864000	320632014
Nontraumatic extradural haemorrhage	660111000006117	397809001	1773153015
Intracranial haemorrhage following injury without open intracranial wound AND with prolonged loss	2521221000006113	1430001	476216010

of consciousness (more than 24 hours) without return to pre-existing level			
Traumatic spinal extradural haematoma	5456211000006115	262720008	390718012
Open #skull vlt with intracranial injury + concussion unspec	263881000006110	207705002	318135012
Intracranial hemorrhage following injury with moderate loss of consciousness	4396481000006119	127310009	598019
Traumatic hemorrhage of subdural space of infratentorial region	7834481000006112	722628000	3325383013
Intracranial haemorrhage co-occurrent and due to complex wound of head	7840891000006115	723141009	3335259011
Traumatic spinal subdural haematoma	5456251000006119	262721007	390721014
Brain stem laceration with open intracranial wound AND concussion	3164101000006115	41222005	68763015
Cerebellar laceration without open intracranial wound AND with brief loss of consciousness (less than one hour)	3059661000006113	34751006	58002019
Extradural hemorrhage following injury without open intracranial wound AND with moderate loss of consciousness (1-24 hours)	3523681000006119	62973004	104678015
Subdural haemorrhage following injury without open intracranial wound AND with prolonged loss of consciousness (more than 24 hours) without return to pre-existing conscious level	3652481000006119	70861009	501707010
Primary traumatic haemorrhage of brainstem	7840831000006119	723138000	3334697014
Oth cerebral h'ge inj + open intracranial wnd+no loss consc	39801000006119	450418003	2915288012
Subarach h'ge inj no open intracran wnd+>24hrs LOC-restored	123321000006111	28048009	46957015
Subarachnoid h'ge inj + open intracran wound + unspec consc	123361000006117	5251007	9804011
Extradural h'ge inj no open intracran wnd+>24hr LOC+recovery	660041000006117	43216008	72101019
Subdural haemorrhage - nontraumatic	300294013	195176009	300294013
Traumatic intracerebral hemorrhage	12757951000006119	82894007	2475363012
Traumatic intracranial subdural haematoma	5459231000006117	262952002	391037016
Extradural hemorrhage following injury without open intracranial wound AND with no loss of consciousness	4198431000006110	111679002	199709019
Cerebellar laceration without open intracranial wound AND with loss of consciousness	3969771000006113	90429009	149873010
Subarachnoid h'ge inj no open intracran wound + unspec consc	123431000006119	28048009	46957015
Open fracture of base of skull with intracranial injury, with more than 24 hours loss of consciousness and return to pre-existing conscious level	264131000006117	207748008	318192013
Subdural haemorrhage following injury without open intracranial wound AND with brief loss of consciousness (less than one hour)	2919461000006118	26205001	483148019
Open #skull/face, mult + intracran inj + concussion, unspec	263891000006113	5468008	10127011
Open #skull NOS + intracranial inj, LOC unspec duration	264301000006111	371161001	1209863015
Traumatic injury of third cranial nerve	3370341000006114	53675004	198460011
Closed #skull bse no intracranial injury, 1-24hr loss consc	560081000006115	27644009	46274013
Closed #skull NOS + intracranial inj, 1-24hrs loss of consc	560151000006110	371162008	1209864014

Closed #skull NOS no intracranial inj, <1hr loss of consc	560201000006118	371162008	1209864014
Closed #skull NOS no intracranial inj, unspec state of consc	560251000006119	371162008	1209864014
Closed #skull/face,mult + intracran inj, LOC unspec duration	560551000006117	5468008	10127011
Cortex lacn no open intracranial wnd + 1-24hr loss of consc	597941000006111	78914008	130942015
Mult #skull/face+other bones, closed + intracranial injury	696141000006119	5468008	10127011
Hind brain cont + open intracranial wnd +LOC unspec duration	823391000006118	209881009	320652010
Subdural haemorrhage following injury with open intracranial wound AND concussion	3197121000006118	43262000	493561012
Closed fracture of base of skull with intracranial injury, with less than 1 hour loss of consciousness	559981000006117	207728001	318172018
Closed fracture of vault of skull with intracranial injury, with more than 24 hours loss of consciousness without return to pre-existing conscious level	560271000006112	207693007	318123013
Cortex laceration with open intracranial wound, with less than 1 hour loss of consciousness	597871000006114	209865004	320633016
Intracranial haemorrhage following injury	12716261000006116	82894007	505321018
Nontraumatic extradural intracranial haemorrhage	6593151000006119	397809001	2916444013
Intracran inj NOS + open intracran wnd+>24hr LOC -restored	746591000006115	127296001	584010
Intracranial inj NOS no open intracran wnd+>24hr LOC+recover	768841000006116	127296001	584010
Intracranial injury, excluding those with skull fracture NOS	768961000006113	54355006	90339018
Intracranial inj NOS + open intracranial wnd + 1-24hr LOC	769331000006111	127296001	584010
Intracranial inj NOS + open intracranial wnd + unspec consc	769341000006118	127296001	584010
Injury of blood vessels of head, NEC	776021000006111	85564003	141827011
Focal traumatic haemorrhage of brainstem	7834401000006115	722623009	3332922012
Extradural haemorrhage following injury with open intracranial wound AND prolonged loss of consciousness (more than 24 hours) without return to pre-existing conscious level	659951000006118	86182004	506960017
Extradural haemorrhage following injury with open intracranial wound AND brief loss of consciousness (less than one hour)	660011000006116	73308006	502406019
Secondary hemorrhage of brainstem due to traumatic injury	7840871000006116	723139008	3334701012
Cerebellar contusion with open intracranial wound AND moderate loss of consciousness (1-24 hours)	3427941000006119	57099002	94960012
Subarach h'ge inj + open intracran wnd+LOC unspec duration	123301000006118	5251007	9804011
Subarachnoid h'ge inj no open intracran wnd+>24 LOC+recovery	123401000006110	28048009	46957015
Subdural h'ge inj + open intracran wnd+LOC unspec duration	124051000006118	209956005	320770015
Extradural h'ge inj + open intracran wnd+concussion unspec	659981000006114	65189006	108332012
Extradural h'ge inj no open intracran wnd+>24hr LOC-restored	660051000006115	43216008	72101019
Sequela of traumatic intracranial hemorrhage	8031561000006113	100581000119102	2967672017
Closed #skull bse no intracranial injury, >24hr LOC+recovery	560071000006118	27644009	46274013

Closed #skull NOS + intracranial inj, no loss of consc	560171000006117	371162008	1209864014
Closed #skull NOS no intracranial inj, no loss of consc	560241000006116	371162008	1209864014
Closed #skull NOS no intracranial inj,>24hr LOC not restored	560261000006117	371162008	1209864014
Extradural haemorrhage after injury	5459181000006112	262949005	391029012
Extradural hemorrhage following injury with open intracranial wound AND concussion	2951811000006110	28155008	47133012
Head injury with hemorrhage from ear	4063231000006110	95848000	158757013
Subarachnoid hemorrhage following injury with open intracranial wound AND prolonged loss of consciousness (more than 24 hours) AND return to pre-existing conscious level	11903591000006111	73439007	121954015
Traumatic subdural hematoma with open intracranial wound	6053311000006111	315047001	459513015
Traumatic subdural intracranial hemorrhage	4854351000006115	209987007	2912507014
Traumatic subdural hemorrhage	4854331000006110	209987007	320837018
Fracture of frontonasoethmoidal complex with increased intercanthal distance	5461381000006115	263154009	391284014
Traumatic brain injury with loss of consciousness	4396281000006118	127298000	586012
Subdural h'ge inj no open intracran wnd+LOC unspec duration	124141000006116	209947002	320752018
Sequelae of other nontraumatic intracranial haemorrhage	149551000006111	363302008	482447013
Open #skull vlt + intracranial injury, unspec state of consc	263791000006119	207705002	318135012
Open #skull/face, mult + intracranial inj, no loss consc	263931000006116	5468008	10127011
Open #skull NOS + intracranial inj, 1-24hrs loss of consc	264291000006110	371161001	1209863015
Open #skull NOS + intracranial inj, no loss of consc	264311000006114	371161001	1209863015
Open #skull NOS + intracranial inj, unspec state of consc	264321000006118	371161001	1209863015
Focal traumatic hemorrhage of brainstem	7834411000006117	722623009	3332921017
Open #skull NOS + intracranial inj + concussion unspec	264261000006119	371161001	1209863015
Intracranial haemorrhage following injury with open intracranial wound AND loss of consciousness	2710741000006110	13289004	475195013
Subarachnoid haemorrhage following injury with open intracranial wound AND prolonged loss of consciousness (more than 24 hours) without return to pre-existing conscious level	3625151000006114	69178005	3625151000006114
Intracranial hemorrhage following injury	7378281000006118	450410005	2915467017
Intracranial injury with prolonged coma with open wound	455461018	311829000	455461018
Head injury with haemorrhage from nose	4395871000006112	127276009	474087019
Temporal resolution test	5598691000006114	277391005	413892012
Closed fracture of vault of skull with intracranial injury, with more than 24 hours loss of consciousness and return to pre-existing conscious level	560291000006113	207692002	318122015
Cortex contusion with open intracranial wound, with no loss of consciousness	597721000006118	209845006	320613015
Extradural haemorrhage following injury with open intracranial wound AND moderate loss of consciousness (1-24 hours)	659971000006111	59648004	498503011

Traumatic intracranial haemorrhage	660121000006113	450410005	2915482010
Intracranial haemorrhage following injury with open intracranial wound and prolonged loss of consciousness (more than 24 hours) without return to pre-existing level	4198361000006112	111671004	364052019
Injury of multiple blood vessels of head AND/OR neck	2601561000006119	6378004	11573012
Multiple open fractures of skull AND/OR face without intracranial injury	2829351000006110	20714001	34857016
Closed traumatic subdural intracranial hemorrhage	4854151000006118	209947002	2912529013
Traumatic intracranial extradural haematoma	5459201000006113	262949005	2916480013
Traumatic subdural haemorrhage confined to region of tentorium	7834501000006119	722628000	3327785019
Extradural haemorrhage following injury with open intracranial wound AND loss of consciousness	4198441000006117	111681000	364077018
Other cerebral haemorrhage following injury NOS	320897016	450418003	2916363013
Non-traumatic hematoma of subdural space of neuraxis	7375011000006110	449797005	2913008017
Craniotomy and removal of haematoma from extradural space	7312951000006114	445746008	2883205017
Extradural haemorrhage following injury without open intracranial wound AND with moderate loss of consciousness (1-24 hours)	3523671000006117	62973004	499442016
Steroid injection of greater occipital nerve	552331000000115	307951000000101	552331000000115
Subdural haemorrhage following injury with open intracranial wound AND moderate loss of consciousness (1-24 hours)	3965681000006115	90165008	3965681000006115
Extradural hemorrhage following injury without open intracranial wound	3196371000006118	43216008	72101019
Traumatic brain injury with brief loss of consciousness	4396291000006115	127299008	587015
Traumatic intracranial subarachnoid haemorrhage	5459271000006119	262955000	391048011
Subdural hemorrhage following injury without open intracranial wound	4854171000006111	209947002	2915477015
Traumatic intracranial subarachnoid hemorrhage	5459291000006118	262955000	391047018
Non-traumatic spinal subdural hematoma	6028581000006119	313304007	457178019
Laceration of brain with open intracranial wound	2863601000006110	22819008	38306018
Injury of extracranial vessel of head	7863871000006119	725002006	3436884013
Cerebral hemorrhage following injury	7378411000006114	450418003	2915447014
Fracture of frontonasoethmoidal complex	5461371000006118	263153003	391283015
Chronic non-traumatic intracranial subdural hemorrhage	7497201000006112	609382000	2959747017
Brain stem contusion with open intracranial wound	3784231000006114	78968003	131037016
Traumatic hematoma of subdural space of neuraxis	7374991000006117	449796001	2913015013
Traumatic cranial subarachnoid haemorrhage	5459301000006117	262955000	391049015
Subdural hemorrhage following injury with open intracranial wound AND moderate loss of consciousness (1-24 hours)	11903641000006118	90165008	149462011
Traumatic intracranial hemorrhage	7378301000006119	450410005	2915928019
Lobotomy - brain	5559181000006119	273991000	409720015
Subarachnoid haemorrhage following injury with open intracranial wound AND concussion	3284981000006111	48518008	495117010
Extradural hemorrhage following injury without open intracranial wound AND with loss of consciousness	3965891000006118	90178008	149485012

Traumatic spinal extradural hematoma	5456221000006111	262720008	390719016
Cerebral haemorrhage following injury	505322013	450418003	2916363013
Traumatic cerebral haemorrhage	505324014	450418003	2916058017
Traumatic haematoma of subdural space of neuraxis	7374981000006115	449796001	2912448014
Extradural haemorrhage following injury with open intracranial wound	3559941000006115	65189006	3559941000006115
Subdural haemorrhage following injury with open intracranial wound and prolonged loss of consciousness (more than 24 hours) and without return to pre-existing conscious level	124041000006115	111677000	364064010
Open fracture of vault of skull with intracranial injury, with no loss of consciousness	263781000006117	207707005	318137016
Open fracture of base of skull with intracranial injury, with no loss of consciousness	264161000006114	207745006	318189014
Open traumatic subdural intracranial hemorrhage	4854221000006117	209956005	2912899010
Extradural haemorrhage following injury with open intracranial wound AND prolonged loss of consciousness (more than 24 hours) without return to pre-existing conscious level	3901101000006118	86182004	3901101000006118
Vestibular trauma due to head injury	4062681000006118	95817008	512313010
Brain injury without open intracranial wound AND with prolonged loss of consciousness (more than 24 hours) without return to pre-existing conscious level	3267451000006119	47462004	79134018
Intracranial haemorrhage following injury with open intracranial wound AND prolonged loss of consciousness (more than 24 hours) AND return to pre-existing conscious level	3982281000006110	91216000	509357015
Haemorrhage of brainstem due to traumatic injury	7834641000006116	722632006	3332942015
Intracranial haemorrhage following injury without open intracranial wound AND with loss of consciousness	2793481000006115	18485009	478644014
Extradural hemorrhage following injury with open intracranial wound AND prolonged loss of consciousness (more than 24 hours) without return to pre-existing conscious level	11924641000006113	86182004	142911018
Intracerebral injury	4396221000006117	127294003	474102014
Closed #skull bse + intracranial inj, unspec state of consc	559961000006110	111603000	178853015
Closed #skull NOS + intracranial inj, LOC unspec duration	560161000006112	371162008	1209864014
Closed #skull NOS + intracranial inj, unspec state of consc	560181000006119	371162008	1209864014
Closed #skull NOS no intracranial inj, 1-24hr loss of consc	560221000006111	371162008	1209864014
Closed #skull/face, mult + intracranial inj, 1-24hrs LOC	560441000006115	5468008	10127011
Closed #skull/face, mult + intracranial inj, unspec consc	560461000006116	5468008	10127011
Mult #skull/face + other bones, open + intracranial injury	696121000006114	5468008	10127011
Extradural haemorrhage following injury with open intracranial wound AND brief loss of consciousness (less than one hour)	3692151000006113	73308006	3692151000006113
Subdural hemorrhage following injury with open intracranial wound and prolonged loss of consciousness (more than 24 hours) and without return to pre-existing conscious level	4198411000006116	111677000	61538012

Evacuation of intracerebral hematoma	2666411000006111	10458001	18195013
Nontraumatic extradural hemorrhage	6593111000006115	397809001	1774439018
Closed fracture of base of skull with intracranial injury, with more than 24 hours loss of consciousness and return to pre-existing conscious level	559991000006119	207730004	318174017
Closed fracture of base of skull with intracranial injury, with 1-24 hours loss of consciousness	560001000006112	207729009	318173011
Closed fracture of base of skull with intracranial injury, with no loss of consciousness	560021000006119	207727006	318171013
Closed fracture of vault of skull with intracranial injury, with 1-24 hours loss of consciousness	560301000006114	207691009	318121010
Cortex contusion with open intracranial wound, with 1-24 hours loss of consciousness	597701000006111	209848008	320616011
Intracranial nontraumatic haemorrhage of foetus and newborn	744871000006114	206417009	2791314016
Hind brain contusion with open intracranial wound, with less than 1 hour loss of consciousness	823401000006116	209884001	320655012
Hind brain laceration with open intracranial wound, with less than 1 hour loss of consciousness	823531000006111	209903008	320675015
Subarachnoid hemorrhage following injury without open intracranial wound AND with loss of consciousness	3629581000006110	69458009	115392016
Traumatic subdural haematoma with open intracranial wound	459514014	315047001	459514014
Intracranial haematoma following injury	486896012	37955001	486896012
Open fracture of vault of skull with intracranial injury, with more than 24 hours loss of consciousness and return to pre-existing conscious level	52931000006111	207710003	318140016
Brain stem contusion with open intracranial wound AND loss of consciousness	3579631000006117	66393002	110266011
Intracranial haemorrhage following injury without open intracranial wound AND with prolonged loss of consciousness (more than 24 hours) AND return to pre-existing conscious level	3776591000006112	78477003	503848012
Intracranial haemorrhage following injury without open intracranial wound AND with no loss of consciousness	3098131000006113	37134004	486631018
Open #skull/face, mult + intracran inj, LOC unspec duration	263911000006110	5468008	10127011
Open #skull/face, mult + intracranial inj, <1hr LOC	263921000006119	5468008	10127011
Open #skull bse + intracranial injury, LOC unspec duration	264151000006112	111607004	178855010
Hind brain laceration with open intracranial wound, with no loss of consciousness	823561000006119	209902003	320674016
Subarachnoid haemorrhage following injury with open intracranial wound AND prolonged loss of consciousness (more than 24 hours) AND return to pre-existing conscious level	123281000006117	73439007	502436011
Extradural haemorrhage following injury with open intracranial wound AND prolonged loss of consciousness (more than 24 hours) AND return to pre-existing conscious level	659961000006116	81520002	504713019
Intracranial haemorrhage following injury with open intracranial wound	3765291000006112	77768006	503672016
Extradural hemorrhage following injury with open intracranial wound AND brief loss of consciousness (less than one hour)	11924661000006112	73308006	121738013
Intracranial haemorrhage following injury without open intracranial wound AND with concussion	3781101000006114	78757009	503910018

Intracranial haemorrhage following injury with moderate loss of consciousness	4396471000006117	127310009	474111014
Birth brain damage NOS	513921000006111	240312009	359984010
Closed #skull bse no intracranial inj, LOC unspec duration	560031000006116	27644009	46274013
Closed #skull bse no intracranial injury, no loss of consc	560091000006117	27644009	46274013
Closed #skull bse no intracranial injury, unspec state consc	560101000006111	24063002	40395011
Closed #skull NOS + intracranial inj + concussion unspec	560111000006114	371162008	1209864014
Closed #skull NOS no intracranial inj + concussion unspec	560191000006116	371162008	1209864014
Closed #skull/face, mult + intracranial inj, <1hr LOC	560431000006113	5468008	10127011
Cortex cont + open intracranial wound + concussion unspec	597711000006114	209843004	320611018
Cortex lacn no open intracranial wnd + >24hr LOC + recovery	597931000006118	78914008	130942015
Cortex lacn no open intracranial wnd +>24hr LOC not restored	597961000006110	78914008	130942015
Non-traumatic intracranial subdural haematoma	5656921000006118	281864001	419994017
Multiple traumatic haemorrhages of brain tissue	7834651000006119	722633001	3332945018
Epidural haemorrhage	505381016	82999001	505381016
Subarachnoid hemorrhage following injury with open intracranial wound AND loss of consciousness	3727881000006110	75507000	125412015
Subdural haemorrhage following injury with open intracranial wound AND loss of consciousness	3237311000006119	45659008	494311012
Open fracture of base of skull with intracranial injury, with 1-24 hours loss of consciousness	264141000006110	207747003	318191018
Brain contusion with open intracranial wound, with more than 24 hours loss of consciousness and return to pre-existing conscious level	524301000006112	209923009	320695014
Closed fracture of base of skull with intracranial injury, with more than 24 hours loss of consciousness without return to pre-existing conscious level	559951000006113	207731000	318175016
Cortex contusion with open intracranial wound, with less than 1 hour loss of consciousness	597691000006111	209847003	320615010
Extradural haemorrhage following injury with open intracranial wound, with no loss of consciousness	660021000006112	209978003	320817017
Subdural hemorrhage following injury	4854341000006117	209987007	320838011
Closed traumatic subdural intracranial haemorrhage	4854161000006116	209947002	2912797018
Non-traumatic haematoma of subdural space of neuraxis	7375001000006112	449797005	2912549016
Cerebellar laceration with open intracranial wound AND moderate loss of consciousness (1-24 hours)	3879281000006119	84803006	140595014
Intracranial haemorrhage following injury with prolonged loss of consciousness AND return to pre-existing conscious level	4396491000006116	127311008	474112019
Subarachnoid haemorrhage following injury without open intracranial wound	402929011	28048009	483737017
Subdural hemorrhage - nontraumatic	4777911000006112	195176009	300293019
Extradural hemorrhage after injury	5459171000006114	262949005	391027014
Subdural haemorrhage following injury with open intracranial wound	4854241000006112	209956005	2916362015

Intracranial haemorrhage following injury without intracranial wound AND with moderate loss of consciousness (1-24 hours)	3589021000006119	66976009	500582010
Craniotomy with treatment of penetrating wound of brain	3786211000006110	79093007	131239012
Subarachnoid hemorrhage following injury without open intracranial wound AND with moderate loss of consciousness (1-24 hours)	4198391000006116	111673001	195613013
Brain stem laceration without open intracranial wound AND with prolonged loss of consciousness (more than 24 hours) without return to pre-existing conscious level	3596181000006113	67378005	111973015
Brain stem contusion with open intracranial wound AND prolonged loss of consciousness (more than 24 hours) AND return to pre-existing conscious level	2767251000006115	16837005	28508011
Subdural hemorrhage following injury with open intracranial wound	4854231000006119	209956005	2915474010
Concussion of periodontal ligament	5462911000006115	263275009	391448016
Extradural haemorrhage following injury with open intracranial wound AND concussion	2951801000006112	28155008	483768018
Hind brain contusion with open intracranial wound, with no loss of consciousness	823411000006118	209883007	320654011
Hind brain laceration with open intracranial wound, with 1-24 hours loss of consciousness	823541000006118	209904002	320676019
Subdural hemorrhage following injury without open intracranial wound AND with prolonged loss of consciousness (more than 24 hours) without return to pre-existing conscious level	3652491000006116	70861009	117705011
Traumatic extradural hematoma	5459161000006119	262949005	391026017
Subarachnoid haemorrhage following injury without open intracranial wound AND with concussion	2567821000006112	4332009	493572019
Intracranial hemorrhage following injury with open intracranial wound AND no loss of consciousness	2624491000006117	7819003	13934013
Intracranial haemorrhage following injury with intracranial wound AND moderate loss of consciousness (1-24 hours)	3055541000006111	34501004	485859011
Auditory cranial nerve injury	511287019	9441002	511287019
Traumatic cerebral oedema with open intracranial wound	455458019	311826007	455458019
Injury of intracranial vessel of head	7815641000006111	721328009	3324974017
Oth cerebral laceration/contusion + open intracranial wound	39841000006117	269144002	402921014
Subdural h'ge inj no open intracranial wound+<1hr loss consc	124181000006110	209947002	320752018
Extradural haemorrhage following injury without open intracranial wound	402930018	43216008	493552012
Open fracture of vault of skull with intracranial injury, with more than 24 hours loss of consciousness without return to pre-existing conscious level	264421000006112	207711004	318141017
Primary traumatic hemorrhage of brainstem	7840841000006112	723138000	3334696017
Traumatic brain injury of unknown intent	7638391000006118	708728007	3083294016
Intracranial hemorrhage following injury with open intracranial wound AND prolonged loss of consciousness (more than 24 hours) AND return to pre-existing conscious level	3982291000006113	91216000	151139013
Sequela of traumatic intracranial haemorrhage	8031551000006111	100581000119102	2967472012

Intracranial hemorrhage following injury without open intracranial wound AND with no loss of consciousness	3098141000006115	37134004	61953011
Brain stem laceration with open intracranial wound AND no loss of consciousness	2804791000006111	19210000	32368010
Intracranial inj NOS no open intracran wnd + unspec consc	768821000006111	127296001	584010
Intracranial injury NOS + open intracranial wound	768911000006110	127296001	584010
Intracranial injury NOS no open intracranial wound	768921000006119	127296001	584010
Injury to blood vessels of head and neck	774541000006119	85564003	141827011
Hind brain lacn + open intracranial wnd + concussion unspec	823551000006116	209900006	320672017
Hind brain lacn + open intracranial wnd + unspec state consc	823571000006114	209900006	320672017
Subdural h'ge inj no open intracran wound+concussion unspec	124151000006119	209947002	320752018
Subdural h'ge inj no open intracranial wnd + unspec consc	124161000006117	209947002	320752018
Subdural h'ge inj no open intracranial wnd+>24 LOC +recovery	124171000006112	209947002	320752018
Traumatic cerebral haemorrhage	12757931000006114	82894007	505324014
Traumatic cerebral hemorrhage	12757881000006115	82894007	137503010
Complex wound of head with avulsive loss of part of skull and cranial contents	7840931000006112	723143007	3334711017
Nontraumatic subarachnoid intracranial haemorrhage	5518111000006112	270907008	3449602014
Hemorrhage of brainstem due to traumatic injury	7834631000006114	722632006	3332941010
Subdural hemorrhage following injury with open intracranial wound AND brief loss of consciousness (less than one hour)	2976661000006116	29635000	49572012
Nontraumatic intracranial hemorrhage	3533577011	738779002	3533577011
Traumatic intracranial subdural hematoma	5459241000006110	262952002	391038014
Secondary traumatic hemorrhage of brainstem	7840861000006111	723139008	3334699012
Oth cereb h'ge inj + open intracran wnd+concussion unspec	39711000006113	450418003	2915288012
Oth cerebral h'ge inj no open intracranial wnd+no loss consc	39831000006110	450418003	2915288012
Subarach h'ge inj no open intracran wnd+LOC unspec duration	123331000006114	28048009	46957015
Subdural h'ge inj + open intracranial wnd+concussion unspec	124081000006114	209956005	320770015
Extradural h'ge inj no open intracran wnd+concussion unspec	660071000006113	262949005	391026017
Cortex contusion without open intracranial wound AND with loss of consciousness	2777751000006115	17498002	29575016
Cerebellar laceration with open intracranial wound AND brief loss of consciousness (less than one hour)	2651541000006113	9501009	16628012
Focal hemorrhagic contusion of cerebrum	7834271000006110	722615007	3332902013

Table S19. Spinal Injury Aurum codes

Term	Medcode ID	SNOMED CT Concept ID	SNOMED CT Description ID
Injury of spinal cord vasculature	5456141000006113	262715008	390711018

Table S20. Eye Injury Aurum codes

Term	Medcode ID	SNOMED CT Concept ID	SNOMED CT Description ID
Haemophthalmos, except current injury	4771081000006111	193287005	2550868017
Hemophthalmos, except current injury	4771091000006114	193287005	2550869013
Perforating injury of both eyeballs	3515113016	735658005	3515113016
Haemophthalmos without current injury	4771041000006117	193287005	2547718010
Bilateral penetrating injury of eyeball	3515111019	735657000	3515111019
Repair of penetrating eye injury	8255451000006117	800491000000104	1789891000000113
Haemophthalmos (excluding current injury)	297686013	193287005	297686013
Penetrating injury of both eyeballs	3515110018	735657000	3515110018
Repair of penetrating eye injury	11999811000006115	763097008	3637861018
Hemophthalmos without current injury	4771071000006113	193287005	2547719019
Haemorrhage into cornea	5029221000006110	231916005	347514014

Table S21. Estimated glomerular filtration rate Aurum codes

Term	Medcode ID	SNOMED CT Concept ID	SNOMED CT Description ID
EgfrUsingCreatinine(ckd-epi)Per1.73SquareMetres	1942831000006114	1011481000000105	2579541000000113
EstimatedCreatinineClearance	2470380013	395680003	2470380013
EstimatedCreatinineClearance(cockcroft-gaultFormula)	2465711000000116	968191000000100	2465711000000116
GlomerularFiltrationRate	133205018	80274001	133205018
SerumCreatinine	380389013	1000731000000107	2577271000000111
GfrCalculatedAbbreviatedMdrd	976481000006110	1020291000000106	2569781000000116

Table S22. Azole Aurum codes

Term	Medcode ID	SNOMED CT Concept ID	SNOMED CT Description ID
Atherosclerosis aorta	3830431000006112	81817003	2923328010
Aortic thromboembolism	5560171000006116	274101000	409861010
Aortoiliac atherosclerosis	5057931000006110	233956002	350530011
Aortic atherosclerosis	218531000000113	81817003	135728018

Table S23. Dementia Aurum codes

Term	Medcode ID	SNOMED CT Concept ID	SNOMED CT Description ID
H/O: dementia	251625013	161465002	251625013
[X]Predominantly cortical dementia	423221000006117	56267009	93568017
[X]Presenile dementia,Alzheimer's type	423351000006115	416780008	2957138011
[X]Primary degen dementia, Alzheimer's type, presenile onset	423391000006114	416780008	2957119019
Lewy body variant of Alzheimer's disease	3802621000006119	80098002	132894011
SDLT - Senile dementia of the Lewy body type	3802631000006116	80098002	1234435012
[X]Senile dementia,Alzheimer's type	425901000006116	416975007	2957124016
Dementia associated with alcoholism	2502971000006115	281004	1557018
Senile dementia with depressive or paranoid features NOS	294647019	191457008	294644014
[X]Vascular dementia, unspecified	295681016	429998004	2770951017
[X]Dementia in other specified diseases classif elsewhere	295690011	191519005	2547722017
Dementia infantilis	3670601000006111	71961003	119578017
Dementia co-occurrent and due to Pick's disease	8009541000006119	21921000119103	3333186018
Dementia due to Picks disease	8009521000006114	21921000119103	2987132018
ADC - Acquired immune deficiency syndrome dementia complex	6973451000006113	421529006	2622766016
Alzheimer's disease with early onset	499946014	416780008	2957141019
Alzheimer's disease with late onset	500317011	416975007	2957124016
Dementia in Alzheimer's disease with early onset, other symptoms, predominantly delusional	1971541000006114	1971541000006105	1971541000006114
Dementia in Alzheimer's disease with early onset, other symptoms, predominantly hallucinatory	1971771000006112	1971771000006108	1971771000006112
Unspecified dementia, other symptoms, predominantly delusional	1972041000006114	1972041000006105	1972041000006114
Unspecified dementia, other symptoms, predominantly hallucinatory	1972061000006113	1972061000006109	1972061000006113
Dementia in Alzheimer's disease with late onset, other symptoms, predominantly delusional	1972181000006119	1972181000006103	1972181000006119
Dementia in Alzheimer's disease with late onset, other symptoms, predominantly depressive	1972201000006118	1972201000006102	1972201000006118
Dementia in Alzheimer's disease with late onset, other mixed symptoms	1972211000006115	1972211000006104	1972211000006115
Dementia in Alzheimer's dis, atypical or mixed type, other symptoms, predominantly hallucinatory	1972291000006113	1972291000006109	1972291000006113
Vascular dementia of acute onset, other symptoms, predominantly hallucinatory	1972521000006114	1972521000006105	1972521000006114
Subcortical vascular dementia, other symptoms, predominantly depressive	1972771000006116	1972771000006100	1972771000006116
Mixed cortical and subcortical vascular dementia, other symptoms, predominantly hallucinatory	1972871000006113	1972871000006109	1972871000006113
Mixed cortical and subcortical vascular dementia, other symptoms, predominantly depressive	1972911000006111	1972911000006107	1972911000006111
Mental and behav dis due to hallucinogens: resid & late-onset psychot dis, dementia	1973711000006116	1973711000006100	1973711000006116
Presenile dementia	21256010	12348006	21256010
Senile dementia	26545010	15662003	26545010

Alzheimer's disease	45046017	26929004	45046017
VAD - Vascular dementia	3414251000006112	56267009	497560014
Dementia due to Creutzfeldt-Jakob disease	7103601000006116	429458009	2693170019
Primary degenerative dementia of the Alzheimer type, late onset	6900191000006112	416975007	2554010014
Transmissible virus dementia	2511001000006119	792004	2164150017
Binswanger's dementia	3964611000006111	90099008	149345019
Lewy body disease	745381000006119	80098002	132893017
Mental and behav dis due to other stimulants inc caffeine: resid/late-onset psycht dis, dementia	1976091000006118	1976091000006102	1976091000006118
Primary degenerative dementia of the Alzheimer type, early onset	6897221000006113	416780008	2553939016
Acquired immune deficiency syndrome dementia complex	6973461000006110	421529006	2622767013
Dementia associated with AIDS	6973421000006116	421529006	2617355019
MID - Multi-infarct dementia	3414231000006117	56267009	497558012
Arteriosclerotic dementia NOS	294656010	56267009	497559016
[X]Alzheimer's dementia unspec	363021000006113	26929004	45046017
[D] Dementia with Lewy bodies	914931000006119	914931000006103	914931000006119
[D] Dementia	914941000006112	914941000006108	914941000006112
[X] Senile dementia NOS	359141000006111	15662003	26545010
[X]Alzheimer's disease type 2	363041000006118	416780008	2957119019
[X]Dementia in human immunodef virus [HIV] disease	376571000006116	421529006	2622767013
[X]Dementia infantalis	376631000006115	71961003	119576018
Uncomplicated senile dementia	294635013	191449005	294635013
Presenile dementia with delirium	294638010	191452002	294638010
Uncomplicated arteriosclerotic dementia	294652012	191463004	294652012
Arteriosclerotic dementia with delirium	294653019	191464005	294653019
Arteriosclerotic dementia with paranoia	294654013	191465006	294654013
Arteriosclerotic dementia with depression	294655014	191466007	294655014
Dementia	882171000006115	268612007	882171000006115
Dementia in Alzheimer's disease - type 2	6897271000006114	416780008	2957156011
LBD - Lewy body disease	3802641000006114	80098002	1234436013
DLBD - Diffuse Lewy body disease	3802661000006113	80098002	1234438014
Primary degenerative dementia of the Alzheimer type, senile onset	6900181000006114	416975007	2549518019
Dementia of the Alzheimers type with early onset	6897241000006118	416780008	2957133019
Presenile dementia, Alzheimer's type	6897251000006116	416780008	2957138011
[X]Dementia in other diseases classified elsewhere	295684012	191519005	2547722017
[X]Dementia in Pick's disease	295685013	21921000119103	2968126015
[X]Dementia in Creutzfeldt-Jakob disease	295686014	429458009	2695885013
[X]Dementia in Parkinson's disease	295688010	425390006	2921040013
AD - Alzheimer's disease	2931231000006118	26929004	1225144019
Dementia of the Alzheimers type, late onset	6900201000006110	416975007	2957123010
[X]Dementia in Alzheimer's disease	295668011	26929004	45046017

[X]Dementia in Alzheimer's disease, unspecified	295672010	26929004	45046017
[X] Senile dementia, depressed or paranoid type	359151000006113	191457008	294644014
Nuchal dystonia-dementia syndrome	2966081000006114	28978003	48514014
Dementia in Alzheimer's disease with early onset, without additional symptoms	1971401000006111	1971401000006107	1971401000006111
Mental & behav dis due to seds/hypntcs: resid & late-onset psychot dis, dementia	1971661000006114	1971661000006105	1971661000006114
Dementia in Alzheimer's dis, atypical or mixed type, other symptoms, predominantly depressive	1972311000006112	1972311000006108	1972311000006112
Dementia in Alzheimer's dis, atypical or mixed type, other mixed symptoms	1972341000006111	1972341000006107	1972341000006111
Dementia in Alzheimer's disease, unspecified, without additional symptoms	1972371000006115	1972371000006104	1972371000006115
Mental & behav dis due to cannabinoids: resid & late-onset psychot dis, dementia	1972431000006113	1972431000006109	1972431000006113
Vascular dementia of acute onset, other symptoms, predominantly delusional	1972501000006116	1972501000006100	1972501000006116
Other vascular dementia, other symptoms, predominantly depressive	1973381000006112	1973381000006108	1973381000006112
[RFC] Dementia	939491000006118	939491000006102	939491000006118
Drug-induced dementia	294688019	191493005	294688019
JCD - Jakob-Creutzfeldt disease	2510991000006115	792004	1234326016
[X] Unspecified dementia	359241000006119	52448006	87274019
Senile dementia-acute confused	882211000006118	191461002	882211000006118
[X]Dementia in Alzheimer's disease with early onset	376531000006119	416780008	2957119019
[X]Dementia in Alzheimer's disease with late onset	376541000006112	416975007	2957137018
[X]Vascular dementia	431681000006117	429998004	2770951017
[X]Vascular dementia of acute onset	431691000006119	230285003	345110014
Multi infarct dementia	3414261000006114	56267009	2921000019
Alzheimer dementia	2931251000006113	26929004	3424952012
[D] Vascular dementia	914921000006117	914921000006101	914921000006117
[D] Dementia in Alzheimer's disease	914951000006114	914951000006105	914951000006114
Dementia associated with Parkinson Disease	7043661000006117	425390006	2842012010
Senile dementia with delirium	294648012	191461002	294648012
[X]Dementia in Huntington's disease	295687017	442344002	2820374011
Alcoholic dementia NOS	346929012	281004	1225776018
Uncomplicated presenile dementia	294637017	191451009	294637017
Senile dementia with depression	294646011	191459006	294646011
[X] Primary degenerative dementia NOS	359101000006114	279982005	417465012
[X]Alzheimer's disease type 1	363031000006111	416975007	2957137018
Dementia due to Huntingtons disease	7263021000006116	442344002	2951827019
[X]Primary degen dementia of Alzheimer's type, senile onset	423381000006111	416975007	2957137018
Presenile dementia NOS	294643015	12348006	21256010
Mental & behav dis due to use opioids: resid & late-onset psychot dis, dementia	1971701000006118	1971701000006102	1971701000006118
Unspecified dementia, without additional symptoms	1972021000006119	1972021000006103	1972021000006119
Unspecified dementia, other mixed symptoms	1972081000006115	1972081000006104	1972081000006115

Dementia in Alzheimer's disease with early onset, other symptoms, predominantly depressive	1972131000006115	1972131000006104	1972131000006115
Dementia in Alzheimer's disease with early onset, other mixed symptoms	1972141000006113	1972141000006109	1972141000006113
Dementia in Alzheimer's dis, atypical or mixed type, without additional symptoms	1972231000006114	1972231000006105	1972231000006114
Dementia in Alzheimer's dis, atypical or mixed type, other symptoms, predominantly delusional	1972251000006119	1972251000006103	1972251000006119
Dementia in Alzheimer's disease, unspecified, other mixed symptoms	1972471000006111	1972471000006107	1972471000006111
Vascular dementia of acute onset, without additional symptoms	1972481000006114	1972481000006105	1972481000006114
Multi-infarct dementia, other symptoms, predominantly delusional	1972621000006113	1972621000006109	1972621000006113
Multi-infarct dementia, other symptoms, predominantly depressive	1972661000006119	1972661000006103	1972661000006119
Subcortical vascular dementia, without additional symptoms	1972711000006113	1972711000006109	1972711000006113
Subcortical vascular dementia, other symptoms, predominantly delusional	1972731000006119	1972731000006103	1972731000006119
Other vascular dementia, without additional symptoms	1973221000006118	1973221000006102	1973221000006118
Other vascular dementia, other symptoms, predominantly delusional	1973271000006117	1973271000006101	1973271000006117
Other vascular dementia, other mixed symptoms	1973401000006112	1973401000006108	1973401000006112
Mental & behav dis due to use cocaine: resid & late-onset psychot dis, dementia	1973941000006119	1973941000006103	1973941000006119
Multi infarct dementia	696161000006115	56267009	93568017
Subcortical atherosclerotic dementia	3964661000006114	90099008	1235536019
Dementia associated with acquired immunodeficiency syndrome	6973471000006115	421529006	2970885015
CLBD - Cortical Lewy body disease	3802681000006115	80098002	1234440016
[X]Lewy body dementia	299641000000112	80098002	132893017
Mental & behav dis due to tobacco: resid & late-onset psychot dis, dementia	1974931000006114	1974931000006105	1974931000006114
Mental & behav dis due to use alcohol: resid & late-onset psychot dis, dementia	1975591000006119	1975591000006103	1975591000006119
Dementia associated with Parkinson's Disease	7043651000006119	425390006	2645368017
Organic dementia	3350441000006115	52448006	2970981011
Senile/presenile dementia	148381000006115	52448006	87274019
Senile and presenile dementias	882191000006119	268612007	882191000006119
Jakob-Creutzfeldt disease	2386018	792004	2386018
Pick's disease	22408016	13092008	22408016
[X]Delirium superimposed on dementia	295714013	2776000	5694012
Dementia due to Pick disease	8009531000006112	21921000119103	2987352013
Primary degenerative dementia of the Alzheimer type, presenile onset	6897211000006117	416780008	2549310013
[RFC] Alzheimer's disease	905791000006115	905791000006104	905791000006115
Presenile dementia with depression	294642013	191455000	294642013
Senile dementia with depressive or paranoid features	294644014	191457008	294644014
Senile dementia with paranoia	294645010	191458003	294645010
Dementia in Alzheimer's disease with late onset, other symptoms, predominantly hallucinatory	1972191000006116	1972191000006100	1972191000006116

Vascular dementia of acute onset, other symptoms, predominantly depressive	1972541000006119	1972541000006103	1972541000006119
Multi-infarct dementia, without additional symptoms	1972601000006115	1972601000006104	1972601000006115
Subcortical vascular dementia, other mixed symptoms	1972791000006115	1972791000006104	1972791000006115
Mixed cortical and subcortical vascular dementia, without additional symptoms	1972821000006112	1972821000006108	1972821000006112
Mental and behav dis due to vol solvents: resid & late-onset psychotic dis, dementia	1973171000006112	1973171000006108	1973171000006112
Other vascular dementia, other symptoms, predominantly hallucinatory	1973341000006118	1973341000006102	1973341000006118
Vascular dementia, unspecified, without additional symptoms	1973461000006113	1973461000006109	1973461000006113
Vascular dementia, unspecified, other symptoms, predominantly hallucinatory	1973531000006117	1973531000006101	1973531000006117
SD - Senile dementia	2748441000006111	15662003	1221222011
[X]Other vascular dementia	295680015	429998004	2770951017
[X]Other Alzheimer's disease	299325013	26929004	45046017
[X] Presenile dementia NOS	359081000006118	12348006	21256010
[X]Arteriosclerotic dementia	363791000006112	56267009	497559016
[X]Dementia in Alzheimer's dis, atypical or mixed type	295671015	26929004	45046017
Dementia in Alzheimer's disease, unspecified, other symptoms, predominantly delusional	1972401000006117	1972401000006101	1972401000006117
Dementia in Alzheimer's disease, unspecified, other symptoms, predominantly hallucinatory	1972421000006110	1972421000006106	1972421000006110
Multi-infarct dementia, other symptoms, predominantly hallucinatory	1972641000006118	1972641000006102	1972641000006118
Multi-infarct dementia, other mixed symptoms	1972681000006112	1972681000006108	1972681000006112
Vascular dementia, unspecified, other symptoms, predominantly depressive	1973551000006112	1973551000006108	1973551000006112
Vascular dementia, unspecified, other mixed symptoms	1976831000006111	1976831000006107	1976831000006111
Arteriosclerotic dementia	497559016	56267009	497559016
Dementia due to Huntington disease	7263011000006112	442344002	2820375012
Dementia in Alzheimer's disease - type 1	6900241000006112	416975007	2957155010
Unspecified dementia, other symptoms, predominantly depressive	1972071000006118	1972071000006102	1972071000006118
Dementia in Alzheimer's disease with late onset, without additional symptoms	1972171000006117	1972171000006101	1972171000006117
Dementia in Alzheimer's disease, unspecified, other symptoms, predominantly depressive	1972451000006118	1972451000006102	1972451000006118
Vascular dementia of acute onset, other mixed symptoms	1972571000006110	1972571000006106	1972571000006110
Subcortical vascular dementia, other symptoms, predominantly hallucinatory	1972751000006114	1972751000006105	1972751000006114
Mixed cortical and subcortical vascular dementia, other symptoms, predominantly delusional	1972831000006110	1972831000006106	1972831000006110
Mixed cortical and subcortical vascular dementia, other mixed symptoms	1972931000006117	1972931000006101	1972931000006117
Vascular dementia, unspecified, other symptoms, predominantly delusional	1973501000006113	1973501000006109	1973501000006113
Mental and behav dis mlti drg use/oth psych subs: resid/late psychot dis, dementia	1974271000006119	1974271000006103	1974271000006119
Dementia confirmed	1823871000006112	1823871000006108	1823871000006112
AIDS - Acquired immune deficiency syndrome dementia complex	6973441000006111	421529006	2622765017

Alcohol-induced persisting dementia	2502981000006117	281004	1558011
Acquired immune deficiency syndrome-related dementia	6973431000006118	421529006	2622764018
Dementia paralytica	3341641000006113	51928006	86446018
SDAT - Senile dementia, Alzheimer's type	6900221000006117	416975007	2957134013
[X]Mixed cortical and subcortical vascular dementia	398571000006112	230287006	345112018
[X]Alcoholic dementia NOS	362941000006113	281004	1225776018
Dementia of the Lewy body type	3802651000006111	80098002	1234437016
[X]Subcortical vascular dementia	428201000006119	230286002	345111013
Cortical Lewy body disease	3802671000006118	80098002	1234439018
Paralytic dementia	3341701000006116	51928006	1231057014
[X]Multi-infarct dementia	399031000006111	56267009	93568017
Other alcoholic dementia	401760017	281004	1225776018
Senile dementia - simple type	882201000006116	191449005	882201000006116
Presenile dementia with paranoia	294641018	191454001	294641018
Dementia in conditions EC	294718018	191519005	294718018

Chapter 2. Code lists for trial outcomes

Table S24. Cardivascular mortality HES (ICD10) codes

ICD10 codes	Term
I00-I02	Acute rheumatic fever
I05-I09	Chronic rheumatic heart diseases
I10-I15	Hypertensive diseases
I20-I25	Ischaemic heart diseases
I26-I28	Pulmonary heart disease and diseases of pulmonary circulation
I30-I52	Other forms of heart disease
I60-I69	Cerebrovascular diseases
I70-I78 (exclude I78.0, I78.1)	Diseases of arteries, arterioles and capillaries
G46	Vascular syndromes of brain in cerebrovascular diseases
G45	Transient cerebral ischaemic attacks and related syndromes
R57.0	Cardiogenic shock
K76.1	Chronic passive congestion of liver
J81	Pulmonary oedema

Table S25. Ischaemic stroke Aurum codes

Term	Medcode ID	SNOMED CT Concept ID	SNOMED CT Description ID
[X]Other cerebral infarction	300941014	432504007	2770034014
Multiple lacunar infarcts	5967391000006111	307363008	450609012
Stroke due to basilar artery thrombus	3508956015	329641000119104	3508956015
Cerebrovascular accident due to thrombus of right middle cerebral artery	3508961018	16002031000119102	3508961018
Total anterior cerebral circulation stroke	5011071000006114	230694003	345642016
[RFC] Stroke/CVA	907581000006119	907581000006103	907581000006119
H/O: stroke	2476091017	275526006	2476091017
Right sided CVA	300371014	195217004	300371014
Brain stem infarct	4057031000006117	95457000	158114011
History of stroke in last year	5974521000006116	308067002	2986393012
Left sided cerebral hemisphere cerebrovascular accident	4778181000006119	195216008	300369014
Stroke due to thrombus of right middle cerebral artery	3508962013	16002031000119102	3508962013
Cerebral infarction due to embolism of precerebral arteries	300313017	195186005	300313017
PACI - Partial anterior cerebral circulation infarction	5011101000006116	230695002	345646018
Cerebellar stroke	9912271000006112	16371781000119100	3320493016
Cerebellar infarction	158118014	95460007	158118014
TACS - Total anterior cerebral circulation stroke	5011081000006112	230694003	345643014
Infarction of brain stem	4057051000006112	95457000	2966602014
Total anterior cerebral circulation infarction	5011051000006116	230694003	345644015
Cerebrovascular accident	405339016	230690007	345637012
History of cerebrovascular accident	809421000006116	275526006	2986886017
CVA unspecified	605501000006117	230690007	345637012
Posterior cerebral circulation stroke	5011141000006119	230696001	2966565019
Thalamic stroke	7071911000006114	427296003	2966596011
Partial anterior cerebral circulation stroke	5011111000006118	230695002	345647010
Right sided cerebral infarction	451134017	307767006	451134017
Left sided cerebral infarction	451133011	307766002	451133011
Multi-infarct state	5011261000006110	230704000	345662012
Thalamic infarction	7071901000006111	427296003	2674127019
Choroidal infarct	6041261000006115	314003000	458273015
LI - Lacunar infarction	5011181000006113	230698000	345654016
[RFC] Stroke	907591000006116	907591000006100	907591000006116
[V]Personal history of stroke	1227591017	275526006	2476091017
[X]Cerebrl infarctn due/unspcf occlusn or sten/cerebrl artrs	370701000006118	20059004	33759015
Cerebral infarct due to thrombosis of precerebral arteries	300312010	195185009	300312010
Cerebral infarction NOS	12727431000006119	682621000000105	1495311000000117
Lacunar stroke	5011161000006115	230698000	345652017

Cerebral infarction due to occlusion of precerebral artery	542261000006114	125081000119106	3042974014
Cerebral infarction due to thrombosis of cerebral arteries	300321011	195189003	300321011
Cerebral ischaemic stroke due to small artery occlusion	7855631000006111	724424009	3438036013
Haemorrhagic cerebral infarction	5011291000006119	230706003	345667018
Cerebral infarction due to stenosis of cerebral artery	2729571000000119	1089421000000105	2729571000000119
Left sided CVA	300370010	195216008	300370010
Stroke due to thrombus of left middle cerebral artery	3508965010	16002111000119106	3508965010
Cerebral infarction due to embolism of cerebral arteries	300322016	195190007	300322016
Posterior cerebral circulation infarction	5011131000006112	230696001	345649013
Stroke	884531000006117	685631000000102	884531000006117
[RFC] CVA	909171000006115	909171000006104	909171000006115
Brainstem infarction NOS	345650013	95457000	158113017
Lacunar infarction	299342019	230698000	345651012
Sequelae of stroke,not specfd as h'morrhage or infarction	149571000006118	195239002	300403014
Infarction of basal ganglia	2474651019	413102000	2474651019
Brain stem infarction	524541000006117	95457000	158113017
[V]Personal history of cerebrovascular accident (CVA)	1227592012	266995000	397829016
Occipital cerebral infarction	5582311000006110	276219001	412270012
Anterior circulation stroke of uncertain pathology	5011461000006111	230714009	345681016
Occlusive stroke	1212072018	373606000	1212072018
Stroke of uncertain pathology	5011451000006114	230713003	345680015
Cerebral infarction	395780010	432504007	2770034014
PACS - Partial anterior cerebral circulation stroke	5011121000006114	230695002	345648017
Cerebrovascular accident due to cerebral artery occlusion	5011021000006113	230691006	2914970017
Cerebral infarction with haemorrhagic transformation	1573101000006112	1573101000006108	1573101000006112
[X]Sequelae of stroke,not specfd as h'morrhage or infarction	426321000006116	195239002	300403014
CVA - Cerebrovascular accident	605491000006113	230690007	345635016
Stroke/CVA - undefined	884521000006115	685631000000102	884521000006115
Anterior cerebral circulation haemorrhagic infarction	5011331000006114	230707007	345668011
H/O: CVA	411518010	275526006	411518010
Cerebral infarction due to thrombosis of middle cerebral artery	7599001000006116	705130002	3023374015
Ischemic stroke	6990991000006112	422504002	2644233012
Stroke unspecified	122401000006115	230690007	345637012
Partial anterior cerebral circulation infarction	5011091000006110	230695002	345645019
Basal ganglion stroke	6837051000006119	413102000	2966612019
LACI - Lacunar infarction	5011171000006110	230698000	345653010
Brainstem stroke syndrome	524511000006116	195212005	300365015
Right sided cerebral hemisphere cerebrovascular accident	4778201000006118	195217004	300372019

Infarction - cerebral	218511000000117	432504007	2770034014
Stroke and TIA	8221321000006118	720191000000104	1574361000000119
Sequela of ischaemic cerebral infarction	8011371000006117	23671000119107	2967516015
Cerebral infarction due to occlusion of cerebral artery	2729551000000111	1089411000000104	2729551000000111
Cerebral infarction due to embolism of middle cerebral artery	7598981000006119	705128004	3023367013
Infarction - precerebral	345639010	230692004	345639010
Stroke and cerebrovascular accident unspecified	12727691000006117	685631000000102	1501361000000113
H/O: Stroke in last year	451371010	308067002	451371010
Cerebrovascular accident due to occlusion of cerebral artery	11878511000006113	230691006	3636108019
CVA - cerebrovascular accident due to cerebral artery occlusion	605461000006117	230691006	345638019
[X]Cereb infarct due unsp occlus/stenos precerebr arteries	370661000006114	125081000119106	3042974014
Stroke due to cerebral arterial occlusion	122361000006113	230691006	345638019
Stroke	5010981000006119	230690007	345636015
Left hemispheric cerebellar artery embolism with stroke	3509817019	329461000119102	3509817019
Posterior circulation stroke of uncertain pathology	5011471000006116	230715005	345682011
Ischaemic stroke	6990981000006114	422504002	2644234018
Anterior cerebral circulation infarction	5011041000006118	230693009	345640012
TACI - Total anterior cerebral circulation infarction	5011061000006119	230694003	345641011
Sequelae of cerebral infarction	300411016	195243003	300411016
Embolic stroke	6348301000006119	371041009	1209751018
Brain stem stroke	4057041000006110	95457000	2966556014
Cause of Death- Cerebral Infarct	1576261000006112	1576261000006108	1576261000006112
[RFC] Stroke	908801000006114	908801000006105	908801000006114
Cerebrl infarctn due/unspcf occlusn or sten/cerebrl artrs	543141000006110	20059004	33759015
Sequela of ischemic cerebral infarction	8011381000006119	23671000119107	2967553014

Table S26. Ischaemic stroke HES (ICD10) codes

ICD10 codes	Term
I63.0	Cerebral infarction due to thrombosis of precerebral arteries
I63.1	Cerebral infarction due to embolism of precerebral arteries
I63.2	Cerebral infarction due to unspecified occlusion or stenosis of precerebral arteries
I63.3	Cerebral infarction due to thrombosis of cerebral arteries
I63.4	Cerebral infarction due to embolism of cerebral arteries
I63.5	Cerebral infarction due to unspecified occlusion or stenosis of cerebral arteries
I63.6	Cerebral infarction due to cerebral venous thrombosis, nonpyogenic
I63.8	Other cerebral infarction
I63.9	Cerebral infarction, unspecified
I64	Stroke, not specified as haemorrhage or infarction

Table S27. Transient ischaemic attack outcome Aurum codes

Term	Medcode ID	SNOMED CT Concept ID	SNOMED CT Description ID
Intermittent cerebral ischaemia	300353019	195206000	300353019
Anterior cerebral artery syndrome	300363010	195210002	300363010
Anterior circulation transient ischaemic attack	5011491000006115	230716006	345683018
Transient cerebral ischemia	11920121000006117	266257000	395785017
Personal history of transient ischaemic attack	8231151000006110	751371000000107	1653121000000119
Generalised ischaemic cerebrovascular disease NOS	300379011	302909007	444905019
Insufficiency - basilar artery	499739014	64009001	499739014
[V]Personal history of transient ischaemic attack	1667741000000110	751371000000107	1667741000000110
Vertebral artery syndrome	58046010	34781003	58046010
Chronic cerebral ischaemia	360778011	111298007	360778011
Basilar artery syndrome	106392017	64009001	106392017
Vertebrobasilar insufficiency	106394016	64009001	106394016
Transient ischaemic attack	395783012	266257000	395783012
Transient cerebral ischaemia	395788015	266257000	395788015
Other transient cerebral ischaemia	300348012	266257000	395788015
[X]Other transnt cerebral ischaemic attacks+related syndroms	416991000006112	266257000	395788015
Carotid territory transient ischaemic attack	345684012	230716006	345684012
Vertebrobasilar territory transient ischaemic attack	5011521000006118	230717002	345689019
Posterior cerebral artery syndrome	300364016	195211003	300364016
Occlusion and stenosis of cerebral arteries, not resulting in cerebral infarction	267321000006114	195231004	300394010
History of transient ischemic attack	4540591000006113	161511000	2986735018
Impending cerebral ischaemia	300352012	195205001	300352012
AF - Amaurosis fugax	3930471000006118	88032003	507856014
Transient cerebral ischaemia NOS	12733221000006110	584181000000100	1296321000000119
Transient Ischaemic Attacks	884511000006111	584181000000100	884511000006111
Transient cerebral ischaemia NOS	95931000006111	266257000	395788015
AFx - Amaurosis fugax	3930481000006115	88032003	507857017
Transient cerebral ischaemia NOS	300349016	266257000	395788015
Middle cerebral artery syndrome	300362017	195209007	300362017
Amaurosis fugax	145925010	88032003	145925010
Posterior circulation transient ischemic attack	5011541000006113	230717002	345688010
Amaurosis fugax of right eye	9478331000006110	12237951000119105	3326553010
Vertebro-basilar insufficiency	67511000006117	64009001	106394016
Carotid territory transient ischemic attack	5011511000006114	230716006	345686014
Anterior circulation transient ischemic attack	5011501000006111	230716006	345685013
Vertebrobasilar arterial insufficiency	3540781000006117	64009001	106393010
Suspected transient ischaemic attack	1704681000000110	473129008	2955917017
Amaurosis fugax (one sided temporary vision loss)	3930501000006113	88032003	2966609017
Suspected transient ischemic attack	7483201000006116	473129008	2955838012

Amaurosis	3645721000006118	70449006	117033019
H/O: TIA	251692018	161511000	251692018
Vertebro-basilar artery syndrome	67501000006115	195199008	300343015
Transient Ischaemic Attacks	988951000006117	266257000	988951000006117
TIA - Transient ischaemic attack	5492201000006111	266257000	395784018

Table S28. Transient ischaemic attack HES (ICD10) codes

ICD10 codes	Term
G45.2	Multiple and bilateral precerebral artery syndromes
G45.3	Amaurosis fugax
G45.8	Other transient cerebral ischaemic attacks and related syndromes
G45.9	Transient cerebral ischaemic attack, unspecified
G46.0	Middle cerebral artery syndrome
G46.1	Anterior cerebral artery syndrome
G46.2	Posterior cerebral artery syndrome
G46.3	Brain stem stroke syndrome
G46.4	Cerebellar stroke syndrome
G46.5	Pure motor lacunar syndrome
G46.6	Pure sensory lacunar syndrome
G46.7	Other lacunar syndromes
G46.8	Other vascular syndromes of brain in cerebrovascular diseases
I65.0	Occlusion and stenosis of vertebral artery
I65.1	Occlusion and stenosis of basilar artery
I65.2	Occlusion and stenosis of carotid artery
I65.3	Occlusion and stenosis of multiple and bilateral precerebral arteries
I65.8	Occlusion and stenosis of other precerebral artery
I65.9	Occlusion and stenosis of unspecified precerebral artery
I66.0	Occlusion and stenosis of middle cerebral artery
I66.1	Occlusion and stenosis of anterior cerebral artery
I66.2	Occlusion and stenosis of posterior cerebral artery
I66.3	Occlusion and stenosis of cerebellar arteries
I66.4	Occlusion and stenosis of multiple and bilateral cerebral arteries
I66.8	Occlusion and stenosis of other cerebral artery
I66.9	Occlusion and stenosis of unspecified cerebral artery
I67.2	Cerebral atherosclerosis
I67.9	Cerebrovascular disease, unspecified

Table S29. Venous thromoemoblism and pulmonary embolism Aurum codes

Term	Medcode ID	SNOMED CT Concept ID	SNOMED CT Description ID
Embolism and/or thrombosis of the internal iliac artery	300554012	734298005	3503835018
CRVT - Central retinal vein thrombosis	3614311000006116	68478007	501026011
H/O: Deep vein thrombosis	4540531000006114	161508001	4540531000006114
Thrombosis transverse sinus	296939018	192761004	296939018
Coronary thrombosis not resulting in myocardial infarction	299742017	194821006	299742017
Embolism and thrombosis of the brachial artery	300535012	195319003	300535012
Embolism and thrombosis of the posterior tibial artery	300543019	195327007	300543019
Mesenteric embolus	412653014	276500007	412653014
Ischaemic foot	443199013	301755001	443199013
Open removal of cardiac thrombus	2675367019	426067000	2675367019
Percutaneous transluminal thrombolysis of artery	2675878019	426485003	2675878019
Open thrombectomy of renal vein	2675885015	426937008	2675885015
Ischaemic disease of gut	3836741000006113	82196007	504940010
Acute intestinal ischaemic syndrome	3986491000006113	91489000	509476019
Recurrent pulmonary embolism	632111000000118	438773007	2793990014
Intestinal ischaemia	3836721000006118	82196007	504938017
Embolism and thrombosis of the celiac artery	4778981000006116	195342005	300560012
Ischaemia reperfusion injury of liver	7821701000006113	721715000	3325835015
AMI - Acute mesenteric ischaemia	3986481000006110	91489000	509475015
Deep venous thrombosis of lower extremity	6694741000006111	404223003	2156120013
Inner retinal ischaemic spots	3140891000006117	39832008	2620723015
Thrombosis of renal artery bypass graft	449993015	306857000	449993015
Ischaemic toe	450665015	307408003	450665015
Mesenteric thrombus and/or embolus	450682018	307420006	450682018
Thrombosed haemorrhoid incised	5727361000006116	287799004	2871742013
Infarct of liver	2783881000006119	17890003	30227010
TL - Transluminal pulmonary embolectomy	4666351000006113	175268008	271403011
Ischaemic bowel disease	3836731000006115	82196007	504939013
Thrombosis of central nervous system venous sinus NOS	296940016	192759008	296937016
Sagittal sinus thrombosis	3648391000006110	70607008	501619010
History of recurrent deep vein thrombosis	8044851000006112	141911000119100	3043613018
Thrombosed external haemorrhoid	2922231000006111	26373009	483208017
Venous thromboembolic disease	7098321000006117	429098002	2695294011
Deep venous thrombosis - leg	989221000006110	404223003	989221000006110
Cerebral venous thrombosis of lateral sinus	2838001000006118	21258007	1222620011
Ischaemic optic neuropathy	476238012	14357004	476238012
Pulmonary embolus	193601000006115	59282003	98484016
Mesenteric thrombosis	215511000000110	95446005	158099015

Post radiological embolism of upper limb artery	216711000006118	312377009	456079019
Thrombosis of cavernous venous sinus	3962581000006110	89980009	149150017
Unprovoked deep vein thrombosis	2488341000000112	978421000000101	2488341000000112
Ischaemic arm	5058031000006112	233959009	350538016
Thrombosis of transverse sinus	2837991000006111	21258007	35719015
Pulmonary arterial thrombosis	3439711000006111	57834008	96166019
Male genital thrombosis NOS	304536019	198057005	304524013
Ischaemic necrosis	3901731000006110	86217007	142974016
Vascular graft embolus	5925451000006112	304090004	446358019
Deep venous thrombosis of leg	6694761000006110	404223003	2162420015
Superior mesenteric artery embolus	4785631000006110	196999001	302978019
Embolus of central nervous system venous sinus	639231000006117	14246007	1221072019
Acute ischaemic colitis	503091015	75700000	503091015
Thyroid infarction	1220407012	11859008	1220407012
Embolism of central nervous system venous sinus	1221072019	14246007	1221072019
Capillary thrombosis	30091013	17810004	30091013
Acute infarction of papillary muscle	2663441000006117	10273003	17900011
Removal of vessel clot	866201000006119	392031002	866201000006119
Acute deep venous thrombosis of internal jugular vein	8042511000006119	135001000119100	2971631010
PVT - Portal vein thrombosis	2784351000006119	17920008	1221613018
Arteritic ischaemic optic neuropathy	5008941000006114	230508004	345412011
Peripheral ischaemic vascular disease	350535018	233958001	350535018
Upper limb ischaemia	350540014	233959009	350540014
Trendelenburg pulmonary embolectomy	271392011	175262009	271392011
Open embolectomy of bifurcation of aorta	271509013	175348000	271509013
Open embolectomy of carotid artery	271540018	175374006	271540018
Open embolectomy of axillary artery	271639012	175446000	271639012
Percutaneous transluminal embolectomy of subclavian artery	271654018	175456001	271654018
Percutaneous transluminal embolectomy of brachial artery	271656016	175457005	271656016
Percutaneous transluminal embolectomy of axillary artery	271671017	175465008	271671017
Percutaneous transluminal embolectomy of iliac artery	271901019	175613000	271901019
Open thrombectomy of femoral artery	272032018	175704008	272032018
Percutaneous transluminal embolectomy of femoral artery	272055015	175717006	272055015
Percutaneous transluminal embolectomy of popliteal artery	272056019	175718001	272056019
Prosthetic graft thrombectomy	272305017	175891000	272305017
Corpus cavernosum embolism	1231107016	52303006	1231107016
Vein graft thrombectomy	1233341011	71007004	1233341011
Cerebral vein thrombosis	1235913017	95455008	1235913017
Infarction of fallopian tube	94629010	56900004	94629010
Infarct of lung	3551761000006115	64662007	107460013

Cerebral arterial thrombosis	3662321000006117	71444005	118691019
Ischaemic foot	1699111000006110	1699111000006106	1699111000006110
Percutaneous mechanical thromboembolectomy	359731000000112	225291000000108	359731000000112
Unprovoked DVT (deep vein thrombosis)	8361011000006114	978421000000101	2518391000000114
Carotid thromboendarterectomy	3588601000006110	66951008	111209010
Saddle embolus of abdominal aorta	5058281000006118	233972005	350565011
Peripheral arterial embolism	884661000006110	583731000000103	884661000006110
Embolus/thrombus artery NOS	884671000006115	583761000000108	884671000006115
Venous embolism NOS	884741000006117	587851000000108	884741000006117
Embolism	887751000006110	198850008	887751000006110
Evacuation of thrombosed haemorrhoids	3984761000006115	91392002	509429011
Embolus of vein NOS	12719591000006119	587851000000108	1303841000000119
Arterial embolism and thrombosis NOS	12733161000006111	583761000000108	1295451000000114
Cholesterol embolus syndrome	354495010	236489002	354495010
Fogarty embolectomy of artery	1776773010	397047005	1776773010
Thrombosis - coronary	1786198013	398274000	1786198013
Central retinal vein occlusion	113737013	68478007	113737013
Thrombosis of cerebral arteries	3662311000006113	71444005	118690018
BRVT - Branch retinal vein thrombosis	2892681000006110	24596005	481963013
Thrombosed external pile	2922241000006118	26373009	483209013
Thromboendarterectomy of aorta	3295821000006118	49187005	81937019
PC TL embolectomy popliteal artery	4668191000006117	175718001	272057011
Percutaneous transluminal thrombolysis of vein with reconstruction	7098901000006114	429141007	2692268010
Right ventricular thrombus	5989831000006113	309518001	452962013
Thrombosis of testis	3605531000006118	67962003	112890010
Cerebral arterial embolism	3728451000006116	75543006	125471016
IRIS - Inner retinal ischaemic spots	3140901000006118	39832008	2620724014
Deep vein thrombosis of peroneal vein	1119161000000115	443210003	1664901000000117
Renal infarction	1848131000006113	45456005	75778012
[RFC] Venous thrombosis	905491000006112	905491000006108	905491000006112
Ischaemic nephropathy	1160091000000115	710565001	3082872011
Release of ischaemic contracture of forearm	4682421000006112	178253006	275890019
Open embolectomy of inferior mesenteric artery NEC	271760012	397045002	1776771012
Open embolectomy of suprarenal artery NEC	271761011	397045002	1776771012
Open embolectomy of visceral branch of abdominal aorta NEC	271780013	397045002	1776771012
Open embolectomy of artery NEC	272097014	397045002	1776771012
Open removal of thrombus from vein NOS	272248014	175843001	272243017
Open embolectomy of iliac artery	394332017	265521000	394332017
Renal artery thrombosis	158317012	95579008	158317012
Deep vein thrombosis of leg related to air travel	216205019	134399007	216205019
Thrombosis of lateral venous sinus	2837981000006113	21258007	35718011

Percutaneous thrombolysis of vein	5593341000006115	276985008	413389019
Brachial embolectomy	5048991000006113	233313002	349588014
Percutaneous transluminal embolectomy of artery NEC	394347014	397045002	1776771012
Cerebral venous thrombosis	1729331000006116	1729331000006100	1729331000006116
Embolism of corpus cavernosum	3347911000006112	52303006	87035015
Chronic intestinal ischaemia	4194521000006117	111354009	361442014
Deep vein thrombosis of portal vein	2784341000006116	17920008	1221612011
Embolism and thrombosis of other specified artery	300552011	266262004	395794011
Arterial embolism and thrombosis NOS	300564015	266262004	395794011
Infarction of thyroid	2688811000006110	11859008	20465014
Thrombosis of intracranial venous sinus	4768491000006118	192759008	1784812010
Thrombosis of penile vein	3745871000006116	76598006	127203016
Thrombosed hemorrhoid incised	5727411000006112	287799004	2871743015
Occlusive thromboarteriopathy	6206441000006110	359789008	475141015
Percutaneous transluminal thrombolysis of blood vessel of liver	1545591000006112	426059008	2675452017
Percutaneous transluminal thrombolysis and reconstruction of artery	1548401000006112	428068004	2696045014
Aortic bifurcation embolus	5058291000006115	233972005	350566012
External thrombosed hemorrhoids	2922201000006115	26373009	483203014
Acute colonic ischaemia	3730871000006110	75700000	2310141000000117
Thrombosis of cerebral veins	4056971000006118	95455008	158110019
Embolectomy	3668011000006113	71815002	119306015
Embolism and thrombosis of a leg artery NOS	300547018	195318006	300534011
Peripheral arterial embolism and thrombosis NOS	300550015	195318006	300534011
Renal artery embolism	158318019	95580006	158318019
Embolectomy of popliteal artery	5049001000006113	233314008	349592019
Multiple infarcts	2947401000006115	27876002	46655014
DVT - Deep vein thrombosis of lower limb	6694751000006113	404223003	2162419014
Infarction of kidney	3233381000006117	45456005	75780018
Acute deep vein thrombosis of lower limb	7966351000006118	651000119108	2967724010
Superficial venous thrombosis of leg	7103191000006117	429434005	2692641015
Ocular ischaemic syndrome	6045981000006117	314436005	458797013
Mesenteric embolism	412652016	276500007	412652016
Thrombosis of inferior vena cava	419640012	281595001	419640012
Thrombosis of vein of leg	453210011	309735004	453210011
Vertebrobasilar ischaemic vertigo	5034291000006111	232288005	348027016
Infarction	3403531000006115	55641003	92491010
Embolism in vascular graft	446359010	304090004	446359010
Thromboembolus of internal iliac artery	3503835018	734298005	3503835018
SMAE - Superior mesenteric artery embolus	4785641000006117	196999001	302979010
Thrombosis of stent of renal artery	1848121000006110	840961000000108	2182101000000113
Deep venous thrombosis of the popliteal vein	7078601000006114	427776007	2691844011

Thrombosis of vein of lower leg	5059271000006110	234043001	2820167012
Saddle embolus of pulmonary artery	8106001000006111	328511000119109	3322848011
Popliteal artery thrombosis	3918361000006110	87239004	144656012
Saddle embolus	3108321000006117	37778000	63025019
Arterial embolism	3387371000006112	54687002	90875013
Brachiocephalic vein thrombosis	5653441000006111	281597009	419642016
Stroke due to basilar artery thrombus	3508956015	329641000119104	3508956015
Femoral embolectomy	2634331000006113	8409001	1234899012
Hemopericardium due to and following acute myocardial infarction	12220751000006113	194862000	3673281015
Penile venous thrombosis	3745891000006115	76598006	3036947013
Thrombosed external haemorrhoids	2922181000006116	26373009	196520016
Cerebral infarction due to occlusion of precerebral artery	542261000006114	125081000119106	3042974014
Deep vein thrombosis	2162148012	128053003	2162149016
Carotid artery thrombosis	100771000006112	86003009	142588012
Occlusive mesenteric ischaemia	5086201000006115	235842000	353530010
Multiple lacunar infarcts	5967391000006111	307363008	450609012
Venous thrombosis, phlebitis and thrombophlebitis	6018501000006115	312585004	456332011
Thyroid hemorrhage and infarction	4757961000006112	190305000	292442019
Non-occlusive thrombus	2927841000006117	26713004	44719012
Open embolectomy of coeliac artery NEC	271757017	397045002	1776771012
Multifocal infarct	3474711000006110	60007006	99691019
Infarction of spleen	38632019	22996003	1223010014
Pulmonary thromboembolism	5057591000006116	233935004	350497014
Thrombectomy	3205401000006110	43810009	73061013
Thrombosis of superior sagittal sinus	3648381000006112	70607008	117287016
Ischaemic hepatitis	5086761000006113	235877000	353591011
Ischaemic gangrene	6670921000006117	402861007	1773672019
Suspected pulmonary embolism	2549664019	417113001	2549664019
Pulmonary thromboendarterectomy	2675439010	426900002	2675439010
Intracranial venous thrombosis	5836951000006117	297157005	437623015
Thrombosis of atrium, auricular appendage, and ventricle due to and following acute myocardial infarction	12220791000006119	194868001	3673277015
Recurrent DVT (deep vein thrombosis)	7665071000006116	710167004	2496081000000115
Lacunar infarction	299342019	230698000	345651012
Venous embolism	300975010	234049002	350676017
Embolectomy of iliac artery	5153821000006115	240928009	360865017
Embolectomy of femoral artery	2634321000006110	8409001	14892019
Ischaemic ulcer of toe	7108391000006114	429768000	2693575017
Acute deep venous thrombosis of femoral vein	8041581000006116	132291000119106	2971883017
Removal of thrombus of arteriovenous fistula	7081801000006114	427992007	2691808018
Ischemic infarction of muscle	5143791000006114	240126009	359749018

Thyroid haemorrhage or infarction NOS	292445017	190305000	292443012
Thromboangiitis obliterans NOS	300503012	52403007	87208017
Embolism and thrombosis of an arm artery NOS	300538014	195318006	300534011
Thrombosis	7218941000006118	439127006	2794470015
Clotted venous saccule	5086421000006112	235854000	353554017
Venous thrombosis	395803017	111293003	178522012
Thrombosis	5477661000006119	264579008	393059012
Saddle embolus	350563016	233972005	350563016
Infarction of liver	2783891000006116	17890003	1221609013
Provoked DVT (deep vein thrombosis)	8361031000006115	978441000000108	2518461000000111
Femoral artery embolus	5058301000006119	233973000	350567015
Critical lower limb ischaemia	5058111000006119	233962007	350547012
Subclavian artery thrombosis	5836921000006114	297154003	437620017
Lacunar infarct	3818181000006114	81037000	134436018
Ischaemia of feet	5886711000006111	300917007	442114010
Embolism and/or thrombosis of the external iliac artery	300555013	734299002	3503839012
Renal infarct	3233391000006119	45456005	75781019
Embolectomy of carotid artery	5902211000006114	302053004	443591015
Embolism of vein NOS	12759341000006113	587851000000108	1303861000000118
Lower limb arterial embolus	6015571000006117	312378004	456080016
Embolectomy of lower limb artery	3688071000006119	73038007	121315013
Iliofemoral deep vein thrombosis	5059301000006112	234044007	3464381018
Thrombolysis of vein	5050621000006118	233428003	349770015
Thrombolysis of artery	5049421000006111	233335005	349633016
Thyroid haemorrhage and infarction	292443012	190305000	292443012
Embolism cavernous sinus	296932010	192754003	296932010
Embolism lateral sinus	296934011	192756001	296934011
Embolism and thrombosis of an arm or leg artery	300534011	195318006	300534011
Embolism and/or thrombosis of the common iliac artery	300553018	195335005	300553018
Embolism and thrombosis of the splenic artery	300558010	195340002	300558010
Thrombophlebitis of the femoral vein	300672010	195410000	300672010
Thrombophlebitis of the anterior tibial vein	300674011	195412008	300674011
History of thromboembolism	5497011000006116	266996004	2986452015
Acute intestinal ischaemia	3986461000006117	91489000	509473010
History of deep vein thrombosis	4540551000006119	161508001	2986938011
Incision of thrombosed hemorrhoid	3984721000006114	91392002	151416013
Post-radiological embolism of upper limb artery	6015561000006112	312377009	456079019
Colonic ischaemia	2992791000006113	30588004	1216341014
Cerebral venous thrombosis of cavernous sinus	100691000006118	89980009	1235519010
Cerebral venous sinus thrombosis	100721000006111	192759008	296937016
Superior mesenteric vein thrombosis	100821000006116	197001004	302981012

Postoperative deep vein thrombosis	216571000006116	213220000	325066018
Postoperative pulmonary embolus	216591000006115	194883006	299847015
Ischaemic priapism	7252011000006114	441575009	2817057019
Central retinal vein thrombosis	3614331000006110	68478007	501028012
Digital arterial thrombosis	5886741000006110	300919005	442117015
Cerebral infarction due to cerebral venous thrombosis, non-pyogenic	542251000006112	195230003	300393016
Percutaneous transluminal venous thrombolysis NEC	358041000000114	276985008	413389019
Percutaneous removal of thrombus from vein NOS	359571000000118	276977004	413381016
Other specified percutaneous removal of thrombus from vein	359771000000114	276977004	413381016
Evacuation of thrombosed hemorrhoid	3984741000006119	91392002	509427013
Lower limb ischaemia	5058071000006110	233961000	350544017
Arterial embolus	3387391000006113	54687002	497013010
Embolism	3402611000006115	55584005	92392016
Chronic deep vein thrombosis of left iliac vein	12107671000006115	293461000119100	3646306012
Splenic vein thrombosis	2730141000006117	14534009	24693011
Percutaneous transluminal embolectomy of bifurcation of aorta	232881000006113	175356002	271519019
Branch retinal vein thrombosis	2892651000006119	24596005	481960011
Embolus/thrombosis abd. aorta	884641000006111	266263009	884641000006111
Embolus/thrombosis aorta NOS	884651000006113	195317001	884651000006113
Removal of thrombus from intracranial artery	2845481000006110	21710002	36433012
History of thromboembolism of vein	7095381000006112	428904003	2695935017
Percutaneous embolectomy of brachial artery	4667301000006111	175457005	271655017
Transluminal pulmonary embolectomy	4666341000006111	175268008	271402018
Acute mesenteric ischaemia	457631000006114	91489000	509474016
Mesenteric infarction	523601000006115	3558002	7001012
Deep venous thrombosis of deep femoral vein	7078581000006116	427775006	2695171017
Embolism and thrombosis of other and unspec parts aorta	638871000006114	274101000	409861010
Embolus of the superior mesenteric artery	639241000006110	196999001	302978019
Thrombosed external hemorrhoid	2922211000006117	26373009	483205019
Pulmonary infarction	728841000006111	64662007	107459015
Thrombosed internal haemorrhoids	770161000006115	52931009	198419016
Percutaneous transluminal thrombectomy of blood vessel of liver	1545581000006114	428953003	2692834012
Percutaneous transluminal venous thrombolysis with reconstruction	1549761000006110	429141007	1659501000000119
Retinal ischaemia	483237018	26468004	483237018
Ischaemic	5360051000006112	255426005	380631011
Asymptomatic ischaemia	5056261000006112	233823002	350356015
History of pulmonary embolus	4540611000006119	161512007	2986421016
Central retinal vein occlusion - ischaemic	6024101000006114	312997008	456819013
Embolism of iliac artery	3807321000006113	80383008	133386017
Fogarty embolectomy of vein	1776763013	397035004	1776763013

Arterial embolus and thrombosis	395795012	266262004	395795012
Occlusive thrombus	3771791000006113	78195007	129763011
Pulmonary embolism with pulmonary infarction	7967281000006119	1001000119102	2967075019
Non-pyogenic venous sinus thrombosis	586241000006116	195229008	300392014
Arterial ischaemia	5057951000006115	233957006	350532015
Intracranial thrombectomy	2845491000006113	21710002	36434018
Deep vein thrombosis, leg	218551000000118	266267005	395801015
Deep venous thrombosis of the superior mesenteric vein	4785671000006113	197001004	302982017
Acute ischaemia of small intestine	8321371000006112	906191000000102	2329961000000119
Rupture of chordae tendinae due to and following acute myocardial infarction	12220781000006117	194866002	3673278013
Central retinal vein occlusion - non-ischaemic	6024121000006116	312998003	456822010
Ischaemic finger	5967971000006111	307409006	450668018
Ischaemic ulcer	2721231000006112	13954005	475615013
Mural thrombosis	1235655012	91335003	1235655012
Ischaemic leg ulcer	357895013	238793001	357895013
Ischaemic foot ulcer	5126451000006116	238794007	357897017
Left ventricular thrombus	748061000006117	309519009	452964014
Ischaemia	3353921000006110	52674009	496400017
Acute deep venous thrombosis	8041571000006119	132281000119108	2968256017
Embolism and thrombosis of the iliac artery unspecified	300556014	266262004	395794011
[X]Embolism and thrombosis of other arteries	300964011	266262004	395794011
Vessel incision - embolectomy	866261000006118	597641000000105	866261000006118
Venous thrombosis NOS	884751000006115	690131000000103	884751000006115
Pulmonary embolus care	6452821000006114	386280004	1480446016
Thrombosis of pelvic vein	4056781000006117	95448006	158101010
Thrombosis of superior longitudinal sinus	296938014	192760003	296938014
Embolism and thrombosis of the anterior tibial artery	300541017	195325004	300541017
Embolism and thrombosis of the axillary artery	300559019	195341003	300559019
Deep venous thrombosis	2162149016	128053003	194647015
Embolectomy of subclavian artery	5888791000006119	301080008	442323018
[X] Embolism from prosthetic heart valve	358931000006111	35688006	59527012
Acute massive pulmonary embolism	5057621000006119	233936003	350502011
Omental infarction	3722741000006113	75204008	124899011
Embolism and thrombosis of other arteries NOS	300563014	266262004	395794011
Acute atrial infarction	299719012	194809007	299719012
Atrial thrombosis	300234010	195147006	300234010
Embolism and thrombosis of the radial artery	300536013	195320009	300536013
Embolism and thrombosis of the coeliac artery	300561011	195342005	300561011
Embolism and thrombosis of the vena cava	300710016	195437003	300710016
History of embolism	5574351000006111	275545002	2986809014
Provoked deep vein thrombosis	2488381000000116	978441000000108	2488381000000116

Acute deep vein thrombosis of left iliac vein	12107691000006119	293491000119107	3644949016
Cerebral venous thrombosis	4056981000006115	95455008	158111015
Arterial embolism/thrombosis	884631000006118	266262004	884631000006118
Thromboembolism	6348271000006116	371039008	1228915016
Percutaneous aspiration thromboembolectomy	359871000000116	225361000000106	359871000000116
Thrombectomy of arteriovenous fistula	360031000000113	427992007	2694416018
Ischaemic leg	742481000006118	233961000	350546015
Percutaneous venous thrombectomy	1549601000006119	276977004	413381016
Arterial embolectomy	6580991000006118	397045002	1776771012
Peripheral arterial embolism and thrombosis NOS	12733151000006114	583731000000103	1295391000000110
Thrombosis of vein NOS	12728641000006110	690131000000103	1510411000000110
Embolus of vein	5059381000006115	234049002	350677014
Incision of thrombosed haemorrhoid	3984711000006118	91392002	201577017
Thrombectomy of venous graft	3654851000006117	71007004	117941017
Transjugular intrahepatic thrombectomy of portal vein	2691870012	429788001	2691870012
Thrombectomy of vein	6580651000006116	397028007	1776761010
Thrombus	6568121000006118	396339007	1776291018
H/O: pulmonary embolus	251693011	161512007	251693011
Recurrent deep vein thrombosis	2144741000000111	710167004	3043650017
Open embolectomy of subclavian artery	271633013	175440006	271633013
Multi-infarct state	5011261000006110	230704000	345662012
Thrombosis of renal artery	4059051000006119	95579008	158316015
Deep venous thrombosis of lower limb	6694771000006115	404223003	2162421016
Thrombosis of vein of lower limb	5992311000006119	309735004	453209018
Aortic thromboembolism	5560171000006116	274101000	409861010
Incision and evacuation of thrombosed hemorrhoid	5727391000006112	287799004	2547808014
Femoral artery thrombosis	3345371000006113	52156004	86808015
Internal thrombosed piles	884851000006114	52931009	884851000006114
Open embolectomy of artery NEC	12723621000006114	597641000000105	1324221000000116
Ischaemic colitis	12705101000006115	30588004	484643015
Embolism and thrombosis of the abdominal aorta	395796013	266263009	395796013
Open embolectomy of common iliac artery	411214011	275254000	411214011
Right ventricular thrombosis	452963015	309518001	452963015
H/O: embolism	411539015	275545002	411539015
External thrombosed haemorrhoids	483206018	26373009	483206018
Infarction of ovary	6297018	3129009	6297018
Renal infarction	75778012	45456005	75778012
Other embolism and thrombosis	300712012	429098002	2692478010
Embolus of vein NOS	300714013	234049002	350676017
Mesenteric embolus NOS	302983010	276500007	412653014
Thrombosis of external jugular vein	2234411000000115	864211000000100	2234411000000115
Thrombosis of artery of transplanted kidney	2234541000000115	864271000000105	2234541000000115

Excision of external thrombotic haemorrhoid	2591991000006114	5796001	194958014
Percutaneous embolectomy of popliteal artery	4668171000006118	175718001	272058018
Chronic thromboembolic pulmonary hypertension	5057801000006113	233947005	2968345015
Incision and evacuation of thrombosed haemorrhoid	5727401000006114	287799004	2547809018
Critical ischaemia of foot	6021731000006115	312822006	456609017
Splenic infarction	2866521000006111	22996003	38632019
[RFC] Deep vein thrombosis	909471000006114	909471000006105	909471000006114
Thrombosed haemorrhoid - incised	914791000006117	287799004	427359014
Thromboembolic pulmonary hypertension	350517010	233947005	350517010
Percutaneous transluminal embolectomy of renal artery	271705017	175489001	271705017
Open embolectomy of femoral artery	272031013	175704008	272031013
Open femoral embolectomy	272033011	175704008	272033011
Open vein thrombectomy	272244011	175843001	272244011
Embolism of vein NOS	1222321017	234049002	350676017
[V] Personal history of pulmonary embolism	451479013	161512007	251693011
Percutaneous thrombolysis of intracranial artery	5014421000006115	230935003	345980018
Vertebral artery thrombosis	3553621000006119	64775002	107661016
Embolism of intracranial venous sinus	2725621000006114	14246007	24228018
Embolism superior longitudinal sinus	296933017	192755002	296933017
Embolism transverse sinus	296935012	192757005	296935012
Myelopathy due to arterial thrombosis of spinal cord	297138017	192898007	297138017
Embolism and thrombosis of the thoracic aorta	300533017	195317001	300533017
Embolism and thrombosis of the ulnar artery	300537016	195321008	300537016
Embolism and thrombosis of the popliteal artery	300540016	195324000	300540016
Other specified open removal of thrombus from vein	272247016	175843001	272243017
Thrombosed aneurysm	4374441000006113	125271003	193761013
Thrombosis of vein of transplanted kidney	2234621000000111	864311000000105	2234621000000111
Thrombosed external hemorrhoids	2922191000006118	26373009	44177012
LACI - Lacunar infarction	5011171000006110	230698000	345653010
Portal vein thrombosis	30273012	17920008	30273012
Pulmonary embolism	98484016	59282003	98484016
Infarction of breast	128289017	77296004	128289017
Thrombotic infarction	3756351000006111	77255002	128233014
Microvascular embolism of arteriole	7855891000006114	724439005	3438135014
Percutaneous thrombolysis of artery	5593331000006113	276984007	413388010
Ischaemic foot pain when walking	2377911000000114	713674006	3295993015
Thrombosed haemorrhoids	300748014	75955007	200560013
Thrombosis of mesenteric vein	302984016	95446005	158099015
Thromboembolism of vein	300704016	429098002	2692478010
Embolism central nervous system venous sinus NOS	296936013	14246007	24228018
Embolism and thrombosis of the hepatic artery	300562016	195343000	3491726018

Thrombotic microangiopathy NOS	300587011	126729006	135665013
Embolism and thrombosis NOS	300713019	429098002	2692478010
Thrombosis of internal jugular vein	2234371000000119	864191000000104	2234371000000119
Ischaemic foot pain at rest	2377871000000112	713412006	3289284017
Hepatic infarction	30226018	17890003	30226018
Thromboangiitis obliterans	87208017	52403007	87208017
Cardiac rupture due to and following acute myocardial infarction	12221301000006119	233847009	3673284011
DVT - Deep vein thrombosis	4403261000006113	128053003	2162148012
Thrombosis of corpus cavernosum	3215871000006116	44437005	74102013
Anterior spinal artery thrombosis	345723011	230740003	345723011
Peripheral ischaemia	350533013	233958001	350533013
Thrombosis of retinal vein	3244261000006119	46085004	76829016
Nephropathy due to ischaemia	7674011000006115	710565001	3044790019
Irreversible ischaemic colitis	2936461000006116	27241008	483469013
Axillary vein thrombosis	851241000006115	851241000006104	851241000006115
Embolus due to internal prosthetic device	324950011	213131003	324950011
Pulmonary infarct	499917019	64662007	499917019
Thromboembolic disease	6348281000006118	371039008	1228916015
Perianal venous thrombosis	2608401000000118	449815008	2608401000000118
Hepatic vein thrombosis	64466019	82385007	136654014
Infarct	3403521000006118	55641003	92488010
Deep vein thrombosis of lower limb	2162422011	404223003	2162422011
Retinal embolus	6702781000006116	404667009	2156561018
Embolism and thrombosis of hepatic artery	4778991000006118	195343000	3491726018
Arteriovenous fistula thrombosis	350892013	234205007	350892013
Renal artery stent thrombosis	1809151000006116	1809151000006100	1809151000006116
Superior mesenteric artery thrombosis	100811000006112	197000003	302980013
H/O: thromboembolism	217601000000119	266996004	397830014
Choroidal infarct	6041261000006115	314003000	458273015
Thromboembolus of external iliac artery	3503839012	734299002	3503839012
Acute lacunar infarction	7054171000006115	426107000	2674093010
Acute infarct	3400671000006111	55470003	92217011
Acute pulmonary embolism	7609581000006110	706870000	3027244010
Superficial femoral embolectomy	6019821000006117	312689003	456443016
Iliac vein thrombosis	3636257010	762256003	3636257010
Reason for referral: Deep Vein Thrombosis	1776621000006117	1776621000006101	1776621000006117
Percutaneous embolectomy of pulmonary artery	4666331000006118	175268008	271401013
Percutaneous embolectomy of aortoiliac segment	4666711000006113	175356002	271520013
Embolism and thrombosis NOS	638821000006113	429098002	2692478010
Retinal vein occlusion	3244281000006112	46085004	494432015
Thrombosis of vessels of cord	2962491000006118	28773001	48182014
Embolectomy operation	5559451000006118	274021001	409761015

Thrombosis of subclavian vein	2795114019	438647008	2795114019
History of thrombosis	5574371000006118	275546001	2986432016
Ischaemic nephropathy	1757951000006119	1757951000006103	1757951000006119
Arterial thrombosis	218541000000116	65198009	108347015
Thromboendarterectomy	866221000006112	392031002	866221000006112
Open thrombectomy of popliteal artery	272034017	175705009	272034017
Open embolectomy popliteal artery	272035016	175705009	272035016
Open thrombectomy of vein of lower limb	272246013	175845008	272246013
Declotting of thigh vein loop	272283013	175873005	272283013
High probability of deep vein thrombosis	1141351000000111	513291000000109	1141351000000111
Arterial embolism and thrombosis	491241000006118	266262004	395795012
Corpus cavernosum embolus	1231106013	52303006	1231106013
Blood clot	3731671000006117	75753009	125815012
Retinal microembolism	169951000006111	76975009	127782012
Cerebral venous thrombosis of sigmoid sinus	5011651000006111	230721009	345698016
Evacuation of thrombosed hemorrhoids	3984731000006112	91392002	151417016
Thrombosed hemorrhoids	3735111000006119	75955007	126159013
PE - Pulmonary embolism	3462921000006119	59282003	1231937012
Ischaemic lower limb pain at rest	8278561000006114	836711000000108	2172511000000111
H/O: thrombosis	411540018	275546001	411540018
Axillary vein thrombosis	437622013	297156001	437622013
Retinal vein thrombosis	494431010	46085004	494431010
Corpus cavernosum thrombosis	1230092014	44437005	1230092014
Renal artery embolus	1235925019	95580006	1235925019
Deep venous thrombosis of peroneal vein	7273741000006119	443210003	2839752017
Thrombosed hemorrhoid - incised	5727371000006111	287799004	427358018
Blood clot in eye	5233761000006119	246682000	368495017
Recurrent deep vein thrombosis	1786921000006116	1786921000006100	1786921000006116
Thrombosis of middle cerebral artery	7598991000006116	705129007	3023377010
Haemopericardium due to and following acute myocardial infarction	12220761000006110	194862000	3673282010
Penis vein thrombosis	503317010	76598006	503317010
Testicular thrombosis	1232993010	67962003	1232993010
H/O: Deep vein thrombosis	451478017	161508001	2547692019
[V] Personal history DVT (deep vein thrombosis)	1227369013	161508001	2547692019
Thrombosis lateral sinus	100701000006118	21258007	1222620011
Percutaneous transluminal thrombolysis of femoral graft using streptokinase	238621000006112	309449009	452880015
Open pulmonary embolectomy	261021000006119	175262009	271393018
Thrombosis of arteries of lower extremity	3978101000006118	90958004	150730010
Deep venous thrombosis - leg	884731000006110	266267005	884731000006110
External thrombosed piles	884881000006118	26373009	884881000006118
[RFC] Pulmonary embolism/pulmonary hypertension	905451000006118	905451000006102	905451000006118

[RFC] Arterial embolism of limbs	905541000006119	905541000006103	905541000006119
Evacuation of thrombosed haemorrhoid	649671000006118	91392002	509428015
Infarction of prostate	36968017	22035000	36968017
Spinal cord infarct	7156221000006114	432249006	2770833019
Post radiological embolism of lower limb artery	216701000006116	312380005	456083019
Percutaneous transluminal embolectomy of pulmonary artery	232951000006114	175268008	271401013
Renal vein thrombosis	2751261000006119	15842009	26843012
Ischaemic infarction of muscle	359748014	240126009	359748014
Arterial embolic and thrombotic occlusion	395794011	266262004	395794011
CRVO - Central retinal vein occlusion	3614321000006112	68478007	501027019
Limb ischaemia	8009281000006110	21631000119105	2923092011
Open embolectomy of superior mesenteric artery NEC	271759019	397045002	1776771012
Embolism and thrombosis of the femoral artery	300539018	195323006	300539018
Embolism and thrombosis of the dorsalis pedis artery	300542012	195326003	300542012
Embolism and thrombosis of the subclavian artery	300557017	195339004	300557017
Embolism and thrombosis of the renal vein	300711017	195438008	300711017
Scrotal thrombosis	304527018	198059008	304527018
H/O: Deep Vein Thrombosis	251689017	161508001	251689017
Percutaneous embolectomy of femoral artery	4668151000006111	175717006	272054016
Thromboembolic pulmonary hypertension	1749291000006113	1749291000006109	1749291000006113
Post-radiological embolism of lower limb artery	6015601000006112	312380005	456083019
Thrombosed internal hemorrhoids	3358351000006111	52931009	88072011
Infarct of prostate	2850821000006117	22035000	1222704012
Superficial vein thrombosis	5574011000006113	275517008	411509013
Open embolectomy of brachial artery	271635018	175442003	271635018
Open embolectomy of vertebral artery	271636017	175443008	271636017
Percutaneous transluminal embolectomy of vertebral artery	271657013	175458000	271657013
Open embolectomy of renal artery	271695015	175482005	271695015
Open removal of thrombus from vein	272243017	175843001	272243017
Open thrombectomy of vein of upper limb	272245012	175844007	272245012

Table S30. Arterial thromboembolic Aurum codes

Term	Medcode ID	SNOMED CT Concept ID	SNOMED CT Description ID
Embolism and/or thrombosis of the internal iliac artery	300554012	734298005	3503835018
Coronary thrombosis not resulting in myocardial infarction	299742017	194821006	299742017
Embolism and thrombosis of the brachial artery	300535012	195319003	300535012
Embolism and thrombosis of the posterior tibial artery	300543019	195327007	300543019
Mesenteric embolus	412653014	276500007	412653014
Ischaemic foot	443199013	301755001	443199013
Open removal of cardiac thrombus	2675367019	426067000	2675367019
Percutaneous transluminal thrombolysis of artery	2675878019	426485003	2675878019
Ischaemic disease of gut	3836741000006113	82196007	504940010
Acute intestinal ischaemic syndrome	3986491000006113	91489000	509476019
Intestinal ischaemia	3836721000006118	82196007	504938017
Embolism and thrombosis of the celiac artery	4778981000006116	195342005	300560012
Ischaemia reperfusion injury of liver	7821701000006113	721715000	3325835015
AMI - Acute mesenteric ischaemia	3986481000006110	91489000	509475015
Inner retinal ischaemic spots	3140891000006117	39832008	2620723015
Thrombosis of renal artery bypass graft	449993015	306857000	449993015
Ischaemic toe	450665015	307408003	450665015
Mesenteric thrombus and/or embolus	450682018	307420006	450682018
Infarct of liver	2783881000006119	17890003	30227010
Ischaemic bowel disease	3836731000006115	82196007	504939013
Ischaemic optic neuropathy	476238012	14357004	476238012
Mesenteric thrombosis	215511000000110	95446005	158099015
Post radiological embolism of upper limb artery	216711000006118	312377009	456079019
Ischaemic arm	5058031000006112	233959009	350538016
Pulmonary arterial thrombosis	3439711000006111	57834008	96166019
Male genital thrombosis NOS	304536019	198057005	304524013
Ischaemic necrosis	3901731000006110	86217007	142974016
Vascular graft embolus	5925451000006112	304090004	446358019
Superior mesenteric artery embolus	4785631000006110	196999001	302978019
Acute ischaemic colitis	503091015	75700000	503091015
Thyroid infarction	1220407012	11859008	1220407012
Acute infarction of papillary muscle	2663441000006117	10273003	17900011
Removal of vessel clot	866201000006119	392031002	866201000006119
Arteritic ischaemic optic neuropathy	5008941000006114	230508004	345412011
Peripheral ischaemic vascular disease	350535018	233958001	350535018
Upper limb ischaemia	350540014	233959009	350540014
Open embolectomy of bifurcation of aorta	271509013	175348000	271509013
Open embolectomy of carotid artery	271540018	175374006	271540018
Open embolectomy of axillary artery	271639012	175446000	271639012

Percutaneous transluminal embolectomy of subclavian artery	271654018	175456001	271654018
Percutaneous transluminal embolectomy of brachial artery	271656016	175457005	271656016
Percutaneous transluminal embolectomy of axillary artery	271671017	175465008	271671017
Percutaneous transluminal embolectomy of iliac artery	271901019	175613000	271901019
Open thrombectomy of femoral artery	272032018	175704008	272032018
Percutaneous transluminal embolectomy of femoral artery	272055015	175717006	272055015
Percutaneous transluminal embolectomy of popliteal artery	272056019	175718001	272056019
Prosthetic graft thrombectomy	272305017	175891000	272305017
Infarction of fallopian tube	94629010	56900004	94629010
Infarct of lung	3551761000006115	64662007	107460013
Cerebral arterial thrombosis	3662321000006117	71444005	118691019
Ischaemic foot	1699111000006110	1699111000006106	1699111000006110
Percutaneous mechanical thromboembolectomy	359731000000112	225291000000108	359731000000112
Carotid thromboendarterectomy	3588601000006110	66951008	111209010
Saddle embolus of abdominal aorta	5058281000006118	233972005	350565011
Peripheral arterial embolism	884661000006110	583731000000103	884661000006110
Embolus/thrombus artery NOS	884671000006115	583761000000108	884671000006115
Embolism	887751000006110	198850008	887751000006110
Arterial embolism and thrombosis NOS	12733161000006111	583761000000108	1295451000000114
Cholesterol embolus syndrome	354495010	236489002	354495010
Fogarty embolectomy of artery	1776773010	397047005	1776773010
Thrombosis - coronary	1786198013	398274000	1786198013
Thrombosis of cerebral arteries	3662311000006113	71444005	118690018
Thromboendarterectomy of aorta	3295821000006118	49187005	81937019
PC TL embolectomy popliteal artery	4668191000006117	175718001	272057011
Thrombosis of testis	3605531000006118	67962003	112890010
Cerebral arterial embolism	3728451000006116	75543006	125471016
IRIS - Inner retinal ischaemic spots	3140901000006118	39832008	2620724014
Renal infarction	1848131000006113	45456005	75778012
Ischaemic nephropathy	1160091000000115	710565001	3082872011
Release of ischaemic contracture of forearm	4682421000006112	178253006	275890019
Open embolectomy of inferior mesenteric artery NEC	271760012	397045002	1776771012
Open embolectomy of suprarenal artery NEC	271761011	397045002	1776771012
Open embolectomy of visceral branch of abdominal aorta NEC	271780013	397045002	1776771012
Open embolectomy of artery NEC	272097014	397045002	1776771012
Open embolectomy of iliac artery	394332017	265521000	394332017
Renal artery thrombosis	158317012	95579008	158317012
Percutaneous thrombolysis of vein	5593341000006115	276985008	413389019
Brachial embolectomy	5048991000006113	233313002	349588014

Percutaneous transluminal embolectomy of artery NEC	394347014	397045002	1776771012
Chronic intestinal ischaemia	4194521000006117	111354009	361442014
Embolism and thrombosis of other specified artery	300552011	266262004	395794011
Arterial embolism and thrombosis NOS	300564015	266262004	395794011
Infarction of thyroid	2688811000006110	11859008	20465014
Occlusive thromboarteriopathy	6206441000006110	359789008	475141015
Percutaneous transluminal thrombolysis of blood vessel of liver	1545591000006112	426059008	2675452017
Percutaneous transluminal thrombolysis and reconstruction of artery	1548401000006112	428068004	2696045014
Aortic bifurcation embolus	5058291000006115	233972005	350566012
Acute colonic ischaemia	3730871000006110	75700000	2310141000000117
Embolectomy	3668011000006113	71815002	119306015
Embolism and thrombosis of a leg artery NOS	300547018	195318006	300534011
Peripheral arterial embolism and thrombosis NOS	300550015	195318006	300534011
Renal artery embolism	158318019	95580006	158318019
Embolectomy of popliteal artery	5049001000006113	233314008	349592019
Multiple infarcts	2947401000006115	27876002	46655014
Infarction of kidney	3233381000006117	45456005	75780018
Ocular ischaemic syndrome	6045981000006117	314436005	458797013
Mesenteric embolism	412652016	276500007	412652016
Vertebrobasilar ischaemic vertigo	5034291000006111	232288005	348027016
Infarction	3403531000006115	55641003	92491010
Embolism in vascular graft	446359010	304090004	446359010
Thromboembolus of internal iliac artery	3503835018	734298005	3503835018
SMAE - Superior mesenteric artery embolus	4785641000006117	196999001	302979010
Thrombosis of stent of renal artery	1848121000006110	840961000000108	2182101000000113
Popliteal artery thrombosis	3918361000006110	87239004	144656012
Arterial embolism	3387371000006112	54687002	90875013
Stroke due to basilar artery thrombus	3508956015	329641000119104	3508956015
Femoral embolectomy	2634331000006113	8409001	1234899012
Hemopericardium due to and following acute myocardial infarction	12220751000006113	194862000	3673281015
Cerebral infarction due to occlusion of precerebral artery	542261000006114	125081000119106	3042974014
Carotid artery thrombosis	100771000006112	86003009	142588012
Occlusive mesenteric ischaemia	5086201000006115	235842000	353530010
Multiple lacunar infarcts	5967391000006111	307363008	450609012
Thyroid hemorrhage and infarction	4757961000006112	190305000	292442019
Non-occlusive thrombus	2927841000006117	26713004	44719012
Open embolectomy of coeliac artery NEC	271757017	397045002	1776771012
Multifocal infarct	3474711000006110	60007006	99691019
Infarction of spleen	38632019	22996003	1223010014
Thrombectomy	3205401000006110	43810009	73061013

Ischaemic hepatitis	5086761000006113	235877000	353591011
Ischaemic gangrene	6670921000006117	402861007	1773672019
Thrombosis of atrium, auricular appendage, and ventricle due to and following acute myocardial infarction	12220791000006119	194868001	3673277015
Lacunar infarction	299342019	230698000	345651012
Embolectomy of iliac artery	5153821000006115	240928009	360865017
Embolectomy of femoral artery	2634321000006110	8409001	14892019
Ischaemic ulcer of toe	7108391000006114	429768000	2693575017
Removal of thrombus of arteriovenous fistula	7081801000006114	427992007	2691808018
Ischemic infarction of muscle	5143791000006114	240126009	359749018
Thyroid haemorrhage or infarction NOS	292445017	190305000	292443012
Thromboangiitis obliterans NOS	300503012	52403007	87208017
Embolism and thrombosis of an arm artery NOS	300538014	195318006	300534011
Thrombosis	7218941000006118	439127006	2794470015
Thrombosis	5477661000006119	264579008	393059012
Saddle embolus	350563016	233972005	350563016
Infarction of liver	2783891000006116	17890003	1221609013
Femoral artery embolus	5058301000006119	233973000	350567015
Critical lower limb ischaemia	5058111000006119	233962007	350547012
Subclavian artery thrombosis	5836921000006114	297154003	437620017
Lacunar infarct	3818181000006114	81037000	134436018
Ischaemia of feet	5886711000006111	300917007	442114010
Embolism and/or thrombosis of the external iliac artery	300555013	734299002	3503839012
Renal infarct	3233391000006119	45456005	75781019
Embolectomy of carotid artery	5902211000006114	302053004	443591015
Lower limb arterial embolus	6015571000006117	312378004	456080016
Embolectomy of lower limb artery	3688071000006119	73038007	121315013
Thrombolysis of artery	5049421000006111	233335005	349633016
Thyroid haemorrhage and infarction	292443012	190305000	292443012
Embolism and thrombosis of an arm or leg artery	300534011	195318006	300534011
Embolism and/or thrombosis of the common iliac artery	300553018	195335005	300553018
Embolism and thrombosis of the splenic artery	300558010	195340002	300558010
Acute intestinal ischaemia	3986461000006117	91489000	509473010
Post-radiological embolism of upper limb artery	6015561000006112	312377009	456079019
Colonic ischaemia	2992791000006113	30588004	1216341014
Ischaemic priapism	7252011000006114	441575009	2817057019
Digital arterial thrombosis	5886741000006110	300919005	442117015
Percutaneous transluminal venous thrombolysis NEC	358041000000114	276985008	413389019
Percutaneous removal of thrombus from vein NOS	359571000000118	276977004	413381016
Lower limb ischaemia	5058071000006110	233961000	350544017
Arterial embolus	3387391000006113	54687002	497013010

Embolism	3402611000006115	55584005	92392016
Percutaneous transluminal embolectomy of bifurcation of aorta	232881000006113	175356002	271519019
Embolus/thrombosis abd. aorta	884641000006111	266263009	884641000006111
Embolus/thrombosis aorta NOS	884651000006113	195317001	884651000006113
Removal of thrombus from intracranial artery	2845481000006110	21710002	36433012
Percutaneous embolectomy of brachial artery	4667301000006111	175457005	271655017
Acute mesenteric ischaemia	457631000006114	91489000	509474016
Mesenteric infarction	523601000006115	3558002	7001012
Embolism and thrombosis of other and unspec parts aorta	638871000006114	274101000	409861010
Embolus of the superior mesenteric artery	639241000006110	196999001	302978019
Percutaneous transluminal thrombectomy of blood vessel of liver	1545581000006114	428953003	2692834012
Percutaneous transluminal venous thrombolysis with reconstruction	1549761000006110	429141007	1659501000000119
Retinal ischaemia	483237018	26468004	483237018
Ischaemic	5360051000006112	255426005	380631011
Asymptomatic ischaemia	5056261000006112	233823002	350356015
Embolism of iliac artery	3807321000006113	80383008	133386017
Arterial embolus and thrombosis	395795012	266262004	395795012
Occlusive thrombus	3771791000006113	78195007	129763011
Arterial ischaemia	5057951000006115	233957006	350532015
Intracranial thrombectomy	2845491000006113	21710002	36434018
Acute ischaemia of small intestine	8321371000006112	906191000000102	2329961000000119
Rupture of chordae tendinae due to and following acute myocardial infarction	12220781000006117	194866002	3673278013
Ischaemic finger	5967971000006111	307409006	450668018
Ischaemic ulcer	2721231000006112	13954005	475615013
Mural thrombosis	1235655012	91335003	1235655012
Ischaemic leg ulcer	357895013	238793001	357895013
Ischaemic foot ulcer	5126451000006116	238794007	357897017
Left ventricular thrombus	748061000006117	309519009	452964014
Ischaemia	3353921000006110	52674009	496400017
Embolism and thrombosis of the iliac artery unspecified	300556014	266262004	395794011
[X]Embolism and thrombosis of other arteries	300964011	266262004	395794011
Vessel incision - embolectomy	866261000006118	597641000000105	866261000006118
Embolism and thrombosis of the anterior tibial artery	300541017	195325004	300541017
Embolism and thrombosis of the axillary artery	300559019	195341003	300559019
Embolectomy of subclavian artery	5888791000006119	301080008	442323018
[X] Embolism from prosthetic heart valve	358931000006111	35688006	59527012
Omental infarction	3722741000006113	75204008	124899011
Embolism and thrombosis of other arteries NOS	300563014	266262004	395794011
Acute atrial infarction	299719012	194809007	299719012
Atrial thrombosis	300234010	195147006	300234010

Embolism and thrombosis of the radial artery	300536013	195320009	300536013
Embolism and thrombosis of the coeliac artery	300561011	195342005	300561011
Arterial embolism/thrombosis	884631000006118	266262004	884631000006118
Thromboembolism	6348271000006116	371039008	1228915016
Percutaneous aspiration thromboembolectomy	359871000000116	225361000000106	359871000000116
Thrombectomy of arteriovenous fistula	360031000000113	427992007	2694416018
Ischaemic leg	742481000006118	233961000	350546015
Arterial embolectomy	6580991000006118	397045002	1776771012
Peripheral arterial embolism and thrombosis NOS	12733151000006114	583731000000103	1295391000000110
Thrombus	6568121000006118	396339007	1776291018
Open embolectomy of subclavian artery	271633013	175440006	271633013
Multi-infarct state	5011261000006110	230704000	345662012
Thrombosis of renal artery	4059051000006119	95579008	158316015
Aortic thromboembolism	5560171000006116	274101000	409861010
Femoral artery thrombosis	3345371000006113	52156004	86808015
Open embolectomy of artery NEC	12723621000006114	597641000000105	1324221000000116
Ischaemic colitis	12705101000006115	30588004	484643015
Embolism and thrombosis of the abdominal aorta	395796013	266263009	395796013
Open embolectomy of common iliac artery	411214011	275254000	411214011
H/O: embolism	411539015	275545002	411539015
Infarction of ovary	6297018	3129009	6297018
Renal infarction	75778012	45456005	75778012
Other embolism and thrombosis	300712012	429098002	2692478010
Mesenteric embolus NOS	302983010	276500007	412653014
Thrombosis of artery of transplanted kidney	2234541000000115	864271000000105	2234541000000115
Percutaneous embolectomy of popliteal artery	4668171000006118	175718001	272058018
Critical ischaemia of foot	6021731000006115	312822006	456609017
Splenic infarction	2866521000006111	22996003	38632019
Percutaneous transluminal embolectomy of renal artery	271705017	175489001	271705017
Open embolectomy of femoral artery	272031013	175704008	272031013
Open femoral embolectomy	272033011	175704008	272033011
Percutaneous thrombolysis of intracranial artery	5014421000006115	230935003	345980018
Vertebral artery thrombosis	3553621000006119	64775002	107661016
Myelopathy due to arterial thrombosis of spinal cord	297138017	192898007	297138017
Embolism and thrombosis of the thoracic aorta	300533017	195317001	300533017
Embolism and thrombosis of the ulnar artery	300537016	195321008	300537016
Embolism and thrombosis of the popliteal artery	300540016	195324000	300540016
Thrombosed aneurysm	4374441000006113	125271003	193761013
LACI - Lacunar infarction	5011171000006110	230698000	345653010
Infarction of breast	128289017	77296004	128289017
Thrombotic infarction	3756351000006111	77255002	128233014

Microvascular embolism of arteriole	7855891000006114	724439005	3438135014
Percutaneous thrombolysis of artery	5593331000006113	276984007	413388010
Ischaemic foot pain when walking	2377911000000114	713674006	3295993015
Embolism and thrombosis of the hepatic artery	300562016	195343000	3491726018
Thrombotic microangiopathy NOS	300587011	126729006	135665013
Embolism and thrombosis NOS	300713019	429098002	2692478010
Ischaemic foot pain at rest	2377871000000112	713412006	3289284017
Hepatic infarction	30226018	17890003	30226018
Thromboangiitis obliterans	87208017	52403007	87208017
Cardiac rupture due to and following acute myocardial infarction	12221301000006119	233847009	3673284011
Thrombosis of corpus cavernosum	3215871000006116	44437005	74102013
Anterior spinal artery thrombosis	345723011	230740003	345723011
Peripheral ischaemia	350533013	233958001	350533013
Nephropathy due to ischaemia	7674011000006115	710565001	3044790019
Irreversible ischaemic colitis	2936461000006116	27241008	483469013
Embolus due to internal prosthetic device	324950011	213131003	324950011
Thromboembolic disease	6348281000006118	371039008	1228916015
Infarct	3403521000006118	55641003	92488010
Retinal embolus	6702781000006116	404667009	2156561018
Embolism and thrombosis of hepatic artery	4778991000006118	195343000	3491726018
Arteriovenous fistula thrombosis	350892013	234205007	350892013
Renal artery stent thrombosis	1809151000006116	1809151000006100	1809151000006116
Superior mesenteric artery thrombosis	100811000006112	197000003	302980013
Choroidal infarct	6041261000006115	314003000	458273015
Thromboembolus of external iliac artery	3503839012	734299002	3503839012
Acute lacunar infarction	7054171000006115	426107000	2674093010
Acute infarct	3400671000006111	55470003	92217011
Superficial femoral embolectomy	6019821000006117	312689003	456443016
Percutaneous embolectomy of aortoiliac segment	4666711000006113	175356002	271520013
Embolism and thrombosis NOS	638821000006113	429098002	2692478010
Thrombosis of vessels of cord	2962491000006118	28773001	48182014
Embolectomy operation	5559451000006118	274021001	409761015
History of thrombosis	5574371000006118	275546001	2986432016
Ischaemic nephropathy	1757951000006119	1757951000006103	1757951000006119
Arterial thrombosis	218541000000116	65198009	108347015
Thromboendarterectomy	866221000006112	392031002	866221000006112
Open thrombectomy of popliteal artery	272034017	175705009	272034017
Open embolectomy popliteal artery	272035016	175705009	272035016
Arterial embolism and thrombosis	491241000006118	266262004	395795012
Retinal microembolism	169951000006111	76975009	127782012
Ischaemic lower limb pain at rest	8278561000006114	836711000000108	2172511000000111
Corpus cavernosum thrombosis	1230092014	44437005	1230092014

Renal artery embolus	1235925019	95580006	1235925019
Thrombosis of middle cerebral artery	7598991000006116	705129007	3023377010
Haemopericardium due to and following acute myocardial infarction	12220761000006110	194862000	3673282010
Testicular thrombosis	1232993010	67962003	1232993010
Percutaneous transluminal thrombolysis of femoral graft using streptokinase	238621000006112	309449009	452880015
Thrombosis of arteries of lower extremity	3978101000006118	90958004	150730010
[RFC] Arterial embolism of limbs	905541000006119	905541000006103	905541000006119
Infarction of prostate	36968017	22035000	36968017
Spinal cord infarct	7156221000006114	432249006	2770833019
Post radiological embolism of lower limb artery	216701000006116	312380005	456083019
Ischaemic infarction of muscle	359748014	240126009	359748014
Arterial embolic and thrombotic occlusion	395794011	266262004	395794011
Limb ischaemia	8009281000006110	21631000119105	2923092011
Open embolectomy of superior mesenteric artery NEC	271759019	397045002	1776771012
Embolism and thrombosis of the femoral artery	300539018	195323006	300539018
Embolism and thrombosis of the dorsalis pedis artery	300542012	195326003	300542012
Embolism and thrombosis of the subclavian artery	300557017	195339004	300557017
Scrotal thrombosis	304527018	198059008	304527018
Percutaneous embolectomy of femoral artery	4668151000006111	175717006	272054016
Post-radiological embolism of lower limb artery	6015601000006112	312380005	456083019
Infarct of prostate	2850821000006117	22035000	1222704012
Open embolectomy of brachial artery	271635018	175442003	271635018
Open embolectomy of vertebral artery	271636017	175443008	271636017
Percutaneous transluminal embolectomy of vertebral artery	271657013	175458000	271657013
Open embolectomy of renal artery	271695015	175482005	271695015

Table S31. Thromboembolic HES (ICD10) codes

ICD10 codes	Term
D73.5	Infarction of spleen
H34.0	Transient retinal artery occlusion
H34.1	Central retinal artery occlusion
H34.2	Other retinal artery occlusions
H34.8	Other retinal vascular occlusions
H34.9	Retinal vascular occlusion, unspecified
I23.6	Thrombosis of atrium, auricular appendage, and ventricle as current complications following acute myocardial infarction
I26.0	Pulmonary embolism with mention of acute cor pulmonale
I26.9	Pulmonary embolism without mention of acute cor pulmonale
I51.3	Intracardiac thrombosis, not elsewhere classified
I65.0	Occlusion and stenosis of vertebral artery
I65.1	Occlusion and stenosis of basilar artery
I65.2	Occlusion and stenosis of carotid artery
I65.3	Occlusion and stenosis of multiple and bilateral precerebral arteries
I65.8	Occlusion and stenosis of other precerebral artery
I65.9	Occlusion and stenosis of unspecified precerebral artery
I66.0	Occlusion and stenosis of middle cerebral artery
I66.1	Occlusion and stenosis of anterior cerebral artery
I66.2	Occlusion and stenosis of posterior cerebral artery
I66.3	Occlusion and stenosis of cerebellar arteries
I66.4	Occlusion and stenosis of multiple and bilateral cerebral arteries
I66.8	Occlusion and stenosis of other cerebral artery
I66.9	Occlusion and stenosis of unspecified cerebral artery
I67.6	Nonpyogenic thrombosis of intracranial venous system
I73.1	Thromboangiitis obliterans [Buerger]
I74.0	Embolism and thrombosis of abdominal aorta
I74.1	Embolism and thrombosis of other and unspecified parts of aorta
I74.2	Embolism and thrombosis of arteries of upper extremities
I74.3	Embolism and thrombosis of arteries of lower extremities
I74.4	Embolism and thrombosis of arteries of extremities, unspecified
I74.5	Embolism and thrombosis of iliac artery
I74.8	Embolism and thrombosis of other arteries
I74.9	Embolism and thrombosis of unspecified artery
I80.0	Phlebitis and thrombophlebitis of superficial vessels of lower extremities
I80.1	Phlebitis and thrombophlebitis of femoral vein
I80.2	Phlebitis and thrombophlebitis of other deep vessels of lower extremities
I80.3	Phlebitis and thrombophlebitis of lower extremities, unspecified
I80.8	Phlebitis and thrombophlebitis of other sites
I80.9	Phlebitis and thrombophlebitis of unspecified site

I81	Portal vein thrombosis
I82.1	Thrombophlebitis migrans
I82.2	Embolism and thrombosis of vena cava
I82.3	Embolism and thrombosis of renal vein
I82.8	Embolism and thrombosis of other specified veins
I82.9	Embolism and thrombosis of unspecified vein
I84.0	Internal thrombosed haemorrhoids
I84.3	External thrombosed haemorrhoids
I84.7	Unspecified thrombosed haemorrhoids
K55.0	Acute vascular disorders of intestine
K55.9	Vascular disorder of intestine, unspecified
K64.5	Perianal venous thrombosis
K76.3	Infarction of liver
M31.1	Thrombotic microangiopathy
M62.2	Ischaemic infarction of muscle
M62.20	Ischaemic infarction of muscle, unspecified site
M62.21	Ischaemic infarction of muscle, shoulder
M62.22	Ischaemic infarction of muscle, upper arm
M62.23	Ischaemic infarction of muscle, forearm
M62.24	Ischaemic infarction of muscle, hand
M62.25	Ischaemic infarction of muscle, thigh
M62.26	Ischaemic infarction of muscle, lower leg
M62.27	Ischaemic infarction of muscle, ankle and foot
M62.28	Ischaemic infarction of muscle, other site
N28.0	Ischaemia and infarction of kidney

Table S32. Myocardial infarction outcome Aurum codes

Term	Medcode ID	SNOMED CT Concept ID	SNOMED CT Description ID
Electrocardiogram: lateral infarction	4586241000006113	164871004	2619220010
Acute anteroapical infarction	299708014	52035003	3038718019
Subsequent myocardial infarction of inferior wall	299812011	194858006	299812011
Acute Q-wave infarct	447324018	304914007	447324018
Electrocardiographic subendocardial infarct	4586211000006114	164870003	3300442017
Acute transmural myocardial infarction of unspecif site	460681000006116	57054005	94884017
Postmyocardial infarction syndrome	3576371000006117	66189004	109915012
Acute non-Q wave infarction	450322013	307140009	450322013
H/O: Myocardial infarction in last year	451369010	308065005	451369010
Post infarct angina	458410010	314116003	458410010
ECG: antero-septal infarct.	256455012	164868007	256455012
Myocardial infarct	2855351000006111	22298006	1784873012
Acute myocardial infarction of inferolateral wall	3565871000006113	65547006	108912018
Acute inferior myocardial infarction	3699921000006110	73795002	1233665015
Other acute and subacute ischaemic heart disease	39111000006114	414545008	2534663012
Lateral infarction on electrocardiogram	4586221000006118	164871004	2692104017
Electrocardiographic myocardial infarction	4586041000006115	164865005	3300376010
Post-infarction pericarditis	5056941000006119	233885007	350431013
CT - Coronary thrombosis	6601131000006115	398274000	1786196012
Subsequent myocardial infarction of unspecified site	118831000006118	194856005	299808017
Postoperative transmural myocardial infarction other sites	212081000006112	129574000	208365015
Postoperative transmural myocardial infarction unspec site	212091000006110	129574000	208365015
Post infarction pericarditis	216351000006118	233885007	350431013
Acute myocardial infarction of atrium	4775891000006119	194809007	2470032018
Defect of ventricular septum following myocardial infarction	5056521000006115	233846000	3333538017
Acute myocardial infarction of septum	3784911000006111	79009004	131106018
EKG:posterior/inferior infarct	4586171000006112	164869004	3300251011
Aborted myocardial infarction	4775931000006111	194821006	2619483012
EKG: myocardial infarction	4586051000006118	164865005	3300377018
Other acute myocardial infarction NOS	299720018	57054005	94884017
History of myocardial infarction in last year	5974481000006116	308065005	2986726013
Acute papillary muscle infarction	1218860015	10273003	1218860015
Acute myocardial infarction of anterolateral wall	3641641000006116	70211005	116613011
Acute infarction of papillary muscle	2663441000006117	10273003	17900011
Acute Q wave myocardial infarction	5935321000006111	304914007	447323012
Silent myocardial infarction	350376014	233843008	350376014
Microinfarction of heart	1229885017	42531007	1229885017
Coronary artery thrombosis	6601121000006118	398274000	1777817018

EKG: subendocardial infarct	4586201000006111	164870003	3300441012
Coronary thrombosis	884141000006116	57054005	884141000006116
ECG: myocardial infarct NOS	256460011	164865005	256452010
Acute ST segment elevation myocardial infarction	1780491019	401303003	1780491019
Thrombosis - coronary	1786198013	398274000	1786198013
Electrocardiogram finding of infarction	4586031000006113	164865005	2693159014
MI - Myocardial infarction	2855341000006114	22298006	1784872019
True posterior wall infarction	4775881000006117	194802003	2535851011
Pericarditis following myocardial infarction	5056951000006117	233885007	350432018
Postoperative myocardial infarction	208365015	129574000	208365015
MI - Silent myocardial infarction	5056461000006113	233843008	350377017
Acute myocardial infarction of septum alone	3784921000006115	79009004	131107010
Acute myocardial infarction of posterolateral wall	967931000006114	15990001	27071012
Previous myocardial infarction	6619201000006117	399211009	545891000000115
Acute myocardial infarction of lateral wall	3452181000006112	58612006	97399018
Subsequent NSTEMI (non-ST segment elevation myocardial infarction)	7574491000006114	703360004	3008532019
Myocardial infarction with complication	6348651000006112	371068009	6348651000006112
Acute lateral myocardial infarction	299711010	58612006	1231860015
Hemopericardium due to and following acute myocardial infarction	12220751000006113	194862000	3673281015
Infarction of heart	2855311000006110	22298006	37441018
Ventricular septal defect as current complication following acute myocardial infarction	67081000006119	233846000	350381017
Acute inferior ST segment elevation myocardial infarction	7572341000006117	703213009	3007793013
Post-myocardial infarction syndrome	109915012	66189004	500341014
Coronary artery embolism	2981141000006112	29899005	50041016
AMI - Acute myocardial infarction	3427201000006111	57054005	1231678011
Cause of Death- Myocardial Infarction	1576271000006117	1576271000006101	1576271000006117
Rupture of chordae tendinae as current complication following acute myocardial infarction	158611000006118	194866002	299822017
Postoperative transmural myocardial infarction of inferior wall	212071000006114	311793000	455419012
Thrombosis of atrium, auricular appendage, and ventricle due to and following acute myocardial infarction	12220791000006119	194868001	3673277015
Acute anterior myocardial infarction	299707016	54329005	1231324017
Acute posterior myocardial infarction	299710011	233838001	350371016
Rupture of cardiac wall without hemopericardium as current complication following acute myocardial infarction	4776251000006118	194865003	299820013
Other acute myocardial infarction	299718016	57054005	94884017
Acute myocardial infarction of anterior wall	3381601000006117	54329005	90302019
Acute ST segment elevation myocardial infarction of inferior wall	7572321000006112	703213009	3007915011
Anterior myocardial infarction NOS	299709018	54329005	1231324017
True posterior myocardial infarction	299712015	194802003	299712015
Subsequent myocardial infarction of anterior wall	299811016	194857001	299811016

Cardiac infarction	2855321000006119	22298006	37442013
Rupture of papillary muscle as current complication following acute myocardial infarction	159001000006119	194867006	299823010
Acute inferolateral myocardial infarction	457531000006110	65547006	1232697013
Acute STEMI (ST elevation myocardial infarction) of inferior wall	7572331000006110	703213009	3007397011
ECG: subendocardial infarct	256458014	164870003	256458014
Electrocardiogram: anteroseptal infarction	4586141000006116	164868007	2619217019
Acute myocardial infarction of inferoposterior wall	3745741000006117	76593002	127193015
Acute STEMI (ST elevation myocardial infarction) of anterior wall	7571601000006115	703164000	3007542019
Electrocardiographic posterior/inferior infarct	4586181000006110	164869004	3300252016
[RFC] Myocardial infarction (MI)	905351000006113	905351000006109	905351000006113
Septal infarction by electrocardiogram	2515471000006117	1077002	2972454013
Cardiac rupture after acute myocardial infarction	537751000006115	233847009	350383019
Diabetes mellitus insulin-glucose infusion in acute myocardial infarction	616081000006113	315287002	459791015
Rupture of chordae tendinae due to and following acute myocardial infarction	12220781000006117	194866002	3673278013
Infarct myocardiectomy	865031000006114	585461000000102	865031000006114
Acute atrial infarction	299719012	194809007	299719012
Subsequent myocardial infarction	299808017	194856005	299808017
Acute anteroseptal myocardial infarction	455651000006114	62695002	104192010
Heart attack	37443015	22298006	37443015
Myocardial infarction with complication	543291000006110	371068009	1209776017
Acute anterior ST segment elevation myocardial infarction	7571581000006113	703164000	3007817016
ECG: anteroseptal infarction	4586131000006114	164868007	256456013
[X]Acute transmural myocardial infarction of unspecif site	362461000006119	57054005	94884017
First myocardial infarction	932081000006118	932081000006102	932081000006118
ECG:posterior/inferior infarct	256457016	164869004	256457016
STEMI - ST elevation myocardial infarction	6651221000006117	401303003	2840840013
Acute nontransmural infarction	3645351000006114	70422006	116993010
Postoperative subendocardial myocardial infarction	455422014	311796008	455422014
Acute anterolateral myocardial infarction	455641000006112	70211005	1233238016
Atrial septal defect as current complication following acute myocardial infarction	498031000006112	194863005	299818010
[X]Subsequent myocardial infarction of unspecified site	300882013	194856005	299808017
Reinfarction of myocardium	4776171000006116	194856005	299809013
Postoperative myocardial infarction, unspecified	455423016	129574000	208365015
Acute myocardial infarction of diaphragmatic wall	3699911000006119	73795002	122559017
Dressler's syndrome	109916013	66189004	109916013
Post-infarction ventricular septal defect	5056501000006113	233846000	350382012
Anteroseptal infarction on electrocardiogram	4586111000006115	164868007	2695135018
Acute subendocardial infarction	116992017	70422006	116992017
ECG: lateral infarction	256459018	164871004	256459018

Acute myocardial infarction of inferior wall	299714019	73795002	122557015
Acute myocardial infarction NOS	299721019	57054005	94884017
Subsequent myocardial infarction of other sites	299813018	194856005	299808017
Attack - heart	219521000000119	22298006	37443015
Cardiac rupture due to and following acute myocardial infarction	12221301000006119	233847009	3673284011
NSTEMI - Non-ST segment elevation MI	6651391000006114	401314000	1787486017
Hemopericardium as current complication following acute myocardial infarction	4776221000006110	194862000	299817017
ECG: antero-septal infarct	4586151000006119	164868007	2820892012
Acute septal infarction	1234306015	79009004	1234306015
Acute non-ST segment elevation myocardial infarction	1780501013	401314000	1780501013
Rupture of cardiac wall without haemopericardium as current complication following acute myocardial infarction	158601000006116	194865003	299821012
Postoperative transmural myocardial infarction of anterior wall	212061000006119	311792005	455418016
Atrial septal defect due to and following acute myocardial infarction	12220771000006115	194863005	3673273016
First myocardial infarction	6546751000006118	394710008	1488431012
Anterolateral infarction by EKG	3202711000006110	43630006	72749019
Acute anteroapical myocardial infarction	3343471000006116	52035003	86618010
Non-Q wave myocardial infarction	6043771000006118	314207007	458526013
MI - Myocardial infarction aborted	2619484018	194821006	2619484018
Haemopericardium as current complication following acute myocardial infarction	813961000006116	194862000	299816014
[X]Subsequent myocardial infarction of other sites	300881018	194856005	299808017
Acute transmural myocardial infarction	2729671000000118	1089471000000109	2729671000000118
Postmyocardial infarction pericarditis	3576391000006116	66189004	109917016
Cause of Death- Acute Myocardial Infarction	1576301000006115	1576301000006104	1576301000006115
Haemopericardium due to and following acute myocardial infarction	12220761000006110	194862000	3673282010
Acute inferoposterior infarction	1234005010	76593002	1234005010
Coronary thrombosis	1786197015	398274000	1786197015
ECG: myocardial infarction	256452010	164865005	256452010
Thrombosis of atrium, auricular appendage, and ventricle as current complications following acute myocardial infarction	100681000006116	194868001	299824016
Myocardial Infarction	884151000006119	57054005	884151000006119
Myocardial infarction aborted	682481000006118	194821006	2619484018
MI - acute myocardial infarction	219531000000117	57054005	94884017
Acute myocardial infarction	94884017	57054005	94884017
Myocardial infarction	2855301000006112	22298006	37436014
Microinfarct of heart	3185771000006112	42531007	70952012
[X]Other current complicatns following acute myocard infarct	408571000006116	302049001	443587013

Table S33. Myocardial infarction HES (ICD10) codes

ICD10 codes	Term
I21.0	Acute transmural myocardial infarction of anterior wall
I21.1	Acute transmural myocardial infarction of inferior wall
I21.2	Acute transmural myocardial infarction of other sites
I21.3	Acute transmural myocardial infarction of unspecified site
I21.4	Acute subendocardial myocardial infarction
I21.9	Acute myocardial infarction, unspecified
I22.0	Subsequent myocardial infarction of anterior wall
I22.1	Subsequent myocardial infarction of inferior wall
I22.8	Subsequent myocardial infarction of other sites
I22.9	Subsequent myocardial infarction of unspecified site
I23.0	Haemopericardium as current complication following acute myocardial infarction
I23.1	Atrial septal defect as current complication following acute myocardial infarction
I23.2	Ventricular septal defect as current complication following acute myocardial infarction
I23.3	Rupture of cardiac wall without haemopericardium as current complication following acute myocardial infarction
I23.4	Rupture of chordae tendineae as current complication following acute myocardial infarction
I23.5	Rupture of papillary muscle as current complication following acute myocardial infarction
I23.6	Thrombosis of atrium, auricular appendage, and ventricle as current complications following acute myocardial infarction
I23.8	Other current complications following acute myocardial infarction
I24.1	Dressler syndrome

Table S34. Vascular dementia Aurum codes

Term	Medcode ID	SNOMED CT Concept ID	SNOMED CT Description ID
[X]Vascular dementia, unspecified	295681016	429998004	2770951017
Vascular dementia of acute onset, other symptoms, predominantly hallucinatory	1972521000006114	1972521000006105	1972521000006114
Subcortical vascular dementia, other symptoms, predominantly depressive	1972771000006116	1972771000006100	1972771000006116
Mixed cortical and subcortical vascular dementia, other symptoms, predominantly hallucinatory	1972871000006113	1972871000006109	1972871000006113
Mixed cortical and subcortical vascular dementia, other symptoms, predominantly depressive	1972911000006111	1972911000006107	1972911000006111
VAD - Vascular dementia	3414251000006112	56267009	497560014
Subcortical leukoencephalopathy	3964601000006113	90099008	149344015
Binswanger's dementia	3964611000006111	90099008	149345019
Binswanger's encephalopathy	1235534016	90099008	1235534016
Binswanger's disease	149347010	90099008	149347010
MID - Multi-infarct dementia	3414231000006117	56267009	497558012
Arteriosclerotic dementia NOS	294656010	56267009	497559016
Subcortical leucoencephalopathy	3964591000006117	90099008	508841015
Uncomplicated arteriosclerotic dementia	294652012	191463004	294652012
Arteriosclerotic dementia with delirium	294653019	191464005	294653019
Arteriosclerotic dementia with paranoia	294654013	191465006	294654013
Arteriosclerotic dementia with depression	294655014	191466007	294655014
Ischaemic vascular dementia	7840631000006118	723123001	3334662012
Vascular dementia of acute onset, other symptoms, predominantly delusional	1972501000006116	1972501000006100	1972501000006116
Other vascular dementia, other symptoms, predominantly depressive	1973381000006112	1973381000006108	1973381000006112
Vascular dementia, with delirium	2664611000006117	10349009	18021011
[X]Vascular dementia	431681000006117	429998004	2770951017
[X]Vascular dementia of acute onset	431691000006119	230285003	345110014
Multi infarct dementia	3414261000006114	56267009	2921000019
[D] Vascular dementia	914921000006117	914921000006101	914921000006117
Vascular dementia of acute onset, without additional symptoms	1972481000006114	1972481000006105	1972481000006114
Multi-infarct dementia, other symptoms, predominantly delusional	1972621000006113	1972621000006109	1972621000006113
Multi-infarct dementia, other symptoms, predominantly depressive	1972661000006119	1972661000006103	1972661000006119
Subcortical vascular dementia, without additional symptoms	1972711000006113	1972711000006109	1972711000006113
Subcortical vascular dementia, other symptoms, predominantly delusional	1972731000006119	1972731000006103	1972731000006119
Other vascular dementia, without additional symptoms	1973221000006118	1973221000006102	1973221000006118
Other vascular dementia, other symptoms, predominantly delusional	1973271000006117	1973271000006101	1973271000006117
Other vascular dementia, other mixed symptoms	1973401000006112	1973401000006108	1973401000006112
Multi infarct dementia	696161000006115	56267009	93568017
Vascular dementia of acute onset, other symptoms, predominantly depressive	1972541000006119	1972541000006103	1972541000006119

Multi-infarct dementia, without additional symptoms	1972601000006115	1972601000006104	1972601000006115
Subcortical vascular dementia, other mixed symptoms	1972791000006115	1972791000006104	1972791000006115
Mixed cortical and subcortical vascular dementia, without additional symptoms	1972821000006112	1972821000006108	1972821000006112
Other vascular dementia, other symptoms, predominantly hallucinatory	1973341000006118	1973341000006102	1973341000006118
Vascular dementia, unspecified, without additional symptoms	1973461000006113	1973461000006109	1973461000006113
Vascular dementia, unspecified, other symptoms, predominantly hallucinatory	1973531000006117	1973531000006101	1973531000006117
[X]Other vascular dementia	295680015	429998004	2770951017
[X]Arteriosclerotic dementia	363791000006112	56267009	497559016
Multi-infarct dementia, other symptoms, predominantly hallucinatory	1972641000006118	1972641000006102	1972641000006118
Multi-infarct dementia, other mixed symptoms	1972681000006112	1972681000006108	1972681000006112
Vascular dementia, unspecified, other symptoms, predominantly depressive	1973551000006112	1973551000006108	1973551000006112
Vascular dementia, unspecified, other mixed symptoms	1976831000006111	1976831000006107	1976831000006111
Arteriosclerotic dementia	497559016	56267009	497559016
Vascular dementia of acute onset, other mixed symptoms	1972571000006110	1972571000006106	1972571000006110
Subcortical vascular dementia, other symptoms, predominantly hallucinatory	1972751000006114	1972751000006105	1972751000006114
Mixed cortical and subcortical vascular dementia, other symptoms, predominantly delusional	1972831000006110	1972831000006106	1972831000006110
Mixed cortical and subcortical vascular dementia, other mixed symptoms	1972931000006117	1972931000006101	1972931000006117
Vascular dementia, unspecified, other symptoms, predominantly delusional	1973501000006113	1973501000006109	1973501000006113
[X]Mixed cortical and subcortical vascular dementia	398571000006112	230287006	345112018
[X]Subcortical vascular dementia	428201000006119	230286002	345111013
[X]Multi-infarct dementia	399031000006111	56267009	93568017
Subcortical arteriosclerotic encephalopathy	3964651000006112	90099008	1235535015
Vascular dementia with behavioral disturbance	8089241000006117	288631000119104	3290002019

Table S35. Vascular dementia HES (ICD10) codes

ICD10 codes	Term
F01.0	Vascular dementia of acute onset
F01.1	Multi-infarct dementia
F01.2	Subcortical vascular dementia
F01.3	Mixed cortical and subcortical vascular dementia
F01.8	Other vascular dementia
F01.9	Vascular dementia, unspecified

Table S36. Gastrointestinal bleeding Aurum codes

Term	Medcode ID	SNOMED CT Concept ID	SNOMED CT Description ID
Gastric ulcer with hemorrhage AND perforation	3513611000006117	62366003	103655018
Haemorrhage of large intestine co-occurrent and due to diverticular disease of large intestine	11823161000006110	197092000	3512394014
Bleeding duodenal ulcer	2937141000006110	27281001	1225269012
DU - acute + h'ge + perf.	885871000006115	86895006	885871000006115
GU - acute + haemorrhage	885771000006112	89748001	885771000006112
Bleeding diverticulosis of large intestine	11823171000006115	197092000	3512393015
Bleeding chronic duodenal ulcer	1235462014	89469000	1235462014
DU - acute + haemorrhage	885851000006113	12847006	885851000006113
Haemorrhagic duodenitis	4058111000006114	95531001	512171013
Bleeding external hemorrhoids	2923111000006117	26421009	44253010
Bleeding internal hemorrhoids	3733941000006117	75884004	126038015
Vomiting blood - fresh	397912015	267051003	397912015
Chronic peptic ulcer with hemorrhage	3296621000006114	49232000	82009014
RB - Rectal bleeding	2692031000006113	12063002	464498014
GI bleeding	3710501000006113	74474003	123689014
Rectal haemorrhage	464500010	12063002	464500010
Gastric haemorrhage	303660017	61401005	499003013
Acute peptic ulcer with haemorrhage and perforation	361430016	111353003	361430016
Lower GI bleeding	3926401000006112	87763006	145512011
Vomiting blood - coffee ground	492804019	40835002	492804019
Haemorrhage of large intestine with diverticular disease of large intestine	11823191000006119	197092000	3512395010
PU - chronic + h'ge + perf.	886001000006111	61300005	886001000006111
Chronic peptic ulcer with hemorrhage AND perforation	3496021000006116	61300005	101859019
GI - Gastrointestinal hemorrhage	3710571000006119	74474003	1218401013
Chronic gastrojejunal ulcer with hemorrhage AND perforation	3236981000006112	45640006	76108010
Upper GI - gastrointestinal haemorrhage	3102201000006117	37372002	1216567017
Hemorrhage of large intestine co-occurrent and due to diverticular disease of large intestine	11823151000006113	197092000	3512397019
DU - chronic + h'ge + perf.	885911000006117	36975000	885911000006117
Bleeding gastric ulcer	2752321000006114	15902003	1221244018
Rectorrhagia	2691961000006110	12063002	20789018
Chronic gastric ulcer with hemorrhage AND with perforation	3739191000006114	76181002	126521010
Chronic gastric ulcer with hemorrhage	3430281000006116	57246001	95214011
Haematemesis	507679012	8765009	507679012
Duodenal ulcer with haemorrhage	302534015	27281001	483490017
Oesophageal varices with bleeding in diseases EC	300776018	195475003	300776018
Acute upper GI bleeding	3126711000006119	38938002	65309016
Unspecified peptic ulcer with haemorrhage and perforation	302571018	13200003	22592012

Unspecified peptic ulcer with haemorrhage	302568014	13200003	22592012
Hematoma of perianal region	7375281000006110	449815008	2913034013
Rectal bleeding	20792019	12063002	20792019
Chronic peptic ulcer with haemorrhage	495344012	49232000	495344012
Melaena - O/E of faeces	411734011	275782008	411734011
Upper GI hemorrhage	3102171000006116	37372002	62347019
Bleeding acute gastric ulcer	1235493010	89748001	1235493010
Haemorrhage of rectum and anus	396382012	266464001	396382012
Melena	2545151000006112	2901004	5911019
GU - chronic + h'ge + perf.	885831000006118	76181002	885831000006118
GU - chronic + haemorrhage	885811000006112	57246001	885811000006112
History of lower GIT haemorrhage	1819591000006117	1819591000006101	1819591000006117
Gastrooesophageal laceration-hemorrhage syndrome	3068381000006113	35265002	2646918012
PR - Bleeding per rectum	2692001000006117	12063002	464495012
Upper GI bleeding	3102181000006118	37372002	62348012
Acute gastric ulcer with hemorrhage	3958881000006116	89748001	148789010
Duodenal ulcer with hemorrhage	11902881000006118	27281001	45608011
Painless rectal bleeding	2534250012	414992000	2534250012
Acute peptic ulcer with haemorrhage	470365013	12274003	470365013
Rectal hemorrhage	2692051000006118	12063002	464501014
Hematemesis	2639821000006115	8765009	15458019
Acute peptic ulcer with hemorrhage and perforation	4194481000006117	111353003	90139013
Rectal packing for hemorrhage	4716091000006113	182606003	282271018
Chronic gastric ulcer with haemorrhage AND with perforation	554311000006114	76181002	503219010
GI haemorrhage	3710531000006117	74474003	502714014
Haemorrhage of rectum and anus NOS	303317017	266464001	396382012
GI hemorrhage	3710511000006111	74474003	123690017
Evacuation of perianal hematoma	5789981000006110	293018003	433185011
Bleeding hemorrhoid	3335301000006118	51551000	1218192016
Melaena on examination of faeces	5576661000006114	275782008	2771057011
Chronic gastrojejunal ulcer with haemorrhage AND perforation	494304013	45640006	494304013
Chronic gastric ulcer with haemorrhage	497833010	57246001	497833010
Chronic peptic ulcer with haemorrhage AND perforation	498977018	61300005	498977018
Chronic gastrojejunal ulcer with haemorrhage	499386010	62838000	499386010
Oesophageal varices with haemorrhage	2781041000006115	17709002	478406011
Gastric hemorrhage	3497601000006119	61401005	102007012
Oesophageal haemorrhage	2741641000006118	15238002	477249012
Acute duodenal ulcer with hemorrhage AND perforation	3912771000006119	86895006	144111018
Acute duodenal ulcer with hemorrhage	2704011000006118	12847006	22034017
Haematoma of perianal region	7375271000006112	449815008	2912591019

Duodenal ulcer with haemorrhage	2937121000006115	27281001	2937121000006115
Perianal haematoma	233701000006116	449815008	2213971000000113
Hemorrhage of rectum and anus	5493021000006119	266464001	396383019
Unspec gastrojejunal ulcer with haemorrhage and perforation	81321000006111	16121001	27283019
Oesophageal bleeding	2741601000006115	15238002	477250012
Bleeding from anus	481141000006114	6072007	498827015
Internal bleeding haemorrhoids	1216930015	75884004	1216930015
Unspecified gastrojejunal ulcer with haemorrhage	302606019	16121001	27283019
Unspecified gastric ulcer with haemorrhage and perforation	302490012	62366003	499256017
Acute gastric ulcer with bleeding	3958891000006118	89748001	148790018
Melena on examination of feces	5576651000006112	275782008	2770255015
Melaena	5913016	2901004	5913016
Acute gastrojejunal ulcer with haemorrhage	499719010	63954007	499719010
Melena - O/E of feces	5576641000006110	275782008	411735012
Chronic gastrojejunal ulcer with hemorrhage	3521501000006114	62838000	104433010
Gastric bleeding	3497621000006112	61401005	102011018
Anal/rectal haemorrhage	886471000006110	266464001	886471000006110
PU - acute + haemorrhage	885941000006118	12274003	885941000006118
Gastric ulcer with hemorrhage	2752311000006118	15902003	26937011
Internal bleeding hemorrhoids	3733961000006118	75884004	1218417019
Upper GI - gastrointestinal hemorrhage	3102211000006119	37372002	1218058016
Bleeding pile	3335311000006115	51551000	1230996010
Unspecified duodenal ulcer with haemorrhage and perforation	302537010	51868009	86362013
GI - Gastrointestinal haemorrhage	3710541000006110	74474003	1216914011
Bleeding hemorrhoids	3335281000006117	51551000	85856015
Bleeding per rectum	515491000006115	12063002	464494011
Anal margin haematoma	5585681000006116	276465001	412609016
Peptic ulcer with haemorrhage	3542811000006116	64121000	499762011
Esophageal varices with hemorrhage	2781031000006113	17709002	29938017
Chronic duodenal ulcer with hemorrhage AND perforation	3095701000006110	36975000	61676011
Acute duodenal ulcer with haemorrhage	474430017	12847006	474430017
Acute haemorrhagic gastritis	478553011	2367005	481682019
Chronic duodenal ulcer with haemorrhage	508524014	89469000	508524014
Esophageal bleeding	2741651000006116	15238002	477251011
PU - acute + h'ge + perf.	885961000006119	111353003	885961000006119
Upper GI haemorrhage	3102191000006115	37372002	486703016
H/O: upper GIT hemorrhage	6017191000006116	312489007	456219016
PU - acute + perforation	885951000006116	79118000	885951000006116
Proctorrhagia	2691971000006115	12063002	20790010
Gastroesophageal laceration-hemorrhage syndrome	3068351000006117	35265002	58844012

Unspec peptic ulcer; unspec haemorrhage and/or perforation	81501000006111	13200003	22592012
Unspec gastrojejunal ulcer; unspec haemorrhage/perforation	81331000006114	16121001	27283019
Lower GI hemorrhage	3926391000006110	87763006	145511016
Lower GI haemorrhage	3926411000006110	87763006	507737011
Perianal haematoma	483207010	237331002	355700019
Bleeding internal haemorrhoids	3733931000006110	75884004	200553019
Acute duodenal ulcer with haemorrhage AND perforation	507303017	86895006	507303017
Gastric hemorrhage due to erosive gastritis	7987031000006110	7071000119102	3015365015
Chronic duodenal ulcer with hemorrhage	3954241000006119	89469000	148344012
Haemorrhage of oesophagus	477248016	15238002	477248016
Gastrotomy and ligation of bleeding point of stomach	269184010	173778009	269184010
Chronic duodenal ulcer with haemorrhage AND perforation	486596015	36975000	486596015
Acute peptic ulcer with hemorrhage	2695521000006113	12274003	21136016
GU - acute + perforation	885781000006110	19850005	885781000006110
Rectal packing for haemorrhage	4716081000006110	182606003	282269018
BOV - Bleeding oesophageal varices	2781061000006116	17709002	1216061014
Perianal haematoma	1757561000000116	449815008	2213971000000113
Bleeding external haemorrhoids	2923101000006115	26421009	196528011
Esophageal hemorrhage	2741621000006113	15238002	25860014
Bleeding esophageal varices	2781021000006110	17709002	29937010
Unspec duodenal ulcer; unspec haemorrhage and/or perforation	81301000006118	51868009	86362013
Acute gastrojejunal ulcer with haemorrhage AND perforation	504674011	81387001	504674011
H/O: upper GIT haemorrhage	456217019	312489007	456217019
Acute gastric ulcer with hemorrhage AND perforation	3292411000006119	48974009	81590012
PRB - Rectal bleeding	464499018	12063002	464499018
Gastric ulcer with haemorrhage AND perforation	81311000006115	62366003	499256017
Bleeding oesophageal varices	2781011000006119	17709002	478405010
Acute hemorrhagic gastritis	2536601000006112	2367005	5056011
Esophageal varices with bleeding, associated with another disorder	4779741000006118	195475003	2575714018
Gastric ulcer with haemorrhage	302487018	15902003	477573015
Bloody diarrhoea	1566381000006119	95545007	201773015
Perinatal gastrointestinal haemorrhage	495197011	48729005	495197011
Acute gastrojejunal ulcer with hemorrhage	3539941000006116	63954007	106302011
Bleeding diverticulosis	303134017	197092000	303134017
Hemorrhage of large intestine with diverticular disease of large intestine	11823181000006117	197092000	3512398012
Acute gastric ulcer with haemorrhage	508664015	89748001	508664015
Bleeding ulcer	3393951000006114	55075001	91564012
Bleeding chronic gastric ulcer	1231704018	57246001	1231704018
Bleeding oesophageal ulcer	3438321000006112	57748001	3027281014

Vomiting of blood	2639831000006117	8765009	15459010
External bleeding hemorrhoids	2923131000006111	26421009	1217729010
Bleeding haemorrhoid	3335291000006119	51551000	1216702010
GU - acute + h'ge + perf.	885791000006113	48974009	885791000006113
DU - acute + perforation	885861000006110	61347001	885861000006110
PU - chronic + haemorrhage	885981000006112	49232000	885981000006112
DU - chronic + haemorrhage	885891000006119	89469000	885891000006119
PR - Blood per rectum	2692021000006110	12063002	464497016
Vomiting blood	15459010	8765009	507678016
Blood per rectum	2692011000006119	12063002	464496013
Painful rectal bleeding	2534219010	414991007	2534219010
BOV - Bleeding esophageal varices	2781081000006114	17709002	1217568017
Manchester triage - GI bleeding	1984161000006112	1984161000006108	1984161000006112
Oesophageal varices with bleeding, associated with another disorder	4779721000006113	195475003	2575715017
Esophageal varices with bleeding	2781071000006111	17709002	1217567010
Acute gastrojejunal ulcer with hemorrhage AND perforation	3823411000006113	81387001	135007015
Perianal hematoma	1988561000006114	449815008	2213971000000113
Oesophageal varices with bleeding	1216060010	17709002	1216060010
Acute gastric ulcer with haemorrhage AND perforation	495273013	48974009	495273013
Intestinal haemorrhage NOS	303663015	74474003	123688018
Hemorrhage of esophagus	2741611000006117	15238002	25859016
Gastro-oesophageal laceration-haemorrhage syndrome	3068361000006115	35265002	1216440014
Bleeding haemorrhoids	300752014	51551000	198339016
Acute lower GI bleeding	3890061000006117	85521005	141749014

Table S37. Gastrointestinal bleeding HES (ICD10) codes

ICD10 codes	Term
I85.0	Oesophageal varices with bleeding
I98.3	Oesophageal varices with bleeding in diseases classified elsewhere
K22.6	Gastro-oesophageal laceration-haemorrhage syndrome
K25.0	Gastric ulcer. Acute with haemorrhage
K25.2	Gastric ulcer. Acute with both haemorrhage and perforation
K25.4	Gastric ulcer. Chronic or unspecified with haemorrhage
K25.6	Gastric ulcer. Chronic or unspecified with both haemorrhage and perforation
K26.0	Duodenal ulcer. Acute with haemorrhage
K26.2	Duodenal ulcer. Acute with both haemorrhage and perforation
K26.4	Duodenal ulcer. Chronic or unspecified with haemorrhage
K26.6	Duodenal ulcer. Chronic or unspecified with both haemorrhage and perforation
K27.0	Peptic ulcer, site unspecified. Acute with haemorrhage
K27.2	Peptic ulcer, site unspecified. Acute with both haemorrhage and perforation
K27.4	Peptic ulcer, site unspecified. Chronic or unspecified with haemorrhage
K27.6	Peptic ulcer, site unspecified. Chronic or unspecified with both haemorrhage and perforation
K28.0	Gastrojejunal ulcer. Acute with haemorrhage
K28.2	Gastrojejunal ulcer. Acute with both haemorrhage and perforation
K28.4	Gastrojejunal ulcer. Chronic or unspecified with haemorrhage
K28.6	Gastrojejunal ulcer. Chronic or unspecified with both haemorrhage and perforation
K29.0	Acute haemorrhagic gastritis
K62.5	Haemorrhage of anus and rectum
K66.1	Haemoperitoneum
K92.0	Haematemesis
K92.1	Melaena
K92.2	Gastrointestinal haemorrhage, unspecified

Table S38. Bleeding at other anatomy sites Aurum codes

Term	Medcode ID	SNOMED CT Concept ID	SNOMED CT Description ID
Bleeding from nose	5270631000006113	249366005	5270631000006113
O/E - retinal microaneurysms	255335015	163986001	255335015
Nontraumatic scrotal hematoma	4790941000006116	198058000	3036672010
Bleeding into joint	3830271000006111	81808003	1234640010
Drainage of haematoma of pinna and insertion of bolster sutures	4650061000006119	172619007	267229016
Control of hemorrhage	3330351000006114	51241000	85365019
Haemarthrosis of PIP joint of finger	310710010	202409003	310710010
Haemarthrosis of wrist	310705011	202407001	310705011
Haemarthrosis of the ankle	310724014	202415003	310724014
Primary post tonsillectomy hemorrhage	6046521000006116	314483007	458849015
Injury of heart with haemopericardium	320982018	210078001	320982018
Subungual haemorrhage	5125561000006112	238722001	357791012
Postcoital bleeding	81454015	48880000	81454015
Pelvic haematoma	5477481000006114	264558008	393037010
Corneal haemorrhage	5029211000006119	231916005	347513015
Bleeding stoma	353962019	236129005	353962019
Acute posthaemorrhagic anaemia	399228011	267530009	399228011
Vitreous hemorrhage	3005171000006115	31341008	52406015
Hematoma of pinna	3930851000006114	88050005	145963019
Haematoma of lower leg	7373861000006119	449704006	2912608014
Bleeding in mouth	2858361000006116	22490002	481357012
Haematoma of tongue	5455391000006110	262651006	390627015
Extradural haematoma	7085881000006119	428268007	2899390010
Retinal blot haemorrhage	5239831000006118	247133006	2619788016
Haematoma of penis	5681941000006113	283948004	422520011
Perioperative haemorrhage	3636041019	762957002	3636041019
Persistent haematuria	5656871000006111	281859000	419988013
Corpus cavernosum haematoma	304493010	198031007	304493010
Thyroid hemorrhage and infarction	4757961000006112	190305000	292442019
Subretinal haemorrhage	475607010	13937002	475607010
Corpus cavernosum haemorrhage	1216873013	69452005	1216873013
Scrotal haemorrhage	1216798017	62182006	1216798017
Bleeding tooth socket	5700021000006118	285335006	424307013
Retinal hemorrhages	2966421000006119	28998008	1217788016
Massive haemoptysis	2229721000000116	862231000000103	2229721000000116
Head injury with haemorrhage from nose	4395871000006112	127276009	474087019
Spontaneous intraperitoneal hemorrhage	3236641000006110	45626005	76080019
Deformity/haematoma of pinna	883651000006111	286943000	883651000006111
Nontraumatic haemoperitoneum	3236611000006111	45626005	2816649012

Reopening of chest and re-exploration of intra-abdominal operation site and surgical arrest of postoperative bleeding	4679481000006110	177768005	275176015
Nipple bleeding	5755901000006111	290103001	430097013
Contact bleeding	5103751000006111	237133008	355447012
Haematoma of thigh	5967731000006111	307391000	450646011
Moderate vaginal bleeding	5748541000006110	289539007	429439016
Extradural hemorrhage	3850511000006116	82999001	137670017
On examination - epistaxis	4576361000006110	164187003	4576361000006110
Intraperitoneal haematoma	5088411000006117	236000006	353764011
External capsule haemorrhage	300276019	195167002	300276019
Haemophthalmos, except current injury	4771081000006111	193287005	2550868017
Haemorrhage from pharynx	6119831000006110	324618004	465452016
Liver hematoma	5457191000006118	262796008	390818018
Subungual haematoma, hand	5561121000006110	274202000	409975019
Expulsive haemorrhage	2684991000006115	11623000	2474957017
Hemopneumothorax	2763811000006111	16632002	28149019
History of abnormal uterine bleeding	5497401000006114	267016006	2986860010
History of epistaxis	1137551000000110	511511000000101	1137551000000110
Hematoma of spleen	5457481000006111	262818004	390852015
Non-traumatic testicular haematoma	3074001000006117	35644004	1216444017
Deep intra-retinal hemorrhages	3288931000006115	48742005	1490493013
Wrist haemarthrosis	310706012	202407001	310706012
Elbow haemarthrosis	310700018	202405009	310700018
Spontaneous intraperitoneal haemorrhage	3236701000006118	45626005	494285013
Traumatic haematuria	512189014	95567008	512189014
Optic nerve sheath hemorrhage	2729121000006117	14460007	1217537016
Adrenocortical haemorrhage	356328018	237766002	356328018
Haemarthrosis of DIP joint of finger	310711014	202410008	310711014
Liposuction removal of haematoma	275087014	177714000	275087014
Haemorrhage	3325451000006112	50960005	495845019
Bleeding skin	5846621000006115	297968009	438563011
Bleeding gums	143080016	86276007	143080016
Infected haematoma	7510021000006118	698573001	2974732011
Hemarthrosis of wrist	4813321000006115	202407001	310703016
Haemorrhage of cervix	504705015	81488005	504705015
External bleeding haemorrhoids	1216226011	26421009	1216226011
H/O: haematuria	251768018	161550001	251768018
Haematoma of kidney	5458501000006117	262891006	390956018
O/E - vitreous hemorrhages	4573231000006119	163990004	255340011
Evacuation of haematoma from vagina	649591000006116	392256003	1490236018
Bleeding	4454741000006110	131148009	210860014
Haemarthrosis of multiple sites	812741000006113	76427005	503277015

Haematoma - postoperative	813261000006113	213262007	325137018
On examination - subconjunctival hemorrhage	4552921000006116	162815002	2667822015
Haematoma of temporal region	5965691000006113	307214007	450417012
Bleeding varices	3427151000006119	57052009	94882018
Vulval haematoma evacuated	869431000006119	65687007	869431000006119
PU - chronic + haemorrhage	885981000006112	49232000	885981000006112
Bleeding of ear canal	5875341000006114	300129004	441138011
Haemarthrosis - hand joint	890591000006112	27507004	890591000006112
Optic disc haemorrhage	5241101000006119	247233000	369184014
Vulval hematoma	3628381000006110	69385001	1218357015
Blood in anterior chamber	3723211000006110	75229002	1233846014
Normocytic anemia following acute bleed	5914361000006111	303060002	445120015
Breast hematoma	5913301000006112	302924003	444948013
Corpus cavernosum hematoma	4790761000006115	198031007	304492017
Fresh bleeding from vagina	5748511000006111	289536000	429436011
Hemorrhage of cervix	3825061000006115	81488005	135183016
Scrotal hemorrhage	3510231000006116	62182006	1218286015
Bleeding haemorrhoid	3335291000006119	51551000	1216702010
Hemarthrosis of glenohumeral joint	7529911000006111	700018009	2987396013
Perineal hematoma	5105871000006114	237331002	355701015
Haemophthalmos without current injury	4771041000006117	193287005	2547718010
Hemarthrosis of acromioclavicular joint	4813241000006115	202404008	310696014
Haemorrhage of testis	3764241000006119	77708008	503651019
O/E - subconjunctival hemorrhage	4552911000006112	162815002	253782010
Hemorrhage of scrotum	3510211000006110	62182006	103341016
Evacuation of intracranial subdural hematoma	3469871000006118	59712006	498517019
Hemarthrosis of MCP joint	4813361000006114	202408006	310708013
Hemorrhage in optic nerve sheaths	2729081000006119	14460007	24578017
Acute hemorrhagic pancreatitis	2568871000006112	4399003	7699015
Retrohyaloid haemorrhage	2683851000006119	11547003	411994019
Nontraumatic scrotal haematoma	4790951000006119	198058000	3036831010
Evacuation of intracerebral hematoma	2666411000006111	10458001	18195013
Haemorrhage in optic nerve sheaths	2729071000006117	14460007	476340016
Paroxysmal hematoma of the finger	5126821000006110	238824006	357937018
Nontraumatic hematoma of testis	3073991000006114	35644004	59462016
Hematoma	3073011000006117	35566002	59338013
Haemarthrosis of sternoclavicular joint	310694012	202403002	310694012
Haemarthrosis of hip	310715017	202411007	310715017
Haemarthrosis of metacarpophalangeal joint	4813371000006119	202408006	2692465010
Surgical arrest of bleeding from internal nose	267550012	172811007	267550012
Perineal haematoma	355700019	237331002	355700019
Haemarthrosis of acromioclavicular joint	310695013	202404008	310695013

Peri-operative haemorrhage or haematoma	403111013	269302003	403111013
Thyroid haemorrhage and infarction	292443012	190305000	292443012
Haemorrhagic disorder due to circulating anticoagulants	294307015	191287000	294307015
Exophthalmos due to orbital haemorrhage	298692016	194016009	298692016
Tonsillar haemorrhage	5036181000006114	232425007	348218013
Haematemesis - cause unknown	5983251000006115	308904008	452291015
Vaginal bleeding problem	5748581000006116	289543006	429444011
Haematoma of groin	5967711000006117	307390004	450644014
Anticoagulant-induced bleeding	5612371000006113	278365007	415242016
Traumatic haematoma	409951019	274179004	409951019
Adrenal haemorrhage	495316011	49111001	495316011
Thyroid haemorrhage	1216311012	3002002	1216311012
Haemorrhage of eyelid	105913010	63720000	105913010
Intracranial hematoma following injury	3111241000006113	37955001	63313013
Haematoma of lower limb	7373841000006118	449703000	2912531016
Intraocular hemorrhage	4021591000006119	93478000	154753016
Hemorrhagic detachment of retinal pigment epithelium	3312501000006111	50165004	83560014
Haematoma of scrotum	3962411000006116	89966002	508761019
Dissection of carotid artery	7806271000006110	720626009	3441846017
Orbital haemorrhage	3000741000006117	31056006	484766013
Postoperative wound hemorrhage	7510191000006115	698584008	2974777013
Haematoma with intact skin	5518251000006117	270911002	405394012
Flame-shaped haemorrhage	12222161000006112	247132001	3644832012
Respiratory tract haemorrhage	317993018	95431003	512121010
Upper urinary tract haematuria	5097861000006116	236717007	354811014
Haemarthrosis of distal interphalangeal joint of finger	4813431000006112	202410008	2695357016
Nasal septal haematoma	5035491000006111	232377002	348145019
Respiratory tract hemorrhage	4056521000006113	95431003	158078018
Nontraumatic extradural intracranial haemorrhage	6593151000006119	397809001	2916444013
Vaginal hematoma	3362091000006111	53162000	88426019
Wrist hemarthrosis	4813331000006117	202407001	310704010
Hemorrhage of thyroid	2547001000006117	3002002	6091011
Postoperative hemorrhage	4177071000006111	110265006	175034010
Hyphema	3723181000006111	75229002	124940012
Suture of nose for epistaxis	5724391000006117	287400009	2988047014
O/E - epistaxis	11905171000006116	164187003	255582011
Nontraumatic epidural haemorrhage	6593121000006111	397809001	1783616016
On examination epistaxis	11873291000006112	164187003	3634839017
Retinal blot hemorrhages	3288921000006118	48742005	1490492015
Evacuation of haematoma of vulva	3568141000006115	65687007	500187011
Retrobulbar haematoma	4774401000006112	194179009	298915016

Hemarthrosis of hip	4813471000006110	202411007	310713012
Extradural haemorrhage after injury	5459181000006112	262949005	391029012
Vulval haematoma	3628371000006112	69385001	1216870011
Haematuria associated with urinary catheter	1938771000006116	1938771000006100	1938771000006116
Macroscopic haematuria	4790361000006116	197941005	304354019
O/E - throat haemorrhage	255708010	164279008	255708010
Epistaxis control -cryosurgery	860731000006118	287393001	860731000006118
O/E - retinal hemorrhages	4573161000006116	163987005	255336019
Bleeding	3325491000006118	50960005	84923010
Bleeding cervix	5751971000006119	289799006	429729011
Subungual haematoma	403014019	269219004	403014019
Haemorrhage of parathyroid	292686019	190460008	292686019
Haematoma	3073001000006115	35566002	486214017
Intraabdominal hemorrhage	7283681000006111	443826006	2841291010
Haematoma of abdominal wall	7334721000006118	447096000	2883375018
Wound haemorrhage	5131561000006112	239161005	358453011
Surgical arrest of post-extraction haemorrhage	1006061000006112	1006061000006108	1006061000006112
Retinal pigment epithelium haemorrhagic detachment	1216693019	50165004	1216693019
Intraoperative haemorrhage	744731000006115	213261000	325136010
Retinal haemorrhages	814181000006113	28998008	1216289019
Haematoma of broad ligament	813321000006114	83294006	505536016
Hemorrhage postprocedure	4177101000006118	110265006	348212014
Bleeding from vagina	1786762014	289530006	2670003011
Chronic hemorrhagic anemia	6842811000006111	413533008	2536841015
Haemarthrosis - ankle/foot	890621000006114	267948008	890621000006114
Haemarthrosis - hip joint	890601000006116	202411007	890601000006116
Vaginal vault haematoma	5103381000006119	237102006	355396015
Hemarthrosis of subtalar joint	4813591000006110	202416002	310726011
Surgical arrest of post-extraction hemorrhage	4654761000006111	173330006	268350014
Posttonsillectomy bleeding	8015351000006113	35101000119106	3306550017
Blood in urine - haematuria	518011000006114	53298000	1216720018
Pulmonary haemorrhage with glomerulonephritis	3319101000006111	50581000	495714011
Male haematocele	3577551000006116	66259004	500367016
Hemorrhage from ureter	5096521000006110	236606001	354659012
Non-traumatic testicular hematoma	3074011000006119	35644004	1217934015
Evacuation of vulval haematoma	3568161000006116	65687007	1216830016
Subconjunctival haemorrhage of both eyes	8110661000006118	343341000119108	3427632015
Deep retinal hemorrhage	3288871000006111	48742005	81229018
Pulmonary hemorrhage	11989391000006117	78144005	301491000006118
Dot AND blot hemorrhage	3288881000006114	48742005	81231010
Haemorrhage postprocedure	4177091000006112	110265006	348211019
Nontraumatic haematoma of breast	4791291000006110	198123000	2619486016

Liver haematoma	5457181000006116	262796008	5457181000006116
Pharyngeal hemorrhage	11905801000006118	324618004	465449012
Hematoma of vulva	3628361000006117	69385001	115258016
Haemorrhage of prostate	3223401000006119	44843000	494041015
Haemopericardium due to and following acute myocardial infarction	12220761000006110	194862000	3673282010
Hemorrhage at vitreoretinal interface	5239141000006115	247097006	369011013
Bleeding reduction: nasal	3076691000006114	35807001	1490745018
Expulsive choroidal hemorrhage	2684981000006118	11623000	20081013
Extradural haemorrhage	884431000006116	397809001	884431000006116
Manchester triage - PV bleeding	1984281000006110	1984281000006106	1984281000006110
Pleural haemorrhage	3013671000006111	31892009	485062013
Haemorrhage control	3330361000006111	51241000	1490265012
Hemarthrosis of first metatarsophalangeal joint	4813651000006115	202419009	2692707015
Hemorrhage from the ear	2921441000006110	26322001	44087014
Evacuation of hematoma of vagina	6531331000006119	392256003	1477262019
Abnormal vaginal bleeding	456808010	301822002	443305015
[D]Cough with haemorrhage	317408012	66857006	500542015
O/E - bleeding gums	254280011	163164001	254280011
Perinephric haematoma	4789661000006116	197824007	304163014
Haemarthrosis of elbow	310699019	202405009	310699019
Hemarthrosis of PIP joint of finger	4813401000006116	202409003	310709017
Haematoma of occipital scalp	7097721000006119	429059007	2694431010
Hemarthrosis of knee	4813531000006111	202413005	310719011
Urethral bleeding	371989018	249301002	371989018
Nontraumatic breast haematoma	4791311000006114	198123000	3012832013
Bleeding from tonsillar bed	5271551000006114	249423002	372151014
Ecchymosis of eyelid	7002461000006117	423145008	2644948013
Hyphaema	124942016	75229002	124942016
Incision and drainage of haematoma	7276661000006117	443385002	2838330015
Bleeding in mouth and/or pharynx	5271471000006115	249417007	1489865014
Post-surgical epistaxis	5035211000006119	232357009	348118015
Haemarthrosis of hand	812861000006119	27507004	483566016
Postoperative haemorrhage control	5564161000006119	274508002	410324010
Liposuction removal of hematoma	4679251000006115	177714000	275086017
Haemorrhagic choroidal detachment	479008012	19031009	479008012
Superficial retinal haemorrhage	499542018	63297008	499542018
Haematoma of pinna	507866018	88050005	507866018
Optic nerve sheath haemorrhage	1216033015	14460007	1216033015
Extradural h'ge inj no open intracran wnd+>24hr LOC+recovery	660041000006117	43216008	72101019
Nasal hemorrhage	11780441000006118	249366005	3633923016
Bleeding of subgingival space	3902881000006112	86276007	507017016

Total blood loss	3279111000006114	48149007	80230019
Abnormal bleeding/vaginal discharge	981591000006114	981591000006105	981591000006114
Subperiosteal haematoma	989951000006111	274179004	989951000006111
Recurrent microscopic haematuria	5656851000006118	281858008	419987015
[X]Haemorrhage from respiratory passages, unspecified	317999019	95431003	512121010
Respiratory tract haemorrhage	4056511000006117	95431003	4056511000006117
Haemorrhage at vitreoretinal interface	5239131000006113	247097006	369010014
Seen in haematuria clinic	1682771000006111	1682771000006107	1682771000006111
Dot AND blot haemorrhage	3288911000006114	48742005	495203014
VH - Vitreous hemorrhage	3005211000006118	31341008	2579343019
Haemarthrosis of shoulder joint	7529901000006113	700018009	2987346012
Pulmonary hemorrhage with glomerulonephritis	3319071000006118	50581000	84267019
Tracheostomy hemorrhage	3848221000006114	82872004	137462016
Haemorrhagic diarrhoea	4058311000006112	95545007	512178019
Hematoma	6438781000006118	385494008	1477038019
Adrenal hemorrhage	3294631000006115	49111001	81821014
Deformity/haematoma of pinna	5721171000006118	286943000	426436012
Intragel vitreous haemorrhage	3005181000006117	31341008	1216354019
Hemorrhage control by packing	4716051000006119	182605004	282264011
Haemarthrosis of glenohumeral joint	7529881000006111	700018009	2987361013
Haemorrhagic pancreatitis	2568891000006113	4399003	493744012
Hemarthrosis of hand	2941131000006113	27507004	46020011
Evacuation of haematoma of vagina	6531321000006117	392256003	1476301016
Pulmonary haemorrhage	3771001000006116	78144005	3771001000006116
Retroperitoneal hematoma	5088461000006119	236002003	353769018
Minor surgery postoperative complication - haemorrhage	914911000006113	914911000006109	914911000006113
Postmenopausal postcoital bleeding	2534105016	415149004	2534105016
Hemarthrosis of metacarpophalangeal joint	4813381000006116	202408006	2694346016
Acute blood loss anemia	5500931000006115	267530009	2536036012
Epistaxis control	11776911000006118	35807001	3633933012
Traumatic extradural hematoma without open intracranial wound	6053331000006117	315048006	459516011
Haemarthrosis of subtalar joint	310725010	202416002	310725010
Hip haemarthrosis	310716016	202411007	310716016
Haemarthrosis of talonavicular joint	310728012	202417006	310728012
Dysfunctional uterine hemorrhage	2803941000006119	19155002	479159011
Intra-abdominal hematoma	5088381000006115	235999009	353760019
Posterior epistaxis	5035191000006115	232355001	348116016
Evacuation of haematoma from temporal lobe of brain	265689012	171473007	265689012
Haemorrhagic fibrinogenolysis	3596661000006113	67406007	500723016
Mediastinal haematoma	2860621000006113	22625005	481391011
Retinal macroaneurysm	369052016	247124009	369052016

Haematoma of palate	5455351000006116	262649007	390623016
Subungual haematoma of foot	11784831000006115	274203005	3513825017
Recurrent frank haematuria	5656811000006119	281856007	419982014
Evacuation of perianal haematoma	433186012	293018003	433186012
Packing for non-obstetric uterine bleeding	4716101000006119	182607007	282272013
Testicular haemorrhage	1216948012	77708008	1216948012
Extradural haemorrhage following injury without open intracranial wound	402930018	43216008	493552012
Haematoma of leg	2674499019	91603007	151760014
Prostatic congestion or haemorrhage NOS	304407017	197973004	304404012
Retinal flame hemorrhage	5239731000006114	247132001	1773842011
Haematoma of testis	5681911000006114	283944002	422515011
Epistaxis	11780411000006117	249366005	3633921019
Haemarthrosis - wrist joint	890581000006114	202407001	890581000006114
Haemarthrosis - knee joint	890611000006118	202413005	890611000006118
C/O p.v. bleeding	12483181000006119	275570002	411565016
Drainage of hematoma of external ear and control cavity with bolster suture	4650071000006114	172619007	267228012
Hemorrhage of testis	3764251000006117	77708008	128969012
Acute post-haemorrhagic anaemia	5500971000006117	267530009	2536040015
Hemoptysis	11905461000006115	66857006	111056015
Persistent microscopic haematuria	5656831000006113	281857003	419984010
Anterior epistaxis	5035151000006114	232354002	348112019
Recurrent haematuria co-occurrent and due to minor glomerular abnormality	183201000006117	714823009	3300550011
Conjunctival hemorrhage	2835651000006118	21117005	35492010
Hemophthalmos (excluding current injury)	4771051000006115	193287005	297685012
Bleeding varicose vein of leg	4779581000006117	195448005	300726015
Nasal haemorrhage	11780431000006111	249366005	3633922014
Suprachoroidal haemorrhage	6939791000006112	419596007	2578131018
Haemarthrosis of first metatarsophalangeal joint	4813641000006117	202419009	2692686017
Gingival haemorrhage	3902921000006116	86276007	507021011
Hemorrhagic pancreatitis	2568881000006110	4399003	7701015
O/E - speculum bleeding through os	4563041000006113	163415000	4563041000006113
Kidney hematoma without rupture of capsule, with open wound into cavity	4855101000006116	210205007	321132016
Postoperative control of hemorrhage of adenoids	3135621000006114	39508008	66269014
Macular subretinal hemorrhage	5240211000006116	247161000	369102011
Acute post-hemorrhagic anemia	5500921000006118	267530009	2536035011
Hemorrhage from pharynx	6119821000006112	324618004	465450012
Packing for haemorrhage	4716031000006114	182605004	282265012
Essential haematuria	458617017	314280007	458617017
Control of hemorrhage of nose	3076641000006117	35807001	59727010
Drainage of hematoma of pinna	4650011000006117	172617009	267222013
Exophthalmos due to orbital hemorrhage	4773741000006115	194016009	298691011

Bleeding from breast	1805191000006113	52297004	87019010
Intraretinal hemorrhage	2966441000006114	28998008	1783893010
Hematoma of kidney	5458511000006119	262891006	390957010
Crevicular bleeding of gum	3902901000006114	86276007	507019018
Haemarthrosis of shoulder	2540431000006115	2602008	1216220017
O/E - throat hemorrhage	4577941000006112	164279008	255707017
Hemorrhage of parathyroid	4758531000006114	190460008	292687011
Testicular hemorrhage	3764271000006110	77708008	1218435011
Traumatic extradural hematoma	5459161000006119	262949005	391026017
Postoperative hematoma formation	4872571000006110	213262007	325138011
Hemarthrosis of the ankle and/or foot	5502491000006111	267948008	1477399016
Airway hemorrhage	4056541000006118	95431003	1784236019
On examination - retinal hemorrhages	4573171000006111	163987005	2668326010
Gingival crevice bleeding	3902891000006110	86276007	507018014
Haematoma of auricle	3930871000006116	88050005	507867010
Bleeding unrelated to menstrual cycle	3557191000006117	64996003	108033013
Superficial retinal hemorrhage	3529381000006112	63297008	105219016
Adrenocortical hemorrhage	5112201000006111	237766002	356329014
Stomal bleeding	5090211000006119	236129005	353961014
Other abnormal uterine and vaginal bleeding	456805013	301822002	443305015
Pleural hemorrhage	3013661000006116	31892009	53279018
Bloody diarrhea	4058331000006118	95545007	158255016
On examination - vitreous hemorrhages	4573251000006114	163990004	2668347014
History of postmenopausal bleeding	4544131000006114	161788002	2986807011
Drainage of hematoma of external ear	4650031000006111	172617009	267224014
Normocytic anaemia following acute bleed	445119014	303060002	445119014
Surgical arrest of bleeding post dental extraction	117221000006116	173330006	268351013
Haematoma of perineal wound	6357731000006110	371610007	1207307018
Control of nasal haemorrhage	3076671000006113	35807001	1490108018
Haemarthrosis of lesser metatarsophalangeal joint	812721000006118	202420003	310733011
Control of epistaxis by suture	5724371000006118	287400009	2988411019
On examination - throat hemorrhage	4577951000006114	164279008	2667890017
[X]Vitreous haemorrhage in diseases classified elsewhere	299491012	31341008	484848010
Secondary and recurrent haemorrhage	157451000006115	212373009	323899017
Recurrent haematuria co-occurrent and due to diffuse mesangiocapillary glomerulonephritis	189181000006115	714819001	3300538017
Intragel vitreous hemorrhage	3005191000006119	31341008	1217853012
Hemarthrosis of shoulder	2540441000006113	2602008	1217723011
Hyphaemia	3723201000006112	75229002	502959013
Haematoma with intact skin	405394012	105618004	169739016
Prostatic haemorrhage	1216632014	44843000	1216632014
Prostatic congestion or haemorrhage	304404012	197973004	304404012

Haemarthrosis of other specified site	12728591000006117	687801000000104	1505711000000112
Haemarthrosis of the knee	219591000000116	202413005	310720017
Perirenal haematoma	304164015	197824007	304164015
Epistaxis control - suture	860721000006116	287400009	860721000006116
Control of haemorrhage	878511000006114	51241000	878511000006114
Recurrent haematuria co-occurrent and due to dense deposit disease	304001012	714813000	3300520015
Surgical arrest of postoperative bleeding from tonsillar bed	268510012	173440008	268510012
Hemorrhage into bladder wall	2779531000006112	17615006	29772016
Re-exploration of organ and surgical arrest of postoperative bleeding NOC	184131000006117	621531000000109	1372281000000112
[X]Sequelae of other nontraumatic intracranial haemorrhage	426231000006114	363302008	482447013
Uterine hemorrhage	3116561000006117	38280009	64072010
Renal artery hemorrhage	4789521000006111	197812000	304141017
Haemarthrosis of knee	310720017	202413005	310720017
Haemarthrosis of MCP joint	310707015	202408006	310707015
Epistaxis control - cautery	5724261000006115	287382003	426914018
Pack to control postnatal haemorrhage	394488013	265641004	394488013
Hemarthrosis of distal radioulnar joint	4813301000006113	202406005	310702014
Choroidal haemorrhage and rupture	297942018	193474002	297942018
Painless haematuria	304345017	197938001	304345017
Frank haematuria	304353013	197941005	304353013
Haemorrhage of seminal vesicle	2635281000006115	8465005	506159011
Spontaneous hyphaema	5914111000006110	303019009	445086014
Secondary haemorrhage	5459051000006115	262938004	391013016
Secondary post tonsillectomy hemorrhage	6046541000006111	314484001	458850015
Haemorrhage of blood vessel	6715161000006118	405539004	2153971019
Acute haemorrhagic pancreatitis	493743018	4399003	493743018
Deep retinal haemorrhage	495202016	48742005	495202016
Liver haematoma and contusion with open wound into cavity	321093013	21580006	36197018
Epistaxis from anterior nasal septum	5035171000006116	232354002	348114018
Periorbital haematoma	2626321000006115	7927006	504059010
Traumatic intracranial haemorrhage	660121000006113	450410005	2915482010
Scrotal hematoma due to non-traumatic cause	4790931000006114	198058000	304526010
Extradural hemorrhage following injury without open intracranial wound	3196371000006118	43216008	72101019
Subcutaneous haematoma	2584481000006117	5309003	496551013
Conjunctival haemorrhage	298518014	21117005	480926011
Haematoma of skin	5625851000006111	279420009	416697010
Perioperative haematoma	3636045011	762958007	3636045011
Haematuria NOS	886851000006119	34436003	886851000006119
Chemical haematuria	5097881000006114	236718002	354812019
Haematoma of cervix	5682141000006118	283965008	422540016

Pharyngeal haemorrhage	317317013	324618004	465451011
Haematoma of scalp	7223281000006117	439461004	2794134010
Perinephric hematoma	4789671000006111	197824007	304161011
Evidence of recent epistaxis	5596491000006110	277236000	413696012
Epistaxis	11905371000006119	12441001	21406015
Macular subretinal haemorrhage	5240201000006119	247161000	369103018
Traumatic intracranial extradural hematoma	5459191000006110	262949005	2915790014
Intra-alveolar haemorrhage	3771051000006117	78144005	503766010
Vaginal introitus bleeding	5264711000006114	248978005	371563010
Pelvic hematoma	5477491000006112	264558008	393036018
Deformity/hematoma of pinna	5721181000006115	286943000	426435011
PCB - Postcoital bleeding	3290931000006116	48880000	1230662018
Subungual hematoma	5510911000006111	269219004	403015018
Cortical hemorrhage	3299621000006119	49422009	82320015
Haematoma of spleen	5457461000006118	262818004	390850011
Haematuria of undiagnosed cause	6044681000006112	314280007	458614012
Haemorrhage of thyroid	2546991000006113	3002002	484460015
Haemarthrosis of proximal interphalangeal joint of finger	4813391000006118	202409003	1772566017
Hemorrhage into cornea	5029241000006115	231916005	347516011
Bleeding from nipple	1805151000006119	248838002	371379012
Hemarthrosis of proximal interphalangeal joint of finger	4813421000006114	202409003	1773835011
Complaining of per vaginam bleeding	5574801000006117	275570002	5574801000006117
Evacuation of subungual hematoma	3939701000006111	88584000	146879013
Haemorrhagic detachment of retinal pigment epithelium	3312491000006115	50165004	495600016
Hemarthrosis-1st metatarsophalangeal joint	4813631000006110	202419009	310731013
Retinal hemorrhage	2966401000006112	28998008	48549019
Haemorrhage from the ear	2921451000006112	26322001	483175014
Secondary and recurrent haemorrhage	323899017	212373009	323899017
Aspiration of haematoma of organ NOC	279090012	621461000000103	1372141000000114
Ureteric haemorrhage	354662010	236606001	354662010
Haemorrhage control by packing	282266013	182605004	282266013
Profuse vaginal bleeding	5748551000006112	289540009	429440019
Secondary post tonsillectomy haemorrhage	458851016	314484001	458851016
Breast haematoma	5913291000006111	302924003	444949017
Diabetic vitreous haemorrhage	6022831000006119	312910009	456713019
Haemophthalmos (excluding current injury)	297686013	193287005	297686013
Renal artery haemorrhage	304142012	197812000	304142012
Haematuria - cause not known	6044721000006117	314280007	458616014
Subhyaloid haemorrhage	5239151000006118	247098001	369013011
Haemorrhage - postoperative	814171000006110	110265006	202906017
VH - Vitreous haemorrhage	3005201000006116	31341008	2579342012

Hemarthrosis of distal interphalangeal joint of finger	4813451000006117	202410008	2694113016
Haemarthrosis - knee joint	989491000006111	202413005	989491000006111
Vaginal vault bleeding	5650051000006112	281309004	419293019
Abnormal uterine bleeding unrelated to menstrual cycle	456806014	312984006	456806014
Vitreous haemorrhage	484848010	31341008	484848010
Insertion of Brighton epistaxis balloon	267563018	172821004	267563018
Haematuria/abnormal colour	962031000006113	962031000006109	962031000006113
Haemarthrosis - elbow joint	890571000006111	202405009	890571000006111
Hip hemarthrosis	4813481000006113	202411007	310714018
Anaemia due to acute blood loss	5500951000006110	267530009	2536038013
Nontraumatic hematoma of breast	4791281000006112	198123000	2619485017
Intraocular bleeding	4021601000006110	93478000	154754010
Haemoperitoneum	3236681000006116	45626005	494283018
Haematoma AND contusion of liver with open wound into abdominal cavity	2843221000006113	21580006	481063010
Peritoneal haemorrhage	3236691000006118	45626005	494284012
Evacuation of hematoma from temporal lobe of brain	4644231000006112	171473007	265688016
Prostatic congestion or hemorrhage	4790551000006114	197973004	304405013
Choroidal haemorrhage or rupture NOS	297946015	193474002	297942018
Hemarthrosis of talonavicular joint	4813611000006116	202417006	310727019
PMB - Postmenopausal bleeding	3748141000006114	76742009	503350010
Hemorrhagic fibrinogenolysis	3596641000006114	67406007	112019014
Preretinal hemorrhage	2683841000006116	11547003	19960018
Evacuation of vaginal haematoma	6531341000006112	392256003	1490236018
Haemarthrosis of tibiofibular joint	4813541000006118	202414004	310721018
Haematoma	982751000006117	982751000006101	982751000006117
Haemarthrosis - shoulder joint	890561000006116	2602008	890561000006116
Nontraumatic epidural hemorrhage	6593131000006114	397809001	1784005019
EDH - Extradural hematoma	3850541000006117	82999001	1218488017
Postoperative bleeding	4177081000006114	110265006	175035011
Nontraumatic extradural hemorrhage	6593111000006115	397809001	1774439018
Haemorrhage of dialysis arteriovenous shunt	2240911000000119	867031000000105	2240911000000119
Drainage of haematoma of pinna	4650001000006115	172617009	267225010
Surgical arrest of bleeding from internal nose OS	117211000006112	172811007	267550012
On examination - vitreous haemorrhages	4573241000006112	163990004	2668346017
Evacuation of hematoma	4305861000006115	118441006	176736013
Hemorrhage of eyelid	3536341000006111	63720000	105912017
Paroxysmal haematoma of the finger	1697911000006119	238824006	357936010
Evacuation of intracranial extradural hematoma	4645651000006115	171713001	265969013
[X]Haemorrhage, not elsewhere classified	318044011	131148009	210860014
Optic nerve haemorrhage	2729101000006110	14460007	476341017
Superficial haematoma	5518261000006115	270911002	405395013

Nasal packing for control of haemorrhage	5563931000006112	274491002	2669563011
Surgical arrest of bleeding from internal nose NOS	267560015	172811007	267550012
VTE risk assessment - active bleeding	1992421000006117	1992421000006101	1992421000006117
Secondary and recurrent hemorrhage	4868001000006115	212373009	323900010
Control of epistaxis using nasal packing	5563951000006117	274491002	2793090012
Tracheostomy haemorrhage	505306012	82872004	505306012
Haematoma of rectus sheath	451473014	308154003	451473014
Control of epistaxis by cautery	5724251000006117	287382003	2988735013
Hemophthalmos, except current injury	4771091000006114	193287005	2550869013
Hematoma of broad ligament	3855481000006115	83294006	138135015
Testicular haematoma due to nontraumatic cause	110341000006115	35644004	1216444017
Surgical arrest of post-extraction haemorrhage	117241000006111	173330006	268351013
Nontraumatic extradural intracranial hemorrhage	6593141000006116	397809001	2915638010
Haemarthrosis of sacroiliac joint	812781000006119	202412000	310717013
Bleeding from nose	317316016	249366005	372073016
Hemarthrosis of lesser metatarsophalangeal joint	4813671000006113	202420003	310734017
Splenic haematoma	5457451000006115	262818004	390849011
Hemarthrosis of sacroiliac joint	4813511000006117	202412000	310718015
Peri-operative haemorrhage or haematoma	11904821000006110	269302003	403111013
Hemorrhage of prostate	3223411000006116	44843000	74812016
Choroidal haemorrhage	2500391000006119	122003	2500391000006119
Pack to control postnatal vaginal bleeding	394487015	265641004	394487015
Evacuation of haematoma of vulva	500187011	65687007	1216830016
Recurrent haematuria co-occurrent and due to diffuse crescentic glomerulonephritis	189131000006116	714815007	3300526014
Postoperative haemorrhage	217221000006118	110265006	202906017
Hematoma with intact skin	5518211000006118	270911002	405389012
Haemopericardium	39303019	23412002	39303019
Gingival crevicular bleeding	3902871000006114	86276007	507016013
Intramuscular haematoma	768621000006112	262969000	391070014
Intraocular haemorrhage	769531000006117	93478000	510363011
Extradural h'ge inj no open intracranial wnd+<1hr loss consc	660101000006115	43216008	72101019
Extradural h'ge inj no open intracranial wnd + no loss consc	660081000006111	43216008	72101019
Haemorrhage of corpus cavernosum	3629431000006110	69452005	501277010
Haemarthrosis of other tarsal joint	310729016	267948008	400242010
Preretinal haemorrhage	207961000006117	11547003	411993013
Haemarthrosis of the forearm	812851000006116	202407001	310703016
Incision and drainage of breast haematoma	5570921000006110	275236004	411190012
Evacuation of hematoma of vulva	3568151000006118	65687007	109140016
Corpus cavernosum hemorrhage	3629461000006118	69452005	1218360010
Packing for hemorrhage	4716071000006112	182605004	282267016
Haemarthrosis - multiple joint	890641000006119	76427005	890641000006119

Peritoneal hemorrhage	3236661000006114	45626005	76084011
Hemarthrosis of multiple sites	3743251000006114	76427005	126927017
Pulmonary hemorrhage	11905681000006111	78144005	129684011
Nontraumatic breast hematoma	4791301000006111	198123000	3012814013
Acute blood loss anaemia	5500961000006112	267530009	2536039017
Retrobulbar hemorrhage	4774381000006112	194179009	298913011
Hemorrhagic choroidal detachment	2802071000006118	19031009	32080012
Pneumohaemothorax	2763821000006115	16632002	28150019
On examination - bleeding gums	4558931000006115	163164001	2667369016
Bleeding after menopause	3748151000006111	76742009	503351014
Drainage of hematoma of pinna and insertion of bolster sutures	4650091000006110	172619007	267231013
Evacuation of vaginal hematoma	6531351000006114	392256003	1490465014
Haemorrhage of dialysis arteriovenous fistula	2240871000000116	867011000000102	2240871000000116
Haematuria - symptom	813391000006111	34436003	57480016
Haemarthrosis of distal radioulnar joint	812671000006110	202406005	310701019
Haemarthrosis-1st metatarsophalangeal joint	812951000006113	202419009	310732018
H/O: postcoital bleeding	811291000006116	161786003	252099013
Scrotal haematoma due to non-traumatic cause	156461000006116	198058000	304525014
Recurrent haematuria co-occurrent and due to focal and segmental glomerular lesions	189151000006111	714827005	3300562010
Hemarthrosis of shoulder region	2540421000006118	2602008	5429017
Retrobulbar hematoma	4774391000006110	194179009	298914017
Male genital haematoma NOS	304528011	198057005	304524013
Functional uterine haemorrhage NOS	305075011	19155002	32278010
Complaining of per vaginam bleeding	411565016	275570002	2984033019
Recurrent and persistent haematuria, dense deposit disease	11904541000006117	281860005	189231000006110
Subretinal hemorrhage	2721051000006114	13937002	23758016
Deep intra-retinal haemorrhages	3288901000006111	48742005	495201011
Internal prosthetic device causing hemorrhage	4871651000006112	213133000	324953013
Airway haemorrhage	4056531000006111	95431003	1783845016
Vaginal haematoma	496575013	53162000	496575013
Anti-GBM nephritis with pulmonary hemorrhage	3319091000006117	50581000	84270015
Hemarthrosis of the ankle	4813571000006114	202415003	310723015
[X]Other specified abnormal uterine and vaginal bleeding	305260014	301822002	443305015
Contact bleeding from cervix	590531000006114	248980004	371565015
Retinal blot haemorrhages	3288891000006112	48742005	495200012
Subconjunctival hemorrhage	3781231000006112	78768009	130708019
Microscopic haematuria	304349011	197940006	304349011
Liver haematoma	739711000006114	262796008	390818018
Liver haematoma	12704641000006114	262796008	390819014
Hemarthrosis of shoulder joint	7529891000006114	700018009	2987325015
Haemarthrosis - other joint	890631000006112	687801000000104	890631000006112

Choroidal haemorrhage	297943011	122003	1256014
Extradural h'ge inj no open intracran wnd+>24hr LOC-restored	660051000006115	43216008	72101019
Haemarthrosis of the lower leg	812881000006112	202413005	310719011
Epidural haematoma	7583041000006117	703861005	3010111010
Bleeding of oral mucosa	5271491000006119	249418002	372144017
Control of epistaxis by unlisted technique	3076661000006118	35807001	59732011
Control of haemorrhage	3330341000006112	51241000	495965010
Hematoma of perineal wound	6357741000006117	371610007	1208119015
Closed heart injury with haemopericardium	455462013	311830005	455462013
Tanner devascularisation for bleeding varices	114801000006118	173617001	268812014
Traumatic retinopathy	5030871000006117	232030000	347668010
Haemorrhage of dialysis vascular access	2237901000000119	865801000000100	2237901000000119
Extradural haemorrhage	3850521000006112	82999001	505380015
Spleen haematoma without mention of open wound into cavity	135631000006119	262818004	390849011
Elbow hemarthrosis	4813271000006111	202405009	310698010
Haematoma	813271000006118	385494008	1476077014
Ureteric hemorrhage	5096531000006113	236606001	354661015
Choroidal hemorrhage	2500401000006117	122003	1256014
Haemorrhage from ureter	5096511000006119	236606001	354660019
[X]Sequelae of stroke,not specfd as h'morrhage or infarction	426321000006116	195239002	300403014
Haemopneumothorax	477873012	16632002	477873012
Haematoma of vulva	501268015	69385001	501268015
Bleeding from urethra	371988014	249301002	371988014
Irregular uterine bleeding	3804361000006111	80182007	504362017
Kidney haematoma without mention of open wound into cavity	754831000006113	262891006	390956018
Kidney haematoma without rupture of capsule, with open wound into cavity	754821000006110	210205007	321133014
Hemorrhagic diarrhea	4058321000006116	95545007	158254017
Intraretinal haemorrhage	2966431000006116	28998008	1783522015
Haemarthrosis of the ankle and/or foot	5502461000006115	267948008	1476436016
Choroidal haemorrhage	12702351000006114	122003	469795010
Extradural h'ge inj no open intracranial wnd + unspec consc	660091000006114	262949005	391026017
Hyphaema of right eye	3526020015	680521000119101	3526020015
Haemarthrosis of shoulder	1216220017	700018009	2987325015
Epistaxis symptom	647631000006114	249366005	372073016
Has nosebleeds - epistaxis	816991000006115	249366005	372073016
Subperiosteal haematoma	933081000006112	669341000000103	933081000006112
Nontraumatic haematoma of testis	3073981000006111	35644004	486230013
Retinal flame haemorrhage	5239721000006111	247132001	1772573010
Hemorrhage in uterus	3116551000006119	38280009	64071015
Hematoma of auricle	3930861000006111	88050005	145964013

Perirenal hematoma	4789681000006114	197824007	304162016
Postoperative hemorrhage control	5564171000006114	274508002	410325011
Hemorrhage of corpus cavernosum	3629441000006117	69452005	115380016
Secondary and recurrent haemorrhage	157461000006118	212373009	323899017
Reopening of abdomen and re-exploration of intra-abdominal operation site and surgical arrest of postoperative bleeding	177571000006110	177956008	275461019
Renal haematoma with open wound into cavity	176891000006112	210205007	321133014
Postoperative haematoma formation	217211000006114	213262007	325137018
Hemarthrosis of interphalangeal joint of toe	4813691000006114	202421004	310735016
Gingival hemorrhage	3902911000006112	86276007	507020012
Hemarthrosis of the ankle and foot	5502471000006110	267948008	400242010
Reopening of abdomen and reexploration of intra-abdominal operation site and surgical arrest of postoperative bleeding	4680671000006110	177956008	2164235018
Intramuscular hematoma	5459481000006117	262969000	391071013
Chronic haematoma of pinna	2950691000006112	28072004	1216250013
Bleeding hemorrhoids	3335281000006117	51551000	85856015
Painful haematuria	304348015	197939009	304348015
Breast haematoma due to non-traumatic cause	525281000006114	198123000	304630018
Extradural h'ge inj no open intracra wnd+LOC unspec duration	660031000006110	262949005	391026017
Peri-operative hemorrhage or hematoma	5511401000006112	269302003	403112018
Evacuation of extradural haematoma	649541000006113	171713001	265968017
[X]Haemorrhage from other sites in respiratory passages	12469521000006114	397271000000104	388341000006114
Postoperative control of haemorrhage of adenoids	12705111000006117	39508008	492111018
Flame-shaped hemorrhage	12222151000006110	247132001	3644831017
Subconjunctival haemorrhage	503913016	78768009	503913016
Liver haematoma and contusion without open wound into cavity	11989661000006117	262796008	11989661000006117
Thyroid hemorrhage	2547021000006110	3002002	1217810012
Haemarthrosis of the upper arm	812911000006112	202405009	310697017
Retinal dot haemorrhage	5239801000006114	247133006	1772574016
Subchorionic haematoma	7494711000006110	609204004	2958468013
Ventricular haemorrhage	2871241000006118	23276006	481581018
Vaginal bleeding	1783697014	289530006	2983956017
Bleeding from mouth	2858321000006110	22490002	37748013
Subchondral haematoma	5651931000006113	281464006	419472015
Epistaxis from Little's area	5035181000006118	232354002	348115017
Pneumohemothorax	2763831000006117	16632002	28152010
Hyphemia	3723191000006114	75229002	124941011
Intra-alveolar hemorrhage	3771021000006114	78144005	129685012
Hemarthrosis of elbow	4813261000006116	202405009	310697017
Abnormal uterine and vaginal bleeding, unspecified	456809019	312984006	456806014
Extradural hemorrhage after injury	5459171000006114	262949005	391027014

Thyroid haemorrhage or infarction NOS	292445017	190305000	292443012
Corneal hemorrhage	5029231000006113	231916005	347515010
Haemarthrosis of interphalangeal joint of toe	812701000006111	202421004	310736015
O/E - subconjunctival haemorrhage	270631000006114	162815002	253783017
Hemarthrosis of tibiofibular joint	4813551000006116	202414004	310722013
Haemorrhage into cornea	5029221000006110	231916005	347514014
Superficial hematoma	5518221000006114	270911002	405390015
Renal haematoma without mention of open wound into cavity	176901000006111	262891006	390956018
Haemarthrosis of shoulder region	812901000006114	2602008	483092019
Haemarthrosis of tibio-fibular joint	812921000006116	202414004	310721018
Epistaxis control by cryosurgery	5724341000006114	287393001	426925010
Hemophthalmos without current injury	4771071000006113	193287005	2547719019
O/E - retinal haemorrhages	255337011	163987005	255337011
Splenic hematoma	5457471000006113	262818004	390851010
Abnormal uterine bleeding	456807017	44991000119100	2995739016
Intraoperative hemorrhage	4872551000006117	213261000	325135014
Bleeding after intercourse	3290921000006119	48880000	1230661013
Male genital haemorrhage NOS	396748016	198057005	304524013
Recurrent haematuria co-occurrent and due to diffuse endocapillary proliferative glomerulonephritis	189161000006113	714817004	3300532016
Excessive bleeding at onset of menopause	3936961000006118	88424000	146605017
Bleeding from ear	2921411000006111	26322001	483174013
Hemorrhage	4454751000006112	131148009	3035867011
Extradural h'ge inj no open intracran wnd+concussion unspec	660071000006113	262949005	391026017
Urethral meatal bleeding during urinary catheterisation	1938681000006112	1938681000006108	1938681000006112
Wound haematoma	5131541000006113	239160006	358450014
Arterial haemorrhage	5058751000006119	234003006	350613018
Delayed/excessive haemorrhage	887711000006114	198827002	887711000006114
Drainage of haematoma of external ear and control cavity with bolster suture	628481000006113	172619007	267230014
Scrotal hematoma	3962431000006110	89966002	149129014
Hemorrhage control	3330371000006116	51241000	1490495018
Drainage of subungual haematoma	508104019	88584000	508104019
Evacuation of vulval hematoma	3568171000006111	65687007	1218318011
Heavy episode of vaginal bleeding	459713018	315224006	459713018
Bladder hemorrhage	4790081000006111	197887003	304254016
Haemorrhage	300840014	131148009	3035879011
Recurrent haematuria	303986013	281860005	419991013
Optic nerve hemorrhage	2729091000006116	14460007	24580011
Pharyngeal haemorrhage	6119801000006119	324618004	6119801000006119
Haematoma - perineal wound	813251000006111	371610007	1207307018
Haemarthrosis of ankle	812651000006117	202415003	310724014

Retinal pigment epithelium hemorrhagic detachment	3312521000006118	50165004	1218183019
Drainage of haematoma of external ear	267223015	172617009	267223015
Haematuria	57480016	34436003	57480016
Chronic hematoma of pinna	2950701000006112	28072004	1217750018
Epidural intracranial haemorrhage	3850551000006115	82999001	2915908018
Haemorrhage into bladder wall	478383018	17615006	478383018
Subungual Haematoma	1576441000006114	1576441000006105	1576441000006114
Recurrent haematuria co-occurrent and due to diffuse membranous glomerulonephritis	189141000006114	714821006	3300544018
Retroperitoneal haematoma	353768014	236002003	353768014
On examination - epistaxis	255582011	164187003	2667170012
Epistaxis control	860691000006114	35807001	860691000006114
Bladder haemorrhage	304253010	197887003	304253010
Submembranous retinal haemorrhage	2683861000006117	11547003	411995018
Nontraumatic extradural haemorrhage	660111000006117	397809001	1773153015
Traumatic extradural haematoma without open intracranial wound	86021000006119	315048006	459515010
Prostatic hemorrhage	3223431000006110	44843000	1218121019
Gingival bleeding	3902861000006119	86276007	143081017
Intrarenal haematoma	304143019	197813005	304143019
Clot haematuria	304355018	197942003	304355018
Post-op. haemorrhage control	878521000006118	274508002	878521000006118
Haemoperitoneum - non-traumatic	813971000006111	45626005	494282011
H/O: postmenopausal bleeding	811301000006115	161788002	252102013
Haemarthrosis of the pelvic region and thigh	812891000006110	202411007	310713012
Postoperative control of haemorrhage of adenoids	1543141000006110	39508008	66269014
Bulbar haemorrhage	483988011	732923001	3467313018
Haemarthrosis - shoulder joint	989471000006110	2602008	989471000006110
Spleen injury with haematoma without rupture of capsule, with open wound into cavity	135621000006117	210190001	321114016
Haemorrhage of dialysis arteriovenous graft	2240831000000118	866991000000105	2240831000000118
Recurrent haematuria co-occurrent and due to diffuse mesangial proliferative glomerulonephritis	189171000006118	714825002	3300556017
Expulsive choroidal haemorrhage	420970014	11623000	420970014
Drainage of breast haematoma	5106761000006119	237402002	355796018
Acute haematoma of pinna	5912831000006117	302906000	444900012
Hemarthrosis of sternoclavicular joint	4813221000006110	202403002	310693018
Haemorrhage of scrotum	3510201000006112	62182006	499223018
Retinal haemorrhage	297864014	28998008	484055013
Bleeding from the ear	2921431000006117	26322001	44085018
Hematoma of rectus sheath	5975711000006116	308154003	451472016
Bleeding - vaginal NOS	371562017	289530006	2983956017
Haemarthrosis - elbow joint	989481000006113	202405009	989481000006113
Haemorrhage in uterus	3116591000006113	38280009	489888015

Finding of bleeding of nose	5270651000006118	249366005	1224241013
Epidural intracranial hemorrhage	3850561000006118	82999001	2916559017
Evacuation of haematoma	279096018	118441006	444575012
Choroidal hemorrhage and rupture	4771941000006116	193474002	297941013
Intrarenal hematoma	4789541000006116	197813005	304144013
Breast hematoma due to non-traumatic cause	4791271000006114	198123000	304631019
Haemopericardium as current complication following acute myocardial infarction	813961000006116	194862000	299816014
Packing for bleeding	4716041000006116	182605004	282263017
Nosebleed/epistaxis symptom	297191000006112	249366005	372073016
O/E - vitreous haemorrhages	255341010	163990004	255341010
Acute posthemorrhagic anemia	5500911000006114	267530009	399227018
Anemia due to acute blood loss	5500941000006113	267530009	2536037015
Haemarthrosis of the ankle and foot	400243017	267948008	400243017
Epistaxis control - cautery	860711000006112	287382003	860711000006112
Retrobulbar haemorrhage	298916015	194179009	298916015
Extradural h'ge inj no open intracran wnd+1-24hr loss consc	660061000006118	43216008	72101019
Unspecified choroidal haemorrhage	11989321000006119	122003	11989321000006119
Postmenopausal bleeding	127423017	76742009	127423017
Epistaxis control - suture	5724381000006115	287400009	426932018
Hematoma AND contusion of liver with open wound into abdominal cavity	2843231000006111	21580006	36197018
Pulmonary haemorrhage	317409016	78144005	503767018
Control of epistaxis	3076651000006115	35807001	59731016
Drainage of haematoma of nasal septum	2753321000006117	15962000	477593011
Epistaxis control NOS	860741000006111	35807001	860741000006111
Pericardial haematoma evacuat.	865621000006117	562891000000104	865621000006117
Subperiosteal haematoma	6552151000006115	395183000	1476344017
Spontaneous haemarthrosis	5650711000006114	281370001	419361012
Epistaxis control	5724171000006115	287374003	426905011
Persistent frank haematuria	5656791000006118	281855006	419981019
Subcapsular haematoma of spleen	5065651000006112	234507003	351359017

Table S39. Bleeding at other anatomy sites HES (ICD10) codes

ICD10 codes	Term
D62	Acute posthaemorrhagic anaemia
D68.3	Haemorrhagic disorder due to circulating anticoagulants
D69.8	Other specified haemorrhagic conditions
D69.9	Haemorrhagic condition, unspecified
H11.3	Conjunctival haemorrhage
H21.0	Hyphema
H31.3	Choroidal haemorrhage and rupture
H35.6	Retinal haemorrhage
H43.1	Vitreous haemorrhage
H45.0	Vitreous haemorrhage in diseases classified elsewhere
H92.2	Otorrhagia
I23.0	Haemopericardium as current complication following acute myocardial infarction
I31.2	Haemopericardium, not elsewhere classified
I71.1	Thoracic aortic aneurysm, ruptured
I71.3	Abdominal aortic aneurysm, ruptured
I71.5	Thoracoabdominal aortic aneurysm, ruptured
J94.2	Haemothorax
K76.2	Central haemorrhagic necrosis of liver
K92.0	Haematemesis
K92.1	Melaena
M25.0	Haemarthrosis
M25.00	Haemarthrosis, unspecified joint
M25.01	Haemarthrosis, shoulder
M25.02	Haemarthrosis, elbow
M25.03	Haemarthrosis, wrist
M25.04	Haemarthrosis, hand
M25.05	Haemarthrosis, hip
M25.06	Haemarthrosis, knee
M25.07	Haemarthrosis, ankle and foot
M25.08	Haemarthrosis, other specified site
N02.0	Recurrent and persistent haematuria. Minor glomerular abnormality
N02.1	Recurrent and persistent haematuria. Focal and segmental glomerular lesions
N02.2	Recurrent and persistent haematuria. Diffuse membranous glomerulonephritis
N02.3	Recurrent and persistent haematuria. Diffuse mesangial proliferative glomerulonephritis
N02.4	Recurrent and persistent haematuria. Diffuse endocapillary proliferative glomerulonephritis
N02.5	Recurrent and persistent haematuria. Diffuse mesangiocapillary glomerulonephritis
N02.6	Recurrent and persistent haematuria. Dense deposit disease

N02.7	Recurrent and persistent haematuria. Diffuse crescentic glomerulonephritis
N02.8	Recurrent and persistent haematuria. Other
N02.9	Recurrent and persistent haematuria. Unspecified
N42.1	Congestion and haemorrhage of prostate
N83.6	Hematosalpinx
N83.7	Haematoma of broad ligament
N85.7	Hematometra
N89.7	Hematocolpos
N93.0	Postcoital and contact bleeding
N93.8	Other specified abnormal uterine and vaginal bleeding
N93.9	Abnormal uterine and vaginal bleeding, unspecified
N95.0	Postmenopausal bleeding
R04.0	Epistaxis
R04.1	Haemorrhage from throat
R04.2	Haemoptysis
R04.8	Haemorrhage from other sites in respiratory passages
R04.9	Haemorrhage from respiratory passages, unspecified
R31	Unspecified haematuria
R58	Haemorrhage, not elsewhere classified
S26.0	Injury of heart with haemopericardium
T81.0	Haemorrhage and haematoma complicating a procedure, not elsewhere classified

Table S40. Hospitalisation Aurum codes

Term	Medcode ID	SNOMED CT Concept ID	SNOMED CT Description ID
Admit geriatric emergency	12482071000006111	183457004	283525018
Admission to colorectal surgery department	5940241000006118	305422004	447841011
Admission by surgeon	5938551000006116	305293001	447669018
Night hospital care	283555013	183483009	283555013
Refer to hospital casualty	283795013	183659006	283795013
Admission to medical department	5939401000006112	305354007	447756010
Admission to ophthalmology department	5940281000006112	305426001	447845019
Rheumatology emergency hospital admission	283536013	183465001	3082921015
Discharge from Inpatient Care	8049681000006116	163391000000107	214171000000113
Emergency hospital admission to general surgical service	8453071000006119	1078041000000108	2702931000000114
Patient in hospital	284397011	184091000	284397011
Patient died in hospital	284637019	184297005	284637019
Refer to hospital	2476476011	310449005	2476476011
Discharged from accident and emergency	2549670013	417119002	2549670013
Discharge summary awaited	21521000000115	17301000000103	21521000000115
Admit to intensive care unit	447747019	305351004	447747019
Admission by assistant GP	967731000000117	449201000000100	967731000000117
Hospital admission, short-term	3321091000006111	50699000	84496015
Place to which Pt admitted (Gen Surg)	1594641000006111	1594641000006107	1594641000006111
Hospital admission, transfer from other hospital or health care facility	2571671000006112	4563007	7859016
Inpatient stay 6 days	783711000006116	183804009	284001011
Admission by locum general practitioner	1823591000006110	476981000000106	1690371000000119
Hospital inpatient note	1898071000006117	1898071000006101	1898071000006117
Discharge to rented accommodation	1951041000006116	1951041000006100	1951041000006116
Hospital Inpatient	1563381000006113	1563381000006109	1563381000006113
Accident and Emergency department discharge to neonatal intensive care unit	8439411000006116	1066401000000108	2680731000000115
Place to which Pt admitted (Ophth)	1598011000006110	1598011000006106	1598011000006110
Hematology emergency hospital admission	4723611000006111	183470008	3082879019
Vascular surgery emergency hospital admission	7517441000006118	699122005	7517441000006118
Admission by co-operative general practitioner	8129421000006110	388751000000101	8129421000006110
Discharged from private hospital	4725511000006111	183670004	4725511000006111
Emergency department discharge to ICU (intensive care unit)	8439381000006119	1066391000000105	2678241000000110
Post hospital discharge followed up within 3 days	2391861000000116	933601000000101	2391861000000116
Admit trauma emergency	12482281000006119	183463008	283534011
Post-operative assessment at 4-6 hours after secondary care discharge	1628141000006112	298941000000105	528521000000113
Admission by deputising general practitioner	1845391000006119	395581000000108	962331000000116
Ophthalmological emergency hospital admission	4723471000006117	183464002	4723471000006117
Admit cardiology emergency	12482021000006110	313385005	457274011

Discharged - treatment completed	968851000006112	968851000006108	968851000006112
[RFC] Contact after hospital discharge/ A&E visit	910381000006112	910381000006108	910381000006112
Refer to hospital eye casualty	184781000006115	183661002	283798010
Death in hospital	608281000006119	183676005	283820019
Admission to accident and emergency department	1230914014	50849002	1230914014
Admission to neurological intensive care unit	283516012	183448005	2900917012
Cardiothoracic emergency hospital admission	283549015	183477006	3082969017
Inpatient stay 5 days	783701000006119	183803003	284000012
Admission by orthopaedic surgeon	5938821000006111	305314005	447700011
Admit other unit	949781000006113	949781000006109	949781000006113
Emergency hospital admission to general medical service	8453341000006112	1078291000000109	2703431000000113
Admission to ward	5939171000006112	305342007	447733018
Discharge from hospital	5976961000006114	308283009	451609018
Admission to gastroenterology department	5939531000006117	305367005	447769014
Facilitated early discharge from hospital	726311000000112	343961000000102	726311000000112
Admission by ophthalmologist	5938791000006119	305312009	447696015
Hospital inpatient report	62131000000111	24661000000108	62131000000111
Discharge from intermediate care	62671000000115	25131000000105	62671000000115
Urology emergency hospital admission	4723551000006111	183468004	4723551000006111
Admission to gastrointestinal surgery department	5940211000006117	305419001	447838019
Admission by casualty doctor	5937711000006113	305226003	447581010
Discharge to hospital at home service	8439271000006114	1066351000000102	2678131000000114
Admit vascular surgery emergency	12491011000006112	699122005	2982822015
Admission to respiratory intensive care unit	4723181000006114	183447000	4723181000006114
Date Pt admitted (Musc/Skel)	1596111000006113	1596111000006109	1596111000006113
Place to which Pt admitted (Musc/Skel)	1596121000006117	1596121000006101	1596121000006117
Renal medicine emergency hospital admission	4723701000006118	183475003	4723701000006118
Emergency department discharge to ambulatory emergency care service	8439251000006116	1066341000000100	2678111000000118
Emergency room admission	3323561000006114	50849002	84735018
Discharge letter faxed to general practitioner	1756601000006113	1756601000006109	1756601000006113
[V]Delayed discharge - social services	1227761013	309568009	453018012
Discharge to care home	1951031000006114	1951031000006105	1951031000006114
Discharge to other establishment	1951251000006111	1951251000006107	1951251000006111
Discharge to acute hospital	1951201000006112	1951201000006108	1951201000006112
Cardiothoracic emergency hospital admission	4723741000006116	183477006	4723741000006116
Acute secondary care intervention on development of symptoms	2180221000000114	840121000000102	2180221000000114
Discharge letter given to patient	1693771000000110	763571000000107	1693771000000110
Hospital discharge letter received	2343861000000113	911911000000100	2343861000000113
Accident and Emergency department discharge to CCU (coronary care unit)	8439331000006115	1066371000000106	2680701000000114
Neurology emergency hospital admission	4723531000006116	183467009	4723531000006116
Admission by co-op GP	12491231000006111	388751000000101	851091000000111

Admission by accident and emergency doctor	447580011	305226003	1775689013
Trauma emergency hospital admission	4723451000006110	183463008	4723451000006110
Admission to cardiac intensive care unit	4723161000006116	183446009	4723161000006116
Discharge from A & E service	5957881000006110	306563004	1785121019
Delayed discharge to nursing home	283813014	183672007	283813014
Inpatient care	1488379018	394656005	1488379018
Urology emergency hospital admission	283539018	183468004	3082876014
Place to which Pt admitted (General Medicine)	1594091000006112	1594091000006108	1594091000006112
Long stay hospital inpatient	736561000006112	160741006	250569018
Inpatient stay longer than 12 hours	783601000006114	183798007	283995016
Place to which Pt admitted (Gastro)	1593541000006113	1593541000006109	1593541000006113
Discharge from Accident and Emergency service	5957861000006117	306563004	1775696010
Admission to cardiology department	5939431000006116	305357000	447759015
Inpatient surgery care	3524772015	736774000	3524772015
Discharge from adult intensive care service	5957951000006111	306569000	449638012
Emergency hospital admission to gastroenterology service	8453311000006113	1078261000000103	2703371000000116
Discharge by Accident and Emergency doctor	5955681000006119	306390007	1775695014
Admit haematology emergency	12482091000006112	183470008	283541017
Emergency hospital admission to thoracic surgery service	8453021000006115	1077991000000101	2702801000000111
Discharge to day ward	5959671000006119	306707002	449818014
Discharged from inpatient care	283805013	183667003	283805013
Emergency hospital admission to accident and emergency service	8458471000006117	1082421000000101	2712531000000116
Neurology emergency hospital admission	283538014	183467009	3082917010
Admission by ear, nose and throat surgeon	5938651000006115	305302002	447682014
Inpatient Care and Treatment Review	8433711000006113	1060741000000104	2666481000000111
Hospital inpatient	397755014	266938001	397755014
Admit to respiratory ITU	12482271000006117	183447000	283515011
Admission by urologist	5938901000006112	305320006	447707014
24 hours post admission	5650851000006116	281380002	419373012
Admission to neurological intensive care unit	4723201000006110	183448005	4723201000006110
Refer to hospital casualty NOS	283801016	183659006	283795013
Accident and Emergency department discharge to SCBU (special care baby unit)	8439361000006112	1066381000000108	2680741000000112
Vascular surgery emergency hospital admission	1137891000000117	699122005	3082834018
Admission with abdominal symptoms	1838001000006117	1838001000006101	1838001000006117
Date admit other unit	949961000006117	949961000006101	949961000006117
DOA (dead on arrival) at hospital	3528421000006114	63238001	2121181000000118
Discharged from community hospital	1562411000000110	712671000000101	1562411000000110
Admit hospital emergency NOS	283552011	183452005	283520011
Admission by general practitioner	5937781000006118	305230000	2692897015
Post-operative assessment at 12-24 hours after secondary care discharge	1627141000006113	298971000000104	528581000000114

Discharged from hospital	283803018	183665006	283803018
Post-operative assessment at 6 weeks after secondary care discharge	1768661000006118	760301000000104	1681501000000110
Post hospital discharge medication reconciliation with medical notes	1753671000006114	730061000000107	1601761000000119
Appropriate non-elective hospital admission	1955941000006117	1955941000006101	1955941000006117
Accident and Emergency department discharge to ICU (intensive care unit)	8439391000006116	1066391000000105	2680721000000117
Admission to hospital	1227951014	32485007	1227951014
Discharge letter emailed to general practitioner	1706631000000118	766251000000108	1706631000000118
Inpatient stay 3 days	783681000006117	183801001	283998019
Discharge letter sent to GP (general practitioner)	8090731000006111	294331000000109	1676661000000111
Geriatric emergency hospital admission	283525018	183457004	3082885014
Admission to cardiac intensive care unit	283514010	183446009	2901401018
Inpatient stay 2 days	783671000006115	183800000	283997012
Patient self-discharge	242581000006111	183955003	284179014
Adjustment reaction due to hospitalisation	461701000006114	192065003	295515011
Discharge to private nursing home	5959551000006111	306695007	449802015
Trauma emergency hospital admission	283534011	183463008	3082906014
Renal medicine emergency hospital admission	283547018	183475003	3082929018
Admit to intensive c.u. NOS	283519017	305351004	447746011
Discharge to tertiary referral hospital	5959621000006115	306703003	449811015
Admission to neurology department	5939691000006119	305379005	447786015
Oral surgical emergency hospital admission	4723661000006114	183473005	4723661000006114
Discharged from hospital within 6 hours of delivery	283814015	183673002	283814015
Admission to department	5939271000006117	305349003	447743015
Cardiology emergency hospital admission	457274011	313385005	3082972012
Inpatient stay 4 days	783691000006119	183802008	283999010
Admission to stroke unit	449935012	306803007	449935012
Admission to general medical department	5939541000006110	305368000	447770010
Discharge report	63261000000118	25571000000103	63261000000118
Admission by GP registrar	851111000000118	424211000000104	851111000000118
Received hospital death discharge letter	193281000006110	184277000	284626015
Ophthalmological emergency hospital admission	462221000006116	183464002	3082894015
Discharge to ward	5959661000006114	306706006	449817016
Emergency department discharge to high dependency unit	8439281000006112	1066361000000104	2678161000000116
Admission by A & E doctor	5937721000006117	305226003	447582015
Patient transfer, in-hospital, service-to-service	1846191000006113	57976004	96383017
Discharged from private hosp'l	12484901000006111	183670004	283811011
Admission by own GP	1823541000006118	305231001	1823541000006118
Admission by associate GP (general practitioner)	1823571000006114	437171000000107	1674951000000110
Admission by assistant GP (general practitioner)	1823561000006119	449201000000100	1674411000000118
Patient transfer from hospital to hospital	1825121000006116	239531000000108	1825121000006116
Neurosurgical emergency hospital admission	4723721000006111	183476002	4723721000006111

Discharge letter emailed to general practitioner	1756571000006118	1756571000006102	1756571000006118
Post hospital discharge medication reconciliation with patient	1753661000006119	730021000000104	1601671000000117
Hospital death disch. NOS	284628019	309039003	452370013
Hospital death discharge notification	828231000006117	184274007	284623011
Admit to ITU	462351000006119	305351004	447746011
Admit surgical emergency unsp.	462321000006111	183452005	283520011
Admit medical emergency unsp.	462181000006113	183452005	283520011
Hospital re-admission	1782016	417005	1782016
Admission to respiratory intensive care unit	283515011	183447000	2902327017
Haematology emergency hospital admission	283541017	183470008	3082962014
Inpatient stay 1 day	783611000006112	183799004	283996015
Accident and Emergency department discharge to ambulatory emergency care service	8439261000006119	1066341000000100	2680751000000110
Inpatient final discharge letter	8271641000006116	824331000000106	2145271000000110
Admission to general gastrointestinal surgery department	5940221000006113	305420007	447839010
Taking own discharge	5957831000006114	306560001	449625016
Reason for admission	8310281000006117	886861000000108	2285241000000112
Admission to adult intensive care unit	5939341000006118	305352006	447750016
Admitted other	950751000006110	950751000006106	950751000006110
Admission to respiratory medicine department	5939461000006113	305360007	447762017
Admit ENT emergency	12482061000006116	183462003	283533017
Referral to inpatient unit	2729321000000110	1089301000000106	2729321000000110
On admission	5611821000006112	278307001	415178019
[V]Delayed discharge - nursing home vacancy awaited	1227760014	183930009	284147017
Admission to thoracic medicine department	5939451000006111	305359002	447761012
A&E attendance - discharged no follow up treatment required	1981781000006116	1981781000006100	1981781000006116
Admission by GP	447587014	305230000	447587014
Discharge to community hospital	449808016	306701001	449808016
Premature hospital discharge	1918091000006114	1918091000006105	1918091000006114
Admit cardiothoracic emergency	12482031000006113	183477006	283549015
Date Pt admitted (Cardio)	1592091000006115	1592091000006104	1592091000006115
Emergency department discharge to coronary care unit	8439311000006114	1066371000000106	2678191000000110
Emergency department discharge to CCU (coronary care unit)	8439321000006118	1066371000000106	2678181000000113
Discharged from community hospital	1744481000006110	1744481000006106	1744481000006110
Patient transfer from hospital to hospital	1156751000000119	239531000000108	1156751000000119
Admission by GP partner	1823551000006116	305232008	1823551000006116
Admission by GP (general practitioner) registrar	1823581000006112	424211000000104	1675721000000119
Premature hospital discharge	2444251000000115	958801000000109	2444251000000115
Discharge letter faxed to general practitioner	1693731000000113	763551000000103	1693731000000113
Place to which Pt admitted (Cardio)	1592101000006114	1592101000006105	1592101000006114
Date Pt discharged (Gastro)	1593561000006112	1593561000006108	1593561000006112

Date Pt admitted (General Medicine)	1594081000006114	1594081000006105	1594081000006114
Admit ophthalmological emergency	12482131000006114	183464002	283535012
Admit anticoagulation emergency	2198591000000112	848101000000108	2198591000000112
Gynaecological emergency hospital admission	4723341000006117	183459001	4723341000006117
Admit neurosurgical emergency	12482111000006115	183476002	283548011
Admit to cardiac ITU	12482241000006113	183446009	283514010
Admitted	949761000006115	949761000006104	949761000006115
Inpatient stay 12 days	783641000006111	183810009	284007010
ENT emergency hospital admission	283533017	183462003	3082975014
Admission to oral surgery department	5940291000006110	305427005	447847010
Orthopaedic emergency hospital admission	283532010	183461005	3082948019
Discharge letter	8270971000006113	823701000000103	2143921000000116
Emergency hospital admission to stroke service	8453821000006113	1078671000000108	2704231000000114
Admission to hepatobiliary surgical department	5940261000006119	305424003	447843014
Discharge letter offered to patient	8306951000006115	879761000000102	2269661000000111
Emergency department discharge to intensive care unit	8439371000006117	1066391000000105	2678251000000113
Discharge summary	1212385013	373942005	1212385013
Discharge from intensive care service	5957941000006114	306568008	449637019
Admission to gynaecology department	5939761000006118	305385003	447793016
Hospital admission, emergency, from emergency room, medical nature	2816831000006113	19951005	33588016
Discharge to residential home	449798016	306691003	449798016
In-patient admission	841531000006114	841531000006105	841531000006114
Admission by GP locum	851101000000115	476981000000106	851101000000115
Hospital admission note	62121000000114	24651000000105	62121000000114
Admission to emergency department	3323601000006114	50849002	2995318014
Intervention - hospital admission	971111000006119	971111000006103	971111000006119
Accident and Emergency department discharge to HDU (high dependency unit)	8439301000006111	1066361000000104	2680711000000111
Admission to adult ITU	5939351000006116	305352006	447751017
Admission by co-operative general practitioner	851091000000111	388751000000101	1692241000000117
Emergency department discharge to emergency department short stay ward	8439231000006111	1066331000000109	2678091000000111
Date Pt admitted (Oral Medicine and Surgery)	1597591000006112	1597591000006108	1597591000006112
Date Pt admitted (Ophth)	1598001000006112	1598001000006108	1598001000006112
Inpatient stay 10 days	783621000006116	183808007	284005019
Discharge Summary (Full)	1563121000006112	1563121000006108	1563121000006112
Admission notification	2304471000000113	895571000000108	2304471000000113
Discharged from acute assessment unit	2172541000000112	836721000000102	2172541000000112
ENT emergency hospital admission	4723421000006118	183462003	4723421000006118
Discharge to nursing home	449801010	306694006	449801010
Awaiting hospital death discharge letter	500831000006114	184276009	284625016
Ear, nose and throat emergency hospital admission	4723441000006113	183462003	3082934019
Reviewed at hospital	265639016	763287008	3638423011

Inpatient discharge summary received	15051000000111	16831000000102	15051000000111
Emergency hospital admission to intermediate care service	8453391000006115	1078331000000102	2703511000000118
Inpatient stay 7 days	783721000006112	183805005	284002016
Inpatient stay	782791000006115	308540004	451902013
Transfer of care from hospital	95461000006112	710112000	3043107011
Long stay hospital inpatient	250569018	160741006	250569018
Admission to high dependency unit	6598861000006115	398162007	1777720019
Admission to Inpatient Care	8101001000006119	313071000000104	566451000000110
Admission to endocrinology department	5939521000006115	305366001	447768018
Admission by doctor	2476441014	305260005	2476441014
Discharge to home	2901225012	306689006	2901225012
Hospital patient	397754013	266938001	397754013
Admission to day ward	447734012	305343002	447734012
Hospital admission, emergency, from emergency room, accidental injury	2665021000006115	10378005	18064016
Admission by associate GP	851081000000114	437171000000107	851081000000114
Admission to private hospital	5939121000006111	305339001	447728017
Place to which Pt admitted (Neuro)	1596671000006113	1596671000006109	1596671000006113
Admission to cardiac surgery department	5940121000006115	305412005	447829016
Admit oral surgical emergency	12482141000006116	183473005	283545014
Inpatient stay 11 days	783631000006118	183809004	284006018
Discharge letter given to patient	1756581000006115	1756581000006104	1756581000006115
Admit neurology emergency	12482101000006118	183467009	283538014
Admit to neurological ITU	12482261000006112	183448005	283516012
Follow-up appointment offered after hospital discharge	1882401000006115	1882401000006104	1882401000006115
ICU - Admission to intensive care unit	5939331000006111	305351004	447748012
Date Pt admitted (Urology)	1599441000006110	1599441000006106	1599441000006110
Orthopedic emergency hospital admission	4723411000006114	183461005	3082890012
Haematology emergency hospital admission	4723591000006117	183470008	4723591000006117
Admit urology emergency	12482291000006116	183468004	283539018
Emergency department discharge to HDU (high dependency unit)	8439291000006110	1066361000000104	2678151000000119
Date Pt admitted (Gastro)	1593531000006115	1593531000006104	1593531000006115
Admission by general practitioner partner	5937821000006112	305232008	2972101017
Discharge to relative's home	449797014	306690002	449797014
Cardiology emergency hospital admission	6029451000006115	313385005	6029451000006115
Review at hospital	265638012	763287008	3638423011
Discharge letter sent to general practitioner	517481000000119	294331000000109	517481000000119
Discharged from private hospital	283811011	183670004	3029377018
Seen in hospital ward	285196018	185212007	285196018
Accident and Emergency department discharge to Accident and Emergency department short stay ward	8439241000006118	1066331000000109	2680761000000113
Death notification from hospital	608291000006116	184275008	284624017

Inpatient stay 9 days	782781000006118	183807002	284004015
Emergency hospital admission to endocrinology service	8453321000006117	1078271000000105	2703391000000117
Discharge summary awaited	1676631000006113	17301000000103	21521000000115
Oral surgical emergency hospital admission	283545014	183473005	3082839011
Plastic surgery emergency hospital admission	283543019	183471007	3082940014
Neurosurgical emergency hospital admission	283548011	183476002	3082967015
Date Pt admitted (Neuro)	1596661000006118	1596661000006102	1596661000006118
Hospital admission, emergency, direct	3779761000006116	78680009	130558011
Long stay hospital	3016541000006111	32074000	1227906014
Admission to orthopaedic department	5940311000006114	305428000	447849013
Admission by dental surgeon	5938601000006119	305298005	447677019
Admission by own general practitioner	5937801000006119	305231001	2972278017
Admission to surgical department	5940081000006117	305408004	447825010
Death in hospital	2769631000006119	16983000	28745019
Admission to vascular surgery department	5940401000006119	305435008	447857011
Emergency hospital admission	283520011	183452005	283520011
Died in hospital	283820019	183676005	283820019
Seen in hospital casualty	285194015	185210004	285194015
Admission for treatment	5931041000006116	304566005	446903011
Inpatient	6897591000006113	416800000	2549330012
Hospital admission, emergency, from emergency room	3696791000006119	73607007	122228018
Admission by own GP	447588016	305231001	447588016
Admission by GP partner	447589012	305232008	447589012
Discharge to hospital	449806017	306699001	449806017
Patient transfer, in-hospital	3107571000006112	37729005	62934015
Admission to intensive care unit	5939301000006115	305351004	447749016
Discharge summary sent to general practitioner	8237241000006114	763591000000106	1693801000000113
Admit orthopaedic emergency	12482151000006119	183461005	283532010
Admitted: Hospital	1583651000006112	1583651000006108	1583651000006112
Inappropriate non-elective hospital admission	1955951000006115	1955951000006104	1955951000006115
Hospital admission, emergency, indirect	1728541000006114	18083007	30547015
Admission to acute assessment unit	2172381000000110	836651000000103	2172381000000110
Emergency hospital admission from walk-in centre	2186461000000117	842951000000109	2186461000000117
Admission to A & E department	3323591000006118	50849002	1230916011
Discharge summary report	63271000000113	25581000000101	63271000000113
Hospital admission	283592013	32485007	54238014
Admission by physician	5938171000006116	305260005	447631011
Hospital notified of death	1780508019	401321000	1780508019
A&E attendance - admitted to hospital bed/lodged patient	1981761000006114	1981761000006105	1981761000006114
Self-referral to hospital	283832015	183687002	283832015
Patient transfer, in-hospital, service-to-service	1824251000006116	57976004	1824251000006116

Discharge from Accident and Emergency service with advice for follow up treatment by general practitioner	8368771000006114	989501000000106	2514921000000119
Inpatient stay 8 days	783731000006110	183806006	284003014
Inpatient stay 14 days	783661000006110	183812001	284009013
Inpatient stay 13 days	783651000006113	183811008	284008017
Emergency room admission, died in emergency room	3719361000006111	75004002	124573013
Listed for Geriatrics admission	283967016	183774000	2668801012
Admission to urology department	5940391000006116	305434007	447856019
Admission to general surgical department	5940251000006116	305423009	447842016
A&E attendance - discharged with follow up treatment by GP	1981771000006119	1981771000006103	1981771000006119
Patient died in acute hospital	1964501000006119	1964501000006103	1964501000006119

Table S41. Intracranial haemorrhage Aurum codes

Term	Medcode ID	SNOMED CT Concept ID	SNOMED CT Description ID
Intrapontine haemorrhage	2622631000006114	7713009	503468012
Cerebral hemisphere haemorrhage	3687811000006118	73020009	502318010
Traumatic subdural hematoma without open intracranial wound	6053291000006112	315046005	459512013
Subarachnoid haemorrhage following injury	391042012	450375008	2915309017
Cortical haemorrhage	495394013	49422009	495394013
Internal capsule haemorrhage	496232015	52201006	496232015
Intracranial haemorrhage	300298011	1386000	475553012
Other cerebral haemorrhage following injury NOS	320897016	450418003	2916363013
Basal ganglia haemorrhage	503791000006114	195165005	300272017
Sequelae of intracerebral haemorrhage	300407010	195241001	300407010
Spontaneous subarachnoid haemorrhage	5518041000006112	270907008	405380011
Subarachnoid hemorrhage from carotid siphon and bifurcation	4777721000006118	195155004	300243018
Cerebellar hemorrhage	3719851000006116	75038005	124627016
Cerebral infarction with haemorrhagic transformation	1573101000006112	1573101000006108	1573101000006112
Subdural intracranial haemorrhage	3071681000006119	35486000	2913218019
Traumatic intracranial subarachnoid haemorrhage	5459271000006119	262955000	391048011
Subarachnoid intracranial haemorrhage	2841161000006110	21454007	2916299018
Subarachnoid haemorrhage following injury without open intracranial wound	2950231000006111	28048009	2950231000006111
Middle meningeal haemmorhage following injury	702641000006119	30400005	484576014
Intracerebral haemorrhage with intraventricular haemorrhage	4777861000006119	195168007	2915438018
Subdural hemorrhage	3071661000006112	35486000	59206018
Epidural hemorrhage	3850501000006119	82999001	137667016
Cerebral haemorrhage	884421000006119	274100004	884421000006119
Subdural h'ge inj no open intracran wnd+>24hr LOC -restored	124121000006111	209947002	320752018
Subdural h'ge inj no open intracranial wound+<1hr loss consc	124181000006110	209947002	320752018
Cerebral haemorrhage NOS	884451000006111	700251000000105	884451000006111
Traumatic hemorrhage into subarachnoid space of neuraxis	7377831000006117	450375008	2915309017
Closed traumatic subdural haemorrhage	320752018	209947002	320752018
Subarachnoid haemorrhage from carotid siphon and bifurcation	300244012	195155004	300244012
Haematoma of brain	5898441000006118	301764006	443223014
SDH - Subdural haematoma	4056891000006113	95453001	1217311015
Spontaneous subacute subdural hemorrhage	8089871000006114	291591000119107	2959496014
Traumatic subdural intracranial haemorrhage	4854361000006118	209987007	2912530015
Subarachnoid hemorrhage	12761901000006117	21454007	36011016
Cerebral haemorrhage NOS	989211000006119	274100004	989211000006119
Ruptured aneurysm of basilar artery	5597601000006115	277324009	413805016

Subarachnoid haemorrhage from middle cerebral artery aneurysm	5583021000006113	276280009	412353018
External capsule haemorrhage	300276019	195167002	300276019
Sequelae of intracerebral hemorrhage	4778351000006113	195241001	300408017
Subdural hemorrhage following injury	4854341000006117	209987007	320838011
Subdural h'ge inj no open intracranial wnd+>24 LOC +recovery	124171000006112	209947002	320752018
Intrapontine hemorrhage	2622641000006116	7713009	13755017
Subdural haematoma evacuation	3469891000006117	59712006	498519016
Other cerebral haemorrhage following injury	320853015	450418003	2916363013
Intracranial subarachnoid haemorrhage from vertebral artery	4777761000006112	195160000	2916525016
ICH - intracerebral hemorrhage	12223131000006114	274100004	3673218019
Intracerebral hemorrhage, multiple localized	4777891000006110	195169004	300279014
Intracerebral haemorrhage	12223101000006118	274100004	3673215016
Traumatic intracerebral hemorrhage	7378401000006111	450418003	2915288012
[X]Other intracerebral haemorrhage	11919571000006110	274100004	11919571000006110
Subarachnoid h'ge inj no open intracran wound + 1-24hr LOC	123421000006117	28048009	46957015
Subarachnoid hemorrhage from basilar artery aneurysm	5583111000006118	276284000	412362016
Ruptured aneurysm of posterior inferior cerebellar artery	5597611000006117	277325005	413807012
Thalamic haemorrhage	5011411000006113	230711001	345676013
Haemorrhagic cerebral infarction	5011291000006119	230706003	345667018
Intracranial haemorrhage following injury without open intracranial wound	4198341000006113	111668007	189842012
Pontine hemorrhage	2622651000006119	7713009	13756016
Evacuation of subdural haematoma	3469851000006111	59712006	498516011
Pontine haemorrhage	503469016	7713009	503469016
Non-traumatic subdural hemorrhage	4777931000006118	195176009	2469434017
Subdural haemorrhage	2534198011	35486000	486181011
Subarachnoid h'ge inj no open intracran wnd+>24 LOC+recovery	123401000006110	28048009	46957015
Subarach h'ge inj no open intracran wnd+LOC unspec duration	123331000006114	28048009	46957015
Subarach h'ge inj no open intracran wnd + concussion unspec	123311000006115	28048009	46957015
Subarachnoid h'ge inj no open intracran wnd+no loss consc	123411000006113	28048009	46957015
Subarachnoid haemorrhage following injury without open intracranial wound	402929011	28048009	483737017
Cerebral haemorrhage following injury	12757911000006115	82894007	505322013
Cerebral hemorrhage following injury	12757891000006117	82894007	505319011
Traumatic intracranial hemorrhage	12757901000006118	82894007	505320017
Right sided intracerebral haemorrhage, unspecified	163261000006119	195168007	300277011
[X]Other subarachnoid haemorrhage	300936018	21454007	481028017
Cerebral haemorrhage	122371000006118	274100004	409860011
Other and unspecified intracranial haemorrhage	300290016	62914000	104563015
Traumatic cerebral haemorrhage	505324014	450418003	2916058017

Cerebral hemorrhage	605471000006112	274100004	409859018
Ventricular haemorrhage	2871241000006118	23276006	481581018
Subarachnoid haemorrhage due to ruptured aneurysm	5011581000006119	230719004	345695018
Cerebral haemorrhage	300939013	274100004	409860011
Subdural haemorrhage following injury	320836010	209987007	320836010
Hemorrhage of medulla oblongata	7951281000006118	732923001	3467313018
Lobar cerebral haemorrhage	345675012	230710000	345675012
Ruptured berry aneurysm	300242011	195154000	300242011
Sequelae of subarachnoid haemorrhage	300406018	195240000	300406018
Subdural h'ge after injury	991811000006115	209987007	991811000006115
[X]Intracerebral haemorrhage in hemisphere, unspecified	300956017	274100004	409860011
Subdural h'ge inj no open intracranial wnd + unspec consc	124161000006117	209947002	320752018
Subdural hemorrhage following injury without open intracranial wound	4854171000006111	209947002	2915477015
Pituitary haemorrhage	5111331000006119	237702003	356253018
SAH - Subarachnoid hemorrhage	2841151000006113	21454007	1217630014
Non-traumatic intracranial subdural haemorrhage	4777951000006113	195176009	2913186018
History of subarachnoid haemorrhage	4540691000006112	161515009	2986875019
Subdural hemorrhage - nontraumatic	4777911000006112	195176009	300293019
Intracerebral hemorrhage with intraventricular hemorrhage	4777871000006114	195168007	2916313015
Intracranial hemorrhage following injury	7378281000006118	450410005	2915467017
Intracranial haemorrhage following injury	12716261000006116	82894007	505321018
Traumatic cranial subdural hematoma	5459251000006112	262952002	391039018
Subdural intracranial hemorrhage	3071671000006117	35486000	2912630010
Intracerebral haemorrhage NOS	300287010	274100004	2819959010
Subdural h'ge inj no open intracranial wound+no loss consc	124191000006113	209947002	320752018
Traumatic subdural hematoma	391035012	262952002	2912898019
Cerebral hemorrhage	13031191000006112	274100004	122371000006118
Subdural hematoma	4056881000006110	95453001	158107014
Ruptured cerebral arteriovenous malformation	5597281000006110	277299009	413775010
Intracerebral haemorrhage, multiple localised	4777881000006112	195169004	300280012
Closed traumatic subdural hemorrhage	4854141000006115	209947002	320753011
H/O subarachnoid hemorrhage	4540671000006111	161515009	251700011
Subdural hematoma evacuation	3469881000006115	59712006	498518012
Traumatic cerebral hemorrhage	7378441000006113	450418003	2916150010
Intraventricular haemorrhage	2871271000006114	23276006	481580017
Subarach h'ge inj no open intracran wnd+>24hrs LOC-restored	123321000006111	28048009	46957015
Traumatic subdural haemorrhage	320835014	209987007	320835014
Subdural haemorrhage - nontraumatic	300294013	195176009	300294013
Nontraumatic intracerebral haemorrhage	8089851000006116	291571000119106	3331108011
Intraventricular hemorrhage	2871261000006119	23276006	39068015

SDH - Subdural hematoma	4056901000006112	95453001	1218799016
Brain stem haemorrhage	4056931000006116	95454007	512138014
Traumatic cranial subarachnoid hemorrhage	5459281000006116	262955000	391046010
Traumatic subdural intracranial hemorrhage	4854351000006115	209987007	2912507014
Subarachnoid hemorrhage from any perinatal cause	2836991000006113	21202004	35631013
Traumatic cranial subarachnoid haemorrhage	5459301000006117	262955000	391049015
Cerebral haemorrhage	989201000006117	274100004	989201000006117
Cerebral haemorrhage following injury NOS	320852013	450418003	2916363013
Evacuation of subdural hematoma	3469861000006113	59712006	99196013
Subarachnoid haemorrhage from basilar artery aneurysm	5583101000006116	276284000	412361011
Cerebral haemorrhage following injury	505322013	450418003	2916363013
Traumatic intracerebral haemorrhage	7378421000006118	450418003	2915664016
External capsule hemorrhage	4777831000006111	195167002	300275015
Haemorrhage of medulla oblongata	7951271000006116	732923001	3467314012
Cerebral hemorrhage following injury	7378411000006114	450418003	2915447014
Intracranial hemorrhage	2520541000006112	1386000	3421016
Traumatic intracranial haemorrhage	12757921000006111	82894007	505323015
Cerebral h'ge after injury	896531000006110	82894007	896531000006110
Subarachnoid haemorrhage NOS	300257016	21454007	481028017
Bulbar haemorrhage	483988011	732923001	3467313018
Traumatic cerebral haemorrhage	12757931000006114	82894007	505324014
Sequelae of other nontraumatic intracranial haemorrhage	149551000006111	363302008	482447013
Intracerebral haemorrhage in hemisphere, unspecified	744921000006116	274100004	409860011
Thalamic hemorrhage	5011421000006117	230711001	345677016
Sequelae of subarachnoid hemorrhage	4778331000006118	195240000	300405019
Subdural haemorrhage	884441000006114	195176009	884441000006114
Cerebral hemorrhage	12762021000006116	274100004	605471000006112
Traumatic intracerebral haemorrhage	12757941000006116	82894007	2475153011
Stroke due to intracerebral haemorrhage	11903571000006110	274100004	11903571000006110
Subarachnoid hemorrhage	428181000006115	21454007	481028017
Subarachnoid haemorrhage from anterior communicating artery aneurysm	5583061000006119	276282001	412357017
Traumatic intracranial subarachnoid hemorrhage	5459291000006118	262955000	391047018
ICH - intracerebral haemorrhage	12223121000006111	274100004	3673217012
Traumatic intracranial subdural hematoma	5459241000006110	262952002	391038014
Intracerebral hemorrhage	12223111000006115	274100004	3673216015
Traumatic cranial subdural haematoma	5459221000006115	262952002	391036013
Lobar cerebral hemorrhage	5011401000006110	230710000	345674011
History of subarachnoid hemorrhage	4540681000006114	161515009	2986825016
Intracerebral haemorrhage, intraventricular	300277011	195168007	300277011
Subdural h'ge inj no open intracran wnd+LOC unspec duration	124141000006116	209947002	320752018

Intracranial subarachnoid hemorrhage from vertebral artery	4777751000006110	195160000	2915522014
Intracranial haemorrhage following injury	7378271000006116	450410005	2915426010
Subarachnoid hemorrhage from vertebral artery	4777741000006113	195160000	300254011
Subarachnoid haemorrhage from posterior communicating artery	123521000006118	21454007	36011016
Intracerebral hemorrhage (ICH)	5560161000006111	274100004	2819960017
Closed traumatic subdural intracranial hemorrhage	4854151000006118	209947002	2912529013
Subarachnoid h'ge inj no open intracran wnd+<1hr loss consc	123391000006113	28048009	46957015
Traumatic subdural haematoma without open intracranial wound	459511018	315046005	459511018
Non-traumatic intracranial subdural hemorrhage	4777941000006111	195176009	2913068019
Intracranial hemorrhage following injury	12757871000006118	82894007	137497019
Intracranial haemorrhage NOS	12729181000006117	700251000000105	1530681000000111
Evacuation of haematoma from cerebellum	265690015	171474001	265690015
Evacuation of hematoma from cerebellum	4644251000006117	171474001	265691016
Occipital subdural haematoma	7090271000006110	428561000	2694218015
Cerebellar haemorrhage	502878012	75038005	502878012
Subarachnoid hemorrhage following injury without open intracranial wound	11923791000006114	28048009	46957015
Traumatic subdural haematoma	5459261000006114	262952002	2913152010
[X]Subarachnoid haemorrhage from other intracranial arteries	300935019	21454007	36011016
Basal ganglia hemorrhage	4777811000006117	195165005	300271012
Subdural h'ge inj no open intracran wnd+1-24hr loss consc	124131000006114	209947002	320752018
Left sided intracerebral haemorrhage, unspecified	748941000006115	274100004	409859018
Subarachnoid haemorrhage from middle cerebral artery	123511000006114	21454007	36011016
Intracerebral haemorrhage, multiple localized	746571000006116	195169004	300280012
Traumatic cerebral hemorrhage	12757881000006115	82894007	137503010
Right sided intracerebral haemorrhage, unspecified	12722481000006116	308128006	451441015
Intraparenchymal haematoma of brain	7134241000006113	431266005	2768748018
Subarachnoid h'ge inj no open intracran wound + unspec consc	123431000006119	28048009	46957015
Subarachnoid haemorrhage from posterior communicating artery aneurysm	5583081000006112	276283006	412360012
Inferior cerebellar artery syndrome	3778011000006112	78569004	130376017
SAH - Subarachnoid haemorrhage	2841141000006111	21454007	1216125018
Ruptured cerebral aneurysm	5058411000006114	233983001	350578011
Internal capsule hemorrhage	3346051000006110	52201006	86879010
Anterior cerebral circulation haemorrhagic infarction	5011331000006114	230707007	345668011
Closed traumatic subdural intracranial haemorrhage	4854161000006116	209947002	2912797018
Subcortical hemorrhage	2832661000006113	20908003	35158017
Subdural haemorrhage following injury without open intracranial wound	4854181000006114	209947002	2915859018
Subdural h'ge inj no open intracran wound+concussion unspec	124151000006119	209947002	320752018
Subdural haematoma	4056871000006112	95453001	512137016

Middle meningeal hemorrhage following injury	2989681000006112	30400005	50888016
Intracerebral hemorrhage, intraventricular	4777851000006116	195168007	300278018
Subarachnoid intracranial hemorrhage	2841171000006115	21454007	2916568015
Subarachnoid haemorrhage	481028017	21454007	481028017
Non-traumatic subdural haemorrhage	2475119012	195176009	2469010019
Traumatic intracranial hemorrhage	7378301000006119	450410005	2915928019
H/O subarachnoid haemorrhage	808111000006110	161515009	251701010
Subarachnoid haemorrhage from basilar artery	123491000006115	276284000	412361011
Traumatic subdural hemorrhage	4854331000006110	209987007	320837018
Subarachnoid haemorrhage from anterior communicating artery	123481000006118	21454007	36011016
Subarachnoid haemorrh from intracranial artery, unspecif	123441000006112	21454007	481028017
Intracerebral haemorrhage (ICH)	744901000006114	274100004	2819959010
Traumatic intracerebral hemorrhage	12757951000006119	82894007	2475363012
Subarachnoid haemorrhage from vertebral artery	300253017	195160000	300253017
Ruptured aneurysm of middle cerebral artery	5597491000006112	277316004	413794017

Table S42. Intracranial haemorrhage HES (ICD10) codes

ICD10 codes	Term
I60.0	Subarachnoid haemorrhage from carotid siphon and bifurcation
I60.1	Subarachnoid haemorrhage from middle cerebral artery
I60.2	Subarachnoid haemorrhage from anterior communicating artery
I60.3	Subarachnoid haemorrhage from posterior communicating artery
I60.4	Subarachnoid haemorrhage from basilar artery
I60.5	Subarachnoid haemorrhage from vertebral artery
I60.6	Subarachnoid haemorrhage from other intracranial arteries
I60.7	Subarachnoid haemorrhage from intracranial artery, unspecified
I60.8	Other subarachnoid haemorrhage
I60.9	Subarachnoid haemorrhage, unspecified
I61.0	Intracerebral haemorrhage in hemisphere, subcortical
I61.1	Intracerebral haemorrhage in hemisphere, cortical
I61.2	Intracerebral haemorrhage in hemisphere, unspecified
I61.3	Intracerebral haemorrhage in brain stem
I61.4	Intracerebral haemorrhage in cerebellum
I61.5	Intracerebral haemorrhage, intraventricular
I61.6	Intracerebral haemorrhage, multiple localized
I61.8	Other intracerebral haemorrhage
I61.9	Intracerebral haemorrhage, unspecified
I62.0	Subdural haemorrhage (acute)(nontraumatic)
I62.1	Nontraumatic extradural haemorrhage
I62.9	Intracranial haemorrhage (nontraumatic), unspecified
S06.4	Epidural haemorrhage
S06.5	Traumatic subdural haemorrhage
S06.6	Traumatic subarachnoid haemorrhage
I69.0	Sequelae of subarachnoid haemorrhage
I69.1	Sequelae of intracerebral haemorrhage
I69.2	Sequelae of other nontraumatic intracranial haemorrhage

Table S43. Heart failure outcome Aurum codes

Term	Medcode ID	SNOMED CT Concept ID	SNOMED CT Description ID
Acute left ventricular failure	300190010	195114002	300190010
Impaired left ventricular function	411506018	275514001	411506018
O/E - pulmonary oedema	253994013	162970000	253994013
New York Heart Association Classification - Class II	2616471011	421704003	2616471011
Congestive heart failure due to valvular disease	2675255018	426611007	2675255018
Left ventricular cardiac dysfunction	2694523019	429589006	2694523019
O/E - pulmonary edema	4555461000006110	162970000	253993019
CCF - Congestive cardiac failure	3182551000006115	42343007	493288018
Chronic pulmonary oedema	494669012	46847001	494669012
Pulmonary congestion and hypostasis NOS	301695010	196115007	301689014
Admit heart failure emergency	12490061000006118	416683003	2549208013
Acute congestive heart failure	18472010	10633002	18472010
COCM - Congestive cardiomyopathy	6615881000006112	399020009	1786620011
Emergency hospital admission for heart failure	6895651000006113	416683003	6895651000006113
Heart failure as a complication of care	350484012	233924009	350484012
Hypertensive heart disease NOS with CCF	741681000006111	64715009	107545013
Congestive heart failure	70653017	42343007	70653017
Other transplantation of heart	12719481000006116	586351000000102	1300781000000114
Left ventricular diastolic dysfunction	1489358014	395704004	1489358014
Congestive cardiomyopathy	1778488011	399020009	1778488011
Cardiac failure	139482012	84114007	139482012
Congestive heart disease	3182531000006110	42343007	70654011
Right ventricular failure	1816101000006113	367363000	490972013
Postoperative pulmonary oedema	401870011	11468004	401870011
Right heart failure	206703015	128404006	206703015
Heart failure with preserved ejection fraction	7321121000006119	446221000	3496968011
Congestive cardiac failure	3182541000006117	42343007	493287011
CCM - Congestive cardiomyopathy	6615871000006114	399020009	1786619017
Heart failure with preserved ejection fraction	2227501000000110	446221000	2227501000000110
Acute pulmonary oedema unspecified	301741013	40541001	492666016
Left ventricular failure	141306010	85232009	141306010
Chronic congestive heart failure	147247018	88805009	147247018
Nonischemic congestive cardiomyopathy	7964821000006113	111000119104	2920819015
Acute cardiac pulmonary oedema	6212521000006115	360371003	476096017
Heart failure NOS	395772015	84114007	139475013
HF - Heart failure	3868341000006118	84114007	1234906013
Heart failure with reduced ejection fraction due to heart valve disease	7573211000006119	703276005	3008139015
History of heart failure in last year	5990971000006113	309634009	2986867013
Cause of Death- Congestive Cardiac Failure	1576321000006113	1576321000006109	1576321000006113

Chronic heart failure	3283871000006117	48447003	80720010
Pulmonary edema - acute	3152731000006113	40541001	1490485013
New York Heart Association Classification - Class IV	2616473014	422293003	2616473014
Pulmonary oedema NOS	301694014	19242006	479262018
[D]Cardiorespiratory failure	317955011	410431009	2472092014
Chronic left ventricular systolic dysfunction	7119761000006112	430396006	2770949016
Impaired left ventricular func	5573991000006118	275514001	1495323018
Biventricular failure	4005301000006110	92506005	510016018
Hypertensive heart&renal dis wth (congestive) heart failure	741701000006114	194779001	299672017
Heart failure due to end stage congenital heart disease	7475721000006114	471880001	2950981012
Acute edema of lung	3152701000006117	40541001	67601010
Cardiac cirrhosis	3713561000006119	74669004	123997016
Left-sided heart failure	3886061000006119	85232009	201199018
On examination - pulmonary edema	4555471000006115	162970000	2667198010
Heart failure confirmed	1488804017	395105005	1488804017
Hyperten heart&renal dis+both(congestv)heart and renal fail	789941000006117	194781004	299674016
Acute pulmonary edema	3152691000006117	40541001	67598017
Systolic heart failure	6914191000006115	417996009	2577903013
Decompensated cardiac failure	300179017	195111005	300179017
Compensated cardiac failure	300180019	195112003	300180019
HFNEF - heart failure with normal ejection fraction	1661371000000112	446221000	1713091000000115
Acute oedema of lung, unspecified	1216090015	40541001	67598017
Severe left ventricular systolic dysfunction	1991651000006115	1991651000006104	1991651000006115
LVF - Left ventricular failure	3886071000006114	85232009	1235017018
Left heart failure	3886041000006118	85232009	141303019
Right heart failure due to pulmonary hypertension	7052811000006113	426012001	2674125010
Cardiac failure NOS	223981000000118	84114007	139482012
Diastolic heart failure	6919191000006119	418304008	2577902015
[RFC] Cardiac failure	905391000006119	905391000006103	905391000006119
Acute oedema of lung	3152711000006119	40541001	492667013
Congestive cardiac failure	493287011	42343007	70653017
Acute heart failure	94251011	56675007	94251011
Pulmonary congestion	112265015	67599009	112265015
Post cardiac operation heart failure NOS	300217019	195130005	300214014
CHF - Congestive heart failure	3182561000006118	42343007	493289014
Chronic pulmonary edema	3257341000006116	46847001	78084015
Heart failure	139475013	84114007	139475013
Right ventricular failure	490972013	367363000	490972013
Fluid overload pulmonary oedema	5054791000006119	233712009	350205014
Fluid overload pulmonary oedema	5054791000006119	233712009	350205014
Heart failure with normal ejection fraction	1647701000000118	446221000	2883808011

Postoperative pulmonary edema	2682661000006114	11468004	19840013
New York Heart Association Classification - Class III	2616472016	420913000	2616472016
Acute pulmonary oedema NOS	301743011	40541001	492666016
Echocardiogram shows left ventricular systolic dysfunction	2159197017	407596008	2159197017
Echocardiogram shows left ventricular diastolic dysfunction	2159198010	407597004	2159198010
Pulmonary oedema - acute	3152721000006110	40541001	1490256017
Pulmonary oedema - acute	1490256017	40541001	492666016
Suspected heart failure	1488591011	394887005	1488591011
Malignant hypertensive heart disease with CCF	728671000006119	83105008	1236017010
Exacerbation of congestive heart failure	8030311000006111	96311000119109	2921293019
H/O: Heart failure in last year	453099015	309634009	453099015
Acute right-sided heart failure	6204381000006117	359617009	474047012
Acute right-sided heart failure	6204381000006117	359617009	474047012
Biventricular failure	510016018	92506005	153058012
Heart failure with reduced ejection fraction	7573171000006116	703272007	3008211011
Impaired left ventricular function	784191000006110	275514001	411506018
Decompensated chronic heart failure	7025691000006110	424404003	2645367010
Left ventricular systolic dysfunction	216207010	134401001	216207010
Pulmonary congestion and hypostasis	301689014	196115007	301689014
Congestive heart failure with right heart failure	8011111000006111	23341000119109	2984250012

Table S44. Heart failure HES (ICD10) codes

ICD10 codes	Term
I50.0	Congestive heart failure
I50.1	Left ventricular heart failure
I50.9	Heart failure, unspecified
I11.0	Hypertensive heart disease with (congestive) heart failure
I13.0	Hypertensive heart and renal disease with (congestive) heart failure
I13.2	Hypertensive heart and renal disease with both (congestive) heart failure and renal failure
I42.0	Dilated cardiomyopathy
K76.1	Chronic passive congestion of liver
J81	Pulmonary oedema
R57.0	Cardiogenic shock

Chapter 3. Code lists for baseline characteristics

Table S45. Ethinicity Aurum codes

Term	Medcode ID	SNOMED CT Concept ID	SNOMED CT Description ID
Black Arab	411574019	275587000	411574019
Black Iranian	411575018	275588005	411575018
Black East African Asian	411576017	275589002	411576017
Caribbean Island (NMO)	411578016	275591005	411578016
Iranian (NMO)	411582019	275595001	411582019
Turkish Cypriot (NMO)	411597016	275602002	411597016
O/E - Asian origin	412016016	276029002	412016016
Australian origin	250227018	160518001	250227018
Middle Eastern origin	250229015	160520003	250229015
Far Eastern origin	250230013	160521004	250230013
West Indian origin	250231012	160522006	250231012
O/E - Europeanoid	253629014	162731001	253629014
Other ethnic NEC (NMO)	285988010	186005001	285958018
New Zealand ethnic group NOS	286022019	186035008	286006015
Afghan	1564151000006117	1564151000006101	1564151000006117
Australian	1564241000006111	1564241000006107	1564241000006111
Bangladeshi	1564291000006119	1564291000006103	1564291000006119
Burmese	1564431000006119	1564431000006103	1564431000006119
Chilean	1564511000006114	1564511000006105	1564511000006114
Cuban	1564581000006119	1564581000006103	1564581000006119
Filipino	1564731000006113	1564731000006109	1564731000006113
Greek	1564811000006111	1564811000006107	1564811000006111
Israeli	1564971000006113	1564971000006109	1564971000006113
Italian	1564981000006111	1564981000006107	1564981000006111
Korean (North)	1565071000006113	1565071000006109	1565071000006113
Malaysian	1565211000006111	1565211000006107	1565211000006111
Moroccan	1565321000006110	1565321000006106	1565321000006110
New Zealander	1565391000006112	1565391000006108	1565391000006112
Saudi Arabian	1565611000006114	1565611000006105	1565611000006114
Somali	1565701000006116	1565701000006100	1565701000006116
Thai	1565821000006119	1565821000006103	1565821000006119
Venezuelan	1565941000006116	1565941000006100	1565941000006116
Vietnamese	1565951000006119	1565951000006103	1565951000006119
On examination - Asian origin	5580031000006118	276029002	2669882010
Ethnic group not recorded	456652017	312861003	456652017
Other ethnic, mixed white origin	4740381000006118	186022004	2668812012
Mixed multiple ethnic groups: White and Black Caribbean - England and Wales ethnic category 2011 census	1968091000006110	976711000000103	2484791000000114

Asian or Asian British: Bangladeshi - England and Wales ethnic category 2011 census	1968151000006117	976831000000100	2485031000000111
Black or African or Caribbean or Black British: other Black or African or Caribbean background - England and Wales ethnic category 2011 census	1968201000006114	976931000000109	2485231000000119
Asian or Asian British: Pakistani - Northern Ireland ethnic category 2011 census	1968311000006115	977711000000100	2486881000000113
Asian or Asian British: Chinese - Northern Ireland ethnic category 2011 census	1968331000006114	977751000000101	2486961000000110
Asian or Asian Scottish or Asian British: Chinese - Scotland ethnic category 2011 census	1968511000006114	978191000000109	2487851000000117
Caribbean or Black: any other Black or Caribbean group - Scotland ethnic category 2011 census	1968571000006117	978361000000101	2488221000000112
British ethnic minority unspecified (NMO)	4740261000006118	186007009	2668698012
On examination - Mongoloid origin	5507871000006110	268914005	2669720011
Black Caribbean	514611000006111	185988007	285930014
Chinese	550541000006110	33897005	56590016
White British	459726019	315236000	459726019
White Irish	459727011	315237009	459727011
Other Asian ethnic group	459784018	315281001	459784018
Irish traveller	459786016	315283003	459786016
White: Irish - England and Wales ethnic category 2011 census	2484671000000118	976651000000108	2484671000000118
White: Scottish - Scotland ethnic category 2011 census	2487281000000112	977911000000103	2487281000000112
White: Polish - Scotland ethnic category 2011 census	2487481000000113	978011000000101	2487481000000113
Sri Lankan - ethnic category 2001 census	136081000000111	86461000000107	136081000000111
Albanian - ethnic category 2001 census	138231000000116	88971000000106	138231000000116
Jewish - ethnic category 2001 census	138241000000113	88991000000105	138241000000113
White and Black African - ethnic category 2001 census	141331000000116	92431000000100	141331000000116
Other Asian background - ethnic category 2001 census	141381000000117	92481000000101	141381000000117
Welsh - ethnic category 2001 census	141441000000119	92551000000106	141441000000119
Northern Irish - ethnic category 2001 census	141451000000116	92561000000109	141451000000116
Chinese and White - ethnic category 2001 census	141491000000112	92601000000109	141491000000112
Kashmiri - ethnic category 2001 census	141531000000112	92651000000105	141531000000112
East African Asian - ethnic category 2001 census	141541000000115	92661000000108	141541000000115
British Asian - ethnic category 2001 census	141561000000119	92681000000104	141561000000119
Caribbean Asian - ethnic category 2001 census	141571000000114	92691000000102	141571000000114
Nigerian - ethnic category 2001 census	141601000000119	92731000000108	141601000000119
Filipino - ethnic category 2001 census	141641000000116	92771000000105	141641000000116
Cypriot (part not stated) - ethnic category 2001 census	141661000000115	92791000000109	141661000000115
Greek - ethnic category 2001 census	142701000000116	93931000000104	142701000000116
Kosovan - ethnic category 2001 census	142741000000118	93981000000100	142741000000118
Croatian - ethnic category 2001 census	142761000000117	94001000000108	142761000000117
Mixed Irish and other White - ethnic category 2001 census	142781000000114	94021000000104	142781000000114
North African - ethnic category 2001 census	142811000000112	94061000000107	142811000000112

Buddhist - ethnic category 2001 census	142881000000117	94131000000103	142881000000117
Caribbean - ethnic category 2001 census	154401000000118	107691000000105	154401000000118
English - ethnic category 2001 census	157281000000117	110761000000106	157281000000117
Sinhalese - ethnic category 2001 census	157301000000116	110781000000102	157301000000116
Serbian - ethnic category 2001 census	157991000000110	88981000000108	157991000000110
Asian and Chinese - ethnic category 2001 census	158361000000116	92611000000106	158361000000116
Mixed Black - ethnic category 2001 census	158371000000111	92721000000106	158371000000111
Italian - ethnic category 2001 census	158481000000115	93961000000109	158481000000115
British ethnic minority specified (NMO)	4740241000006117	186006000	2668697019
Tongan	504723011	81560001	504723011
White Scottish	1780407014	401213008	1780407014
Other white British ethnic group	1780408016	401214002	1780408016
Caucasian	6846371000006111	413773004	2533842018
Romanian	1158211000000111	445343003	2872780011
Black Caribbean	285930014	185988007	285930014
Black, other, non-mixed origin	285931013	185989004	285931013
Black Indian sub-continent	285948017	185995003	285948017
Black - other Asian	285949013	185996002	285949013
Other Black - Black/White orig	285952017	185999009	285952017
Other African countries (NMO)	285971015	186010002	285971015
Indian sub-continent (NMO)	285976013	186012005	285976013
Other European (NMO)	285987017	186017004	285987017
Other ethnic, Black/White orig	285990011	186020007	285990011
Other ethnic, Asian/White orig	285991010	186021006	285991010
Other ethnic, other mixed orig	285993013	186023009	285993013
New Zealand ethnic groups	286006015	186035008	286006015
Other European in New Zealand	286009010	186037000	286009010
New Zealand Maori	286012013	186039002	286012013
Tokelauan	286015010	186042008	286015010
Turkish/Turkish Cypriot (NMO)	405071018	270467002	405071018
Race: White	2537217015	413773004	2537217015
Black Caribbean/West India/Guyana	5516681000006114	270460000	2983029017
Commonwealth of (Russian) Independent States - ethnic category 2001 census	937311000006113	88961000000104	138221000000118
Other republics which made up the former Yugoslavia - ethnic category 2001 census	937371000006116	94011000000105	142771000000112
Middle Eastern (excluding Israeli, Iranian and Arab) - ethnic category 2001 census	937871000006114	94071000000100	142821000000118
Multi-ethnic islands: Mauritian or Seychellois or Maldivian or St Helena - ethnic category 2001 census	937941000006111	94121000000100	142871000000119
Black N African/Arab/Iranian	405065012	270461001	405065012
Indo-Caribbean (NMO)	411584018	275597009	411584018
Greek (NMO)	411594011	275599007	411594011
Black West Indian	453109012	309643000	453109012

Other black ethnic group	459782019	315279003	459782019
Black African and White	460154012	315635008	460154012
White: English or Welsh or Scottish or Northern Irish or British - England and Wales ethnic category 2011 census	1968051000006116	976631000000101	2484631000000115
Mixed multiple ethnic groups: any other Mixed or multiple ethnic background - England and Wales ethnic category 2011 census	1968121000006114	976771000000108	2484911000000115
Asian or Asian British: Indian - England and Wales ethnic category 2011 census	1968131000006112	976791000000107	2484951000000116
Asian or Asian British: Pakistani - England and Wales ethnic category 2011 census	1968141000006119	976811000000108	2484991000000112
Black or African or Caribbean or Black British: African - England and Wales ethnic category 2011 census	1968181000006113	976891000000104	2485151000000115
Black or African or Caribbean or Black British: Caribbean - England and Wales ethnic category 2011 census	1968191000006111	976911000000101	2485191000000111
Other ethnic group: Arab - England and Wales ethnic category 2011 census	1968211000006112	976951000000102	2485271000000117
Asian or Asian British: Indian - Northern Ireland ethnic category 2011 census	1968301000006118	977591000000103	2486641000000116
Asian or Asian British: Bangladeshi - Northern Ireland ethnic category 2011 census	1968321000006111	977731000000108	2486921000000119
Asian or Asian British: any other Asian background - Northern Ireland ethnic category 2011 census	1968341000006116	977771000000105	2487001000000110
Black or African or Caribbean or Black British: African - Northern Ireland ethnic category 2011 census	1968351000006119	977791000000109	2487041000000113
Black or African or Caribbean or Black British: other Black or African or Caribbean background - Northern Ireland ethnic category 2011 census	1968371000006112	977831000000102	2487121000000119
Other ethnic group: any other ethnic group - Northern Ireland ethnic category 2011 census	1968391000006113	977871000000100	2487201000000119
Asian or Asian Scottish or Asian British: Pakistani, Pakistani Scottish or Pakistani British - Scotland ethnic category 2011 census	1968481000006118	978071000000106	2487601000000117
Asian or Asian Scottish or Asian British: any other Asian group - Scotland ethnic category 2011 census	1968521000006118	978211000000108	2487891000000113
African: any other African - Scotland ethnic category 2011 census	1968541000006113	978251000000107	2487971000000118
Other ethnic group: Arab, Arab Scottish or Arab British - Scotland ethnic category 2011 census	1968581000006119	978381000000105	2488261000000116
Other ethnic group: any other ethnic group - Scotland ethnic category 2011 census	1968591000006116	978401000000105	2488301000000114
Black - ethnic group	459730016	315240009	459730016
Mixed ethnic census group	459729014	315239007	459729014
Asian - ethnic group	286020010	315280000	459783012
Race	371005013	103579009	265558016
White - ethnic group	459728018	185984009	285926011
Chinese	1564521000006118	1564521000006102	1564521000006118
Colombian	1564531000006115	1564531000006104	1564531000006115
Cypriot	1564591000006116	1564591000006100	1564591000006116
Peruvian	1565491000006119	1565491000006103	1565491000006119
Taiwanese	1565791000006111	1565791000006107	1565791000006111
Brazilian	1573191000006117	1573191000006101	1573191000006117

Indian	781081000006113	414481008	2533844017
Yemeni	523591000000116	296841000000102	523591000000116
Roma ethnic group	2645811000000115	718958002	2678971000000113
White Irish - ethnic category 2001 census	1064041000000111	494161000000100	1064041000000111
Polish - ethnic category 2001 census	138201000000110	88941000000100	138201000000110
Irish - ethnic category 2001 census	141301000000110	92401000000106	141301000000110
White and Black Caribbean - ethnic category 2001 census	141321000000118	92421000000102	141321000000118
Scottish - ethnic category 2001 census	141431000000111	92541000000108	141431000000111
Tamil - ethnic category 2001 census	141551000000117	92671000000101	141551000000117
Somali - ethnic category 2001 census	141591000000113	92711000000100	141591000000113
Ulster Scots - ethnic category 2001 census	142691000000116	93921000000101	142691000000116
Kurdish - ethnic category 2001 census	142841000000113	94091000000101	142841000000113
Sikh - ethnic category 2001 census	142891000000115	94141000000107	142891000000115
British or mixed British - ethnic category 2001 census	158341000000117	92391000000108	158341000000117
Ethnic group	459785017	372148003	1210724015
Black British	285932018	185990008	285932018
Indian	285954016	414481008	2533844017
Pakistani	285955015	186002003	285955015
Brit. ethnic minor. spec.(NMO)	285959014	186006000	285959014
Other Asian (NMO)	285977016	186013000	285977016
Other ethnic, mixed white orig	285992015	186022004	285992015
Pakeha	286008019	186036009	286008019
South East Asian	286018012	186044009	286018012
Arabs	196621000006115	90027003	149237014
Caucasian race	196641000006110	413773004	2537215011
Mixed racial group	196681000006116	414752008	2533847012
Oriental	196701000006118	414978006	2533849010
Unknown racial group	196731000006114	415794004	2533839012
Black East African Asian/Indo-Caribbean	514651000006112	270462008	405066013
Caribbean I./W.I./Guyana (NMO)	405067016	270463003	405067016
Black African	30683015	18167009	30683015
RACE: Bangladeshi	196631000006117	186003008	285956019
White British - ethnic category 2001 census	1063981000000117	494131000000105	1063981000000117
Black - other African country	285943014	185993005	285943014
Other ethnic, mixed origin	285989019	186019001	285989019
Cook Island Maori	286013015	186040000	286013015
Niuean	286014014	186041001	286014014
Afro-Caribbean	196601000006113	413465009	2533840014
Koreans	196671000006119	38361009	63432018
Other New Zealand ethnic group	286021014	186035008	286006015
Patient ethnicity unknown	308131000000114	202171000000101	308131000000114

Gypsy	3146431000006116	40182006	2162400016
Bangladeshi or British Bangladeshi - ethnic category 2001 census	937541000006115	92471000000103	141371000000119
Mixed multiple ethnic groups: White and Black African - Northern Ireland ethnic category 2011 census	1968271000006115	977411000000108	2486281000000114
Mixed multiple ethnic groups: any other Mixed or multiple ethnic background - Northern Ireland ethnic category 2011 census	1968291000006119	977551000000106	2486561000000119
White: any other White ethnic group - Scotland ethnic category 2011 census	1968461000006111	978031000000109	2487521000000113
South American origin	250226010	160517006	250226010
Japanese - ethnic category 2001 census	141631000000113	92761000000103	141631000000113
Bosnian - ethnic category 2001 census	142751000000115	93991000000103	142751000000115
O/E - Mongoloid origin	402434017	268914005	402434017
N African Arab/Iranian (NMO)	405068014	270464009	405068014
Guyana (NMO)	411580010	275593008	411580010
Greek Cypriot (NMO)	411595012	275600005	411595012
Black Guyana	453110019	309644006	453110019
Black Indo-Caribbean	411577014	275590006	411577014
West Indian (NMO)	411579012	275592003	411579012
Black Caribbean and White	460153018	315634007	460153018
Black	459731017	315240009	459731017
White	285925010	185984009	285925010
Bangladeshi	285956019	186003008	285956019
Irish (NMO)	285978014	186014006	285978014
Portuguese	133078012	80208004	133078012
Pakistani	1565441000006111	1565441000006107	1565441000006111
Black Black - other	285950013	185989004	285931013
European origin	250222012	160513005	250222012
Asian origin	250224013	160515003	250224013
White - Northern Ireland ethnic category 2011 census	2486161000000112	977351000000100	2486161000000112
White: other British - Scotland ethnic category 2011 census	2487321000000116	977931000000106	2487321000000116
White: Irish - Scotland ethnic category 2011 census	2487361000000112	977951000000104	2487361000000112
Other Pacific ethnic group	286017019	372148003	1210724015
Nepalese	1745831000006112	1745831000006108	1745831000006112
Caucasoid race	6846391000006112	413773004	2537216012
Japanese	196661000006114	414551003	2533843011
Race not stated	196691000006118	415226007	2533846015
Other ethnic, Black/White origin	4740341000006112	186020007	2763938013
Gypsy/Romany - ethnic category 2001 census	138191000000113	88931000000109	138191000000113
Pakistani or British Pakistani - ethnic category 2001 census	141361000000114	92461000000105	141361000000114
Black and Asian - ethnic category 2001 census	141471000000113	92581000000100	141471000000113
Black and Chinese - ethnic category 2001 census	141481000000110	92591000000103	141481000000110

Mixed Asian - ethnic category 2001 census	141511000000116	92631000000103	141511000000116
Vietnamese - ethnic category 2001 census	141621000000111	92751000000101	141621000000111
Turkish Cypriot - ethnic category 2001 census	142721000000113	93951000000106	142721000000113
Turkish - ethnic category 2001 census	156921000000110	110401000000103	156921000000110
Hindu - ethnic category 2001 census	157351000000115	110831000000107	157351000000115
Slovak	1551471000000116	36329002	3314524013
Other White European or European unspecified or Mixed European - ethnic category 2001 census	937391000006115	94041000000106	142801000000110
Other Black or Black unspecified - ethnic category 2001 census	937731000006115	92741000000104	141611000000117
Fijian	501416013	69865008	501416013
Afro-Caucasian	196611000006111	413466005	2533841013
Gypsies	850671000006119	40182006	63802019
Baltic States (Estonian or Latvian or Lithuanian) - ethnic category 2001 census	937301000006110	88951000000102	138211000000112
Other Mixed or Mixed unspecified - ethnic category 2001 census	937511000006119	92621000000100	141501000000118
Other Asian or Asian unspecified - ethnic category 2001 census	937651000006117	92701000000102	141581000000111
Asian or Asian British: Chinese - England and Wales ethnic category 2011 census	1968161000006115	976851000000107	2485071000000113
Mixed multiple ethnic groups: White and Black Caribbean - Northern Ireland ethnic category 2011 census	1968261000006110	977391000000108	2486241000000118
Mixed multiple ethnic groups: White and Asian - Northern Ireland ethnic category 2011 census	1968281000006117	977431000000100	2486321000000118
Asian or Asian Scottish or Asian British: Indian, Indian Scottish or Indian British - Scotland ethnic category 2011 census	1968491000006115	978111000000100	2487681000000110
Other ethnic non-mixed (NMO)	285958018	186005001	285958018
Brit. ethnic minor. unsp (NMO)	285960016	186007009	285960016
New Zealand European	286007012	186036009	286007012
Czech	1160331000000119	286009	3314518019
Bulgarian	1158301000000115	29343004	1158301000000115
Irish Traveller - ethnic category 2001 census	138171000000114	88911000000101	138171000000114
Traveller - ethnic category 2001 census	138181000000111	88921000000107	138181000000111
South and Central American - ethnic category 2001 census	138271000000119	89021000000101	138271000000119
Muslim - ethnic category 2001 census	138281000000117	89031000000104	138281000000117
Other White background - ethnic category 2001 census	141311000000112	92411000000108	141311000000112
Other Mixed background - ethnic category 2001 census	141351000000111	92451000000107	141351000000111
African - ethnic category 2001 census	141391000000115	92491000000104	141391000000115
Chinese - ethnic category 2001 census	141401000000117	92511000000107	141401000000117
Other - ethnic category 2001 census	141411000000115	92521000000101	141411000000115
Cornish - ethnic category 2001 census	141461000000118	92571000000102	141461000000118
Punjabi - ethnic category 2001 census	141521000000110	92641000000107	141521000000110
Malaysian - ethnic category 2001 census	141651000000118	92781000000107	141651000000118
Greek Cypriot - ethnic category 2001 census	142711000000119	93941000000108	142711000000119
Latin American - ethnic category 2001 census	142861000000114	94111000000106	142861000000114

Black and White - ethnic category 2001 census	157291000000115	110771000000104	157291000000115
Other Black background - ethnic category 2001 census	158351000000119	92501000000105	158351000000119
White: any other White background - England and Wales ethnic category 2011 census	1968081000006112	976691000000100	2484751000000118
Mixed multiple ethnic groups: White and Black African - England and Wales ethnic category 2011 census	1968101000006116	976731000000106	2484831000000119
Asian or Asian British: any other Asian background - England and Wales ethnic category 2011 census	1968171000006110	976871000000103	2485111000000119
Other ethnic group: Arab - Northern Ireland ethnic category 2011 census	1968381000006110	977851000000109	2487161000000110
Mixed or multiple ethnic groups: any Mixed or multiple ethnic group - Scotland ethnic category 2011 census	1968471000006116	978051000000102	2487561000000117
Albanian	1564161000006115	1564161000006104	1564161000006115
Brazilian	1564381000006110	1564381000006106	1564381000006110
Indian	1564921000006112	1564921000006108	1564921000006112
Iranian	1564941000006117	1564941000006101	1564941000006117
Mexican	1565281000006116	1565281000006100	1565281000006116
E Afric Asian/Indo-Carib (NMO)	405069018	270465005	405069018
Greek/Greek Cypriot (NMO)	405070017	270466006	405070017
North African Arab (NMO)	411581014	275594002	411581014
East African Asian (NMO)	411583012	275596000	411583012
Turkish (NMO)	411596013	275601009	411596013
American	1564181000006113	1564181000006109	1564181000006113
Cambodian	1564451000006114	1564451000006105	1564451000006114
Central African	1564491000006115	1564491000006104	1564491000006115
Japanese	1565011000006116	1565011000006100	1565011000006116
Sri Lankan	1565731000006112	1565731000006108	1565731000006112
Yugoslavian	1565971000006112	1565971000006108	1565971000006112
Nepali	1572831000000110	718131000000106	1572831000000110
Other White or White unspecified - ethnic category 2001 census	937411000006115	94051000000109	158491000000118
White: Gypsy or Irish Traveller - England and Wales ethnic category 2011 census	1968071000006114	976671000000104	2484711000000117
Mixed multiple ethnic groups: White and Asian - England and Wales ethnic category 2011 census	1968111000006118	976751000000104	2484871000000117
Other ethnic group: any other ethnic group - England and Wales ethnic category 2011 census	1968221000006116	976971000000106	2485311000000117
Irish Traveller - Northern Ireland ethnic category 2011 census	1968251000006113	977371000000109	2486201000000116
Black or African or Caribbean or Black British: Caribbean - Northern Ireland ethnic category 2011 census	1968361000006117	977811000000105	2487081000000117
White: Gypsy or Irish Traveller - Scotland ethnic category 2011 census	1968441000006112	977971000000108	2487401000000115
Asian or Asian Scottish or Asian British: Bangladeshi, Bangladeshi Scottish or Bangladeshi British - Scotland ethnic category 2011 census	1968501000006111	978171000000105	2487801000000118
African: African, African Scottish or African British - Scotland ethnic category 2011 census	1968531000006115	978231000000100	2487931000000115

Caribbean or Black: Caribbean, Caribbean Scottish or Caribbean British - Scotland ethnic category 2011 census	1968551000006110	978271000000103	2488011000000113
Caribbean or Black: Black, Black Scottish or Black British - Scotland ethnic category 2011 census	1968561000006112	978341000000102	2488181000000119
Other ethnic, other mixed origin	4740401000006118	186023009	2668813019
Samoan	507015012	86275006	507015012
Vietnamese	456650013	312859007	456650013
African origin	250223019	160514004	250223019
North American origin	250225014	160516002	250225014
Indian origin	250228011	160519009	250228011
Race: West indian	250243013	160531006	250243013
Refusal by patient to provide information about ethnic group	456651012	763726001	3643639011
Arab - ethnic category 2001 census	138251000000111	89001000000105	138251000000111
Iranian - ethnic category 2001 census	138261000000114	89011000000107	138261000000114
White and Asian - ethnic category 2001 census	141341000000113	92441000000109	141341000000113
Ethnic category not stated - 2001 census	141421000000114	92531000000104	141421000000114
Other mixed White - ethnic category 2001 census	142791000000111	94031000000102	142791000000111
Israeli - ethnic category 2001 census	142831000000116	94081000000103	142831000000116
Moroccan - ethnic category 2001 census	142851000000111	94101000000109	142851000000111
Any other group - ethnic category 2001 census	142901000000119	94151000000105	142901000000119
Indian or British Indian - ethnic category 2001 census	157271000000119	110751000000108	157271000000119
Black British - ethnic category 2001 census	157311000000119	110791000000100	157311000000119
RACE: Chinese	196651000006112	33897005	56590016
RACE: Pakistani	196721000006111	186002003	285955015
Irish	1564961000006118	1564961000006102	1564961000006118
Korean (South)	1565081000006111	1565081000006107	1565081000006111
Micronesian	1565291000006118	1565291000006102	1565291000006118
Nepalese	1565371000006111	1565371000006107	1565371000006111
Black Caribbean/W.I./Guyana	405064011	270460000	405064011
Black North African	411573013	275586009	411573013
Other ethnic, Asian/White origin	4740361000006111	186021006	2764123018
Chinese	56590016	33897005	56590016
Patient declined to provide information about ethnic group	12009371000006110	763726001	3644134011
Black - other, mixed	285951012	185998001	285951012
Other Black - Black/Asian orig	285953010	186000006	285953010

Table S46. Smoking Aurum codes

Term	Medcode ID	SNOMED CT Concept ID	SNOMED CT Description ID
**Current smoker**			
user of electronic cigarette	1879431000006110	1879431000006106	1879431000006110
tobacco abuse	3959141000006110	89765005	148823015
tobacco use, continuous	4765711000006110	191887008	2951660019
ready to stop smoking	1488577017	394872000	1488577017
tobacco dependence	102951000006115	89765005	2951656017
grade c moderate smoker (11-20/day)	854981000006117	854981000006101	854981000006117
smoking cessation advice	338608011	225323000	338608011
grade d heavy smoker (>20 day)	855001000006114	855001000006105	855001000006114
referred for copd structured smoking assessment	1704551000006110	375851000000108	739301000000115
current smoker	503483019	77176002	503483019
smoking free weeks	1488873010	395177003	1488873010
light smoker - 1-9 cigs/day	743331000006116	160603005	250358015
smokes drugs in cigarette form	342444018	228378005	342444018
smokes drugs through a pipe	342445017	228379002	342445017
cigar consumption	344794017	230057008	344794017
pipe tobacco consumption	344795016	230058003	344795016
[v]tobacco abuse counselling	461114016	711028002	3047058010
very heavy smoker - 40+cigs/d	67621000006112	160606002	250362014
tobacco consumption	102921000006112	266918002	397730015
cigarette consumption	344793011	230056004	344793011
tobacco dependence, unspecified	295256013	89765005	148820017
referral to smoking cessation advisor	1489355012	395700008	1489355012
smokers' cough	78013015	46802002	78013015
pipe smoker	136515019	82302008	136515019
tobacco dependence, episodic	295258014	191888003	295258014
rolls own cigarettes	250375014	160619003	250375014
keeps trying to stop smoking	250368013	160612007	250368013
smoking started	2669652019	266929003	2669652019
provision of smoking cessation leaflet	2982426011	699033005	2982426011
smokes/uses tobacco products	961581000006114	961581000006105	961581000006114
smoking cessation referral declined	1591651000006110	1591651000006106	1591651000006110
referral to stop-smoking clinic	459722017	315232003	459722017
smoking restarted	137791000006118	308438006	2670126018
smoker (read codes)	137711000006111	137711000006107	137711000006111
cigarette smoker	854021000006115	854021000006104	854021000006115
cigar smoker	99639019	59978006	99639019
not interested in stopping smoking	1488578010	394873005	1488578010
reason for restarting smoking	1780360012	401159003	1780360012

current smoker annual review - enhanced services admin	1714541000006110	505651000000103	1152111000000118
not interested in stopping smoking	903981000006117	903981000006101	903981000006117
previous smoking quit attempts	904021000006118	904021000006102	904021000006118
thinking about stopping smoking	904031000006115	904031000006104	904031000006115
carbon monoxide validation of smoking status	904051000006110	904051000006106	904051000006110
smoking cessation bupropion therapy	904221000006110	904221000006106	904221000006110
[rfc] smoking cessation	909391000006117	909391000006101	909391000006117
smoking reduced	216212011	134406006	216212011
current smoker nos	604961000006114	604961000006105	604961000006114
smoker	128130017	77176002	128130017
ready to stop smoking	904001000006111	904001000006107	904001000006111
waking time to first cigarette	904041000006113	904041000006109	904041000006113
carbon monoxide validation confirms smoker	904121000006117	904121000006101	904121000006117
cigarette smoker	108938018	65568007	108938018
failed attempt to stop smoking	1592611000000110	446172000	2882460017
[v]tobacco use	460828018	110483000	175325014
[x]mental and behavioural disorder due to use of tobacco	295951010	30310000	50736017
varenicline smoking cessation therapy offered	2462431000000110	966991000000104	2462431000000110
occasional smoker	397733018	428041000124106	2717161000000119
smoking restarted	2670126018	308438006	2670126018
minutes from waking to first tobacco consumption	2474719011	413173009	2474719011
smoker - amount smoked	137721000006115	266918002	397730015
thinking about stopping smoking	1488576014	394871007	1488576014
moderate smoker - 10-19 cigs/d	700121000006118	160604004	250359011
tobacco dependence, continuous	295257016	191887008	295257016
trivial smoker - < 1 cig/day	88471000006112	266920004	397735013
gradual smoking reduction	852111000006118	852111000006102	852111000006118
current smoker	854071000006119	854071000006103	854071000006119
tobacco consumption nos	250387019	266918002	397730015
grade b light smoker (1-10/day)	854961000006110	854961000006106	854961000006110
rolls own cigarettes	852981000006111	852981000006107	852981000006111
refuses stop smoking monitor	285792013	765001003	3656021018
heavy smoker - 20-39 cigs/day	819331000006110	160605003	250360018
[x]mental and behav dis due to use of tobacco: harmful use	397911000006114	724697004	3445165019
trying to give up smoking	250372012	160616005	250372012
exposure to cigarette/cigar smoke	981841000006115	981841000006104	981841000006115
tobacco dependence nos	295260011	89765005	2951656017
**Past smoker**			
ex-smoker - amount unknown	250371017	8517006	15047015
ex smoker	649841000006110	8517006	15047015
current non-smoker	250374013	160618006	250374013

smoking cessation therapy nos	489931000000114	710081004	3043296015
ex-cigar smoker	852991000006114	852991000006105	852991000006114
ex-smoker nos	853001000006110	853001000006106	853001000006110
ex-trivial smoker (<1/day)	250363016	266921000	397736014
ex-light smoker (1-9/day)	250364010	266922007	397737017
ex-heavy smoker (20-39/day)	250366012	266924008	397739019
ex-very heavy smoker (40+/day)	250367015	266925009	397740017
past smoker	854111000006110	854111000006106	854111000006110
ex cigar smoker	649821000006115	160621008	250377018
ex- rolled tobacco smoker	649851000006112	649851000006108	649851000006112
smoking cessation drug therapy	482771000000118	713700008	3297364011
smoking cessation therapy	492511000000117	710081004	3043296015
ex-smoker annual review - enhanced services administration	1123951000000110	505761000000105	1154471000000114
ex-smoker nos	903041000006110	266928006	903041000006110
cigarette pack-years	1780396011	401201003	1780396011
ex-pipe smoker	854051000006112	854051000006108	854051000006112
date stopped smoking	854151000006111	854151000006107	854151000006111
tobacco dependence in remission	295259018	191889006	295259018
ex-cigarette smoker	418914010	281018007	418914010
smoking cessation-maintain abstinence	852121000006114	852121000006105	852121000006114
other specified smoking cessation therapy	482871000000111	710081004	3043296015
cessation of smoking	2636041000006110	8517006	15048013
stopped smoking	250373019	160617001	250373019
ex pipe smoker	649831000006117	160620009	250376010
ex-moderate smoker (10-19/day)	250365011	266923002	397738010
ex-cigarette smoker	649861000006114	649861000006105	649861000006114

Table S47. Measurements Aurum codes

Term	Medcode ID	SNOMED CT Concept ID	SNOMED CT Description ID
**Height**			
O/E - height	923831000006115	923831000006104	923831000006115
O/E - height	253669010	248333004	370729019
Estimated height	1910921000006114	1910921000006105	1910921000006114
O/E - height NOS	253676017	248333004	370729019
Reported height	1910931000006112	1910931000006108	1910931000006112
**Weight**			
O/E - weight NOS	253688015	27113001	45352010
Baseline weight	1780175010	400967004	1780175010
Weight monitoring	451201014	307818003	451201014
Weight static	2536041016	271398006	2536041016
Reported weight	1910911000006118	1910911000006102	1910911000006118
O/E - weight	923851000006110	923851000006106	923851000006110
Weight screen	59281000006111	804271000000109	1798011000000116
Estimated weight	1910901000006116	1910901000006100	1910901000006116
O/E - weight	253677014	27113001	45352010
**BMI**			
OBESITY MULTIDISCIPLINARY CASE REVIEW	2310101000000115	898011000000103	2310101000000115
INTENSIVE WEIGHT MANAGEMENT PROGRAMME DECLINED	2326491000000114	904631000000104	2326491000000114
OBESE CLASS I (BODY MASS INDEX 30.0 - 34.9)	2350241000000116	914721000000105	2350241000000116
OBESE CLASS II (BODY MASS INDEX 35.0 - 39.9)	2350261000000115	914731000000107	2350261000000115
BODY MASS INDEX	100716012	60621009	100716012
TREATMENT OF OBESITY STARTED	264758010	170799008	264758010
REASON FOR OBESITY THERAPY - OCCUPATIONAL	264768017	170807005	264768017
OBESITY MONITORING NOS	264769013	268522006	401534016
INTERVENTION FOR RISK TO HEALTH ASSOCIATED WITH OVERWEIGHT AND OBESITY, ADVICE ABOUT DIET AND PHYSICAL ACTIVITY	1753321000006116	756021000000103	1663521000000115
INTERVENTION FOR RISK TO HEALTH ASSOCIATED WITH OVERWEIGHT AND OBESITY, ADVICE ABOUT DIET AND PHYSICAL ACTIVITY, CONSIDER DRUGS	1753331000006118	756041000000105	1663561000000111
INTERVENTION FOR RISK TO HEALTH ASSOCIATED WITH OVERWEIGHT AND OBESITY, ADVICE ABOUT DIET AND PHYSICAL ACTIVITY, CONSIDER DRUGS, CONSIDER SURGERY	1753341000006111	756061000000106	1663601000000111
BODY MASS INDEX LESS THAN 20	453856012	310252000	453856012
BODY MASS INDEX NORMAL K/M2	253844013	35425004	59104012
INTENSIVE WEIGHT MANAGEMENT PROGRAMME COMMENCED	2326311000000114	904551000000107	2326311000000114
HAS SEEN DIETICIAN - OBESITY	264756014	170797005	264756014

BODY MASS INDEX 18.5-24.9	1808071000006119	1808071000006103	1808071000006119
BODY MASS INDEX 30+ - OBESITY	253848011	162864005	253848011
OBESITY MONITORING	401534016	268522006	401534016
FOLLOW-UP OBESITY ASSESSMENT	264753018	170795002	264753018
TREATMENT OF OBESITY CHANGED	264757017	170798000	264757017
BODY MASS INDEX HIGH K/M2	253845014	48499001	80800016
BODY MASS INDEX LOW K/M2	253846010	6497000	11777011
INTENSIVE WEIGHT MANAGEMENT PROGRAMME ENDED	2326451000000118	904611000000107	2326451000000118
TELEHEALTH OBESITY MONITORING	2423851000000119	715279006	3302073018
TREATMENT OF OBESITY STOPPED	264759019	170800007	264759019
ATTENDS SLIMMING CLINIC	264760012	170801006	264760012
BODY MASS INDEX 20-24 - NORMAL	2474325012	412768003	2474325012
RISK TO HEALTH ASSOCIATED WITH OVERWEIGHT AND OBESITY, AT HIGH RISK	1753291000006113	755921000000105	1663311000000118
BASELINE BODY MASS INDEX	2196071000000116	846931000000101	2196071000000116
BODY MASS INDEX LESS THAN 18.5	1808061000006114	1808061000006105	1808061000006114
OBESE CLASS III (BMI EQUAL TO OR GREATER THAN 40.0)	1900331000006113	914741000000103	2350281000000112
BODY MASS INDEX 40+ - SEVERELY OBESE	2160062010	408512008	2160062010
BODY MASS INDEX CENTILE	1551651000000111	446974000	2882934011
BODY MASS INDEX INDEX 25-29 - OVERWEIGHT	253847018	162863004	253847018
BODY MASS INDEX	923861000006112	923861000006108	923861000006112
INITIAL OBESITY ASSESSMENT	264752011	170794003	264752011
RISK TO HEALTH ASSOCIATED WITH OVERWEIGHT AND OBESITY, AT NO INCREASED RISK	1753271000006112	755881000000104	1663231000000115
RISK TO HEALTH ASSOCIATED WITH OVERWEIGHT AND OBESITY, AT INCREASED RISK	1753281000006110	755901000000101	1663271000000118
RISK TO HEALTH ASSOCIATED WITH OVERWEIGHT AND OBESITY, AT VERY HIGH RISK	1753301000006114	755941000000103	1663351000000119
INTERVENTION FOR RISK TO HEALTH ASSOCIATED WITH OVERWEIGHT AND OBESITY, GENERAL ADVICE ON HEALTHY WEIGHT AND LIFESTYLE	1753311000006112	756001000000107	1663481000000115
**Blood pressure and Heart rate**			
Systolic arterial pressure	114311000006111	72313002	120159016
Diastolic arterial pressure	619931000006119	1091811000000102	2734671000000117
Heart rate	487210016	364075005	487210016
Baseline heart rate	2378591000000110	928001000000104	2378591000000110

Table S48. Blood test Aurum codes

Term	Medcode ID	SNOMED CT Concept ID	SNOMED CT Description ID
**Creatinine**			
EstimatedCreatinineClearance	2470380013	395680003	2470380013
EstimatedCreatinineClearance(cockcroft-gaultFormula)	2465711000000116	968191000000100	2465711000000116
SerumCreatinine	380389013	1000731000000107	2577271000000111
**eGFR**			
GfrCalculatedAbbreviatedMdrd	976481000006110	1020291000000106	2569781000000116
EgfrUsingCreatinine(ckd-epi)Per1.73SquareMetres	1942831000006114	1011481000000105	2579541000000113
GlomerularFiltrationRate	133205018	80274001	133205018
**Hb**			
Haemoglobin estimation	813551000006113	1022431000000105	2553471000000116
**HbA1C**			
HbA1c (haemoglobin A1c) level (diagnostic reference range) - IFCC (International Federation of Clinical Chemistry and Laboratory Medicine) standardised	1986181000006111	1049301000000100	2643101000000114
HbA1c - Haemoglobin A1c level	3199181000006110	43396009	493589010
Hemoglobin A1c (HBA1c) target	6764291000006117	408591000	2621921019
HbA1c - Hemoglobin A1c level	3199191000006113	43396009	493590018
HbA1c (haemoglobin A1c) target level - IFCC (International Federation of Clinical Chemistry and Laboratory Medicine) standardised	1753211000006115	446074002	1725291000000118
HbA1c measurement (DCCT aligned)	6036701000006116	313835008	457955014
HBA1c (Hemoglobin A1c) level	7285151000006113	443911005	2840604014
HbA1c level (Diabetes Control and Complications Trial aligned)	457954013	1019431000000105	2566161000000115
HbA1c (haemoglobin A1c) molar concentration in blood	12622041000006111	1107481000000106	2773291000000116
HbA1c level (DCCT aligned)	6036711000006118	313835008	457954013
HBA1c target	2160141012	408591000	1739591000000117
HbA1c - Glycated hemoglobin-A1c	7960911000006117	733830002	3474736019
HBA1c (Haemoglobin A1c) level	7285141000006111	443911005	2840603015
Raised HbA1c level	1927791000006119	1927791000006103	1927791000006119
HBA1c target	6764261000006113	408591000	2160141012
HbA1c (haemoglobin A1c) level (monitoring ranges) - IFCC (International Federation of Clinical Chemistry and Laboratory Medicine) standardised	1986191000006114	1049321000000109	2643141000000112
Target HbA1c level	6764271000006118	408591000	2163995019
HbA1c - Glycated haemoglobin-A1c	7960921000006113	733830002	3474737011
HbA1c (haemoglobin A1c) target - IFCC (International Federation of Clinical Chemistry and Laboratory Medicine) standardised	7318701000006114	446074002	1725281000000115
**Fasting lipids**			
High cholesterol	2716221000006111	13644009	3035252016
Fasting serum cholesterol	667191000006111	667191000006107	667191000006111
Cholesterol/HDL ratio	457911015	313811003	457911015

Serum cholesterol very high	259230019	166831007	259230019
Non HDL cholesterol level	8411411000006113	1030411000000101	2595221000000113
Serum cholesterol borderline high	7261561000006118	442234001	2820789017
Serum HDL (high density lipoprotein):non-HDL (high density lipoprotein) cholesterol ratio	8396091000006112	1015271000000109	2607241000000118
Cholesterol screening	5579571000006112	275972003	411949013
Serum non HDL (high density lipoprotein) cholesterol level	8386311000006116	1006191000000106	2607621000000111
Serum cholesterol borderline	12716491000006111	166829003	259228016
Calculated LDL cholesterol level	6550661000006112	395065005	1488764011
Lipoprotein cholesterol ratio measurement	6040921000006112	313988001	458247013
HDL cholesterol	4079571000006118	102737005	166116016
Target serum low density lipoprotein cholesterol level	8241901000006112	774851000000106	1730921000000119
Estimated serum non-high density lipoprotein cholesterol level	1924001000006115	920471000000100	2362791000000114
Serum high density lipoprotein cholesterol level	259232010	1005681000000107	2577901000000110
Serum random HDL (high density lipoprotein) cholesterol level	259243019	1026461000000104	2599971000000117
Serum random LDL (low density lipoprotein) cholesterol level	259247018	1026481000000108	2599981000000115
Serum total cholesterol measurement	6831591000006111	412808005	2474364010
High density lipoprotein cholesterol measurement	2949971000006113	28036006	46931018
Total cholesterol level	8292941000006114	853681000000104	2210431000000111
Cholesterol level	8290381000006115	850981000000101	2204831000000119
Serum VLDL (very low density lipoprotein) cholesterol level	259236013	1003441000000101	2607471000000113
Serum fasting LDL (low density lipoprotein) cholesterol level	259246010	1026471000000106	2599941000000111
Total cholesterol:HDL (high density lipoprotein) ratio	259250015	1028551000000102	2602701000000112
Plasma fasting HDL (high density lipoprotein) cholesterol level	259567011	1028841000000102	2600031000000117
HDL/total cholesterol ratio measurement	4108441000006119	104583003	168549011
Total cholesterol:HDL ratio measurement	4600421000006112	166842003	259249015
LDL-C (low density lipoprotein cholesterol) substance concentration in serum	12623411000006112	1108551000000102	2774161000000118
Target serum LDL (low density lipoprotein) cholesterol level	8241911000006110	774851000000106	1730931000000117
Plasma cholesterol/HDL (high density lipoprotein) ratio	458252015	1015691000000106	2557701000000111
Serum cholesterol/low density lipoprotein ratio	458253013	1015701000000106	2565681000000110
Plasma cholesterol/LDL (low density lipoprotein) ratio	458256017	1015711000000108	2580131000000114
Serum cholesterol/very low density lipoprotein ratio	458257014	1015721000000102	2557711000000113
Plasma HDL (high density lipoprotein) cholesterol level	458313013	1010581000000101	2557051000000110
Calculated LDL (low density lipoprotein) cholesterol level	1488764011	1014501000000104	2552501000000118
HDL - High density lipoprotein cholesterol level	2950001000006114	28036006	483733018
Target serum high density lipoprotein cholesterol level	8241871000006112	774821000000101	1730851000000119
Non-HDL cholesterol	6014411000006114	312260007	455948011

Serum HDL cholesterol measurement	4600201000006115	166832000	259231015
Fasting cholesterol level	6052931000006111	315017003	459479011
HDL (high density lipoprotein) cholesterol/total cholesterol ratio in plasma	12622411000006116	1107731000000104	2771891000000118
Serum fasting HDL (high density lipoprotein) cholesterol level	259242012	1026451000000102	2599931000000119
LDL - Low density lipoprotein cholesterol level	4217681000006112	113079009	380428016
Serum HDL cholesterol level	4600211000006117	166832000	259232010
Cholesterol/HDL ratio measurement	6036221000006115	313811003	457910019
Serum LDL cholesterol level	4600241000006118	166833005	259233017
Serum cholesterol borderline low	7263101000006113	442350007	2819746011
Serum LDL cholesterol measurement	4600231000006111	166833005	259234011
Serum total cholesterol level	6831601000006115	412808005	2478443019
Serum cholesterol/high density lipoprotein ratio	458249011	1015681000000109	2574851000000110
Serum cholesterol level	6279041000006119	365794002	489235016
Serum cholesterol level	150921000006118	1005671000000105	2551271000000118
Total cholesterol:HDL ratio	4600431000006110	166842003	259250015
LDL cholesterol	4079621000006115	102739008	166118015
Serum cholesterol normal	259227014	166828006	259227014
Serum cholesterol raised	259229012	166830008	259229012
Hypercholesterolaemia	475418015	13644009	475418015
Serum triglyceride/HDL cholesterol ratio	1916571000006117	1916571000006101	1916571000006117
Target serum total cholesterol level	8241111000006113	773271000000103	1726681000000112
HDL (high density lipoprotein) cholesterol/total cholesterol ratio in serum	12622421000006112	1107741000000108	2771911000000115
Serum cholesterol/HDL ratio	6040951000006115	313989009	458249011
Fasting lipids	854781000006119	854781000006103	854781000006119
Serum non high density lipoprotein cholesterol level	2344491000000115	1006191000000106	2585861000000117
Plasma random HDL (high density lipoprotein) cholesterol level	259564016	1028831000000106	2600041000000114
Plasma random LDL (low density lipoprotein) cholesterol level	259569014	1028851000000104	2600051000000112
Plasma fasting LDL (low density lipoprotein) cholesterol level	259570010	1028861000000101	2600061000000110
Plasma cholesterol/VLDL (very low density lipoprotein) ratio	458260019	1015731000000100	2580141000000117
Serum HDL (high density lipoprotein) cholesterol level	8385731000006118	1005681000000107	2607231000000110
HDL (high density lipoprotein) cholesterol substance concentration in serum	12622321000006118	1107681000000108	2771791000000114
HDL : total cholesterol ratio	4108451000006117	104583003	279462018
HDL cholesterol level	855771000006118	855771000006102	855771000006118
Serum cholesterol borderline	259228016	166829003	259228016
Serum total cholesterol level	2478443019	994351000000103	2581451000000115
Cholesterol	34521000033114	84698008	34521000033114
Serum cholesterol NOS	259252011	1005671000000105	2551271000000118
Pre-treatment serum cholesterol level	1488851015	395153009	1488851015
Serum HDL/non-HDL cholesterol ratio	5519791000006113	271059008	405628014

Serum cholesterol/HDL (high density lipoprotein) ratio	8396611000006112	1015681000000109	2607531000000112
Plasma LDL (low density lipoprotein) cholesterol level	458314019	1010591000000104	2586471000000117
Serum high density lipoprotein:non-high density lipoprotein cholesterol ratio	144141000006119	1015271000000109	2565581000000114
Serum low density lipoprotein cholesterol level	259233017	1022191000000100	2566581000000116
Cholesterol	3877651000006116	84698008	140417016
LDL cholesterol level	857141000006116	857141000006100	857141000006116
Serum cholesterol/LDL ratio	6041011000006113	313991001	458253013
Low density lipoprotein cholesterol measurement	4217651000006116	113079009	186163011
Plasma total cholesterol level	1484985012	1017161000000104	2552911000000113
Target cholesterol level	1484928011	390896004	1484928011
Target serum cholesterol	107301000006115	107301000006104	107301000006115
Total cholesterol measurement	186091018	121868005	186091018
Target serum non high density lipoprotein cholesterol level	13602641000006117		

Table S49. Atrial ablation Aurum codes

Term	Medcode ID	SNOMED CT Concept ID	SNOMED CT Description ID
Open ablation of atrioventricular node	271176014	175095005	271176014
Percutaneous transluminal ablation of atrioventricular node	271209012	175125003	271209012
Percutaneous transluminal ablation of accessory pathway	2694913017	428581004	2694913017
Percutaneous transluminal ablation of conducting system of heart	238631000006110	448042001	2900001016
Percutaneous transluminal chemical mediated septal ablation	357821000000110	437746009	3037266010
Percutaneous transluminal ablation of wall of atrium	376621000000113	428290007	2693316014
Percutaneous transluminal laser ablation of vein	385021000000117	609165007	2958779019
Percutaneous transluminal ablation of atrial wall NEC	488161000000110	428290007	2693316014
Transluminal radiofreq ablation heart conducting system NEC	998521000006116	448042001	2900001016
Percutaneous transluminal ablation of congenital heart malformation	1547441000006113	428391009	2692484013
Percutaneous transluminal ablation of pulmonary vein to left atrium conducting system	1587601000006117	707831001	3032482017
Percutaneous transluminal ablation of atrial wall for atrial flutter	1587611000006119	707832008	3032484016
Percutaneous radiofrequency ablation of epicardium	1686211000000116	609233003	2958816014
Catheter ablation of tissue of heart	2790601000006119	18286008	30881015
Catheter ablation of lesion of heart	3076931000006116	35823001	59752012
Ablation	3550661000006116	64597002	199515017
Rotablation	3567581000006119	65659003	2478792014
Radiofrequency ablation procedure phase	4422211000006110	129089003	207797015
Ablation operation for arrhythmia	5046591000006116	233159005	349323018
Diathermy ablation operation for arrhythmia	5046601000006112	233160000	349324012
Cryoablation operation for arrhythmia	5046611000006110	233161001	349325013
Laser ablation operation for arrhythmia	5046621000006119	233162008	349326014
Radiofrequency ablation operation for arrhythmia	5046631000006116	233163003	349327017
Ablation - action	5386721000006118	257729003	383646017
Ablation	5386731000006115	257729003	383645018
Cryoablation - action	5653621000006118	281609006	419659014
Cryoablation	5653641000006113	281609006	419661017
Radiofrequency ablation device	6360191000006115	371791001	1210387010
Ablation frequency	6450421000006111	386132006	1480316015
Percutaneous transluminal coronary angioplasty by rotoablation	6583111000006116	397193006	1776881019
History of cardiac radiofrequency ablation	7081241000006112	427951003	2694709018
Percutaneous transluminal ablation	7087871000006111	428389001	2693563010
Ablation of atrioventricular node	7091821000006115	428663009	2693280013
CT guided laser ablation	7111561000006115	429929004	3027495010
CT guided ablation	7117191000006114	430269006	3027505015
Endocardial pulmonary vein ablation	7142291000006112	431626004	706461000000113

Endocardial ablation of pulmonary vein using fluoroscopic guidance	7142281000006114	431626004	2772721014
Percutaneous transluminal septal myocardial ablation	7199391000006119	437746009	2793908011
Catheter ablation for cardiac arrhythmia	7484761000006112	473229000	2956223014
Cardiac ultrasound ablation system	7556141000006117	702209009	3014237014
Cardiac radiofrequency ablation system	7594051000006116	704706000	3014227016
Cardiac ablation system	7603631000006111	705732002	3024696011

Table S50. Hyperthyoidism Aurum codes

Term	Medcode ID	SNOMED CT Concept ID	SNOMED CT Description ID
H/O: hyperthyroidism	251587014	161442007	251587014
Toxic adenomatous goitre	3438831000006118	57777000	497989019
Toxic uninodular goitre with thyrotoxic crisis	3627481000006117	69329005	501258013
Graves' eye disease	5581851000006111	276177000	2536088017
Thyrotoxicosis without mention of goitre or cause no crisis	101891000006113	90739004	150375010
Thyrotoxicosis with toxic single thyroid nodule	3700961000006117	73869005	502554013
Toxic multinodular goitre with crisis	292342015	62278002	103507011
Toxic diffuse goitre NOS	292331012	267374005	398780011
Toxic uninodular goitre NOS	292338018	73869005	200396013
Toxic multinodular goitre NOS	292343013	26389007	196525014
Thyrotoxicosis of other specified origin with no crisis	292361016	90739004	150375010
Thyrotoxicosis without mention of goitre or other cause	292366014	90739004	150375010
Graves' disease	472452010	353295004	472452010
Thyrotoxicosis from ectopic thyroid tissue	3918281000006110	87232008	507482011
Thyrotoxicosis due to uninodular goitre	3700971000006112	73869005	502555014
Thyrotoxicosis with or without goiter	5721001000006113	286909009	426394012
Thyrotoxicosis from ectopic thyroid nodule	507481016	87232008	507481016
Thyrotoxic exophthalmos	33469013	19885005	33469013
Toxic multinodular goitre with thyrotoxic storm	3511901000006114	62278002	499238017
Toxic multinodular goitre with thyrotoxic crisis	3511871000006114	62278002	499237010
Hyperthyroidism	57561015	34486009	57561015
Hyperthyroidism due to ectopic thyroid tissue	4757781000006116	190255006	3035757011
Thyrotoxicosis due to multinodular goitre	2922501000006118	26389007	483220013
History of hyperthyroidism	4539511000006111	161442007	2986456017
Toxic multinodular thyroid goitre	2922531000006114	26389007	3036943012
[RFC] Hyperthyroidism	905741000006112	905741000006108	905741000006112
Toxic diffuse goitre with crisis	292330013	190242005	292330013
Toxic diffuse goitre	398780011	267374005	398780011
Graves' ophthalmopathy	5581801000006112	276177000	2536083014
Toxic multinodular thyroid goiter	2922521000006111	26389007	3036654016
Toxic uninodular goitre	200396013	73869005	200396013
Thyrotoxicosis of other specified origin	398782015	90739004	150375010
Thyrotoxicosis with diffuse goiter	5500071000006116	267374005	398778017
Thyrotoxicosis of other specified origin NOS	292365013	90739004	150375010
Toxic uninodular goitre with thyrotoxic storm	3627511000006113	69329005	200068013
Toxic nodular goitre unspecified	292346017	57777000	497988010
Toxic multinodular goiter with thyrotoxic crisis	3511881000006112	62278002	103507011
Thyrotoxicosis due to uninodular goiter	3700981000006110	73869005	502556010
Thyrotoxicosis due to multinodular goiter	2922481000006111	26389007	483218010

Toxic multinodular goiter with thyrotoxic storm	3511891000006110	62278002	103508018
Toxic nodular goitre unspecified with no crisis	292347014	57777000	96075015
Diffuse toxic goitre	5500051000006114	267374005	398776018
Thyrotoxicosis from ectopic thyroid nodule with no crisis	292355019	190255006	292355019
Thyrotoxicosis from ectopic thyroid nodule with crisis	292356018	190256007	292356018
Toxic uninodular goitre with crisis	292335015	69329005	115161014
Toxic diffuse goitre with no crisis	292327018	190241003	292327018
Thyrotoxicosis with toxic multinodular goitre	2922511000006115	26389007	483221012
Toxic nodular goiter	3438791000006113	57777000	96075015
Thyrotoxicosis without mention of goitre, cause with crisis	101911000006110	90739004	150375010
Toxic nodular goitre NOS	292354015	57777000	497988010
Thyrotoxicosis NOS	292372014	90739004	150375010
Toxic uninodular thyroid goitre	3700991000006113	73869005	3035712019
Toxic multinodular goiter	2922461000006118	26389007	44198012
Graves' dermopathy	5112881000006118	237825005	356407011
Toxic goitre	355932011	237498007	355932011
Toxic multinodular goitre	196525014	26389007	196525014
Thyrotoxicosis with toxic multinodular goiter	2922491000006114	26389007	483219019
Thyrotoxicosis +/- goitre	881371000006111	286909009	881371000006111
Toxic diffuse thyroid goitre	5500101000006114	267374005	3037800012
Thyrotoxic myopathy	3613541000006112	68437005	113658014
Thyrotoxicosis with or without goitre	5720991000006112	286909009	426395013
Thyrotoxic crisis	2966801000006110	29028009	48598011
Hyperthyroidism resolved	2548990010	416477006	2548990010
Thyrotoxicosis from ectopic thyroid nodule NOS	292357010	87232008	144647016
Thyrotoxicosis of other specified origin with crisis	292362011	90739004	150375010
Thyrotoxicosis with diffuse goitre	5500041000006112	267374005	398775019
Toxic multinodular goiter with no crisis	4757741000006110	190247004	292340011
Hyperthyroidism due to ectopic thyroid nodule	3918261000006117	87232008	144647016
Thyrotoxic heart disease	100438013	60446003	100438013
Thyrotoxicosis	150375010	90739004	150375010
Toxic uninodular goitre with no crisis	292334016	190244006	292334016
Toxic multinodular goitre with no crisis	292339014	190247004	292339014
Toxic nodular goitre unspecified with crisis	292350012	57777000	96075015

Table S51. Hypothyroidism Aurum codes

Term	Medcode ID	SNOMED CT Concept ID	SNOMED CT Description ID
Myasthenic syndrome due to hypothyroidism	297588017	193212008	297588017
Congenital hypothyroidism with diffuse goitre	415432015	278503003	415432015
H/O: hypothyroidism	251589012	161443002	251589012
Hypothyroidism annual review	637341000000118	341861000000109	637341000000118
Hypothyrotropic hypothyroidism	3843711000006117	82598004	505170011
Congenital hypothyroidism with diffuse goiter	5614081000006117	278503003	415433013
Postsurgical hypothyroidism	2933341000006117	27059002	2536050019
Primary hypothyroidism AND adrenocortical insufficiency	3862381000006114	83728000	505751010
Other postablative hypothyroidism	292394018	237527007	355975017
Postablative hypothyroidism NOS	292396016	237527007	355975017
Cerebral degeneration due to myxoedema	542671000006117	192816007	297016016
Diabetes mellitus, Addison's disease and myxoedema	3862341000006115	83728000	505747013
Myxoedema	493538010	43153006	493538010
Endemic cretinism - neurological type	5108961000006115	237566004	356025012
Total substernal thyroidectomy	858351000006117	52826006	858351000006117
Complete thyroidectomy	40998019	24443003	40998019
Other acquired hypothyroidism	12729831000006113	654491000000108	1438601000000113
Hypothyroidism with positive thyroid antibodies	5108381000006112	237519003	355965015
Hypothyroidism-congen.+ acqui.	881381000006114	111566002	881381000006114
Primary hypothyroidism	4197341000006114	111566002	363808017
Congenital hypothyroidism without goitre	355957018	237515009	355957018
Autoimmune myxoedema	355964016	237519003	355964016
Post-infectious hypothyroidism	5108491000006117	237528002	355976016
Cretinism	600791000006112	217710005	329968011
Irradiation hypothyroidism	292395017	190277005	292395017
Hypothyroidism resulting from phenylbutazone	292402012	190283008	292402012
Acquired hypothyroidism	178809013	111566002	178809013
Hypothyroidism monitoring verbal invite	2548835019	416326003	2548835019
Congenital hypothyroidism NOS	398783013	190268003	292373016
Postoperative hypothyroidism	2933321000006112	27059002	2532363015
Hypothyroidism monitoring administration	940091000006115	940091000006104	940091000006115
Hypothyroidism monitoring invite 2	940111000006112	940111000006108	940111000006112
Hypothyroidism monitoring invite 3	940121000006116	940121000006100	940121000006116
Hypothyroidism monitoring administration	225971000000116	713741000000106	1564981000000116
Hypothyroidism monitoring telephone invitation	406721000000115	248341000000100	406721000000115
Iatrogenic hypothyroidism	292400016	88273006	146340011
Iatrogenic hypothyroidism NOS	292404013	88273006	146340011
Myxoedema encephalopathy	4768951000006118	192816007	297016016
Other acquired hypothyroidism	292408011	111566002	178809013

Hypothyroidism clinical management plan	2474336017	736287007	3517030018
Post-surgical hypothyroidism	2536050019	27059002	45271010
Autoimmune hypothyroidism	5108351000006116	237519003	355966019
Acquired hypothyroidism NOS	881391000006112	654491000000108	881391000006112
Hypothyroidism resulting from para-aminosalicylic acid	292401017	190282003	292401017
History of hypothyroidism	4539531000006117	161443002	2986407018
Endemic cretinism - mixed type	5108941000006119	237565000	356023017
Hypothyroidism NOS	12727341000006116	681051000000104	1492161000000113
Hypothyroidism confirmed	1823931000006116	1823931000006100	1823931000006116
[X]Systemic atrophy affecting the CNS in myxoedema	428591000006112	23853001	40063015
Postinfectious hypothyroidism	211321000006115	237528002	355976016
Congenital hypothyroidism without goiter	5108301000006115	237515009	355958011
Congenital iodine-deficiency syndrome, myxoedematous type	583291000006115	75065003	502897012
Atrophy of thyroid - acquired	5108811000006113	237558008	356013014
Hypothyroidism resulting from resorcinol	292403019	190284002	292403019
Congenital thyroid insufficiency	588661000006115	190268003	292373016
Myopathy due to myxoedema	682701000006117	87844004	507778013
Complete parathyroidectomy	2696261000006111	12330002	21229013
Hypothyroidism monitoring second letter	2533534011	414441001	2533534011
Hypothyroidism medication review	939981000006114	939981000006105	939981000006114
Hypothyroidism monitoring invite 1	940101000006114	940101000006105	940101000006114
Hypothyroidism with sensorineural deafness	3644031000006116	70348004	116847011
Pretibial myxoedema - hypothyroid	208391000006113	40930008	68268011
Thyroid insufficiency	101581000006112	40930008	68268011
Acquired hypothyroidism NOS	988831000006119	111566002	988831000006119
Goitrous cretin	415431010	278503003	415431010
Endemic cretinism	12704331000006114	75065003	124678011
Myxoedema coma	35727012	21263006	35727012
Acquired thyroid atrophy	5108831000006119	237558008	3037562011
Hypothyroidism NOS	398784019	40930008	68268011
Congenital hypothyroidism	292373016	190268003	292373016
Thyroid atrophy	292448015	190309006	292448015
Other specified congenital hypothyroidism	292376012	190268003	292373016
Myxoedema myopathy	3927661000006113	87844004	507778013
Total thyroidectomy	40996015	24443003	40996015
Cretinism	329968011	217710005	329968011
Hypothyroidism monitoring third letter	2533420013	414442008	2533420013
Hypothyroidism monitoring first letter	2533422017	414439002	2533422017
Hypothyroidism after surgery	2933351000006115	27059002	3037380018
Cerebellar ataxia due to myxoedema	542301000006117	192876003	297115015
[X]Other sp cified hypothyroidism	413721000006119	40930008	68268011

Myxoedema cerebellar degeneration	4769091000006117	192876003	297115015
Congenital hypothyroidism not due to iodine deficiency	4904391000006111	217710005	329969015
Hypothyroidism clinical management plan no longer in place	1877411000006112	1877411000006108	1877411000006112
Endemic cretinism - hypothyroid	3720391000006119	75065003	502894017
Acquired atrophy of thyroid	356012016	237558008	356012016
Aplasia of thyroid with myxoedema	4012861000006111	92978002	510171015
Thyroid deficiency	101171000006116	40930008	68268011
Postablative hypothyroidism	216181000006112	237527007	355975017
Hypothyroidism	68268011	40930008	68268011
Iodine hypothyroidism	292397013	190279008	292397013

Table S52. Chronic obstructive pulmonary disease Aurum codes

Term	Medcode ID	SNOMED CT Concept ID	SNOMED CT Description ID
COPD GOLD group C	11932351000006110	11932351000006106	11932351000006110
COPD GOLD group D	11932361000006112	11932361000006108	11932361000006112
Chronic asthmatic bronchitis	301450011	195949008	301450011
Chronic wheezy bronchitis	301451010	195949008	301451010
Chronic bullous emphysema	301460019	195957006	301460019
Segmental bullous emphysema	301463017	195958001	301463017
Zonal bullous emphysema	301464011	195959009	301464011
Access to online patient COPD education given	1916531000006115	1916531000006104	1916531000006115
Mild chronic obstructive pulmonary disease	457168017	313296004	457168017
Moderate chronic obstructive pulmonary disease	457169013	313297008	457169013
Severe chronic obstructive pulmonary disease	457171013	313299006	457171013
COPD (chronic obstructive pulmonary disease) 6 monthly review	8235131000006118	760621000000103	1710191000000113
COPD self-management plan review	1811741000006117	810951000000101	2115731000000110
On COPD (chr obstruc pulmonary disease) supportv cre pathway	1813881000006119	826111000000109	2155811000000119
Asthma-chronic obstructive pulmonary disease overlap syndrom	1948051000006112	10692761000119107	3046456013
Chron obstruct pulmonary dis wth acute exacerbation, unspec	553211000006119	195951007	301453013
Chr. airway obstruction NOS	990651000006112	13645005	990651000006112
Chronic obstructive pulmonary disease	475431013	13645005	475431013
Admit COPD emergency	12489801000006116	408501008	2160051010
Telehealth chronic obstructive pulmonary disease monitoring	2423731000000115	716358000	3305683010
COPD (chronic obstructive pulmonary disease) disturbs sleep	8058301000006110	198401000000104	1677391000000113
Chronic bronchitis, acute exac	851261000006116	851261000006100	851261000006116
Simple chronic bronchitis NOS	301444018	61937009	102938011
Other chronic bronchitis NOS	301458016	63480004	105519017
Chronic bullous emphysema NOS	301468014	195957006	301460019
COPD patient unsuitable for pulmonary rehabilitation	1656601000006119	1656601000006103	1656601000006119
Giant bullous emphysema	27096010	16003001	27096010
Shared care COPD (chronic obstructive pulmonary disease) monitoring	8316481000006116	897311000000101	2312551000000112
Panacinar emphysema	2578881000006118	4981000	9336013
Chronic obstructive pulmonary disease disturbs sleep	299001000000116	198401000000104	299001000000116
Obliterative bronchiolitis due to chemical fumes	269761000006112	196027008	301573017
Not suitable for step down change in COPD management plan	2009511000006113	2009511000006109	2009511000006113
Acute non-infective exacerbation of chronic obstructive pulmonary disease	2010061000006113	847091000000104	2196421000000114
COPD (chronic obstructive pulmonary disease) care pathway	8288261000006119	848431000000106	2199291000000116
ACOS - asthma-chronic obstructive pulmonary disease overlap syndrome	9317341000006115	10692761000119107	2496071000000117

Acute exacerbation of COPD	4781421000006111	195951007	3012683010
Obstructive chronic bronchitis	4733021000006111	185086009	285104011
MacLeod's unilateral emphysema	1230190015	45145000	1230190015
Seen in chronic obstructive pulmonary disease clinic	1839331000006117	1839331000006101	1839331000006117
QOF (Quality and Outcomes Framework) chronic obstructive pulmonary disease quality indicator-related care invitation	12626281000006113	1110861000000102	2779701000000115
Chronic obstructive pulmonary disease annual review	1488424013	394703002	1488424013
Chronic obstructive pulmonary disease leaflet given	1488856013	395159008	1488856013
Chronic obstructive pulmonary disease monitoring by nurse	1780380013	401184000	1780380013
Chronic obstructive pulmonary disease monitoring by doctor	1780381012	401185004	1780381012
Centrilobular emphysema	113497011	68328006	113497011
COB - Chronic obstructive bronchitis	4733011000006115	185086009	285103017
COPD (chronic obstructive pulmonary disease) management plan declined	8312921000006111	892321000000109	2297721000000117
Chronic obstructive pulmonary disease severity	1856571000006116	1856571000006100	1856571000006116
Chronic bronchitis with emphysema	4732991000006119	185086009	285101015
Chronic emphysema caused by chemical fumes	4781821000006113	196026004	3296151011
COPD follow-up	845451000006118	394702007	1488423019
COPD structured smoking assessment declined	1704581000006119	375911000000102	739421000000116
[RFC] Chronic obstructive pulmonary disease (COPD)	909711000006111	909711000006107	909711000006111
[RFC] Emphysema	909721000006115	909721000006104	909721000006115
Atrophic (senile) emphysema	396109014	266356006	396109014
1 COPD exacerbation in past year	1882421000006113	1882421000006109	1882421000006113
3+ COPD exacerbations in past year	1882431000006111	1882431000006107	1882431000006111
2 COPD exacerbations in past year	1882441000006118	1882441000006102	1882441000006118
Interstitial emphysema	12704691000006117	77690003	770361000006117
COLD - Chronic obstructive lung disease	2716351000006113	13645005	475430014
COPD (Chronic obstructive pulmonary disease) patient unsuitable for pulmonary rehabilitation	8120981000006112	371611000000107	730041000000110
COPD (chronic obstructive pulmonary disease) written self management plan declined	8294671000006116	857811000000102	2219931000000113
COPD medication review	939991000006112	939991000006108	939991000006112
COPD monitoring administration	940131000006118	940131000006102	940131000006118
COPD monitoring invite 2	940151000006113	940151000006109	940151000006113
COPD monitoring invite 3	940161000006110	940161000006106	940161000006110
Chronic obstructive pulmonary disease monitoring verb invite	967571000006116	716281000000103	1568831000000118
Emergency COPD admission since last appointment	977901000006111	414087000	2533539018
Refer COPD structured smoking assessment - enhanc serv admin	1704561000006112	375851000000108	739301000000115
COPD structured smoking assessment declined - enh serv admin	1704571000006117	375911000000102	739421000000116
Mucopurulent chronic bronchitis NOS	301448015	74417001	123588010
End stage chronic obstructive pulmonary disease	4510801000006114	135836000	3034045017

Chronic obstructive pulmonary disease 3 monthly review	1683181000000112	760601000000107	1683181000000112
Bullous emphysema with collapse	396108018	266355005	396108018
Preferred place of care for next exacerbation of COPD	1784071000006113	789661000000102	1765431000000114
GP OOH service notified of COPD care plan	1785171000006113	783631000000109	1752261000000117
Health education - chronic obstructive pulmonary disease	405121000000111	741056003	3543021016
Chronic obstructive pulmonary disease care pathway	2199261000000110	848431000000106	2199261000000110
COPD (chronic obstructive pulmonary disease) rescue pack declined	8294651000006114	857791000000103	2219871000000119
Interstitial emphysema	19421011	77690003	503646013
Optimization of medication for chronic obstructive lung disease	7484341000006112	473202005	2956280014
Step down change in chronic obstructive pulmonary disease management plan	3011135010	704123000	3011135010
Suitable for step down change in COPD management plan	2009501000006110	2009501000006106	2009501000006110
Acute infective exacerbation of chronic obstructive airways disease	424365019	285381006	424365019
Chronic obstructive pulmonary disease post discharge review	2009451000006113	2009451000006109	2009451000006113
Acute non-infective exacerbation of COPD (chronic obstructive pulmonary disease)	8287171000006115	847091000000104	2196431000000111
COPD self-management plan given	839001000006111	390891009	1484924013
[X]Other emphysema	301835010	87433001	144964015
Other emphysema NOS	396110016	87433001	144964015
COPD GOLD group A	11932331000006115	11932331000006104	11932331000006115
Chr. airway obstruction NOS	885301000006119	611541000000106	885301000006119
Acute exacerbation of chronic obstructive airways disease	301453013	195951007	301453013
Chronic obstructive pulmonary disease monitoring 1st letter	189761000000117	716241000000106	1568801000000112
Issue of COPD (chronic obstructive pulmonary disease) rescue pack	8219501000006114	718241000000107	1573141000000119
Shared care chronic obstructive pulmonary disease monitoring	1882371000006118	1882371000006102	1882371000006118
COPD (chronic obstructive pulmonary disease) does not disturb sleep	8058321000006117	198411000000102	1677401000000111
GP (general practitioner) OOH (out of hours) service notified of COPD (chronic obstructive pulmonary disease) care plan	8244871000006114	783631000000109	1752291000000111
Fetid chronic bronchitis	3873191000006110	84409004	139979010
Chronic catarrhal bronchitis	508562012	89549007	508562012
COPD (chronic obstructive pulmonary disease) 3 monthly review	8235101000006114	760601000000107	1709911000000112
COPD clinical pathway protocol followed	1824071000006115	1824071000006104	1824071000006115
Chronic obstruct pulmonary dis with acute lower resp infectn	555461000006119	196001008	301542016
Vesicular emphysema	2578901000006116	4981000	9338014
Chronic obstructive pulmonary disease monitoring due	1484971019	390941006	1484971019
Chronic obstructive pulmonary disease follow-up	1488423019	394702007	1488423019
Interstitial pulmonary emphysema	3764031000006119	77690003	3764031000006119
Mixed simple and mucopurulent chronic bronchitis	301456017	195953005	301456017

Acute vesicular emphysema	301470017	195963002	301470017
Chronic obstructive bronchitis	4733031000006114	185086009	2668815014
Suspected chronic obstructive pulmonary disease	314261000000118	204991000000107	314261000000118
Optimisation of medication for chronic obstructive lung disease	7484351000006114	473202005	3297724010
Chronic obstructive airways disease	555471000006114	13645005	475423015
Emphysema	640491000006111	87433001	144964015
Chronic obstructive pulmonary disease monitoring admin	967531000006119	713731000000102	1564971000000118
Bullous emphysema	4781471000006112	195957006	301462010
Referral to COPD community nursing team	1784371000006111	790301000000101	1766811000000114
Chronic obstructive pulmonary disease clini management plan	966841000006111	736283006	3517019016
Chronic obst. pulm. dis. NOS	885311000006116	611541000000106	885311000006116
Emphysema of lung	3921361000006112	87433001	144967010
Other chronic bronchitis	301457014	63480004	105519017
Chronic bronchitis NOS	301459012	63480004	105519017
[X]Other specified chronic obstructive pulmonary disease	301836011	13645005	475431013
Seen in chronic obstructive pulmonary disease clinic	2219551000000113	857661000000104	2219551000000113
Very severe chronic obstructive pulmonary disease	516801000000112	293991000000106	516801000000112
Chronic emphysema due to chemical fumes	301572010	196026004	301572010
Chronic bronchitis	105519017	63480004	105519017
End stage chronic obstructive airways disease	216596014	135836000	216596014
Has chronic obstructive pulmonary disease care plan	2152091000000112	827571000000106	2152091000000112
Seen in COPD (chronic obstructive pulmonary disease) clinic	8294561000006110	857661000000104	2219581000000119
Centriacinar emphysema	3611791000006110	68328006	113496019
Chronic obstructive lung disease	2716231000006114	13645005	23287019
Emergency hospital admission for chronic obstructive pulmonary disease	6763231000006119	408501008	6763231000006119
Panlobular emphysema	9337016	4981000	9337016
Shared care chronic obstructive pulmonary disease monitoring	2308511000000113	897311000000101	2308511000000113
Chronic obstructive airways disease NOS	301545019	13645005	475428012
Obstructive chronic bronchitis NOS	301455018	185086009	285100019
Emphysema NOS	301477019	87433001	144964015
Chronic obstructive pulmonary disease rescue pack declined	2219861000000114	857791000000103	2219861000000114
Eosinophilic bronchitis	2240631000000119	866901000000103	2240631000000119
COPD monitoring invite 1	940141000006111	940141000006107	940141000006111
COPD patient unsuitable for pulmonary rehabilitation	1704541000006113	371611000000107	1152601000000114
Other emphysema	301469018	87433001	144964015
Chronic tracheobronchitis	87480013	52571006	87480013
COPD - Chronic obstructive pulmonary disease	2716321000006116	13645005	475427019
Unilateral emphysema	3228301000006115	45145000	75281018

Chronic obstructive airways disease NOS	12728161000006116	611541000000106	1352161000000113
Fetid chronic bronchitis	139979010	84409004	506053014
Obstructive chronic bronchitis	285104011	185086009	285100019
Referred for COPD structured smoking assessment	1704551000006110	375851000000108	739301000000115
COPD GOLD group B	11932341000006113	11932341000006109	11932341000006113
Chronic obstructive pulmonary disease monitoring in primary care	1856491000006119	1856491000006103	1856491000006119
Chronic obstructive pulmonary disease monitoring secondary care	1856501000006110	1856501000006106	1856501000006110
Chronic obstructive pulmonary disease multidisciplinary review	1771201000006116	1771201000006100	1771201000006116
Referral to COPD (chronic obstructive pulmonary disease) community nursing team	8249051000006111	790301000000101	1766841000000110
At risk of COPD (chronic obstructive pulmonary disease) exacerbation	8125721000006119	383611000000102	756791000000118
Chronic obstructive pulmonary disease confirmed	1823851000006119	1823851000006103	1823851000006119
Acute exacerbation of chronic obstructive pulmonary disease	4781431000006114	195951007	3012710012
Issue of chronic obstructive pulmonary disease rescue pack	1573111000000115	718241000000107	1573111000000115
Emergency hospital admission for COPD (chronic obstructive pulmonary disease)	6763251000006114	408501008	3082978011
History of chronic obstructive airway disease	5516841000006119	270473001	2986462010
Interstitial emphysema of lung	3764021000006117	77690003	128944012
Chronic obstructive pulmonary disease follow-up assessment	1856591000006115	1856591000006104	1856591000006115
COPD accident and emergency attendance since last visit	977891000006112	413845009	2533538014
Multiple COPD emergency hospital admissions	998281000006115	198901000000105	300201000000114
Chronic obstructive pulmonary disease monitoring 3rd letter	199481000000116	717521000000104	1570061000000113
Chronic obst. pulm. dis. NOS	990641000006110	13645005	990641000006110
Number of COPD exacerbations in past year	977911000006114	723245007	3335171010
On chronic obstructive pulmonary disease supprtv cre pathway	1813871000006117	826111000000109	2149041000000110
Other specified chronic obstructive airways disease	301539010	13645005	475428012
Purulent chronic bronchitis	506053014	84409004	506053014
Chronic obstructive pulmonary disease monitoring	1484924013	390891009	1484924013
Chronic obstructive pulmonary disease 6 monthly review	1683221000000119	760621000000103	1683221000000119
Alveolar emphysema of lung	2578911000006118	4981000	9339018
COPD management plan declined	1880061000006110	1880061000006106	1880061000006110
COPD patient unsuitable for pulmonary rehab - enh serv admin	1704531000006115	371611000000107	1152601000000114
COPD self-management plan agreed	1811661000006110	811961000000106	2117851000000118
Admit COPD emergency	2160051010	408501008	3082901016
COPD (chronic obstructive pulmonary disease) self-management plan review	8260631000006112	810951000000101	2115741000000118
Asthma-COPD overlap syndrome (ACOS)	9317331000006113	10692761000119107	3046475015
Chronic obstructive pulmonary disease monitor phone invite	967581000006118	716901000000101	1569451000000115
Optimization of medication for chronic obstructive pulmonary disease	7484331000006119	473202005	2956201010

Simple chronic bronchitis	508561017	61937009	102938011
Other specified chronic obstructive pulmonary disease	1222334016	13645005	475431013
Chronic obstructive pulmonary disease NOS	1222335015	13645005	475431013
Chronic obstructive pulmonary disease monitoring 2nd letter	198471000000111	717021000000106	1569571000000113
Chronic obstructive pulmonary disease NOS	12759361000006112	611541000000106	1352151000000110
Emphysematous bulla	4781461000006117	195957006	301461015
Mucopurulent chronic bronchitis	123588010	74417001	123588010
GP out of hours notified of COPD care plan	1763751000006117	1763751000006101	1763751000006117
History of chronic obstructive pulmonary disease	1765681000000110	270473001	1765681000000110
Emphysematous bronchitis	285100019	185086009	285100019

Table S53. Diabetic retinopathy Aurum codes

Term	Medcode ID	SNOMED CT Concept ID	SNOMED CT Description ID
Moderate nonproliferative diabetic retinopathy of left eye	12085211000006112	769186002	3689288014
O/E - sight threatening diabetic retinopathy	2549896013	417677008	2549896013
Diabetic retinopathy clinic	7567371000006117	702850009	3006342012
Left proliferative diabetic retinopathy	856621000006118	856621000006102	856621000006118
Left non-proliferative diabetic retinopathy	857031000006113	857031000006109	857031000006113
Right proliferative diabetic retinopathy	857411000006110	857411000006106	857411000006110
Right non-proliferative diabetic retinopathy	857971000006110	857971000006106	857971000006110
On examination - right eye background diabetic retinopathy	6761781000006114	408409007	2621799017
Diabetic retinopathy screening offered	12121381000006114	1103701000000102	2763011000000112
Proliferative diabetic retinopathy - high risk	6022721000006112	312907002	456707011
On examination - right eye preproliferative diabetic retinopathy	6761821000006115	408411003	2621610013
DR - Diabetic retinopathy	2576431000006117	4855003	1230610015
Proliferative diabetic retinopathy	98476015	59276001	98476015
On examination - left eye stable treated proliferative diabetic retinopathy	6866541000006117	414894003	2621811014
Severe NPDR (nonproliferative diabetic retinopathy)	12224761000006115	312905005	3691865016
High risk proliferative diabetic retinopathy	1785163015	312907002	1785163015
O/E - right eye proliferative diabetic retinopathy	2159977011	408413000	2159977011
O/E - left eye proliferative diabetic retinopathy	2159978018	408414006	2159978018
Nonproliferative diabetic retinopathy	6515171000006112	390834004	2643005014
On examination - right eye stable treated proliferative diabetic retinopathy	6866901000006111	414910007	2621810010
Mild nonproliferative diabetic retinopathy	6022611000006112	312903003	2921114017
Mild nonproliferative diabetic retinopathy of right eye	12085071000006114	769183005	3689271019
Preproliferative diabetic retinopathy of left eye	12085051000006116	769182000	3689267017
Very severe proliferative diabetic retinopathy	6630581000006113	399865004	1779161016
O/E - left eye stable treated proliferative diabetic retinopathy	975261000006113	414894003	2532964019
Severe nonproliferative diabetic retinopathy of right eye	12085251000006113	769187006	3689295017
Moderate nonproliferative diabetic retinopathy	6022621000006116	312904009	1775724010
Proliferative diabetic retinopathy - non high risk	6022671000006115	312906006	456706019
Left laser treated diabetic retinopathy	856631000006115	856631000006104	856631000006115
Left preproliferative diabetic retinopathy	857051000006118	857051000006102	857051000006118
Right laser treated diabetic retinopathy	857421000006119	857421000006103	857421000006119
Moderate non-proliferative diabetic retinopathy of left eye	12085231000006118	769186002	3689292019
Mild nonproliferative diabetic retinopathy of left eye	12085121000006114	769184004	3689282010
Proliferative diabetic retinopathy - quiescent	6022761000006118	312908007	456708018

Mild non-proliferative diabetic retinopathy of left eye	12085141000006119	769184004	3689278013
Moderate nonproliferative diabetic retinopathy of right eye	12085171000006110	769185003	3689286013
Severe nonproliferative diabetic retinopathy with no macular oedema	6630751000006114	399873008	1773465013
Severe nonproliferative diabetic retinopathy of left eye	12085291000006119	769188001	3689299011
Background diabetic retinopathy	1785332013	390834004	1785332013
O/E - right eye background diabetic retinopathy	2159973010	408409007	2159973010
Diabetic retinopathy 6 month review	2159949019	408385003	2159949019
Very severe nonproliferative diabetic retinopathy	6630851000006115	399876000	1779167017
Impaired vision due to diabetic retinopathy	733161000000116	373041000000101	733161000000116
On examination - left eye preproliferative diabetic retinopathy	6761841000006110	408412005	2621611012
O/E - right eye stable treated proliferative diabetic retinopathy	975251000006111	414910007	2532963013
On examination - left eye proliferative diabetic retinopathy	6761881000006116	408414006	2621613010
On examination - left eye background diabetic retinopathy	6761801000006113	408410002	2621800018
PPDR - Preproliferative diabetic retinopathy	4771421000006116	193349004	2579151010
Diabetic retinopathy detected by national screening programme	8242331000006114	775841000000109	1733071000000112
Preproliferative diabetic retinopathy	297754014	193349004	297754014
Diabetic retinopathy NOS	297758012	4855003	9093013
Laser treated diabetic retinopathy	856611000006114	856611000006105	856611000006114
O/E - left eye background diabetic retinopathy	2159974016	408410002	2159974016
O/E - right eye preproliferative diabetic retinopathy	2159975015	408411003	2159975015
NPDR - Non proliferative diabetic retinopathy	6515201000006111	390834004	2579521014
BDR - Background diabetic retinopathy	6515211000006114	390834004	2621399015
Moderate non-proliferative diabetic retinopathy of right eye	12085181000006113	769185003	3689284011
Early proliferative diabetic retinopathy	6022691000006119	312906006	1785158019
Right preproliferative diabetic retinopathy	857981000006113	857981000006109	857981000006113
Non proliferative diabetic retinopathy	1484867016	390834004	1484867016
Diabetic retinopathy 12 month review	2159948010	408384004	2159948010
O/E - left eye preproliferative diabetic retinopathy	2159976019	408412005	2159976019
Mild non-proliferative diabetic retinopathy of right eye	12085091000006110	769183005	3689273016
Mild non-proliferative diabetic retinopathy	6022581000006116	312903003	2642672017
PDR - proliferative diabetic retinopathy	12189891000006110	59276001	3699415016
On examination - right eye proliferative diabetic retinopathy	6761861000006114	408413000	2621612017
Preproliferative diabetic retinopathy of right eye	12085021000006113	769181007	3689265013
Severe nonproliferative diabetic retinopathy	841011000006112	312905005	1775725011
Moderate non-proliferative diabetic retinopathy	12224731000006112	312904009	3688869017
Diabetic retinopathy	9093013	4855003	9093013

Table S54. Retinal vascular occlusion Aurum codes

Term	Medcode ID	SNOMED CT Concept ID	SNOMED CT Description ID
BRVO - Branch retinal vein occlusion	2892671000006112	24596005	481962015
Branch retinal vein occlusion	481961010	24596005	481961010
Central retinal vein occlusion with macular oedema	5031001000006113	232039004	347681015
Central retinal artery occlusion	64469014	38742007	64469014
Central retinal vein occlusion	113737013	68478007	113737013
BRAO - Branch retinal artery occlusion	3323131000006114	50821009	1230907012
Branch retinal vein occlusion with macular oedema	5031181000006115	232048009	347698015
Hemicentral retinal vein occlusion	5031091000006118	232043000	2619565019
Central retinal vein occlusion - juvenile with macular oedema	5031061000006114	232042005	347686013
Cilioretinal artery occlusion	5030961000006116	232036006	347676012
Hemispheric retinal vein occlusion with macular oedema	5031131000006116	232045007	347692019
Branch retinal artery occlusion	1230906015	50821009	1230906015
Central retinal vein occlusion - ischaemic	6024101000006114	312997008	456819013
Central retinal vein occlusion - non-ischaemic	6024121000006116	312998003	456822010
Central retinal vein occlusion with neovascularisation	5030981000006114	232038007	347678013
Macular branch retinal vein occlusion	5239581000006115	247121001	369049012
Branch retinal vein occlusion with no neovascularisation	6041221000006114	314000002	458269018
Central retinal vein occlusion - ischemic	6024111000006112	312997008	456820019
Retinal artery occlusion	299476010	232035005	347674010
Retinal vein occlusion	3244281000006112	46085004	494432015
Hemispheric retinal vein occlusion	5031081000006116	232043000	347688014
CRAO - Central retinal artery occlusion	3123581000006112	38742007	1229456016
CRVO - Central retinal vein occlusion	3614321000006112	68478007	501027019

Table S55. Glaucoma Aurum codes

Term	Medcode ID	SNOMED CT Concept ID	SNOMED CT Description ID
COAG - Chronic open-angle glaucoma	3753261000006114	77075001	503455016
Secondary angle-closure glaucoma with pupil block	4772381000006115	193561006	298063016
Primary angle-closure glaucoma residual stage	298048019	193546005	298048019
Steroid-induced glaucoma glaucomatous stage	298050010	193548006	298050010
Glaucoma due to ocular cyst	411465018	275477002	411465018
Chronic angle-closure glaucoma	3041311000006118	33647009	485587011
Secondary glaucoma	298053012	95717004	158563019
Family history: Glaucoma	4530051000006112	160347007	2666122010
Glaucoma associated with ocular trauma	3610251000006115	68241007	113353013
Borderline glaucoma NOS	298033013	193531003	298024011
Open-angle glaucoma NOS	298042018	84494001	140102012
Unspecified primary angle-closure glaucoma	298047012	392288006	1486268015
Primary angle-closure glaucoma NOS	298049010	392288006	1486268015
Glaucoma associated with other ocular disorders	298061019	95717004	158563019
Other specified forms of glaucoma	298072012	23986001	40268016
Other specified glaucoma NOS	298074013	23986001	40268016
[X]Other glaucoma	299482013	23986001	40268016
Congenital glaucoma	582671000006113	204113001	312991013
Glaucomatous atrophy of optic disc	2517641000006119	1207009	3144014
Simple chronic glaucoma	141721000006112	77075001	503452018
Corticosteroid-induced glaucoma	2524871000006112	1654001	3874015
Adverse reaction to Drugs Used For Glaucoma	1010721000006116	1010721000006100	1010721000006116
Secondary open-angle glaucoma with pigment dispersion	3245761000006114	46168003	494450010
Phacomorphic glaucoma	6532921000006114	392300000	1486280011
Neovascular glaucoma	347755010	232086000	347755010
Primary angle-closure glaucoma	1486268015	392288006	1486268015
Phacolytic glaucoma	54899010	32893002	54899010
Pigmentary glaucoma	76978010	46168003	76978010
Low tension glaucoma	84105018	50485007	84105018
Glaucoma due to ocular inflammation	803011000006118	37155002	486637019
Intermittent angle-closure glaucoma	3564451000006117	65460003	108757016
Needling of bleb following glaucoma surgery	383421000000110	236891000000101	383421000000110
Chronic angle closure glaucoma	6050201000006111	314784002	1490667012
[X]Glaucoma in endocrine,nutritional+metabolic diseases CE	388101000006114	193556009	298058015
Family history of glaucoma	4530041000006110	160347007	249916014
Phacogenic glaucoma	3872051000006113	84333006	1490002015
Chronic narrow angle glaucoma	3041351000006117	33647009	485589014
Revision of bleb NEC following glaucoma surgery	378061000000112	236891000000101	383421000000110
Other specified operations following glaucoma surgery	378721000000118	792741000000100	1772301000000112

[X]Glaucoma	388091000006115	23986001	40268016
Glaucoma due to ocular tumour or cyst	399447011	267625001	399447011
Pseudoexfoliation glaucoma	178757012	111514006	178757012
Narrow-angle glaucoma	6532591000006118	392291006	2647495010
Glaucoma screening	803131000006113	171215009	265311011
Steroid-induced glaucoma NOS	298052019	1654001	3874015
Glaucoma associated with other ocular disorders NOS	298071017	95717004	158563019
Glaucoma drainage surgery	6353481000006112	371370007	2476699016
Glaucoma due to combination of mechanisms	4010061000006117	92829008	153471016
Open angle glaucoma	3874601000006113	84494001	2579462017
Bilateral angle-closure glaucoma	9846871000006112	15736721000119106	3373331019
Injection of filtering bleb following glaucoma surgery	7089401000006110	428494000	2694522012
Glaucoma due to another disorder	299484014	95717004	158563019
Aphakic glaucoma	2743971000006118	15374009	26072012
Subcapsular glaucomatous flecks	3601481000006119	67733005	112484015
Subacute closed-angle glaucoma	3564481000006113	65460003	1232678013
Acute angle-closure glaucoma of right eye	8107961000006110	336611000119109	3316703019
Glaucoma associated with disorders of the lens	802911000006110	84333006	1234928013
Wide-angle glaucoma	3874591000006117	84494001	140106010
Glaucoma due to other anterior segment anomaly	298056016	95717004	158563019
Glaucoma NOS	298075014	23986001	40268016
Secondary open-angle glaucoma	2848811000006113	21928008	36783018
Glaucoma due to episode of increased venous pressure	802981000006115	34623005	1228189019
Glaucoma due to pupillary block	803061000006115	193561006	298063016
Steroid-induced glaucoma residual stage	298051014	193549003	298051014
Glaucoma with lens disorder	3872071000006115	84333006	1234928013
Uveitic glaucoma	6041601000006118	314033007	458309018
Glaucoma surgery	3899381000006119	86077009	2621280010
Glaucoma suspect	5031661000006115	232079008	347743016
Steroid-induced glaucoma	1221368012	1654001	1221368012
Glaucomatous subcapsular flecks	1232965011	67733005	1232965011
Suspected angle-closure glaucoma	7629711000006119	708176002	3042911012
Glaucoma following surgery	5031801000006115	232090003	347762018
Mixed mechanism glaucoma	4010091000006113	92829008	2770265014
Suspected glaucoma of bilateral eyes caused by steroids	9351591000006115	11001071000119105	3326549013
Chronic closed-angle glaucoma	556201000006114	33647009	485588018
Cupping of optic discs of bilateral eyes co-occurrent and due to open-angle glaucoma	3526208016	737006004	3526208016
Bilateral absolute glaucoma	8111981000006112	347401000119106	3316706010
No family history of glaucoma	4528321000006118	160267000	249716018
Borderline glaucoma steroid responder	1490617017	302895007	1490617017

ACG - Angle closure glaucoma	6532581000006116	392291006	2621430019
Residual stage of open angle glaucoma	47081000006113	66990007	111276019
Secondary/other glaucoma	883301000006118	660261000000103	883301000006118
Angle-closure glaucoma of both eyes	9846911000006110	15736721000119106	3373334010
Angle-closure glaucoma	6532571000006119	392291006	1486271011
Glaucoma right eye	856591000006115	856591000006104	856591000006115
Glaucoma left eye	856601000006111	856601000006107	856601000006111
Borderline glaucoma	298024011	193531003	298024011
Glaucoma due to chamber angle anomaly	298054018	193552006	298054018
Glaucoma due to iris anomaly	298055017	193553001	298055017
Operation following glaucoma surgery	361511000000118	792741000000100	1772301000000112
Glaucoma caused by silicone oil	7727931000006113	715144004	3301594012
Primary open angle glaucoma	3753211000006111	77075001	127956010
Secondary open-angle glaucoma with pseudoexfoliation	4196741000006113	111514006	1219666018
Secondary angle-closure glaucoma	2843081000006117	21571006	36185019
Closed angle glaucoma	560611000006117	392288006	1486268015
Newborn glaucoma	674741000006115	204113001	312991013
Narrow angle glaucoma	6532601000006114	392291006	2647496011
Glaucoma tube shunt	5484711000006110	265291005	2551168012
Absolute glaucoma	802901000006112	19144002	32262013
Glaucoma due to disease NOS	298059011	95717004	158563019
Operations following glaucoma surgery NOS	383401000000118	792741000000100	1772301000000112
Removal of releasable suture following glaucoma surgery	2676361011	426877004	2676361011
Glaucoma associated with disorders of the lens NOS	298060018	84333006	139867011
Needling of bleb following glaucoma surgery	7089411000006113	428494000	3032787012
Acute-on-chronic glaucoma	6041451000006116	314017009	458290014
Angle-closure glaucoma - borderline	521031000006117	193534006	298027016
Glaucoma monitoring	264949013	170946006	264949013
Unspecified open-angle glaucoma	298034019	84494001	140102012
Glaucoma due to unspecified ocular disorder	298062014	95717004	158563019
Glaucomatous optic atrophy	2517661000006115	1207009	1220515013
Primary closed-angle glaucoma	883291000006119	392288006	883291000006119
At risk of glaucoma	1697781000006112	314018004	458291013
Injection of bleb following glaucoma surgery	383381000000118	428494000	1659491000000113
Traumatic glaucoma	3610261000006118	68241007	1233025018
Chronic simple glaucoma	3753221000006115	77075001	127957018
Glaucomatocyclitic crisis	2974881000006116	29538005	49421018
Glaucoma due to systemic syndrome	298057013	193555008	298057013
Glaucoma in endocrine, nutritional and metabolic diseases	298058015	193556009	298058015
Open-angle glaucoma	140102012	84494001	140102012
Narrow angle glaucoma of bilateral eyes	9846901000006112	15736721000119106	3373333016

Glaucoma surgery	6979851000006114	421885009	2618478010
Glaucoma	40268016	23986001	40268016
Hypersecretion glaucoma	49139016	29369005	49139016
Primary congenital glaucoma	6871531000006110	415176004	2535441016
Laser suture lysis following glaucoma surgery	378701000000110	234611000000100	378701000000110
Suspected glaucoma	558691000000111	232079008	2408401000000115
Advanced open-angle glaucoma	4196691000006111	111513000	178756015
Panretinal photocoagulation for glaucoma	249331000006112	172586003	267176015
CSG - Chronic simple glaucoma	3753251000006112	77075001	503454017
POAG - Primary open-angle glaucoma	3753241000006110	77075001	503453011
Low tension glaucoma	734071000006115	50485007	84105018
Suspected glaucoma	1629641000006111	1629641000006107	1629641000006111
Glaucoma implant surgery	5484721000006119	265291005	2883614019
CNAG - Chronic narrow angle glaucoma	3041361000006115	33647009	2620650018
Referral to glaucoma clinic	2533408017	415273001	2533408017
[V]Screening for glaucoma	461299019	171215009	265311011
Bilateral narrow angle glaucoma	9846881000006110	15736721000119106	3373330018
Unspecified preglaucoma	298025012	23986001	40268016
Primary open-angle glaucoma	503452018	77075001	503452018
Optic disc glaucomatous atrophy	1220514012	1207009	1220514012
Glaucoma due to ocular trauma	1233026017	68241007	1233026017
Open-angle glaucoma - borderline	264071000006115	193533000	298026013
Acute closed-angle glaucoma	459721000006110	30041005	1226810019
No evidence of glaucoma	12624091000006111	1109141000000102	2775491000000119
Acute angle-closure glaucoma	2983571000006113	30041005	50268012
Normal pressure glaucoma	495680018	50485007	495680018
AACG - Acute angle closure glaucoma	2983591000006114	30041005	2620418016
Glaucoma micro-stent	3532561018	738510009	3532561018
Glaucomatocyclitic crises	1226368013	29538005	1226368013
Glaucoma with ocular inflammation	3098461000006112	37155002	486637019
Angle recession glaucoma	6535011000006114	392352004	1486331015
[RFC] Glaucoma	907841000006110	907841000006106	907841000006110
Intermittent primary angle-closure glaucoma	770481000006118	65460003	1232679017
Cupping of optic disc co-occurrent and due to open angle glaucoma	3526206017	736838001	3526206017
Glaucoma due to ocular vascular disorder	298064010	193562004	298064010
H/O: glaucoma	251653013	161488001	251653013
Normal tension glaucoma	3317631000006118	50485007	495682014
Open angle glaucoma suspect	14154791000006118		
Angle closure glaucoma suspect	14153851000006115		
Glaucoma suspect	13933811000006119		

Table S56. Age-related macular degeneration Aurum codes

Term	Medcode ID	SNOMED CT Concept ID	SNOMED CT Description ID
Myopic macular degeneration	6022521000006115	312898002	456698016
Drusen stage macular degeneration	5478331000006117	264633009	393115018
Disciform macular degeneration	6853451000006118	414173003	2538082010
Atrophic age-related macular degeneration	6866121000006116	414875008	2537363011
Senile macular degeneration	399676018	267718000	399676018
EMD - Exudative macular degeneration	6853541000006119	414173003	2621806012
Age related macular degeneration	78571000006118	267718000	399670012
AAMD - Age related macular degeneration	5501831000006117	267718000	2620215016
Neovascular age-related macular degeneration	6853501000006116	414173003	2538087016
ARMD - Age-related macular degeneration	5501781000006114	267718000	399672016
Dry senile macular degeneration	2537365016	414875008	2537365016
Wet senile macular degeneration	2538088014	414173003	2538088014
AMD - Age-related macular degeneration	5501811000006111	267718000	399675019
Nonexudative age-related macular degeneration	6866111000006112	414875008	2534426012
Exudative age-related macular degeneration	6853441000006115	414173003	2535500012
Age-related macular degeneration	5501771000006111	267718000	399671011
Disciform macular degeneration	14198731000006115		

Table S57. Retinal Laser Aurum codes

Term	Medcode ID	SNOMED CT Concept ID	SNOMED CT Description ID
Panretinal laser photocoagulation to lesion of retina NEC	361631000000110	283851000000107	489321000000113
Panretinal laser photocoagulation	6020151000006115	312713003	1785150014
Retinal laser photocoagulation	6630611000006117	399867007	1787015019
Focal retinal laser photocoagulation	6588671000006112	397538008	1785845018
Panretinal laser photocoagulation to lesion of retina	489321000000113	283851000000107	489321000000113
O/E - No retinal laser photocoagulation scars	2534106015	414902006	2534106015
Scatter retinal laser photocoagulation	6020161000006118	312713003	2536171014
Retinal laser therapy	262909018	169425004	262911010

Table S58. Vitrectomy Aurum codes

Term	Medcode ID	SNOMED CT Concept ID	SNOMED CT Description ID
Mechanical vitrectomy by pars plana approach	3914871000006116	87021001	144315019
Pars plana vitrectomy	507373018	87021001	507373018
Vitrectomy	3731361000006116	75732000	125783018
Anterior vitrectomy	267129012	172555009	267129012
Vitrectomy using pars plana approach	465551000000114	87021001	465551000000114
PPV - Pars plana vitrectomy	3914891000006115	87021001	507374012
Anterior vitrectomy	486601000006113	172555009	267129012
Vitrectomy by anterior approach	1541981000006119	172555009	267130019
Phacoemulsification/vitrectomy system	7406901000006117	462940007	2944713011

Table S59. Scleral buckle Aurum codes

Term	Medcode ID	SNOMED CT Concept ID	SNOMED CT Description ID
Scleral buckle	6346771000006113	370944007	2673018011
Removal of scleral buckle	6353851000006118	371394007	1210056018
Extrusion of scleral buckle	6516171000006117	390909000	1484941010
Scleral buckle procedure	2929071000006111	26786002	2162369019

Table S60. Intravitreal injection Aurum codes

Term	Medcode ID	SNOMED CT Concept ID	SNOMED CT Description ID
Ranibizumab 10mg/mL solution for intravitreal injection 0.05mL vial	7021931000006119	424204000	2645710015
Intravitreal injection	8255441000006119	800481000000101	1789871000000114

Table S61. Antiplatelet Aurum product codes

Term from EMIS	Prod code ID	dmd ID	Drug substance name
Angettes 75 tablets (Bristol-Myers Squibb Pharmaceuticals Ltd)	71241000033119	800511000001106	Aspirin
Aspirin 300mg dispersible tablets	82841000033117	39695211000001102	Aspirin
Aspirin 75mg dispersible tablets	82941000033113	319773006	Aspirin
Aspirin 75mg gastro-resistant tablets	83041000033115	319781007	Aspirin
Aspirin 300mg gastro-resistant tablets	83141000033116	322225002	Aspirin
Aspirin 300mg modified-release tablets	84441000033114	4558011000001103	Aspirin
Aspirin 300mg tablets	86641000033119	329525004	Aspirin
Aspav dispersible tablets (Actavis UK Ltd)	86841000033118	3690911000001103	Aspirin/ Papaveretum
Aspirin 75mg tablets	87041000033110	319775004	Aspirin
Caprin 75mg gastro-resistant tablets (Wockhardt UK Ltd)	189841000033119	927411000001108	Aspirin
Caprin 300mg gastro-resistant tablets (Pinewood Healthcare)	212341000033117	901211000001107	Aspirin
Co-codaprin 8mg/400mg tablets	370941000033119	11762111000001100	Aspirin/ Codeine phosphate
Co-codaprin 8mg/400mg dispersible tablets	371041000033112	39695111000001108	Aspirin/ Codeine phosphate
Disprin CV 300mg modified-release tablets (Reckitt Benckiser Healthcare (UK) Ltd)	452541000033115	4532311000001104	Aspirin
Nu-Seals 75 gastro-resistant tablets (Alliance Pharmaceuticals Ltd)	991441000033115	414311000001102	Aspirin
Nu-Seals 300 gastro-resistant tablets (Alliance Pharmaceuticals Ltd)	993341000033113	183311000001101	Aspirin
PostMI 75 dispersible tablets (Ashbourne Pharmaceuticals Ltd)	1098041000033111	636011000001100	Aspirin
PostMI 75 EC tablets (Ashbourne Pharmaceuticals Ltd)	1101541000033119	38411000001101	Aspirin
Asasantin Retard capsules (Boehringer Ingelheim Ltd)	1571541000033117	3292511000001108	Aspirin/ Dipyridamole
Enprin 75mg gastro-resistant tablets (Galpharm International Ltd)	1729141000033110	473711000001109	Aspirin
Imazin XL tablets (Napp Pharmaceuticals Ltd)	1739541000033116	3460911000001104	Aspirin/ Isosorbide mononitrate
Imazin XL forte tablets (Napp Pharmaceuticals Ltd)	1739641000033115	3461511000001104	Aspirin/ Isosorbide mononitrate
Aspirin 300mg suppositories	2013341000033115	322232006	Aspirin
Aspirin 150mg suppositories	2013441000033114	322233001	Aspirin
Aspirin powder	2085241000033111	5145711000001107	Aspirin
MigraMax oral powder sachets (Zentiva Pharma UK Ltd)	2086841000033117	3637911000001108	Aspirin DL-Lysine/ Metoclopramide hydrochloride
Micropirin 75mg gastro-resistant tablets (Dexcel-Pharma Ltd)	2273741000033118	281311000001104	Aspirin
Dipyridamole 200mg modified-release / Aspirin 25mg capsules	3160741000033110	36069911000001109	Aspirin/ Dipyridamole
Aspirin 75mg / Isosorbide mononitrate 60mg modified-release tablets	3162041000033113	35903111000001101	Aspirin/ Isosorbide mononitrate

Aspirin 150mg / Isosorbide mononitrate 60mg modified-release tablets	3162141000033112	35903011000001102	Aspirin/ Isosorbide mononitrate
Aspirin 900mg / Metoclopramide 10mg oral powder sachets sugar free	3836841000033119	322784004	Aspirin DL-Lysine/ Metoclopramide hydrochloride
Aspirin 500mg / Papaveretum 7.71mg dispersible tablets sugar free	3849841000033110	39695311000001105	Aspirin/ Papaveretum
Aspirin 500mg / Codeine 8mg dispersible tablets sugar free	3934341000033112	4956111000001107	Aspirin/ Codeine phosphate
Codis 500 dispersible tablets (Reckitt Benckiser Healthcare (UK) Ltd)	3934441000033118	4951811000001105	Aspirin/ Codeine phosphate
Aspro Clear 300mg effervescent tablets (Bayer Plc)	4431841000033118	4649111000001101	Aspirin
Maximum Strength Aspro Clear 500mg effervescent tablets (Bayer Plc)	4431941000033114	4648711000001109	Aspirin
Molita 200mg/25mg modified-release capsules (Dr Reddy's Laboratories (UK) Ltd)	8619141000033116	23241511000001106	Aspirin/ Dipyridamole
Bisoprolol 5mg / Aspirin 75mg capsules	10024941000033116	28365711000001103	Aspirin/ Bisoprolol fumarate
Bisoprolol 5mg / Aspirin 100mg capsules	10025041000033116	28365611000001107	Aspirin/ Bisoprolol fumarate
Bisoprolol 10mg / Aspirin 100mg capsules	10025241000033112	28365311000001102	Aspirin/ Bisoprolol fumarate
Danamep 75mg dispersible tablets (Ecogen Europe Ltd)	11716941000033112	32968711000001107	Aspirin
Clopidogrel 75mg tablets	1583541000033113	39689111000001106	Clopidogrel
Plavix 75mg tablets (Sanofi)	1667041000033119	454611000001104	Clopidogrel
Clopidogrel 300mg tablets	4519641000033119	429540001	Clopidogrel hydrogen sulfate
Plavix 300mg tablets (Sanofi)	4519741000033111	13663511000001101	Clopidogrel hydrogen sulfate
Grepid 75mg tablets (Kent Pharma (UK) Ltd)	5242341000033112	15907411000001101	Clopidogrel
Clopidogrel 75mg/5ml oral suspension	6122841000033118	8426611000001103	Clopidogrel hydrogen sulfate
Clopidogrel 1mg/ml oral suspension	8031441000033112	18747911000001101	Clopidogrel hydrogen sulfate
Clopidogrel 4mg oral powder sachets	8300741000033118	22342711000001109	Clopidogrel hydrogen sulfate
Clopidogrel 75mg/5ml oral solution	8301241000033117	22402511000001104	Clopidogrel hydrogen sulfate
Clopidogrel 25mg/5ml oral suspension	13283841000033111	16072911000001108	Clopidogrel hydrogen sulfate
Ticagrelor 90mg tablets	6059341000033111	704465002	Ticagrelor
Brilique 90mg tablets (AstraZeneca UK Ltd)	6059441000033117	18290311000001104	Ticagrelor
Ticagrelor 60mg tablets	11422441000033119	32472211000001106	Ticagrelor
Brilique 60mg tablets (AstraZeneca UK Ltd)	11422741000033114	32447511000001106	Ticagrelor
Ticagrelor 90mg orodispersible tablets sugar free	12371041000033112	34713411000001108	Ticagrelor
Brilique 90mg orodispersible tablets (AstraZeneca UK Ltd)	12371141000033111	34672011000001105	Ticagrelor
Dipyridamole 10mg/2ml solution for injection ampoules	447141000033112	36069811000001104	Dipyridamole
Dipyridamole 200mg modified-release capsules	454141000033119	39020811000001108	Dipyridamole
Dipyridamole 100mg tablets	469641000033119	319759002	Dipyridamole

Dipyridamole 25mg tablets	469741000033111	319758005	Dipyridamole
Persantin 10mg/2ml solution for injection ampoules (Boehringer Ingelheim Ltd)	1052141000033119	642111000001104	Dipyridamole
Persantin Retard 200mg capsules (Boehringer Ingelheim Ltd)	1054941000033114	452911000001101	Dipyridamole
Persantin 100mg tablets (Boehringer Ingelheim Ltd)	1065441000033113	623011000001106	Dipyridamole
Persantin 25mg tablets (Boehringer Ingelheim Ltd)	1065541000033114	635611000001102	Dipyridamole
Dipyridamole 50mg/5ml oral suspension sugar free	1728341000033118	36070011000001108	Dipyridamole
Dipyridamole 200mg/5ml oral suspension	5020341000033117	8456311000001107	Dipyridamole
Dipyridamole 100mg/5ml oral suspension	5052341000033115	8456511000001101	Dipyridamole
Dipyridamole 250mg/5ml oral solution	5401541000033119	8456211000001104	Dipyridamole
Dipyridamole 100mg/5ml oral solution	5889941000033115	8456611000001102	Dipyridamole
Dipyridamole 200mg/5ml oral solution	5992041000033118	8456411000001100	Dipyridamole
Attia 200mg modified-release capsules (Dr Reddy's Laboratories (UK) Ltd)	9165341000033117	24435511000001109	Dipyridamole
Ofcram PR 200mg capsules (Advanz Pharma)	10333041000033118	26779011000001104	Dipyridamole
Dipyridamole 200mg/5ml oral suspension sugar free	11072641000033113	31948811000001109	Dipyridamole
Trolactin 200mg modified-release capsules (Actavis UK Ltd)	12195441000033115	34175911000001100	Dipyridamole
Prasugrel 5mg tablets	4956441000033110	39702911000001108	Prasugrel
Prasugrel 10mg tablets	4956541000033111	39702811000001103	Prasugrel
Efient 5mg tablets (Daiichi Sankyo UK Ltd)	4956641000033112	15241111000001105	Prasugrel
Efient 10mg tablets (Daiichi Sankyo UK Ltd)	4956741000033115	15240211000001105	Prasugrel

Table S62. Thiazide diuretics Aurum product codes

Term from EMIS	Prod code ID	dmd ID	Drug substance name
Accuretic 10mg/12.5mg tablets (Pfizer Ltd)	11241000033113	260211000001104	Hydrochlorothiazide/ Quinapril hydrochloride
Acezide 25mg/50mg tablets (Bristol-Myers Squibb Pharmaceuticals Ltd)	13141000033119	263311000001101	Captopril/ Hydrochlorothiazide
Amilmaxco 5mg/50mg tablets (Ashbourne Pharmaceuticals Ltd)	57341000033115	712211000001108	Amiloride hydrochloride/ Hydrochlorothiazide
Amil-Co 5mg/50mg tablets (IVAX Pharmaceuticals UK Ltd)	60341000033110	636611000001107	Amiloride hydrochloride/ Hydrochlorothiazide
Capozide LS 12.5mg/25mg tablets (Bristol-Myers Squibb Pharmaceuticals Ltd)	209241000033119	546711000001107	Captopril/ Hydrochlorothiazide
Carace 10 Plus tablets (Organon Pharma (UK) Ltd)	209841000033115	3144511000001107	Hydrochlorothiazide/ Lisinopril
Capozide 25mg/50mg tablets (Bristol-Myers Squibb Pharmaceuticals Ltd)	216441000033110	17311000001102	Captopril/ Hydrochlorothiazide
Carace 20 Plus tablets (Organon Pharma (UK) Ltd)	217041000033116	3143511000001109	Hydrochlorothiazide/ Lisinopril
Co-amilozide 5mg/50mg/5ml oral solution	351941000033111	8792311000001109	Amiloride hydrochloride/ Hydrochlorothiazide
Co-amilozide 5mg/50mg tablets	369141000033118	377566005	Amiloride hydrochloride/ Hydrochlorothiazide
Co-triamterzide 50mg/25mg tablets	370241000033111	410896007	Hydrochlorothiazide/ Triamterene
Cozaar-Comp 50mg/12.5mg tablets (Organon Pharma (UK) Ltd)	370841000033110	255911000001105	Hydrochlorothiazide/ Losartan potassium
Co-Betaloc tablets (Pfizer Ltd)	372741000033116	2977611000001106	Hydrochlorothiazide/ Metoprolol tartrate
Co-Betaloc SA tablets (Pfizer Ltd)	372841000033114	3853411000001104	Hydrochlorothiazide/ Metoprolol tartrate
Co-amilozide 2.5mg/25mg tablets	376941000033117	318121006	Amiloride hydrochloride/ Hydrochlorothiazide
Dyazide 50mg/25mg tablets (Advanz Pharma)	492341000033115	132811000001106	Hydrochlorothiazide/ Triamterene
Hydrosaluric 25mg tablets (Organon Pharma (UK) Ltd)	742541000033112	4544011000001109	Hydrochlorothiazide
Hydrosaluric 50mg tablets (Organon Pharma (UK) Ltd)	742641000033113	4546211000001109	Hydrochlorothiazide
Hydrochlorothiazide 25mg tablets	744941000033110	376209006	Hydrochlorothiazide
Hydrochlorothiazide 50mg tablets	745441000033118	376508004	Hydrochlorothiazide
Innozide 20mg/12.5mg tablets (Organon Pharma (UK) Ltd)	770141000033115	146811000001108	Enalapril maleate/ Hydrochlorothiazide
Kalten capsules (M & A Pharmachem Ltd)	793741000033117	237011000001100	Amiloride hydrochloride/ Atenolol/ Hydrochlorothiazide
Moducren tablets (Organon Pharma (UK) Ltd)	935341000033118	74311000001101	Amiloride hydrochloride/ Hydrochlorothiazide/ Timolol maleate
Moduret 25 tablets (Organon Pharma (UK) Ltd)	935441000033112	314211000001100	Amiloride hydrochloride/ Hydrochlorothiazide

Moduretic 5mg/50mg tablets (Merck Sharp & Dohme (UK) Ltd)	935541000033113	453811000001103	Amiloride hydrochloride/ Hydrochlorothiazide
Monozide 10 tablets (Wyeth Pharmaceuticals)	937341000033111	4542911000001104	Bisoprolol fumarate/ Hydrochlorothiazide
Secadrex 200mg/12.5mg tablets (Sanofi)	1274641000033111	878911000001106	Acebutolol hydrochloride/ Hydrochlorothiazide
Triamaxco 50mg/25mg tablets (Ashbourne Pharmaceuticals Ltd)	1468141000033118	721311000001104	Hydrochlorothiazide/ Triamterene
Triam-Co 50mg/25mg tablets (IVAX Pharmaceuticals UK Ltd)	1470341000033112	191211000001106	Hydrochlorothiazide/ Triamterene
Zestoretic 10 tablets (AstraZeneca UK Ltd)	1551841000033118	3144311000001101	Hydrochlorothiazide/ Lisinopril
Zestoretic 20 tablets (AstraZeneca UK Ltd)	1552341000033118	3143111000001100	Hydrochlorothiazide/ Lisinopril
Co-zidocapt 12.5mg/25mg tablets	1893141000033119	318806002	Captopril/ Hydrochlorothiazide
Co-zidocapt 25mg/50mg tablets	1893241000033114	318807006	Captopril/ Hydrochlorothiazide
CoAprovel 150mg/12.5mg tablets (Sanofi)	2216141000033115	792411000001108	Hydrochlorothiazide/ Irbesartan
CoAprovel 300mg/12.5mg tablets (Sanofi)	2216241000033110	682711000001109	Hydrochlorothiazide/ Irbesartan
Hydrochlorothiazide 50mg/5ml oral solution	2746441000033111	8529611000001101	Hydrochlorothiazide
MicardisPlus 40mg/12.5mg tablets (Boehringer Ingelheim Ltd)	2864241000033110	3806311000001109	Hydrochlorothiazide/ Telmisartan
MicardisPlus 80mg/12.5mg tablets (Boehringer Ingelheim Ltd)	2864341000033117	3806911000001105	Hydrochlorothiazide/ Telmisartan
Caralpha 10mg/12.5mg tablets (Actavis UK Ltd)	3136041000033117	7385911000001108	Hydrochlorothiazide/ Lisinopril
Caralpha 20mg/12.5mg tablets (Actavis UK Ltd)	3136141000033118	7385711000001106	Hydrochlorothiazide/ Lisinopril
Co-Diovan 160mg/12.5mg tablets (Novartis Pharmaceuticals UK Ltd)	3152541000033112	7668611000001104	Hydrochlorothiazide/ Valsartan
Co-Diovan 160mg/25mg tablets (Novartis Pharmaceuticals UK Ltd)	3152641000033113	7668911000001105	Hydrochlorothiazide/ Valsartan
Quinapril 10mg / Hydrochlorothiazide 12.5mg tablets	3160641000033118	39693411000001102	Hydrochlorothiazide/ Quinapril hydrochloride
Lisinopril 10mg / Hydrochlorothiazide 12.5mg tablets	3160841000033117	318884002	Hydrochlorothiazide/ Lisinopril
Lisinopril 20mg / Hydrochlorothiazide 12.5mg tablets	3160941000033113	318880006	Hydrochlorothiazide/ Lisinopril
Irbesartan 150mg / Hydrochlorothiazide 12.5mg tablets	3161141000033116	134461004	Hydrochlorothiazide/ Irbesartan
Irbesartan 300mg / Hydrochlorothiazide 12.5mg tablets	3161241000033111	134460003	Hydrochlorothiazide/ Irbesartan
Metoprolol 100mg / Hydrochlorothiazide 12.5mg tablets	3161341000033118	318546001	Hydrochlorothiazide/ Metoprolol tartrate
Valsartan 160mg / Hydrochlorothiazide 12.5mg tablets	3161441000033112	395521005	Hydrochlorothiazide/ Valsartan
Valsartan 160mg / Hydrochlorothiazide 25mg tablets	3161541000033113	409298002	Hydrochlorothiazide/ Valsartan
Losartan 50mg / Hydrochlorothiazide 12.5mg tablets	3161741000033117	318959004	Hydrochlorothiazide/ Losartan potassium
Enalapril 20mg / Hydrochlorothiazide 12.5mg tablets	3162241000033117	39691911000001109	Enalapril maleate/ Hydrochlorothiazide
Telmisartan 40mg / Hydrochlorothiazide 12.5mg tablets	3162641000033119	407855002	Hydrochlorothiazide/ Telmisartan
Telmisartan 80mg / Hydrochlorothiazide 12.5mg tablets	3162741000033111	407856001	Hydrochlorothiazide/ Telmisartan

Acebutolol 200mg / Hydrochlorothiazide 12.5mg tablets	3162941000033114	39725011000001105	Acebutolol hydrochloride/ Hydrochlorothiazide
Valsartan 80mg / Hydrochlorothiazide 12.5mg tablets	3190741000033112	377488008	Hydrochlorothiazide/ Valsartan
Co-Diovan 80mg/12.5mg tablets (Novartis Pharmaceuticals UK Ltd)	3190841000033119	8150111000001108	Hydrochlorothiazide/ Valsartan
Lisicostad 10mg/12.5mg tablets (Genus Pharmaceuticals Ltd)	3279541000033115	8145711000001102	Hydrochlorothiazide/ Lisinopril
Lisicostad 20mg/12.5mg tablets (Genus Pharmaceuticals Ltd)	3279641000033119	8145911000001100	Hydrochlorothiazide/ Lisinopril
Losartan 100mg / Hydrochlorothiazide 25mg tablets	3346841000033117	395497004	Hydrochlorothiazide/ Losartan potassium
Cozaar-Comp 100mg/25mg tablets (Organon Pharma (UK) Ltd)	3346941000033113	9566911000001105	Hydrochlorothiazide/ Losartan potassium
Olmesartan medoxomil 20mg / Hydrochlorothiazide 12.5mg tablets	3908941000033112	409184002	Hydrochlorothiazide/ Olmesartan medoxomil
Olmesartan medoxomil 20mg / Hydrochlorothiazide 25mg tablets	3909041000033115	10270711000001105	Hydrochlorothiazide/ Olmesartan medoxomil
Olmetec Plus 20mg/12.5mg tablets (Daiichi Sankyo UK Ltd)	3909141000033116	10261511000001103	Hydrochlorothiazide/ Olmesartan medoxomil
Olmetec Plus 20mg/25mg tablets (Daiichi Sankyo UK Ltd)	3909241000033111	10261811000001100	Hydrochlorothiazide/ Olmesartan medoxomil
Irbesartan 300mg / Hydrochlorothiazide 25mg tablets	3995241000033112	10970311000001105	Hydrochlorothiazide/ Irbesartan
CoAprovel 300mg/25mg tablets (Sanofi)	3995341000033119	10968611000001106	Hydrochlorothiazide/ Irbesartan
Losartan 100mg / Hydrochlorothiazide 12.5mg tablets	4424741000033110	13112711000001103	Hydrochlorothiazide/ Losartan potassium
Cozaar-Comp 100mg/12.5mg tablets (Organon Pharma (UK) Ltd)	4424841000033117	13094111000001102	Hydrochlorothiazide/ Losartan potassium
Telmisartan 80mg / Hydrochlorothiazide 25mg tablets	4548741000033111	13731911000001109	Hydrochlorothiazide/ Telmisartan
MicardisPlus 80mg/25mg tablets (Boehringer Ingelheim Ltd)	4548841000033118	13719711000001103	Hydrochlorothiazide/ Telmisartan
Timolol 10mg / Amiloride 2.5mg / Hydrochlorothiazide 25mg tablets (Essential Generics Ltd)	5376441000033114	16458411000001107	Amiloride hydrochloride/ Hydrochlorothiazide/ Timolol maleate
Olmesartan medoxomil 40mg / Hydrochlorothiazide 12.5mg tablets	5566341000033111	409185001	Hydrochlorothiazide/ Olmesartan medoxomil
Olmetec Plus 40mg/12.5mg tablets (Daiichi Sankyo UK Ltd)	5566441000033117	17220911000001102	Hydrochlorothiazide/ Olmesartan medoxomil
Hydrochlorothiazide 5mg/5ml oral solution	5969841000033119	12538711000001107	Hydrochlorothiazide
Hydrochlorothiazide 5mg/5ml oral suspension	5969941000033110	12538811000001104	Hydrochlorothiazide
Sevikar HCT 20mg/5mg/12.5mg tablets (Daiichi Sankyo UK Ltd)	6180641000033112	18986411000001108	Amlodipine besilate/ Hydrochlorothiazide/ Olmesartan medoxomil
Sevikar HCT 40mg/5mg/12.5mg tablets (Daiichi Sankyo UK Ltd)	6180741000033115	18986711000001102	Amlodipine besilate/ Hydrochlorothiazide/ Olmesartan medoxomil
Sevikar HCT 40mg/10mg/12.5mg tablets (Daiichi Sankyo UK Ltd)	6180841000033113	18987011000001101	Amlodipine besilate/ Hydrochlorothiazide/

			Olmesartan medoxomil
Sevikar HCT 40mg/5mg/25mg tablets (Daiichi Sankyo UK Ltd)	6180941000033117	18987311000001103	Amlodipine besilate/ Hydrochlorothiazide/ Olmesartan medoxomil
Sevikar HCT 40mg/10mg/25mg tablets (Daiichi Sankyo UK Ltd)	6181041000033110	18987611000001108	Amlodipine besilate/ Hydrochlorothiazide/ Olmesartan medoxomil
Actelsar HCT 40mg/12.5mg tablets (Actavis UK Ltd)	9159341000033112	24411411000001100	Hydrochlorothiazide/ Telmisartan
Actelsar HCT 80mg/12.5mg tablets (Actavis UK Ltd)	9159441000033118	24412511000001109	Hydrochlorothiazide/ Telmisartan
Actelsar HCT 80mg/25mg tablets (Accord Healthcare Ltd)	9159541000033117	24413611000001103	Hydrochlorothiazide/ Telmisartan
Tolucombi 40mg/12.5mg tablets (Consilient Health Ltd)	9292641000033116	24573211000001101	Hydrochlorothiazide/ Telmisartan
Tolucombi 80mg/12.5mg tablets (Consilient Health Ltd)	9292741000033113	24573411000001102	Hydrochlorothiazide/ Telmisartan
Lisoretic 10mg/12.5mg tablets (Bristol Laboratories Ltd)	13443441000033115	38240211000001100	Hydrochlorothiazide/ Lisinopril
Lisoretic 20mg/12.5mg tablets (Bristol Laboratories Ltd)	13443541000033119	38240011000001105	Hydrochlorothiazide/ Lisinopril
Chlorothiazide 250mg/5ml oral suspension	246241000033116	408039005	Chlorothiazide
Chlorothiazide 200mg/5ml oral suspension	3924141000033111	12502311000001102	Chlorothiazide
Chlorothiazide 250mg tablets	5093641000033110	395516007	Chlorothiazide
Chlorothiazide 150mg/5ml oral suspension	5899341000033118	8358511000001105	Chlorothiazide
Chlorothiazide 25mg/5ml oral suspension	5899441000033112	8358711000001100	Chlorothiazide
Chlorothiazide 250mg/5ml oral solution	5991141000033110	12503011000001109	Chlorothiazide
Spironolactone 4mg / Chlorothiazide 40mg capsules	7681541000033117	18520511000001107	Chlorothiazide/ Spironolactone
Spironolactone 3mg / Chlorothiazide 30mg capsules	9278541000033112	16072811000001103	Chlorothiazide/ Spironolactone
Aprinox 2.5mg tablets (Advanz Pharma)	74541000033119	672111000001100	Bendroflumethiazide
Aprinox 5mg tablets (Amdipharm Plc)	74641000033118	120911000001103	Bendroflumethiazide
Centyl K modified-release tablets (LEO Pharma)	234841000033110	3932711000001105	Bendroflumethiazide/ Potassium chloride
Corgaretic 40mg tablets (Sanofi-Synthelabo Ltd)	375441000033118	3886211000001102	Bendroflumethiazide/ Nadolol
Corgaretic 80mg tablets (Sanofi-Synthelabo Ltd)	375541000033117	4057911000001102	Bendroflumethiazide/ Nadolol
Inderetic 80mg/2.5mg capsules (AstraZeneca UK Ltd)	756641000033114	333111000001104	Bendroflumethiazide/ Propranolol hydrochloride
Inderex 160mg/5mg modified-release capsules (AstraZeneca UK Ltd)	756741000033117	350811000001100	Bendroflumethiazide/ Propranolol hydrochloride
Neo-Naclex 5mg tablets (Mercury Pharma Group Ltd)	965341000033114	817911000001100	Bendroflumethiazide
Neo-Naclex-K modified-release tablets (Mercury Pharma Group Ltd)	967041000033118	3638211000001100	Bendroflumethiazide/ Potassium chloride
Prestim tablets (Meda Pharmaceuticals Ltd)	1132741000033111	98411000001109	Bendroflumethiazide/ Timolol maleate
Tenben 25mg/1.25mg capsules (Galen Ltd)	1414241000033117	721411000001106	Atenolol/ Bendroflumethiazide
Urizide 5mg tablets (Dr Reddy's Laboratories (UK) Ltd)	1495441000033116	898211000001109	Bendroflumethiazide

Urizide 2.5mg tablets (Dr Reddy's Laboratories (UK) Ltd)	2955941000033119	485311000001101	Bendroflumethiazide
Bendroflumethiazide 2.5mg tablets	3083141000033112	317919004	Bendroflumethiazide
Bendroflumethiazide 5mg tablets	3083241000033117	317920005	Bendroflumethiazide
Bendroflumethiazide 2.5mg/5ml oral suspension	3151841000033117	8306811000001101	Bendroflumethiazide
Bendroflumethiazide 2.5mg / Potassium chloride 573mg (potassium 7.7mmol) modified-release tablets	3161041000033115	35910011000001105	Bendroflumethiazide/ Potassium chloride
Bendroflumethiazide 2.5mg / Potassium chloride 630mg (potassium 8.4mmol) modified-release tablets	3162841000033118	35910111000001106	Bendroflumethiazide/ Potassium chloride
Timolol 10mg / Bendroflumethiazide 2.5mg tablets	3346641000033118	318556002	Bendroflumethiazide/ Timolol maleate
Bendroflumethiazide 5mg/5ml oral suspension	5401741000033110	8306711000001109	Bendroflumethiazide
Bendroflumethiazide 1.25mg/5ml oral suspension	5453941000033119	8307011000001105	Bendroflumethiazide
Neo-Naclex 2.5mg tablets (Advanz Pharma)	6045641000033115	18149011000001101	Bendroflumethiazide
Nephril 1mg tablets (Pfizer Ltd)	965441000033115	4549211000001100	Polythiazide
Polythiazide 1mg tablets	1110141000033119	317967009	Polythiazide

Table S63. Thiazide like diuretics Aurum product codes

Term from EMIS	Prod code ID	dmd ID	Drug substance name
Indapamide 1.5mg modified-release tablets	765741000033116	39020711000001100	Indapamide
Indapamide 2.5mg tablets	768641000033118	39696211000001108	Indapamide hemihydrate
Nindaxa 2.5 tablets (Ashbourne Pharmaceuticals Ltd)	769741000033118	424311000001108	Indapamide hemihydrate
Natrilix SR 1.5mg tablets (Servier Laboratories Ltd)	953141000033119	456611000001108	Indapamide
Natrilix 2.5mg tablets (Servier Laboratories Ltd)	955341000033117	321811000001109	Indapamide hemihydrate
Coversyl Plus tablets (Servier Laboratories Ltd)	2739541000033117	562511000001109	Indapamide/ Perindopril erbumine
Perindopril erbumine 4mg / Indapamide 1.25mg tablets	3161641000033114	3437611000001100	Indapamide/ Perindopril erbumine
Perindopril arginine 5mg / Indapamide 1.25mg tablets	4454541000033118	13454311000001101	Indapamide/ Perindopril arginine
Coversyl Arginine Plus 5mg/1.25mg tablets (Servier Laboratories Ltd)	4454641000033117	13444311000001104	Indapamide/ Perindopril arginine
Ethibide XL 1.5mg tablets (Genus Pharmaceuticals Ltd)	4571241000033110	13824811000001102	Indapamide
Tensaid XL 1.5mg tablets (Mylan)	4656741000033119	14242211000001100	Indapamide
Indipam XL 1.5mg tablets (Accord Healthcare Ltd)	5053141000033113	15436111000001104	Indapamide
Mapemid XL 1.5mg tablets (Teva UK Ltd)	5128741000033110	15600711000001107	Indapamide
Rawel XL 1.5mg tablets (Consilient Health Ltd)	5566041000033114	16737911000001105	Indapamide
Varbim XL 1.5mg tablets (Teva UK Ltd)	5816741000033112	14693611000001102	Indapamide
Indapamide 2.5mg/5ml oral suspension	8197041000033112	21578911000001105	Indapamide
Perindopril tosilate 5mg / Indapamide 1.25mg tablets	8263641000033112	21940011000001101	Indapamide/ Perindopril tosilate
Cardide SR 1.5mg tablets (Teva UK Ltd)	9121841000033115	24331611000001104	Indapamide
Alkapamid XL 1.5mg tablets (HBS Healthcare Ltd)	12312641000033118	34494911000001106	Indapamide
Lorvacs XL 1.5mg tablets (Torrent Pharma (UK) Ltd)	12988441000033113	36812711000001103	Indapamide
Diurexan 20mg tablets (Mylan)	470741000033113	348911000001105	Xipamide
Xipamide 20mg tablets	1543941000033111	317970008	Xipamide
Viskaldix tablets (Advanz Pharma)	1523941000033114	3638411000001101	Clopamide/ Pindolol
Pindolol 10mg / Clopamide 5mg tablets	3163141000033117	318552000	Clopamide/ Pindolol

Table S64. Potassium sparing diuretics Aurum product codes

Term from EMIS	Prod code ID	dmd ID	Drug substance name
Amiloride 5mg/5ml oral solution sugar free	53841000033117	35900111000001108	Amiloride hydrochloride
Amiloride 5mg tablets	57141000033118	318052005	Amiloride hydrochloride
Amilmaxco 5mg/50mg tablets (Ashbourne Pharmaceuticals Ltd)	57341000033115	712211000001108	Amiloride hydrochloride/ Hydrochlorothiazide
Amil-Co 5mg/50mg tablets (IVAX Pharmaceuticals UK Ltd)	60341000033110	636611000001107	Amiloride hydrochloride/ Hydrochlorothiazide
Burinex A 5mg/1mg tablets (LEO Pharma)	172341000033112	33911000001104	Amiloride hydrochloride/ Bumetanide
Co-amilozide 5mg/50mg/5ml oral solution	351941000033111	8792311000001109	Amiloride hydrochloride/ Hydrochlorothiazide
Co-amilozide 5mg/50mg tablets	369141000033118	377566005	Amiloride hydrochloride/ Hydrochlorothiazide
Co-amilofruse 5mg/40mg tablets	376641000033112	318136009	Amiloride hydrochloride/ Furosemide
Co-amilofruse 10mg/80mg tablets	376741000033115	318137000	Amiloride hydrochloride/ Furosemide
Co-amilofruse 2.5mg/20mg tablets	376841000033113	318135008	Amiloride hydrochloride/ Furosemide
Co-amilozide 2.5mg/25mg tablets	376941000033117	318121006	Amiloride hydrochloride/ Hydrochlorothiazide
Frumil LS 20mg/2.5mg tablets (Sanofi)	614341000033113	550711000001106	Amiloride hydrochloride/ Furosemide
Frumil 40mg/5mg tablets (Sanofi)	614641000033117	427411000001106	Amiloride hydrochloride/ Furosemide
Fru-Co 5mg/40mg tablets (Teva UK Ltd)	615241000033116	818511000001106	Amiloride hydrochloride/ Furosemide
Frumil Forte 10mg/80mg tablets (Sanofi)	615641000033118	82611000001106	Amiloride hydrochloride/ Furosemide
Kalten capsules (M & A Pharmachem Ltd)	793741000033117	237011000001100	Amiloride hydrochloride/ Atenolol/ Hydrochlorothiazide
Lasoride 5mg/40mg tablets (Sanofi)	818941000033112	678511000001106	Amiloride hydrochloride/ Furosemide
Moducren tablets (Organon Pharma (UK) Ltd)	935341000033118	74311000001101	Amiloride hydrochloride/ Hydrochlorothiazide/ Timolol maleate
Moduret 25 tablets (Organon Pharma (UK) Ltd)	935441000033112	314211000001100	Amiloride hydrochloride/ Hydrochlorothiazide
Moduretic 5mg/50mg tablets (Merck Sharp & Dohme (UK) Ltd)	935541000033113	453811000001103	Amiloride hydrochloride/ Hydrochlorothiazide

Navispare 2.5mg/250microgram tablets (Advanz Pharma)	956741000033114	535711000001100	Amiloride hydrochloride/ Cyclopenthiazide
Froop Co 5mg/40mg tablets (Ashbourne Pharmaceuticals Ltd)	1611441000033117	331311000001103	Amiloride hydrochloride/ Furosemide
Amiloride 5mg / Bumetanide 1mg tablets	1845641000033119	318097001	Amiloride hydrochloride/ Bumetanide
Amiloride 2.5mg / Cyclopenthiazide 250microgram tablets	1903541000033116	318096005	Amiloride hydrochloride/ Cyclopenthiazide
Amilamont 5mg/5ml oral solution sugar free (Rosemont Pharmaceuticals Ltd)	2101641000033119	799711000001108	Amiloride hydrochloride
Amoride 5mg tablets (Dr Reddy's Laboratories (UK) Ltd)	2956041000033112	220711000001102	Amiloride hydrochloride
Timolol 10mg / Amiloride 2.5mg / Hydrochlorothiazide 25mg tablets (Essential Generics Ltd)	5376441000033114	16458411000001107	Amiloride hydrochloride/ Hydrochlorothiazide/ Timolol maleate
Co-amilofruse 5mg/40mg/5ml oral suspension	5710441000033117	8427011000001108	Amiloride hydrochloride/ Furosemide
Co-triamterzide 50mg/25mg tablets	370241000033111	410896007	Hydrochlorothiazide/ Triamterene
Dytac 50mg capsules (Advanz Pharma)	491641000033116	3907911000001100	Triamterene
Dytide capsules (Mercury Pharma Group Ltd)	491741000033113	714911000001108	Benzthiazide/ Triamterene
Dyazide 50mg/25mg tablets (Advanz Pharma)	492341000033115	132811000001106	Hydrochlorothiazide/ Triamterene
Frusene 50mg/40mg tablets (Orion Pharma (UK) Ltd)	615041000033112	25411000001108	Furosemide/ Triamterene
Kalspare tablets (DHP Healthcare Ltd)	796541000033115	3252011000001105	Chlortalidone/ Triamterene
Triamterene 50mg capsules	1455341000033115	318082004	Triamterene
Triamaxco 50mg/25mg tablets (Ashbourne Pharmaceuticals Ltd)	1468141000033118	721311000001104	Hydrochlorothiazide/ Triamterene
Triam-Co 50mg/25mg tablets (IVAX Pharmaceuticals UK Ltd)	1470341000033112	191211000001106	Hydrochlorothiazide/ Triamterene
Triamterene 50mg / Furosemide 40mg tablets	3092741000033119	318101005	Furosemide/ Triamterene
Triamterene 50mg / Benzthiazide 25mg capsules	3161941000033119	318098006	Benzthiazide/ Triamterene
Triamterene 50mg / Chlortalidone 50mg tablets	3162341000033110	318100006	Chlortalidone/ Triamterene

Table S65. Dihydropyridines Aurum product codes

Term from EMIS	Prod code ID	dmd ID	Drug substance name
Istin 5mg tablets (Upjohn UK Ltd)	787941000033112	172711000001100	Amlodipine
Istin 10mg tablets (Upjohn UK Ltd)	788041000033110	408111000001107	Amlodipine
Amlodipine 5mg tablets	3038041000033111	39732011000001102	Amlodipine
Amlodipine 10mg tablets	3038141000033110	39731911000001109	Amlodipine
Amlostin 5mg tablets (Discovery Pharmaceuticals)	3188741000033111	8046211000001107	Amlodipine
Amlostin 10mg tablets (Discovery Pharmaceuticals)	3188841000033118	8046411000001106	Amlodipine
Amlodipine 10mg/5ml oral suspension	3963741000033115	8278111000001105	Amlodipine
Amlodipine 5mg / Valsartan 80mg tablets	4021441000033114	11160311000001109	Amlodipine besilate/ Valsartan
Amlodipine 5mg / Valsartan 160mg tablets	4021541000033110	11160211000001101	Amlodipine besilate/ Valsartan
Amlodipine 10mg / Valsartan 160mg tablets	4021641000033111	11160111000001107	Amlodipine besilate/ Valsartan
Exforge 5mg/80mg tablets (Novartis Pharmaceuticals UK Ltd)	4021741000033119	11161811000001108	Amlodipine besilate/ Valsartan
Exforge 5mg/160mg tablets (Novartis Pharmaceuticals UK Ltd)	4021841000033112	11161511000001105	Amlodipine besilate/ Valsartan
Exforge 10mg/160mg tablets (Novartis Pharmaceuticals UK Ltd)	4021941000033116	11160711000001108	Amlodipine besilate/ Valsartan
Amlodipine 5mg/5ml oral solution sugar free	4508041000033119	29826311000001101	Amlodipine
Amlodipine 1.5mg/5ml oral suspension	4897741000033118	15773511000001108	Amlodipine
Olmesartan medoxomil 20mg / Amlodipine 5mg tablets	5005841000033114	429502004	Amlodipine besilate/ Olmesartan medoxomil
Olmesartan medoxomil 40mg / Amlodipine 5mg tablets	5005941000033118	429503009	Amlodipine besilate/ Olmesartan medoxomil
Olmesartan medoxomil 40mg / Amlodipine 10mg tablets	5006041000033111	429678006	Amlodipine besilate/ Olmesartan medoxomil
Sevikar 20mg/5mg tablets (Daiichi Sankyo UK Ltd)	5006141000033110	15773211000001105	Amlodipine besilate/ Olmesartan medoxomil
Sevikar 40mg/5mg tablets (Daiichi Sankyo UK Ltd)	5006241000033115	15772911000001108	Amlodipine besilate/ Olmesartan medoxomil
Sevikar 40mg/10mg tablets (Daiichi Sankyo UK Ltd)	5006341000033113	15772611000001102	Amlodipine besilate/ Olmesartan medoxomil
Amlodipine 5mg/5ml oral solution	5490241000033113	13892511000001100	Amlodipine
Amlodipine 5mg/5ml oral suspension	5888741000033110	8278311000001107	Amlodipine
Sevikar HCT 20mg/5mg/12.5mg tablets (Daiichi Sankyo UK Ltd)	6180641000033112	18986411000001108	Amlodipine besilate/ Hydrochlorothiazide/ Olmesartan medoxomil
Sevikar HCT 40mg/5mg/12.5mg tablets (Daiichi Sankyo UK Ltd)	6180741000033115	18986711000001102	Amlodipine besilate/ Hydrochlorothiazide/ Olmesartan medoxomil
Sevikar HCT 40mg/10mg/12.5mg tablets (Daiichi Sankyo UK Ltd)	6180841000033113	18987011000001101	Amlodipine besilate/ Hydrochlorothiazide/ Olmesartan medoxomil

Sevikar HCT 40mg/5mg/25mg tablets (Daiichi Sankyo UK Ltd)	6180941000033117	18987311000001103	Amlodipine besilate/ Hydrochlorothiazide/ Olmesartan medoxomil
Sevikar HCT 40mg/10mg/25mg tablets (Daiichi Sankyo UK Ltd)	6181041000033110	18987611000001108	Amlodipine besilate/ Hydrochlorothiazide/ Olmesartan medoxomil
Amlodipine 10mg/5ml oral solution	7740641000033112	20478011000001105	Amlodipine
Perindopril erbumine 4mg / Amlodipine 5mg tablets	9105241000033114	23985011000001108	Amlodipine besilate/ Perindopril erbumine
Perindopril erbumine 4mg / Amlodipine 10mg tablets	9105341000033116	23984911000001108	Amlodipine besilate/ Perindopril erbumine
Perindopril erbumine 8mg / Amlodipine 5mg tablets	9105441000033110	23985211000001103	Amlodipine besilate/ Perindopril erbumine
Perindopril erbumine 8mg / Amlodipine 10mg tablets	9105541000033111	23985111000001109	Amlodipine besilate/ Perindopril erbumine
Amlodipine 10mg/5ml oral solution sugar free	10387441000033111	29826211000001109	Amlodipine
Amlodipine 5mg/5ml oral suspension sugar free	12682241000033110	36237311000001101	Amlodipine
Amlodipine 2.5mg tablets	13422241000033114	429828006	Amlodipine
Felodipine 2.5mg modified-release tablets	568041000033111	39020311000001104	Felodipine
Felodipine 10mg modified-release tablets	569041000033115	39020511000001105	Felodipine
Felodipine 5mg modified-release tablets	569141000033116	39020611000001109	Felodipine
Plendil 2.5mg modified-release tablets (AstraZeneca UK Ltd)	1093941000033116	562711000001104	Felodipine
Plendil 10mg modified-release tablets (AstraZeneca UK Ltd)	1094041000033119	48511000001101	Felodipine
Plendil 5mg modified-release tablets (AstraZeneca UK Ltd)	1094141000033115	490211000001101	Felodipine
Triapin 5mg/5mg modified-release tablets (Sanofi)	1834341000033110	3887911000001109	Felodipine/ Ramipril
Cabren 2.5mg modified-release tablets (Teva UK Ltd)	2928841000033117	3800311000001106	Felodipine
Cabren 5mg modified-release tablets (Teva UK Ltd)	2928941000033113	3800511000001100	Felodipine
Cabren 10mg modified-release tablets (Teva UK Ltd)	2929041000033116	3800711000001105	Felodipine
Felotens XL 5mg tablets (Thornton & Ross Ltd)	2979941000033119	4785111000001103	Felodipine
Felotens XL 10mg tablets (Thornton & Ross Ltd)	2980041000033115	4785511000001107	Felodipine
Vascalpha 5mg modified-release tablets (Accord Healthcare Ltd)	3034341000033117	5638311000001102	Felodipine
Vascalpha 10mg modified-release tablets (Accord Healthcare Ltd)	3034441000033111	5638811000001106	Felodipine
Cardioplen XL 5mg tablets (Chiesi Ltd)	3154841000033119	7887011000001104	Felodipine
Cardioplen XL 10mg tablets (Chiesi Ltd)	3154941000033110	7887511000001107	Felodipine
Felodipine 5mg modified-release / Ramipril 5mg tablets	3163241000033112	318177008	Felodipine/ Ramipril
Felodipine 2.5mg modified-release / Ramipril 2.5mg tablets	3163341000033119	318176004	Felodipine/ Ramipril
Felogen XL 5mg tablets (Mylan)	3177441000033110	4972811000001103	Felodipine
Felogen XL 10mg tablets (Mylan)	3177541000033111	4973011000001100	Felodipine
Cardioplen XL 2.5mg tablets (Chiesi Ltd)	4152841000033112	11506711000001103	Felodipine
Felotens XL 2.5mg tablets (Thornton & Ross Ltd)	4429241000033115	13127311000001107	Felodipine
Neofel XL 2.5mg tablets (Actavis UK Ltd)	4521541000033113	18167311000001103	Felodipine
Neofel XL 5mg tablets (Kent Pharma (UK) Ltd)	4521641000033114	8090111000001106	Felodipine

Neofel XL 10mg tablets (Kent Pharma (UK) Ltd)	4521741000033117	8089811000001107	Felodipine
Folpik XL 10mg tablets (Teva UK Ltd)	5576341000033118	5008911000001100	Felodipine
Folpik XL 2.5mg tablets (Teva UK Ltd)	5576441000033112	13565311000001101	Felodipine
Folpik XL 5mg tablets (Teva UK Ltd)	5576541000033113	5008511000001107	Felodipine
Triapin 2.5mg/2.5mg modified-release tablets (Sanofi)	8264941000033119	4093211000001109	Felodipine/ Ramipril
Parmid XL 2.5mg tablets (Sandoz Ltd)	9121541000033117	24221811000001105	Felodipine
Parmid XL 5mg tablets (Sandoz Ltd)	9121641000033116	7387911000001103	Felodipine
Parmid XL 10mg tablets (Sandoz Ltd)	9121741000033113	7388311000001103	Felodipine
Isradipine 2.5mg tablets	784641000033112	319280009	Isradipine
Prescal 2.5mg tablets (Novartis Pharmaceuticals UK Ltd)	1140241000033117	3689711000001108	Isradipine
Lercanidipine 10mg tablets	825941000033112	319316005	Lercanidipine hydrochloride
Zanidip 10mg tablets (Recordati Pharmaceuticals Ltd)	1550841000033111	20011000001105	Lercanidipine hydrochloride
Lercanidipine 20mg tablets	3908741000033114	10225911000001102	Lercanidipine hydrochloride
Zanidip 20mg tablets (Recordati Pharmaceuticals Ltd)	3908841000033116	10198711000001102	Lercanidipine hydrochloride
Cardene 20mg capsules (Astellas Pharma Ltd)	176741000033114	344811000001108	Nicardipine hydrochloride
Cardene 30mg capsules (Astellas Pharma Ltd)	176841000033116	291111000001102	Nicardipine hydrochloride
Cardene SR 30mg capsules (Astellas Pharma Ltd)	198941000033118	540311000001105	Nicardipine hydrochloride
Cardene SR 45mg capsules (Astellas Pharma Ltd)	199041000033110	118811000001102	Nicardipine hydrochloride
Nicardipine 30mg capsules	967141000033119	319218009	Nicardipine hydrochloride
Nicardipine 20mg capsules	967741000033115	319217004	Nicardipine hydrochloride
Nicardipine 30mg modified-release capsules	971441000033112	39021711000001108	Nicardipine hydrochloride
Nicardipine 45mg modified-release capsules	971541000033113	39021811000001100	Nicardipine hydrochloride
Adalat 10mg capsules (Bayer Plc)	14041000033115	782511000001108	Nifedipine
Adalat 5mg capsules (Bayer Plc)	14141000033116	27111000001107	Nifedipine
Adalat LA 30mg tablets (Bayer Plc)	17141000033112	2881311000001105	Nifedipine
Adalat LA 60mg tablets (Bayer Plc)	17241000033117	235511000001104	Nifedipine
Adipine MR 20 tablets (Chiesi Ltd)	18241000033116	74111000001103	Nifedipine
Adipine MR 10 tablets (Chiesi Ltd)	18341000033114	833611000001109	Nifedipine
Adalat retard 20mg tablets (Bayer Plc)	19341000033119	5011000001109	Nifedipine
Adalat retard 10mg tablets (Bayer Plc)	19441000033113	569011000001108	Nifedipine
Angiopine 10 capsules (Ashbourne Pharmaceuticals Ltd)	61841000033111	811311000001107	Nifedipine
Angiopine MR 20mg tablets (Ashbourne Pharmaceuticals Ltd)	65241000033114	865011000001105	Nifedipine
Angiopine MR 10mg tablets (Ashbourne Pharmaceuticals Ltd)	66141000033114	568911000001104	Nifedipine
Beta-Adalat modified-release capsules (Bayer Plc)	124341000033119	3142711000001107	Atenolol/ Nifedipine
Cardilate MR 20mg tablets (IVAX Pharmaceuticals UK Ltd)	199141000033114	905711000001103	Nifedipine
Coracten SR 10mg capsules (UCB Pharma Ltd)	336641000033114	126411000001108	Nifedipine

Coracten SR 20mg capsules (UCB Pharma Ltd)	364241000033112	389611000001101	Nifedipine
Fortipine LA 40 tablets (Advanz Pharma)	605941000033110	188711000001108	Nifedipine
Hypolar Retard 20 tablets (Sandoz Ltd)	738141000033119	677411000001108	Nifedipine
Nifedipine 10mg capsules	967241000033114	319223009	Nifedipine
Nifedipine 5mg capsules	967341000033116	319222004	Nifedipine
Nifedipine 20mg modified-release tablets	970941000033113	39022811000001109	Nifedipine
Nifedipine 10mg modified-release capsules	971141000033116	39022911000001104	Nifedipine
Nifedipine 30mg modified-release tablets	971241000033111	39111711000001105	Nifedipine
Nifedipine 60mg modified-release tablets	971341000033118	39022611000001105	Nifedipine
Nifedipine 10mg modified-release tablets	971841000033110	39022711000001101	Nifedipine
Nifedipine 40mg modified-release tablets	972441000033116	39107611000001108	Nifedipine
Nifedipine 20mg modified-release capsules	974141000033119	39022511000001106	Nifedipine
Tenif 50mg/20mg modified-release capsules (AstraZeneca UK Ltd)	1414941000033114	3142511000001102	Atenolol/ Nifedipine
Tensipine MR 10 tablets (Genus Pharmaceuticals Ltd)	1421241000033110	413111000001101	Nifedipine
Tensipine MR 20 tablets (Genus Pharmaceuticals Ltd)	1421341000033117	385611000001104	Nifedipine
Cardilate MR 10mg tablets (Teva UK Ltd)	1580841000033116	25911000001100	Nifedipine
Slofedipine 20mg tablets (Sterwin Medicines)	1697641000033111	843411000001105	Nifedipine
Coracten XL 30mg capsules (UCB Pharma Ltd)	1724941000033113	162811000001100	Nifedipine
Coracten XL 60mg capsules (UCB Pharma Ltd)	1725041000033113	3381511000001105	Nifedipine
Nifedipress MR 10 tablets (Dexcel-Pharma Ltd)	1745041000033110	904011000001104	Nifedipine
Nifedipress MR 20 tablets (Dexcel-Pharma Ltd)	1745141000033114	619111000001101	Nifedipine
Slofedipine XL 30mg tablets (Zentiva Pharma UK Ltd)	1752941000033115	2882011000001104	Nifedipine
Slofedipine XL 60 tablets (Zentiva Pharma UK Ltd)	1753041000033113	630411000001107	Nifedipine
Adalat LA 20mg tablets (Bayer Plc)	1766341000033113	881811000001100	Nifedipine
Nifedipine 30mg modified-release capsules	1778741000033118	39107511000001109	Nifedipine
Nifedipine 60mg modified-release capsules	1778841000033111	38896511000001101	Nifedipine
Coroday MR 20mg tablets (Mylan)	2051241000033116	309611000001102	Nifedipine
Nifopress Retard 20mg tablets (Advanz Pharma)	2189541000033117	280811000001100	Nifedipine
Calchan MR 10 tablets (Ranbaxy (UK) Ltd)	2295641000033116	627111000001104	Nifedipine
Calchan MR 20 tablets (Ranbaxy (UK) Ltd)	2295741000033113	17011000001100	Nifedipine
Nifedipine 20mg/ml oral drops	2639341000033117	9096811000001100	Nifedipine
Hypolar XL 30 tablets (Sandoz Ltd)	2776541000033111	2881811000001101	Nifedipine
Valni 20 Retard tablets (Tillomed Laboratories Ltd)	2955041000033115	693311000001101	Nifedipine
Adipine XL 30mg tablets (Chiesi Ltd)	3225441000033117	9049711000001108	Nifedipine
Adipine XL 60mg tablets (Chiesi Ltd)	3225541000033116	9049911000001105	Nifedipine
Atenolol 50mg / Nifedipine 20mg modified-release capsules	3246941000033118	35903311000001104	Atenolol/ Nifedipine
Nimodrel XL 30mg tablets (Zurich Pharmaceuticals)	3952641000033110	10189111000001106	Nifedipine
Nimodrel XL 60mg tablets (Zurich Pharmaceuticals)	3952741000033118	10189311000001108	Nifedipine
Valni XL 30mg tablets (Zentiva Pharma UK Ltd)	4451941000033110	13401911000001109	Nifedipine

Valni XL 60mg tablets (Zentiva Pharma UK Ltd)	4452041000033116	13402111000001101	Nifedipine
Nifedipine 5mg/5ml oral suspension	5056241000033112	8670311000001108	Nifedipine
Nifedipine 2.5mg/5ml oral suspension	5402541000033112	12303311000001109	Nifedipine
Neozipine XL 30mg tablets (Kent Pharma (UK) Ltd)	5817441000033119	10751211000001100	Nifedipine
Neozipine XL 60mg tablets (Kent Pharma (UK) Ltd)	5817541000033118	10751411000001101	Nifedipine
Adanif XL 30mg tablets (Advanz Pharma)	5891941000033115	17666011000001106	Nifedipine
Adanif XL 60mg tablets (Advanz Pharma)	5892041000033114	17666211000001101	Nifedipine
Nifedipine 10mg/5ml oral suspension	5973741000033117	8670111000001106	Nifedipine
Nidef 30mg modified-release tablets (Morningside Healthcare Ltd)	12372341000033117	34685211000001104	Nifedipine
Nidef 60mg modified-release tablets (Morningside Healthcare Ltd)	12372441000033111	34685811000001103	Nifedipine
Nimodipine 10mg/50ml solution for infusion vials	970741000033110	36031711000001104	Nimodipine
Nimodipine 30mg tablets	977441000033119	323273000	Nimodipine
Nimotop 30mg tablets (Bayer Plc)	977741000033114	3879211000001104	Nimodipine
Nisoldipine 10mg modified-release tablets	972141000033112	36031811000001107	Nisoldipine
Nisoldipine 20mg modified-release tablets	972241000033117	36031911000001102	Nisoldipine
Nisoldipine 30mg modified-release tablets	972341000033110	36032011000001109	Nisoldipine
Syscor MR 10 tablets (Forest Laboratories UK Ltd)	1401341000033110	3877011000001100	Nisoldipine
Syscor MR 20 tablets (Forest Laboratories UK Ltd)	1401441000033116	4070011000001100	Nisoldipine

Table S66. Non Dihydropyridines Aurum product codes

Term from EMIS	Prod code ID	dmd ID	Drug substance name
Adizem-SR 120mg capsules (Napp Pharmaceuticals Ltd)	17341000033110	2887311000001104	Diltiazem hydrochloride
Adizem-SR 180mg capsules (Napp Pharmaceuticals Ltd)	17441000033116	2886711000001103	Diltiazem hydrochloride
Adizem-SR 90mg capsules (Napp Pharmaceuticals Ltd)	17641000033119	2887011000001102	Diltiazem hydrochloride
Adizem-XL 120mg capsules (Napp Pharmaceuticals Ltd)	17741000033111	2937811000001108	Diltiazem hydrochloride
Adizem-XL 180mg capsules (Napp Pharmaceuticals Ltd)	17841000033118	2938011000001101	Diltiazem hydrochloride
Adizem-XL 240mg capsules (Napp Pharmaceuticals Ltd)	17941000033114	2886311000001102	Diltiazem hydrochloride
Adizem-XL 300mg capsules (Napp Pharmaceuticals Ltd)	18041000033112	2886111000001104	Diltiazem hydrochloride
Adizem-SR 120mg tablets (Napp Pharmaceuticals Ltd)	19641000033110	2885611000001102	Diltiazem hydrochloride
Angitil SR 90 capsules (Ethypharm UK Ltd)	65341000033116	857011000001109	Diltiazem hydrochloride
Angitil SR 120 capsules (Ethypharm UK Ltd)	65441000033110	298811000001103	Diltiazem hydrochloride
Angitil SR 180 capsules (Ethypharm UK Ltd)	65541000033111	336611000001101	Diltiazem hydrochloride
Angiozem CR 90mg tablets (Ashbourne Pharmaceuticals Ltd)	65941000033117	440711000001104	Diltiazem hydrochloride
Angiozem CR 120mg tablets (Ashbourne Pharmaceuticals Ltd)	66041000033110	144811000001101	Diltiazem hydrochloride
Angiozem 60mg modified-release tablets (Ashbourne Pharmaceuticals Ltd)	71141000033114	672311000001103	Diltiazem hydrochloride
Calcicard CR 120mg tablets (Teva UK Ltd)	199241000033119	104111000001100	Diltiazem hydrochloride
Calcicard CR 90mg tablets (Teva UK Ltd)	199341000033112	219611000001107	Diltiazem hydrochloride
Diltiazem 120mg modified-release capsules	451841000033110	39023211000001102	Diltiazem hydrochloride
Diltiazem 180mg modified-release capsules	451941000033119	39023311000001105	Diltiazem hydrochloride
Diltiazem 300mg modified-release capsules	452041000033113	39023611000001100	Diltiazem hydrochloride
Diltiazem 90mg modified-release capsules	452141000033112	39023111000001108	Diltiazem hydrochloride
Dilzem SR 60 capsules (Teva UK Ltd)	452641000033119	417111000001109	Diltiazem hydrochloride
Dilzem SR 90 capsules (Teva UK Ltd)	452741000033111	682311000001105	Diltiazem hydrochloride
Dilzem SR 120 capsules (Teva UK Ltd)	452841000033118	5711000001106	Diltiazem hydrochloride
Diltiazem 60mg modified-release capsules	452941000033114	39023011000001107	Diltiazem hydrochloride
Dilzem XL 120 capsules (Teva UK Ltd)	453041000033116	243111000001108	Diltiazem hydrochloride
Dilzem XL 180 capsules (Teva UK Ltd)	453141000033117	254911000001106	Diltiazem hydrochloride
Dilzem XL 240 capsules (Teva UK Ltd)	453241000033112	733511000001107	Diltiazem hydrochloride
Diltiazem 240mg modified-release capsules	453741000033118	39023511000001104	Diltiazem hydrochloride
Diltiazem 200mg modified-release capsules	453941000033115	39023411000001103	Diltiazem hydrochloride

Diltiazem 60mg modified-release tablets	454041000033118	39023811000001101	Diltiazem hydrochloride
Diltiazem 90mg modified-release tablets	458841000033115	39023911000001106	Diltiazem hydrochloride
Diltiazem 120mg modified-release tablets	459641000033113	39024011000001109	Diltiazem hydrochloride
Slozem 120mg capsules (Zentiva Pharma UK Ltd)	1346441000033111	130211000001109	Diltiazem hydrochloride
Slozem 180mg capsules (Zentiva Pharma UK Ltd)	1346541000033112	119211000001108	Diltiazem hydrochloride
Slozem 240mg capsules (Zentiva Pharma UK Ltd)	1346641000033113	599811000001104	Diltiazem hydrochloride
Tildiem LA 300 capsules (Sanofi)	1441741000033119	893111000001107	Diltiazem hydrochloride
Tildiem LA 200 capsules (Sanofi)	1441841000033112	261611000001107	Diltiazem hydrochloride
Tildiem Retard 120mg tablets (Sanofi)	1442741000033113	383911000001109	Diltiazem hydrochloride
Tildiem Retard 90mg tablets (Sanofi)	1442841000033115	103611000001105	Diltiazem hydrochloride
Tildiem 60mg modified-release tablets (Sanofi)	1443841000033113	762011000001102	Diltiazem hydrochloride
Viazem XL 120mg capsules (Thornton & Ross Ltd)	1520141000033118	32311000001105	Diltiazem hydrochloride
Viazem XL 180mg capsules (Thornton & Ross Ltd)	1520241000033113	33211000001108	Diltiazem hydrochloride
Viazem XL 240mg capsules (Thornton & Ross Ltd)	1520341000033115	886511000001107	Diltiazem hydrochloride
Viazem XL 300mg capsules (Thornton & Ross Ltd)	1520441000033114	407011000001105	Diltiazem hydrochloride
Viazem XL 360mg capsules (Thornton & Ross Ltd)	1520541000033110	648211000001107	Diltiazem hydrochloride
Angitil XL 240 capsules (Ethypharm UK Ltd)	1570441000033110	75111000001104	Diltiazem hydrochloride
Angitil XL 300 capsules (Ethypharm UK Ltd)	1570541000033111	467111000001105	Diltiazem hydrochloride
Dilcardia SR 120mg capsules (Mylan)	1602341000033116	723811000001107	Diltiazem hydrochloride
Dilcardia SR 60mg capsules (Mylan)	1602441000033110	937011000001101	Diltiazem hydrochloride
Dilcardia SR 90mg capsules (Mylan)	1602541000033111	353011000001101	Diltiazem hydrochloride
Optil 60mg modified-release tablets (Opus Pharmaceuticals Ltd)	1661641000033116	527711000001108	Diltiazem hydrochloride
Diltiazem 360mg modified-release capsules	1800341000033113	39023711000001109	Diltiazem hydrochloride
Zemtard 120 XL capsules (Galen Ltd)	1833041000033110	105211000001107	Diltiazem hydrochloride
Zemtard 180 XL capsules (Galen Ltd)	1833141000033114	813611000001103	Diltiazem hydrochloride
Zemtard 240 XL capsules (Galen Ltd)	1833241000033119	345411000001107	Diltiazem hydrochloride
Zemtard 300 XL capsules (Galen Ltd)	1833341000033112	866811000001107	Diltiazem hydrochloride
Slozem 300mg capsules (Zentiva Pharma UK Ltd)	2261241000033118	550211000001104	Diltiazem hydrochloride
Adizem-XL 200mg capsules (Napp Pharmaceuticals Ltd)	2653341000033118	2886511000001108	Diltiazem hydrochloride
Disogram SR 60mg capsules (Ranbaxy (UK) Ltd)	2674841000033110	469811000001106	Diltiazem hydrochloride
Disogram SR 90mg capsules (Ranbaxy (UK) Ltd)	2674941000033119	256611000001109	Diltiazem hydrochloride

Disogram SR 120mg capsules (Ranbaxy (UK) Ltd)	2675041000033119	116711000001106	Diltiazem hydrochloride
Disogram SR 180mg capsules (Ranbaxy (UK) Ltd)	2675341000033117	713411000001103	Diltiazem hydrochloride
Disogram SR 240mg capsules (Ranbaxy (UK) Ltd)	2675641000033113	108711000001107	Diltiazem hydrochloride
Disogram SR 300mg capsules (Ranbaxy (UK) Ltd)	2676041000033111	34711000001104	Diltiazem hydrochloride
Bi-Carzem SR 60mg capsules (Tillomed Laboratories Ltd)	2779141000033112	540011000001107	Diltiazem hydrochloride
Bi-Carzem SR 90mg capsules (Tillomed Laboratories Ltd)	2779241000033117	764511000001100	Diltiazem hydrochloride
Bi-Carzem SR 120mg capsules (Tillomed Laboratories Ltd)	2779341000033110	580711000001101	Diltiazem hydrochloride
Diltiazem 2% cream	2920141000033113	8793111000001101	Diltiazem hydrochloride
Zildil 90mg modified-release capsules (Healthcare Pharma Ltd)	2952341000033113	200811000001104	Diltiazem hydrochloride
Zildil SR 120mg capsules (Healthcare Pharma Ltd)	2952441000033119	655411000001104	Diltiazem hydrochloride
Zemret 180 XL capsules (Tillomed Laboratories Ltd)	2955141000033116	924711000001109	Diltiazem hydrochloride
Zemret 240 XL capsules (Tillomed Laboratories Ltd)	2955241000033111	823511000001107	Diltiazem hydrochloride
Zemret 300 XL capsules (Tillomed Laboratories Ltd)	2955341000033118	640311000001101	Diltiazem hydrochloride
Retalzem 60 modified-release tablets (Kent Pharma (UK) Ltd)	4590741000033116	865711000001107	Diltiazem hydrochloride
Diltiazem 60mg/5ml oral suspension	4953741000033114	8456911000001108	Diltiazem hydrochloride
Bi-Carzem XL 240mg capsules (Tillomed Laboratories Ltd)	5816841000033119	884811000001103	Diltiazem hydrochloride
Bi-Carzem XL 300mg capsules (Tillomed Laboratories Ltd)	5816941000033110	158411000001105	Diltiazem hydrochloride
Diltiazem 2% ointment	5991841000033116	8793311000001104	Diltiazem hydrochloride
Diltiazem 60mg/5ml oral solution	5991941000033112	8457011000001107	Diltiazem hydrochloride
Kenzem SR 60mg capsules (Kent Pharma (UK) Ltd)	9118241000033117	8885711000001100	Diltiazem hydrochloride
Kenzem SR 90mg capsules (Kent Pharma (UK) Ltd)	9118341000033110	8886211000001101	Diltiazem hydrochloride
Kenzem SR 120mg capsules (Kent Pharma (UK) Ltd)	9118441000033116	8886511000001103	Diltiazem hydrochloride
Diltiazem 0.2% cream	13771541000033112	18519711000001103	Diltiazem hydrochloride
Cordilox 120mg tablets (IVAX Pharmaceuticals UK Ltd)	374841000033119	867011000001103	Verapamil hydrochloride
Cordilox 160mg tablets (IVAX Pharmaceuticals UK Ltd)	374941000033110	783511000001101	Verapamil hydrochloride
Cordilox 40mg tablets (IVAX Pharmaceuticals UK Ltd)	375041000033110	557111000001106	Verapamil hydrochloride
Cordilox 80mg tablets (IVAX Pharmaceuticals UK Ltd)	375141000033114	258911000001104	Verapamil hydrochloride
Half Securon SR 120mg tablets (Mylan)	660641000033112	846611000001102	Verapamil hydrochloride
Securon IV 5mg/2ml solution for injection ampoules (Mylan)	1271741000033116	4399811000001108	Verapamil hydrochloride
Securon 120mg tablets (Abbott Laboratories Ltd)	1274741000033119	140411000001109	Verapamil hydrochloride
Securon SR 240mg tablets (Mylan)	1275141000033117	540111000001108	Verapamil hydrochloride

Tarka modified-release capsules (Abbott Laboratories Ltd)	1406341000033117	3691211000001101	Trandolapril/ Verapamil hydrochloride
Univer 120mg modified-release capsules (Teva UK Ltd)	1489541000033117	219711000001103	Verapamil hydrochloride
Univer 180mg modified-release capsules (Teva UK Ltd)	1489641000033116	391811000001102	Verapamil hydrochloride
Univer 240mg modified-release capsules (Teva UK Ltd)	1489741000033113	65411000001102	Verapamil hydrochloride
Verapamil 120mg modified-release tablets	1509041000033119	35367911000001103	Verapamil hydrochloride
Verapamil 240mg modified-release tablets	1509141000033115	38750211000001107	Verapamil hydrochloride
Verapamil 40mg/5ml oral solution sugar free	1509841000033114	4139411000001106	Verapamil hydrochloride
Verapamil 120mg modified-release capsules	1511141000033118	36565011000001105	Verapamil hydrochloride
Verapamil 180mg modified-release capsules	1511241000033113	36149411000001103	Verapamil hydrochloride
Verapamil 240mg modified-release capsules	1511341000033115	39021011000001106	Verapamil hydrochloride
Verapamil 120mg tablets	1512941000033111	318206008	Verapamil hydrochloride
Verapamil 160mg tablets	1513141000033119	318248001	Verapamil hydrochloride
Verapamil 40mg tablets	1513241000033114	318204006	Verapamil hydrochloride
Verapamil 80mg tablets	1513341000033116	318205007	Verapamil hydrochloride
Vertab SR 240 tablets (Chiesi Ltd)	1754841000033116	675611000001104	Verapamil hydrochloride
Verapress MR 240mg tablets (Dexcel-Pharma Ltd)	1833541000033117	525311000001101	Verapamil hydrochloride
Cordilox MR 240mg tablets (Teva UK Ltd)	2129041000033118	740811000001102	Verapamil hydrochloride
Zolvera 40mg/5ml oral solution (Rosemont Pharmaceuticals Ltd)	2641041000033116	4129511000001107	Verapamil hydrochloride
Ranvera MR 240mg tablets (Ranbaxy (UK) Ltd)	2845841000033119	3634111000001100	Verapamil hydrochloride
Vera-Til SR 120mg tablets (Tillomed Laboratories Ltd)	2890641000033116	7418511000001104	Verapamil hydrochloride
Vera-Til SR 240mg tablets (Tillomed Laboratories Ltd)	2890741000033113	7418711000001109	Verapamil hydrochloride
Verapamil 180mg modified-release / Trandolapril 2mg capsules	3163041000033116	36149211000001102	Trandolapril/ Verapamil hydrochloride
Verapamil 5mg/2ml solution for injection ampoules	4260341000033110	36149611000001100	Verapamil hydrochloride

Table S67. Angiotensin converting enzyme inhibitors Aurum product codes

Term from EMIS	Prod code ID	dmd ID	Drug substance name
Ramipril 1.25mg capsules	1151341000033117	318900007	Ramipril
Ramipril 2.5mg capsules	1151441000033111	318901006	Ramipril
Ramipril 5mg capsules	1151541000033112	318902004	Ramipril
Tritace 1.25mg capsules (Aventis Pharma)	1455541000033110	111611000001109	Ramipril
Tritace 2.5mg capsules (Sanofi)	1455641000033111	835411000001105	Ramipril
Tritace 5mg capsules (Sanofi)	1455741000033119	802311000001101	Ramipril
Ramipril 10mg capsules	1769841000033111	318906001	Ramipril
Tritace 10mg capsules (Sanofi)	1769941000033115	43711000001100	Ramipril
Triapin 5mg/5mg modified-release tablets (Sanofi)	1834341000033110	3887911000001109	Felodipine/ Ramipril
Ramipril 5mg/5ml oral suspension	2883341000033118	8720911000001100	Ramipril
Ramipril 1.25mg tablets	2989541000033115	408040007	Ramipril
Ramipril 2.5mg tablets	2989641000033119	408050008	Ramipril
Ramipril 5mg tablets	2989741000033111	408051007	Ramipril
Ramipril 10mg tablets	2989841000033118	408052000	Ramipril
Tritace 1.25mg tablets (Sanofi)	2989941000033114	5010511000001106	Ramipril
Tritace 2.5mg tablets (Sanofi)	2990041000033116	5010811000001109	Ramipril
Tritace 5mg tablets (Sanofi)	2990141000033117	5011111000001108	Ramipril
Tritace 10mg tablets (Sanofi)	2990241000033112	5011411000001103	Ramipril
Lopace 2.5mg capsules (Discovery Pharmaceuticals)	3159741000033111	7948711000001102	Ramipril
Lopace 5mg capsules (Discovery Pharmaceuticals)	3159841000033118	7948911000001100	Ramipril
Lopace 10mg capsules (Discovery Pharmaceuticals)	3159941000033114	7949111000001105	Ramipril
Felodipine 5mg modified-release / Ramipril 5mg tablets	3163241000033112	318177008	Felodipine/ Ramipril
Felodipine 2.5mg modified-release / Ramipril 2.5mg tablets	3163341000033119	318176004	Felodipine/ Ramipril
Ramipril 1.25mg/5ml oral suspension	4152241000033113	8720311000001101	Ramipril
Ramipril 2.5mg/5ml oral suspension	4805941000033114	8720711000001102	Ramipril
Ramipril 10mg/5ml oral solution	5887841000033118	8720411000001108	Ramipril
Ramipril 10mg/5ml oral suspension	5887941000033114	8720511000001107	Ramipril
Ramipril 5mg/5ml oral solution	5890341000033110	8720811000001105	Ramipril
Ramipril 2.5mg/5ml oral solution	5890541000033115	8720611000001106	Ramipril
Ramipril 1.25mg/5ml oral solution	5998241000033112	8720211000001109	Ramipril
Ramipril 2.5mg/5ml oral solution sugar free	6517041000033110	19877111000001100	Ramipril
Triapin 2.5mg/2.5mg modified-release tablets (Sanofi)	8264941000033119	4093211000001109	Felodipine/ Ramipril
Enalapril 10mg tablets	522641000033110	318853004	Enalapril maleate
Enalapril 20mg tablets	522741000033118	318855006	Enalapril maleate
Enalapril 5mg tablets	522841000033111	318851002	Enalapril maleate
Enalapril 2.5mg tablets	523541000033115	318850001	Enalapril maleate
Innovace 10mg tablets (Organon Pharma (UK) Ltd)	769041000033116	316111000001104	Enalapril maleate

Innovace 2.5mg tablets (Organon Pharma (UK) Ltd)	769141000033117	749611000001107	Enalapril maleate
Innovace 20mg tablets (Organon Pharma (UK) Ltd)	769241000033112	302311000001104	Enalapril maleate
Innovace 5mg tablets (Organon Pharma (UK) Ltd)	769341000033119	730211000001104	Enalapril maleate
Innozide 20mg/12.5mg tablets (Organon Pharma (UK) Ltd)	770141000033115	146811000001108	Enalapril maleate/ Hydrochlorothiazide
Pralenal 2.5 tablets (Opus Pharmaceuticals Ltd)	1918641000033112	448611000001106	Enalapril maleate
Pralenal 5 tablets (Opus Pharmaceuticals Ltd)	1918741000033115	578511000001100	Enalapril maleate
Pralenal 10 tablets (Opus Pharmaceuticals Ltd)	1918841000033113	761411000001101	Enalapril maleate
Pralenal 20 tablets (Opus Pharmaceuticals Ltd)	1918941000033117	508511000001105	Enalapril maleate
Enalapril 20mg / Hydrochlorothiazide 12.5mg tablets	3162241000033117	39691911000001109	Enalapril maleate/ Hydrochlorothiazide
Enalapril 5mg/5ml oral suspension sugar free	3279241000033117	20092911000001108	Enalapril maleate
Enalapril 10mg/5ml oral solution	5967041000033117	8485711000001108	Enalapril maleate
Enalapril 10mg/5ml oral suspension	5967141000033118	8485811000001100	Enalapril maleate
Enalapril 5mg/5ml oral solution	5992541000033111	8486911000001103	Enalapril maleate
Enalapril 5mg/5ml oral suspension	5992641000033112	8487011000001104	Enalapril maleate
Enalapril 1.25mg/5ml oral solution	6012441000033118	8485511000001103	Enalapril maleate
Enalapril 1.25mg/5ml oral suspension	6012541000033117	8485611000001104	Enalapril maleate
Enalapril 20mg tablets (Imported)	13740241000033119	38955411000001109	Enalapril maleate

Table S68. Angiotensin II receptor blocker Aurum product codes

Term from EMIS	Prod code ID	dmd ID	Drug substance name
Azilsartan medoxomil 20mg tablets	7754841000033119	20418711000001104	Azilsartan medoxomil
Azilsartan medoxomil 40mg tablets	7754941000033110	449109006	Azilsartan medoxomil
Azilsartan medoxomil 80mg tablets	7755041000033110	449333009	Azilsartan medoxomil
Edarbi 20mg tablets (Takeda UK Ltd)	7755141000033114	20350911000001100	Azilsartan medoxomil
Edarbi 40mg tablets (Takeda UK Ltd)	7755241000033119	20351211000001103	Azilsartan medoxomil
Edarbi 80mg tablets (Takeda UK Ltd)	7755341000033112	20351811000001102	Azilsartan medoxomil
Amias 16mg tablets (Takeda UK Ltd)	59141000033113	908511000001100	Candesartan cilexetil
Amias 2mg tablets (Takeda UK Ltd)	59241000033118	97311000001103	Candesartan cilexetil
Amias 4mg tablets (Takeda UK Ltd)	59341000033111	857411000001100	Candesartan cilexetil
Amias 8mg tablets (Takeda UK Ltd)	59441000033117	36011000001106	Candesartan cilexetil
Candesartan 16mg tablets	211541000033119	318980005	Candesartan cilexetil
Candesartan 2mg tablets	211641000033118	318977009	Candesartan cilexetil
Candesartan 4mg tablets	211741000033110	318978004	Candesartan cilexetil
Candesartan 8mg tablets	211841000033117	318979007	Candesartan cilexetil
Candesartan 32mg tablets	3227241000033111	376998003	Candesartan cilexetil
Amias 32mg tablets (Takeda UK Ltd)	3227341000033118	8983911000001107	Candesartan cilexetil
Eprosartan 300mg tablets	2036341000033115	318994006	Eprosartan mesilate
Eprosartan 400mg tablets	2036441000033114	318995007	Eprosartan mesilate
Eprosartan 600mg tablets	2036541000033110	318996008	Eprosartan mesilate
Teveten 300mg tablets (Mylan)	2040841000033113	401211000001105	Eprosartan mesilate
Teveten 400mg tablets (Abbott Healthcare Products Ltd)	2040941000033117	151411000001103	Eprosartan mesilate
Teveten 600mg tablets (Mylan)	2041041000033110	872011000001109	Eprosartan mesilate
Aprovel 150mg tablets (Sanofi)	75941000033114	859711000001103	Irbesartan
Aprovel 300mg tablets (Sanofi)	76041000033116	323211000001107	Irbesartan
Aprovel 75mg tablets (Sanofi)	76141000033117	434511000001104	Irbesartan
Irbesartan 150mg tablets	775441000033117	318969005	Irbesartan
Irbesartan 300mg tablets	775541000033116	318970006	Irbesartan
Irbesartan 75mg tablets	775641000033115	318968002	Irbesartan
CoAprovel 150mg/12.5mg tablets (Sanofi)	2216141000033115	792411000001108	Hydrochlorothiazide/ Irbesartan
CoAprovel 300mg/12.5mg tablets (Sanofi)	2216241000033110	682711000001109	Hydrochlorothiazide/ Irbesartan
Irbesartan 150mg / Hydrochlorothiazide 12.5mg tablets	3161141000033116	134461004	Hydrochlorothiazide/ Irbesartan
Irbesartan 300mg / Hydrochlorothiazide 12.5mg tablets	3161241000033111	134460003	Hydrochlorothiazide/ Irbesartan
Irbesartan 300mg / Hydrochlorothiazide 25mg tablets	3995241000033112	10970311000001105	Hydrochlorothiazide/ Irbesartan
CoAprovel 300mg/25mg tablets (Sanofi)	3995341000033119	10968611000001106	Hydrochlorothiazide/ Irbesartan
Irbesartan 300mg/5ml oral suspension	4954341000033111	12639511000001103	Irbesartan

Irbesartan 150mg/5ml oral suspension	5970641000033117	8580811000001101	Irbesartan
Irbesartan 37.5mg oral powder sachets	6435741000033117	19481911000001105	Irbesartan
Ifirmasta 75mg tablets (Consilient Health Ltd)	8554141000033116	22720311000001108	Irbesartan
Ifirmasta 150mg tablets (Consilient Health Ltd)	8554241000033111	22720611000001103	Irbesartan
Ifirmasta 300mg tablets (Consilient Health Ltd)	8554341000033118	22720811000001104	Irbesartan
Cozaar 25mg tablets (Organon Pharma (UK) Ltd)	370441000033112	266511000001104	Losartan potassium
Cozaar 50mg tablets (Organon Pharma (UK) Ltd)	370541000033113	53611000001106	Losartan potassium
Cozaar-Comp 50mg/12.5mg tablets (Organon Pharma (UK) Ltd)	370841000033110	255911000001105	Hydrochlorothiazide/ Losartan potassium
Losartan 25mg tablets	851841000033117	318955005	Losartan potassium
Losartan 50mg tablets	851941000033113	318956006	Losartan potassium
Losartan 100mg tablets	2720141000033115	407784004	Losartan potassium
Cozaar 100mg tablets (Organon Pharma (UK) Ltd)	2720241000033110	245811000001102	Losartan potassium
Losartan 50mg / Hydrochlorothiazide 12.5mg tablets	3161741000033117	318959004	Hydrochlorothiazide/ Losartan potassium
Losartan 100mg / Hydrochlorothiazide 25mg tablets	3346841000033117	395497004	Hydrochlorothiazide/ Losartan potassium
Cozaar-Comp 100mg/25mg tablets (Organon Pharma (UK) Ltd)	3346941000033113	9566911000001105	Hydrochlorothiazide/ Losartan potassium
Losartan 100mg / Hydrochlorothiazide 12.5mg tablets	4424741000033110	13112711000001103	Hydrochlorothiazide/ Losartan potassium
Cozaar-Comp 100mg/12.5mg tablets (Organon Pharma (UK) Ltd)	4424841000033117	13094111000001102	Hydrochlorothiazide/ Losartan potassium
Losartan 12.5mg tablets	4957541000033114	15148111000001100	Losartan potassium
Cozaar 12.5mg tablets (Organon Pharma (UK) Ltd)	4957641000033110	15138911000001101	Losartan potassium
Losartan 2.5mg/ml oral suspension sugar free	5149941000033118	15507411000001105	Losartan potassium
Cozaar 2.5mg/ml oral suspension (Organon Pharma (UK) Ltd)	5150041000033110	15506811000001105	Losartan potassium
Losartan 100mg/5ml oral solution	5971741000033116	15451111000001107	Losartan potassium
Losartan 100mg/5ml oral suspension	5971841000033114	15451211000001101	Losartan potassium
Losartan 50mg/5ml oral solution	5971941000033118	14159411000001106	Losartan potassium
Losartan 50mg/5ml oral suspension	5972041000033112	14159511000001105	Losartan potassium
Olmesartan medoxomil 10mg tablets	2944541000033111	408055003	Olmesartan medoxomil
Olmesartan medoxomil 20mg tablets	2944641000033112	385542009	Olmesartan medoxomil
Olmesartan medoxomil 40mg tablets	2944741000033115	385543004	Olmesartan medoxomil
Olmetec 10mg tablets (Daiichi Sankyo UK Ltd)	2944841000033113	4624011000001101	Olmesartan medoxomil
Olmetec 20mg tablets (Daiichi Sankyo UK Ltd)	2944941000033117	4624311000001103	Olmesartan medoxomil
Olmetec 40mg tablets (Daiichi Sankyo UK Ltd)	2945041000033117	4624611000001108	Olmesartan medoxomil
Olmesartan medoxomil 20mg / Hydrochlorothiazide 12.5mg tablets	3908941000033112	409184002	Hydrochlorothiazide/ Olmesartan medoxomil
Olmesartan medoxomil 20mg / Hydrochlorothiazide 25mg tablets	3909041000033115	10270711000001105	Hydrochlorothiazide/ Olmesartan medoxomil

Olmetec Plus 20mg/12.5mg tablets (Daiichi Sankyo UK Ltd)	3909141000033116	10261511000001103	Hydrochlorothiazide/ Olmesartan medoxomil
Olmetec Plus 20mg/25mg tablets (Daiichi Sankyo UK Ltd)	3909241000033111	10261811000001100	Hydrochlorothiazide/ Olmesartan medoxomil
Olmesartan medoxomil 10mg/5ml oral suspension	4273241000033118	14680711000001103	Olmesartan medoxomil
Olmesartan medoxomil 20mg / Amlodipine 5mg tablets	5005841000033114	429502004	Amlodipine besilate/ Olmesartan medoxomil
Olmesartan medoxomil 40mg / Amlodipine 5mg tablets	5005941000033118	429503009	Amlodipine besilate/ Olmesartan medoxomil
Olmesartan medoxomil 40mg / Amlodipine 10mg tablets	5006041000033111	429678006	Amlodipine besilate/ Olmesartan medoxomil
Sevikar 20mg/5mg tablets (Daiichi Sankyo UK Ltd)	5006141000033110	15773211000001105	Amlodipine besilate/ Olmesartan medoxomil
Sevikar 40mg/5mg tablets (Daiichi Sankyo UK Ltd)	5006241000033115	15772911000001108	Amlodipine besilate/ Olmesartan medoxomil
Sevikar 40mg/10mg tablets (Daiichi Sankyo UK Ltd)	5006341000033113	15772611000001102	Amlodipine besilate/ Olmesartan medoxomil
Olmesartan medoxomil 40mg / Hydrochlorothiazide 12.5mg tablets	5566341000033111	409185001	Hydrochlorothiazide/ Olmesartan medoxomil
Olmetec Plus 40mg/12.5mg tablets (Daiichi Sankyo UK Ltd)	5566441000033117	17220911000001102	Hydrochlorothiazide/ Olmesartan medoxomil
Sevikar HCT 20mg/5mg/12.5mg tablets (Daiichi Sankyo UK Ltd)	6180641000033112	18986411000001108	Amlodipine besilate/ Hydrochlorothiazide/ Olmesartan medoxomil
Sevikar HCT 40mg/5mg/12.5mg tablets (Daiichi Sankyo UK Ltd)	6180741000033115	18986711000001102	Amlodipine besilate/ Hydrochlorothiazide/ Olmesartan medoxomil
Sevikar HCT 40mg/10mg/12.5mg tablets (Daiichi Sankyo UK Ltd)	6180841000033113	18987011000001101	Amlodipine besilate/ Hydrochlorothiazide/ Olmesartan medoxomil
Sevikar HCT 40mg/5mg/25mg tablets (Daiichi Sankyo UK Ltd)	6180941000033117	18987311000001103	Amlodipine besilate/ Hydrochlorothiazide/ Olmesartan medoxomil
Sevikar HCT 40mg/10mg/25mg tablets (Daiichi Sankyo UK Ltd)	6181041000033110	18987611000001108	Amlodipine besilate/ Hydrochlorothiazide/ Olmesartan medoxomil
Diovan 160mg capsules (Novartis Pharmaceuticals UK Ltd)	437641000033118	117011000001107	Valsartan
Diovan 40mg capsules (Novartis Pharmaceuticals UK Ltd)	437741000033110	777611000001101	Valsartan
Diovan 80mg capsules (Novartis Pharmaceuticals UK Ltd)	437841000033117	554511000001105	Valsartan
Valsartan 160mg capsules	1498241000033111	318963006	Valsartan
Valsartan 40mg capsules	1498341000033118	318961008	Valsartan
Valsartan 80mg capsules	1498441000033112	318962001	Valsartan

Co-Diovan 160mg/12.5mg tablets (Novartis Pharmaceuticals UK Ltd)	3152541000033112	7668611000001104	Hydrochlorothiazide/ Valsartan
Co-Diovan 160mg/25mg tablets (Novartis Pharmaceuticals UK Ltd)	3152641000033113	7668911000001105	Hydrochlorothiazide/ Valsartan
Valsartan 160mg / Hydrochlorothiazide 12.5mg tablets	3161441000033112	395521005	Hydrochlorothiazide/ Valsartan
Valsartan 160mg / Hydrochlorothiazide 25mg tablets	3161541000033113	409298002	Hydrochlorothiazide/ Valsartan
Valsartan 80mg / Hydrochlorothiazide 12.5mg tablets	3190741000033112	377488008	Hydrochlorothiazide/ Valsartan
Co-Diovan 80mg/12.5mg tablets (Novartis Pharmaceuticals UK Ltd)	3190841000033119	8150111000001108	Hydrochlorothiazide/ Valsartan
Valsartan 40mg tablets	3201341000033115	416515008	Valsartan
Diovan 40mg tablets (Novartis Pharmaceuticals UK Ltd)	3201441000033114	8263211000001101	Valsartan
Amlodipine 5mg / Valsartan 80mg tablets	4021441000033114	11160311000001109	Amlodipine besilate/ Valsartan
Amlodipine 5mg / Valsartan 160mg tablets	4021541000033110	11160211000001101	Amlodipine besilate/ Valsartan
Amlodipine 10mg / Valsartan 160mg tablets	4021641000033111	11160111000001107	Amlodipine besilate/ Valsartan
Exforge 5mg/80mg tablets (Novartis Pharmaceuticals UK Ltd)	4021741000033119	11161811000001108	Amlodipine besilate/ Valsartan
Exforge 5mg/160mg tablets (Novartis Pharmaceuticals UK Ltd)	4021841000033112	11161511000001105	Amlodipine besilate/ Valsartan
Exforge 10mg/160mg tablets (Novartis Pharmaceuticals UK Ltd)	4021941000033116	11160711000001108	Amlodipine besilate/ Valsartan
Valsartan 320mg tablets	4424941000033113	376487009	Valsartan
Diovan 320mg tablets (Novartis Pharmaceuticals UK Ltd)	4425041000033113	13143311000001102	Valsartan
Valsartan 80mg tablets	6515441000033113	375034009	Valsartan
Valsartan 160mg tablets	6515541000033114	375035005	Valsartan
Valsartan 3mg/ml oral solution	6528441000033113	20007411000001100	Valsartan
Diovan 3mg/1ml oral solution (Novartis Pharmaceuticals UK Ltd)	6528541000033114	20001711000001102	Valsartan
Sacubitril 24mg / Valsartan 26mg tablets	10943641000033117	31142011000001103	Sacubitril/ Valsartan
Sacubitril 49mg / Valsartan 51mg tablets	10943741000033114	31142111000001102	Sacubitril/ Valsartan
Sacubitril 97mg / Valsartan 103mg tablets	10943841000033116	31142211000001108	Sacubitril/ Valsartan
Entresto 24mg/26mg tablets (Novartis Pharmaceuticals UK Ltd)	10943941000033112	31136811000001109	Sacubitril/ Valsartan
Entresto 49mg/51mg tablets (Novartis Pharmaceuticals UK Ltd)	10944041000033114	31136411000001107	Sacubitril/ Valsartan
Entresto 97mg/103mg tablets (Novartis Pharmaceuticals UK Ltd)	10944141000033113	31138011000001104	Sacubitril/ Valsartan

Table S69. Beta blockers Aurum product codes

Term from EMIS	Prod code ID	dmd ID	Drug substance name
Sotalol 25mg/5ml oral solution	3953441000033116	8726811000001107	Sotalol hydrochloride
Sotalol 25mg/5ml oral suspension	9776441000033119	8726911000001102	Sotalol hydrochloride
Acebutolol 100mg capsules	3041000033115	318412000	Acebutolol hydrochloride
Sectral 100mg capsules (Sanofi)	1268141000033119	632811000001105	Acebutolol hydrochloride
Sectral 200mg capsules (Sanofi)	1268241000033114	925711000001108	Acebutolol hydrochloride
Acebutolol 200mg capsules	3141000033116	318413005	Acebutolol hydrochloride
Acebutolol 400mg tablets	10741000033112	318414004	Acebutolol hydrochloride
Sectral 400mg tablets (Sanofi)	1278241000033118	298111000001105	Acebutolol hydrochloride
Atenix 50 tablets (Ashbourne Pharmaceuticals Ltd)	92541000033118	271911000001106	Atenolol
Tenormin LS 50mg tablets (AstraZeneca UK Ltd)	1426541000033115	423911000001107	Atenolol
Antipressan 50mg tablets (Teva UK Ltd)	71041000033110	874711000001101	Atenolol
Atenolol 50mg tablets	91141000033119	318420003	Atenolol
Atenolol 100mg tablets	91041000033118	318421004	Atenolol
Antipressan 100mg tablets (Teva UK Ltd)	70941000033117	734011000001102	Atenolol
Atenix 100 tablets (Ashbourne Pharmaceuticals Ltd)	90541000033117	181611000001107	Atenolol
Tenormin 100mg tablets (AstraZeneca UK Ltd)	1426441000033116	162411000001102	Atenolol
Tenormin 25mg tablets (AstraZeneca UK Ltd)	1428741000033112	317111000001101	Atenolol
Atenix 25 tablets (Ashbourne Pharmaceuticals Ltd)	91841000033113	482511000001101	Atenolol
Antipressan 25mg tablets (Teva UK Ltd)	69441000033112	877011000001106	Atenolol
Atenolol 25mg tablets	90641000033116	318434003	Atenolol
Betaloc 100mg tablets (AstraZeneca UK Ltd)	144341000033117	193911000001101	Metoprolol tartrate
Lopresor 100mg tablets (Recordati Pharmaceuticals Ltd)	850841000033110	756911000001107	Metoprolol tartrate
Metoprolol 100mg tablets	897741000033111	318474009	Metoprolol tartrate
Metoprolol 50mg tablets	897841000033118	318475005	Metoprolol tartrate
Betaloc 50mg tablets (AstraZeneca UK Ltd)	144441000033111	31811000001105	Metoprolol tartrate
Lopresor 50mg tablets (Recordati Pharmaceuticals Ltd)	850941000033119	113511000001109	Metoprolol tartrate
Corgard 40mg tablets (Sanofi-Synthelabo Ltd)	375241000033119	3686511000001108	Nadolol
Nadolol 40mg tablets	956341000033113	318480001	Nadolol
Nadolol 80mg tablets	956541000033118	318481002	Nadolol
Corgard 80mg tablets (Sanofi)	375341000033112	3687411000001106	Nadolol
Trasicor 20mg tablets (Amdipharm Plc)	1469641000033119	3379011000001106	Oxprenolol hydrochloride
Oxprenolol 20mg tablets	1026141000033119	318483004	Oxprenolol hydrochloride
Oxprenolol 40mg tablets	1026241000033114	318484005	Oxprenolol hydrochloride
Trasicor 40mg tablets (Amdipharm Plc)	1469741000033111	592911000001104	Oxprenolol hydrochloride

Trasicor 80mg tablets (Amdipharm Plc)	1469841000033118	568511000001106	Oxprenolol hydrochloride
Oxprenolol 80mg tablets	1026341000033116	318485006	Oxprenolol hydrochloride
Pindolol 5mg tablets	1091241000033112	318512002	Pindolol
Visken 5mg tablets (Advanz Pharma)	1524041000033111	3706111000001105	Pindolol
Visken 15mg tablets (Advanz Pharma)	1525141000033111	3887411000001101	Pindolol
Pindolol 15mg tablets	1091141000033117	318513007	Pindolol
Sotalol 40mg tablets	1367441000033117	318525005	Sotalol hydrochloride
Beta-Cardone 40mg tablets (Focus Pharmaceuticals Ltd)	144041000033119	900511000001103	Sotalol hydrochloride
Sotacor 80mg tablets (Cheplapharm Arzneimittel GmbH)	1366141000033111	463811000001109	Sotalol hydrochloride
Beta-Cardone 80mg tablets (Focus Pharmaceuticals Ltd)	144141000033115	104011000001101	Sotalol hydrochloride
Sotalol 80mg tablets	1367541000033116	318526006	Sotalol hydrochloride
Sotalol 200mg tablets	1367241000033118	318527002	Sotalol hydrochloride
Beta-Cardone 200mg tablets (Advanz Pharma)	143941000033116	447111000001104	Sotalol hydrochloride
Sotacor 160mg tablets (Bristol-Myers Squibb Pharmaceuticals Ltd)	1366041000033112	446211000001108	Sotalol hydrochloride
Sotalol 160mg tablets	1367141000033113	318528007	Sotalol hydrochloride
Timolol 10mg tablets	1444541000033113	318534000	Timolol maleate
Betim 10mg tablets (Meda Pharmaceuticals Ltd)	144741000033116	72811000001104	Timolol maleate
Co-Betaloc tablets (Pfizer Ltd)	372741000033116	2977611000001106	Hydrochlorothiazide/ Metoprolol tartrate
Metoprolol 100mg / Hydrochlorothiazide 12.5mg tablets	3161341000033118	318546001	Hydrochlorothiazide/ Metoprolol tartrate
Corgaretic 40mg tablets (Sanofi-Synthelabo Ltd)	375441000033118	3886211000001102	Bendroflumethiazide/ Nadolol
Corgaretic 80mg tablets (Sanofi-Synthelabo Ltd)	375541000033117	4057911000001102	Bendroflumethiazide/ Nadolol
Viskaldix tablets (Advanz Pharma)	1523941000033114	3638411000001101	Clopamide/ Pindolol
Pindolol 10mg / Clopamide 5mg tablets	3163141000033117	318552000	Clopamide/ Pindolol
Moducren tablets (Merck Sharp & Dohme Ltd)	935341000033118	74311000001101	Amiloride hydrochloride/ Hydrochlorothiazide/ Timolol maleate
Prestim tablets (Meda Pharmaceuticals Ltd)	1132741000033111	98411000001109	Bendroflumethiazide/ Timolol maleate
Timolol 10mg / Bendroflumethiazide 2.5mg tablets	3346641000033118	318556002	Bendroflumethiazide/ Timolol maleate
Tenben 25mg/1.25mg capsules (Galen Ltd)	1414241000033117	721411000001106	Atenolol/ Bendroflumethiazide
Tenoret 50mg/12.5mg tablets (AstraZeneca UK Ltd)	1426241000033117	825011000001103	Atenolol/ Chlortalidone
Tenchlor 50mg/12.5mg tablets (Teva UK Ltd)	1425441000033113	439411000001104	Atenolol/ Chlortalidone
Atenix Co 50 tablets (Ashbourne Pharmaceuticals Ltd)	92441000033119	629011000001109	Atenolol/ Chlortalidone
Co-tenidone 50mg/12.5mg tablets	376441000033110	318575007	Atenolol/ Chlortalidone
Secadrex 200mg/12.5mg tablets (Sanofi)	1274641000033111	878911000001106	Acebutolol hydrochloride/ Hydrochlorothiazide
Acebutolol 200mg / Hydrochlorothiazide 12.5mg tablets	3162941000033114	318586009	Acebutolol hydrochloride/ Hydrochlorothiazide

Kalten capsules (M & A Pharmachem Ltd)	793741000033117	237011000001100	Amiloride hydrochloride/ Atenolol/ Hydrochlorothiazide
Celectol 200mg tablets (Cheplapharm Arzneimittel GmbH)	236141000033110	864811000001100	Celiprolol hydrochloride
Celiprolol 200mg tablets	236041000033111	318619000	Celiprolol hydrochloride
Celiprolol 400mg tablets	233341000033116	318622003	Celiprolol hydrochloride
Celectol 400mg tablets (Cheplapharm Arzneimittel GmbH)	233241000033114	479211000001100	Celiprolol hydrochloride
Eucardic 12.5mg tablets (Roche Products Ltd)	556941000033118	334711000001101	Carvedilol
Carvedilol 12.5mg tablets	209941000033111	318631003	Carvedilol
Carvedilol 25mg tablets	210041000033115	318632005	Carvedilol
Eucardic 25mg tablets (Roche Products Ltd)	557041000033117	709911000001107	Carvedilol
Eucardic 3.125mg tablets (Roche Products Ltd)	1608941000033115	873111000001104	Carvedilol
Carvedilol 3.125mg tablets	1581241000033110	318633000	Carvedilol
Carvedilol 6.25mg tablets	1581341000033117	318635007	Carvedilol
Eucardic 6.25mg tablets (Roche Products Ltd)	1609041000033112	690511000001101	Carvedilol
Nadolol 40mg/5ml oral suspension	5378241000033112	12300811000001107	Nadolol
Tenormin 25mg/5ml syrup (AstraZeneca UK Ltd)	1424541000033110	373311000001100	Atenolol
Atenolol 25mg/5ml oral solution sugar free	90441000033118	35903211000001107	Atenolol
Tenif 50mg/20mg modified-release capsules (AstraZeneca UK Ltd)	1414941000033114	3142511000001102	Atenolol/ Nifedipine
Beta-Adalat modified-release capsules (Bayer Plc)	124341000033119	3142711000001107	Atenolol/ Nifedipine
Atenolol 50mg / Nifedipine 20mg modified-release capsules	3246941000033118	35903311000001104	Atenolol/ Nifedipine
Tenormin 5mg/10ml solution for injection ampoules (AstraZeneca UK Ltd)	1420741000033114	9111000001107	Atenolol
Atenolol 5mg/10ml solution for injection ampoules	89541000033111	35903411000001106	Atenolol
Metoprolol 5mg/5ml solution for injection ampoules	887141000033113	3631211000001109	Metoprolol tartrate
Betaloc I.V. 5mg/5ml solution for injection ampoules (Recordati Pharmaceuticals Ltd)	131141000033116	3615711000001109	Metoprolol tartrate
Sotacor 40mg/4ml solution for injection ampoules (Bristol-Myers Squibb Pharmaceuticals Ltd)	1358541000033113	694711000001100	Sotalol hydrochloride
Sotalol 40mg/4ml solution for injection ampoules	2908541000033114	35930811000001109	Sotalol hydrochloride
Slow-Trasicor 160mg tablets (Advanz Pharma)	1347441000033114	473011000001107	Oxprenolol hydrochloride
Oxprenolol 160mg modified-release tablets	1026041000033118	36023011000001102	Oxprenolol hydrochloride
Co-Betaloc SA tablets (Pfizer Ltd)	372841000033114	3853411000001104	Hydrochlorothiazide/ Metoprolol tartrate
Lopresor SR 200mg tablets (Recordati Pharmaceuticals Ltd)	851041000033112	916111000001108	Metoprolol tartrate
Betaloc-SA 200mg tablets (AstraZeneca UK Ltd)	144541000033112	185111000001102	Metoprolol tartrate
Metoprolol 200mg modified-release tablets	891641000033111	36035411000001108	Metoprolol tartrate
Nadolol 30mg/5ml oral solution	7871341000033114	16109911000001102	Nadolol
Nadolol 30mg/5ml oral suspension	10650141000033117	16110011000001107	Nadolol

Nadolol 20mg/5ml oral solution	5814141000033119	16130911000001101	Nadolol
Nadolol 20mg/5ml oral suspension	10650041000033116	16131011000001109	Nadolol
Timolol 10mg / Amiloride 2.5mg / Hydrochlorothiazide 25mg tablets (Essential Generics Ltd)	5376441000033114	16458411000001107	Amiloride hydrochloride/ Hydrochlorothiazide/ Timolol maleate
Co-tenidone 100mg/25mg tablets	376541000033111	377211005	Atenolol/ Chlortalidone
Atenix Co 100 tablets (Ashbourne Pharmaceuticals Ltd)	90741000033113	101411000001108	Atenolol/ Chlortalidone
Tenoretic 100mg/25mg tablets (AstraZeneca UK Ltd)	1426341000033110	288711000001105	Atenolol/ Chlortalidone
Tenchlor 100mg/25mg tablets (Teva UK Ltd)	1425541000033114	298311000001107	Atenolol/ Chlortalidone
Trasidrex modified-release tablets (Mercury Pharma Group Ltd)	1469941000033114	3444411000001107	Cyclopenthiazide/ Oxprenolol hydrochloride
Co-prenozide 160mg/0.25mg modified-release tablets	377341000033119	36091611000001101	Cyclopenthiazide/ Oxprenolol hydrochloride
Carvedilol 5mg/5ml oral suspension	5288741000033116	8356811000001107	Carvedilol
Metoprolol 12.5mg/5ml oral solution	2616041000033116	8668211000001107	Metoprolol tartrate
Metoprolol 12.5mg/5ml oral suspension	4815641000033117	8668411000001106	Metoprolol tartrate
Metoprolol 25mg/5ml oral solution	5575541000033119	8668711000001100	Metoprolol tartrate
Metoprolol 50mg/5ml oral solution	5972841000033117	8668911000001103	Metoprolol tartrate
Metoprolol 50mg/5ml oral suspension	5972941000033113	8669111000001108	Metoprolol tartrate

Table S70. Alpha blockers Aurum product codes

Term from EMIS	Prod code ID	dmd ID	Drug substance name
Cardura 1mg tablets (Upjohn UK Ltd)	216741000033115	907711000001109	Doxazosin mesilate
Cardura 2mg tablets (Upjohn UK Ltd)	216841000033113	41811000001109	Doxazosin mesilate
Doxazosin 1mg tablets	480641000033111	318781001	Doxazosin mesilate
Doxazosin 2mg tablets	480741000033119	318782008	Doxazosin mesilate
Doxazosin 4mg tablets	480841000033112	318783003	Doxazosin mesilate
Doxazosin 4mg modified-release tablets	2273841000033111	39020411000001106	Doxazosin mesilate
Doxazosin 8mg modified-release tablets	2273941000033115	39021111000001107	Doxazosin mesilate
Cardura XL 4mg tablets (Upjohn UK Ltd)	2274041000033118	123911000001106	Doxazosin mesilate
Cardura XL 8mg tablets (Upjohn UK Ltd)	2274141000033119	873411000001109	Doxazosin mesilate
Cascor 2mg tablets (Ranbaxy (UK) Ltd)	2760041000033110	904511000001107	Doxazosin mesilate
Cascor 4mg tablets (Ranbaxy (UK) Ltd)	2760141000033114	179311000001107	Doxazosin mesilate
Doxadura 1mg tablets (Dexcel-Pharma Ltd)	2957941000033110	4857511000001108	Doxazosin mesilate
Doxadura 2mg tablets (Dexcel-Pharma Ltd)	2958041000033113	4857711000001103	Doxazosin mesilate
Doxadura 4mg tablets (Dexcel-Pharma Ltd)	2958141000033112	4858111000001103	Doxazosin mesilate
Slocinx XL 4mg tablets (Zentiva Pharma UK Ltd)	4021141000033118	11098311000001104	Doxazosin mesilate
Doxadura XL 4mg tablets (Dexcel-Pharma Ltd)	4063741000033112	11269911000001101	Doxazosin mesilate
Oxandosin XL 4mg tablets (Ratiopharm UK Ltd)	4288341000033111	11812211000001105	Doxazosin mesilate
Doxazosin 1mg/5ml oral suspension	4432941000033119	8483411000001101	Doxazosin mesilate
Doxzogen XL 4mg tablets (Mylan)	4575941000033115	13811611000001108	Doxazosin mesilate
Cardozin XL 4mg tablets (Almus Pharmaceuticals Ltd)	4936841000033112	18197411000001107	Doxazosin mesilate
Doxazosin 4mg/5ml oral suspension	5138341000033111	8483511000001102	Doxazosin mesilate
Colixil XL 4mg tablets (Sandoz Ltd)	5403641000033117	11757711000001107	Doxazosin mesilate
Larbex XL 4mg tablets (Teva UK Ltd)	5809641000033111	17338211000001104	Doxazosin mesilate
Raporsin XL 4mg tablets (Accord Healthcare Ltd)	6036141000033119	18164911000001108	Doxazosin mesilate
Doxazosin 4mg/5ml oral solution	8870641000033119	23466611000001105	Doxazosin mesilate
Doxazosin 1mg/5ml oral solution	9173941000033111	24509711000001103	Doxazosin mesilate
Doxazosin 8mg tablets	12682141000033115	421069003	Doxazosin mesilate
Rogitine 10mg/1ml solution for injection ampoules (Alliance Pharmaceuticals Ltd)	1180941000033115	4078811000001109	Phentolamine mesilate
Invicorp 1 solution for injection (Senetek Plc)	1619941000033111	5048711000001108	Aviptadil/ Phentolamine mesilate

Invicorp 2 solution for injection (Senetek Plc)	1620041000033114	5049011000001101	Aviptadil/ Phentolamine mesilate
Phentolamine 10mg/1ml solution for injection ampoules	3108541000033116	36015411000001100	Phentolamine mesilate
Phentolamine 2mg/0.35ml / Aviptadil 25micrograms/0.35ml solution for injection ampoules	10617641000033119	5049911000001102	Aviptadil/ Phentolamine mesilate
Invicorp 25micrograms/2mg/0.35ml solution for injection ampoules (Evolan Pharma AB)	10617741000033111	30737111000001103	Aviptadil/ Phentolamine mesilate
Baratol 25mg tablets (Amdipharm Plc)	116741000033118	3689111000001107	Indoramin hydrochloride
Doralese Tiltab 20mg tablets (Chemidex Pharma Ltd)	480541000033110	3354611000001100	Indoramin hydrochloride
Indoramin 20mg tablets	768541000033119	39696511000001106	Indoramin hydrochloride
Indoramin 25mg tablets	769841000033111	39696711000001101	Indoramin hydrochloride
Dibenyline 10mg capsules (Mercury Pharma Group Ltd)	438441000033119	3895211000001109	Phenoxybenzamine hydrochloride
Phenoxybenzamine 10mg capsules	1070341000033119	318745004	Phenoxybenzamine hydrochloride
Phenoxybenzamine 25mg/5ml oral suspension	2757941000033118	8671511000001109	Phenoxybenzamine hydrochloride
Alphavase 1 tablets (Ashbourne Pharmaceuticals Ltd)	37741000033118	934411000001100	Prazosin hydrochloride
Alphavase 2 tablets (Ashbourne Pharmaceuticals Ltd)	37841000033111	570011000001106	Prazosin hydrochloride
Alphavase 5 tablets (Ashbourne Pharmaceuticals Ltd)	37941000033115	74711000001102	Prazosin hydrochloride
Hypovase 500microgram tablets (Pfizer Ltd)	743541000033118	321311000001100	Prazosin hydrochloride
Hypovase 1mg tablets (Pfizer Ltd)	743641000033117	347411000001101	Prazosin hydrochloride
Hypovase 2mg tablets (Pfizer Ltd)	743741000033114	150911000001104	Prazosin hydrochloride
Prazosin 500microgram tablets	1131341000033118	318767003	Prazosin hydrochloride
Prazosin 1mg tablets	1131441000033112	318768008	Prazosin hydrochloride
Prazosin 2mg tablets	1131541000033113	318769000	Prazosin hydrochloride
Prazosin 5mg tablets	1131641000033114	318770004	Prazosin hydrochloride
Prazosin 500micrograms/5ml oral solution	11567441000033119	13078011000001104	Prazosin hydrochloride
Hytrin 10mg tablets (Advanz Pharma)	745641000033116	3150911000001107	Terazosin hydrochloride
Hytrin 2mg tablets (Advanz Pharma)	745741000033113	3147611000001100	Terazosin hydrochloride
Hytrin 5mg tablets (Advanz Pharma)	745841000033115	3154311000001103	Terazosin hydrochloride
Terazosin 2mg tablets	1425341000033119	318779003	Terazosin hydrochloride
Terazosin 10mg tablets	1429041000033118	318777001	Terazosin hydrochloride
Terazosin 5mg tablets	1429141000033119	318776005	Terazosin hydrochloride
Benph 5mg tablets (Mylan)	11807841000033119	33628611000001102	Terazosin hydrochloride

Table S71. Aldosterone Aurum product codes

Term from EMIS	Prod code ID	dmd ID	Drug substance name
Spironolactone 50mg/5ml oral suspension	1371341000033114	8726411000001105	Spironolactone
Spironolactone 25mg/5ml oral suspension	1371041000033112	8726511000001109	Spironolactone
Spironolactone 15mg/5ml oral suspension	5975441000033113	8727011000001103	Spironolactone
Spironolactone 10mg/5ml oral suspension	1371241000033116	8727311000001100	Spironolactone
Spironolactone 100mg/5ml oral suspension	2011541000033114	8727611000001105	Spironolactone
Spironolactone 25mg tablets	1374241000033118	318056008	Spironolactone
Aldactone 25mg tablets (Pfizer Ltd)	40541000033112	930511000001105	Spironolactone
Aldactone 50mg tablets (Pfizer Ltd)	40641000033113	921811000001103	Spironolactone
Spironolactone 50mg tablets	1374341000033111	318057004	Spironolactone
Spironolactone 100mg tablets	1374141000033113	318058009	Spironolactone
Spirospare 100 tablets (Ashbourne Pharmaceuticals Ltd)	1374541000033116	3411000001104	Spironolactone
Aldactone 100mg tablets (Pfizer Ltd)	40441000033111	421611000001100	Spironolactone
Lasilactone 20mg/50mg capsules (Sanofi)	808841000033119	3645811000001107	Furosemide/ Spironolactone
Spironolactone 50mg / Furosemide 20mg capsules	3162541000033115	318102003	Furosemide/ Spironolactone
Aldactide 25 tablets (Pfizer Ltd)	40241000033110	762511000001105	Hydroflumethiazide/ Spironolactone
Co-flumactone 25mg/25mg tablets	377041000033116	318127005	Hydroflumethiazide/ Spironolactone
Co-flumactone 50mg/50mg tablets	377141000033117	318128000	Hydroflumethiazide/ Spironolactone
Aldactide 50 tablets (Pfizer Ltd)	40341000033117	4669111000001107	Hydroflumethiazide/ Spironolactone
Spironolactone 250mg/5ml oral solution	5975541000033114	13353511000001101	Spironolactone
Spironolactone 250mg/5ml oral suspension	5975641000033110	13353611000001102	Spironolactone
Spironolactone 100mg/5ml oral solution	5999141000033111	13894611000001109	Spironolactone
Spironolactone 10mg/5ml oral solution	5999241000033116	13894711000001100	Spironolactone
Spironolactone 25mg/5ml oral solution	5890141000033112	13894811000001108	Spironolactone
Spironolactone 50mg/5ml oral solution	5890441000033116	13894911000001103	Spironolactone
Spironolactone 5mg/5ml oral solution	5999341000033114	13895011000001103	Spironolactone
Spironolactone 3mg / Chlorothiazide 30mg capsules	9278541000033112	16072811000001103	Chlorothiazide/ Spironolactone
Inspra 25mg tablets (Upjohn UK Ltd)	3199041000033116	8477211000001104	Eplerenone
Eplerenone 25mg tablets	3198841000033117	407011007	Eplerenone
Eplerenone 50mg tablets	3198941000033113	407012000	Eplerenone
Inspra 50mg tablets (Upjohn UK Ltd)	3199141000033117	8479811000001106	Eplerenone
Spironolactone 4mg / Chlorothiazide 40mg capsules	7681541000033117	18520511000001107	Chlorothiazide/ Spironolactone
Spironolactone 5mg/5ml oral suspension	1371141000033111	8726311000001103	Spironolactone

Table S72. Other antihypertensive Aurum product codes

Term from EMIS	Prod code ID	dmd ID	Drug substance name
Apraclonidine 1% eye drops 0.25ml unit dose preservative free	73541000033116	3958011000001107	Apraclonidine hydrochloride
Apraclonidine 5mg/ml eye drops	73641000033115	330873002	Apraclonidine hydrochloride
Catapres 150micrograms/1ml solution for injection ampoules (Glenwood GmbH)	196241000033111	364911000001108	Clonidine hydrochloride
Catapres 100microgram tablets (Glenwood GmbH)	215341000033112	215111000001101	Clonidine hydrochloride
Catapres 300microgram tablets (Boehringer Ingelheim Ltd)	215441000033118	368711000001103	Clonidine hydrochloride
Clonidine 250microgram modified-release capsules	261841000033118	36089511000001100	Clonidine hydrochloride
Clonidine 150micrograms/1ml solution for injection ampoules	275641000033110	36089211000001103	Clonidine hydrochloride
Clonidine 100microgram tablets	285841000033118	318667005	Clonidine hydrochloride
Clonidine 25microgram tablets	285941000033114	322840006	Clonidine hydrochloride
Clonidine 300microgram tablets	287241000033110	318668000	Clonidine hydrochloride
Dixarit 25microgram tablets (Boehringer Ingelheim Ltd)	464341000033111	344511000001105	Clonidine hydrochloride
Iopidine 1% eye drops 0.25ml unit dose (Novartis Pharmaceuticals UK Ltd)	771741000033114	3956211000001103	Apraclonidine hydrochloride
Iopidine 5mg/ml eye drops (Novartis Pharmaceuticals UK Ltd)	771841000033116	3799011000001100	Apraclonidine hydrochloride
Clonidine 200micrograms/24hours transdermal patches	5289041000033110	7660211000001104	Clonidine
Clonidine 50micrograms/5ml oral solution	5900141000033118	8398511000001100	Clonidine hydrochloride
Clonidine 50micrograms/5ml oral suspension	5900241000033113	8398611000001101	Clonidine hydrochloride
Clonidine 100micrograms/24hours transdermal patches	12106941000033117	10448511000001104	Clonidine
Clonidine 300micrograms/24hours transdermal patches	12107041000033116	10449111000001101	Clonidine
Catapres TTS 1 patches (Imported (Germany))	12107141000033117	10448811000001101	Clonidine
Catapres TTS 2 patches (Imported (Germany))	12117941000033118	7657311000001101	Clonidine
Catapres TTS 3 patches (Imported (Germany))	12118541000033113	10449311000001104	Clonidine
Clonidine 100micrograms/5ml oral suspension	12569041000033115	11813011000001109	Clonidine hydrochloride
Clonidine 50micrograms/5ml oral solution sugar free	12684041000033114	36392711000001102	Clonidine hydrochloride
Guanfacine 1mg modified-release tablets	10740841000033116	31087611000001109	Guanfacine hydrochloride
Guanfacine 2mg modified-release tablets	10740941000033112	31087711000001100	Guanfacine hydrochloride
Guanfacine 3mg modified-release tablets	10741041000033119	31087811000001108	Guanfacine hydrochloride

Guanfacine 4mg modified-release tablets	10741141000033115	31087911000001103	Guanfacine hydrochloride
Intuniv 1mg modified-release tablets (Takeda UK Ltd)	10741241000033110	31074011000001104	Guanfacine hydrochloride
Intuniv 2mg modified-release tablets (Takeda UK Ltd)	10741341000033117	31074311000001101	Guanfacine hydrochloride
Intuniv 3mg modified-release tablets (Takeda UK Ltd)	10741441000033111	31074611000001106	Guanfacine hydrochloride
Intuniv 4mg modified-release tablets (Takeda UK Ltd)	10741541000033112	31074911000001100	Guanfacine hydrochloride
Aldomet 250mg tablets (Aspen Pharma Trading Ltd)	40941000033118	253711000001107	Methyldopa anhydrous
Aldomet 500mg tablets (Aspen Pharma Trading Ltd)	41041000033111	73611000001108	Methyldopa anhydrous
Methyldopa 250mg/5ml oral suspension	892341000033112	8667311000001100	Methyldopa anhydrous
Methyldopa 125mg tablets	897341000033110	318671008	Methyldopa anhydrous
Methyldopa 250mg tablets	897441000033116	318672001	Methyldopa anhydrous
Methyldopa 500mg tablets	897541000033115	318673006	Methyldopa anhydrous
Moxonidine 200microgram tablets	938541000033115	318707000	Moxonidine
Moxonidine 400microgram tablets	938641000033119	318708005	Moxonidine
Physiotens 200microgram tablets (Mylan)	1080841000033119	41111000001102	Moxonidine
Physiotens 400microgram tablets (Mylan)	1080941000033110	142811000001107	Moxonidine
Moxonidine 300microgram tablets	2635641000033111	408604009	Moxonidine
Physiotens 300microgram tablets (Mylan)	2635741000033119	522011000001109	Moxonidine
Ganda 3 + 0.5 eye drops (Bausch & Lomb UK Ltd)	622441000033118	4668411000001105	Adrenaline/ Guanethidine monosulfate
Ganda 1 + 0.2 eye drops (Bausch & Lomb UK Ltd)	622641000033116	4668111000001100	Adrenaline/ Guanethidine monosulfate
Guanethidine 10mg/1ml solution for injection ampoules	655241000033111	36053311000001103	Guanethidine monosulfate
Ismelin 10mg/1ml solution for injection ampoules (Amdipharm Plc)	780741000033110	4369611000001106	Guanethidine monosulfate
Loniten 10mg tablets (Pfizer Ltd)	850441000033112	3666711000001100	Minoxidil
Loniten 2.5mg tablets (Pfizer Ltd)	850541000033113	3667011000001104	Minoxidil
Loniten 5mg tablets (Pfizer Ltd)	850641000033114	3666411000001106	Minoxidil
Minoxidil 10mg tablets	920641000033119	318657000	Minoxidil
Minoxidil 2.5mg tablets	920841000033118	318655008	Minoxidil
Minoxidil 5mg tablets	920941000033114	318656009	Minoxidil
Minoxidil 5% solution	921841000033111	36036311000001106	Minoxidil
Minoxidil 2% solution	921941000033115	36036211000001103	Minoxidil
Regaine for Women Regular Strength 2% solution (McNeil Products Ltd)	4026341000033113	9524011000001105	Minoxidil
Regaine for Men Regular Strength 2% solution (McNeil Products Ltd)	4026741000033114	9524811000001104	Minoxidil
Regaine for Men Extra Strength 5% scalp solution (Johnson & Johnson Ltd)	4026941000033112	9525311000001107	Minoxidil

Minoxidil 2% gel	4125241000033118	11472511000001105	Minoxidil
Regaine for Men 2% gel (McNeil Products Ltd)	4125341000033111	11465411000001105	Minoxidil
Regaine for Men Extra Strength 5% scalp foam (McNeil Products Ltd)	6448241000033118	18550611000001107	Minoxidil
Minoxidil 5% foam	6448341000033111	421143007	Minoxidil
Regaine for Women Once a Day 5% scalp foam (McNeil Products Ltd)	11803241000033110	32066411000001107	Minoxidil
Sodium nitroprusside 50mg powder for solution for infusion vials	1357141000033115	375899001	Sodium nitroprusside dihydrate
Apresoline 20mg powder for solution for injection ampoules (Advanz Pharma)	73441000033117	3925011000001100	Hydralazine hydrochloride
Apresoline 25mg tablets (Advanz Pharma)	74341000033114	657011000001101	Hydralazine hydrochloride
Hydralazine 20mg powder for solution for injection ampoules	734141000033114	34193811000001100	Hydralazine hydrochloride
Hydralazine 25mg tablets	741941000033113	318649003	Hydralazine hydrochloride
Hydralazine 50mg tablets	742041000033119	318650003	Hydralazine hydrochloride
Hydralazine 10mg/5ml oral suspension	4456241000033111	8528611000001105	Hydralazine hydrochloride
Hydralazine 25mg/5ml oral solution	11072741000033116	8580911000001106	Hydralazine hydrochloride
Hydralazine 10mg tablets	12877141000033119	414426001	Hydralazine hydrochloride
Aliskiren 150mg tablets	4213641000033112	39731411000001101	Aliskiren hemifumarate
Aliskiren 300mg tablets	4213741000033115	39731511000001102	Aliskiren hemifumarate
Rasilez 150mg tablets (Noden Pharma DAC)	4213841000033113	11960911000001108	Aliskiren hemifumarate
Rasilez 300mg tablets (Noden Pharma DAC)	4213941000033117	11961711000001103	Aliskiren hemifumarate

Table S73. Antiarrhythmic drugs class 1 Aurum product codes

Term from EMIS	Prod code ID	dmd ID	Drug substance name
Emla 5% cream (Aspen Pharma Trading Ltd)	511941000033116	2936311000001102	Lidocaine/ Prilocaine
Xylotox 2% E80 Dental injection 2.2ml cartridges (Dentsply Ltd)	1544441000033116	4207211000001108	Adrenaline acid tartrate/ Lidocaine hydrochloride
Lidoderm 5% patches (Teikoku Seiyaku)	2942141000033112	10570211000001105	Lidocaine
Solarcaine 0.5% gel (Schering-Plough Ltd)	2971941000033115	4011311000001103	Lidocaine hydrochloride
Glucose 5% / Lidocaine 500mg/500ml (0.1%) infusion 500ml bags	3096041000033116	5042311000001106	Glucose anhydrous/ Lidocaine hydrochloride
Glucose 5% / Lidocaine 1g/500ml (0.2%) infusion 500ml bags	3096141000033117	5042411000001104	Glucose anhydrous/ Lidocaine hydrochloride
Lidocaine 100mg/10ml (1%) solution for injection Minijet pre-filled syringes (UCB Pharma Ltd)	3096241000033112	4716611000001103	Lidocaine hydrochloride
Lidocaine 100mg/5ml (2%) solution for injection Minijet pre-filled syringes (UCB Pharma Ltd)	3096341000033119	9153311000001102	Lidocaine hydrochloride
Lignospan Special 20mg/ml / 12.5micrograms/ml solution for injection 2.2ml cartridges (Septodont Ltd)	4292941000033112	21313511000001103	Adrenaline acid tartrate/ Lidocaine hydrochloride
Lignospan Special 20mg/ml / 12.5micrograms/ml solution for injection 1.8ml cartridges (Septodont Ltd)	4418841000033118	35061911000001107	Adrenaline acid tartrate/ Lidocaine hydrochloride
Lidocaine 70mg / Tetracaine 70mg medicated plasters	4434341000033112	13210811000001108	Lidocaine/ Tetracaine
Rapydan 70mg/70mg medicated plasters (EUSA Pharma (UK) Ltd)	4434441000033118	13205811000001100	Lidocaine/ Tetracaine
Lidocaine 4% cream	4657441000033112	14203911000001107	Lidocaine
Denela 5% cream (Teva UK Ltd)	8840141000033111	23364211000001105	Lidocaine/ Prilocaine
Ralvo 700mg medicated plasters (Grunenthal Ltd)	12193941000033114	34178211000001102	Lidocaine
Lidocaine 200mg/20ml (1%) solution for injection vials	6132841000033115	3602411000001109	Lidocaine hydrochloride
Lidocaine 400mg/20ml (2%) solution for injection vials	6132641000033116	36039711000001100	Lidocaine hydrochloride
Nulbia 5% cream (Glenmark Pharmaceuticals Europe Ltd)	12421141000033115	34881811000001106	Lidocaine/ Prilocaine
Lidocaine 50mg/5ml (1%) solution for injection Mini-Plasco ampoules (B.Braun Medical Ltd)	12685841000033115	35863311000001108	Lidocaine hydrochloride
Lidocaine 200mg/20ml (1%) solution for injection Mini-Plasco ampoules (B.Braun Medical Ltd)	12685941000033111	35863611000001103	Lidocaine hydrochloride
Lidocaine 100mg/5ml (2%) solution for injection Mini-Plasco ampoules (B.Braun Medical Ltd)	12686041000033118	35863911000001109	Lidocaine hydrochloride
Lidocaine 400mg/20ml (2%) solution for injection Mini-Plasco ampoules (B.Braun Medical Ltd)	12686141000033119	35864311000001105	Lidocaine hydrochloride
Lidocaine 100mg/10ml (1%) solution for injection Mini-Plasco ampoules (B.Braun Medical Ltd)	12686341000033116	3768311000001105	Lidocaine hydrochloride
Lidocaine 200mg/10ml (2%) solution for injection Mini-Plasco ampoules (B.Braun Medical Ltd)	12686441000033110	4881511000001106	Lidocaine hydrochloride
Kinidin 250mg Durules (AstraZeneca UK Ltd)	804541000033110	3848511000001106	Quinidine bisulfate
Quinidine sulfate 200mg tablets	1149641000033114	318317007	Quinidine sulfate
Quinidine bisulfate 250mg modified-release tablets	1150941000033112	35933811000001102	Quinidine bisulfate

Procainamide 1g/10ml solution for injection vials	1119441000033119	34194111000001109	Procainamide hydrochloride
Procainamide 250mg capsules	5265241000033119	375342002	Procainamide hydrochloride
Disopyramide 100mg capsules	438841000033116	39690511000001109	Disopyramide
Disopyramide 150mg capsules	438941000033112	39690611000001108	Disopyramide
Disopyramide 50mg/5ml solution for injection ampoules	447241000033117	36070411000001104	Disopyramide phosphate
Disopyramide 150mg modified-release tablets	465241000033119	36070111000001109	Disopyramide phosphate
Disopyramide 250mg modified-release tablets	465341000033112	36070311000001106	Disopyramide phosphate
Dirythmin SA 150mg tablets (AstraZeneca UK Ltd)	469841000033118	3691511000001103	Disopyramide phosphate
Rythmodan 100mg capsules (Neon Healthcare Ltd)	1203941000033114	3465211000001104	Disopyramide
Rythmodan 150mg capsules (Cheplapharm Arzneimittel GmbH)	1204041000033111	3734711000001106	Disopyramide
Rythmodan 50mg/5ml solution for injection ampoules (Sanofi)	1204241000033115	4867311000001109	Disopyramide phosphate
Rythmodan Retard 250mg tablets (Neon Healthcare Ltd)	1205041000033112	3692111000001102	Disopyramide phosphate
Disopyramide 250mg modified-release capsules	2907741000033117	36070211000001103	Disopyramide phosphate
Isomide CR 250mg capsules (Tillomed Laboratories Ltd)	2907841000033110	3982011000001100	Disopyramide phosphate
Disopyramide 50mg/5ml oral solution	8770141000033110	8454511000001107	Disopyramide phosphate
Mexiletine 50mg capsules	872841000033111	318299008	Mexiletine hydrochloride
Mexitil 50mg capsules (Boehringer Ingelheim Ltd)	873041000033113	3877911000001101	Mexiletine hydrochloride
Mexitil PL Perlongets 360mg capsules (Boehringer Ingelheim Ltd)	873141000033112	4543311000001105	Mexiletine hydrochloride
Mexiletine 250mg/10ml solution for injection ampoules	879641000033116	36035711000001102	Mexiletine hydrochloride
Mexitil 250mg/10ml solution for injection ampoules (Boehringer Ingelheim Ltd)	879741000033113	4378311000001101	Mexiletine hydrochloride
Mexiletine 100mg capsules	8129541000033115	21394911000001108	Mexiletine hydrochloride
Mexiletine hydrochloride 200mg (Mexiletine 167mg) capsules	12888441000033116	36595811000001101	Mexiletine hydrochloride
Namuscla 167mg capsules (Lupin Healthcare (UK) Ltd)	12888541000033115	36538811000001103	Mexiletine hydrochloride
Flecainide 150mg/15ml solution for injection ampoules	587241000033119	36067511000001109	Flecainide acetate
Flecainide 50mg tablets	594541000033114	318276009	Flecainide acetate
Flecainide 100mg tablets	595141000033116	318277000	Flecainide acetate
Tambocor 150mg/15ml solution for injection ampoules (Teva UK Ltd)	1405841000033116	638511000001109	Flecainide acetate
Tambocor 50mg tablets (Teva UK Ltd)	1407441000033114	77511000001104	Flecainide acetate
Tambocor 100mg tablets (Teva UK Ltd)	1408041000033118	516411000001107	Flecainide acetate
Flecainide 25mg/5ml oral solution	2780641000033119	8514511000001105	Flecainide acetate
Flecainide 200mg modified-release capsules	4043141000033117	11169611000001105	Flecainide acetate
Tambocor XL 200mg capsules (Teva UK Ltd)	4043241000033112	11158611000001107	Flecainide acetate
Flecainide 10mg/5ml oral suspension	4432741000033117	8514411000001106	Flecainide acetate
Flecainide 25mg/5ml oral suspension	5993141000033114	8514611000001109	Flecainide acetate

Arythmol 150mg tablets (Mylan)	81441000033111	273811000001102	Propafenone hydrochloride
Arythmol 300mg tablets (Mylan)	81541000033112	69011000001108	Propafenone hydrochloride
Propafenone 150mg tablets	1140341000033110	318334004	Propafenone hydrochloride
Propafenone 300mg tablets	1140441000033116	318335003	Propafenone hydrochloride

Table S74. Antiarrhythmic drugs class 3 Aurum product codes

Term from EMIS	Prod code ID	dmd ID	Drug substance name
Amiodarone 150mg/3ml solution for injection ampoules	48341000033119	35900911000001106	Amiodarone hydrochloride
Amiodarone 100mg tablets	57641000033111	318186003	Amiodarone hydrochloride
Amiodarone 200mg tablets	57741000033119	318187007	Amiodarone hydrochloride
Cordarone X 100 tablets (Zentiva Pharma UK Ltd)	374641000033115	294711000001103	Amiodarone hydrochloride
Cordarone X 200 tablets (Zentiva Pharma UK Ltd)	374741000033112	269511000001109	Amiodarone hydrochloride
Amiodarone 300mg/10ml solution for injection pre-filled syringes	2644341000033117	35901011000001103	Amiodarone hydrochloride
Amyben 100mg tablets (Lexon (UK) Ltd)	2866241000033119	9108411000001107	Amiodarone hydrochloride
Amyben 200mg tablets (Lexon (UK) Ltd)	2866341000033112	9108711000001101	Amiodarone hydrochloride
Amiodarone 25mg/5ml oral suspension	3980241000033116	8275411000001105	Amiodarone hydrochloride
Amiodarone 100mg/5ml oral solution	3984941000033117	8274311000001102	Amiodarone hydrochloride
Amiodarone 200mg/5ml oral suspension	4505441000033115	8274611000001107	Amiodarone hydrochloride
Amiodarone 50mg/5ml oral solution	5897541000033119	8276111000001106	Amiodarone hydrochloride
Amiodarone 50mg/5ml oral suspension	5897641000033118	8276211000001100	Amiodarone hydrochloride
Amiodarone 100mg/5ml oral suspension	9774741000033117	8274411000001109	Amiodarone hydrochloride
Beta-Cardone 200mg tablets (Advanz Pharma)	143941000033116	447111000001104	Sotalol hydrochloride
Beta-Cardone 40mg tablets (Focus Pharmaceuticals Ltd)	144041000033119	900511000001103	Sotalol hydrochloride
Beta-Cardone 80mg tablets (Focus Pharmaceuticals Ltd)	144141000033115	104011000001101	Sotalol hydrochloride
Sotacor 40mg/4ml solution for injection ampoules (Bristol-Myers Squibb Pharmaceuticals Ltd)	1358541000033113	694711000001100	Sotalol hydrochloride
Sotacor 160mg tablets (Bristol-Myers Squibb Pharmaceuticals Ltd)	1366041000033112	446211000001108	Sotalol hydrochloride
Sotacor 80mg tablets (Neon Healthcare Ltd)	1366141000033111	463811000001109	Sotalol hydrochloride
Sotalol 160mg tablets	1367141000033113	318528007	Sotalol hydrochloride
Sotalol 200mg tablets	1367241000033118	318527002	Sotalol hydrochloride
Sotalol 40mg tablets	1367441000033117	318525005	Sotalol hydrochloride
Sotalol 80mg tablets	1367541000033116	318526006	Sotalol hydrochloride
Sotalol 40mg/4ml solution for injection ampoules	2908541000033114	35930811000001109	Sotalol hydrochloride
Sotalol 25mg/5ml oral solution	3953441000033116	8726811000001107	Sotalol hydrochloride
Sotalol 25mg/5ml oral suspension	9776441000033119	8726911000001102	Sotalol hydrochloride
Dronedarone 400mg tablets	5566541000033116	39691711000001107	Dronedarone hydrochloride
Multaq 400mg tablets (Sanofi)	5566641000033115	17066411000001106	Dronedarone hydrochloride

Table S75. Digoxin Aurum product codes

Term from EMIS	Prod code ID	dmd ID	Drug substance name
Azilsartan medoxomil 20mg tablets	7754841000033119	20418711000001104	Azilsartan medoxomil
Azilsartan medoxomil 40mg tablets	7754941000033110	449109006	Azilsartan medoxomil
Azilsartan medoxomil 80mg tablets	7755041000033110	449333009	Azilsartan medoxomil
Edarbi 20mg tablets (Takeda UK Ltd)	7755141000033114	20350911000001100	Azilsartan medoxomil
Edarbi 40mg tablets (Takeda UK Ltd)	7755241000033119	20351211000001103	Azilsartan medoxomil
Edarbi 80mg tablets (Takeda UK Ltd)	7755341000033112	20351811000001102	Azilsartan medoxomil
Amias 16mg tablets (Takeda UK Ltd)	59141000033113	908511000001100	Candesartan cilexetil
Amias 2mg tablets (Takeda UK Ltd)	59241000033118	97311000001103	Candesartan cilexetil
Amias 4mg tablets (Takeda UK Ltd)	59341000033111	857411000001100	Candesartan cilexetil
Amias 8mg tablets (Takeda UK Ltd)	59441000033117	36011000001106	Candesartan cilexetil
Candesartan 16mg tablets	211541000033119	318980005	Candesartan cilexetil
Candesartan 2mg tablets	211641000033118	318977009	Candesartan cilexetil
Candesartan 4mg tablets	211741000033110	318978004	Candesartan cilexetil
Candesartan 8mg tablets	211841000033117	318979007	Candesartan cilexetil
Candesartan 32mg tablets	3227241000033111	376998003	Candesartan cilexetil
Amias 32mg tablets (Takeda UK Ltd)	3227341000033118	8983911000001107	Candesartan cilexetil
Eprosartan 300mg tablets	2036341000033115	318994006	Eprosartan mesilate
Eprosartan 400mg tablets	2036441000033114	318995007	Eprosartan mesilate
Eprosartan 600mg tablets	2036541000033110	318996008	Eprosartan mesilate
Teveten 300mg tablets (Mylan)	2040841000033113	401211000001105	Eprosartan mesilate
Teveten 400mg tablets (Abbott Healthcare Products Ltd)	2040941000033117	151411000001103	Eprosartan mesilate
Teveten 600mg tablets (Mylan)	2041041000033110	872011000001109	Eprosartan mesilate
Aprovel 150mg tablets (Sanofi)	75941000033114	859711000001103	Irbesartan
Aprovel 300mg tablets (Sanofi)	76041000033116	323211000001107	Irbesartan
Aprovel 75mg tablets (Sanofi)	76141000033117	434511000001104	Irbesartan
Irbesartan 150mg tablets	775441000033117	318969005	Irbesartan
Irbesartan 300mg tablets	775541000033116	318970006	Irbesartan
Irbesartan 75mg tablets	775641000033115	318968002	Irbesartan
CoAprovel 150mg/12.5mg tablets (Sanofi)	2216141000033115	792411000001108	Hydrochlorothiazide/ Irbesartan
CoAprovel 300mg/12.5mg tablets (Sanofi)	2216241000033110	682711000001109	Hydrochlorothiazide/ Irbesartan
Irbesartan 150mg / Hydrochlorothiazide 12.5mg tablets	3161141000033116	134461004	Hydrochlorothiazide/ Irbesartan
Irbesartan 300mg / Hydrochlorothiazide 12.5mg tablets	3161241000033111	134460003	Hydrochlorothiazide/ Irbesartan
Irbesartan 300mg / Hydrochlorothiazide 25mg tablets	3995241000033112	10970311000001105	Hydrochlorothiazide/ Irbesartan
CoAprovel 300mg/25mg tablets (Sanofi)	3995341000033119	10968611000001106	Hydrochlorothiazide/ Irbesartan
Irbesartan 300mg/5ml oral suspension	4954341000033111	12639511000001103	Irbesartan

Irbesartan 150mg/5ml oral suspension	5970641000033117	8580811000001101	Irbesartan
Irbesartan 37.5mg oral powder sachets	6435741000033117	19481911000001105	Irbesartan
Ifirmasta 75mg tablets (Consilient Health Ltd)	8554141000033116	22720311000001108	Irbesartan
Ifirmasta 150mg tablets (Consilient Health Ltd)	8554241000033111	22720611000001103	Irbesartan
Ifirmasta 300mg tablets (Consilient Health Ltd)	8554341000033118	22720811000001104	Irbesartan
Cozaar 25mg tablets (Organon Pharma (UK) Ltd)	370441000033112	266511000001104	Losartan potassium
Cozaar 50mg tablets (Organon Pharma (UK) Ltd)	370541000033113	53611000001106	Losartan potassium
Cozaar-Comp 50mg/12.5mg tablets (Organon Pharma (UK) Ltd)	370841000033110	255911000001105	Hydrochlorothiazide/ Losartan potassium
Losartan 25mg tablets	851841000033117	318955005	Losartan potassium
Losartan 50mg tablets	851941000033113	318956006	Losartan potassium
Losartan 100mg tablets	2720141000033115	407784004	Losartan potassium
Cozaar 100mg tablets (Organon Pharma (UK) Ltd)	2720241000033110	245811000001102	Losartan potassium
Losartan 50mg / Hydrochlorothiazide 12.5mg tablets	3161741000033117	318959004	Hydrochlorothiazide/ Losartan potassium
Losartan 100mg / Hydrochlorothiazide 25mg tablets	3346841000033117	395497004	Hydrochlorothiazide/ Losartan potassium
Cozaar-Comp 100mg/25mg tablets (Organon Pharma (UK) Ltd)	3346941000033113	9566911000001105	Hydrochlorothiazide/ Losartan potassium
Losartan 100mg / Hydrochlorothiazide 12.5mg tablets	4424741000033110	13112711000001103	Hydrochlorothiazide/ Losartan potassium
Cozaar-Comp 100mg/12.5mg tablets (Organon Pharma (UK) Ltd)	4424841000033117	13094111000001102	Hydrochlorothiazide/ Losartan potassium
Losartan 12.5mg tablets	4957541000033114	15148111000001100	Losartan potassium
Cozaar 12.5mg tablets (Organon Pharma (UK) Ltd)	4957641000033110	15138911000001101	Losartan potassium
Losartan 2.5mg/ml oral suspension sugar free	5149941000033118	15507411000001105	Losartan potassium
Cozaar 2.5mg/ml oral suspension (Organon Pharma (UK) Ltd)	5150041000033110	15506811000001105	Losartan potassium
Losartan 100mg/5ml oral solution	5971741000033116	15451111000001107	Losartan potassium
Losartan 100mg/5ml oral suspension	5971841000033114	15451211000001101	Losartan potassium
Losartan 50mg/5ml oral solution	5971941000033118	14159411000001106	Losartan potassium
Losartan 50mg/5ml oral suspension	5972041000033112	14159511000001105	Losartan potassium
Olmesartan medoxomil 10mg tablets	2944541000033111	408055003	Olmesartan medoxomil
Olmesartan medoxomil 20mg tablets	2944641000033112	385542009	Olmesartan medoxomil
Olmesartan medoxomil 40mg tablets	2944741000033115	385543004	Olmesartan medoxomil
Olmetec 10mg tablets (Daiichi Sankyo UK Ltd)	2944841000033113	4624011000001101	Olmesartan medoxomil
Olmetec 20mg tablets (Daiichi Sankyo UK Ltd)	2944941000033117	4624311000001103	Olmesartan medoxomil
Olmetec 40mg tablets (Daiichi Sankyo UK Ltd)	2945041000033117	4624611000001108	Olmesartan medoxomil
Olmesartan medoxomil 20mg / Hydrochlorothiazide 12.5mg tablets	3908941000033112	409184002	Hydrochlorothiazide/ Olmesartan medoxomil
Olmesartan medoxomil 20mg / Hydrochlorothiazide 25mg tablets	3909041000033115	10270711000001105	Hydrochlorothiazide/ Olmesartan medoxomil
Olmetec Plus 20mg/12.5mg tablets (Daiichi Sankyo UK Ltd)	3909141000033116	10261511000001103	Hydrochlorothiazide/ Olmesartan medoxomil

Olmetec Plus 20mg/25mg tablets (Daiichi Sankyo UK Ltd)	3909241000033111	10261811000001100	Hydrochlorothiazide/ Olmesartan medoxomil
Olmesartan medoxomil 10mg/5ml oral suspension	4273241000033118	14680711000001103	Olmesartan medoxomil
Olmesartan medoxomil 20mg / Amlodipine 5mg tablets	5005841000033114	429502004	Amlodipine besilate/ Olmesartan medoxomil
Olmesartan medoxomil 40mg / Amlodipine 5mg tablets	5005941000033118	429503009	Amlodipine besilate/ Olmesartan medoxomil
Olmesartan medoxomil 40mg / Amlodipine 10mg tablets	5006041000033111	429678006	Amlodipine besilate/ Olmesartan medoxomil
Sevikar 20mg/5mg tablets (Daiichi Sankyo UK Ltd)	5006141000033110	15773211000001105	Amlodipine besilate/ Olmesartan medoxomil
Sevikar 40mg/5mg tablets (Daiichi Sankyo UK Ltd)	5006241000033115	15772911000001108	Amlodipine besilate/ Olmesartan medoxomil
Sevikar 40mg/10mg tablets (Daiichi Sankyo UK Ltd)	5006341000033113	15772611000001102	Amlodipine besilate/ Olmesartan medoxomil
Olmesartan medoxomil 40mg / Hydrochlorothiazide 12.5mg tablets	5566341000033111	409185001	Hydrochlorothiazide/ Olmesartan medoxomil
Olmetec Plus 40mg/12.5mg tablets (Daiichi Sankyo UK Ltd)	5566441000033117	17220911000001102	Hydrochlorothiazide/ Olmesartan medoxomil
Sevikar HCT 20mg/5mg/12.5mg tablets (Daiichi Sankyo UK Ltd)	6180641000033112	18986411000001108	Amlodipine besilate/ Hydrochlorothiazide/ Olmesartan medoxomil
Sevikar HCT 40mg/5mg/12.5mg tablets (Daiichi Sankyo UK Ltd)	6180741000033115	18986711000001102	Amlodipine besilate/ Hydrochlorothiazide/ Olmesartan medoxomil
Sevikar HCT 40mg/10mg/12.5mg tablets (Daiichi Sankyo UK Ltd)	6180841000033113	18987011000001101	Amlodipine besilate/ Hydrochlorothiazide/ Olmesartan medoxomil
Sevikar HCT 40mg/5mg/25mg tablets (Daiichi Sankyo UK Ltd)	6180941000033117	18987311000001103	Amlodipine besilate/ Hydrochlorothiazide/ Olmesartan medoxomil
Sevikar HCT 40mg/10mg/25mg tablets (Daiichi Sankyo UK Ltd)	6181041000033110	18987611000001108	Amlodipine besilate/ Hydrochlorothiazide/ Olmesartan medoxomil
Diovan 160mg capsules (Novartis Pharmaceuticals UK Ltd)	437641000033118	117011000001107	Valsartan
Diovan 40mg capsules (Novartis Pharmaceuticals UK Ltd)	437741000033110	777611000001101	Valsartan
Diovan 80mg capsules (Novartis Pharmaceuticals UK Ltd)	437841000033117	554511000001105	Valsartan
Valsartan 160mg capsules	1498241000033111	318963006	Valsartan
Valsartan 40mg capsules	1498341000033118	318961008	Valsartan
Valsartan 80mg capsules	1498441000033112	318962001	Valsartan
Co-Diovan 160mg/12.5mg tablets (Novartis Pharmaceuticals UK Ltd)	3152541000033112	7668611000001104	Hydrochlorothiazide/ Valsartan
Co-Diovan 160mg/25mg tablets (Novartis Pharmaceuticals UK Ltd)	3152641000033113	7668911000001105	Hydrochlorothiazide/ Valsartan

Valsartan 160mg / Hydrochlorothiazide 12.5mg tablets	3161441000033112	395521005	Hydrochlorothiazide/ Valsartan
Valsartan 160mg / Hydrochlorothiazide 25mg tablets	3161541000033113	409298002	Hydrochlorothiazide/ Valsartan
Valsartan 80mg / Hydrochlorothiazide 12.5mg tablets	3190741000033112	377488008	Hydrochlorothiazide/ Valsartan
Co-Diovan 80mg/12.5mg tablets (Novartis Pharmaceuticals UK Ltd)	3190841000033119	8150111000001108	Hydrochlorothiazide/ Valsartan
Valsartan 40mg tablets	3201341000033115	416515008	Valsartan
Diovan 40mg tablets (Novartis Pharmaceuticals UK Ltd)	3201441000033114	8263211000001101	Valsartan
Amlodipine 5mg / Valsartan 80mg tablets	4021441000033114	11160311000001109	Amlodipine besilate/ Valsartan
Amlodipine 5mg / Valsartan 160mg tablets	4021541000033110	11160211000001101	Amlodipine besilate/ Valsartan
Amlodipine 10mg / Valsartan 160mg tablets	4021641000033111	11160111000001107	Amlodipine besilate/ Valsartan
Exforge 5mg/80mg tablets (Novartis Pharmaceuticals UK Ltd)	4021741000033119	11161811000001108	Amlodipine besilate/ Valsartan
Exforge 5mg/160mg tablets (Novartis Pharmaceuticals UK Ltd)	4021841000033112	11161511000001105	Amlodipine besilate/ Valsartan
Exforge 10mg/160mg tablets (Novartis Pharmaceuticals UK Ltd)	4021941000033116	11160711000001108	Amlodipine besilate/ Valsartan
Valsartan 320mg tablets	4424941000033113	376487009	Valsartan
Diovan 320mg tablets (Novartis Pharmaceuticals UK Ltd)	4425041000033113	13143311000001102	Valsartan
Valsartan 80mg tablets	6515441000033113	375034009	Valsartan
Valsartan 160mg tablets	6515541000033114	375035005	Valsartan
Valsartan 3mg/ml oral solution	6528441000033113	20007411000001100	Valsartan
Diovan 3mg/1ml oral solution (Novartis Pharmaceuticals UK Ltd)	6528541000033114	20001711000001102	Valsartan
Sacubitril 24mg / Valsartan 26mg tablets	10943641000033117	31142011000001103	Sacubitril/ Valsartan
Sacubitril 49mg / Valsartan 51mg tablets	10943741000033114	31142111000001102	Sacubitril/ Valsartan
Sacubitril 97mg / Valsartan 103mg tablets	10943841000033116	31142211000001108	Sacubitril/ Valsartan
Entresto 24mg/26mg tablets (Novartis Pharmaceuticals UK Ltd)	10943941000033112	31136811000001109	Sacubitril/ Valsartan
Entresto 49mg/51mg tablets (Novartis Pharmaceuticals UK Ltd)	10944041000033114	31136411000001107	Sacubitril/ Valsartan
Entresto 97mg/103mg tablets (Novartis Pharmaceuticals UK Ltd)	10944141000033113	31138011000001104	Sacubitril/ Valsartan

Table S76. Sodium-glucose co-transporter-2 inhibitors Aurum product codes

Term from EMIS	Prod code ID	dmd ID	Drug substance name
Forxiga 5mg tablets (AstraZeneca UK Ltd)	8199541000033113	21609511000001105	Dapagliflozin propanediol monohydrate
Dapagliflozin 5mg tablets	8199341000033118	703679006	Dapagliflozin propanediol monohydrate
Dapagliflozin 10mg tablets	8199441000033112	703680009	Dapagliflozin propanediol monohydrate
Forxiga 10mg tablets (AstraZeneca UK Ltd)	8199641000033114	21609811000001108	Dapagliflozin propanediol monohydrate
Xigduo 5mg/1000mg tablets (AstraZeneca UK Ltd)	9106241000033119	24018511000001102	Dapagliflozin propanediol monohydrate/ Metformin hydrochloride
Dapagliflozin 5mg / Metformin 1g tablets	9106041000033110	24054611000001100	Dapagliflozin propanediol monohydrate/ Metformin hydrochloride
Dapagliflozin 5mg / Metformin 850mg tablets	9106141000033114	24054711000001109	Dapagliflozin propanediol monohydrate/ Metformin hydrochloride
Xigduo 5mg/850mg tablets (AstraZeneca UK Ltd)	9106341000033112	24018111000001106	Dapagliflozin propanediol monohydrate/ Metformin hydrochloride
Saxagliptin 5mg / Dapagliflozin 10mg tablets	11898041000033111	33745311000001109	Dapagliflozin propanediol monohydrate/ Saxagliptin hydrochloride
Qtern 5mg/10mg tablets (AstraZeneca UK Ltd)	11898141000033110	33682311000001103	Dapagliflozin propanediol monohydrate/ Saxagliptin hydrochloride
Jardiance 10mg tablets (Boehringer Ingelheim Ltd)	9337141000033114	25238811000001107	Empagliflozin
Empagliflozin 10mg tablets	9336641000033119	25290511000001101	Empagliflozin
Empagliflozin 25mg tablets	9336841000033118	25290611000001102	Empagliflozin
Jardiance 25mg tablets (Boehringer Ingelheim Ltd)	9337241000033119	25239711000001108	Empagliflozin
Empagliflozin 12.5mg / Metformin 1g tablets	10614441000033112	30318111000001109	Empagliflozin/ Metformin hydrochloride
Synjardy 12.5mg/1000mg tablets (Boehringer Ingelheim Ltd)	10614841000033110	30175711000001100	Empagliflozin/ Metformin hydrochloride
Synjardy 12.5mg/850mg tablets (Boehringer Ingelheim Ltd)	10614741000033117	30175011000001102	Empagliflozin/ Metformin hydrochloride
Empagliflozin 12.5mg / Metformin 850mg tablets	10614341000033118	30318211000001103	Empagliflozin/ Metformin hydrochloride

Empagliflozin 5mg / Metformin 1g tablets	10614241000033111	30318311000001106	Empagliflozin/ Metformin hydrochloride
Synjardy 5mg/1000mg tablets (Boehringer Ingelheim Ltd)	10614641000033114	30174111000001100	Empagliflozin/ Metformin hydrochloride
Synjardy 5mg/850mg tablets (Boehringer Ingelheim Ltd)	10614541000033113	30173411000001107	Empagliflozin/ Metformin hydrochloride
Empagliflozin 5mg / Metformin 850mg tablets	10614141000033116	30318411000001104	Empagliflozin/ Metformin hydrochloride
Empagliflozin 10mg / Linagliptin 5mg tablets	13116741000033112	37280311000001109	Empagliflozin/ Linagliptin
Glyxambi 10mg/5mg tablets (Boehringer Ingelheim Ltd)	13116941000033110	37225211000001100	Empagliflozin/ Linagliptin
Glyxambi 25mg/5mg tablets (Boehringer Ingelheim Ltd)	13117041000033111	37225511000001102	Empagliflozin/ Linagliptin
Empagliflozin 25mg / Linagliptin 5mg tablets	13116841000033119	37280511000001103	Empagliflozin/ Linagliptin
Canagliflozin 100mg tablets	9110341000033112	703682001	Canagliflozin hemihydrate
Invokana 100mg tablets (Napp Pharmaceuticals Ltd)	9110541000033117	24088611000001101	Canagliflozin hemihydrate
Canagliflozin 300mg tablets	9110441000033118	24104511000001103	Canagliflozin hemihydrate
Invokana 300mg tablets (Napp Pharmaceuticals Ltd)	9110641000033116	24088311000001106	Canagliflozin hemihydrate
Canagliflozin 50mg / Metformin 1g tablets	9851541000033114	28049211000001101	Canagliflozin hemihydrate/ Metformin hydrochloride
Vokanamet 50mg/1000mg tablets (Napp Pharmaceuticals Ltd)	9851841000033111	28024411000001103	Canagliflozin hemihydrate/ Metformin hydrochloride
Vokanamet 50mg/850mg tablets (Napp Pharmaceuticals Ltd)	9851741000033118	28022511000001109	Canagliflozin hemihydrate/ Metformin hydrochloride
Canagliflozin 50mg / Metformin 850mg tablets	9851641000033110	28049311000001109	Canagliflozin hemihydrate/ Metformin hydrochloride

Table S77. Nonsteroidal anti-inflammatory drug Aurum product codes

Term from EMIS	Prod code ID	dmd ID	Drug substance name
Aspirin 75mg dispersible tablets	82941000033113	319773006	Aspirin
PostMI 75 dispersible tablets (Ashbourne Pharmaceuticals Ltd)	1098041000033111	636011000001100	Aspirin
Danamep 75mg dispersible tablets (Ecogen Europe Ltd)	11716941000033112	32968711000001107	Aspirin
Mandaprin 75mg dispersible tablets (M & A Pharmachem Ltd)	12684641000033115	35581511000001100	Aspirin
Angettes 75 tablets (Bristol-Myers Squibb Pharmaceuticals Ltd)	71241000033119	800511000001106	Aspirin
Aspirin 75mg tablets	87041000033110	319775004	Aspirin
Aspirin 75mg gastro-resistant tablets	83041000033115	319781007	Aspirin
Caprin 75mg gastro-resistant tablets (Wockhardt UK Ltd)	189841000033119	927411000001108	Aspirin
Enprin 75mg gastro-resistant tablets (Galpharm International Ltd)	1729141000033110	473711000001109	Aspirin
Nu-Seals 75 gastro-resistant tablets (Alliance Pharmaceuticals Ltd)	991441000033115	414311000001102	Aspirin
Micropirin 75mg gastro-resistant tablets (Dexcel-Pharma Ltd)	2273741000033118	281311000001104	Aspirin
PostMI 75 EC tablets (Ashbourne Pharmaceuticals Ltd)	1101541000033119	38411000001101	Aspirin
Combogesic 500mg/150mg tablets (Thornton & Ross Ltd)	11781641000033113	33563011000001103	Ibuprofen/ Paracetamol
Paracetamol 500mg / Ibuprofen 150mg tablets	11781541000033112	33568211000001100	Ibuprofen/ Paracetamol
Ibuprofen lysine 400mg tablets	5376641000033111	10245111000001102	Ibuprofen lysine
Nurofen Maximum Strength Migraine Pain 684mg caplets (Reckitt Benckiser Healthcare (UK) Ltd)	5376741000033119	10239611000001109	Ibuprofen lysine
Caprin 300mg gastro-resistant tablets (Pinewood Healthcare)	212341000033117	901211000001107	Aspirin
Nu-Seals 300 gastro-resistant tablets (Alliance Pharmaceuticals Ltd)	993341000033113	183311000001101	Aspirin
Aspirin 300mg gastro-resistant tablets	83141000033116	322225002	Aspirin
Aspirin 300mg suppositories	2013341000033115	322232006	Aspirin
Aspirin 150mg suppositories	2013441000033114	322233001	Aspirin
Co-codaprin 8mg/400mg dispersible tablets	371041000033112	322310004	Aspirin/ Codeine phosphate
Aspav dispersible tablets (Actavis UK Ltd)	86841000033118	3690911000001103	Aspirin/ Papaveretum
Aspirin 500mg / Papaveretum 7.71mg dispersible tablets sugar free	3849841000033110	322321008	Aspirin/ Papaveretum
MigraMax oral powder sachets (Zentiva)	2086841000033117	3637911000001108	Aspirin DL-Lysine/ Metoclopramide hydrochloride
Aspirin 900mg / Metoclopramide 10mg oral powder sachets sugar free	3836841000033119	322784004	Aspirin DL-Lysine/ Metoclopramide hydrochloride
Brufen Retard 800mg tablets (Mylan)	159441000033110	368011000001100	Ibuprofen
Ibuprofen 800mg modified-release tablets	746941000033117	39110311000001101	Ibuprofen
Ibuprofen 200mg / Phenylephrine 6.1mg tablets	12496141000033117	34878411000001108	Ibuprofen/ Phenylephrine

Bisoprolol 10mg / Aspirin 100mg capsules	10025241000033112	28365311000001102	Aspirin/ Bisoprolol fumarate
Bisoprolol 10mg / Aspirin 75mg capsules	10025141000033117	28365411000001109	Aspirin/ Bisoprolol fumarate
Bisoprolol 5mg / Aspirin 100mg capsules	10025041000033116	28365611000001107	Aspirin/ Bisoprolol fumarate
Bisoprolol 5mg / Aspirin 75mg capsules	10024941000033116	28365711000001103	Aspirin/ Bisoprolol fumarate
Co-codaprin 8mg/400mg tablets	370941000033119	11762111000001100	Aspirin/ Codeine phosphate
Imazin XL forte tablets (Napp Pharmaceuticals Ltd)	1739641000033115	3461511000001104	Aspirin/ Isosorbide mononitrate
Aspirin 150mg / Isosorbide mononitrate 60mg modified-release tablets	3162141000033112	35903011000001102	Aspirin/ Isosorbide mononitrate
Imazin XL tablets (Napp Pharmaceuticals Ltd)	1739541000033116	3460911000001104	Aspirin/ Isosorbide mononitrate
Aspirin 75mg / Isosorbide mononitrate 60mg modified-release tablets	3162041000033113	35903111000001101	Aspirin/ Isosorbide mononitrate
Nurofen Meltlets 200mg tablets (Reckitt Benckiser Healthcare (UK) Ltd)	2839541000033115	3869111000001103	Ibuprofen
Ibuprofen 200mg orodispersible tablets sugar free	2839441000033116	3875511000001104	Ibuprofen
Aspirin 300mg tablets	86641000033119	329525004	Aspirin
Aspirin 300mg dispersible tablets	82841000033117	329526003	Aspirin
Ibuprofen 200mg tablets	747341000033119	329652003	Ibuprofen
Cuprofen 200mg tablets (SSL International Plc)	4432641000033114	640911000001100	Ibuprofen
Brufen 200mg tablets (Abbott Laboratories Ltd)	162841000033113	643711000001106	Ibuprofen
Nurofen 200mg caplets (Reckitt Benckiser Healthcare (UK) Ltd)	2970741000033111	621911000001109	Ibuprofen
Arthrofen 200 tablets (Ashbourne Pharmaceuticals Ltd)	81641000033113	389511000001100	Ibuprofen
Librofem 200mg tablets (LPC Medical (UK) Ltd)	1627241000033117	901911000001103	Ibuprofen
Advil 200mg tablets (Wyeth Consumer Healthcare)	2968941000033119	8970311000001109	Ibuprofen
Hedex Ibuprofen 200mg tablets (Omega Pharma Ltd)	2968541000033113	17042011000001106	Ibuprofen
Motrin 400mg tablets (Pfizer Ltd)	939741000033119	868911000001103	Ibuprofen
Arthrofen 400 tablets (Ashbourne Pharmaceuticals Ltd)	81741000033116	933011000001107	Ibuprofen
Brufen 400mg tablets (Mylan)	162941000033117	263211000001109	Ibuprofen
Ibuprofen 400mg tablets	747441000033113	329653008	Ibuprofen
Ibuprofen 600mg tablets	747541000033114	329654002	Ibuprofen
Brufen 600mg tablets (Mylan)	163041000033110	230111000001100	Ibuprofen
Arthrofen 600 tablets (Ashbourne Pharmaceuticals Ltd)	81841000033114	415611000001108	Ibuprofen
Motrin 600mg tablets (Pfizer Ltd)	939841000033112	550011000001109	Ibuprofen
Ibuprofen 200mg / Codeine 12.8mg tablets	12708841000033116	329683004	Codeine phosphate/ Ibuprofen

Nurofen Plus tablets (Reckitt Benckiser Healthcare (UK) Ltd)	2228341000033113	3915411000001104	Codeine phosphate/ Ibuprofen
Solpaflex tablets (GlaxoSmithKline Consumer Healthcare)	2968641000033114	3916211000001109	Codeine phosphate/ Ibuprofen
Cuprofen PLUS tablets (SSL International Plc)	3077941000033110	8092711000001106	Codeine phosphate/ Ibuprofen
Motrin 800mg tablets (Pfizer Ltd)	939941000033116	62911000001102	Ibuprofen
Ibuprofen 800mg tablets	746541000033111	329708004	Ibuprofen
Ibuprofen lysine 200mg tablets	4661241000033110	4557011000001104	Ibuprofen lysine
Disprin CV 300mg modified-release tablets (Reckitt Benckiser Healthcare (UK) Ltd)	452541000033115	4532311000001104	Aspirin
Aspirin 300mg modified-release tablets	84441000033114	4558011000001103	Aspirin
Maximum Strength Aspro Clear 500mg effervescent tablets (Bayer Plc)	4431941000033114	4648711000001109	Aspirin
Aspro Clear 300mg effervescent tablets (Bayer Plc)	4431841000033118	4649111000001101	Aspirin
Codafen Continus tablets (Napp Pharmaceuticals Ltd)	336741000033117	3420111000001109	Codeine phosphate/ Ibuprofen
Ibuprofen 300mg modified-release / Codeine 20mg tablets	3229141000033111	36045211000001108	Codeine phosphate/ Ibuprofen
Aspirin 500mg / Codeine 8mg dispersible tablets sugar free	3934341000033112	4956111000001107	Aspirin/ Codeine phosphate
Codis 500 dispersible tablets (Reckitt Benckiser Healthcare (UK) Ltd)	3934441000033118	4951811000001105	Aspirin/ Codeine phosphate
Aspirin powder	2085241000033111	5145711000001107	Aspirin
Asasantin Retard capsules (Boehringer Ingelheim Ltd)	1571541000033117	3292511000001108	Aspirin/ Dipyridamole
Dipyridamole 200mg modified-release / Aspirin 25mg capsules	3160741000033110	36069911000001109	Aspirin/ Dipyridamole
Atransipar 200mg/25mg modified-release capsules (Par Laboratories Europe Ltd)	12352041000033117	34609311000001105	Aspirin/ Dipyridamole
Molita 200mg/25mg modified-release capsules (Dr Reddy's Laboratories (UK) Ltd)	8619141000033116	23241511000001106	Aspirin/ Dipyridamole
Ibuprofen 200mg / Pseudoephedrine hydrochloride 30mg tablets	4123241000033119	407904005	Ibuprofen/ Pseudoephedrine hydrochloride
Nurofen Cold and Flu tablets (Reckitt Benckiser Healthcare (UK) Ltd)	4123741000033113	4606211000001109	Ibuprofen/ Pseudoephedrine hydrochloride
Dexibuprofen 300mg tablets	3334141000033116	418352003	Dexibuprofen
Seractil 300mg tablets (Gebro Pharma GmbH)	3334341000033118	35096411000001107	Dexibuprofen
Dexibuprofen 400mg tablets	3334241000033111	418855005	Dexibuprofen
Seractil 400mg tablets (Thornton & Ross Ltd)	3334441000033112	9447611000001101	Dexibuprofen
Nuromol 200mg/500mg tablets (Reckitt Benckiser Healthcare (UK) Ltd)	6386841000033117	18594011000001100	Ibuprofen/ Paracetamol
Paracetamol 500mg / Ibuprofen 200mg tablets	6386741000033110	18595211000001106	Ibuprofen/ Paracetamol
