# Supplementary material for: Systematic approach to outcome assessment from coded electronic healthcare records in the DaRe2THINK NHS-embedded randomized trial
Source: Eur Heart J Digit Health. 2022 Sep 16;3(3):426–36. doi: 10.1093/ehjdh/ztac046 (PMC9708037; doi:10.1093/ehjdh/ztac046)
Supplement: ztac046_Supplementary_Data [file ztac046_supplementary_data.zip › DaRe2THINK_protocol_v3.0_27Jan2022.pdf]

OPEN ACCESS AFTER REC APPROVAL

PROTOCOL TITLE

Preventing stroke, premature death and cognitive decline in a broader community of patients with atrial fibrillation using healthcare data for pragmatic research: A randomised controlled trial

SHORT STUDY TITLE / ACRONYM

DaRe2THINK

PROTOCOL VERSION NUMBER AND DATE

Version 3.0; 27 Jan 2022

[This protocol has regard for the HRA guidance]

SPONSOR

University of Birmingham, UK

FUNDER

National Institute for Health Research (HTA 19/109 - NIHR130280)

RESEARCH REFERENCE NUMBERS

**Sponsor Number:**	RG_20-141
**IRAS ID:**	290420
**REC Number:**	21/NE/0021
**Clinicaltrials.gov:**	NCT04700826
**ISRCTN:**	ISRCTN21157803
**EudraCT Number:**	2020-005774-10

COMPLIANCE STATEMENT

This protocol describes the DaRe2THINK trial only. The protocol should not be used as a guide for the treatment of patients not taking part in DaRe2THINK.

The study will be conducted in compliance with the latest versions of the approved protocol, UK Policy Framework for Health and Social Care Research, Data Protection legislation, and the principals of Good Clinical Practice.

Whilst every care has been taken during the drafting of this protocol, future amendments may be necessary which will require approvals prior to implementation.

OPEN ACCESS AFTER REC APPROVAL

PROTOCOL DEVELOPMENT AND SIGN OFF

Protocol Amendments

The following amendments and/or administrative changes have been made to this protocol since the implementation of the first approved version:

Amendment number	Date of amendment	Protocol version number	Type of amendment	Summary of amendment
1	17 Jun 2021	2.0	Substantial	1. Clarification of exclusion criterion for women based on latest NICE guidance.<br>2. Addition of exclusion criterion for patients already enrolled in a Clinical trial.<br>3. Clarification of per protocol and per treatment analysis.<br>4. Addition of imaging sub-study.<br>5. Other minor/non-substantial corrections.
2	27 Jan 2021	3.0	Substantial	1. Option for Primary Care investigators to send out text messages via NHS clinical systems to potentially eligible participants.<br>2. Addition of mechanism for potential study participants to contact the central study team after invitation from Primary Care investigators.<br>3. Addition of new links to NICE prescribing support for anticoagulation.<br>4. Update to Key Study Contacts page.

CHIEF INVESTIGATOR & STATISTICIAN SIGNATURE PAGE

The undersigned confirm that the following protocol has been agreed and accepted, and that the study will be conducted in compliance with the approved protocol and will adhere to the principles outlined in the Medicines for Human Use (Clinical Trials) Regulations 2004 (SI 2004/1031), amended regulations (SI 2006/1928) and any subsequent amendments of the clinical trial regulations, GCP guidelines, the Sponsor's (and any other relevant) Standard Operating Procedures, and other regulatory requirements as amended.

I agree to ensure that the confidential information contained in this document will not be used for any other purpose other than the evaluation or conduct of the investigation without the prior written consent of the Sponsor.

I also confirm that I will make the findings of the study publicly available through publication or other dissemination tools without any unnecessary delay and that an honest accurate and transparent account of the study will be given; and that any discrepancies from the study as planned in this protocol will be explained.

Chief Investigator:

Signature: Signature Date:

..../....../......

Name (please print): Protocol Version: Protocol Date:

.... ………………… ....../....../......

Trial Statistician:

Signature: Signature Date:

..../....../......

Name (please print): Protocol Version: Protocol Date:

.... ………………… ....../....../......

Sponsor Statement

Where the University of Birmingham takes on the sponsor role for protocol development oversight, the signing of the IRAS form by the Sponsor will serve as confirmation of the approval of this protocol.

PRINCIPAL INVESTIGATOR SIGNATURE PAGE

I have read and agree to the protocol, as detailed in this document. I agree to adhere to the protocol as outlined and agree that any suggested changes to the protocol must be approved by the Trial Steering Committee prior to seeking approval from the Research Ethics Committee and Regulatory Authority.

I am aware of my responsibilities as an Investigator under the guidelines of Good Clinical Practice (GCP), the Declaration of Helsinki, local regulations (as applicable) and the trial protocol and I agree to conduct the trial according to these guidelines and to appropriately direct and assist the staff under my control who will be involved in the trial.

Site Principal Investigator:

Signature: Signature Date:

..../....../......

Name (please print): Protocol Version: Protocol Date:

.... …………………. ....../....../......

Site Name and Postcode (please print):

....

....

TABLE OF CONTENTS

**PROTOCOL TITLE**		**1**
**PROTOCOL DEVELOPMENT AND SIGN OFF**		**2**
**CHIEF INVESTIGATOR & STATISTICIAN SIGNATURE PAGE**		**3**
**PRINCIPAL INVESTIGATOR SIGNATURE PAGE**		**4**
**TABLE OF CONTENTS**		**5**
**KEY STUDY CONTACTS**		**7**
**STUDY SUMMARY**		**9**
**ABBREVIATIONS**		**11**
**PLAIN ENGLISH SUMMARY**		**12**
**SCIENTIFIC ABSTRACT**		**13**
**TRIAL FLOW CHART**		**14**
**1**	**BACKGROUND, RATIONALE & AIMS**	**15**
	1.1 The burden of atrial fibrillation	*15*
	1.2 The need for new trials	*15*
	1.3 A joined-up approach across the NHS	*16*
	1.4 The DaRe2 trial pipeline	*16*
	1.5 Application to patients with AF and beyond	*17*
**2**	**DESIGN CONSIDERATIONS**	**18**
	2.1 Design summary	*18*
	2.2 Assessment of risk for control patients	*18*
	2.3 Assessment of risk for anticoagulated patients	*18*
	2.4 Hypotheses for primary, key secondary & health economic outcomes	*19*
	2.5 Hypotheses for other secondary outcomes	*19*
	2.6 Overarching challenge and opportunity	*19*
**3**	**STUDY METHODS**	**20**
	3.1 Approach to study selection criteria	*20*
	3.2 Study participant inclusion criteria	*20*
	3.3 Study participant exclusion criteria	*20*
	3.4 General Practice selection criteria	*21*
	3.5 Participant identification and Screening	*21*
	3.6 Consent to contact	*22*
	3.7 Consent procedures for trial enrolment	*22*
	3.8 Randomisation	*24*
	3.9 Trial treatments	*24*
	3.10 Blinding	*26*
	3.11 Internal pilot	*26*
	3.12 Withdrawal	*28*
	3.13 Change of Primary Care provider	*28*
	3.14 Treatment duration and End of Trial	*29*
**4**	**OUTCOMES**	**30**
	4.1 Timing & assessment of outcomes	*30*
	4.2 Primary outcome	*30*
	4.3 Secondary outcomes	*30*
	4.4 Cost-effectiveness outcomes	*30*
	4.5 Process outcomes	*31*
	4.6 Rationale for outcomes	*31*
**5**	**DATA MANAGEMENT**	**32**
	5.1 Baseline and follow-up data	*32*
	5.2 Quality of CPRD data	*32*
	5.3 Validation of clinical events	*33*
	5.4 Patient-reported cognitive function	*35*
	5.5 Patient-reported quality of life	*36*
	5.6 Additional patient-reported events	*37*
	5.7 Coordination of data processes	*37*
**6**	**SAFETY REPORTING**	**38**
	6.1 Overview	*38*
	6.2 Definitions	*38*

	6.3 Operational use within a pragmatic NHS-embedded trial	*39*
	6.4 Reporting of SUSARs	*40*
	6.5 Safety net to capture adverse events	*41*
	6.6 Responsibilities of the Principal Investigator	*41*
	6.7 Responsibilities of the Clinical Practice Research Datalink team	*42*
	6.8 Responsibilities of the Chief Investigator/ Deputy Chief Investigator or delegate	*42*
	6.9 Responsibilities of the Sponsor	*43*
	6.10 Responsibilities of the oversight committees	*43*
**7**	**STATISTICAL CONSIDERATIONS**	**44**
	7.1 Sample size derivation	*44*
	7.2 Primary outcome on composite clinical events	*44*
	7.3 Key secondary outcome on cognitive function	*44*
	7.4 Data analysis overview	*44*
	7.5 Analysis of the primary outcome	*45*
	7.6 Analysis of secondary outcomes	*45*
	7.7 Planned Subgroup Analyses	*45*
	7.8 Interim analyses and impact on sample size	*46*
	7.9 Stopping Criteria	*46*
	7.10 Health economic evaluation	*46*
	7.11 Analysis of virtual control data	*47*
**8**	**ETHICAL, REGULATORY & GOVERNANCE ISSUES**	**48**
	8.1 Ethical framework	*48*
	8.2 Ethical review	*48*
	8.3 Protocol review	*48*
	8.4 Public and Patient Involvement (PPI)	*49*
	8.5 Regulatory compliance	*49*
**9**	**DATA SECURITY**	**50**
	9.1 Data security at the University of Birmingham (coordinating institution)	*50*
	9.2 Data security at University Hospitals Birmingham (lead NHS institution)	*51*
	9.3 Data security at CPRD	*51*
	9.4 Data security for cognitive function	*51*
	9.5 Data security for health economic analysis	*52*
	9.6 Data protection and patient confidentiality	*52*
**10**	**OVERSIGHT & MONITORING**	**53**
	10.1 Trial Management Group	*53*
	10.2 Trial Steering Committee	*53*
	10.3 Data Monitoring Committee	*53*
	10.4 Expert Advisory Group	*53*
	10.5 Protocol deviations	*53*
	10.6 Data access	*54*
	10.7 Archiving	*54*
	10.8 Trial monitoring	*54*
	10.9 Clinician responsibility	*54*
**11**	**FINANCIAL CONSIDERATIONS**	**55**
	11.1 Trial funding	*55*
	11.2 Payment to participants	*55*
	11.3 Insurance	*55*
**12**	**DISSEMINATION POLICY**	**56**
	12.1 Study teams	*56*
	12.2 Publications	*56*
	12.3 Study participants	*56*
	12.4 Community engagement	*56*
**13**	**DaRe2THINK sub-studies**	**57**
	13.1 Validation of clinical events	*57*
	13.2 Neuro-vascular imaging	*57*
**14**	**REFERENCES**	**60**
**15**	**SCHEDULE OF ASSESSMENT**	**63**

KEY STUDY CONTACTS

**Chief Investigators**	Dipak Kotecha (Chief Investigator), Professor of Cardiology, Institute of Cardiovascular Sciences, University of Birmingham & Institute of Translational Medicine, University Hospitals Birmingham NHS Foundation Trust; Email: d.kotecha@bham.ac.uk<br><br>David Shukla (Deputy Chief Investigator), General Practitioner & Lead for the West Midlands Primary Care CRN team, Institute of Applied Health Research, University of Birmingham; Email: david.shukla@nihr.ac.uk
**Trial Management Group ***	Susan Beatty, Clinical Operations Manager, Clinical Practice Research Datalink, MHRA; Email: Susan.Beatty@mhra.gov.uk<br><br>Samir Mehta, Statistician, Birmingham Clinical Trials Unit; Email: s.mehta.1@bham.ac.uk<br><br>Tim Williams, Head of Interventional Research, Clinical Practice Research Datalink, MHRA; Email: tim.williams@mhra.gov.uk<br><br>Sophie Breeze, Clinical Study Development Manager, Clinical Practice Research Datalink, MHRA; Email: sophie.breeze@mhra.gov.uk<br><br>Alastair Mobley, DaRe2THINK Trial Coordinator; Email: alastair.mobley@nihr.ac.uk<br><br>Xiaoxia Wang, DaRe2THINK Health Data Analyst; Email: xiaoxia.wang@uhb.nhs.uk<br><br>Rebecca Ghosh, Senior Researcher, Clinical Practice Research Datalink, MHRA, Email: Rebecca.Ghosh@mhra.gov.uk<br><br>Karen Lancaster, Primary Care Recruitment Lead, Clinical Practice Research Datalink, MHRA, Email: Karen.Lancaster@mhra.gov.uk<br><br>Stuart Fordyce, Primary Care Recruitment Manager, Clinical Practice Research Datalink, MHRA, Email: Stuart.Fordyce@mhra.gov.uk
**Data Coordination Team ***	Krish Nirantharakumar (Chair), UKRI Innovation Clinical Fellow & Honorary Consultant in Public Health Medicine, University of Birmingham; Email: k.nirantharan@bham.ac.uk [Health record coding]<br><br>Naomi Allen, Professor in Epidemiology, University of Oxford & Chief Scientist, UK Biobank; Email: naomi.allen@ndph.ox.ac.uk [Cognitive function]<br><br>Melanie Calvert, Professor of Outcomes Methodology & Director, Centre for Patient Reported Outcomes Research & Birmingham Health Partners Centre for Regulatory Science and Innovation; Email: m.calvert@bham.ac.uk [Patient-reported outcomes]<br><br>Alastair Denniston, Director of INSIGHT - the Health Data Research Hub for Eye Health & Honorary Professor, University Hospitals Birmingham NHS Foundation Trust & University of Birmingham; Email: a.denniston@bham.ac.uk [Secondary care data integration]<br><br>George Gkoutos, Professor of Bioinformatics, University of Birmingham & University Hospitals Birmingham NHS Foundation Trust; Email: g.gkoutos@bham.ac.uk [Outcome validation]<br><br>Puja Myles, Head of Observational Research, Clinical Practice Research Datalink, MHRA; Email: puja.myles@mhra.gov.uk [Primary care data integration]
**Health Economics Team ***	Sahan Jayawardana, Health Economist, London School of Economics and Political Science; Email: s.a.jayawardana@lse.ac.uk<br><br>Elias Mossialos, Professor & and Director of LSE Health, London School of Economics and Political Science; Email: e.a.mossialos@lse.ac.uk

**Expert Advisory Group ***	Simon Ball (Chair), Executive Medical Director, University Hospitals Birmingham NHS Foundation Trust & Health Data Research UK Midlands; Email: simon.ball@uhb.nhs.uk<br>Colin Baigent, Professor of Epidemiology & Deputy Director, Clinical Trial Service Unit, University of Oxford; Email: colin.baigent@ndph.ox.ac.uk<br>Peter Brocklehurst, Director, Birmingham Clinical Trials Unit; Email: p.brocklehurst@bham.ac.uk<br>Will Lester, Consultant Haematologist, University Hospitals Birmingham NHS Foundation Trust; Email: will.lester@uhb.nhs.uk<br>Richard McManus, Professor of Primary Care & General Practitioner, University of Oxford; Email: richard.mcmanus@phc.ox.ac.uk<br>Stefano Seri, Professor & Consultant in Clinical Neurophysiology, Birmingham Women's and Children's NHS Foundation Trust & Aston University; Email: s.seri@aston.ac.uk<br>Janet Valentine, Director of the Clinical Practice Research Datalink, MHRA; Email: janet.valentine@mhra.gov.uk
**Trial Steering Committee ***	A John Camm (Chair), Professor of Clinical Cardiology, St. George's University of London; Email: jcamm@sgul.ac.uk<br>Martin Cowie, Professor of Cardiology, National Heart & Lung Institute, Imperial College London & Royal Brompton & Harefield NHS Trust; Email: m.cowie@imperial.ac.uk<br>Sandra Haynes MBE, Patient and Public Involvement team lead<br>Dame Julie Moore, Professor of Healthcare Systems, University of Warwick; Email: julie@damejuliemoore.uk<br>Amy Rogers, General Practitioner & University of Dundee; Email: a.rogers@dundee.ac.uk<br>Mary Stanbury, Patient & Public Involvement
**Data Monitoring Committee**	Marcus Flather, Professor of Medicine and Clinical Trials, University of East Anglia; Email: m.flather@uea.ac.uk<br>Suzy Walker, General Practitioner, Fortrose Medical Practice, Highland; Email: suzy.walker@nhs.scot<br>Duolao Wang, Professor of Biostatistics, Liverpool School of Tropical Medicine; Email: duolao.wang@lstmed.ac.uk

* The Chief Investigator and Deputy Chief Investigator will co-chair the Trial Management Group and contribute to other committees as required.

STUDY SUMMARY

Title	Preventing stroke, premature death and cognitive decline in a broader community of patients with atrial fibrillation using healthcare data for pragmatic research: A randomised controlled trial
Acronym	DaRe2THINK
Trial Design	Individual-patient, randomised, parallel-group, open-label, event-driven superiority trial
Funder	National Institute for Health Research (NIHR) Health Technology Assessment Programme
Sponsor	University of Birmingham, UK
Trial Methods	Data-enabled randomised trial embedded within NHS Primary Care using the Clinical Practice Research Datalink (CPRD) Interventional Research Services Platform, with automated screening, targeted patient enrolment and ‘no-visit’ follow-up through innovative technology-supported methods; Primary outcome assessment at five years from study start (or when primary outcome events reached)
Trial Medications	1:1 allocation to direct oral anticoagulants (DOAC) or no therapy; choice of DOAC (apixaban, dabigatran, edoxaban or rivaroxaban) according to local practice
Primary Outcome	Composite of cardiovascular mortality, ischaemic cerebrovascular events (stroke and transient ischaemic attacks), all thromboembolic events (including venous and arterial thromboembolism), myocardial infarction and vascular dementia
Key secondary outcome	Change in cognitive function status assessed through validated periodic objective testing with the UK Biobank cognitive function panel (primary parameter: fluid intelligence score)
Health economic outcome	Incremental cost per quality-adjusted life-year gained from the use of DOAC compared to no therapy from healthcare and societal perspectives
Additional secondary outcomes	• Individual components of the primary outcome<br>• Cumulative event rates for components of the primary outcome<br>• Composite of non-fatal stroke, non-fatal myocardial infarction and cardiovascular death<br>• Any major bleeding or clinically-relevant non-major bleeding that requires hospitalisation<br>• Minor bleeding that requires attention from primary care<br>• Haemorrhagic stroke and other types of intracranial bleeding<br>• All-cause general practice visits<br>• All-cause hospital admissions and duration of stay<br>• Heart failure hospitalisation and duration of stay<br>• All-cause mortality<br>• Patient-reported quality of life using the EQ-5D-5L index score<br>• Patient-reported quality of life using the EQ-5D-5L visual analogue score
Process outcomes	• Number/proportion of potential participants located by CPRD and notified to the lead NIHR Clinical Research Network (CRN)<br>• Number/proportion of primary care practices that have completed sign-up processes<br>• Number/proportion of patients eligible on automated screening that are successfully recruited

	• Rate of patient recruitment<br>• Patient-reported compliance to DOAC therapy in the DOAC arm<br>• Repeat prescriptions obtained for DOAC therapy<br>• Missing data rates for patient-reported outcomes
Outcome derivation	Extraction of coded electronic health records from primary care (CPRD) and secondary care (Hospital Episode Statistics); patient-reported cognitive function (yearly) and quality of life (6-monthly) obtained through digital methods
Trial Duration	Anticipated 2 years recruitment and further 3 years follow-up to achieve number of events; consent for further outcome assessment at 10-years and lifetime follow-up through electronic records
Planned Trial Sites	Up to 600 General Practices across England that are part of the CPRD network, facilitated by the NIHR CRN
Total Number of Participants	3,000
Inclusion criteria	1. Diagnosis of AF (previous, current or chronic)<br>2. Age at enrolment ≥60 years to ≤73 years
Exclusion criteria	1. Existing use of an anticoagulant.<br>2. Another clinical indication for anticoagulation.<br>3. Hypersensitivity or known intolerance to direct oral anticoagulants.<br>4. Prior documented stroke, transient ischaemic attack or thromboembolism.<br>5. Two or more CHA_2DS_2-VASc one-point risk factors indicating a risk of stroke or thromboembolism: Heart failure, Hypertension; Age 65 years or older; Diabetes mellitus; Previous myocardial infarction, peripheral artery disease or aortic plaque; and/or Female gender (if in the presence of other risk factors).<br>6. Active clinically-significant bleeding.<br>7. Prior major bleeding, defined as any intracranial bleed, or bleeding that resulted in a drop in haemoglobin ≥2g/dL, required hospitalisation or transfusion.<br>8. Condition that poses a significant risk for bleeding (within 12 months) including gastrointestinal ulceration, brain/spinal/ophthalmic injury or surgery, arteriovenous malformations or vascular aneurysms, major intraspinal or intracerebral vascular abnormalities, hepatic disease associated with coagulopathy, known or suspected oesophageal varices, and cancer with high bleeding risk.<br>9. Estimated glomerular filtration rate <30 mL/min/1.73m^2 measured within the last 12 months.<br>10. Patients receiving systemic treatment with azole-antimycotics within the last 3 months (ketoconazole, itraconazole, voriconazole and posaconazole).<br>11. Current diagnosis of dementia.<br>12. Life expectancy <2 years.<br>13. Unable or unwilling to provide informed consent for access and linkage of past and future electronic healthcare records.<br>14. Currently participating in another clinical trial.

ABBREVIATIONS

ABPI	Association of the British Pharmaceutical Industry
AE	Adverse event
AF	Atrial fibrillation
AR	Adverse reaction
BNF	British National Formulary
CCG	Clinical Commissioning Group
CHA_2DS_2-VASc	Thromboembolic risk score (including heart failure, hypertension, age, diabetes, vascular disease and gender)
CPRD	Clinical Practice Research Datalink
CRN	Clinical Research Network
CTA	Clinical Trial Authorisation
CTIMP	Clinical Trial of Investigational Medicinal Product
DIBD	Developmental International Birth Date
DMC	Data Monitoring Committee
DOACs	Direct oral anticoagulants
DSUR	Development Safety Update Report
EudraCT No.	European Union Drug Regulating Authorities Clinical Trials Number
GCP	Good Clinical Practice
GP	General Practitioner
HDR-UK	Health Data Research - United Kingdom
HES	Hospital episode statistics
ICER	Incremental cost-effectiveness ratio
IRSP	Interventional Research Services Platform
ISRCTN	International Standard Randomised Controlled Trial Number
MHRA	Medicines and Healthcare products Regulatory Agency
NHS	National Health Service
NICE	National Institute for Health and Care Excellence
ONS	Office for National Statistics
NIHR	National Institute for Health Research
PIS	Participant Information Sheet
PPI	Public and Patient Involvement
PRO	Patient-reported outcome
QALY	Quality-adjusted life-year
RCT	Randomised controlled trial
REC	Research Ethics Committee
REDCap	Research Electronic Data Capture case report system
RSI	Reference Safety Information
SAE	Serious adverse event
SAR	Serious adverse reaction
SAP	Statistical analysis plan
SmPC	Summary of Product Characteristics
SUSAR	Suspected unexpected serious adverse reaction
SWAT	Study-Within-A-Trial
TMG	Trial Management Group
TSC	Trial Steering Committee
UHB	University Hospitals Birmingham NHS Foundation Trust

PLAIN ENGLISH SUMMARY

The National Health Service (NHS) is unlike any other in the world, caring for people throughout their lives both in the community and in hospitals. At the heart of DaRe2THINK is that health data collected within these services can be used for the benefit of patients. Clinical trials are an important way to understand how new treatments can be used in the NHS, but many trials struggle to find the right patients, or be relevant to their needs. The DaRe2 approach (healthcare Data for pragmatic clinical Research in the NHS – primary 2 secondary) will test a new way of running trials based at General Practitioner (GP) surgeries using routine NHS information. We will include patients that don't normally take part in clinical trials and follow them up without the need to revisit their GP or attend hospital. This approach could improve the health and well-being of those treated by the NHS, whilst reducing the time needed from staff and patients to engage in important research.

As an example of this new system, DaRe2THINK will target an issue of huge importance to patients, our NHS and the social care system. Atrial fibrillation (AF) is a common heart rhythm condition that leads to a high chance of stroke, frequent hospital admissions and poor quality of life. Patients also have a much higher risk of cognitive decline (trouble remembering, concentrating or making every-day decisions) and dementia. This may be due to silent 'micro-strokes' that gradually damage the brain over time. Blood thinning tablets (anticoagulants) greatly reduce the number of patients with AF that will suffer a stroke, but are usually only given to older patients or those with other health issues. This may be too late to avoid dementia. It also leaves those younger than 65 years, and some patients aged 65-75 without treatment that could prevent these devastating complications.

A new class of blood thinning tablets are now widely used in the NHS which are more convenient for patients to take, and have a lower risk of bleeding than older treatments. These drugs could provide an effective way to prevent strokes, brain damage and dementia in later life for a broader group of patients, but this needs to be tested in a clinical trial. With the support of a Patient and Public Involvement Team and a national network of research nurses and GPs, the trial will include 3,000 patients from up to 600 GP surgeries across England. Each patient will either continue their current treatment or start an additional blood thinning tablet on a random basis. Patients will be followed up automatically within the NHS to look at the difference in those who suffer from strokes, blood clots, heart attacks, other problems with the blood vessels and dementia. Patients will self-report their memory, reaction times and quality of life using simple questionnaires through their mobile phone or the internet, again without needing to revisit their doctor.

DaRe2THINK will answer important questions for a growing number of patients with AF. The combination of information from the community as well as hospitals across the NHS will allow us to see whether these blood thinning tablets should be prescribed more widely. DaRe2THINK will allow us to develop and improve this new clinical trial system so that future research in the NHS will continue to benefit those patients most in need.

SCIENTIFIC ABSTRACT

Research question: Using an efficient, nationwide, primary care approach for an NHS-embedded randomised controlled trial (RCT), does direct oral anticoagulant (DOAC) therapy reduce premature death, stroke and other thromboembolic consequences of atrial fibrillation (AF) in younger patients, including prevention of cognitive decline and vascular dementia?

Background: Current RCT methodology often leads to recruitment of highly selected participants with less diversity than the clinical population, and challenges with enrolment and retention of patients. New RCT approaches are needed that can realise the value of the world-leading data quality and infrastructure of the NHS. AF is the most common heart rhythm abnormality, expected to double in prevalence in the next few decades, and leads to a considerable burden for patients and society at-large. In particular, the impact of stroke, cognitive decline and vascular dementia are all major public health concerns.

Aims and objectives: DaRe2THINK will test the hypothesis that DOACs are effective and cost-effective in patients with AF at low or intermediate risk of stroke by using an ambitious and innovative data-enabled approach through the Clinical Practice Research Datalink (CPRD) in Primary Care General Practices across England.

Methods: Individual-patient, open-label, event-driven RCT with 1:1 allocation to DOAC or no additional therapy (usual care). Automated screening of over 12 million patients, with targeted recruitment to practices with eligible patients, regular updates to General Practitioners, simple processes for centre inclusion and patient randomisation, and no additional visits after baseline for any patient. The primary outcome is a comprehensive composite of any thromboembolic event, including cardiovascular mortality, ischaemic stroke, pulmonary or venous thromboembolism, myocardial infarction and vascular dementia, ascertained entirely using electronic healthcare records within both primary and secondary NHS care. The key secondary outcome is the change in cognitive function, using technology solutions to provide ‘no-visit’ patient-reported follow-up, saving time for clinical staff and patients. We will carefully assess and validate safety outcomes relating to major and minor bleeding, and a systematic health economic analysis will determine NHS and societal cost-effectiveness. A sub-study using cerebral and retinovascular imaging will be performed to understand the mechanistic effects of DOAC therapy.

Timelines for delivery: Total duration 60 months, including 3-stage internal pilot (8 months), patient recruitment (24 months), and additional 36 months follow-up for primary and secondary outcomes. A further outcome assessment at 10 years will specifically target development of vascular dementia.

Anticipated impact and dissemination: DaRe2THINK will demonstrate the operational capabilities of using the NHS record for interventional research. We will recruit a diverse, population-relevant cohort using automated nationwide screening, prioritisation of centres with recruitable patients, and remote technology-enhanced follow-up. These innovations will allow us to answer a key question for 21st century healthcare relating to an increasingly common condition with considerable burden on patient quality of life, the health of the nation and our economy. Current and future impacts from AF and vascular dementia will be unsustainable unless we can expand prevention. DaRe2THINK will develop close collaboration between the NIHR Clinical Research Network, CPRD, patient groups, academic institutions and the NHS to address this and future evidence-gaps in clinical practice.

TRIAL FLOW CHART

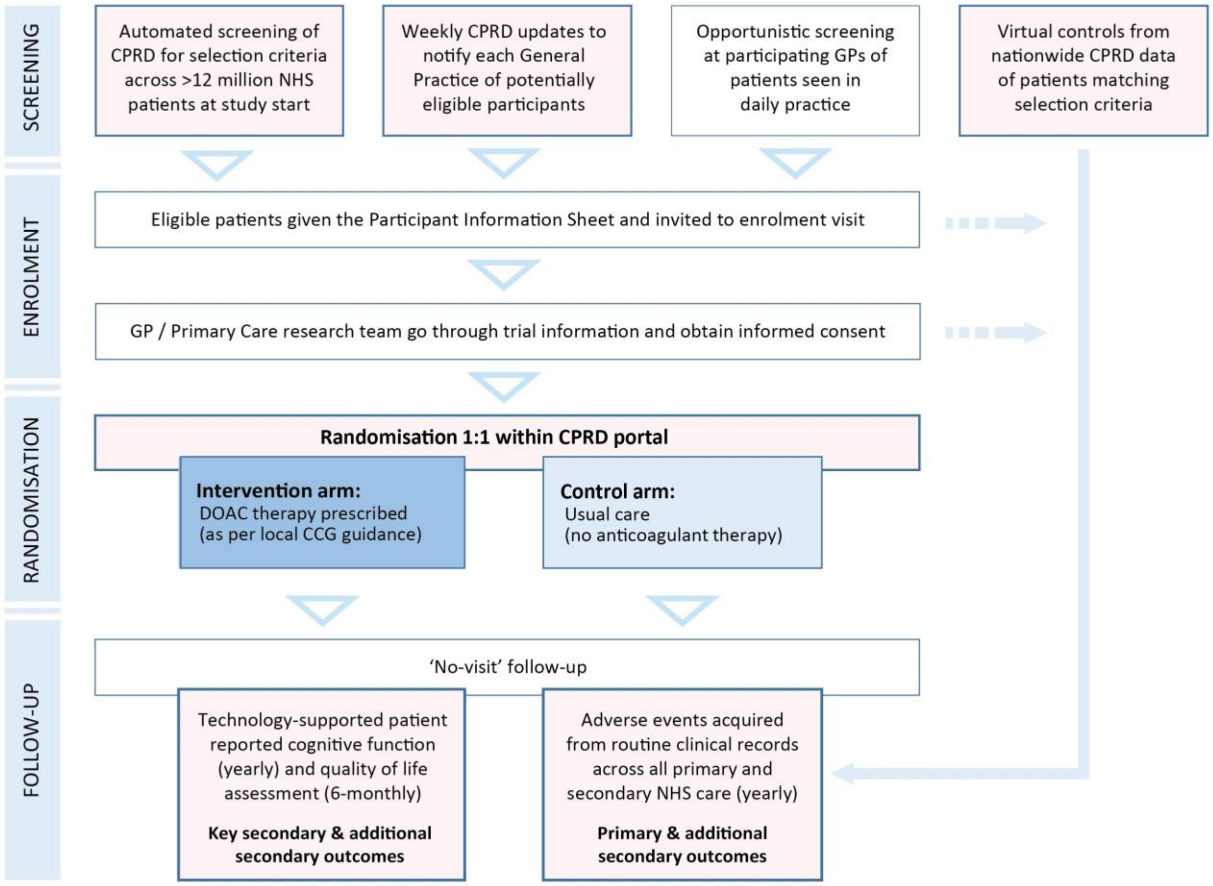

PARTICIPANT SELECTION CRITERIA

Automated selection criteria from electronic health record / Additional criteria and confirmation by GP

Inclusion criteria:

- Diagnosis of AF (previous, current or chronic)
- Age at enrolment ≥60 years to ≤73 years

Exclusion criteria:

- Existing use of an anticoagulant
- Another clinical indication for anticoagulation
- Hypersensitivity or known intolerance to direct oral anticoagulants
- Prior documented stroke, transient ischaemic attack or thromboembolism
- Two or more CHA_2DS_2-VASc one-point risk factors indicating a risk of stroke or thromboembolism: Heart failure*; Hypertension*; Age 65 years or older; Diabetes mellitus*; Previous myocardial infarction, peripheral artery disease or aortic plaque; and/or Female gender †
- Active clinically-significant bleeding
- Prior major bleeding, defined as any intracranial bleed, or bleeding that resulted in a drop in haemoglobin ≥2g/dL, required hospitalisation or transfusion
- Condition that poses a significant risk for bleeding (within 12 months) including gastrointestinal ulceration, brain/spinal/ophthalmic injury or surgery, arteriovenous malformations or vascular aneurysms, major intraspinal or intracerebral vascular abnormalities, hepatic disease associated with coagulopathy, known or suspected oesophageal varices and cancers with high bleeding risk
- Estimated glomerular filtration rate <30 mL/min/1.73m² measured within the last 12 months
- Patients receiving systemic treatment with azole-antimycotics within the last 3 months (ketoconazole, itraconazole, voriconazole and posaconazole)
- Current diagnosis of dementia
- Life expectancy <2 years
- Unable or unwilling to provide informed consent for access and linkage of past and future electronic healthcare records
- Currently participating in another Clinical Trial

Indicates data-driven automated process

CCG = Clinical Commissioning Groups; CPRD = Clinical Practice Research Datalink; DOAC = direct oral anticoagulant; GP = General Practitioner; NHS = National Health Service.

** For the automated pre-screening via CPRD, these criteria will be confirmed by the concurrent use of relevant medical therapy: Heart failure (confirmed by use of loop diuretic therapy within the last 3 months); Hypertension (confirmed by use of anti-hypertensive therapy within the last 3 months); Diabetes mellitus (confirmed by use of oral antidiabetic therapy or insulin within the last 3 months). † In the absence of other CHA_2DS_2-VASc risk factors, female gender is not independently associated with a higher risk of stroke or thromboembolism.*

1 BACKGROUND, RATIONALE & AIMS

1.1 The burden of atrial fibrillation

Atrial fibrillation (AF) is one of the most pressing concerns in the National Health Service (NHS) due to high cost, poor patient quality of life, and excess morbidity and mortality. Using primary care data from the Clinical Practice Research Datalink (CPRD), the predicted prevalence of AF in the UK will increase from 700,000 patients in 2010, to between 1.3 and 1.8 million patients by 2060.[1]

This burden will largely fall on general practice, but also have major implications on secondary care; one in four patients admitted with a stroke have AF [2], half of AF patients develop heart failure that responds poorly to treatment [3,4], and the rate of death and hospital admissions (regardless of cause) are doubled at all ages. Strokes secondary to AF lead to greater neurological damage, and the common occurrence of undetected and clinically-silent 'micro-strokes' in AF patients has intensified attention on early diagnosis.[5] Even at an average age of 64 years, 30% of patients with AF have evidence of cognitive impairment [6], with dementia risk higher in those with AF regardless of previously diagnosed strokes.[7]

Oral anticoagulation substantially reduces morbidity in AF patients [8], and the direct oral anticoagulants (DOACs) now used routinely in the NHS have distinct advantages compared to conventional warfarin therapy.[9] However, choosing which patients with AF should receive anticoagulants is difficult as clinical risk scores only have a modest ability to predict stroke. Risk scores such as CHA_2DS_2-VASc used in the NICE guidelines [10] have revolutionised our approach to stroke prevention, but typically prioritise treatment of older patients or those with multiple risk factors. In contrast, patients aged less than 65 years, and some of those aged 65-75, do not routinely receive anticoagulation due to a lack of trial data. Although their annual risk of events is relatively low, when strokes and cardiovascular events occur in this younger population they can have a profound effect on patients and their families, with long-term social and NHS impact. Further, although warfarin does not reduce the rate of cognitive decline compared to aspirin in older patients [11], observational data is suggestive that in younger AF patients at low or intermediate risk of stroke, anticoagulants could potentially reduce the risk of dementia.[12] Preventing cognitive decline and dementia is a national priority due to the high burden and cost of these conditions (£26 billion per year in the UK in 2014), and the prediction that dementia will affect over 2 million British people by 2050.[13] Interrupting the process of strokes and micro-strokes due to AF could have important advantages for the patient, the NHS and society as a whole.

1.2 The need for new trials

The increase in UK prevalence of AF in recent years [14] is predicted to rise even further.[1] The financial cost of AF is high and increasing [15]; this will be unsustainable for the NHS unless we develop better approaches to tackle the high rate of hospitalisation (a key driver of NHS cost), and the adverse impact on cognitive function (societal cost). Patients with AF at low risk of stroke treated with anticoagulants in the nationwide Swedish registry had a significantly lower risk of dementia (38% reduction, 95% CI 19-52%).[12] Similar findings have been seen in other cohort studies [16], with DOACs associated with half the rate of new-onset dementia compared to warfarin.[17] However, we must take care not to over-extrapolate observational data, as treatment biases can play a major role.[18] A robust, randomised controlled trial (RCT) is the only way to demonstrate the effect of

DOACs and thereby provide guidance to the National Institute for Health and Care Excellence and clinicians.

A conventionally run RCT would have major challenges in screening sufficient numbers of patients for such a trial. Current RCT methodology often leads to costly recruitment of highly selected participants with less diversity than the clinical population. Most RCTs are based in secondary care and suffer from challenges in enrolment and retention of patients. The current coronavirus pandemic has further curtailed clinical research. Additionally, in patients at lower risk of stroke, the benefit of oral anticoagulation must be balanced against the risk of bleeding.[5] In particular, minor bleeding is common [19] and could have an impact on quality of life that mitigates other advantages. Accurate identification of these events is only possible through integration of both community and hospital-level data, as many patients will not come to the attention of hospital clinicians. Hence a new approach is needed that will allow for efficiencies in screening, recruitment and outcome assessment, whilst also reducing cost and achieving generalizable results.

1.3 A joined-up approach across the NHS

With the majority of patient contacts in the NHS occurring within Primary Care, General Practice provides the ideal setting for undertaking larger-scale research, recruiting from diverse populations not normally accessible for hospital-based research studies. 300 million consultations per year occur within General Practice, compared to 24 million Emergency Department visits.[20] The NIHR Clinical Research Network (CRN) works with 38% of 'research active' General Practices across England, and with an increasing shift from acute to community ('place-based') care, a major opportunity exists for primary care research to add value to the NHS and benefit patients.[21]

CPRD is a government agency and part of the Medicines and Healthcare products Regulatory Agency (MHRA). CPRD staff have been working hard over the last decade to create a research environment in Primary Care that is secure, robust and unique across the world. Electronic health records from the major software providers in the UK are available within the CPRD system, and data are updated on a daily basis. Over 1,800 General Practices are currently part of the CPRD network which continues to grow, resulting in a catchment population of over 15 million patients across the UK, and over 12 million in England. CPRD is representative of the diverse UK population and therefore provides a unique opportunity for real-world RCTs that can improve medical care for actual NHS patients. CPRD has a similar spread across deciles of age and gender compared to data from the Office for National Statistics [22], and a comparable ethnicity profile compared to the UK Census (e.g. 12.7% total non-white in the Census, versus 13.2% for age-standardised CPRD).[23]

By linking CPRD data with that from NHS Digital, which includes Hospital Episode Statistics (HES) from all secondary care utilisation within the NHS, we can obtain a near totality of clinical event data for patients who provide consent. This joined-up approach is key to addressing important gaps in evidence for common and high-morbidity conditions such as AF.

1.4 The DaRe2 trial pipeline

A broad spectrum of current and foreseeable challenges to the NHS could be tackled by better linkage of routine clinical data from secondary to primary care (and vice-versa), providing a robust basis for new evidence generation. The DaRe2 approach (healthcare Data for pragmatic clinical Research in the NHS – primary 2 secondary) was designed to operationalise efficient, nationwide, primary care

approaches for NHS-embedded RCTs, providing automated screening, targeted patient enrolment and ‘no-visit’ follow-up through innovations in big data and technology solutions:

1. A National platform utilising CPRD with the capacity to run clinician-designed RCTs in primary care, demonstrating the capability of research for patient benefit within the NHS.
2. Automated screening of inclusion and exclusion criteria across more than 12 million primary care patients within 1 in 5 of GP surgeries in the NHS, providing rapid and cost-efficient screening of a diverse and representative proportion of the UK population.
3. Targeted enrolment at GP surgeries identified as having potentially eligible patients, reducing the time taken for recruitment and avoiding screening failures.
4. Updates to participating GPs on a weekly basis highlighting potentially eligible patients, simplifying the process of patient identification and recruitment.
5. Established and straightforward enrolment of patients at the GP surgery with built-in randomisation, allowing for immediate entry to an RCT, and then drug prescription via clinical systems.
6. No-visit follow-up and minimal loss to follow-up, utilising all the capabilities of NHS records for capture of endpoints: (a) without the patient needing to attend a research facility or to schedule periodic visits; (b) with no need for NHS staff to complete arduous case report forms; and (c) no requirement for costly and complex trials unit management or a contract research organisation. The coronavirus pandemic has clearly demonstrated the need for new approaches that can avoid the need for patient visits, whilst still providing robust data suitable for regulatory and guideline recommendations.
7. Innovations in technology for e-consent and patient-reported outcomes, with secure data acquisition through web-based approaches via the patient’s own smartphone, tablet or computer.
8. Sample size and power calculations based on real-world outcomes in UK patients matching inclusion and exclusion criteria, ensuring that NHS resources are not wasted on ineffective trials.
9. ‘Virtual’ controls, with full data on outcomes in patients meeting enrolment criteria nationally but not consented to enter the trial, a key innovation to explore variation in clinical events and for external validation of results.
10. An approach that showcases the potential for world-leading NHS-based research and the underpinning methodology, whilst mitigating risk through the experience of CPRD and the NIHR Primary Care CRN.

1.5 Application to patients with AF and beyond

DaRe2THINK will be the first exemplar of this system, and is appropriately focused on the intersection of key national priorities for healthcare; atrial fibrillation (a heart rhythm condition that will double in prevalence in the next few decades) and the impact this condition has on stroke, thromboembolic events, cognitive impairment and vascular dementia. These are all major burdens on our patients, as well as health and social care services. DaRe2THINK will test the hypothesis that direct oral anticoagulants (DOACs), now commonly used in the NHS for older patients with AF, are effective and cost-effective at reducing major adverse clinical events in younger patients at low or intermediate risk of stroke, and can reduce the high rate of cognitive decline. The health technology innovations noted above will allow us to answer this important clinical question, as well as demonstrate the capacity and potential of this system for future, large-scale NHS-embedded clinical trials for patient benefit.

2 DESIGN CONSIDERATIONS

2.1 Design summary

DaRe2THINK has been designed with an active Patient and Public Involvement (PPI) team and is an individual-patient, randomised, parallel-group, open-label, event-driven superiority trial with 1:1 allocation to either DOAC or no added therapy. A staged internal pilot programme is incorporated into the design of this Clinical Trial of an Investigational Medicinal Product (CTIMP). Sub-studies are listed in section 13.

DaRe2THINK will also test a health technology platform for research for patient benefit based in Primary Care in the NHS, including patient screening entirely through the CPRD Interventional Research Services Platform (IRSP), and follow-up through linked NHS outcomes and integrated technology solutions. For feasibility reasons, DaRe2THINK will focus on England only, where the 15 local CRNs can provide their expert assistance in promotion, setting-up and then enrolling General Practices and patients.

2.2 Assessment of risk for control patients

AF is associated with considerable morbidity, including 10-40% of patients hospitalised every year and 20-30% of strokes due to AF [2], heart failure in 30-50% of patients [24], and substantial impairment of quality life and psychosocial impact on patients and their families.[25] These risks are not confined to older patients. Using the UK primary care THIN dataset (2005-2018), we compared clinical outcomes in 16,574 AF patients with CHA_2DS_2-VASc <2 and not taking anticoagulants against 18,895 patients without AF. Rates of major adverse outcomes were still double in these younger patients, despite their apparently ‘low or intermediate’ risk status: adjusted incidence rate ratio 1.94 for mortality (95% CI 1.80-2.10), 3.09 for stroke (2.66-3.61), 1.81 for other thromboembolic events (1.52-2.15) and 2.03 for ischaemic heart disease (1.81-2.27). Patients with AF had higher incidence of vascular dementia (1.82; 1.07-3.06), with no increase in Alzheimer’s Disease (0.99; 0.68-1.42), as would be expected from the assumed pathophysiology that AF leads to multiple silent micro-strokes causing ‘vascular’ cognitive decline.

Patients with AF under the age of 60 have much lower incidence rates for the composite of ischaemic cerebrovascular and all thromboembolic events, even for those with other risk factors, providing a useful distinction between the balance of benefit and potential risk from anticoagulation. For example, the incidence rate for the composite outcome was 16.6 per 1000 patient years for AF patients aged 60-73 (regardless of comorbidity), compared to 8.0 for patients aged 50-60 with heart failure and a prescription for diuretics, 14.5 for those aged 50-60 with diabetes and at least one antidiabetic therapy, and 14.3 for those aged 50-60 with hypertension treated with two or more drugs.

2.3 Assessment of risk for anticoagulated patients

Although anticoagulants dramatically reduce thromboembolic events in patients with AF [26], the risk of bleeding is a concern, particularly when the event rates for stroke and thromboembolism are low. The bleeding risk with DOACs is lower than vitamin K antagonists, both for major and minor bleeding, and in particular is halved for intracranial haemorrhage.[27] In the Apixaban for Reduction in Stroke and other Thromboembolic Events in Atrial Fibrillation (ARISTOTLE) trial, the rate of non-major bleeding was 10.1% for apixaban versus 14.2% for warfarin, and 3.1% versus 4.5% for major bleeding

respectively.[19] DOACs have demonstrated equivalent or better efficacy for prevention of stroke and other adverse events compared to warfarin in both trials and real-world data.[8,9,28] It is also important to note that risk factors for thromboembolism are also risk factors for bleeding.[29] Based on this literature, it is clear that DOACs are the preferred therapy to test the balance of thromboembolic prevention and bleeding risk with anticoagulants in the patient group with low or intermediate risk of stroke.

2.4 Hypotheses for primary, key secondary & health economic outcomes

Compared to standard of care (no anticoagulant therapy), DOACs in patients with AF and a low or intermediate expected risk of stroke will:

1. Reduce the composite of cardiovascular mortality, ischaemic stroke, thromboembolic events, myocardial infarction and vascular dementia compared to no treatment, ascertained using electronic health record data from primary and secondary NHS care.
2. Prevent cognitive decline, determined by yearly technology-supported cognitive testing completed remotely by participants.
3. Be cost-effective from a healthcare and societal perspective at a willingness to pay threshold of £20,000 - £30,000 per quality-adjusted life-year.

2.5 Hypotheses for other secondary outcomes

Compared to standard of care (no anticoagulant therapy), DOACs in patients with AF and a low or intermediate expected risk of stroke will:

1. Reduce individual components of the primary outcome and their cumulative event rates, in addition to the conventional composite of non-fatal stroke, non-fatal myocardial infarction and cardiovascular death, as well as all-cause mortality.
2. Have an acceptable safety profile in terms of major bleeding, clinically-relevant non-major bleeding and minor bleeding that requires clinical attention.
3. Reduce healthcare utilisation, including General Practice visits, hospital admissions (all-cause and heart failure-specific) and the duration of hospital stay.
4. Improve patient-reported quality of life using the EQ-5D-5L questionnaire.

2.6 Overarching challenge and opportunity

RCTs are the foundation of evidence-based practice but involve escalating cost and usually target a selected group of patients. The advantages of a trial based at the community level of healthcare include:

1. Results that are more generalizable to the true population.
2. Utilisation and repurposing of data already collected as part of standard NHS care.
3. Operational, logistical and efficiency benefits leading to the ability to test interventions or NHS pathways at a scale not previously possible.

3 STUDY METHODS

3.1 Approach to study selection criteria

DaRe2THINK will operate using a system of automated patient pre-screening using coded Primary Care health records across the >12 million patients registered in CPRD GP surgeries across England. The trial adopts a pragmatic approach to the screening criteria, aiming to display to Investigators only those patients who are likely to fulfil enrolment criteria. Investigators who are medical practitioners are still required to approve automated selection criteria, and are able to exclude potential participants on review of the medical record (see section 3.4 for further details, and section 5 for information about data processing and coding). Automated pre-screening exclusion criteria for medical conditions are based on secure definitions (presence of coding for that condition plus recent prescription of relevant medical therapy) to avoid exclusion where an unsubstantiated or transient code is present in the medical record.

3.2 Study participant inclusion criteria

1. Diagnosis of AF (previous, current or chronic).
2. Age at enrolment ≥60 years to ≤73 years.

3.3 Study participant exclusion criteria

1. Existing use of an anticoagulant.
2. Another clinical indication for anticoagulation.
3. Hypersensitivity or known intolerance to direct oral anticoagulants.
4. Prior documented stroke, transient ischaemic attack or thromboembolism.
5. Two or more CHA_2DS_2-VASc one-point risk factors indicating a risk of stroke or thromboembolism: Heart failure*; Hypertension*; Age 65 years or older; Diabetes mellitus*; Previous myocardial infarction, peripheral artery disease or aortic plaque; and/or Female gender[†].
6. Active clinically-significant bleeding.
7. Prior major bleeding, defined as any intracranial bleed, or bleeding that resulted in a drop in haemoglobin ≥2g/dL, required hospitalisation or transfusion.
8. Condition that poses a significant risk for bleeding (within 12 months) including gastrointestinal ulceration, brain/spinal/ophthalmic injury or surgery, arteriovenous malformations or vascular aneurysms, major intraspinal or intracerebral vascular abnormalities, hepatic disease associated with coagulopathy, known or suspected oesophageal varices, and cancers with high bleeding risk.
9. Estimated glomerular filtration rate <30 mL/min/1.73m^2 measured within the last 12 months.
10. Patients receiving systemic treatment with azole-antimycotics within the last 3 months (ketoconazole, itraconazole, voriconazole and posaconazole).
11. Current diagnosis of dementia.

12. Life expectancy <2 years.
13. Unable or unwilling to provide informed consent for access and linkage of past and future electronic healthcare records.
14. Currently participating in another Clinical Trial.

* For the automated pre-screening via CPRD IRSP, these criteria will be confirmed by the concurrent use of relevant medical therapy: Heart failure (confirmed by use of loop diuretic therapy within the last 3 months); Hypertension (confirmed by use of anti-hypertensive therapy within the last 3 months); Diabetes mellitus (confirmed by use of oral antidiabetic therapy or insulin within the last 3 months).

† In the absence of other CHA_2DS_2-VASc risk factors, female gender is not independently associated with a higher risk of stroke or thromboembolism.[30]

3.4 General Practice selection criteria

This trial will recruit participants from GP practices in England that contribute to CPRD; as of September 2020, 1,337 practices in England (15%) are part of CPRD. Centres that use the Egton Medical Information Systems (EMIS) web software platform will be included first, with practices using TPP SystmOne and InPS Vision software used to supplement recruitment if required. Practices that are participating in any anticoagulant intervention studies where there is potential to confound or modify the effects in DaRe2THINK will be excluded.

3.5 Participant identification and Screening

The selection criteria will be applied within the CPRD IRSP at study start, based on the predefined code list. A pseudonymised patient list is generated specific to each Primary Care practice. Following the site approval process, a delegated health professional at each practice will access the list, and re-identify the patients. The patient list is then reviewed by the health professional who responds to a specified set of screening questions. Recruitment will be focused on practices with multiple potential participants for cost efficiency, targeting up to 600 GP surgeries across England. A further update will be performed for patients meeting the trial selection criteria at each participating practice on a weekly basis. The IRSP maintains confidentiality of all patient data according to CPRD internal governance and in line with the Data Protection Act 2018 (see section 9: Data Security).

GPs, practice nurses and other research staff in Primary Care (e.g. Research Nurses and CRN Research Facilitators) will review records of potential participants on the IRSP. For patients that meet the selection criteria, the Primary Care team will send out an invitation letter and/or text message informing them about the study, and providing access to the Participant Information Sheet. This includes a link to the website with an information video for potential participants developed by the PPI team and the items on the informed consent form (ICF). This will allow potential participants the opportunity to consider whether they would like to participate in the study.

Study invitation letters will be generated by Investigators using a protocol embedded in the EMIS software that is activated at the practice. When an invitation letter is generated, a NIHR code for “Invitation to participate in research study” will be inserted into the patient’s medical record. The letter includes a weblink and Quick Response (QR) code for potential participants to complete the ‘consent to contact’ electronic form (see section 3.6). A reminder alert to follow-up with the patient will appear

on the patient record seven days after this letter is generated. Text messages will be sent via the practice's own text messaging system, normally used to inform patients about their NHS appointment. The text message will inform the patient about their eligibility for the study, and provide the weblink to the 'consent to contact' electronic form. Telephone calls will also be made to potential participants, in particular to ensure that Black, Asian, and minority ethnic persons are not unduly disadvantaged from participating in the trial. The process of weekly updates of potential participants will continue throughout the recruitment period to enrol incident AF patients and those that newly meet the inclusion criteria. As part of the ongoing GP engagement plan, participating practices will be contacted regularly to notify them of patients suitable for screening at their practice.

If a patient attends with newly-diagnosed AF, an investigator may choose to update the patient's medical record as normal, and send the study invitation letter to the patient while they are waiting for the weekly patient list update on IRSP. Screening must take place on IRSP before the patient can be given access to the ICF on REDCap. Investigators may contact CPRD if they believe a patient is eligible but does not appear on their patient list in IRSP. CPRD will investigate any instances where this is reported, and can alert the Investigator if the patient does not meet the stipulated selection criteria.

3.6 Consent to contact

Eligible participants who have been successfully screened and invited by their Primary Care team will be directed to a Research Electronic Data Capture case report system (REDCap) electronic form to consent to communication by the central study team. After reviewing the PPI information video, potential participants will be asked to complete the 'consent to contact' form by entering their name, Primary Care practice name, telephone number, email address and best timeslot for contact. Central study staff will then telephone the potential participant, go through any questions arising from the Participant Information Sheet, and activate the REDCap e-consent process as documented in section 3.7.

3.7 Consent procedures for trial enrolment

Informed consent must be obtained prior to the participant undergoing procedures that are specifically for the purposes of the trial and are out-with standard routine care at the participating site. The Site Principal Investigator (PI; usually the lead GP for the study at that centre) retains overall responsibility for the conduct of research at their site; this includes the taking of informed consent of participants at their site. They must ensure that any person delegated responsibility to participate in the informed consent process (such as Practice Nurses or CRN Research Nurses) are duly authorised, trained and competent to participate according to the ethically approved protocol, principles of Good Clinical Practice (GCP) and the Declaration of Helsinki. The consent process includes explicit consent for the transfer of identifiable information on the consent form itself.

The consent process should include:

- A two-way discussion between the potential participant and an individual knowledgeable about the research, the nature and objectives of the trial and possible risks associated with their participation.

- The discussion of written material, namely the Participant Information Sheet and consent documentation approved by the Research Ethics Committee (REC), supplemented by verbal explanations from practice nurses, research nurses or GPs.
- The opportunity for potential participants to ask questions.
- An assessment of capacity; for consent to be ethical and valid in law, participants must be capable of giving consent for themselves. A capable person will understand: (1) the purpose and nature of the research; (2) what the research involves; (3) its benefits and risks; (4) alternatives to taking part; and be able to: (5) retain the information long enough to make an effective decision; (6) make a free choice; (7) make this particular decision at the time it needs to be made (though their capacity may fluctuate, and they may be capable of making some decisions but not others depending on their complexity); and (8) make the decision free of coercion.

In the case of any patients that lack capacity for consent, they will not be accepted into the study, even for those who have a legally-designated representative. Where a participant is able to consent but later becomes incapacitated, the original consent given endures the loss of capacity. The right of a participant to refuse participation without giving reasons must be respected.

Documentation of informed consent will take the form of:

(1) An online portal using REDCap which can be accessed by the potential participant through any internet connected device to complete remote e-consent. The latter is designed to avoid unnecessary physical contact, and where consultations are performed through telephone or video methods, which are now routine in Primary Care due to the coronavirus pandemic. The latest version of the REC-approved ICF should be used. Electronic consent processes will follow the Sponsor's SOPs and guidelines on remote consent (BCTU-GDL11), which are compliant with the HRA and MHRA Joint Statement on Seeking Consent by Electronic Methods (version 1.2; published 2018). An e-signature is captured by the participant using their finger, stylus or mouse (depending on their setup and type of device they are using); see section 9.2 for details on database security for e-consent. The Investigator or delegate(s) will then sign and date their portion of the e-ICF, and provide the NHS number for future linkage of healthcare records for the validation programme (see section 5.3). Regardless of whether the participant uses the consent to contact approach, or is directly enrolled by their Primary Care team, countersignature of the consent process must be performed by the Investigator or delegate and cannot be completed by any central study staff.

(2) For potential participants that attend in person or contact their Primary Care team, the Investigator or delegate is required to log into the REDCap system with their practice ID and password to initiate the consent procedure. The participant will be asked to complete the ICF on their internet connected device or a computer at the site. The Investigator or delegate(s) will then sign and date the ICF and complete recruitment processes as above. A written ICF will also be available at sites that can be completed physically if required; however, this still requires the Investigator to initiate and complete the consent process on REDCap using their practice ID and password. Written ICFs are required to be uploaded to the REDCap system on completion.

Regardless of the method of completion, consent will be documented in the electronic patient record, and the Trial Master File, with a copy provided to the participant (physical copy or email depending on the route of consent). Any original written ICFs should remain in the Investigator Site File. In the case of e-consent, copies of the digitally-signed form will be automatically sent as a PDF file to the patient

and available for review by authorised REDCap users, including the Primary Care research team, Trial Coordinator, CI and sponsor. After consent processes have been completed on REDCap, the Investigator is required to confirm consent in the CPRD IRSP in order to complete participant enrolment. CPRD IRSP generates a study specific identifier translating into a practice and patient identifier to avoid any collection of identifiable personal data.

3.8 Randomisation

Method: A simple randomisation will allocate the participants 1:1 to either DOAC therapy (intervention group) or to continue without oral anticoagulation (control; standard of care).

Implementation: The CPRD IRSP includes a module for randomisation once informed consent and selection criteria have been confirmed. The patient is assigned a unique randomisation number which is automatically generated by the IRSP. Where the participant is allocated to the DOAC arm, the resulting prescription will be logged both on the IRSP and the electronic health record as per usual clinical practice.

Statistical software: The randomisation sequence will be generated using Stata version 15.1 (StataCorp LP, Texas).

Concealment of allocation: The Investigator is blinded to the allocation sequence, however, as an open label trial, both the Investigator and participant will be aware of the allocation post randomisation.

Out-of-hours access to randomisation codes: There is no requirement to access randomisation codes out of hours or in an emergency situation in this trial.

Procedures for handling incorrectly enrolled or randomised patients: Patients who fail to meet the eligibility criteria should not, under any circumstances, be enrolled. Patients who are enrolled, but subsequently found not to meet all the eligibility criteria must not be randomised and must be withdrawn from the trial. Where a patient does not meet all the eligibility criteria but is randomised in error, the Investigator should inform the Sponsor (or delegate) via the Trial Coordinator immediately, and a discussion should occur between the Sponsor (or delegate) and the Investigator regarding whether to continue or discontinue the patient from treatment. The Sponsor (or delegate) must ensure all decisions are appropriately documented.

Procedure where a patient withdraws from the trial: If a patient withdraws from participation in the study, then their randomisation code cannot be reused. No additional patients will be recruited to the trial to replace those patients withdrawn or lost to follow up.

3.9 Trial treatments

All participants will be provided with website links to further information on AF and anticoagulation therapy created and made freely available by the British Heart Foundation.

If allocated to the control group no further action is required by the Investigator other than that required as standard of care.

If allocated to the intervention group, the Investigator selects a particular DOAC in line with clinical requirements for that patient and regional prescription guidelines by the Clinical Commissioning Group (CCG) for each participating General Practice, taking into account any relevant contraindications from the Summary of Product Characteristics (SmPC).

Potential trial treatments (apixaban, dabigatran, edoxaban and rivaroxaban) are approved for use in the NHS. For the purposes of this trial, the Reference Safety Information (RSI) for apixaban will be used, available at www.medicines.org.uk/emc/product/2878/smpc. See section 6 for details on safety data. Prior to Investigators prescribing therapy, a list of specific contradictions and interactions will be presented on the CPRD IRSP to assist in the choice of DOAC. Clinical responsibility and eligibility for the chosen DOAC will remain with the Investigator and will not be monitored, as there may be clinical reasons outside of the trial for this choice. This also includes consideration for any national or local guidelines in existence, that might include safety baseline blood tests, and any ongoing monitoring requirements. The NICE Clinical Knowledge Summaries detail prescribing information on DOACs including initiation and monitoring (available at https://cks.nice.org.uk/topics/anticoagulation-oral/management/).

Dose reduction should only be used in specific patients as clinically required (see Table 1), with all other patients receiving the usual daily dose as demonstrated in RCTs. Drug prescription will occur via the clinical electronic medical record system and dispensed alongside the patient's usual medication by their community pharmacist. When the DOAC drug is added to the medical record, Investigators are required to add the following note in the 'pharmacy text' section: "This patient is part of the DaRe2THINK trial and is being prescribed an anticoagulant as part of an NHS drug trial – see www.birmingham.ac.uk/dare2think". This text should also be added as a 'major alert' and selected to display whenever the record is swapped to or entered. This will allow other organisations (such as out-of-hours providers) to view the record.

Table 1: Suggested dose of DOACs

DOAC	Usual dose*	Reasons for dose reduction	Reduced dose
Apixaban	5mg twice daily	Two out of three indications: weight <60kg, age >80 years, serum creatinine >133mmol/L (or estimated creatinine clearance <30mL/min)	2.5mg twice daily
Dabigatran	150mg twice daily	Patients receiving regular oral verapamil<br>(Consider dose reduction on an individual basis if estimated creatinine clearance 30-50mL/min, in patients with gastritis, esophagitis or gastroesophageal reflux, and others at increased risk of bleeding)	110mg twice daily
Edoxaban	60mg once daily	Any of: weight <60kg, estimated creatinine clearance <50mL/min, or concomitant therapy with potent P-glycoprotein inhibitors	30mg once daily
Rivaroxaban	20mg once daily	Creatinine clearance <50mL/min	15mg once daily

** Investigators should follow local CCG (or equivalent) guidance. Dose and dose adjustment for each DOAC should be in line with the respective Summary of Product Characteristics. Antiplatelet agents should be stopped in most patients when commencing a DOAC (please see text in section 3.9).*

All medications are oral, with no specific storage requirements. Drug accountability will be according to standard practice for NHS prescriptions, with no additional clinical trial label. Any product recall will be managed via the usual clinical systems. Patients will not be withdrawn from the trial if they cross

over to the other arm of the trial (e.g. due to development of other risk factors for stroke and commencement of anticoagulation, or discontinuation of DOACs), or switch to an alternative DOAC or vitamin-K antagonist. Investigators and other clinicians may need to pause or discontinue anticoagulation for a range of clinical scenarios, including certain intercurrent illness such as bleeding, development of new treatment contraindications, adverse events, or requests to withdraw therapy by the participant or other clinicians (for example, due to upcoming surgical procedures). Similarly, there may be reasons outside of the trial that require clinicians to start anticoagulation in patients randomised to the control arm. These decisions remain the responsibility of the prescribing clinician. Prescription data will be collated automatically from all participants from Primary Care data, and participants will be asked to complete anticoagulation and compliance assessments every 6-months (see section 5.6). Where use of anticoagulation differs from randomised allocation, Site Investigators will be asked to provide information to the central study team on the clinical reasons for this change.

Most patients who commence a DOAC should stop antiplatelet therapy such as aspirin, dipyridamole, clopidogrel or prasugrel, as per clinical guidelines. This includes patients who are taking antiplatelets for primary prevention reasons and those with stable coronary, cerebral or vascular disease, where monotherapy with a DOAC is recommended in patients with AF. If a patient with prior acute coronary syndrome or percutaneous coronary stenting receives a DOAC, then in most cases antiplatelet therapy should cease at 12 months after the event, and thereafter the patient should receive monotherapy with a DOAC alone. Cardiologists will have explicitly stated any exceptions to this rule in clinical documentation (for example, patients with unstable complex lesions or plans for further intervention). When prescribing the DOAC, the Investigator is required to review the medical record for any antiplatelet agents and make appropriate plans for cessation, including a clear instruction to the patient and any relevant changes to the electronic prescription system.

3.10 Blinding

The trial is open-label and therefore trial participants and their care providers will not be blinded or masked with respect to intervention allocation. Outcome assessment is based on coded health outcomes and therefore less susceptible to assessment bias on the part of the research team; nonetheless, the Trial Management Group will remain blinded to intervention allocation from an analysis standpoint. The exception to this is where knowledge of allocation in individual participants is needed to facilitate communication with patients/GPs, or in cases of valid medical or safety reasons.

3.11 Internal pilot

DaRe2THINK will operate a staged internal pilot programme, which is focused on the feasibility of recruitment (both screening and actual randomisation). No outcome measures will be analysed to avoid jeopardising the full trial. A traffic light system will be used to operationalise criteria into green, amber and red for the benefit of the Trial Steering Committee who will make recommendations to the funder on continuation of the trial.

Pilot stage 1: England-wide national automated screening

Description: Access to data and screening of potential participants that meet inclusion and exclusion criteria from over 12 million primary care patients across England using the CPRD IRSP.

Time period: 2 months (clock starts at first data query by CPRD).

Measured outcomes: Defined access to General Practice data to enable patient screening across all contributing CPRD sites; Number of potential participants located and notified to the lead NIHR CRN.

	**Green**	**Amber**	**Red**
Criterion (data access)	>75% of eligible sites providing daily data updates to CPRD	50-75% of eligible sites providing daily data updates to CPRD	<50% of eligible sites providing daily data updates to CPRD
Criterion (screening)	>2000	1000-2000	<1000
Outcome	Progression without major modification (whilst also resolving any identified barriers)	Progression whilst under review for identification and modification of trial processes	Detailed review of project viability by the Trial Steering Committee and funder

Pilot stage 2: GP surgeries set up and ready to recruit patients

Description: Assessment and engagement with priority primary care sites (i.e. those with multiple potential participants), on-boarding of practice investigators, bringing the practices to a state of recruitment readiness, and continued access to data.

Time period: 3 months.

Measured outcomes: Number of primary care practices that have completed sign-up processes with CPRD and the NIHR CRN; Continued ongoing access to practice data to facilitate patient recruitment into DaRe2THINK.

	**Green**	**Amber**	**Red**
Criterion (practices)	>60	30-60	<30
Criterion (data access)	Ongoing data collection from >75% of practices	Ongoing data collection from 50-75% of practices	Ongoing data collection from <50% of practices
Outcome	Progression without major modification (whilst also resolving any identified barriers)	Progression whilst under review for identification and modification of trial processes	Detailed review of project viability by the Trial Steering Committee and funder

Pilot stage 3: Recruitment of patients for DaRe2THINK

Description: Conversion to recruitment of eligible patients at primary care centres taking part in DaRe2THINK.

Time period: 3 months (endpoint month 08).

Measured outcomes: Proportion of patients eligible on automated screening that are successfully recruited (future feasibility assessment therefore no criterion); Rate of patient recruitment (numbers include a 50% reduction in recruitment rate during the pilot).

	**Green**	**Amber**	**Red**
Criterion (recruitment)	>60 per month by month 8	30-60 per month by month 8	<30 per month by month 8
Outcome	Progression without major modification (whilst also resolving any identified barriers)	Progression whilst under review for identification and modification of trial processes	Detailed review of project viability by the Trial Steering Committee and funder

3.12 Withdrawal

Participants may withdraw at any time during the trial without giving reasons and without prejudicing their further treatment, or if their clinical team feel that continued participation in the trial is inappropriate. This will not affect the patient's access to any future NHS care.

As per the consent process, personal data collected up to the point of withdrawal can be used. For participants that wish to withdraw, an option will be given to remove themselves from patient-reported outcomes, NHS electronic health record follow-up (no contact with the study team), or both.

3.13 Change of Primary Care provider

If the enrolled participant changes their Primary Care provider, the flow of electronic health record data to CPRD will cease. The registration end date will be monitored by CPRD for enrolled participants, and any instances where this occurs will be reported alongside the safety reporting as described in section 6.5 to the Trial Coordinator and CI. If the participant moves to another Primary Care practice that contributes to CPRD, then the new practice and patient identifier will be captured by CPRD and the flow of electronic health record data to CPRD will resume. If the practice does not already contribute to CPRD, then they will be approached to join CPRD and the study by the CPRD recruitment team. If the practice is unwilling or unable to join CPRD and the study, then the participant will be withdrawn from the trial (discussed explicitly in the Participant Information Sheet). As an additional check, enrolled participants will be asked at the 6-monthly patient-reported outcome timepoint if they have moved, or are considering a move to another Primary Care provider. In this

circumstance, an automated REDCap alert will be sent to the Trial Coordinator to telephone the participant.

3.14 Treatment duration and End of Trial

If randomised to the DOAC arm, participants will receive treatment at least until the end of the trial (see below). At this point, the Investigator and patient should make a shared decision as to whether the DOAC will continue based on the individual clinical circumstances of each patient and an appraisal of risk factors at that time for stroke and thromboembolism (as per normal clinical practice).

'End of trial' for regulatory purposes is defined as the last data capture of the last participant for phase 1 of follow-up (estimated at five years after the trial commences). As this is an event-driven trial, the precise date will depend on the accumulation of outcome events in the intervention and control arms, which will be monitored by the independent Data Monitoring Committee (DMC) and Trial Steering Committee (TSC). The Trials Office will notify the MHRA and REC that the trial has ended within 90 days of the end of trial and provide them with a summary of the clinical trial report within 12 months.

Participants will also be consented for 10-year follow-up with patient-reported and NHS care outcomes (phase 2), and then lifetime follow-up for NHS care outcomes (phase 3; pending further funding) – these elements constitute the non-interventional (observational) phase of the trial.

4 OUTCOMES

4.1 Timing & assessment of outcomes

All outcome data will be collated on a yearly basis, except for EQ-5D-5L quality of life and DOAC compliance data which is collected 6-monthly. Primary analysis will take place at 5-years follow-up from first patient randomisation, or when the specified event numbers have been reached (see section 7.1).

4.2 Primary outcome

The primary outcome for DaRe2THINK is a composite of cardiovascular mortality, ischaemic cerebrovascular events (stroke and transient ischaemic attacks), all thromboembolic events (including venous and arterial thromboembolism), myocardial infarction and vascular dementia.

4.3 Secondary outcomes

The key secondary outcome is:

- Change in cognitive function status assessed through validated periodic objective testing with the UK Biobank cognitive function panel (primary parameter is the fluid intelligence score).

Additional secondary outcomes are:

- Individual components of the primary outcome.
- Cumulative event rates for each individual component of the primary outcome.
- Conventional major adverse cardiovascular events (composite of non-fatal stroke, non-fatal myocardial infarction and cardiovascular death).
- Any major bleeding or clinically-relevant non-major bleeding that requires hospitalisation.
- Minor bleeding that requires attention from primary care (any bleeding that leads to a primary care consultation).
- Haemorrhagic stroke and other types of intracranial bleeding.
- All-cause general practice visits.
- All-cause hospital admissions and duration of stay.
- Heart failure hospitalisation and duration of stay.
- All-cause mortality.
- Patient-reported quality of life using the EQ-5D-5L index score.
- Patient-reported quality of life using the EQ-5D-5L visual analogue score.

4.4 Cost-effectiveness outcomes

The primary outcome measure for the economic evaluation will be quality-adjusted life-years (QALY) gained. Cost-effectiveness will be measured in terms of the incremental cost per QALY gained from the healthcare perspective. The secondary outcome measure for the economic evaluation will consider the societal perspective.

4.5 Process outcomes

1. Number/proportion of potential participants located by CPRD and notified to the lead NIHR CRN (reported monthly).
2. Number/proportion of primary care practices that have completed sign-up processes (reported monthly).
3. Number/proportion of patients eligible on automated search and thought eligible by the GP (reported monthly).
4. Rate of patient recruitment (reported monthly).
5. Patient-reported compliance to DOAC therapy in the DOAC arm only (reported annually; see section 5.6).
6. Repeat prescriptions obtained for DOAC therapy in the DOAC arm only, using Primary Care prescription data (reported annually).
7. Missing data rates for patient-reported outcomes (reported annually).

4.6 Rationale for outcomes

Four focus groups were undertaken (led by the PPI team) to understand what is most important to patients with AF and the relevant impact on quality of life.[25] The patients were clear that they see AF as a multisystem disorder that it difficult to separate from other comorbidities. The impact on their physical and emotional wellbeing was not solely due to conventional cardiovascular outcomes (such as myocardial infarction), but more broadly related to all thromboembolic complications. AF is known to have a considerable and broad adverse impact on patient quality of life due to the burden of a wide range of symptoms and psychosocial consequences.[31] We are also conscious that this trial is based in primary care and so outcomes should reflect the short and long-term burden on GPs from all aspects of AF and its associated sequelae, ranging from a near-fatal stroke to a deep vein thrombosis. The rationale for the primary outcome composite is the increased incidence of these events in the context of AF compared to sinus rhythm, the impact these events have on patient wellbeing, and the considerable burden placed on the NHS as a result.

Although cognitive decline is of key interest in DaRe2THINK, this is a secondary outcome as the true impact of DOACs on cognitive function is currently unknown. The patient-reported approach has distinct advantages [32] and in this context allows us to abolish trial visits, enhancing efficiency of the programme. In 2019, 83% of those aged 55-64 in the UK accessed the internet daily or almost every day, and only 10% had not used the internet in the previous 3 months; 73% of respondents preferred to access the internet via smartphone.[33] Although our approach will cater for the majority of trial participants and will be supported by the PPI team, we anticipate attrition from yearly patient-reported testing (see sample size calculation in section 7.3).

The rationale for the health economic evaluation is to assess the cost-effectiveness of DOACs in patients with AF at a low or intermediate expected risk of stroke compared to no treatment. This will include both the English NHS and societal perspectives, due to the substantial impact that AF and its consequences have on the community.[34] The cost-effectiveness analysis will be conducted in parallel to the DaRe2THINK randomised trial by an expert team at the London School of Economics.

5 DATA MANAGEMENT

5.1 Baseline and follow-up data

Electronic health record data will be collected directly through the CPRD system, and so there is no requirement for Investigators or front-line NHS staff in Primary Care to complete case report forms to record demography and patient characteristics. This includes medical history (such as previous vascular/thromboembolic disease, bleeding episodes, diabetes and other comorbidities, and COVID-19 etc.), medications, clinical measurements and tests, and blood results. The Statistical Analysis Plan will detail each variable collected and relevant descriptions/determinants.

No follow-up visits are required as data linkage will occur through CPRD of primary care data, secondary care data (via HES) and data from the Office for National Statistics (ONS). Efficacy and safety outcomes will be collated from these sources based on a pre-specified clinical code set supplemented, where required, with a safety reporting process via IRSP. The code lists will be published in advance of the first endpoint assessment at one year. No physical patient follow-up is required; a remote, digital system will collect cognitive function yearly and patient-reported quality of life at six-month intervals, following notification by text and email to consenting individuals.

5.2 Quality of CPRD data

Adequacy of data: As CPRD uses real-world data, it does not modify data values but adds quality markers to every dataset. There are three levels of data quality assessment for data validation (see Table 2). Level 1 and 2 checks are undertaken by CPRD as part of their data quality processes. CPRD employs over 900 data quality assurance checks covering integrity and format of the data. This includes practice and patient-level quality markers, with the ability to audit all data values to comply with Good Clinical Practice. Additional checks are made for recruitment in the context of interventional research. Patients meeting selection criteria based on the pre-screened electronic healthcare records are presented to primary care research staff. This allows for clinical validation of coded information, assessment of fitness to participate by GPs, and incorporation of other non-coded clinical information. Level 3 checks are study-specific and will be undertaken by the University of Birmingham.

Table 2: Quality assurance processes

Quality assurance	Timing	Purpose & process	Outcome
Level 1	The first series of checks that focus on verifying that the data meet agreed specifications and requirements. These are undertaken as part of the daily Extract, Transform and Load (ETL) process when data flow into CPRD databases.	To ensure that any received data contains only expected data files and that all data elements are structured correctly as per the agreed specification. Duplicate records are removed and sequencing is verified before integration into a master dataset.	These checks ensure that data are uncorrupted and have both structural and referential integrity as defined by the specification. Any Level 1 failure results in the data not being used and a resupply of those data being requested.
Level 2	These checks revolve around research quality validation and cover the actual content of the data.	CPRD provides a patient-level data quality marker (acceptability flag) based on	Representativeness of data is assessed by comparing the prevalence of selected Quality Outcomes Framework

	They are generated every time CPRD builds and releases a research database.	internal data consistency at the individual-patient level.	conditions against national figures [35], with quality markers produced on the entire database.
Level 3	Checks on released databases provided by CPRD to the University of Birmingham.	Study-specific checks that will be undertaken as part of good data management. For example, examining trends in clinical coding and consistency for outcomes of interest over time to inform the definitions of code lists.	Quality indicators tailored to specific areas or subsets of the data.

Timeliness and scale of data: CPRD databases used for interventional research receive daily updates of primary care data, providing access to up-to-date information across all contributing practices, thereby enabling: (1) Pre-screening searches carried out at scale and standardised according to the trial protocol; (2) Contemporaneous application of selection criteria with weekly refreshes; (3) GP screening through the IRSP interface allowing the study team to actively monitor screening activity in real-time and the flexibility to adjust the search algorithm in response to recruitment metrics; (4) In-built recording of serious adverse events according to Good Clinical Practice and regulatory/ethics requirements via IRSP; and (5) Regular, restricted, safety-focused data downloads to monitor safety directly from the electronic healthcare record, configurable to provide regular updates to the Data Monitoring Committee.

UK NHS data coding: Due to the General Medical Services Quality and Outcomes Framework contract that operates in Primary Care, accurate coding is rewarded and incentivised. This underpins how the UK leads the world in accurate healthcare coding, with high accuracy demonstrated in a systematic review for primary codes used after 2004.[36] Multiple studies have validated disease-specific accuracy of CPRD data, including for complex diseases [37] and behavioural conditions.[38] Pharmacotherapy is similarly coded in the Primary Care record; for full transparency Table 3 provides the UK British National Formulary (BNF) codes for medical therapy used for the participant criteria. A full list of codes for all outcomes will be published on the trial website and design paper.

Table 3: Drug codes used for the participant selection criteria

Pharmacotherapy	BNF code(s)
Therapy for diabetes (oral; insulin)	06010100-3; 06010201-4
Therapy for hypertension (various drug classes)	02020-100,300,400; 02040000; 02050-100,200, 400; 02050501-2; 02060200; 02050504
Therapy for heart failure (loop diuretics)	02020200

5.3 Validation of clinical events

A **Study-Within-A-Trial (SWAT)** will operate alongside the main DaRe2THINK trial involving patients under the care of the University Hospitals Birmingham (UHB) NHS Foundation Trust (see Table 4 for summary of the SWAT). Including four hospitals, UHB is one of the largest healthcare providers in Europe, treating more than 2.2 million patients each year. The large geographical footprint and provision of secondary, tertiary and quaternary care provides a unique ability to capture a wide variety

of endpoints in patients recruited in the West Midlands. UHB has one of the most sophisticated electronic healthcare systems in the world, which has been in active use at for over 10 years. This includes the Patient Information Communication System (PICS), which integrates prescribing, drug administration, observation charting, diagnostic coding, laboratory and radiology tests. These data are used to enhance safe and efficient patient care through rules-based clinical decision support.

For patients recruited in DaRe2THINK, we will search our integrated healthcare record across the four hospitals for pertinent data, *for example an admission due to stroke or an outcome related to dementia*. Searches will be based on NHS number, date of birth and name (recruitment for the trial includes informed consent for NHS data mining). We will systematically identify relevant clinical datasets including healthcare records, clinical notes, imaging and time-series data. These structured, semi-structured and unstructured datasets will be aligned, harmonised and integrated across various modalities before application of artificial intelligence behind the NHS firewall. Using Natural Language Processing and text mining approaches, we will generate an automated, semantic characterisation of clinical endpoints for each patient and code them using the International Classification of Diseases (ICD) framework. This will be compared with the coded data outcomes obtained through CPRD (primary care) and HES (secondary care) to generate a statistical representation of the frequency and accuracy of outcomes. *In the example on stroke, we will collect information on the validity of the stroke outcome, admission duration, the type of stroke (ischaemic or haemorrhagic), confirmatory imaging reports, interventions or relevant treatments, and outcomes (discharge status, in addition to secondary events such as subsequent myocardial infarction or recurrent stroke). For dementia, we will collect causation (vascular, Alzheimer's, other), confirmatory imaging for these causes, mini-mental state examination scores, discharge location, etc.* In the same way, we will extract relevant hospital-level data for other components of the primary outcome and safety outcomes, providing a broad validation that will be applicable and useful to future NHS-embedded research.

Table 4: Data validation Study-Within-A-Trial

SWAT design	Details
Population	Sub-study of DaRe2THINK patients with data held within the UHB network.
Clinical events of interest	Mortality, cerebrovascular events, thromboembolic events, myocardial infarction, vascular dementia, intracranial bleeding or any other clinically-relevant bleeding.
Methodology of data extraction	Automated machine learning approaches behind the NHS firewall, including all clinical noting, imaging reports, clinical measurements, laboratory results and therapeutics (medications and interventions); staff blind to randomised treatment.
Comparators	Coded data obtained from primary and secondary care (CPRD and HES).
Time period	Same patient timeframe as the DaRe2THINK trial.
Outcomes	1. Accuracy of the primary care database; percentage of patients with correct coding.<br>2. Accuracy of HES; percentage of patients with correct coding using a) primary codes, b) secondary codes and c) both primary and secondary codes.<br>3. Additional unreported events; percentage of patients with clinical events of interest that are missing from either primary or secondary care datasets.

Adjudication	An independent clinical team will adjudicate any discrepancies identified between the UHB network data and those coded in CPRD or HES.
Statistical analysis	Summary data, inter-curator agreement and kappa statistics.
Impact & Value	1. To the DaRe2THINK team to assist the process of trial outcome analysis and reporting at the end of the trial.<br>2. To global researchers on the value of NHS coded data and relevant limitations.<br>3. Potential enrichment of patient outcomes using a systematic and automated process than can be applied to future research and clinician decision support.
Registration	The SWAT will be prospectively registered with the MRC Northern Ireland Hub for Trials Methodology research.
Dissemination	Interim (internal) report at month 36; Final (published) report submitted after trial data collection has completed at month 60.

5.4 Patient-reported cognitive function

DaRe2THINK will use technology solutions to provide 'no-visit' follow-up of cognitive function, which will substantially lower participant burden in order to maximise response rates. To limit any technology bias, the system is designed for use by a range of devices including all smartphones, tablets, laptops and personal computers. Should a patient not have access to such a device, they will be able to complete these tests at a library, or through a friend or family member's device. Proxy completion of cognitive testing will not be permitted. A PPI-led focus group of public advisors will appraise and help to improve our technology approaches.

DaRe2THINK will use the Online Questionnaire Sharing Service developed by the Nuffield Department of Population Health at the University of Oxford. In brief, this system will allow a bespoke and simple web-based interface for patients to access and complete a range of cognitive function tests that measure different cognitive domains. These tests are based on those performed as part of the UK Biobank study.[39] The results of the cognitive function test will not be returned to participants and we will make it clear that these tests are for research purposes only, and will not be reviewed by external parties or the Primary Care team.

Patients will access the online questionnaire through a customised web-link (provided by the University of Oxford) that is texted and emailed to consenting study participants. Due to the nature of the tests, only online completion of cognitive testing is available, with source data provided entirely by the participant with no editing capability by the Sponsor. No personal data (for example, mobile phone number or email address) will be shared with the University of Oxford (or any other entity) and will remain restricted behind our firewall. The web-link incorporates a timestamp and a numeric participant ID, and only the DaRe2THINK study team will be able to link the corresponding study ID with a participant. Data from the online cognitive function questionnaires are stored on a secure server at the University of Oxford and are made available to the DaRe2THINK research team via a Secure File Transfer Protocol on a periodic basis.

The cognitive testing battery assesses several aspects of cognition known to be sensitive to ageing and which are often precursors to the diagnosis of a range of neurodegenerative conditions (particularly dementia), such as processing speed and non-verbal reasoning. Items have been

selected from existing cognitive batteries (e.g. the Cardiff Cognitive Battery and COGNITO [40,41]) and have been developed by recognised experts for use in large-scale population-based cohorts, such as UK Biobank. The specific tests that DaRe2THINK will use include:

1. Fluid intelligence/reasoning test: This test gathers data on verbal and numerical reasoning. Respondents are asked fourteen logical verbal and numerical questions. Each question has five possible answers. The score is the number of correct answers provided within two minutes, with incorrect or unattempted questions scored zero.
2. ‘Trail making’ test: This test assesses visual attention and provides information on visual search, scanning, speed of processing, mental flexibility, and executive function. Respondents must link circles in order by clicking on the next item in the sequence. There are two presentations; one using numbers and one using numbers and letters.
3. Symbol digit substitution: This code-breaking test measures complex processing abilities. Respondents are presented with one grid linking symbols to single-digit integers and a second grid containing only the symbols. They are then asked to indicate the numbers attached to each of the symbols in the second grid, using the first one as a key.
4. Matrices: This test measures non-verbal fluid reasoning. Respondents are presented with a logically constructed design that is missing a piece. They must choose the piece that completes the design from different alternatives. The items start easy and become progressively more difficult.

5.5 Patient-reported quality of life

Similar to cognitive function, questionnaires will be delivered directly to patients via text message to their mobile phone and email to their nominated address (but on a 6-monthly basis). This can be filled in by participants using any digital device or computer with internet access, at home or place of participant preference. Where data are not received, the system will automatically send reminders to the patient for timely completion. After 3 reminders, the Trial Coordinator will telephone the participant to ensure receipt and willingness to continue in the study. Proxy completion by someone other than the trial participant is not permitted. Data will be housed securely within a REDCap database at the University of Birmingham. The system requires a mandatory set of questions to be completed to avoid missing data. It will be made clear to all participants (in the participant information sheet, consent form and on the electronic system) that patient-reported outcomes are for research purposes only, and will not be reviewed by medical staff to inform their care. In rare cases where online completion of quality of life cannot be completed (e.g. disability or blindness), alternative arrangements such as verbal or paper responses will be explored. Any transcribed data will be clearly noted as such on the case report system and logged with the users access details (see section 9.1).

The EQ-5D-5L questionnaire is a valid, reliable, responsive measure, where respondents rate their health on 5 dimensions on the day of completion (mobility, self-care, usual activities, pain/discomfort and anxiety/depression), in addition to a visual analogue scale of health perception.[42,43] It is a generic health questionnaire available in a range of languages and widely used across different disciplines and for quantification of health economic benefit. Although not specific to AF, we have already used and tested this questionnaire within an RCT, with focus groups in patients with AF confirming ease of completion.[25] AF-specific questionnaires are much more complex, may not be

suitable for completion without assistance from research staff, and have a number of concerns regarding validation and methodology.[44]

5.6 Additional patient-reported events

Concurrent with the questions on quality of life at 6-monthy intervals collected remotely on REDCap, all participants will be asked:

1. If they are currently prescribed any blood thinning (anticoagulant) therapy (specifying acenocoumarol, apixaban, dabigatran, edoxaban, phenindione, rivaroxaban and warfarin to ensure participants do not confuse with antiplatelet therapy). If ticked, participants will then be asked whether their compliance with medication in the previous two weeks is (a) taking all tablets; (b) missing one dose; (c) missing more than one dose; (d) stopped/paused on their own decision without medical advice; or (e) stopped/paused on medical advice. If options (c) or (d) are selected, then participants will automatically be advised that safe and effective treatment (in particular prevention of stroke and blood clots) requires good adherence to tablets, and to speak to their GP if they have concerns.
2. If they have moved to another GP, or are considering a move to another GP in future. If ticked, participants will automatically be advised that the Trial Coordinator will arrange a telephone call to discuss the implications on their participation in the trial.

5.7 Coordination of data processes

All aspects relating to data, data processing and security will be managed by a specific group of experts in their respective fields who will convene at regular intervals. The Data Coordination Team (see members on page 7) will also have remit to ensure that any processes developed for this trial can be applied to future DaRe2 studies.

6 SAFETY REPORTING

6.1 Overview

DaRe2THINK will employ a risk-adapted and pragmatic approach to adverse event reporting. The rationale for this is: (1) Collection of HES outcomes will only occur on a yearly basis; (2) Events are captured from routine NHS care coding, meaning that events have already been identified and managed within the NHS; (3) Hospitalisation, prolongation of hospitalisation and death are specified outcomes or components of outcomes in the trial; (4) GPs in England are already experienced in the prescription and monitoring of patients taking DOAC therapy, for AF as well as other clinical indications such as venous thromboembolism; and (5) the DOACs used as interventional therapy in this trial have an established safety profile in patients with AF. This includes RCTs, where DOACs have been extensively studied in numerous large phase III trials, including 42,411 participants receiving a DOAC compared to 29,272 randomised to warfarin in the landmark trials for apixaban, dabigatran, rivaroxaban and edoxaban in AF (ARISTOTLE, RE-LY, ROCKET AF and ENGAGE AF TIMI 48).[8] . Real-world safety data have also been extensively reported on, as summarised by the European Medicines Agency Committee for Medicinal Products for Human Use. DOAC use was assessed in eight databases which include a total of 186,405 new users with AF, 156,636 users in a meta-analysis of population-based AF cohorts, and 407,586 new users of DOACs across other clinical indications.[45] These data reflect that the DOAC class have been one of the most intensively studied group of therapeutics in the contemporary era. As indications for DOAC therapy continue to broaden [46], clinicians across the NHS have daily experience in their use allowing a risk-proportionate approach in this trial.

6.2 Definitions

Standard definitions for different types of adverse events are listed in Table 5.

Table 5: Definitions of adverse events

Term	Definition
Adverse Event (AE)	Any untoward medical occurrence in a participant to whom a medicinal product has been administered, including occurrences which are not necessarily caused by or related to that product.
Adverse Reaction (AR)	An untoward and unintended response in a participant to an investigational medicinal product which is related to any dose administered to that participant.<br><br>The phrase "response to an investigational medicinal product" means that a causal relationship between a trial medication and an AE is at least a reasonable possibility, i.e. the relationship cannot be ruled out.<br><br>All cases judged by either the reporting medically qualified professional or the Sponsor as having a reasonable suspected causal relationship to the trial medication qualify as adverse reactions. It is important to note that this is entirely separate to the known side effects listed in the Summary of Product Characteristics (SmPC). It is specifically a temporal relationship between taking the drug, the half-life, and the time of the event or any valid alternative aetiology that would explain the event.

Serious Adverse Event (SAE)	A serious adverse event is any untoward medical occurrence that:<br>• results in death<br>• is life-threatening<br>• requires inpatient hospitalisation or prolongation of existing hospitalisation<br>• results in persistent or significant disability/incapacity<br>• consists of a congenital anomaly or birth defect<br>Other 'important medical events' may also be considered serious if they jeopardise the participant or require an intervention to prevent one of the above consequences.<br>• NOTE: The term "life-threatening" in the definition of "serious" refers to an event in which the participant was at risk of death at the time of the event; it does not refer to an event which hypothetically might have caused death if it were more severe.
Serious Adverse Reaction (SAR)	An adverse event that is both serious and, in the opinion of the reporting Investigator, believed with reasonable probability to be due to one of the trial treatments, based on the information provided.
Suspected Unexpected Serious Adverse Reaction (SUSAR)	A serious adverse reaction, the nature and severity of which is not consistent with the information about the medicinal product in question set out in the reference safety information:<br>• in the case of a product with a marketing authorisation, this could be in the SmPC for that product, so long as it is being used within its licence. If it is being used off label an assessment of the SmPCs suitability will need to be undertaken.<br>• in the case of any other investigational medicinal product, in the investigator's brochure (IB) relating to the trial in question

6.3 Operational use within a pragmatic NHS-embedded trial

To limit unnecessary time spent by frontline NHS staff for a class of drugs with an established safety profile, DaRe2THINK will operate a risk-adjusted approach to safety reporting.

SAEs and SARs will not be reported in an expedited fashion. In particular, major and minor bleeding, hospitalisation (any cause), prolongation of hospitalisation and death will not be reportable SAEs as they are nominated outcomes of the trial. The RSI for DaRe2THINK will be Section 4.8 of the SmPC for apixaban, as the exemplar of the DOAC class with the most clinical experience within the UK (available at https://www.medicines.org.uk/emc/product/2878/smpc). Operationally, outcomes from CPRD/HES will be matched against this list on a yearly basis and a summary table of SAEs/SARs generated and reported to the Data Monitoring Committee, Trial Steering Committee, Sponsor and MHRA. If SAEs are reported beyond a pre-defined limit, this will trigger more frequent reporting. The precise trigger points for this eventuality will be set by the independent Data Monitoring Committee at their first meeting, and ratified by the Trial Steering Committee, but would be expected to be twice the rate observed in the SmPC (for example major gastrointestinal bleeding rate of 1.52% per year). A multifaceted 'safety net' process will also occur to ensure that important safety events are not missed (detailed below) and to develop trial processes for future DaRe2 pipeline randomised trials.

The CPRD IRSP allows Investigators to directly input a potential SAE for any recruited participant. This entry is then automatically flagged to the CPRD team and Trial Coordinator to process the potential SAE, with expectedness determined by the Chief Investigator, Deputy Chief Investigator or

delegate, as described in section 6.8. Source data for the SAE form is controlled by the Investigator with no editing rights by CPRD, Sponsor or CI. Any amendments or updates by the Investigator lead to the creation of a new linked SAE form with a clear audit trail.

Specific procedures are in place to deal with incident intracranial events, major bleeds and dementia outcomes. Identification and delineation of strokes will be made using a bespoke system within the CPRD Interventional Research Services Platform (IRSP), which will ask Site PIs to classify any new intracranial events according to type: (1) ischaemic stroke; (2) epidural hematoma; (3) subdural hematoma; (4) subarachnoid haemorrhage; and (5) intracerebral haemorrhage. A similar process will occur for categorisation of non-intracranial major or clinically relevant bleeds, depending on the anatomical site and clinical consequences. For dementia outcomes, categories are: (1) Alzheimer's disease; (2) vascular dementia; (3) Lewy Body disease; (4) Fronto-temporal dementia; (5) mixed dementia; and (6) other causes (e.g. due to alcohol, Parkinson’s disease and viruses). As with other events, further detail will be collated from CPRD and HES.

Due to the age criteria of the trial, it is not possible that pregnancy will occur in the recruited population. Nevertheless, pregnancy is not considered an adverse event unless a negative or consequential outcome is recorded for the mother or child/foetus (which if serious would be considered an SAE). DOACs are not used/stopped in patients who become pregnant in favour of low molecular weight heparin where thromboembolic risk is elevated.

As with any potential adverse reaction in the NHS, Investigators will be encouraged to complete an MHRA Yellow Card submission, but these will not be collected as part of trial data. The MHRA’s Yellow Card Scheme is a national system for collecting and monitoring information on suspected adverse drug reactions by health professionals and patients. Since February 2020, reporting of suspected ARs to the Yellow Card Scheme has been rolled out across England, and is integrated into practices that use the EMIS web medical record platform.

6.4 Reporting of SUSARs

Due to the immense volume of safety data collected on this class of drugs, and their common use in NHS routine practice, it is improbable that new SUSARs will be identified for DOACs in this trial. Data collection in DaRe2THINK will operate entirely from NHS coded outcomes from primary and secondary care; the purpose of which is to enable a more efficient approach to clinical trials within the NHS. As such, it is not likely that further details of any SAE will be available to the central study team, and any action/outcomes may only be known at the next yearly data collation point. Although this limits the value of expedited reporting from either a safety or regulatory perspective, the processes in place still meet the Sponsor’s legal obligations in terms of SUSAR reporting.

If an Investigator believes a particular SAE in a participant receiving DOACs is both unexpected and potentially due to the DOAC, they will be asked to complete a SAE report page on IRSP as soon as possible after becoming aware of the event. Once recorded on IRSP, a report is generated which is immediately sent to the Sponsor, Trial Coordinator and the CPRD study team. The Investigator will also be required to telephone the Trial Coordinator within 72 working hours of becoming aware of the event. On receipt of the report, CPRD, in collaboration with the Trial Coordinator, will liaise with the CI to formally assess the event, and where assessed as a possible SUSAR, will be reported in line with regulatory guidelines. As in most cases this will be the result of a hospital admission being coded into

the Primary Care record, this will provide time for receipt of documentation from the secondary care provider and to account for part-time GPs.

All SUSARs occurring from the time of randomisation until the end of the study must be recorded on the relevant form and sent to the Sponsor by the Chief Investigator/Deputy Chief Investigator or delegate within 7 days of the research staff becoming aware of the event. Any SUSAR will need to be reported to the Sponsor irrespective of how long after administration the reaction has occurred until resolved. The sponsor will inform the MHRA and REC within the required expedited reporting timescales.

For each SUSAR the following information will be collected:

- Full details in medical terms and case description (if applicable and known).
- Event duration (start and end dates, if applicable and known).
- Vital status of the patient (where known).
- Opinion on causality (i.e. relatedness to the DOAC).
- Seriousness criteria.
- Confirmation that the event is unexpected.

6.5 Safety net to capture adverse events

To guard against unreported events, DaRe2THINK will operate a safety net process to capture additional potential SAEs. CPRD receives daily data collections from General Practices, which are processed into a secure database. For participants recruited into DaRe2THINK, this data will be made available for regular, frequent searches. Such searches could identify key new events occurring in the patient's coded electronic health record, including potential SAEs such as major bleeding. Upon identification of such events, the Site PI will be notified by email and asked to undertake additional activity, for example to record details of the potential SAE within the IRSP. This process will work in concert with the extraction of coded data within primary and secondary care, and will feed into the safety reporting processes described above. It will also assist the development of the DaRe2 pipeline for future trials with higher-risk CTIMPs. As a backup procedure, paper SAE forms will also be available at all sites and held as part of the Site File content (for example, in case of system/internet failure, or if a participant moves their Primary Care provider to another CPRD practice). Training regarding SAE reporting via IRSP and via the back-up paper route will be provided to all Investigators as part of site initiation. If a SAE needs to be reported via the back-up route, the Investigator will complete a paper SAE form and return it as a PDF to CPRD and the Trial Coordinator. A copy would also be sent to the Sponsor. All data from both SAE reporting routes will be transcribed into a SAE line listing document, with data entry being entered and checked by two different members of the CPRD team. All correspondence and PDFs associated with reported SAEs will be held in the Trial Master File.

6.6 Responsibilities of the Principal Investigator

1. Ensuring that any AEs or ARs are accurately recorded in the GP record when participants attend for treatment/follow-up, and the CPRD IRSP where applicable.

2. Completion of SAE documentation as soon as practicable, and to communicate any potential SUSARs to the Trial Coordinator within 72 working hours.
3. For incident intracranial events, major bleeding and dementia, using medical judgement in assigning seriousness, causality and aetiology.
4. Standard adverse reaction reporting as per clinical norm, using the MHRA Yellow Card system.

6.7 Responsibilities of the Clinical Practice Research Datalink team

1. Configuring and maintaining IRSP to support the reporting of SAEs by Investigators.
2. Configuring and maintaining the IRSP to support intracranial events, major bleeds and dementia reporting by Investigators.
3. Collating the information required to produce safety reports for the Chief Investigator, Sponsor and oversight committee(s).
4. Providing the annual data extract to support SAE analysis.
5. Following up SAEs, intracranial events, major bleeds and dementia reports with the Investigator until resolution or end of trial (as applicable).
6. Supporting timely and accurate submission of SUSARs.
7. Collaborate with the CI to submit the Development Safety Update Report (DSUR).
8. Generating the Trial Monitoring Plan and Safety Monitoring Plan.

6.8 Responsibilities of the Chief Investigator/ Deputy Chief Investigator or delegate

1. Clinical oversight of the safety of patients participating in the trial, including an ongoing review of the risk / benefit.
2. Using medical judgement in assigning the SAEs seriousness and causality (in line with the Reference Safety Information) where it has not been possible to obtain local medical assessment. This includes expectedness of SARs against the RSI.
3. Immediate review of all SUSARs.
4. Review of specific SAEs and SARs such as intracranial haemorrhage, in accordance with the trial risk assessment and protocol as detailed in the Trial Monitoring Plan.
5. Assigning Medical Dictionary for Regulatory Activities (MedDRA) or Body System coding to SAEs.
6. Provide a DSUR once a year throughout the clinical trial, or as necessary, to the Competent Authority (MHRA), and where relevant the REC and sponsor. The report will be submitted within 60 days of the Developmental International Birth Date (DIBD) of the trial each year until the trial is declared ended. In the context of this pragmatic NHS-embedded trial (and where hospitalisation is common in this patient group), the DSUR will provide a condensed format of SAEs.
7. Central data collection of safety data according to the trial protocol onto a database.
8. Annual checking of updates to the Reference Safety Information.

9. Give immediate written notice to the MHRA and the relevant REC of any urgent safety measures taken and the circumstances giving rise to those measures.

6.9 Responsibilities of the Sponsor

1. Ensuring reporting safety information to the CI, delegate or independent clinical reviewer for the ongoing assessment of the risk / benefit according to the Trial Monitoring Plan.
2. Ensuring reporting safety information to the independent oversight committees identified for the trial (Data Monitoring Committee and / or Trial Steering Committee) according to the Trial Monitoring Plan.
3. Ensuring expedited reporting of SUSARs to the MHRA and REC within required timelines.
4. Ensuring standard tables and other relevant information for the DSUR are prepared in collaboration with the CI and ensuring timely submission to the MHRA and REC.

6.10 Responsibilities of the oversight committees

In accordance with their relevant Charters, the DMC are responsible for periodically reviewing overall safety data to determine patterns and trends of events, or to identify safety issues which would not be apparent on an individual case basis; and the TSC are responsible for periodically reviewing safety data and liaising with the DMC regarding safety issues. As noted, the primary source of safety reporting will be annual outcome data combining CPRD, HES and ONS matched against the SmPC for apixaban. In addition, the safety net reporting process described in section 6.5 will also be presented to the DMC and TSC.

7 STATISTICAL CONSIDERATIONS

7.1 Sample size derivation

The sample size has been calculated using actual NHS primary care data on 16,574 patients with AF not receiving anticoagulation and selected by our inclusion and exclusion criteria (2005-2018; THIN dataset). This provides real-world control group outcomes in patients similar to those we will recruit in DaRe2THINK. Rates of mortality, stroke, thromboembolism, myocardial infarction and vascular dementia per 100 person-years in patients matching enrolment criteria were 2.60, 0.78, 1.18, 0.40 and 0.10, respectively.

7.2 Primary outcome on composite clinical events

Applying these numbers conservatively to account for lower risk patients tending to enter clinical trials, our sample size calculation is based on a total of 4.0 events per 100 person-years (which includes a cardiovascular death rate of 1.5). The reduction in primary composite outcomes with DOAC therapy is estimated at 35% (includes a weighted mortality reduction of 17%). This is consistent with previous trial data [47] and is biologically plausible given the results of historical trials using warfarin versus no therapy [26]. The sample size calculation is for a time-to-event superiority analysis with 2 years of recruitment. To detect a hazard ratio of 0.65 with 90% power and a 2-sided alpha of 0.05, a total of 391 events will be required from an expected total of 2978 patients randomised (rounded up to 3000 patients). The sample size calculation includes a progressive crossover to anticoagulation in the control group (5%, 10%, 15%, 20% and 35% at the end of years 1 through 5 respectively), 15% cumulative withdrawal for DOACs, and loss to follow-up of 1% (negligible as data are collected through NHS care). The upper age limit of 73 years will ensure that at least 2 years of follow-up is possible in all patients until they reach the current age requirement for anticoagulant therapy of 75 years. However, we expect that mean age will be in the mid 60’s thereby providing sufficient follow-up time during the trial for primary outcome assessment. The sample size of 3000 patients will still provide 85% power if event rates are lower than expected (3.5% per year; other parameters identical; 335 events and 2896 patients).

7.3 Key secondary outcome on cognitive function

Cognitive function over time will be measured using the UK Biobank panel of tests that cover reasoning, visual attention, complex processing and reaction time (see Data Collection section on page 35 below).[39] Our analysis of UK Biobank data on 22,160 AF patients and 480,456 without AF shows the cognitive impact of AF is the same as 5 years of additional ageing.[unpublished] Using these values and accounting for missing data, 2000 patients would provide 80% power (2-sided alpha of 0.05) to detect an effect size of 12.5% of standard deviation using UK Biobank data (0.25 mean score difference in fluid intelligence), and 92% power to detect an effect size of 15% of standard deviation (0.30 mean score difference). Power will be enhanced by adjusting for baseline values; however this is a secondary outcome as the true impact of DOACs is currently unknown.

7.4 Data analysis overview

A Statistical Analysis Plan (SAP) will be drafted by the trial statistician, reviewed by senior statisticians at the Birmingham Clinical Trials Unit, and approved by the Trial Steering Committee. In view of the

open-label design of the trial, the SAP will be completed before participants are recruited. A brief outline of the analysis methods are given below.

The primary comparison groups will be composed of those who are randomised to intervention arm versus those randomised to the control arm. All analyses will be based on the intention to treat principle, i.e. all participants will be analysed in the groups to which they were randomised regardless of withdrawals or crossovers.

For all major outcomes, summary statistics and differences between groups (e.g. hazard ratio, mean differences, relative risks, etc.) will be presented with 95% confidence interval from two-sided tests. Analyses will be adjusted for the randomisation variables and baseline scores (where appropriate). There will be no adjustment for multiple testing.

7.5 Analysis of the primary outcome

Primary outcome data will be analysed as time-to-first event and will compare between treatment groups using survival analysis methods. Kaplan-Meier survival curves will be constructed for visual presentation of time-to-first-event comparisons. A Cox proportional hazard model will be fitted, and results will be expressed as the adjusted hazard ratio with 95% confidence intervals.

As a sensitivity analysis, we will perform a competing risk analysis for the primary outcome using the method of Fine and Gray; this will account for the competing risk of death where other outcomes (e.g. non-fatal thromboembolism or hospital admission) cannot occur in those patients that have died. A further sensitivity analysis will censor patients when reaching the age of 75 years, due to the indication for DOACs at this time point. A ‘per-protocol’ analysis as well as an ‘as-treated’ analysis will be performed for the primary outcome using prescription data from Primary Care. The ‘as-treated’ analysis will assess use of any anticoagulant, regardless of randomisation.

7.6 Analysis of secondary outcomes

Time to event outcomes (individual components of the primary outcome, all-cause mortality, etc.) will be analysed using the same methods as described for the primary outcome.

For continuous outcomes (cognitive function and EQ-5D-5L), a mixed-effects repeated measures analysis will be carried out on all data across follow-up. Results will be expressed as the adjusted mean difference with 95% confidence interval. Cognitive function analysis will formally compare completion rates between groups using survival analysis methods to determine if differential completion is evident. If this is the case, then further sensitivity analyses for this will be conducted, which will include imputation of missing data using pattern mixture models. EQ-5D-5L data will be scored according to the current NICE guidance (updated October 2019) [47]. Imputation of missing data will not be performed.

Count data (all-cause general practice visits, all-cause hospital admissions, etc.) will be analysed using a Poisson regression model (or negative binomial regression if there is evidence of overdispersion). An offset for the length of time the participant was in the trial will be included in the model. Results will be expressed as the adjusted incidence rate ratio with 95% confidence interval.

7.7 Planned Subgroup Analyses

Subgroup analyses will be performed for the primary outcome according to the DOAC agent prescribed, an age cut-off of 65 years, presence at baseline of heart failure, hypertension, chronic kidney disease and diabetes, COVID-19 diagnosis, the median baseline fluid intelligence cognitive function score, and by gender. The effect of these subgroups will be examined by including a treatment group by subgroup interaction parameter in the model. The results of subgroup analyses will be treated with caution and will be used for the purposes of hypothesis generation only.

7.8 Interim analyses and impact on sample size

Interim assessment of event rates will be performed on a yearly basis as part of the interim analysis for DMC to carefully map the event rate estimates with actual events. This information will allow the Data Monitoring Committee to make recommendations to the Trial Steering Committee on the need to reduce or expand the trial population without impacting on the primary outcome alpha. The Trial Steering Committee will remain blind to randomised group in any presented material.

7.9 Stopping Criteria

Appropriate criteria of proof beyond reasonable doubt cannot be specified precisely, but a difference of at least $p<0.001$ (similar to a Haybittle-Peto stopping boundary) in an interim analysis of a major endpoint may justify halting, or modifying, the study prematurely. If this criterion were to be adopted, it would have the practical advantage that the exact number of interim analyses would be of little importance, so no fixed schedule is proposed. Given the proposed use of the Haybittle-Peto boundary, no adjustment for multiple testing (to control the overall type I error rate) is proposed; hence, the threshold for statistical significance at final analysis will still be $p=0.05$.

A separate DMC reporting template will be drafted and agreed by the DMC including an agreement on which outcomes will be reported at interim analyses. The statistical methods stated in the Statistical Analysis Plan will be followed for the agreed outcomes.

7.10 Health economic evaluation

Type of economic evaluation: Cost-effectiveness will be assessed by the incremental cost per quality-adjusted life-years (QALY) gained.

Model structure and modelling framework: The probabilistic decision analytical model will comprise of two components; (1) a decision tree that captures the short-term clinical outcomes and costs associated with the treatment strategies for the duration of the trial follow-up period; and (2) a long-term Markov cohort model, which extrapolates the costs and outcomes over a lifetime horizon. The Markov model will characterise the course of the disease in terms of health states (for example, stroke, myocardial infarction, systemic embolism, major bleeding, clinically relevant non-major bleeding, etc.) and the possible transitions between them using 6-week cycles, where patients will accrue healthcare costs, life years, and QALYs.

Identification, measurement and valuation of outcomes: Clinical event rates in the model will be obtained from the DaRe2THINK trial and converted to risks per cycle, informing the transition probabilities between disease states. Beyond the trial period, mortality will be modelled based on age and gender-specific general UK life tables and a hazard ratio adjusting for the impact of AF. Patients will be assigned utility values according to their health states. EQ-5D-5L scores, measured from the

trial at baseline, will be used to establish baseline utilities. In addition to EQ-5D-5L data collected periodically during the trial period, utility inputs for the Markov model will be obtained from a UK-based utility catalogue [48], with utility weights obtained using the NICE-recommended mapping function.[49]

Identification, measurement and valuation of resource use: The model will accrue costs in several resource categories including treatment costs, management costs, acute care costs associated with clinical events, cost associated with other cardiovascular hospitalisations, and long-term social care costs. Resource use data collected in the DaRe2THINK trial will include dates on inpatient and outpatient care for clinical events, number of nights in hospital and interventional procedures (via linkage to HES), plus general practice visits and primary care treatment (via CPRD). Long-term social care and loss of productivity will be estimated from established literature. Baseline measures of resource use will be used to reduce the variance in incremental cost estimates. Cost data will be obtained from the NHS drug tariffs, the Monthly Index of Medical Specialties and NHS reference costs. The cost of productivity loss will be calculated using a human capital approach, which assumes that the production loss to society is equal to the value of lost earnings. This is a comprehensive approach where the maximum of potentially-possible production loss to society is estimated, and will be combined with extensive sensitivity analyses. Additionally, the treatment of elements of productivity costs other than paid work-time will be considered and discussed.

Analysis for health economic evaluation: Baseline characteristics of the patients in the intervention and standard of care groups will be summarised according to intention-to-treat. Differences in resource use and costs between the two groups will be tested using two-sample t-tests and Chi-squared tests for continuous and categorical variables, respectively. Cost-effectiveness of the intervention versus standard of care will be assessed using the incremental cost-effectiveness ratio (ICER) and the results will be presented in terms of point estimates, cost-effectiveness planes and cost-effectiveness acceptability curves. The standard NICE willingness to pay threshold for the NHS of £20,000 - £30,000 per QALY will be used for the base case analysis, with health and cost outcomes discounted by 3.5%. Univariate deterministic sensitivity analysis will be performed where each parameter will be varied according to the 95% confidence intervals and standard deviations (where applicable), while holding other parameters constant. Since main DOAC patents are set to expire during the trial period, a scenario analysis will also consider the impact of generic provision. Probabilistic sensitivity analysis will be performed to account for variability in outcomes due to statistical uncertainty in inputs. The values of key input parameters will be assigned a probability distribution and varied concurrently to generate ICERs by varying event rates, costs, utilities and risks simultaneously.

7.11 Analysis of virtual control data

Anonymised linked data will be provided by CPRD on individuals that meet the pre-screening automated selection criteria. Summary data will be compared between these ‘virtual’ control patients and patients in the randomised control group to assess for external validity of the trial cohort and to improve data processes and data flows for future studies. Full detail on statistical methods will be provided in the trial SAP.

8 ETHICAL, REGULATORY & GOVERNANCE ISSUES

8.1 Ethical framework

DaRe2THINK is designed to operationalise a national approach to NHS-embedded research. Extensive support will be provided by the NIHR CRN network, led by the West Midlands Primary Care team. Existing governance procedures are in place to ensure Good Clinical Practice, training and site initiation for multiple GPs and Practice Nurses at each site plus the Practice Manager. In addition, there are established processes for mail-out to screened patients, and potential additional opportunistic enrolment at GP appointments. The CRNs provide informed consent training and have ongoing relationships with community pharmacies for drug dispensing and in-practice monitoring. Each local CRN Primary care team is able to offer Research Facilitators and Research Nurses to assist with engagement with individual practices and stakeholders (Clinical Commissioning Groups, Super Partnerships, Federations, and more recently Primary Care Networks established as part of the Government's General Practice Forward View). These local teams will complete HRA approvals, facilitate delivery of the trial through their experience of clinical software systems, continually ensure recruitment targets are met throughout the lifecycle of the portfolio, and can screen, consent and collect patient data within a high-quality and ethical framework.

The DaRe2THINK protocol has been developed in accordance with the Standard Protocol Items for Randomized Trials (SPIRIT) [50] and SPIRIT-PRO guidelines.[51]

8.2 Ethical review

Before the start of the trial, approval will be sought from a Research Ethics Committee (REC) & local R&D for the trial protocol and other relevant documents. The study will be performed in accordance with World Medical Association recommendations, the Research Governance Framework for Health and Social Care, and applicable UK Statutory Instruments, which include the latest Data Protection legislation, the Human Tissue Act, and Guidelines for Good Clinical Practice (GCP).

Substantial amendments that require review by REC will not be implemented until the REC grants a favourable opinion for the trial (note that amendments may also need to be reviewed and accepted by the MHRA and/or NHS R&D departments before they can be implemented in practice at sites). All correspondence with the REC will be retained in the Trial Master File/Investigator Site File. An annual progress report (APR) will be submitted to the REC and Sponsor Research Governance office within 30 days of the anniversary date on which the favourable opinion was given, and annually until the trial is declared ended. It is the responsibility of the Chief Investigator or delegate to produce the annual reports as required and notify the REC and Sponsor of the end of the trial. If the trial is ended prematurely, the Chief Investigator or delegate will notify the REC and Sponsor, including the reasons for the premature termination. Within one year after the end of the trial, the Chief Investigator or delegate will submit a final report to the REC with any results, including any publications/abstracts.

8.3 Protocol review

Expert, independent review of the trial programme has been undertaken as part of the funders grant procedures. This protocol has been reviewed within the Sponsor's institution (University of Birmingham, including the Birmingham Clinical Trials Unit), our NHS partner (University Hospitals Birmingham NHS Trust), CPRD and the NIHR CRN West Midlands Primary Care team.

8.4 Public and Patient Involvement (PPI)

A PPI team of three individuals was set up in 2016 to initiate a range of studies and trials for patient benefit, coordinated by the University of Birmingham Institute of Cardiovascular Sciences. We are grateful to the NIHR CRN West Midlands and the NIHR Academy for providing funds to initiate and support ongoing PPI work.

This protocol has been developed in line with work performed by the PPI team on the DaRe2THINK trial and DaRe2 pipeline. Their involvement has centred on four specific areas: (1) Development of the overall concept of a data-enabled NHS trial, considering the needs of patients as research participants, the needs of the NHS and society in general, and the balance of give and take with respect to time (personal and family) and commitment involved in taking part in clinical research; (2) Issues relating to ethical concerns, data dissemination and data security; (3) Potential benefits to patients and the public of these innovations in trial process; and (4) The no-visit follow-up plans, including access, comfort and limitations to utilising technology solutions.

During DaRe2THINK, the PPI team will: (1) Advise the Trial Management Group on any potential concerns raised by participants; (2) Help to revise any patient resources after feedback from participants; (3) Co-design and help to evaluate the interpretation of trial results, especially patient-reported outcomes; (4) Invited to all Trial Steering Committee meetings, with two PPI members having permanent voting positions; and (5) Assist with dissemination of results and plain English summaries.

8.5 Regulatory compliance

DaRe2THINK will not commence until a Clinical Trial Authorisation (CTA) is obtained from the MHRA with favourable REC opinion. This protocol, and the conduct of any intervention testing, will comply with the Medicines for Human Use (Clinical Trials) Regulations 2004 and any relevant amendments.

Before any site can enrol patients into the study, the Chief Investigator/Principal Investigator or designee will ensure that appropriate approvals from participating organisations are in place. Specific arrangements are in place at Primary NHS Care sites in order to comply with the relevant guidance.

For any amendment to the study, the Chief Investigator or designee, in agreement with the sponsor, will submit information to the appropriate body in order for them to issue approval for the amendment. The Chief Investigator or designee will work with sites (R&D departments at NHS sites as well as the study delivery team) so they can put the necessary arrangements in place to implement the amendment to confirm their support for the study as amended.

9 DATA SECURITY

9.1 Data security at the University of Birmingham (coordinating institution)

DaRe2THINK will utilise the Research Electronic Data Capture (REDCap) system, originally developed by Vanderbilt University with ongoing support from the US National Institutes of Health. REDCap is a browser-based data capture software, supported by an experienced local team at the University of Birmingham. The system as deployed at the University of Birmingham is compliant with the Data Protection Act, and includes access restricted to nominated study staff, with clear audit trails for data/user monitoring. User rights per project are governed by the lead PI or nominated REDCap administrator. All access, changes and addition details are logged within a project's logging section.

REDCap is operated across two virtual servers hosted on the University network: a web application server and a MySQL database server. All University virtual severs are built to a secure standard. Daily backups of the server infrastructure are taken to allow fall backs to previous versions if required. A documented build process for installing REDCap and all security settings is followed by local college IT staff. The University servers sit behind a site firewall that helps protect access. The web application server has a secure connection through a specific firewall rule to the MySQL database server. The authentication to REDCap is via a University user account (LDAP), along with utilising REDCap's User allowlist. An administrator has to provide access to REDCap and a secure verification process is required before users can log on. Regular server security scans and reports are produced to identify any missing security patches. The report is emailed to system administrators with the relevant information for patches, upgrades or bug fixes. An external website security scan is checked via a third party website, the results are stored and can be viewed upon request. The REDCap database is backed up via the College Backup system using CommVault software. Backups occur daily, weekly and monthly and are stored in an offsite fireproof safe.

All incoming data In REDCap gets intentionally filtered, sanitized, and escaped. This includes all data submitted in an HTTP Post request and all query string data found in every URL while accessing REDCap, among other modes through which user-defined data gets submitted in the application. Server environment variables that are vulnerable to forgery by users are also checked and sanitized. All user submitted data is properly filtered for any possibly harmful mark-up tags (e.g. <script>) and is then escaped before ever being displayed on a web page within the application. SQL queries sent to the database server from REDCap are all properly escaped before being sent. If any values used in an SQL query originated from user-defined values, they would have already been sanitized beforehand as well, as described above. User-defined data used within SQL queries also have their data type checked to prevent any mismatching of data types (e.g. making sure a number is really a number). These processes of sanitization, filtering, data type checking, and escaping all help to protect against methods of attack, such as Cross-Site Scripting (XSS) and SQL Injection. To specifically protect against Cross-Site Request Forgery (CSRF), REDCap utilizes a "nonce" (a secret, user-specific token) on every web form used in the application. The nonce is generated anew on each web page as the user navigates within REDCap during a session.

With regards to remote e-consent, DaRe2THINK will utilise built-in features within REDCap, supported by the REDCap team at the University of Birmingham. The system displays the latest version of the REC-approved PIS and ICF, with the potential participant able to scroll through each document before electronically accepting and providing their name, date and e-signature (see also section 3.7). A PDF of the ICF is stored within the database for viewing by access-authorised users and automatically

emailed to the participant. The participant's NHS number is entered by the Investigator at the time of confirming the participant's consent on REDCap.

9.2 Data security at University Hospitals Birmingham (lead NHS institution)

Personal data for consented participants will be stored in a dedicated sever within the University Hospitals Birmingham NHS Foundation Trust infrastructure and behind the Health and Social Care Network (HSCN) firewall. This includes a copy of identifiers and patient-reported EQ-5D-5L collected on REDCap, outcome and safety data from CPRD/HES/ONS, and cognitive function data from the University of Oxford Online Questionnaire Sharing Service. All data and results will be backed up within the same environment and will be allocated to isolated data containers.

All patient level data will be stored for a minimum period of 5 years as per NHS requirements. Data access will be monitored and limited to authorised users who will be granted password restricted audited access through Secure Shell (SSH; a cryptographic network protocol). A Data Privacy Impact Assessment has been completed. No identifiable data will be moved, manipulated or stored outside of this environment.

9.3 Data security at CPRD

The CPRD platform enables secure screening of patients for recruitment and does not require sharing of any patient identifiers outside the practice. Data processing is conducted on a locked-down suite of servers with no external internet capabilities and yearly penetration tests.

CPRD IRSP servers are in one of two data centres, both operated by Server House. The data centres have climate control, redundant power (including generators), redundant connectivity, fire suppression and strict physical access controls. All visits must be pre-booked by identified individuals; photo ID must be presented, and records of this are maintained by the datacentre. Once within the facility, an individual's movements are controlled by a radio-frequency identification pass and monitored by closed-circuit cameras.

The CPRD network is segmented into two parts; an internal subnet that contains the machines in the cprd.hosted.dataline.co.uk Windows domain, and a demilitarized zone which contains the standalone internet-facing servers (e.g. webservers). There is also a link to IQVIA infrastructure. Strict firewall policies control the flow of traffic between the various zones, to control potential lateral movement on the part of an attacker, and to prevent unnecessary exposure of services

System administration is handled by IQVIA as a service contractor for CPRD/MHRA. User access to the IRSP environment is managed by the CPRD Interventional Research team. Access is granted based on the user's role (either within the Team, or as Study Investigators or patient end-users).

CPRD SOPs and policies are in place that define the policies and procedures for data access, external data transfer etc. A full list is defined in the trial Data Management Plan.

9.4 Data security for cognitive function

Data is collected by a dedicated Linux server protected by the main watchguard firewall at the Nuffield Department of Population Health (NDPH; University of Oxford). Information collected is stored within a protected relational database on a separate system which is not directly accessible from the internet.

The hardware storage components are part of the NDPH virtual system which is bolted into locked racks and in a location protected by three physical layers of entry checks. All information is linked solely to unique one-off session keys which have no relationship to participant identifiers. The linkage between these session keys and participant identifiers is held remotely by the DaRe2THINK research team and is unknown to both the online server and the NDPH staff managing the service. Information gathered is pre-coded wherever practicable during the input process and any residual free-text information is protected by AES 256 encryption.

9.5 Data security for health economic analysis

Pseudonymised data provided to the health economics team will reside within secure servers at the London School of Economics (LSE) Primary Data Centre. LSE encrypts the data link between its on-campus firewalls and its firewalls hosted within its own equipment in the Data Centre; there is no facility for the traffic to be intercepted and decrypted. LSE encrypts the traffic using AES 256 between client machines and servers storing the data. All ingress to and egress from the LSE network is controlled by use of next-generation firewalls which are Common Criteria EAL4 compliant. Access to LSE resources is governed by the stringent Access Control Policy of the university.

9.6 Data protection and patient confidentiality

All investigators and trial site staff must comply with the requirements of the Data Protection Act and General Data Protection Regulation with regards to the collection, storage, processing and disclosure of personal information and will uphold the Act’s core principles. Access will be limited to the minimum number of individuals necessary for quality control, audit and analysis. The controller of the data is the University of Birmingham, and all staff are expected to comply with this institution’s Standard Operating Procedures.

In brief, all data will be used in line with the Act, for example the principles of: (1) Fair, lawful and transparent use by only using anonymised data for analysis; (2) Explicit use of this data for the purposes of health improvement in specified patient subgroups; (3) Relevant and limited use of data to what is necessary to answer the research questions; (4) Applying of our established data pipelines to ensure accuracy, and identify and rectify anomalies; (5) Keeping data for no longer than is necessary and permit collaboration/data sharing with other research groups, where applicable, to ensure the full extent of value from the data obtained; (6) Handling data in a way that ensures security and prevents loss or misuse; and (7) Technical and organisational procedures in place to ensure accountability, in addition to PPI input on research questions and data use. These approaches are also consistent with the European General Data Protection Regulation.

10 OVERSIGHT & MONITORING

10.1 Trial Management Group

The Trial Management Group (TMG) will be responsible for the day-to-day running and management of DaRe2THINK and will convene at regular intervals (see members on page 7). The TMG will delegate specific work around data processes and data security to the Data Coordinating Team.

10.2 Trial Steering Committee

The role of the Trial Steering Committee (TSC) is to provide the overall supervision of the trial, monitor trial progress and conduct, and advise on scientific credibility of potential interventions. The TSC will meet at least annually, and comprise of at least 75% independent members (including an independent chair and PPI representatives), as per the funder's policies. The TSC will consider and act, as appropriate, upon the recommendations of the Data Monitoring Committee (DMC). Further details of the remit and role of the TSC are available in the respective charter (see members on page 7).

10.3 Data Monitoring Committee

An independent DMC will be established to oversee the safety of participants in the trial. The DMC will meet prior to the trial opening to the first intervention, and then meet at least annually, or as per a timetable agreed by the committee at the first meeting. Data analyses will be supplied in confidence to the DMC by the trial statistician. The DMC will be asked to give advice on whether the accumulated data from the intervention, together with the results from other relevant research, justifies the continuing recruitment of further participants. The DMC will operate in accordance with their respective charter and will consist of an independent Chair, and independent clinician and an independent statistician (see members on page 7).

10.4 Expert Advisory Group

An Expert Advisory Group (EAG) will support the TMG by providing expertise in pragmatic clinical trials and integration with the NHS, with a focus on developing sustainable methods. The EAG consists of UK leaders in health data research, anticoagulation, cognition and clinical trials (see members on page 7). The EAG will formally meet every 6 months, or as specified by the Chair.

10.5 Protocol deviations

Prospective, planned deviations or waivers to the protocol are not allowed under UK regulations on Clinical Trials and must not be used; for example, it is not acceptable to enrol a participant if they do not meet the eligibility criteria or restrictions specified in this protocol.

Only major deviations from the protocol will be collated and registered, for example a deviation from consent processes or in the administration of the intervention. To ensure patients that are randomised to treatment receive it, daily data flows to CPRD will be periodically tracked to facilitate study monitoring.

Where needed, processes are in place to escalate breaches for immediate action by the central coordination team.

A "serious breach" is a breach which is likely to effect to a significant degree either (a) the safety or physical or mental integrity of the participants of the trial; or (b) the scientific value of the trial. Where this occurs during the trial, the Sponsor will be notified immediately, and the Sponsor will ensure the licensing authority is notified in writing of any serious breach of (a) the conditions and principles of GCP in connection with that trial; or (b) the protocol relating to that trial, as amended from time to time, within 7 days of becoming aware of that breach. The Chief Investigator Agreement identifies tasks delegated to the CI on the sponsor's behalf.

10.6 Data access

Direct access will be granted to authorised representatives from the Sponsor, host institution and the regulatory authorities to permit trial-related monitoring/audits/inspections, in line with consent.

10.7 Archiving

Archiving will be authorised by the Sponsor following submission of the end of trial report, and will include relevant trial documents, the trial database and all essential material for a minimum of 25 years after completion of the trial. Archiving and destruction of documents will follow the Sponsor's Standard Operating Procedures.

10.8 Trial monitoring

A Trial Monitoring Plan will be developed and agreed by the Trial Management Group based on the trial risk assessment. Monitoring will be kept to the minimum needed to ensure compliance and patient safety, relating to participant enrolment, consent, eligibility, allocation and reporting of harm.

Risk adapted trial monitoring will be carried out centrally and remotely via the CPRD IRSP based on an ongoing risk assessment process.

Central monitoring in the trial will be carried out through the IRSP and includes monitoring of recruitment, electronic case report form completion of SAEs, protocol deviations, and reportable outcomes.

Central site and recruitment monitoring will be undertaken via trial specific dashboards on IRSP.

Site visits will only be carried out where 'for cause' criteria are triggered (as defined in the Trial Monitoring Plan) but may include: (1) Quality concerns at site following central monitoring checks; (2) Identification of a potential risk to the trial; (3) Investigation into a potential serious breach of the trial protocol/GCP; and (4) Other reasons as recommended by the Sponsor.

Monitoring will be carried out by the CPRD study team who operate independently from the Sponsor and Principal Investigators. Where a triggered on-site monitoring visit is required, this will be carried out by CPRD staff not directly part of the day-to-day management team (Clinical Data Manager or equivalent). Monitoring reports will be compiled for each oversight committee as required.

10.9 Clinician responsibility

This study has no impact on any individual clinicians' responsibility to take immediate action if thought necessary to protect the health and interest of patients.

11 FINANCIAL CONSIDERATIONS

11.1 Trial funding

DaRe2THINK is funded through a Health Technology Assessment grant from the National Institute for Health Research and the UK Department of Health and Social Care (HTA 19/109 - NIHR130280). The funder has no role in trial design, conduct, data analysis, interpretation or manuscript writing, but will review any manuscripts prior to dissemination to ensure that they meet the funder's policies.

11.2 Payment to participants

Participants will not be paid for enrolment in the trial.

11.3 Insurance

The University of Birmingham has in place Clinical Trials indemnity coverage for this trial which provides cover to the University for harm which comes about through the University's, or its staff's, negligence in relation to the design or management of the trial and may alternatively, and at the University's discretion provide cover for non-negligent harm to participants.

With respect to the conduct of the trial at the Clinical Sites and other clinical care of the patient, responsibility for the care of the patients remains with the NHS organisation responsible for the Clinical Site and is therefore indemnified through the NHS Litigation Authority.

The University of Birmingham is independent of any pharmaceutical company, and as such it is not covered by the Association of the British Pharmaceutical Industry (ABPI) guidelines for participant compensation.

12 DISSEMINATION POLICY

12.1 Study teams

Regular newsletters will keep research staff and collaborators informed of the progress of the trial, and regular meetings will be held to report the progress of the trial and to address any problems encountered in the conduct of the trial. The Deputy CI will coordinate dissemination of data to the Primary Care research teams through the NIHR CRN.

12.2 Publications

All publications and presentations relating to the main trial, including abstracts, will be authorised by the TSC. All findings of clinical relevance will be submitted to a suitable medical journal for publication after peer review. The PPI team will provide a short lay summary of results that will be published alongside the scientific paper. Named authors on any publication must satisfy the International Committee of Medical Journal Editors (ICMJE) criteria for authorship (contribute to drafting of the article or revision for important intellectual content), provide timely approval of the final version to be published and supply detailed statements on any potential conflict of interest or financial relationships. Members of the group who do not fulfil ICMJE criteria for authorship will be listed in the article appendix.

12.3 Study participants

Participants will be sent a text message and email at the end of the trial providing a brief, plain English summary of the results written by the PPI team. We will also work with our local teams (patient engagement, volunteers and media) to publicise important results relevant to our community and through national press.

12.4 Community engagement

Relevant and accessible summaries of findings and presentations will be aimed at key stakeholder groups such as CCGs, Primary Care Networks (PCNs), General Practices, Royal Colleges, Medical Schools, professional societies (such as the British Cardiovascular Society and European Society of Cardiology), charities (such as the British Heart Foundation) and patient support groups (including the AF Association).

13 DaRe2THINK sub-studies

13.1 Validation of clinical events

Section 6.3 provides information on the Study-Within-A-Trial (SWAT) that will be used to validate clinical events.

This sub-study is funded within the original DaRe2THINK grant (NIHR Health Technology Assessment; NIHR-130280). The number of participants will depend on trial recruitment in the West Midlands local to the University Hospitals Birmingham NHS Foundation Trust.

13.2 Neuro-vascular imaging

DaRe2THINK-NeuroVascular(NV) is an observational sub-study of 160 patients, funded by the NIHR Efficacy and Mechanism Evaluation programme (NIHR-132974).

Background: Whilst it is clear that silent brain infarcts in patients with AF lead to a decline in cognitive function [52] and a higher risk of dementia [53], there is inconsistent evidence for the association of AF and specific changes on brain magnetic resonance imaging (MRI).[54] These studies were unable to account for anticoagulation use and the general vascular disease seen in patients with AF. Due to the randomisation to DOACs or usual care, DaRe2THINK provides an opportunity for unbiased assessment of the mechanistic effects of DOACs, as well as markers of future dementia risk. In a pooled analysis of three studies of 175 patients with AF at a median age of 60 years, 14 (8%) had evidence of a silent infarction on brain MRI prior to the procedure and white matter hyperintensities (WMH) were seen in 81 (46%).[55] In the SWISS-AF cohort of 1,390 AF patients with a mean age of 72 years but no history of stroke or transient ischaemic attack, infarcts were seen in 446 patients (32%) and WMH in 1,372 (99%). Of these, 694 (50%) had moderate WMH lesions (Fazekas scale ≥2).[56] Thus, brain injury is commonplace in patients with AF, and these lesions progressively accumulate over time leading to potential cognitive decline.[57] In a meta-analysis of eight observational cohorts (age range 71-82 years), patients with AF receiving anticoagulation had a lower incidence of cognitive impairment compared to those not prescribed an anticoagulant (relative risk 0.72; 95% CI 0.69-0.75).[16] However, observational data should be interpreted with caution due to considerable prescription biases; in a randomised controlled trial, warfarin did not reduce the rate of cognitive decline compared to aspirin in older patients with high risk of thromboembolism.[11] This sub-study will allow us to investigate if and how DOAC therapy used in younger patients with AF can prevent cerebrovascular disease and resultant cognitive impairment. Retinovascular imaging using optical coherence tomography (OCT) and OCT angiography will be used to determine if the mechanism of DOACs is related to cerebral lesions or due to effects on the general vasculature.

Approach: Patients randomised in DaRe2THINK and geographically near to the study site will be invited to a visit at baseline and at 3 years. Each visit (approximately 2 hours in duration) will include a brain MRI scan, retinal scans, cognitive function assessment using the Cambridge Neuropsychological Test Automated Battery (CANTAB), blood tests for cardiac biomarkers and a 12-lead electrocardiogram (ECG). Table 6 provides a detailed synopsis of the DaRe2THINK-NV study. Unless otherwise stipulated, all process will follow those noted for the main trial, including consent processes, data security, ethical approval, oversight and dissemination.

Table 6: DaRe2THINK-NV sub-study

Design	Details
Population	160 participants randomised in DaRe2THINK (80 participants from each arm with baseline imaging tests).
Inclusion criteria	Consents to join DaRe2THINK-NV within 3 months of randomisation in DaRe2THINK.
Exclusion criteria	1. Claustrophobia prohibiting the use of MRI.<br>2. Contraindication to MRI scanning due to implanted metal clips or devices with the potential to cause tissue damage in a magnetic field (e.g. aneurysm clips and certain cardiac pacemakers).<br>3. Weight over 30 stone or 190 kg (maximum limit for MRI scanner).
Time period	Participants of the main trial will be invited to baseline assessment within 3 months of randomisation. A follow-up visit will be performed at 3 years.
Site(s)	Queen Elizabeth Hospital; University Hospitals Birmingham (UHB) NHS Trust
Recruitment	DaRe2THINK participants who have agreed to additional studies on their consent form who reside within/near the West Midlands will be approached through text, email and telephone by the UHB study team. A patient information leaflet and video have been generated in collaboration with the PPI team. Remote e-consent will be performed as stipulated in section 3.7 using a specific DaRe2THINK-NV consent form after appropriate time to review the patient information leaflet.
Hypothesis	To elucidate if the mechanism of action of DOACs, in an effort to preserve cognitive function in patients with AF, is due to the prevention of cerebral white matter lesions, reduction in incident silent brain infarcts, protection of cognitive neural tracts, or an overall decrease in the burden of vascular disease.
Primary outcome	New cerebral infarction or at least moderate WMH (defined as Fazekas scale ≥2) on brain MRI at follow-up.
Key secondary outcome	Retinal capillary density of the radial peripapillary capillary network on OCT angiography at follow-up.
Other secondary outcomes	Neuroimaging outcomes:<br>• Grey/white matter volume and regional variation in cortical thickness/volume using high resolution T1 imaging<br>• WMH lesion count, volume and damage metric using T2/FLAIR imaging<br>• Microstructural integrity and tractography using diffusion-weighted imaging<br>• Cerebral microbleed count and vascular abnormalities on susceptibility-weighted imaging<br>Retinovascular outcomes:<br>• Vascular tortuosity, calibre, fractal dimensions, bifurcation angle and retinopathy on retinal fundus photography<br>• Retinal nerve fibre layer and ganglion cell-inner plexiform layer thickness, and macular volume using 'swept source or spectral domain'OCT

	• Vessel and perfusion density of the superficial, and deep vascular plexus on spectral domain OCT angiography<br><br>Cognitive outcomes:<br>• CANTAB cognitive testing battery, addressing executive function, attention, speed of processing and memory function<br>Health data outcomes:<br>• High-sensitivity troponin; B-type natriuretic peptide<br>• ECG: AF or other cardiac arrhythmias; heart rate; PR/QRS/QTc duration
Analysis	Image interpretation will follow standardised reporting templates for both cerebral and retinal images, using an established protocol that will be published on the trial website. A Statistical Analysis Plan (SAP) will be generated by the DaRe2THINK TMG and approved by the TSC/DMC prior to any analysis.<br>Analyses will follow an intention-to-treat approach based on the original randomised group, comparing the outcomes stipulated above between the DOAC and control groups. As we are primarily interested in the actual effects of anticoagulant therapy, each analysis will also use a modified 'as-treated' approach. Statistical methods are as described in Section 7.<br>Sensitivity analyses will be performed to exclude patients with existing moderate WMH for the primary outcome, and existing eye disease for the key secondary outcome (glaucoma, retinal vein occlusion or proliferative diabetic retinopathy). Additional sensitivity analyses will be performed of comorbidities with sufficient numbers of patients (e.g. hypertension).
Sample size calculation	Sample size is based on the aforementioned cohort studies [55,56] and our analysis of the THIN observational database of AF patients matching our trial criteria. With a conservative control prevalence for the primary outcome of 50%, a 50% reduction in cerebral thromboembolic disease from DOACs [26,47], 0.85 power and 2-sided alpha of 0.05, a total of 126 patients are required. A sample size of 160 patients would account for death/ withdrawal prior to the follow-up scan (estimated at 10%), cessation of DOAC therapy due to adverse events (estimated at 6%), and provide sufficient power for the key secondary outcome (>0.99 for an absolute difference of 6% in temporal radial peripapillary capillary density and 0.92 for a delta of 4%, informed by a prospective cross-sectional study [58].

14 REFERENCES

1. DA Lane, F Skjoth, GYH Lip, TB Larsen, D Kotecha. Temporal Trends in Incidence, Prevalence, and Mortality of Atrial Fibrillation in Primary Care. *J Am Heart Assoc.* 2017;6:e005155.
2. P Kirchhof, S Benussi, D Kotecha, A Ahlsson, D Atar, B Casadei, ... P Vardas. 2016 ESC Guidelines for the management of atrial fibrillation developed in collaboration with EACTS. *Eur Heart J.* 2016;37:2893-962.
3. D Kotecha, J Holmes, H Krum, DG Altman, L Manzano, JG Cleland, ... Beta-Blockers in Heart Failure Collaborative Group. Efficacy of beta blockers in patients with heart failure plus atrial fibrillation: an individual-patient data meta-analysis. *Lancet.* 2014;384:2235-43.
4. D Kotecha, CS Lam, DJ Van Veldhuisen, IC Van Gelder, AA Voors, M Rienstra. Heart failure with preserved ejection fraction and atrial fibrillation: Vicious twins. *J Am Coll Cardiol.* 2016;68:2217-28.
5. D Kotecha, G Breithardt, AJ Camm, GYH Lip, U Schotten, A Ahlsson, ... P Kirchhof. Integrating new approaches to atrial fibrillation management: the 6th AFNET/EHRA Consensus Conference. *Europace.* 2018;20:395-407.
6. P Kirchhof, KG Haeusler, B Blank, J De Bono, D Callans, A Elvan, ... L Di Biase. Apixaban in patients at risk of stroke undergoing atrial fibrillation ablation. *Eur Heart J.* 2018;39:2942-55.
7. LY Chen, FL Norby, RF Gottesman, TH Mosley, EZ Soliman, SK Agarwal, ... A Alonso. Association of Atrial Fibrillation With Cognitive Decline and Dementia Over 20 Years: The ARIC-NCS (Atherosclerosis Risk in Communities Neurocognitive Study). *J Am Heart Assoc.* 2018;7:e007301.
8. CT Ruff, RP Giugliano, E Braunwald, EB Hoffman, N Deenadayalu, MD Ezekowitz, ... EM Antman. Comparison of the efficacy and safety of new oral anticoagulants with warfarin in patients with atrial fibrillation: a meta-analysis of randomised trials. *Lancet.* 2014;383:955-62.
9. D Kotecha, CV Pollack, Jr., R De Caterina, G Renda, P Kirchhof. Direct Oral Anticoagulants Halve Thromboembolic Events After Cardioversion of AF Compared With Warfarin. *J Am Coll Cardiol.* 2018;72:1984-6.
10. National Institute for Health and Care Excellence. Atrial fibrillation: the management of atrial fibrillation. *NICE clinical guideline 180.* 2014: http://www.nice.org.uk/guidance/cg180/.
11. N Mavaddat, A Roalfe, K Fletcher, GY Lip, FD Hobbs, D Fitzmaurice, J Mant. Warfarin versus aspirin for prevention of cognitive decline in atrial fibrillation: randomized controlled trial (Birmingham Atrial Fibrillation Treatment of the Aged Study). *Stroke.* 2014;45:1381-6.
12. L Friberg, T Andersson, M Rosenqvist. Less dementia and stroke in low-risk patients with atrial fibrillation taking oral anticoagulation. *Eur Heart J.* 2019, 10.1093/eurheartj/ehz304.
13. Alzheimer's Research UK. Dementia UK update. 2019; https://www.dementiastatistics.org/.
14. JC Cowan, J Wu, M Hall, A Orlowski, RM West, CP Gale. A 10 year study of hospitalized atrial fibrillation-related stroke in England and its association with uptake of oral anticoagulation. *Eur Heart J.* 2018;39:2975-83.
15. SE Wolowacz, M Samuel, VK Brennan, JG Jasso-Mosqueda, IC Van Gelder. The cost of illness of atrial fibrillation: a systematic review of the recent literature. *Europace.* 2011;13:1375-85.
16. D Zeng, C Jiang, C Su, Y Tan, J Wu. Anticoagulation in atrial fibrillation and cognitive decline: A systematic review and meta-analysis. *Medicine (Baltimore).* 2019;98:e14499.
17. V Jacobs, HT May, TL Bair, BG Crandall, MJ Cutler, JD Day, ... TJ Bunch. Long-Term Population-Based Cerebral Ischemic Event and Cognitive Outcomes of Direct Oral Anticoagulants Compared With Warfarin Among Long-term Anticoagulated Patients for Atrial Fibrillation. *Am J Cardiol.* 2016;118:210-4.
18. OJ Ziff, DA Lane, M Samra, M Griffith, P Kirchhof, GY Lip, ... D Kotecha. Safety and efficacy of digoxin: systematic review and meta-analysis of observational and controlled trial data. *BMJ.* 2015;351:h4451.
19. MC Bahit, RD Lopes, DM Wojdyla, C Held, M Hanna, D Vinereanu, ... CB Granger. Non-major bleeding with apixaban versus warfarin in patients with atrial fibrillation. *Heart.* 2017;103:623-8.
20. NHS England. Next steps on the NHS five year forward view. 2017: https://www.england.nhs.uk/wp-content/uploads/2017/03/NEXT-STEPS-ON-THE-NHS-FIVE-YEAR-FORWARD-VIEW.pdf.
21. D Shukla. Opportunities to develop a research interest in general practice. *InnovAiT.* 2019;12:466-71.
22. A Wolf, D Dedman, J Campbell, H Booth, D Lunn, J Chapman, P Myles. Data resource profile: Clinical Practice Research Datalink (CPRD) Aurum. *Int J Epidemiol.* 2019;48:1740-g.

23. R Mathur, K Bhaskaran, N Chaturvedi, DA Leon, T vanStaa, E Grundy, L Smeeth. Completeness and usability of ethnicity data in UK-based primary care and hospital databases. *J Public Health (Oxf).* 2014;36:684-92.
24. D Kotecha, M Lainscak. Comorbidity (HFrEF and HFpEF) - Atrial fibrillation. In: AJ Camm, ed. *ESC Textbook of Cardiovascular Medicine 3rd edition*: Oxford University Press; 2018.
25. J Jones, M Stanbury, S Haynes, KV Bunting, T Lobban, AJ Camm, ... D Kotecha. Importance and Assessment of Quality of Life in Symptomatic Permanent Atrial Fibrillation: Patient Focus Groups from the RATE-AF Trial. *Cardiology.* 2020;145:666-75.
26. RG Hart, LA Pearce, MI Aguilar. Meta-analysis: Antithrombotic Therapy to Prevent Stroke in Patients Who Have Nonvalvular Atrial Fibrillation. *Ann Intern Med.* 2007;146:857-67.
27. Y Vinogradova, C Coupland, T Hill, J Hippisley-Cox. Risks and benefits of direct oral anticoagulants versus warfarin in a real world setting: cohort study in primary care. *BMJ.* 2018;362:k2505.
28. TG von Lueder, D Atar, S Agewall, JK Jensen, I Hopper, D Kotecha, ... VL Serebruany. All-Cause Mortality and Cardiovascular Outcomes With Non-Vitamin K Oral Anticoagulants Versus Warfarin in Patients With Heart Failure in the Food and Drug Administration Adverse Event Reporting System. *Am J Ther.* 2019, 10.1097/mjt.0000000000000883.
29. M Rohla, TW Weiss, L Pecen, G Patti, JM Siller-Matula, RB Schnabel, ... P Kirchhof. Risk factors for thromboembolic and bleeding events in anticoagulated patients with atrial fibrillation: the prospective, multicentre observational PREvention oF thromboembolic events - European Registry in Atrial Fibrillation (PREFER in AF). *BMJ Open.* 2019;9:e022478.
30. National Institute for Health and Care Excellence. Atrial fibrillation: diagnosis and management. *NICE clinical guideline 196.* 2021: www.nice.org.uk/guidance/ng196.
31. M Rienstra, SA Lubitz, S Mahida, JW Magnani, JD Fontes, MF Sinner, ... EJ Benjamin. Symptoms and Functional Status of Patients With Atrial Fibrillation: State of the Art and Future Research Opportunities. *Circulation.* 2012;125:2933-43.
32. SC Rivera, DG Kyte, OL Aiyegbusi, AL Slade, C McMullan, MJ Calvert. The impact of patient-reported outcome (PRO) data from clinical trials: a systematic review and critical analysis. *Health Qual Life Outcomes.* 2019;17:156.
33. Office for National Statistics. Internet access – households and individuals, Great Britain. 2019: Available at: ons.gov.uk; Release date 12 August 2019.
34. WP Wodchis, RS Bhatia, K Leblanc, N Meshkat, D Morra. A review of the cost of atrial fibrillation. *Value Health.* 2012;15:240-8.
35. E Herrett, AM Gallagher, K Bhaskaran, H Forbes, R Mathur, T van Staa, L Smeeth. Data Resource Profile: Clinical Practice Research Datalink (CPRD). *Int J Epidemiol.* 2015;44:827-36.
36. EM Burns, E Rigby, R Mamidanna, A Bottle, P Aylin, P Ziprin, OD Faiz. Systematic review of discharge coding accuracy. *J Public Health (Oxf).* 2012;34:138-48.
37. JK Quint, H Mullerova, RL DiSantostefano, H Forbes, S Eaton, JR Hurst, ... L Smeeth. Validation of chronic obstructive pulmonary disease recording in the Clinical Practice Research Datalink (CPRD-GOLD). *BMJ Open.* 2014;4:e005540.
38. KW Hagberg, SS Jick. Validation of autism spectrum disorder diagnoses recorded in the Clinical Practice Research Datalink, 1990-2014. *Clin Epidemiol.* 2017;9:475-82.
39. DM Lyall, CA Celis-Morales, J Anderson, JM Gill, DF Mackay, AM McIntosh, ... JP Pell. Associations between single and multiple cardiometabolic diseases and cognitive abilities in 474 129 UK Biobank participants. *Eur Heart J.* 2017;38:577-83.
40. J Gallacher, R Collins, P Elliott, S Palmer, P Burton, C Mitchell, ... R Lyons. A Platform for the Remote Conduct of Gene-Environment Interaction Studies. *PLOS ONE.* 2013;8:e54331.
41. K Ritchie, dR Guilhem., CW Ritchie, A Besset, V Poulain, S Artero, M-L Ancelin. COGNITO: Computerized Assessment of Information Processing. *Journal of Psychology & Psychotherapy.* 2014;4.
42. M Herdman, C Gudex, A Lloyd, M Janssen, P Kind, D Parkin, ... X Badia. Development and preliminary testing of the new five-level version of EQ-5D (EQ-5D-5L). *Qual Life Res.* 2011;20:1727-36.
43. NJ Devlin, PF Krabbe. The development of new research methods for the valuation of EQ-5D-5L. *Eur J Health Econ.* 2013;14 Suppl 1:S1-3.
44. D Kotecha, A Ahmed, M Calvert, M Lencioni, CB Terwee, DA Lane. Patient-reported outcomes for quality of life assessment in atrial fibrillation: A systematic review of measurement properties. *PLoS ONE.* 2016;11:e0165790.

45. European Medicines Agency Committee for Medicinal Products for Human Use. Assessment report: Direct oral anticoagulants (DOACs). *EMA/194375/2020; Procedure number: EMEA/H/A-5(3)/1487*: European Medicines Agency, Amsterdam; 2020.
46. JW Eikelboom, SJ Connolly, J Bosch, GR Dagenais, RG Hart, O Shestakovska, ... C Investigators. Rivaroxaban with or without Aspirin in Stable Cardiovascular Disease. *N Engl J Med.* 2017;377:1319-30.
47. SJ Connolly, J Eikelboom, C Joyner, HC Diener, R Hart, S Golitsyn, ... S Yusuf. Apixaban in patients with atrial fibrillation. *N Engl J Med.* 2011;364:806-17.
48. PW Sullivan, JF Slejko, MJ Sculpher, V Ghushchyan. Catalogue of EQ-5D scores for the United Kingdom. *Med Decis Making.* 2011;31:800-4.
49. B van Hout, MF Janssen, YS Feng, T Kohlmann, J Busschbach, D Golicki, ... AS Pickard. Interim scoring for the EQ-5D-5L: mapping the EQ-5D-5L to EQ-5D-3L value sets. *Value Health.* 2012;15:708-15.
50. AW Chan, JM Tetzlaff, PC Gotzsche, DG Altman, H Mann, JA Berlin, ... D Moher. SPIRIT 2013 explanation and elaboration: guidance for protocols of clinical trials. *BMJ.* 2013;346:e7586.
51. M Calvert, D Kyte, R Mercieca-Bebber, A Slade, AW Chan, MT King, ... T Groves. Guidelines for Inclusion of Patient-Reported Outcomes in Clinical Trial Protocols: The SPIRIT-PRO Extension. *JAMA.* 2018;319:483-94.
52. D Conen. Incidence Of Silent Brain Infarcts In Anticoagulated Patients With Atrial Fibrillation in Swiss-AF Study. *Heart Rhythm Society Late-Breaking Clinical Trials.* 2020:May 09; Virtual Conference.
53. CS Kwok, YK Loke, R Hale, JF Potter, PK Myint. Atrial fibrillation and incidence of dementia: a systematic review and meta-analysis. *Neurology.* 2011;76:914-22.
54. JP Berman, FL Norby, T Mosley, EZ Soliman, RF Gottesman, PL Lutsey, ... LY Chen. Atrial Fibrillation and Brain Magnetic Resonance Imaging Abnormalities. *Stroke.* 2019;50:783-8.
55. J Herm, J Schurig, MR Martinek, R Holtgen, A Schirdewan, P Kirchhof, ... KG Haeusler. MRI-detected brain lesions in AF patients without further stroke risk factors undergoing ablation - a retrospective analysis of prospective studies. *BMC Cardiovasc Disord.* 2019;19:58.
56. D Conen, N Rodondi, A Muller, JH Beer, P Ammann, G Moschovitis, ... AFSI Swiss. Relationships of Overt and Silent Brain Lesions With Cognitive Function in Patients With Atrial Fibrillation. *J Am Coll Cardiol.* 2019;73:989-99.
57. S Kalantarian, TA Stern, M Mansour, JN Ruskin. Cognitive impairment associated with atrial fibrillation: a meta-analysis. *Ann Intern Med.* 2013;158:338-46.
58. J-Y Lee, JP Kim, H Jang, J Kim, SH Kang, JS Kim, ... HJ Kim. Optical coherence tomography angiography as a potential screening tool for cerebral small vessel diseases. *Alzheimer's Research & Therapy.* 2020;12:73.

15 SCHEDULE OF ASSESSMENT

Procedures	Baseline	6 months	12 months	18 months	24 months	30 months	36 months	42 months	48 months	54 months	60 months	// 10 years
Confirmation of selection criteria by the Investigator	X											
Informed consent taken and recorded	X											
Randomisation & allocation through the CPRD IRSP	X											
DOAC prescription by GP for participants randomised to the intervention group	X											
Patient-reported cognitive tests	X		X		X		X		X		X	X
Patient-reported quality of life	X	X	X	X	X	X	X	X	X	X	X	X
Patient self-report of compliance to DOAC therapy		X	X	X	X	X	X	X	X	X	X	
EHR extraction – CPRD			X		X		X		X		X	X
EHR extraction – HES/ONS			X		X		X		X		X	X

Shaded rows are for the participants randomised to DOAC therapy only. Due to a 2-year recruitment window, not all participants will have regular reassessment up to 60 months. 10-year outcome is part of the non-interventional phase of the trial.

CPRD = Clinical Practice Research Datalink; DOAC = Direct oral anticoagulant; EHR = Electronic health record; GP = General Practitioner; IRSP = Interventional Research Services Platform; ONS = Office for National Statistics; HES = Hospital Episode Statistics.
